# Supplementary material for: A prebiotically plausible scenario of an RNA–peptide world
Source: Nature. 2022 May 11;605(7909):279–84. doi: 10.1038/s41586-022-04676-3 (PMC9095488; doi:10.1038/s41586-022-04676-3)
Supplement: Supplementary file 1 — Supplementary text, compounds, figures and references. [file 41586_2022_4676_MOESM1_ESM.pdf]

nature portfolio

https://doi.org/10.1038/s41586-022-04676-3

Supplementary information

A prebiotically plausible scenario of an RNA–peptide world

In the format provided by the authors and unedited

A prebiotically plausible scenario of an RNA-peptide world

Felix Müller[1*], Luis Escobar[1*], Felix Xu[1], Ewa Węgrzyn[1], Milda Nainytė[1], Tynchtyk Amatov[1], Chun-Yin Chan[1], Alexander Pichler[1] and Thomas Carell[1#]

[1] Department of Chemistry, Ludwig-Maximilians-Universität (LMU) München, Butenandtstrasse 5-13, 81377 München, Germany.

* These authors contributed equally.

[#] E-Mail: thomas.carell@lmu.de

Supplementary Information

Table of Contents

1. General information and instruments for phosphoramidites, amino acids and peptides S3
2. Synthesis and characterization data S3
   2.1 Nucleobase-modified 5-methyluridine phosphoramidites S3
   2.2 Npe-protected amino acids and peptides S6
   2.3 Nucleobase-modified *N*[6]-carbamoyl adenosine phosphoramidites..S8
   2.4 Nucleobase-modified *N*[6]-triglycinylcarbamoyl adenosine nucleoside S16
   2.5 Nucleobase-modified *N*[6]-methylurea adenosine nucleoside..S17
   2.6 Nucleobase-modified *N*[6]-triglycinylcarbamoyl adenosine nucleoside under prebiotic conditions S18
   2.7 Nucleobase-modified 5-methyluridine 2'-methoxy phosphoramidite...S20
   2.8 Nucleobase-modified 2'-methoxy *N*[6]-carbamoyl adenosine phosphoramidite S22
3. General information and instruments for oligonucleotides S24
   3.1 Synthesis and purification of oligonucleotides S24
   3.2 Analysis of coupling and cleavage reactions by HPLC and MALDI-TOF mass spectrometry S25
   3.3 Coupling of amino acids and peptides to ONs anchored to the solid support beads S25
4. Synthesized oligonucleotides using a DNA/RNA automated synthesizer S26
   4.1 Canonical oligonucleotides (**CON**) S26
   4.2 Donor oligonucleotides (**ON1**) with a complementary sequence S26
   4.3 Acceptor oligonucleotides (**ON2**) with a complementary sequence S27
   4.4 Donor oligonucleotides with non-complementary sequences S28
5. HPLC calibration curves using canonical oligonucleotides (**CON1**-**6**) and hairpin-type intermediate (**ON3a**) S28
6. Coupling reactions between donor and acceptor oligonucleotides, **ON1** and **ON2** S31
   6.1 Control experiments S31
   6.2 Screening of activators using **ON1a** (m[6]g[6]A) and **ON2a** (mnm[5]U)...S31
   6.3 Screening of activators using **ON1a** (m[6]g[6]A) and **ON2b** (nm[5]U) S33
   6.4 Screening of activators using **ON1a** (m[6]g[6]A) and **ON2c** (vmnm[5]U)..S34
   6.5 Coupling reactions of **ON1j** (m[6]g[6]A, amino nitrile) with **ON2a**-**c** S35
   6.6 Coupling reactions of **ON1b**-**i** (m[6]aa[6]A) with **ON2a** S37
   6.7 Coupling reactions of **ON1b**-**i** (m[6]aa[6]A) with **ON2c** S41
7. Synthesized peptide-oligonucleotides using solid support beads S45
   7.1 Donor peptide-oligonucleotides with a complementary sequence S45
   7.2 Acceptor peptide-oligonucleotides with a complementary sequence S46
8. Coupling reactions between donor and acceptor peptide-oligonucleotides...S47
   8.1 Coupling reactions of donor peptide-oligonucleotides with **ON2c**..S47

8.2 Coupling reactions of **ON1a** (m^{6}g^{6}A) with acceptor peptide-oligonucleotides S49
8.3 Coupling reactions of donor and acceptor peptide-oligonucleotides S51
9. Concentration of the product versus time in selected coupling reactions S52
10. Coupling reactions between oligonucleotides containing multiple donor or acceptor units S53
11. Coupling reactions between **ON2c** and donor oligonucleotides with non-complementary sequences . S55
12. Coupling reactions between **ON2c** and donor oligonucleotides with different lengths S56
13. Stability of selected acceptor oligonucleotides (**ON2**) S58
14. Cleavage of urea in selected oligonucleotides and cyclic peptide products S59
14.1 Cleavage reactions of **ON1c** (m^{6}v^{6}A) and **ON1k** (v^{6}A) at pH 5 .... S59
14.2 Cleavage reaction of **ON3a** (m^{6}g^{6}A coupled with mnm^{5}U) S60
14.3 Cleavage reactions of **ON3c** (m^{6}g^{6}A coupled with vmnm^{5}U) S62
14.4 Cleavage reactions of peptide-oligonucleotides at pH 4 .... S65
15. Coupling and cleavage reactions between donor and acceptor oligonucleotides containing 2'-OMe nucleosides S68
15.1 Coupling and cleavage reactions of **ON1a** (m^{6}g^{6}A) with **ON2g** S68
15.2 Coupling and cleavage reactions of **ON1o** (m^{6}g^{6}Am) with **ON2h** S71
15.3 Coupling and cleavage reactions of donor and acceptor-peptide oligonucleotides S73
15.4 Coupling reactions between **ON2g** and donor oligonucleotides of different length S74
16. Determination of melting temperatures by UV spectroscopic experiments S76
16.1 Melting temperature of a double strand from canonical oligonucleotides S76
16.2 Melting temperatures of double strands from donor and acceptor oligonucleotides S76
16.3 Melting temperatures of double strands from donor and acceptor peptide-oligonucleotides S78
16.4 Melting temperatures of selected cyclic peptide products S79
17. NMR spectra of synthesized compounds S81
18. References S156

1. General information and instruments for phosphoramidites, amino acids and peptides

Reagents were purchased from commercial suppliers and used without further purification unless otherwise stated. All anhydrous solvents stored under inert atmosphere were also purchased. All reactions involving air/moisture sensitive reagents/intermediates were performed under inert atmosphere using oven-dried glassware. Routine ^{1}H NMR, $^{13}C\{^{1}H\}$ NMR and $^{31}P\{^{1}H\}$ NMR were recorded on a Bruker Ascend 400 spectrometer (400 MHz for ^{1}H NMR, 100 MHz for ^{13}C NMR and 162 MHz for ^{31}P NMR) or a Bruker ARX 600 spectrometer (600 MHz for ^{1}H NMR, 150 MHz for ^{13}C NMR and 243 MHz for ^{31}P NMR). Deuterated solvents used are indicated in the characterization and chemical shifts (δ) are reported in ppm. Residual solvent peaks were used as reference.[1] All NMR J values are given in Hz. COSY, HMQC and HMBC experiments were recorded to help with the assignment of ^{1}H and ^{13}C signals. NMR spectra were analyzed using MestReNova software version 10.0. High Resolution Mass Spectra (HRMS) were measured on a Thermo Finnigan LTQ-FT with ESI as ionization mode. IR spectra were recorded on a Perkin-Elmer Spectrum BX II FT-IR instrument equipped with an ATR accessory. Column chromatography was performed with silica gel technical grade (Macherey-Nagel), 40-63 µm particle size. Reaction progress was monitored by Thin Layer Chromatography (TLC) analysis on silica gel 60 F254 and stained with *para*-anisaldehyde, potassium permanganate or cerium ammonium molybdate solution.

2. Synthesis and characterization data

2.1 Nucleobase-modified 5-methyluridine phosphoramidites

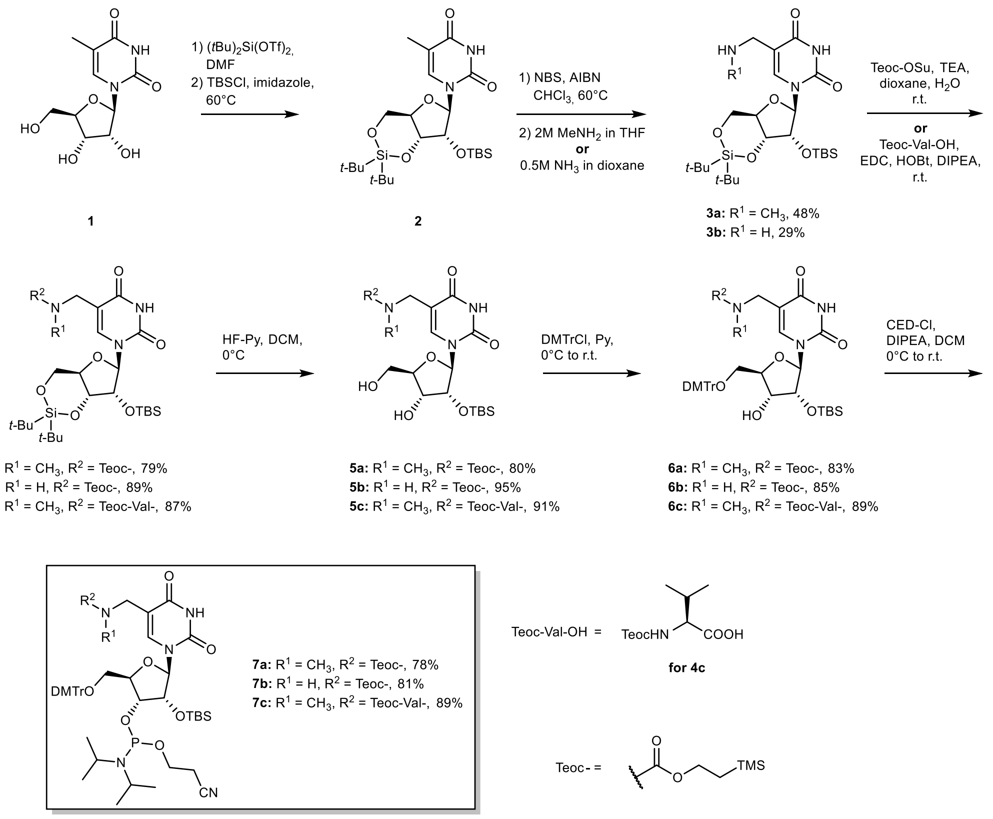

Scheme S1. Synthesis of nucleobase-modified 5-methyluridine phosphoramidites.

General procedure for the synthesis of 3a,b:

Silyl-protected 5-methyluridine **2** was synthesized starting from 5-methyluridine **1** following a procedure previously described in literature.[2] A solution of **2** (1.0 equiv.) in dry $CHCl_3$ was heated at 60°C. *N*-bromosuccinimide (NBS) (1.2 equiv., previously purified by recrystallization) and azobisisobutyronitrile (AIBN) (0.12 equiv.) were added and the reaction was stirred under reflux for 1.5 h. After that, the reaction mixture was cooled to r.t. and either $MeNH_2$ (2 M in THF, 5.0 equiv.) for **3a** or NH_3 (0.5 M in 1,4-dioxane, 5.0 equiv.) for **3b** were added. The resulting suspension was stirred for 2 h at r.t. and, subsequently, it was diluted with aq. sat. $NaHCO_3$ solution. The crude was extracted three times with DCM. The combined organic layers were dried ($MgSO_4$), filtered and concentrated. The crude was purified by silica gel column chromatography to furnish **3a**,**b** as a yellow foam.

3a: Yield: 48%; R_f = 0.11 (9:1 DCM/MeOH); IR (ATR) $\tilde{\nu}$ (cm^{-1}): 2931 (w), 2858 (w), 2359 (w), 1682 (s), 1462 (m), 1386 (w), 1254 (m), 1202 (w), 1167 (w), 1115 (m), 1057 (s), 1000 (m), 938 (w), 882 (m), 827 (s), 778 (s), 754 (m), 685 (w); ^{1}H NMR (400 MHz, $CDCl_3$, 298 K) δ (ppm): 7.35 (s, 1H), 5.66 (s, 1H), 4.47 (dd, J = 9.5, 4.7 Hz, 1H), 4.28 (d, J = 4.7 Hz, 1H), 4.18-4.06 (m, 1H), 4.05-3.97 (m, 1H), 3.92 (dd, J = 9.5, 4.7 Hz, 1H), 3.58-3.47 (m, 2H), 2.41 (s, 3H), 1.03 (s, 9H), 1.01 (s, 9H), 0.91 (s, 9H), 0.15 (s, 3H), 0.12 (s, 3H); ^{13}C{^{1}H} NMR (100 MHz, $CDCl_3$, 298 K) δ (ppm): 164.2, 150.1, 138.1, 111.1, 94.1, 76.1, 75.3, 74.6, 67.7, 47.6, 35.0, 27.6, 27.1, 26.0, 22.9, 20.5, 18.4, -4.2, -4.9; HRMS (ESI) m/z: [M+H]$^+$ Calcd. for $C_{25}H_{48}N_3O_6Si_2$ 542.3076; Found 542.3076.

3b: Yield: 29%; R_f = 0.25 (100:5 DCM/MeOH); IR (ATR) $\tilde{\nu}$ (cm^{-1}): 3052 (w), 2934 (w), 2858 (w), 2363 (w), 1687 (m), 1471 (w), 1422 (w), 1388 (w), 1264 (s), 1204 (w), 1168 (w), 1115 (m), 1059 (m), 999 (m), 938 (w), 896 (w), 882 (m), 828 (s), 780 (m), 731 (s), 702 (s); ^{1}H NMR (400 MHz, $CDCl_3$, 298 K) δ (ppm): 7.29 (s, 1H), 5.69 (s, 1H), 4.49 (dd, J = 9.1, 5.0 Hz, 1H), 4.28 (d, J = 4.8 Hz, 1H), 4.18-4.08 (m, 1H), 4.01 (dd, J = 10.6, 9.1 Hz, 1H), 3.92 (dd, J = 9.5, 4.8 Hz, 1H), 3.60 (s, 2H), 1.05 (s, 9H), 1.02 (s, 9H), 0.92 (s, 9H), 0.16 (s, 3H), 0.13 (s, 3H); ^{13}C{^{1}H} NMR (100 MHz, $CDCl_3$, 298 K) δ (ppm): 163.2, 149.6, 136.3, 94.1, 76.2, 75.4, 74.6, 67.7, 39.2, 27.6, 27.1, 26.0, 22.9, 20.5, 18.4, -4.2, -4.9; HRMS (ESI) m/z: [M+H]$^+$ Calcd. for $C_{24}H_{46}N_3O_6Si_2$ 528.2920; Found 528.2921.

General procedures for the synthesis of 4a-c:

Procedure A (for compounds 4a,b): To a solution of **3a**,**b** (1.0 equiv.) in 1,4-dioxane and H_2O (1:1 v/v) were added teoc-OSu (1.1 equiv.) and triethylamine (TEA) (1.5 equiv.). The mixture was stirred at r.t. for 16 h. After that, the crude was diluted with water and extracted three times with Et_2O. The combined organic layers were washed with water, dried ($MgSO_4$), filtered and concentrated. The obtained residue was purified by silica gel column chromatography to yield the teoc-protected compound **4a,b** as a white solid.

Procedure B (for compound 4c): Teoc-protected valine was synthesized following a previously reported procedure in literature.[3] Teoc-Val-OH (1.2 equiv.) was dissolved in dry DCM and DMF (99:1 v/v). To the solution, 1-hydroxybenzotriazole hydrate (HOBt•H_2O) (1.2 equiv.), 1-ethyl-3-(3-dimethylaminopropyl)carbodiimide hydrochloride (EDC•HCl) (1.2 equiv.) and *N*,*N*-diisopropylethylamine (DIPEA) (1.2 equiv.) were added. After stirring at r.t. for 30 min, a solution of **3a** (1.0 equiv.) in DCM was added and the reaction was stirred for 24 h. The reaction mixture was extracted three times with DCM. The combined organic layers were dried ($MgSO_4$), filtered and concentrated. Purification by silica gel column chromatography furnished the amino acid conjugate **4c** as a white foam.

4a: Yield: 79%; R_f = 0.34 (4:1 *i*-Hexane/EtOAc); IR (ATR) $\tilde{\nu}$ (cm^{-1}): 3054 (w), 2956 (w), 2359 (w), 1692 (m), 1463 (w), 1422 (w), 1264 (s), 1214 (w), 1167 (w), 1146 (w), 1059 (w), 1000 (w), 938 (w), 895 (m), 838 (m), 730 (s), 702 (s); ^{1}H NMR (400 MHz, $CDCl_3$, 298 K) δ (ppm): 9.14 (s, 1H), 7.54 (s, 1H), 5.65 (s, 1H), 4.48 (dd, J = 9.2, 4.2 Hz, 1H), 4.28 (d, J = 4.2 Hz, 1H), 4.23-3.98 (m, 6H), 3.91 (dd, J = 9.2, 4.2 Hz, 1H), 2.96 (s, 3H), 1.05 (s, 9H), 1.03-0.96 (m, 11H), 0.93 (s, 9H), 0.18 (s, 3H), 0.13 (s, 3H), 0.04 (s, 9H); ^{13}C{^{1}H} NMR (100 MHz, $CDCl_3$, 298 K) δ (ppm): 163.6, 157.1, 149.7, 139.4, 110.8, 93.8, 76.0, 75.5, 74.9, 67.6, 63.8, 45.0, 35.6, 27.7, 27.1, 26.0, 22.8, 20.5, 18.4, 17.9, -1.3, -4.2, -4.9; HRMS (ESI) m/z: [M+H]$^+$ Calcd. for $C_{31}H_{60}N_3O_8Si_3$ 686.3683; Found 686.3683.

4b: Yield: 89%; R_f = 0.23 (4:1 *i*-Hexane/EtOAc); IR (ATR) $\tilde{\nu}$ (cm^{-1}): 2937 (w), 2359 (w), 2167 (w), 1690 (m), 1470 (w), 1251 (m), 1213 (w), 1127 (w), 1061 (m), 999 (m), 831 (m), 779 (m), 730 (s); ^{1}H NMR (400 MHz, $CDCl_3$, 298 K): δ (ppm) 8.13 (s, 1H), 7.46 (s, 1H), 5.65 (s, 1H), 5.23 (t, J = 5.9 Hz, 1H), 4.50 (dd, J = 9.0, 4.9 Hz, 1H), 4.28 (d, J = 4.6 Hz, 1H), 4.20-4.05 (m, 4H), 3.98 (d, J = 6.3 Hz, 2H), 3.90 (dd, J = 9.5, 4.6 Hz, 1H), 1.06 (s, 9H), 1.02 (s, 9H), 0.99-0.88 (m, 11H), 0.18 (s, 3H), 0.14 (s, 3H), 0.03 (s, 9H); ^{13}C{^{1}H} NMR (100 MHz, $CDCl_3$, 298 K) δ (ppm): 162.9, 156.9, 149.4, 138.4, 111.4, 93.9, 76.0, 75.5, 74.9, 67.6, 63.4, 37.7, 27.7, 27.1, 26.0, 22.9, 20.5, 18.4, 17.8, -1.3, -4.1, -4.9; HRMS (ESI) m/z: [M+H]$^+$ Calcd. for $C_{30}H_{58}N_3O_8Si_3$ 672.3526; Found 672.3535.

4c: Yield: 87%; R_f = 0.29 (100:5 DCM/MeOH); IR (ATR) $\tilde{\nu}$ (cm^{-1}): 3053 (w), 2956 (w), 2859 (w), 2359 (w), 1689 (m), 1648 (w), 1586 (w), 1536 (w), 1471 (m), 1382 (w), 1366 (m), 1311 (w), 1264 (s), 1168 (w), 1114 (m), 1059 (m), 1002 (w), 938 (w), 835 (m), 732 (s), 702 (s); For major rotamer: ^{1}H NMR (400 MHz, $CDCl_3$, 298 K) δ (ppm): 9.22 (s, 1H), 7.66 (s, 1H), 5.69 (s, 1H), 5.40 (d, J = 9.0 Hz, 1H), 4.51-4.42 (m, 2H), 4.25 (d, J = 14.3 Hz, 1H), 4.21-4.01 (m, 6H), 3.97 (dd, J = 9.0, 4.8 Hz, 1H), 3.21 (s, 3H), 1.92-1.87 (m, 1H), 1.09 (s, 9H), 1.05-0.98 (m, 11H), 0.94-0.89 (m, 12H), 0.80 (d, J = 6.7 Hz, 3H), 0.14 (s, 3H), 0.11 (s, 3H), 0.02 (s, 9H); ^{13}C{^{1}H} NMR (100 MHz, $CDCl_3$, 298 K) δ (ppm): 172.7, 163.6, 157.0, 149.6, 141.3, 110.1, 93.9, 76.1, 75.6, 74.8, 67.6, 63.4, 55.4, 44.5, 37.3, 31.3, 27.7, 27.1, 26.0, 22.8, 20.5, 19.6, 18.4, 17.8, 17.1, -1.3, -4.2, -5.0; HRMS (ESI) m/z: [M+H]$^+$ Calcd. for $C_{36}H_{69}N_4O_9Si_3$ 785.4367; Found 785.4363.

General procedure for the synthesis of 5a-c:

The modified 5-methyluridine **4a-c** (1.0 equiv.) was dissolved in DCM/pyridine (9:1 v/v) and cooled to 0°C in a plastic reaction vessel. Subsequently, a solution of 70% HF-pyridine (5.0 equiv.) was slowly added, and the reaction mixture was stirred at 0°C for 2 h. The reaction was quenched by adding aq. sat. $NaHCO_3$ and the crude was extracted three times with DCM. The combined organic layers were washed with water, dried ($MgSO_4$), filtered and concentrated. The crude product was purified by silica gel column chromatography to afford the diol compound **5a-c** as a white foam.

5a: Yield: 80%; R_f = 0.42 (100:5 DCM/MeOH); IR (ATR) $\tilde{\nu}$ (cm^{-1}): 3417 (w), 3060 (w), 2949 (w), 2856 (w), 2359 (w), 1673 (s), 1462 (m), 1401 (w), 1362 (w), 1250 (m), 1214 (w), 1144 (m), 1088 (m), 1060 (m), 1005 (w), 938 (w), 833 (s), 777 (s), 693 (w); ^{1}H NMR (400 MHz, $CDCl_3$, 298 K) δ (ppm): 9.47 (s, 1H), 8.19 (s, 1H), 5.87 (d, *J* = 5.2 Hz, 1H), 4.48 (t, *J* = 5.1 Hz, 1H), 4.30-3.85 (m, 7H), 3.83-3.74 (m, 1H), 2.97 (s, 3H), 2.78 (br s, 1H), 1.03-0.92 (m, 2H), 0.88 (s, 9H), 0.06 (s, 6H), 0.02 (s, 9H) (some proton signals appeared too broad for an unequivocal assignment); ^{13}C{^{1}H} NMR (100 MHz, $CDCl_3$, 298 K) δ (ppm): 163.8, 157.4, 150.5, 141.9, 111.4, 90.2, 85.8, 75.3, 71.3, 64.1, 62.2, 44.5, 35.6, 25.8, 18.1, -1.4, -4.7 (some carbon signals appeared too broad for an unequivocal assignment); HRMS (ESI) *m*/*z*: $[M+H]^+$ Calcd. for $C_{23}H_{44}N_3O_8Si_2$ 546.2661; Found 546.2666.

5b: Yield: 95%; R_f = 0.23 (100:5 DCM/MeOH); IR (ATR) $\tilde{\nu}$ (cm^{-1}): 3386 (w), 2950 (w), 2854 (w), 2362 (w), 1674 (s), 1524 (m), 1470 (m), 1390 (w), 1333 (w), 1248 (s), 1179 (w), 1115 (m), 1086 (w), 1060 (s), 1001 (w), 938 (w), 902 (w), 857 (m), 833 (s), 779 (s), 694 (w); ^{1}H NMR (400 MHz, $CDCl_3$, 298 K) δ (ppm): 8.51 (s, 1H), 8.09 (s, 1H), 5.80 (s, 1H), 5.35 (t, *J* = 6.2 Hz, 1H), 4.49 (t, *J* = 4.8 Hz, 1H), 4.33-4.22 (m, 1H), 4.17-4.05 (m, 4H), 4.02-3.92 (m, 3H), 3.81 (dd, *J* = 12.0, 5.3 Hz, 1H), 3.57 (t, *J* = 5.3 Hz, 1H), 2.70 (d, *J* = 4.3 Hz, 1H), 0.99-0.92 (m, 3H), 0.90 (s, 9H), 0.11-0.08 (m, 6H), 0.02 (s, 9H); ^{13}C{^{1}H} NMR (100 MHz, $CDCl_3$, 298 K) δ (ppm): 163.0, 157.2, 150.2, 141.0, 111.8, 90.9, 85.7, 75.1, 71.0, 63.6, 62.1, 37.2, 25.8, 18.1, 17.8, -1.3, -4.6, -5.0; HRMS (ESI) *m*/*z*: $[M+H]^+$ Calcd. for $C_{22}H_{42}N_3O_8Si_2$ 532.2505; Found 532.2509.

5c: Yield: 91%; R_f = 0.18 (100:5 DCM/MeOH); IR (ATR) $\tilde{\nu}$ (cm^{-1}): 3440 (w), 3054 (w), 2953 (w), 2857 (w), 2359 (w), 1677 (s), 1463 (m), 1401 (w), 1362 (w), 1264 (s), 1250 (m), 1215 (w), 1137 (m), 1112 (w), 1089 (w), 1060 (w), 1005 (w), 937 (w), 836 (s), 779 (m), 733 (s), 701 (s); For major rotamer: ^{1}H NMR (400 MHz, $CDCl_3$, 298 K) δ (ppm): 8.80 (s, 1H), 8.06 (s, 1H), 5.94 (d, *J* = 5.0 Hz, 1H), 5.45 (d, *J* = 9.9 Hz, 1H), 4.59 (d, *J* = 15.0 Hz, 1H), 4.49 (dd, *J* = 9.9, 5.4 Hz, 1H), 4.43-4.05 (m, 5H), 3.91 (d, *J* = 15.0 Hz, 2H), 3.80 (d, *J* = 12.0 Hz, 1H), 3.19 (s, 3H), 2.71 (d, *J* = 3.1 Hz, 1H), 2.01-1.94 (m, 1H), 1.01-0.93 (m, 5H), 0.93-0.85 (m, 13H), 0.07 (s, 3H), 0.06 (s, 3H), 0.03 (s, 9H) (some proton signals appeared too broad for an unequivocal assignment); ^{13}C{^{1}H} NMR (100 MHz, $CDCl_3$, 298 K) δ (ppm): 172.9, 163.2, 157.5, 150.2, 139.8, 110.0, 89.5, 85.7, 75.9, 71.3, 63.9, 61.8, 55.8, 44.5, 36.7, 31.0, 25.8, 19.7, 18.1, 17.9, 17.1, -1.4, -4.7, -5.1 (some carbon signals appeared too broad for an unequivocal assignment); HRMS (ESI) *m*/*z*: $[M+H]^+$ Calcd. for $C_{28}H_{53}N_4O_9Si_2$ 645.3346; Found 645.3349.

General procedure for the synthesis of 6a-c:

To a solution of the 3',5'-deprotected 5-methyluridine derivative **5a-c** (1.0 equiv.) in pyridine was added 4,4'-dimethoxytrityl chloride (DMTrCl) (1.5 equiv.). After stirring at r.t. for 16 h, the reaction mixture was concentrated and purified by silica gel column chromatography with an addition of 0.1% of pyridine to the eluent to afford the DMTr-protected compound **6a-c** as a white foam.

6a: Yield: 83%; R_f = 0.57 (1:1 *i*-Hexane/EtOAc); IR (ATR) $\tilde{\nu}$ (cm^{-1}): 3444 (w), 3055 (w), 2953 (w), 2857 (w), 2359 (w), 1678 (s), 1608 (w), 1583 (w), 1508 (m), 1463 (m), 1401 (w), 1342 (w), 1297 (w), 1264 (m), 1248 (s), 1175 (m), 1150 (m), 1113 (w), 1089 (w), 1034 (m), 1006 (w), 938 (w), 910 (w), 830 (s), 780 (m), 733 (s), 701 (s); For major rotamer: ^{1}H NMR (400 MHz, acetone-d_6, 298 K) δ (ppm): 10.21 (s, 1H), 7.76 (s, 1H), 7.57-7.46 (m, 2H), 7.45-7.37 (m, 4H), 7.37-7.29 (m, 2H), 7.28-7.19 (m, 1H), 6.90 (d, *J* = 8.9 Hz, 4H), 5.94 (s, 1H), 4.44 (br s, 1H), 4.22-4.03 (m, 3H), 3.84-3.71 (m, 8H), 3.44 (br s, 2H), 2.90 (br s, 3H), 1.08-0.81 (m, 11H), 0.15 (s, 6H), 0.03 (s, 9H) (some proton signals appeared too broad for an unequivocal assignment); ^{13}C{^{1}H} NMR (100 MHz, acetone-d_6, 298 K) δ (ppm): 163.8, 159.6, 156.7, 151.2, 146.1, 136.7, 131.1, 131.0, 129.0, 128.7, 114.0, 114.0, 113.6, 111.4, 89.7, 87.3, 84.3, 76.5, 71.5, 64.6, 63.6, 55.5, 46.5, 35.7, 26.2, 18.7, 18.3, -1.4, -4.6, -4.6 (some carbon signals appeared too broad for an unequivocal assignment); HRMS (ESI) *m*/*z*: $[M-H]^-$ Calcd. for $C_{44}H_{60}N_3O_{10}Si_2$ 846.3823; Found 846.3825.

6b: Yield: 85%; R_f = 0.33 (2:1 *i*-Hexane/EtOAc); IR (ATR) $\tilde{\nu}$ (cm^{-1}): 3342 (w), 2950 (w), 2855 (w), 2358 (w), 1708 (s), 1607 (w), 1582 (w), 1508 (m), 1462 (m), 1390 (w), 1248 (s), 1175 (m), 1116 (m), 1089 (w), 1063 (m), 1035 (m), 969 (w), 937 (w), 859 (m), 835 (s), 780 (s), 754 (m), 726 (w), 699 (m); ^{1}H NMR (400 MHz, acetone-d_6, 298 K) δ (ppm): 10.18 (s, 1H), 7.78 (s, 1H), 7.52 (d, *J* = 7.6 Hz, 2H), 7.41 (d, *J* = 8.8 Hz, 4H), 7.34 (t, *J* = 7.6 Hz, 2H), 7.24 (t, *J* = 7.6 Hz, 1H), 6.92 (d, *J* = 8.8 Hz, 4H), 6.05 (t, *J* = 5.0 Hz, 1H), 5.95 (d, *J* = 4.6 Hz, 1H), 4.46 (t, *J* = 5.0 Hz, 1H), 4.28-4.22 (m, 1H), 4.17-4.12 (m, 1H), 4.11-4.01 (m, 2H), 3.82 (d, *J* = 5.8 Hz, 1H), 3.79 (s, 6H), 3.64 (dd, *J* = 14.5, 5.5 Hz, 1H), 3.56 (dd, *J* = 14.5, 5.9 Hz, 1H), 3.45 (dd, *J* = 10.8, 4.2 Hz, 1H), 3.39 (dd, *J* = 10.8, 2.5 Hz, 1H),

0.97-0.83 (m, 11H), 0.15 (s, 3H), 0.14 (s, 3H), 0.02 (s, 9H); $^{13}C\{^1H\}$ NMR (100 MHz, acetone-d_6, 298 K) δ (ppm): 163.5, 159.6, 157.0, 151.2, 146.0, 138.9, 136.7, 136.6, 131.0, 129.0, 128.8, 127.6, 114.0, 112.2, 89.6, 87.4, 84.3, 76.7, 71.6, 64.4, 62.8, 55.5, 38.5, 26.2, 18.7, 18.4, -1.4, -4.6, -4.7; HRMS (ESI) *m/z*: $[M+H]^+$ Calcd. for $C_{43}H_{60}N_3O_{10}Si_2$ 834.3812; Found 834.3801.

6c: Yield: 89%; R_f = 0.42 (1:1 *i*-Hexane/EtOAc); IR (ATR) $\tilde{\nu}$ (cm^{-1}): 3054 (w), 2954 (w), 2930 (w), 2857 (w), 2359 (w), 1687 (s), 1644 (w), 1608 (w), 1508 (m), 1463 (m), 1389 (w), 1263 (m), 1249 (s), 1175 (m), 1115 (w), 1083 (w), 1061 (w), 1035 (m), 967 (w), 935 (w), 914 (w), 858 (m), 833 (s), 780 (w), 733 (s), 700 (s); For major rotamer: ^1H NMR (400 MHz, $CDCl_3$, 298 K) δ (ppm): 9.10 (s, 1H), 7.75 (s, 1H), 7.45 (d, J = 7.3 Hz, 2H), 7.35 (d, J = 8.8 Hz, 4H), 7.31-7.24 (m, 2H), 7.20 (t, J = 7.3 Hz, 1H), 6.82 (d, J = 8.8 Hz, 4H), 5.88 (d, J = 3.8 Hz, 1H), 5.41 (d, J = 8.9 Hz, 1H), 4.43-4.37 (m, 1H), 4.34-4.29 (m, 1H), 4.18-4.07 (m, 4H), 4.00 (d, J = 14.3 Hz, 1H), 3.77 (s, 6H), 3.54-3.40 (m, 3H), 3.11 (s, 3H), 2.59 (d, J = 6.3 Hz, 1H), 1.92-1.78 (m, 1H), 1.04-0.96 (m, 2H), 0.94-0.86 (m, 12H), 0.79 (d, J = 6.7 Hz, 3H), 0.12 (s, 3H), 0.11 (s, 3H), 0.03 (s, 9H) (some proton signals appeared too broad for an unequivocal assignment); $^{13}C\{^1H\}$ NMR (100 MHz, $CDCl_3$, 298 K) δ (ppm): 172.0, 163.2, 158.7, 156.9, 150.0, 149.8, 144.8, 141.2, 136.2, 135.8, 130.4, 130.3, 128.3, 128.0, 127.0, 123.9, 113.3, 110.3, 89.9, 86.8, 83.7, 75.6, 70.8, 63.6, 63.3, 55.3, 45.5, 37.1, 31.4, 25.8, 19.6, 18.1, 17.9, 17.3, -1.4, -4.5, -5.1 (some carbon signals appeared too broad for an unequivocal assignment); HRMS (ESI) *m/z*: $[M-H]^-$ Calcd. for $C_{49}H_{69}N_4O_{11}Si_2$ 945.4507; Found 945.4508.

General procedure for the synthesis of phosphoramidites 7a-c:

A solution of 5'-DMTr-protected compound **6a**-**c** (1.0 equiv.) and DIPEA (4.0 equiv.) in dry DCM was cooled to 0°C. To this solution was slowly added 2-cyanoethyl *N,N*-diisopropylchlorophosphoramidite (CED-Cl) (2.5 equiv.) and the reaction mixture was stirred at r.t. for 5 h. The reaction was quenched by addition of aq. sat. $NaHCO_3$ and the crude was extracted three times with DCM. The combined organic layers were dried ($MgSO_4$), filtered and concentrated under reduced pressure. After purification by silica gel column chromatography with an addition of 0.1% pyridine and co-lyophilization from benzene, the desired phosphoramidite **7a**-**c** was obtained as a mixture of diastereoisomers and rotamers as a white foam.

7a: Yield: 78%; R_f = 0.17 (2:1 *i*-Hexane/EtOAc); $^{31}P\{^1H\}$ NMR (162 MHz, acetone-d_6, 298 K) δ (ppm): 150.3, 150.2, 148.8, 148.3; HRMS (ESI) *m/z*: $[M-H]^-$ Calcd. for $C_{53}H_{77}N_5O_{11}PSi_2$ 1046.4901, Found 1046.4896.

7b: Yield: 81%; R_f = 0.57 (1:1 *i*-Hexane/EtOAc); $^{31}P\{^1H\}$ NMR (162 MHz, acetone-d_6, 298 K): δ (ppm): 150.2, 148.3; HRMS (ESI) *m/z*: $[M-H]^-$ Calcd. for $C_{52}H_{77}N_5O_{11}PSi_2$ 1032.4744; Found 1032.4745.

7c: Yield: 89%; R_f = 0.45 (1:1 *i*-Hexane/EtOAc); $^{31}P\{^1H\}$ NMR (162 MHz, acetone-d_6, 298 K) δ (ppm): 150.0, 149.9, 149.4, 149.3; HRMS (ESI) *m/z*: $[M-H]^-$ Calcd. for $C_{58}H_{86}N_6O_{12}PSi_2$ 1145.5585; Found 1145.5595.

2.2 Npe-protected amino acids and peptides

The npe-protected amino acids Gly, Val, Thr, Phe and Asp were synthesized following previously reported procedures in the literature.[4]

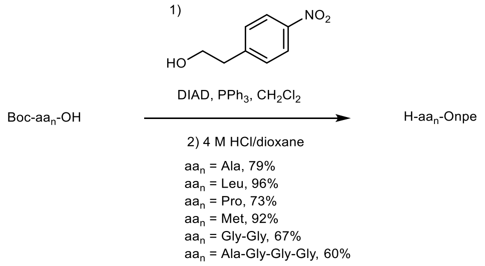

Scheme S2. Synthesis of npe-protected amino acids and peptides.

Procedure A (for Ala, Leu, Pro and Met): Step 1. **Boc-aa-OH** (1.0 equiv.), 2-(4-nitrophenyl)ethanol (1.3 equiv.) and PPh_3 (1.3 equiv.) were dissolved in dry CH_2Cl_2 and stirred at 0ºC under nitrogen atmosphere. Diisopropyl azodicarboxylate (DIAD) (1.3 equiv.) was added dropwise and the reaction was stirred at r.t. overnight. Afterwards, the reaction was stopped and the crude was washed two times with water. The organic layer was dried (Na_2SO_4), filtered and concentrated *in vacuo*. The crude was purified by silica gel column chromatography affording the **Boc-aa-Onpe** as a white solid. Step 2. **Boc-aa-Onpe** (1.0 equiv.) was dissolved in 4 M HCl in 1,4-dioxane at 0ºC. After the reaction was stirred at r.t. for 1 h, the mixture was concentrated obtaining a white solid. The white solid was triturated with Et_2O, filtered and washed with additional Et_2O. The npe-protected amino acid **H-aa-Onpe** chloride salt was isolated as a white solid. For **Pro**, an oil was obtained which was washed with aq. sat. $NaHCO_3$ and the

crude was extracted with EtOAc. The organic layer was dried (Na_2SO_4), filtered and concentrated under reduced pressure. The crude was purified by silica gel column chromatography affording the **H-Pro-Onpe** as a pale-yellow oil.

H-Ala-Onpe•HCl: Yield: 79% over two steps; IR (ATR) $\tilde{\nu}$ (cm^{-1}): 2843 (m), 1730 (s), 1598 (m), 1345 (s), 1269 (w), 1233 (s), 1195 (m), 1115 (m), 820 (m), 746 (m); ^1H NMR (400 MHz, DMSO-d_6, 298 K): δ (ppm): 8.59 (br s, 3H), 8.19-8.17 (m, 2H), 7.61-7.59 (m, 2H), 4.50-4.36 (m, 2H), 4.00 (q, J = 7.2 Hz, 1H), 3.10 (t, J = 6.3 Hz, 2H), 1.33 (d, J = 7.2 Hz, 3H); $^{13}C\{^1H\}$ NMR (100 MHz, DMSO-d_6, 298 K) δ (ppm): 169.9, 146.4, 146.3, 130.4, 123.5, 65.3, 47.8, 33.9, 15.7; HRMS (ESI) m/z: $[M+H]^+$ Calcd. for $C_{11}H_{15}N_2O_4$: 239.1026; Found 239.1027.

H-Leu-Onpe•HCl: Yield: 96% over two steps; IR (ATR) $\tilde{\nu}$ (cm^{-1}): 3663 (w), 2871 (m), 1737 (s), 1589 (m), 1516 (s), 1503 (s), 1380 (s), 1260 (w), 1207 (m), 1109 (w), 959 (w), 856 (m), 812 (m), 735 (s); ^1H NMR (400 MHz, DMSO-d_6, 298 K) δ (ppm): 8.63 (s, 3H), 8.17 (d, J = 8.7 Hz, 2H), 7.60 (d, J = 8.7 Hz, 2H), 4.51-4.38 (m, 2H), 3.82 (t, J = 6.5 Hz, 1H), 3.11 (t, J = 6.5 Hz, 2H), 1.54-1.43 (m, 3H), 0.75 (t, J = 5.3 Hz, 6H); $^{13}C\{^1H\}$ NMR (100 MHz, DMSO-d_6, 298 K) δ (ppm): 169.8, 146.4, 130.4, 123.4, 65.3, 50.4, 33.8, 23.7, 22.2, 21.8; HRMS (ESI) m/z: $[M+H]^+$ Calcd. for $C_{14}H_{21}N_2O_4$ 281.1496; Found 281.1495.

H-Pro-Onpe: Yield: 73% over two steps; R_f = 0.30 (9:1 CH_2Cl_2/IPA); IR (ATR) $\tilde{\nu}$ (cm^{-1}): 3400 (w), 2879 (w), 1649 (s), 1513 (s), 1432 (m), 1318 (s), 1159 (w), 1048 (m), 856 (m), 747 (m); ^1H NMR (400 MHz, $CDCl_3$, 298 K) δ (ppm): 8.18-8.16 (m, 2H); 7.40-7.38 (m, 2H); 4.40-4.37 (m, 2H); 3.74-3.71 (m, 1H); 3.07 (t, J = 6.7 Hz, 2H); 3.05-2.87 (m, 2H); 2.29 (br s, 1H); 2.13-2.02 (m, 1H); 1.77-1.67 (m, 3H); $^{13}C\{^1H\}$ NMR (100 MHz, $CDCl_3$, 298 K) δ (ppm): 175.4, 147.0, 145.6, 129.9, 123.9, 64.3, 59.8, 47.1, 35.0, 30.4, 25.6; HRMS (ESI) m/z: $[M+H]^+$ Calcd. for $C_{13}H_{17}N_2O_4$ 265.1183; Found 265.1179.

H-Met-Onpe•HCl: Yield: 92% over two steps; IR (ATR) $\tilde{\nu}$ (cm^{-1}): 2852 (w), 1756 (m), 1743 (m), 1598 (w), 1567 (w), 1509 (s), 1347 (s), 1279 (m), 1256 (w), 1230 (w), 1206 (m), 1194 (m), 1148 (w), 1109 (w), 1066 (m), 1000 (w), 856 (m), 827 (m), 793 (w), 769 (m), 744 (s), 694 (m); ^1H NMR (400 MHz, DMSO-d_6, 298 K) δ (ppm): 8.77 (s, 3H), 8.18-8.15 (m, 2H), 7.62-7.59 (m, 2H), 4.50-4.41 (m, 2H), 4.01 (br s, 1H), 3.11 (t, J = 6.3 Hz, 2H), 2.55-2.43 (m, 1H), 2.38-2.27 (m, 1H), 2.03-1.86 (m, 5H); $^{13}C\{^1H\}$ NMR (100 MHz, DMSO-d_6, 298 K) δ (ppm): 169.1, 146.3, 146.3, 130.4, 123.5, 65.4, 50.8, 33.8, 29.3, 28.2, 14.1; HRMS (ESI) m/z: $[M+H]^+$ Calcd. for $C_{13}H_{19}N_2O_4S$ 299.1060; Found 299.1058.

Procedure B (for Gly-Gly and Ala-Gly-Gly-Gly): Step 1. **Boc-aa$_n$-OH** (1.0 equiv.) and 2-(4-nitrophenyl)ethanol (1.3 equiv.) were suspended in dry ACN. Dry pyridine was added giving a solution. The solution was stirred at 0ºC under nitrogen atmosphere. *N*,*N'*-dicyclohexylcarbodiimide (DCC) (1.3 equiv.) and HOBt (1.3 equiv.) were added and the reaction was stirred at r.t. overnight. After that, the reaction was quenched with 1 M aq. citric acid solution at r.t. for 30 min. The crude was diluted with EtOAc and filtered. The precipitate was washed with EtOAc two times. The organic layer was washed with aq. sat. $NaHCO_3$ solution and water. The organic layer was dried (Na_2SO_4), filtered and concentrated. The crude was purified by silica gel column chromatography affording the boc- and npe-protected peptide. Step 2. **Boc-aa$_n$-Onpe** (1.0 equiv.) was dissolved in 4 M HCl in 1,4-dioxane at 0ºC. After the reaction was stirred at r.t. for 2 h, the mixture was concentrated obtaining a white solid. The white solid was triturated with Et_2O, filtered and washed with additional Et_2O. The npe-protected peptide **H-aa$_n$-Onpe** chloride salt was isolated as a white solid.

H-Gly-Gly-Onpe•HCl: Yield: 67% over two steps; IR (ATR) $\tilde{\nu}$ (cm^{-1}): 1741 (s), 1677 (m), 1660 (s), 1570 (w), 1512 (s), 1479 (w), 1229 (w), 1207 (s), 1199 (s), 1109 (m); ^1H NMR (400 MHz, DMSO-d_6, 298 K) δ (ppm): 8.91 (t, J = 5.7 Hz, 1H), 8.21 (br s, 3H), 8.19-8.17 (m, 2H), 7.58-7.56 (m, 2H), 4.34 (t, J = 6.4 Hz, 2H), 3.91 (d, J = 5.7 Hz, 2H), 3.59 (s, 2H), 3.06 (t, J = 6.4 Hz, 2H); $^{13}C\{^1H\}$ NMR (100 MHz, DMSO-d_6, 298 K) δ (ppm): 169.4, 166.6, 146.5, 146.3, 130.3, 123.5, 64.3, 40.6, 39.7 (the signal overlaps with that of the solvent), 34.0; HRMS (ESI) m/z: $[M+H]^+$ Calcd. for $C_{12}H_{16}N_3O_5$ 282.1084; Found 282.1086.

H-Ala-Gly-Gly-Gly-Onpe•HCl: Yield: 60% over two steps; IR (ATR) $\tilde{\nu}$ (cm^{-1}): 3222 (w), 2931 (w), 1743 (w), 1654 (s), 1514 (s), 1188 (s), 1117 (m), 871 (m), 856 (m), 697 (m); ^1H NMR (400 MHz, DMSO-d_6, 298 K) δ (ppm): 8.80 (t, J = 5.5 Hz, 1H), 8.38-8.33 (m, 2H), 8.25 (br s, 3H), 8.18-8.16 (m, 2H), 7.58-7.55 (m, 2H), 4.31 (t, J = 6.4 Hz, 2H), 3.91-3.87 (m, 1H), 3.84-3.79 (m, 4H), 3.76-3.72 (m, 2H), 3.04 (t, J = 6.4 Hz, 2H), 1.36 (d, J = 6.9 Hz, 3H); $^{13}C\{^1H\}$ NMR (100 MHz, DMSO-d_6, 298 K) δ (ppm): 169.9, 169.7, 169.3, 168.6, 146.5, 146.3, 130.3, 123.5, 64.2, 48.2, 42.0, 41.7, 40.6, 34.0, 17.1; HRMS (ESI) m/z $[M+H]^+$: Calcd. for $C_{17}H_{24}N_5O_7$ 410.1670; Found: 410.1671.

2.3 Nucleobase-modified N^6-carbamoyl adenosine phosphoramidites

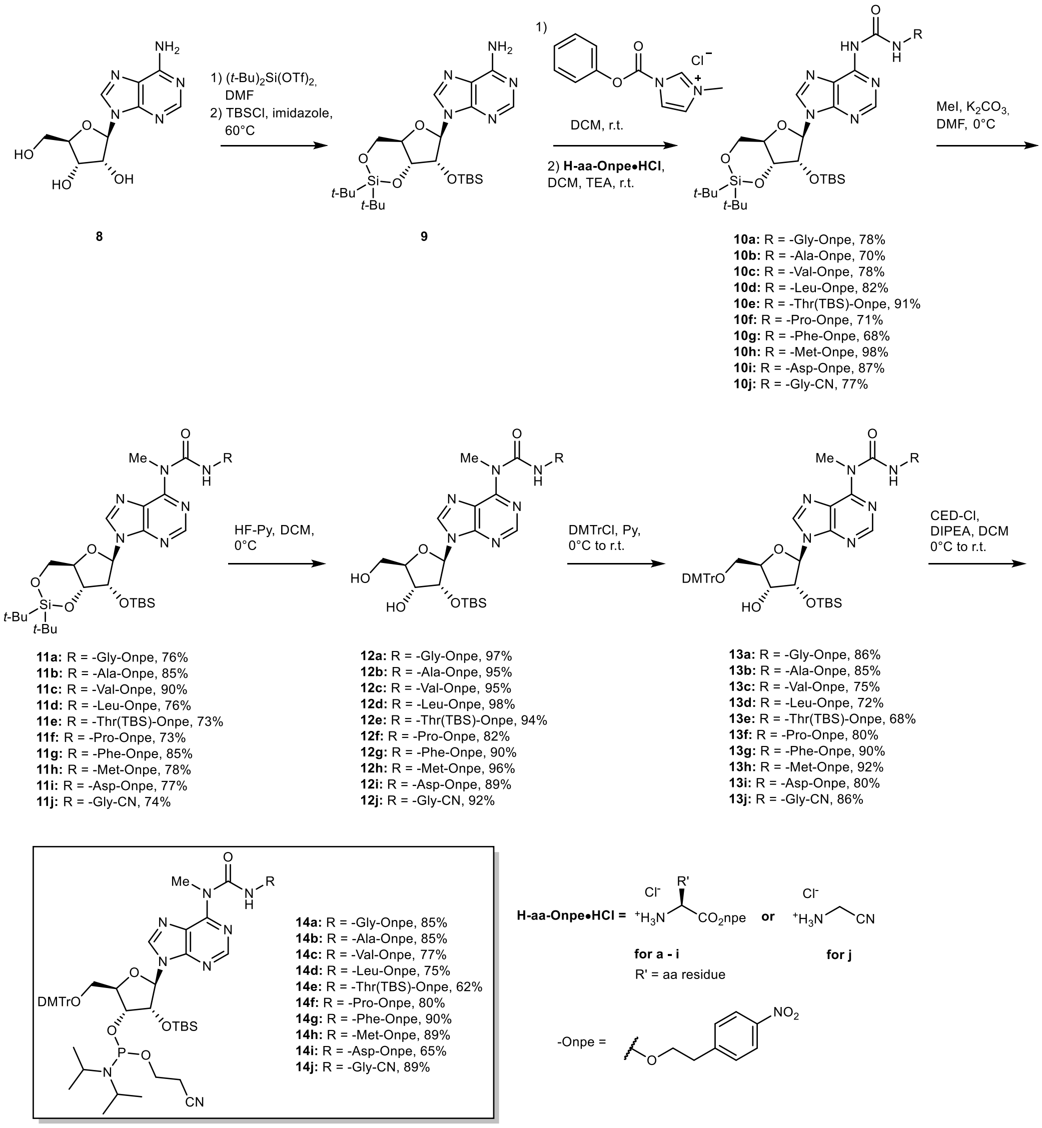

Scheme S3. Synthesis of nucleobase-modified N^6-carbamoyl adenosine phosphoramidites.

General procedure for the synthesis of 10a-j:

The compounds **10a**, **10c**, **10e**, **10g** and **10i** were previously reported in the literature.[4]

To a solution of silyl-protected adenosine **9**[5] (1.0 equiv.) in DCM was added 1-*N*-methyl-3-phenoxycarbonyl-imidazolium chloride (2.0 equiv.). The resulting suspension was stirred at r.t. for 16 h and then **H-aa-Onpe•HCl** (2.0 equiv.) together with NEt_3 (2.0 equiv.) was added. After stirring for 16 h, the reaction mixture was quenched by the addition of aq. sat. $NaHCO_3$ and the crude was extracted three times with DCM. The combined organic layers were dried ($MgSO_4$), filtered and concentrated *in vacuo*. Purification by silica gel column chromatography furnished the amino acid-modified adenosine derivative **10a**-**j** as a white foam.

10b: Yield: 70%; R_f = 0.70 (4:3 *i*-Hexane/EtOAc); IR (ATR) $\tilde{\nu}$ (cm^{-1}): 3239 (w), 2929 (w), 2855 (w), 1744 (m), 1698 (s), 1610 (m), 1586 (m), 1519 (s), 1463 (m), 1344 (s), 1250 (s), 1138 (s), 1054 (s), 998 (m), 894 (s), 825 (s), 782 (s), 749 (s); ^{1}H NMR (400 MHz, $CDCl_3$, 298 K) δ (ppm): 10.02 (d, J = 7.3 Hz, 1H), 8.82 (s, 1H), 8.51 (s, 1H), 8.27 (s, 1H), 8.07 (d, J = 8.7 Hz, 2H), 7.38 (d, J = 8.7 Hz, 2H), 6.00 (s, 1H), 4.68-4.59 (m, 1H), 4.58 (d, J = 4.6 Hz, 1H), 4.55-4.37 (m, 4H), 4.27-4.22 (m, 1H), 4.14-4.05 (m, 1H), 3.09 (t, J = 6.5 Hz, 2H), 1.49 (d, J = 7.2 Hz, 3H), 1.08 (s, 9H), 1.05 (s, 9H), 0.94 (s, 9H), 0.17 (s, 3H), 0.15 (s, 3H); ^{13}C{^{1}H} NMR (100 MHz, $CDCl_3$, 298 K) δ (ppm): 173.0, 153.6, 151.2, 150.3, 149.8, 146.9, 145.5, 141.8, 130.0, 129.9, 123.8, 121.1, 92.5, 75.9, 75.8, 74.9, 67.9, 64.7, 49.2, 35.0, 27.6, 27.1, 26.0, 22.8, 20.5, 18.5, 18.4, -4.2, -4.9; HRMS (ESI) *m*/*z*: $[M+H]^+$ Calcd. for $C_{36}H_{56}N_7O_9Si_2$ 786.3673; Found 786.3682.

10d: Yield: 82%; R_f = 0.43 (4:3 *i*-Hexane/EtOAc); IR (ATR) $\tilde{\nu}$ (cm^{-1}): 3237 (w), 2168 (w), 1666 (s), 1572 (w), 1511 (s), 1429 (w), 1335 (m), 1271 (s), 1227 (m), 1178 (w), 1151 (w), 1119 (m), 1090 (s), 1019 (w), 908 (m), 843 (s), 781 (s); ^{1}H NMR (400 MHz, CDCl$_3$, 298 K) δ (ppm): 9.87 (d, *J* = 7.7 Hz, 1H), 8.50-8.49 (m, 2H), 8.18 (s, 1H), 8.06 (d, *J* = 8.6 Hz, 2H), 7.38 (d, *J* = 8.6 Hz, 2H), 5.98 (s, 1H), 4.64-4.56 (m, 2H), 4.54-4.38 (m, 4H), 4.24 (td, *J* = 10.0, 5.1 Hz, 1H), 4.07 (dd, *J* = 10.0, 9.1 Hz, 1H), 3.09 (t, *J* = 6.5 Hz, 2H), 1.71-1.65 (m, 3H), 1.08 (s, 9H), 1.05 (s, 9H), 0.97-0.91 (m, 15H), 0.18 (s, 3H), 0.16 (s, 3H); ^{13}C{^{1}H} NMR (100 MHz, CDCl$_3$, 298 K) δ (ppm): 173.0, 153.8, 151.2, 150.3, 149.8, 146.9, 145.7, 141.6, 129.9, 123.7, 121.1, 92.6, 75.9, 75.7, 74.9, 67.9, 64.5, 52.1, 41.2, 34.9, 27.6, 27.1, 26.0, 25.2, 23.0, 22.9, 22.0, 20.5, 18.4, -4.2, -4.9; HRMS (ESI) *m/z*: [M+H]$^+$ Calcd. for $C_{39}H_{62}N_7O_9Si_2$ 828.4142; Found 828.4149.

10f: Yield: 71%; R_f = 0.25 (6:4 DCM/EtOAc); IR (ATR) $\tilde{\nu}$ (cm^{-1}): 2933 (w), 1741 (w), 1649 (w), 1519 (m), 1401 (m), 1344 (s), 1166 (m), 1140 (m), 1057 (s), 750 (m); For major rotamer: ^{1}H NMR (400 MHz, CDCl$_3$, 298 K) δ (ppm): 8.65 (s, 1H), 8.10-8.08 (m, 2H), 7.97 (s, 1H), 7.40-7.38 (m, 2H), 5.96 (s, 1H), 4.64 (d, *J* = 4.6 Hz, 1H), 4.61-4.59 (m, 1H), 4.50 (dd, *J* = 9.4, 4.6 Hz, 2H), 4.45-4.42 (m, 2H), 4.24 (ddd, *J* = 9.4, 9.4, 4.6 Hz, 1H), 4.04 (dd, *J* = 9.4, 9.4 Hz, 1H), 3.67-3.64 (m, 2H), 3.10-3.06 (m, 2H), 2.26-2.19 (m, 1H), 2.06-2.00 (m, 3H), 1.07 (s, 9H), 1.04 (s, 9H), 0.93 (s, 9H), 0.17 (s, 3H), 0.15 (s, 3H); ^{13}C{^{1}H} NMR (100 MHz, CDCl$_3$, 298 K) δ (ppm): 172.2, 152.8, 150.8, 147.0, 140.7, 129.9, 123.8, 123.7, 123.2, 92.6, 75.9, 75.6, 74.9, 67.9, 64.3, 59.6, 47.0, 35.0, 29.7, 27.6, 27.1, 26.0, 24.7, 22.9, 20.5, 18.4, -4.1, -4.9 (some carbon signals appeared too broad for an unequivocal assignment); HRMS (ESI) *m/z*: [M+H]$^+$ Calcd. for $C_{38}H_{58}N_7O_9Si_2$ 812.3829; Found 812.3835.

10h: Yield: 98%; R_f = 0.30 (2:1 *i*-Hexane/EtOAc); ^{1}H NMR (400 MHz, CDCl$_3$, 298 K) δ (ppm): 10.01 (d, *J* = 7.8 Hz, 1H), 8.49 (s, 1H), 8.19 (s, 1H), 8.12 (s, 1H), 8.06 (d, *J* = 8.7 Hz, 2H), 7.38 (d, *J* = 8.7 Hz, 2H), 5.97 (s, 1H), 4.74 (td, *J* = 7.8, 5.1 Hz, 1H), 4.62 (d, *J* = 4.6 Hz, 1H), 4.55-4.41 (m, 4H), 4.24 (td, *J* = 10.0, 5.1 Hz, 1H), 4.05 (dd, *J* = 10.5, 9.2 Hz, 1H), 3.09 (t, *J* = 6.5 Hz, 2H), 2.54 (dd, *J* = 8.1, 6.0 Hz, 1H), 2.28-1.98 (m, 1H), 2.10-2.05 (m, 4H), 1.08 (s, 9H), 1.05 (s, 9H), 0.94 (s, 9H), 0.18 (s, 3H), 0.16 (s, 3H); ^{13}C{^{1}H} NMR (100 MHz, CDCl$_3$, 298 K) δ (ppm): 171.9, 153.6, 151.2, 150.1, 149.8, 146.9, 145.5, 141.3, 129.9, 123.8, 121.1, 92.6, 75.9, 75.7, 74.9, 67.9, 64.8, 52.6, 34.9, 31.7, 30.2, 27.6, 27.2, 26.0, 22.9, 20.5, 18.5, 15.6, -4.1, -4.9; HRMS (ESI) *m/z*: [M+H]$^+$ Calcd. for $C_{38}H_{60}N_7O_9SSi_2$ 846.3706; Found 846.3704.

10j: Yield: 77%; R_f = 0.29 (2:1 *i*-Hexane/EtOAc); IR (ATR) $\tilde{\nu}$ (cm^{-1}): 3119 (w), 2930 (m), 2857 (m), 2168 (w), 1706 (s), 1658 (m), 1612 (m), 1525 (m), 1466 (m), 1394 (w), 1353 (m), 1249 (s), 1141 (s), 1058 (s), 997 (m), 892 (m), 825 (s), 783 (s); ^{1}H NMR (600 MHz, CDCl$_3$, 298 K) δ (ppm): 10.10 (t, *J* = 5.7 Hz, 1H), 8.54 (s, 1H), 8.45 (s, 1H), 8.10 (s, 1H), 5.98 (s, 1H), 4.59 (d, *J* = 4.6 Hz, 1H), 4.51 (dd, *J* = 9.3, 4.6 Hz, 1H), 4.45 (dd, *J* = 9.3, 4.6 Hz, 1H), 4.37 (d, *J* = 5.7 Hz, 2H), 4.29-4.21 (m, 1H), 4.06 (dd, *J* = 10.5, 9.3 Hz, 1H), 1.09 (s, 9H), 1.05 (s, 9H), 0.94 (s, 9H), 0.17 (s, 3H), 0.16 (s, 3H); ^{13}C{^{1}H} NMR (150 MHz, CDCl$_3$, 298 K) δ (ppm): 153.7, 151.1, 150.1, 149.8, 141.5, 121.2, 116.4, 92.6, 76.0, 75.8, 75.0, 67.9, 28.4, 27.6, 27.2, 26.0, 22.9, 20.5, 18.5, -4.1, -4.8; HRMS (ESI) *m/z*: [M+H]$^+$ Calcd. for $C_{27}H_{46}N_7O_5Si_2$ 604.3093; Found 604.3094.

General procedure for the synthesis of 11a-j:

The amino acid-modified adenosine derivative **10a**-**j** (1.0 equiv.) was dissolved in DMF and cooled to 0°C. To the solution were added K_2CO_3 (3.0 equiv.) together with MeI (2.0 equiv.) and the reaction was stirred at r.t. for 2 h. The reaction mixture was diluted with H_2O and extracted three times with EtOAc. The combined organic layers were washed with water, dried ($MgSO_4$), filtered and concentrated. The obtained residue was purified by silica gel column chromatography to give **11a**-**j** as a white foam.

11a: Yield: 76%; R_f = 0.22 (2:1 *i*-Hexane/EtOAc); IR (ATR) $\tilde{\nu}$ (cm^{-1}): 3235 (w), 2932 (w), 2858 (w), 1749 (w), 1686 (m), 1568 (w), 1521 (s), 1470 (m), 1347 (s), 1264 (s), 1167 (w), 1135 (m), 1055 (m), 1000 (w), 894 (w), 827 (m), 732 (s); ^{1}H NMR (400 MHz, CDCl$_3$, 298 K) δ (ppm): 10.97 (t, *J* = 5.4 Hz, 1H), 8.50 (s, 1H), 8.11 (d, *J* = 8.7 Hz, 2H), 7.97 (s, 1H), 7.38 (d, *J* = 8.7 Hz, 2H), 6.01 (s, 1H), 4.56 (d, *J* = 4.6 Hz, 1H), 4.51 (dd, *J* = 9.2, 5.2 Hz, 1H), 4.45-4.38 (m, 3H), 4.27-4.22 (m, 1H), 4.16 (dd, *J* = 5.2, 1.7 Hz, 2H), 4.07-3.95 (m, 4H), 3.08 (t, *J* = 6.6 Hz, 2H), 1.07 (s, 9H), 1.04 (s, 9H), 0.94 (s, 9H), 0.18 (s, 3H), 0.15 (s, 3H); ^{13}C{^{1}H} NMR (100 MHz, CDCl$_3$, 298 K) δ (ppm): 170.3, 156.2, 153.2, 151.7, 150.2, 147.0, 145.5, 139.3, 129.9, 123.8, 122.7, 92.4, 76.1, 75.7, 74.8, 68.0, 64.6, 43.0, 35.0, 34.8, 27.6, 27.1, 26.0, 22.9, 20.5, 18.5, -4.1, -4.9; HRMS (ESI) *m/z*: [M+H]$^+$ Calcd. for $C_{36}H_{56}N_7O_9Si_2$ 786.3673; Found 786.3674.

11b: Yield: 85%; R_f = 0.60 (5:3 DCM/EtOAc); IR (ATR) $\tilde{\nu}$ (cm^{-1}): 3190 (w), 2934 (w), 2858 (w), 1744 (m), 1686 (m), 1567 (m), 1519 (s), 1469 (s), 1344 (s), 1250 (m), 1166 (m), 1133 (m), 1057 (s), 1000 (m), 895 (m), 825 (s), 777 (m), 749 (m); ^{1}H NMR (400 MHz, CDCl$_3$, 298 K) δ (ppm): 8.51 (s, 1H), 8.10 (d, *J* = 8.7 Hz, 2H), 8.01 (s, 1H), 7.38 (d, *J* = 8.7 Hz, 2H), 6.01 (s, 1H), 4.63-4.54 (m, 2H), 4.54-4.48 (m, 1H), 4.46-4.39 (m, 3H), 4.30-4.20 (m, 1H), 4.07-4.00 (m, 1H), 3.96 (s, 3H), 3.09 (t, *J* = 6.5 Hz, 2H), 1.46 (d, *J* = 7.2 Hz, 3H), 1.07 (s, 9H), 1.04 (s, 9H), 0.94 (s, 9H), 0.18 (s, 3H), 0.16 (s, 3H); ^{13}C{^{1}H} NMR (100 MHz, CDCl$_3$, 298 K) δ (ppm): 173.4, 155.4, 153.3, 151.6, 150.2, 147.0,

145.6, 139.3, 129.9, 123.8, 122.8, 92.5, 77.4, 76.0, 75.7, 74.8, 68.0, 64.6, 50.0, 35.0, 34.7, 27.6, 27.1, 26.0, 22.9, 20.5, 18.5, 18.4, -4.1, -4.9; HRMS (ESI) *m/z*: $[M+H]^+$ Calcd. for $C_{37}H_{58}N_7O_9Si_2$ 800.3829; Found 800.3836.

11c: Yield: 90%; R_f = 0.34 (1:1 *i*-Hexane/EtOAc); IR (ATR) $\tilde{\nu}$ (cm^{-1}): 3246 (m), 2977 (w), 1732 (m), 1686 (s), 1524 (s), 1469 (w), 1372 (m), 1254 (s), 1177 (m), 1147 (w), 1107 (s), 1050 (s), 1020 (m), 926 (m), 853 (w), 790 (m), 744 (w); ^1H NMR (400 MHz, $CDCl_3$, 298 K) δ (ppm): 11.07 (d, *J* = 7.6 Hz, 1H), 8.48 (s, 1H), 8.09 (d, *J* = 8.7 Hz, 2H), 7.97 (s, 1H), 7.38 (d, *J* = 8.7 Hz, 2H), 5.99 (s, 1H), 4.59 (d, *J* = 4.6 Hz, 1H), 4.54-4.47 (m, 2H), 4.46-4.39 (m, 3H), 4.27-4.22 (m, 1H), 4.03 (dd, *J* = 10.5, 9.2 Hz, 1H), 3.97 (s, 3H), 3.09 (t, *J* = 6.6 Hz, 2H), 2.25-2.18 (m, 1H), 1.07 (s, 9H), 1.04 (s, 9H), 0.99 (d, *J* = 6.8 Hz, 3H), 0.95-0.93 (m, 12H), 0.18 (s, 3H), 0.16 (s, 3H); $^{13}C\{^1H\}$ NMR (100 MHz, $CDCl_3$, 298 K) δ (ppm): 172.4, 156.0, 153.3, 151.6, 150.0, 146.9, 145.7, 139.3, 129.9, 123.8, 122.8, 92.5, 76.0, 75.6, 74.9, 68.0, 64.4, 59.8, 35.0, 34.7, 30.8, 27.6, 27.1, 26.0, 22.9, 20.5, 19.6, 18.5, 18.2, -4.1, -4.8; HRMS (ESI) *m/z*: $[M+H]^+$ Calcd. for $C_{39}H_{62}N_7O_9Si_2$ 828.4142; Found 828.4143.

11d: Yield: 76%; R_f = 0.47 (5:3 *i*-Hexane/EtOAc); IR (ATR) $\tilde{\nu}$ (cm^{-1}): 3230 (w), 2933 (w), 1740 (s), 1690 (s), 1580 (s), 1520 (s), 1469 (s), 1345 (s), 1259 (s), 1134 (s), 1057 (s), 1013 (s), 900 (w), 826 (s), 780 (s), 750 (s); ^1H NMR (400 MHz, $CDCl_3$, 298 K) δ (ppm): 10.92 (d, *J* = 6.8 Hz, 1H), 8.47 (s, 1H), 8.09 (d, *J* = 8.7 Hz, 2H), 7.97 (s, 1H), 7.38 (d, *J* = 8.7 Hz, 2H), 6.00 (s, 1H), 4.59 (d, *J* = 4.6 Hz, 1H), 4.57-4.48 (m, 2H), 4.47-4.39 (m, 3H), 4.25 (td, *J* = 10.1, 5.1 Hz, 1H), 4.03 (dd, *J* = 10.5, 9.2 Hz, 1H), 3.96 (s, 3H), 3.08 (t, *J* = 6.6 Hz, 2H), 1.73-1.61 (m, 3H), 1.07 (s, 9H), 1.05 (s, 9H), 0.95-0.94 (m, 12H), 0.93 (s, 3H), 0.18 (s, 3H), 0.16 (s, 3H); $^{13}C\{^1H\}$ NMR (100 MHz, $CDCl_3$, 298 K) δ (ppm): 173.4, 155.7, 153.3, 151.6, 150.1, 146.9, 145.7, 139.3, 129.9, 123.8, 122.8, 92.5, 76.0, 75.6, 74.9, 68.0, 64.5, 53.0, 41.2, 35.0, 34.7, 27.6, 27.1, 26.0, 25.3, 23.0, 22.9, 22.1, 20.5, 18.5, -4.1, -4.8; HRMS (ESI) *m/z*: $[M+H]^+$ Calcd. for $C_{40}H_{64}N_7O_9Si_2$ 842.4299; Found 842.4296.

11e: Yield: 73%; R_f = 0.34 (4:3 *i*-Hexane/EtOAc); IR (ATR) $\tilde{\nu}$ (cm^{-1}): 3237 (w), 2931 (s), 2857 (s), 1737 (s), 1701 (s), 1610 (s), 1520 (s), 1465 (s), 1345 (s), 1250 (s), 1136 (w), 1057 (s), 998 (w), 894 (w), 840 (s), 777 (s); ^1H NMR (400 MHz, $CDCl_3$, 298 K) δ (ppm): 11.00 (d, *J* = 8.7 Hz, 1H), 8.43 (s, 1H), 8.01-7.96 (m, 3H), 7.32 (d, *J* = 8.7 Hz, 2H), 6.02 (s, 1H), 4.60 (d, *J* = 4.6 Hz, 1H), 4.58 (dd, *J* = 8.7, 1.7 Hz, 1H), 4.53-4.46 (m, 3H), 4.43-4.30 (m, 2H), 4.29-4.22 (m, 1H), 4.04 (t, *J* = 9.5 Hz, 1H), 3.98 (s, 3H), 3.03 (t, *J* = 6.5 Hz, 2H), 1.23 (d, *J* = 6.2 Hz, 3H), 1.08 (s, 9H), 1.05 (s, 9H), 0.95 (s, 9H), 0.88 (s, 9H), 0.19 (s, 3H), 0.16 (s, 3H), 0.05 (s, 3H), -0.05 (s, 3H); $^{13}C\{^1H\}$ NMR (100 MHz, $CDCl_3$, 298 K) δ (ppm): 171.3, 156.4, 153.4, 151.6, 150.2, 146.8, 145.7, 139.4, 129.9, 123.7, 122.8, 92.5, 76.0, 75.7, 74.9, 68.9, 68.0, 64.6, 60.6, 34.9, 27.6, 27.2, 26.0, 25.7, 22.9, 21.3, 20.5, 18.5, 18.0, -4.1, -4.2, -4.9, -5.3; HRMS (ESI) *m/z*: $[M+H]^+$ Calcd. for $C_{44}H_{74}N_7O_{10}Si_3$ 944.4799; Found: 944.4793.

11f: Yield: 73%; R_f = 0.30 (8:2 DCM/EtOAc); IR (ATR) $\tilde{\nu}$ (cm^{-1}): 2933 (w), 1744 (w), 1683 (m), 1583 (m), 1392 (m), 1345 (s), 1166 (m), 1056 (s), 1002 (m), 783 (m); ^1H NMR (400 MHz, $CDCl_3$, 298 K) δ (ppm): 8.46 (s, 1H), 8.17-8.15 (m, 2H), 7.84 (s, 1H), 7.39-7.38 (m, 2H), 5.93 (s, 1H), 4.58-4.57 (m, 1H), 4.54 (br s, 2H), 4.49 (dd, *J* = 9.8, 5.1 Hz, 2H), 4.39-4.35 (m, 1H), 4.22 (ddd, *J* = 9.8, 9.8, 5.1 Hz, 1H), 4.03 (dd, *J* = 9.8, 9.8 Hz, 1H), 3.55 (br s, 3H), 3.06 (br s, 2H), 2.18-2.16 (m, 1H), 1.88-1.86 (m, 3H), 1.08 (s, 9H), 1.04 (s, 9H), 0.92 (s, 9H), 0.15 (s, 3H), 0.14 (s, 3H) (some proton signals of proline appeared too broad for an unequivocal assignment); $^{13}C\{^1H\}$ NMR (100 MHz, $CDCl_3$, 298 K) δ (ppm): 172.1, 156.9, 153.3, 152.5, 150.8, 147.0, 145.6, 139.4, 129.9, 123.9, 92.6, 76.0, 75.6, 74.8, 67.9, 64.6, 60.0, 48.0, 35.0, 34.9, 27.6, 27.1, 26.0, 24.2, 22.9, 20.5, 18.5, -4.2, -4.8 (some carbon signals appeared too broad for an unequivocal assignment); HRMS (ESI) *m/z*: $[M+H]^+$ Calcd. for $C_{39}H_{60}N_7O_9Si_2$ 826.3985; Found 826.3991.

11g: Yield: 85%; R_f = 0.50 (2:1 *i*-Hexane/EtOAc); IR (ATR) $\tilde{\nu}$ (cm^{-1}): 2931 (w), 2857 (w), 1738 (w), 1682 (s), 1568 (s), 1518 (s), 1469 (s), 1344 (s), 1261 (s), 1166 (s), 1134 (s), 1056 (s), 1011 (s), 895 (w), 826 (s), 778 (s); ^1H NMR (400 MHz, CD_2Cl_2, 298 K) δ (ppm): 10.87 (d, *J* = 6.9 Hz, 1H), 8.30 (s, 1H), 8.04 (d, *J* = 8.7 Hz, 2H), 7.99 (s, 1H), 7.34 (d, *J* = 8.7 Hz, 2H), 7.29-7.24 (m, 3H), 7.20-7.12 (m, 2H), 6.02 (s, 1H), 4.77 (q, *J* = 6.5 Hz, 1H), 4.59 (d, *J* = 4.6 Hz, 1H), 4.53-4.42 (m, 2H), 4.38 (td, *J* = 6.5, 3.9 Hz, 2H), 4.25 (td, *J* = 10.0, 5.1 Hz, 1H), 4.06 (dd, *J* = 10.5, 9.2 Hz, 1H), 3.88 (s, 3H), 3.13 (dd, *J* = 6.4, 2.1 Hz, 2H), 3.03 (t, *J* = 6.4 Hz, 2H), 1.09 (s, 9H), 1.06 (s, 9H), 0.96 (s, 9H), 0.19 (s, 3H), 0.17 (s, 3H); $^{13}C\{^1H\}$ NMR (100 MHz, CD_2Cl_2, 298 K) δ (ppm): 172.4, 155.9, 153.5, 152.1, 150.3, 147.3, 146.5, 139.9, 137.2, 130.4, 129.9, 129.0, 127.6, 124.0, 123.1, 92.8, 76.5, 76.1, 75.3, 68.3, 65.0, 56.3, 38.3, 35.3, 34.9, 27.8, 27.4, 26.2, 23.1, 20.8, 18.8, -4.0, -4.7; HRMS (ESI) *m/z*: $[M+H]^+$ Calcd. for $C_{43}H_{62}N_7O_9Si_2$ 876.4142; Found 876.4148.

11h: Yield: 78%; R_f = 0.30 (2.5:1 *i*-Hexane/EtOAc); ^1H NMR (400 MHz, $CDCl_3$, 298 K) δ (ppm): 11.08 (d, *J* = 7.2 Hz, 1H), 8.49 (s, 1H), 8.09 (d, *J* = 8.6 Hz, 2H), 7.97 (s, 1H), 7.38 (d, *J* = 8.6 Hz, 2H), 6.00 (s, 1H), 4.70 (td, *J* = 7.2, 5.1 Hz, 1H), 4.59 (d, *J* = 4.6 Hz, 1H), 4.52 (dd, *J* = 9.2, 5.1 Hz, 1H), 4.46-4.41 (m, 3H), 4.25 (td, *J* = 10.1, 5.1 Hz, 1H), 4.03 (dd, *J* = 10.5, 9.2 Hz, 1H), 3.96 (s, 3H), 3.09 (t, *J* = 6.5 Hz, 2H), 2.52 (td, *J* = 7.3, 1.9 Hz, 2H), 2.24-1.99 (m, 2H), 2.07 (s, 3H), 1.07 (s, 8H), 1.04 (s, 9H), 0.95 (s, 9H), 0.18 (s, 3H), 0.16 (s, 3H); $^{13}C\{^1H\}$ NMR (100 MHz, $CDCl_3$, 298 K) δ (ppm): 172.3, 155.7, 153.2, 151.7, 150.1, 147.0, 145.6, 139.4, 129.9, 123.8, 122.8, 92.5, 76.0, 75.7, 74.9, 68.0, 64.8, 53.5, 35.0, 34.8, 31.7, 30.3, 27.6, 27.1, 26.0, 22.9, 20.5, 18.5, 15.6, -4.1, -4.8; HRMS (ESI) *m/z*: $[M+H]^+$ Calcd. for $C_{39}H_{62}N_7O_9SSi_2$ 860.3863; Found 860.3858.

11i: Yield: 77%; R_f = 0.40 (8:2 DCM/EtOAc); IR (ATR) $\tilde{\nu}$ (cm^{-1}): 2933 (w), 1737 (m), 1683 (m), 1569 (m), 1518 (s), 1344 (s), 1166 (m), 1057 (m), 1000 (m), 780 (m); ^{1}H NMR (400 MHz, $CDCl_3$, 298 K) δ (ppm): 11.32 (d, J = 7.5 Hz, 1H), 8.40 (s, 1H), 8.09-8.06 (m, 2H), 8.02-7.99 (m, 2H), 7.98 (s, 1H), 7.34-7.32 (m, 4H), 6.01 (s, 1H), 4.87 (dt, J = 7.5, 4.5 Hz, 1H), 4.61 (d, J = 4.5 Hz, 1H), 4.52 (dd, J = 9.2, 4.5 Hz, 1H), 4.46-4.40 (m, 3H), 4.40-4.23 (m, 3H), 4.04 (dd, J = 9.2, 9.2 Hz, 1H), 3.94 (s, 3H), 3.06-2.99 (m, 4H), 2.97-2.95 (m, 2H), 1.07 (s, 9H), 1.05 (s, 9H), 0.95 (s, 9H), 0.19 (s, 3H), 0.16 (s, 3H); ^{13}C{^{1}H} NMR (100 MHz, $CDCl_3$, 298 K) δ (ppm): 171.0, 170.9, 155.6, 152.9, 151.7, 149.9, 146.9, 146.8, 145.6, 145.5, 139.5, 129.8 (×2), 123.8, 123.7, 122.6, 92.5, 76.0, 75.6, 74.9, 68.0, 65.0, 64.4, 50.5, 36.6, 34.9, 34.8, 34.7, 27.6, 27.1, 26.0, 22.9, 20.5, 18.5, -4.1, -4.9; HRMS (ESI) m/z: [M+H]$^+$ Calcd. for $C_{46}H_{65}O_{13}N_8Si_2$ 993.4203; Found 993.4215.

11j: Yield: 74%; R_f = 0.34 (4:1 *i*-Hexane/EtOAc); IR (ATR) $\tilde{\nu}$ (cm^{-1}): 3121 (w), 2933 (m), 2896 (w), 2857 (m), 2168 (w), 1692 (s), 1570 (s), 1525 (s), 1469 (s), 1422 (w), 1360 (m), 1328 (m), 1308 (w), 1299 (w), 1278 (m), 1249 (m), 1218 (w), 1198 (w), 1165 (s), 1141 (s), 1111 (m), 1062 (s), 1024 (s), 1001 (s), 968 (w), 889 (m), 825 (s), 784 (s); ^{1}H NMR (600 MHz, $CDCl_3$, 298 K) δ (ppm): 11.14 (t, J = 5.6 Hz, 1H), 8.53 (s, 1H), 7.99 (s, 1H), 6.01 (s, 1H), 4.56 (d, J = 4.6 Hz, 1H), 4.52 (dd, J = 9.4, 4.6 Hz, 1H), 4.39 (dd, J = 9.4, 4.6 Hz, 1H), 4.34 (dd, J = 5.2, 2.0 Hz, 2H), 4.26 (td, J = 9.4, 5.2 Hz, 1H), 4.04-4.02 (m, 1H), 4.02 (s, 3H), 1.07 (s, 9H), 1.05 (s, 9H), 0.95 (s, 9H), 0.18 (s, 3H), 0.16 (s, 3H); ^{13}C{^{1}H} NMR (150 MHz, $CDCl_3$, 298 K) δ (ppm): 156.0, 152.9, 151.9, 150.1, 139.6, 122.8, 116.7, 92.5, 76.1, 75.7, 74.9, 67.9, 35.0, 29.2, 27.6, 27.2, 26.0, 22.9, 20.5, 18.5, -4.1, -4.8; HRMS (ESI) m/z: [M+H]$^+$ Calcd. for $C_{28}H_{48}N_7O_5Si_2$ 618.3250; Found 618.3256.

General procedure for the synthesis of 12a-j:

A solution of the modified adenosine derivative **11a**-**j** (1.0 equiv.) in DCM/pyridine (9:1 v/v) inside a plastic reaction vessel was cooled to 0°C. Subsequently, a solution of 70% HF-pyridine (5.0 equiv.) was slowly added and the reaction mixture was stirred at 0°C for 2 h. The reaction mixture was diluted with aq. sat. $NaHCO_3$ solution and extracted three times with DCM. The combined organic layers were washed with water, dried ($MgSO_4$), filtered and concentrated under reduced pressure. The crude product was purified by silica gel column chromatography to isolate the 3',5'-deprotected adenosine derivative **12a**-**j** as a white foam.

12a: Yield: 97%; R_f = 0.37 (100:5 DCM/MeOH); IR (ATR) $\tilde{\nu}$ (cm^{-1}): 2932 (w), 2857 (w), 1738 (w), 1688 (m), 1606 (w), 1581 (m), 1571 (m), 1518 (s), 1471 (m), 1445 (w), 1345 (s), 1253 (m), 1219 (m), 1135 (m), 1083 (m), 1031 (m), 858 (w), 838 (s), 780 (s), 750 (m); ^{1}H NMR (400 MHz, $CDCl_3$, 298 K) δ (ppm): 10.89 (t, J = 5.3 Hz, 1H), 8.52 (s, 1H), 8.14 (d, J = 8.6 Hz, 2H), 7.96 (s, 1H), 7.39 (d, J = 8.6 Hz, 2H), 5.90 (d, J = 11.6 Hz, 1H), 5.81 (d, J = 7.3 Hz, 1H), 5.12 (dd, J = 7.3, 4.9 Hz, 1H), 4.43 (td, J = 6.7, 1.7 Hz, 2H), 4.39-4.33 (m, 2H), 4.24-4.10 (m, 2H), 4.03-3.91 (m, 4H), 3.82-3.70 (m, 1H), 3.09 (t, J = 6.7 Hz, 2H), 2.81 (s, 1H), 0.80 (s, 9H), -0.18 (s, 3H), -0.39 (s, 3H); ^{13}C{^{1}H} NMR (100 MHz, $CDCl_3$, 298 K) δ (ppm): 170.2, 155.9, 153.8, 151.2, 149.7, 147.0, 145.4, 141.6, 129.9, 123.9, 123.7, 91.4, 87.7, 74.2, 72.8, 64.8, 63.5, 43.0, 35.0, 25.6, 17.9, -5.2, -5.3; HRMS (ESI) m/z: [M+H]$^+$ Calcd. for $C_{28}H_{40}N_7O_9Si$ 646.2651; Found 646.2645.

12b: Yield: 95%; R_f = 0.40 (100:5 DCM/IPA); IR (ATR) $\tilde{\nu}$ (cm^{-1}): 3191 (w), 2927 (w), 2856 (w), 1739 (m), 1681 (s), 1610 (m), 1568 (s), 1519 (s), 1469 (m), 1344 (s), 1261 (m), 1211 (w), 1143 (w), 1018 (m), 998 (m), 836 (s), 779 (s); ^{1}H NMR (400 MHz, $CDCl_3$, 298 K) δ (ppm): 10.73 (br s, 1H), 8.58 (s, 1H), 8.15 (d, J = 8.7 Hz, 2H), 8.11 (s, 1H), 7.41 (d, J = 8.7 Hz, 2H), 5.84 (d, J = 6.9 Hz, 1H), 5.13-5.06 (m, 1H), 4.66-4.53 (m, 1H), 4.49-4.40 (m, 2H), 4.39-4.35 (m, 2H), 4.00-3.93 (m, 4H), 3.78 (d, J = 12.9 Hz, 1H), 3.10 (t, J = 6.6 Hz, 2H), 1.47 (d, J = 7.2 Hz, 3H), 0.82 (s, 9H), -0.14 (s, 3H), -0.34 (s, 3H); ^{13}C{^{1}H} NMR (100 MHz, $CDCl_3$, 298 K) δ (ppm): 173.4, 155.1, 153.9, 151.2, 149.7, 147.1, 145.6, 141.5, 130.0, 123.9, 123.8, 91.5, 87.7, 74.2, 72.9, 64.8, 63.5, 50.1, 35.0, 34.9, 25.6, 18.4, 18.0, -5.2, -5.3; HRMS (ESI) m/z: [M+H]$^+$ Calcd. for $C_{29}H_{42}N_7O_9Si$ 660.2808; Found: 660.2807.

12c: Yield: 95%; R_f = 0.16 (100:3 DCM/MeOH); IR (ATR) $\tilde{\nu}$ (cm^{-1}): 3244 (w), 2952 (w), 2929 (w), 2359 (w), 1736 (w), 1681 (m), 1571 (m), 1518 (s), 1469 (m), 1422 (w), 1345 (s), 1255 (m), 1187 (m), 1145 (m), 1089 (m), 1046 (w), 1016 (m), 907 (m), 857 (m), 837 (s), 780 (s), 746 (m); ^{1}H NMR (400 MHz, $CDCl_3$, 298 K) δ (ppm): 11.02 (d, J = 7.4 Hz, 1H), 8.54 (s, 1H), 8.15 (d, J = 8.6 Hz, 2H), 7.95 (s, 1H), 7.41 (d, J = 8.6 Hz, 2H), 5.93 (dd, J = 11.9, 1.5 Hz, 1H), 5.81 (d, J = 7.4 Hz, 1H), 5.15 (dd, J = 7.4, 4.8 Hz, 1H), 4.51-4.34 (m, 5H), 4.02-3.91 (m, 4H), 3.81-3.71 (m, 1H), 3.10 (t, J = 6.6 Hz, 2H), 2.81 (s, 1H), 2.29-2.15 (m, 1H), 0.98 (d, J = 6.8 Hz, 3H), 0.94 (d, J = 6.9 Hz, 3H), 0.81 (s, 9H), -0.16 (s, 3H), -0.37 (s, 3H); ^{13}C{^{1}H} NMR (100 MHz, $CDCl_3$, 298 K) δ (ppm): 172.4, 155.7, 154.0, 151.2, 149.6, 147.0, 145.6, 141.5, 129.9, 123.9, 123.7, 91.5, 87.7, 74.1, 72.8, 64.6, 63.5, 59.8, 35.0, 34.9, 30.8, 25.6, 19.6, 18.1, 17.9, -5.2, -5.3; HRMS (ESI) m/z: [M+H]$^+$ Calcd. for $C_{31}H_{46}N_7O_9Si$ 688.3121; Found 688.3120.

12d: Yield: 98%; R_f = 0.52 (9:1 DCM/MeOH); IR (ATR) $\tilde{\nu}$ (cm^{-1}): 3244 (w), 2952 (w), 2929 (w), 2856 (w), 1736 (w), 1695 (s), 1610 (s), 1588 (s), 1520 (s), 1469 (s), 1345 (s), 1313 (w), 1250 (s), 1129 (w), 1093 (s), 835 (s), 760 (s); ^{1}H NMR (400 MHz, acetone-d_6, 298 K) δ (ppm): 10.86 (d, J = 7.1 Hz, 1H), 8.63 (s, 1H), 8.58 (s, 1H), 8.13 (d, J = 8.7 Hz, 2H), 7.59 (d, J = 8.7 Hz, 2H), 6.13 (d, J = 5.8 Hz, 1H), 5.04 (dd, J = 8.3, 3.7 Hz, 1H), 4.98 (t, J = 4.7 Hz, 1H), 4.49-4.41 (m, 3H), 4.40-4.37 (m, 1H), 4.21 (dd, J = 2.6 Hz, 1H), 3.98 (d, J = 4.0 Hz, 1H), 3.92 (s, 3H), 3.90-

3.87 (m, 1H), 3.80- 3.75 (m, 1H), 3.15 (t, *J* = 6.4 Hz, 2H), 1.76-1.53 (m, 3H), 0.91 (dd, *J* = 6.4, 3.3 Hz, 6H), 0.81 (s, 9H), -0.05 (s, 3H), -0.18 (s, 3H); $^{13}C\{^1H\}$ NMR (100 MHz, acetone-d_6, 298 K) *δ* (ppm): 173.4, 155.9, 154.0, 152.7, 150.5, 147.6, 147.5, 142.5, 131.1, 124.1, 123.6, 90.5, 87.5, 76.6, 72.4, 65.1, 62.8, 53.7, 41.7, 35.3, 34.8, 26.0, 25.8, 23.1, 22.1, 18.6, -4.9, -5.1; HRMS (ESI) *m/z*: $[M+H]^+$ Calcd. for $C_{32}H_{48}N_7O_9Si$ 702.3277; Found: 702.3279.

12e: Yield: 94%; R_f = 0.37 (9:1 DCM/MeOH); IR (ATR) $\tilde{\nu}$ (cm^{-1}): 3244 (w), 2952 (w), 2929 (w), 2856 (w), 1736 (w), 1695 (s), 1610 (s), 1588 (s), 1520 (s), 1469 (s), 1345 (s), 1313 (w), 1250 (s), 1129 (w), 1093 (s), 835 (s), 760 (s); ^1H NMR (400 MHz, $CDCl_3$, 298 K) *δ* (ppm): 10.90 (d, *J* = 8.6 Hz, 1H), 8.48 (s, 1H), 8.10 (d, *J* = 8.6 Hz, 2H), 7.96 (s, 1H), 7.37 (d, *J* = 8.6 Hz, 2H), 5.82 (d, *J* = 7.3 Hz, 1H), 5.14 (dd, *J* = 7.3, 4.8 Hz, 1H), 4.56 (dd, *J* = 8.6, 1.8 Hz, 1H), 4.50-4.41 (m, 2H), 4.39-4.35 (m, 2H), 4.29-4.23 (m, 1H), 3.99 (s, 3H), 3.96 (dd, *J* = 13.0, 1.8 Hz, 1H), 3.76 (dd, *J* = 13.0, 1.8 Hz, 1H), 3.06 (t, *J* = 6.7 Hz, 2H), 1.22 (d, *J* = 6.2 Hz, 3H), 0.87 (s, 9H), 0.81 (s, 9H), 0.03 (s, 3H), -0.06 (s, 3H), -0.16 (s, 3H), -0.37 (s, 3H); $^{13}C\{^1H\}$ NMR (100 MHz, $CDCl_3$, 298 K) *δ* (ppm): 171.2, 156.1, 154.1, 151.2, 149.8, 147.0, 145.6, 141.5, 130.0, 123.9, 91.5, 87.7, 74.2, 72.9, 68.8, 64.9, 63.5, 60.7, 35.1, 35.0, 25.7, 25.6, 21.3, 18.0, 17.9, -4.1, -5.2, -5.3, -5.3; HRMS (ESI) *m/z*: $[M+H]^+$ Calcd. for $C_{36}H_{58}N_7O_{10}Si_2$ 804.3778; Found: 804.3768.

12f: Yield: 82%; R_f = 0.15 (98:2 DCM/IPA); IR (ATR) $\tilde{\nu}$ (cm^{-1}): 2929 (w), 1743 (w), 1679 (m), 1585 (s), 1519 (m), 1391 (m), 1344 (s), 1090 (m), 1046 (m), 780 (m); ^1H NMR (400 MHz, $CDCl_3$, 298 K) *δ* (ppm): 8.47 (s, 1H), 8.17-8.14 (m, 2H), 7.81 (s, 1H), 7.39 (br s, 2H), 6.35 (d, *J* = 12.1 Hz, 1H), 5.76 (d, *J* = 7.4 Hz, 1H), 5.15 (dd, *J* = 7.4, 4.8 Hz, 1H), 4.61-4.46 (m, 2H), 4.38 (s, 1H), 4.35 (d, *J* = 4.8 Hz, 1H), 3.96 (d, *J* = 12.1 Hz, 1H), 3.75 (dd, *J* = 12.1, 12.1 Hz, 1H), 3.58 (br s, 3H), 3.06 (br s, 2H), 2.80 (s, 1H), 2.18-2.13 (m, 1H), 1.92-1.88 (m, 3H), 0.79 (s, 9H), -0.19 (s, 3H), -0.41 (s, 3H) (some proton signals of proline appeared too broad for an unequivocal assignment); $^{13}C\{^1H\}$ NMR (100 MHz, $CDCl_3$, 298 K) *δ* (ppm): 171.9, 156.3, 153.7, 152.0, 150.1, 147.0, 141.3, 129.9, 123.9, 91.3, 87.8, 74.1, 73.0, 64.7, 63.5, 59.9, 47.9, 35.0, 34.9, 25.6, 24.2, 17.9, -5.2, -5.4 (some carbon signals appeared too broad for an unequivocal assignment); HRMS (ESI) *m/z*: $[M+H]^+$ Calcd. for $C_{31}H_{44}N_7O_9Si$ 686.2964; Found 686.2963.

12g: Yield: 90%; R_f = 0.50 (98:2 *i*-Hexane/EtOAc); IR (ATR) $\tilde{\nu}$ (cm^{-1}): 3391 (w), 3194 (w), 2951 (w), 2855 (w), 1738 (s), 1681 (s), 1568 (s), 1516 (s), 1469 (s), 1344 (s), 1261 (s), 1171 (s), 1128 (s), 1091 (s), 1016 (s), 836 (s), 779 (s); ^1H NMR (400 MHz, CD_2Cl_2, 298 K) *δ* (ppm): 10.81 (d, *J* = 6.7 Hz, 1H), 8.31 (s, 1H), 8.11 (d, *J* = 8.7 Hz, 2H), 7.99 (s, 1H), 7.39 (d, *J* = 8.7 Hz, 2H), 7.33-7.20 (m, 3H), 7.17-7.13 (m, 2H), 5.83 (d, *J* = 7.3 Hz, 1H), 5.67 (dd, *J* = 11.9, 2.0 Hz, 1H), 5.10 (dd, *J* = 7.3, 4.7 Hz, 1H), 4.78 (td, *J* = 6.8, 5.7 Hz, 1H), 4.46-4.36 (m, 2H), 4.36-4.32 (m, 2H), 3.93-3.89 (m, 4H), 3.79-3.68 (m, 1H), 3.13 (dd, *J* = 6.3, 3.6 Hz, 2H), 3.09-2.99 (m, 2H), 2.81 (s, 1H), 0.80 (s, 9H), -0.18 (s, 3H), -0.38 (s, 3H); $^{13}C\{^1H\}$ NMR (100 MHz, CD_2Cl_2, 298 K) *δ* (ppm): 172.3, 155.6, 154.1, 151.7, 150.3, 149.9, 147.4, 146.4, 142.1, 137.1, 130.4, 129.9, 129.1, 127.7, 124.1, 91.7, 88.2, 74.7, 73.3, 65.2, 63.7, 56.3, 38.3, 35.3, 35.0, 25.8, 18.2, -5.1, -5.2; HRMS (ESI) *m/z*: $[M+H]^+$ Calcd. for $C_{35}H_{46}N_7O_9Si$ 736.3121; Found 736.3118.

12h: Yield: 96%; R_f = 0.40 (100:5 DCM/MeOH); ^1H NMR (400 MHz, acetone-d_6, 298 K) *δ* (ppm): 10.93 (d, *J* = 7.1 Hz, 1H), 8.63 (s, 1H), 8.58 (s, 1H), 8.12 (d, *J* = 8.7 Hz, 2H), 7.59 (d, *J* = 8.7 Hz, 2H), 6.12 (d, *J* = 5.8 Hz, 1H), 5.02 (dd, *J* = 8.3, 3.7 Hz, 1H), 4.97 (dd, *J* = 5.9, 4.7 Hz, 1H), 4.60 (td, *J* = 7.5, 5.3 Hz, 1H), 4.46 (td, *J* = 6.5, 1.9 Hz, 2H), 4.38 (td, *J* = 4.4, 2.9 Hz, 1H), 4.22-4.18 (m, 1H), 3.96 (d, *J* = 4.0 Hz, 1H), 3.92-3.87 (m, 4H), 3.82-3.74 (m, 1H), 3.16 (t, *J* = 6.3 Hz, 2H), 2.52 (t, *J* = 7.9 Hz, 2H), 2.15-1.97 (m, 5H), 0.80 (s, 9H), -0.05 (s, 3H), -0.18 (s, 3H); $^{13}C\{^1H\}$ NMR (100 MHz, acetone-d_6, 298 K) *δ* (ppm): 172.6, 155.9, 154.0, 152.8, 150.5, 147.7, 147.4, 142.5, 131.1, 124.2, 123.6, 90.4, 87.5, 76.6, 72.4, 65.4, 62.8, 54.2, 35.3, 34.9, 32.2, 30.6, 26.0, 18.6, 15.1, -4.9, -5.1; HRMS (ESI) *m/z*: $[M+H]^+$ Calcd. for $C_{31}H_{46}N_7O_9SSi$ 720.2841; Found 720.2833.

12i: Yield: 89%; R_f = 0.15 (97:3 DCM/IPA); IR (ATR) $\tilde{\nu}$ (cm^{-1}): 2930 (w), 1735 (m), 1682 (m), 1570 (m), 1516 (s), 1468 (m), 1261 (m), 1018 (m), 837 (m), 781 (m); ^1H NMR (400 MHz, $CDCl_3$, 298 K) *δ* (ppm): 11.31 (d, *J* = 7.5 Hz, 1H), 8.48 (s, 1H), 8.13-8.10 (m, 4H), 7.96 (s, 1H), 7.38-7.33 (m, 4H), 5.87 (d, *J* = 12.5 Hz, 1H), 5.81 (d, *J* = 7.3 Hz, 1H), 5.13 (dd, *J* = 7.3, 4.8 Hz, 1H), 4.87 (dt, *J* = 7.3, 4.8 Hz, 1H), 4.49-4.25 (m, 6H), 3.97 (s, 3H), 3.97-3.94 (m, 1H), 3.77 (dd, *J* = 12.5, 12.5 Hz, 1H), 3.06 (t, *J* = 6.7 Hz, 2H), 3.01 (t, *J* = 6.7 Hz, 2H), 2.99-2.94 (m, 2H), 2.80 (s, 1H), 0.81 (s, 9H), -0.16 (s, 3H), -0.38 (s, 3H); $^{13}C\{^1H\}$ NMR (100 MHz, $CDCl_3$, 298 K) *δ* (ppm): 170.9, 170.8, 155.4, 153.7, 151.3, 150.0, 149.6, 147.0, 145.5, 145.3, 141.7, 129.9, 129.8, 123.9, 123.8, 123.7, 91.5, 87.7, 74.2, 72.8, 65.2, 64.5, 63.5, 50.6, 36.6, 34.9, 34.8 (×2), 25.6, 17.9, -5.2, -5.3; HRMS (ESI) *m/z*: $[M+H]^+$ Calcd. for $C_{38}H_{49}O_{13}N_8Si$ 853.3182; Found 853.3187.

12j: Yield: 92%; R_f = 0.44 (10:1 DCM/MeOH); IR (ATR) $\tilde{\nu}$ (cm^{-1}): 3347 (w), 2929 (m), 2857 (m), 1731 (w), 1681 (s), 1570 (s), 1515 (s), 1462 (s), 1422 (m), 1360 (w), 1329 (w), 1262 (s), 1217 (m), 1126 (s), 1035 (s), 994 (m), 901 (m), 866 (m), 835 (s), 779 (s); ^1H NMR (600 MHz, $CDCl_3$, 298 K) *δ* (ppm): 11.02 (t, *J* = 5.7 Hz, 1H), 8.55 (s, 1H), 7.98 (s, 1H), 5.82 (d, *J* = 7.4 Hz, 1H), 5.77 (d, *J* = 11.4 Hz, 1H), 5.13 (dd, *J* = 7.4, 4.8 Hz, 1H), 4.42-4.29 (m, 4H), 4.04 (s, 3H), 4.00-3.92 (m, 1H), 3.82-3.73 (m, 1H), 2.79 (s, 1H), 0.81 (s, 9H), -0.16 (s, 3H), -0.38 (s, 3H); $^{13}C\{^1H\}$

NMR (150 MHz, $CDCl_3$, 298 K) δ (ppm): 155.7, 153.5, 151.5, 149.6, 141.9, 123.8, 116.6, 110.2, 91.5, 87.7, 74.2, 72.9, 63.5, 35.2, 29.2, 25.6, 18.0, -5.1, -5.3; HRMS (ESI) *m/z*: $[M+H]^+$ Calcd. for $C_{20}H_{32}N_7O_5Si$ 478.2229; Found 478.2231.

General procedure for the synthesis of 13a-j:

The 3',5'-deprotected adenosine derivative **12a**-**j** (1.0 equiv.) was dissolved in pyridine and DMTrCl (1.5 equiv.) was added. The reaction mixture was stirred at r.t. for 16 h and afterwards the solvents were removed *in vacuo*. Purification by silica gel column chromatography with an addition of 0.1% pyridine afforded the DMTr-protected adenosine derivative **13a**-**j** as a white or pale-yellow foam.

13a: Yield: 86%; R_f = 0.16 (1:1 *i*-Hexane/EtOAc); IR (ATR) $\tilde{\nu}$ (cm^{-1}): 3320 (w), 2929 (w), 2853 (w), 1749 (w), 1681 (m), 1606 (w), 1568 (m), 1510 (s), 1466 (m), 1345 (s), 1300 (w), 1250 (s), 1213 (m), 1176 (s), 1066 (w), 1034 (s), 1005 (w), 916 (w), 856 (m), 834 (s), 782 (m), 699 (m); ^1H NMR (400 MHz, acetone-d_6, 298 K) δ (ppm): 10.85 (t, J = 5.6 Hz, 1H), 8.49 (s, 1H), 8.46 (s, 1H), 8.12 (d, J = 8.7 Hz, 2H), 7.58 (d, J = 8.7 Hz, 2H), 7.53-7.47 (m, 2H), 7.37 (dd, J = 9.0, 2.3 Hz, 4H), 7.32-7.19 (m, 3H), 6.86 (dd, J = 9.0, 2.3 Hz, 4H), 6.18 (d, J = 4.4 Hz, 1H), 5.07 (t, J = 4.4 Hz, 1H), 4.54-4.50 (m, 1H), 4.43 (t, J = 6.4 Hz, 2H), 4.31-4.26 (m, 1H), 4.11 (d, J = 5.8 Hz, 2H), 3.98 (d, J = 5.8 Hz, 1H), 3.93 (s, 3H), 3.77 (s, 6H), 3.49-3.43 (m, 2H), 3.13 (t, J = 6.4 Hz, 2H), 0.86 (s, 9H), 0.07 (s, 3H), -0.03 (s, 3H); $^{13}C\{^1H\}$ NMR (100 MHz, acetone-d_6, 298 K) δ (ppm): 170.8, 159.6, 156.5, 153.7, 153.2, 150.7, 147.6, 146.1, 141.8, 136.7, 131.1, 131.0, 129.0, 128.6, 127.6, 124.2, 123.2, 113.9, 89.9, 87.1, 84.7, 76.5, 71.9, 65.1, 64.3, 55.5, 43.4, 35.3, 34.8, 26.1, 18.7, -4.6, -4.8; HRMS (ESI) *m/z*: $[M+H]^+$ Calcd. for $C_{49}H_{58}N_7O_{11}Si$ 948.3958; Found 948.3949.

13b: Yield: 85%; R_f = 0.70 (4:1 DCM/EtOAc); IR (ATR) $\tilde{\nu}$ (cm^{-1}): 2928 (w), 1741 (w), 1681 (w), 1610 (m), 1568 (m), 1508 (s), 1463 (m), 1344 (m), 1251 (s), 1174 (m), 1018 (m), 835 (s), 781 (m); ^1H NMR (400 MHz, CD_2Cl_2, 298 K) δ (ppm) 10.91 (d, J = 6.5 Hz, 1H), 8.44 (s, 1H), 8.17 (s, 1H), 8.10 (d, J = 8.7 Hz, 2H), 7.49-7.44 (m, 2H), 7.42 (d, J = 8.7 Hz, 2H), 7.35 (d, J = 8.9 Hz, 4H), 7.32-7.20 (m, 3H), 6.82 (d, J = 8.9 Hz, 4H), 6.08 (d, J = 4.9 Hz, 1H), 4.97 (t, J = 4.9 Hz, 1H), 4.58-4.45 (m, 1H), 4.45-4.33 (m, 3H), 4.25-4.20 (m, 1H), 3.92 (s, 3H), 3.77 (s, 6H), 3.49 (dd, J = 10.7, 3.1 Hz, 1H), 3.39 (dd, J = 10.7, 4.2 Hz, 1H), 3.09 (t, J = 6.5 Hz, 2H), 2.64 (br s, 1H), 1.44 (d, J = 7.2 Hz, 3H), 0.86 (s, 9H), 0.02 (s, 3H), -0.09 (s, 3H); $^{13}C\{^1H\}$ NMR (100 MHz, CD_2Cl_2, 298 K) δ (ppm): 173.7, 155.7, 153.7, 152.8, 150.5, 147.4, 146.5, 145.4, 140.6, 136.2, 130.6, 130.4, 128.6, 128.4, 127.4, 124.0, 123.1, 113.7, 89.2, 87.1, 84.6, 76.1, 71.9, 65.0, 63.9, 55.8, 50.5, 35.4, 34.9, 25.9, 18.5, 18.4, -4.6, -4.9; HRMS (ESI) *m/z:* $[M+H]^+$ Calcd. for $C_{50}H_{60}N_7O_{11}Si$ 962.4115; Found 962.4128.

13c: Yield: 75%; R_f = 0.15 (2:1 *i*-Hexane/EtOAc); IR (ATR) $\tilde{\nu}$ (cm^{-1}): 2950 (w), 2850 (w), 1730 (w), 1670 (w), 1607 (m), 1577 (s), 1508 (s), 1464 (w), 1347 (s), 1250 (s), 1177 (s), 1150 (w), 1090 (s), 1035 (m), 981 (w), 913 (s), 866 (s), 839 (s), 701 (s); ^1H NMR (400 MHz, acetone-d_6, 298 K) δ (ppm): 11.03 (d, J = 7.7 Hz, 1H), 8.52 (s, 1H), 8.49 (s, 1H), 8.09 (d, J = 8.7 Hz, 2H), 7.58 (d, J = 8.7 Hz, 2H), 7.52-7.48 (m, 2H), 7.41-7.34 (m, 4H), 7.29-7.16 (m, 3H), 6.86 (dd, J = 9.0, 2.7 Hz, 4H), 6.19 (d, J = 4.3 Hz, 1H), 5.06 (t, J = 4.3 Hz, 1H), 4.54-4.37 (m, 4H), 4.32-4.28 (m, 1H), 3.97 (d, J = 5.9 Hz, 1H), 3.93 (s, 3H), 3.77 (s, 6H), 3.48-3.44 (m, 2H), 3.14 (t, J = 6.3 Hz, 2H), 2.81-2.80 (m, 2H), 0.98 (d, J = 6.8 Hz, 3H), 0.94 (d, J = 6.8 Hz, 3H), 0.87 (s, 9H), 0.08 (s, 3H), -0.01 (s, 3H); $^{13}C\{^1H\}$ NMR (100 MHz, acetone-d_6, 298 K) δ (ppm): 172.4, 159.5, 156.2, 153.8, 153.1, 150.5, 147.5, 147.4, 146.1, 141.8, 136.7, 136.6, 131.0, 129.1, 128.9, 128.6, 127.5, 124.1, 123.2, 113.9, 90.0, 87.1, 84.6, 76.5, 71.8, 65.0, 64.3, 60.5, 55.5, 35.3, 34.8, 31.4, 26.1, 19.7, 18.7, 18.4, -4.6, -4.8; HRMS (ESI) *m/z*: $[M+H]^+$ Calcd. for $C_{52}H_{64}N_7O_{11}Si$ 990.4428; Found 990.4430.

13d: Yield: 72%; R_f = 0.20 (2:1 *i*-Hexane/EtOAc); IR (ATR) $\tilde{\nu}$ (cm^{-1}): 2950 (w), 2852 (w), 1729 (w), 1670 (w), 1607 (s), 1577 (s), 1508 (s), 1464 (w), 1347 (s), 1250 (s), 1177 (s), 1152 (w), 1091 (s), 1035 (s), 981 (w), 913 (s), 866 (s), 839 (s), 699 (s); ^1H NMR (400 MHz, acetone-d_6, 298 K) δ (ppm): 10.89 (d, J = 7.1 Hz, 1H), 8.50 (s, 1H), 8.49 (s, 1H), 8.10 (d, J = 8.8 Hz, 2H), 7.58 (d, J = 8.8 Hz, 2H), 7.50 (d, J = 7.2 Hz, 2H), 7.38 (dd, J = 9.0, 2.5 Hz, 4H), 7.31-7.25 (m, 2H), 7.25-7.19 (m, 1H), 6.86 (dd, J = 9.0, 2.5 Hz, 4H), 6.18 (d, J = 4.3 Hz, 1H), 5.05 (t, J = 4.3 Hz, 1H), 4.53-4.37 (m, 4H), 4.29 (dd, J = 4.3, 4.3 Hz, 1H), 3.97 (d, J = 5.9 Hz, 1H), 3.91 (s, 3H), 3.77 (s, 6H), 3.51-3.42 (m, 2H), 3.14 (t, J = 6.3 Hz, 2H), 1.74-1.56 (m, 3H), 0.92 (d, J = 2.0 Hz, 3H), 0.92 (d, J = 2.0 Hz, 3H), 0.86 (s, 9H), 0.07 (s, 3H), -0.02 (s, 3H); $^{13}C\{^1H\}$ NMR (100 MHz, acetone-d_6, 298 K) δ (ppm): 173.4, 159.6, 156.0, 153.8, 153.1, 150.6, 147.6, 147.5, 146.1, 141.9, 136.7, 131.1, 131.0, 131.0, 129.1, 129.0, 128.6, 127.6, 124.1, 123.3, 113.9, 90.0, 87.1, 84.7, 76.5, 71.9, 65.1, 64.3, 55.5, 53.7, 41.7, 35.3, 34.8, 26.1, 25.8, 23.1, 22.2, 18.7, -4.6, -4.8; HRMS (ESI) *m/z*: $[M+H]^+$ Calcd. for $C_{53}H_{66}N_7O_{11}Si$ 1004.4584; Found 1004.4579.

13e: Yield: 68%; R_f = 0.22 (2:1 *i*-Hexane/EtOAc); IR (ATR) $\tilde{\nu}$ (cm^{-1}): 2908 (w), 1757 (w), 1718 (w), 1670 (w), 1608 (w), 1507 (s), 1441 (w), 1294 (w), 1248 (s), 1177 (s), 1090 (s), 1034 (s), 975 (s), 913 (s), 869 (s), 776 (s), 703 (s); ^1H NMR (400 MHz, acetone-d_6, 298 K) δ (ppm): 10.89 (d, J = 8.7 Hz, 1H), 8.50 (s, 1H), 8.42 (s, 1H), 8.03 (d, J = 8.7 Hz, 2H), 7.52 (d, J = 8.8 Hz, 4H), 7.41-7.32 (m, 4H), 7.28 (t, J = 7.4 Hz, 2H), 7.25-7.17 (m, 1H), 6.85 (dd, J = 8.8, 1.8 Hz, 4H), 6.20 (d, J = 4.5 Hz, 1H), 5.11 (t, J = 4.5 Hz, 1H), 4.54-4.48 (m, 3H), 4.45-4.31 (m, 2H), 4.31-4.26

(m, 1H), 3.98 (d, J = 5.7 Hz, 1H), 3.95 (s, 3H), 3.76 (s, 6H), 3.48 (qd, J = 10.5, 4.1 Hz, 2H), 3.12 (t, J = 6.3 Hz, 2H), 1.25 (d, J = 6.3 Hz, 3H), 0.88 (s, 9H), 0.86 (s, 9H), 0.07 (s, 6H), 0.03 (s, 6H); $^{13}C\{^1H\}$ NMR (100 MHz, acetone-d_6, 298 K) δ (ppm): 171.7, 159.6, 153.1, 150.4, 142.0, 136.7, 131.1, 131.0, 129.0, 128.6, 127.6, 124.1, 123.4, 113.9, 90.0, 87.1, 84.8, 76.4, 71.9, 69.7, 65.5, 64.4, 61.2, 55.5, 35.3, 35.1, 26.1, 26.0, 21.6, 18.7, 18.4, -4.2, -4.6, -4.8, -5.2; HRMS (ESI) m/z: $[M+H]^+$ Calcd. for $C_{57}H_{76}N_7O_{12}Si_2$ 1106.5085; Found: 1106.5103.

13f: Yield: 80%; R_f = 0.30 (6:4 DCM/EtOAc); IR (ATR) $\tilde{\nu}$ (cm^{-1}): 2930 (w), 1743 (w), 1680 (m), 1582 (s), 1509 (m), 1391 (m), 1345 (s), 1249 (s), 1174 (s), 782 (m); ^1H NMR (400 MHz, acetone-d_6, 298 K) δ (ppm): 8.37 (s, 1H), 8.33 (s, 1H), 8.18-8.16 (m, 2H), 7.56 (br s, 2H), 7.49 (d, J = 7.3 Hz, 2H), 7.37-7.35 (m, 4H), 7.28 (dd, J = 7.3, 7.3 Hz, 2H), 7.21 (t, J = 7.3 Hz, 1H), 6.88-6.84 (m, 4H), 6.11 (d, J = 4.8 Hz, 1H), 5.10 (dd, J = 4.8, 4.8 Hz, 1H), 4.53-4.49 (m, 1H), 4.45-4.38 (m, 2H), 4.25 (dd, J = 8.2, 4.8 Hz, 1H), 3.94 (d, J = 4.8 Hz, 1H), 3.78 (s, 6H), 3.50-3.39 (m, 6H), 3.12-3.10 (m, 2H), 1.84-1.79 (m, 2H), 1.71 (br s, 1H), 0.83 (s, 9H), 0.04 (s, 3H), -0.08 (s, 3H) (some proton signals of proline appeared too broad for an unequivocal assignment); $^{13}C\{^1H\}$ NMR (100 MHz, acetone-d_6, 298 K) δ (ppm): 172.6, 159.6, 153.8, 152.9, 152.2, 147.7, 147.3, 146.0, 141.7, 136.7, 136.6, 131.0, 130.9, 129.1, 128.6, 127.6, 124.3, 113.9, 89.5, 87.1, 84.8, 84.7, 76.3, 72.0, 71.9, 65.2, 64.3, 60.7, 55.5, 48.4, 35.3, 34.6, 30.4, 26.1, 18.7, -4.7, -4.9; HRMS (ESI) m/z: $[M+H]^+$ Calcd. for $C_{52}H_{62}N_7O_{11}Si$ 988.4270; Found 988.4280.

13g: Yield: 90%; R_f = 0.50 (5:1 DCM/EtOAc); IR (ATR) $\tilde{\nu}$ (cm^{-1}): 3538 (w), 2953 (w), 2855 (w), 1738 (w), 1681 (w), 1568 (s), 1508 (s), 1463 (s), 1344 (s), 1249 (s), 1174 (s), 1031 (s), 1016 (s), 834 (s), 781 (s); ^1H NMR (400 MHz, CD_2Cl_2, 298 K) δ (ppm): 10.89 (d, J = 6.8 Hz, 1H), 8.22 (s, 1H), 8.15 (s, 1H), 8.07 (d, J = 8.7 Hz, 2H), 7.50-7.45 (m, 2H), 7.40-7.16 (m, 12H), 7.14 (dd, J = 7.3, 2.1 Hz, 2H), 6.82 (d, J = 8.9 Hz, 4H), 6.06 (d, J = 5.0 Hz, 1H), 4.97 (t, J = 5.0 Hz, 1H), 4.79-4.75 (m, 1H), 4.41-4.35 (m, 3H), 4.26-4.20 (m, 1H), 3.89 (s, 3H), 3.76 (s, 6H), 3.48 (dd, J = 10.7, 3.1 Hz, 1H), 3.38 (dd, J = 10.7, 4.2 Hz, 1H), 3.12 (d, J = 6.3 Hz, 2H), 3.03 (t, J = 6.5 Hz, 2H), 2.64 (d, J = 4.8 Hz, 1H), 0.86 (s, 9H), 0.02 (s, 3H), -0.10 (s, 3H); $^{13}C\{^1H\}$ NMR (100 MHz, CD_2Cl_2, 298 K) δ (ppm): 172.4, 159.2, 155.9, 153.5, 152.7, 150.3, 150.2, 147.3, 146.5, 145.4, 140.6, 140.1, 137.2, 136.2, 136.2, 130.6, 130.6, 130.4, 129.9, 129.6, 129.0, 128.6, 128.4, 128.3, 128.2, 127.6, 127.4, 124.0, 123.0, 113.7, 113.6, 89.1, 87.1, 84.6, 76.1, 71.9, 65.1, 64.0, 56.3, 55.7, 38.3, 35.3, 34.9, 25.9, 18.4, -4.6, -4.9; HRMS (ESI) m/z: $[M+H]^+$ Calcd. for $C_{56}H_{64}N_7O_{11}Si$ 1038.4428; Found 1038.4447.

13h: Yield: 92%; R_f = 0.25 (100:5 DCM/EtOAc); ^1H NMR (400 MHz, acetone-d_6, 298 K) δ (ppm): 10.98 (d, J = 7.1 Hz, 1H), 8.50 (s, 1H), 8.49 (s, 1H), 8.14-8.07 (d, J = 8.9 Hz, 2H), 7.59 (d, J = 8.6 Hz, 2H), 7.50 (d, J = 7.2 Hz, 2H), 7.37 (dd, J = 9.0, 2.7 Hz, 4H), 7.29 (t, J = 7.4 Hz, 2H), 7.22 (t, J = 7.2 Hz, 1H), 6.86 (dd, J = 8.9, 3.2 Hz, 4H), 6.18 (d, J = 4.3 Hz, 1H), 5.07 (t, J = 4.6 Hz, 1H), 4.61 (td, J = 7.5, 5.3 Hz, 1H), 4.52 (q, J = 5.4 Hz, 1H), 4.47 (td, J = 6.1, 4.3 Hz, 2H), 4.29 (q, J = 4.4 Hz, 1H), 3.98 (d, J = 5.9 Hz, 1H), 3.91 (s, 3H), 3.77 (s, 6H), 3.47 (dd, J = 4.1, 2.1 Hz, 2H), 3.16 (t, J = 6.3 Hz, 2H), 2.53 (td, J = 7.2, 1.5 Hz, 2H), 2.17-1.95 (m, 5H), 0.86 (s, 9H), 0.07 (s, 3H), -0.03 (s, 3H); $^{13}C\{^1H\}$ NMR (100 MHz, acetone-d_6, 298 K) δ (ppm): 172.6, 159.6, 159.6, 156.0, 153.7, 153.1, 150.7, 150.6, 147.6, 147.5, 146.1, 141.9, 136.7, 136.7, 131.0, 131.0, 131.0, 129.0, 128.6, 127.6, 124.6, 124.1, 123.3, 113.9, 90.0, 87.1, 84.7, 76.4, 71.9, 65.3, 64.3, 55.5, 54.1, 35.3, 34.8, 32.2, 30.7, 26.1, 18.7, 15.2, -4.6, -4.8; HRMS (ESI) m/z: $[M+H]^+$ Calcd. for $C_{52}H_{64}N_7O_{11}SSi$ 1022.4148; Found 1022.4137.

13i: Yield: 80%; R_f = 0.25 (95:5 DCM/EtOAc); IR (ATR) $\tilde{\nu}$ (cm^{-1}): 2932 (w), 1737 (m), 1682 (m), 1571 (m), 1518 (s), 1464 (s), 1251 (s), 1176 (s), 1018 (m), 836 (s); ^1H NMR (400 MHz, acetone-d_6, 298 K) δ (ppm): 11.21 (d, J = 7.5 Hz, 1H), 8.49 (s, 1H), 8.39 (s, 1H), 8.09-8.04 (m, 4H), 7.55-7.49 (m, 6H), 7.39-7.35 (m, 4H), 7.29 (dd, J = 7.5, 7.5 Hz, 2H), 7.21 (t, J = 7.5 Hz, 1H), 6.88-6.83 (m, 4H), 6.18 (d, J = 4.2 Hz, 1H), 5.04 (dd, J = 4.2, 4.2 Hz, 1H), 4.82 (dt, J = 7.5, 5.3 Hz, 1H), 4.53 (dd, J = 4.2, 4.2 Hz, 1H), 4.44-4.27 (m, 5H), 3.96 (d, J = 6.1 Hz, 1H), 3.90 (s, 3H), 3.77 (s, 6H), 3.51-3.44 (m, 2H), 3.11 (t, J = 6.2 Hz, 2H), 3.06 (t, J = 6.2 Hz, 2H), 2.96-2.94 (m, 2H), 0.86 (s, 9H), 0.08 (s, 3H), -0.01 (s, 3H); $^{13}C\{^1H\}$ NMR (100 MHz, acetone-d_6, 298 K) δ (ppm): 171.5, 171.2, 159.6, 155.9, 153.5, 153.1, 150.4, 147.6, 147.5, 147.4, 147.2, 146.1, 141.9, 136.7, 131.0 (×2), 130.9, 129.0, 128.6, 127.6, 124.1, 124.0, 123.2, 113.9, 90.1, 87.1, 84.5, 76.5, 71.8, 65.6, 65.0, 64.2, 55.5, 51.4, 37.1, 35.3, 35.2, 34.8, 26.1, 18.7, -4.6, -4.8; HRMS (ESI) m/z: $[M+H]^+$ Calcd. for $C_{59}H_{67}O_{15}N_8Si$ 1155.4489; Found 1155.4504.

13j: Yield: 86%; R_f = 0.21 (5:2 *i*-Hexane/EtOAc); IR (ATR) $\tilde{\nu}$ (cm^{-1}): 3397 (w), 2954 (m), 2926 (s), 2854 (m), 2168 (w), 1682 (m), 1607 (m), 1569 (s), 1508 (s), 1462 (s), 1445 (m), 1362 (w), 1297 (w), 1249 (s), 1174 (s), 1134 (m), 1032 (s), 994 (m), 904 (w), 833 (s), 781 (m), 700 (m); ^1H NMR (600 MHz, $CDCl_3$, 298 K) δ (ppm): 11.02 (t, J = 5.7 Hz, 1H), 8.50 (s, 1H), 8.48 (s, 1H), 7.50 (d, J = 7.2 Hz, 2H), 7.38-7.36 (m, 4H), 7.29 (t, J = 7.6 Hz, 2H), 7.24-7.21 (m, 1H), 6.87-6.83 (m, 4H), 6.18 (d, J = 4.6 Hz, 1H), 5.08 (dd, J = 4.6, 4.6 Hz, 1H), 4.54-4.49 (m, 1H), 4.39 (d, J = 5.7 Hz, 2H), 4.28 (td, J = 4.7, 3.4 Hz, 1H), 3.98 (s, 3H), 3.96 (d, J = 5.8 Hz, 1H), 3.78 (s, 6H), 3.48 (dd, J = 10.2, 3.8 Hz, 1H), 3.45 (dd, J = 10.2, 4.7 Hz, 1H), 0.85 (s, 9H), 0.06 (s, 3H), -0.05 (s, 3H); $^{13}C\{^1H\}$ NMR (150 MHz, $CDCl_3$, 298 K) δ (ppm): 159.6, 159.6, 156.5, 153.5, 153.4, 150.7, 146.1, 142.2, 142.2, 136.7, 131.0, 131.0, 129.0, 129.0, 128.6, 127.6, 123.3, 118.2, 113.9, 89.9, 84.8, 76.4, 71.9, 64.4, 55.5, 55.5, 34.9, 29.7, 26.1, 18.7, -4.6, -4.8; HRMS (ESI) m/z: $[M+H]^+$ Calcd. for $C_{41}H_{50}N_7O_7Si$ 780.3535; Found 780.3538.

General procedure for the synthesis of 14a-j:

To a solution of 5'-DMTr-protected adenosine derivative **13a**-**j** (1.0 equiv.) in anhydrous DCM, *N*,*N*-diisopropylethylamine (DIPEA) (4.0 equiv.) was added. After cooling down to 0°C, 2-cyanoethyl *N*,*N*-diisopropylchlorophosphoramidite (CED-Cl) (2.5 equiv.) was added dropwise and the reaction mixture was stirred at r.t. for 5 h. Afterwards aq. sat. $NaHCO_3$ solution was added to the reaction mixture and the aqueous phase was extracted three times with DCM. The combined organic layers were dried ($MgSO_4$), filtered and concentrated *in vacuo*. The crude product was purified by silica gel column chromatography with addition of 0.1% pyridine and co-lyophilized from benzene to afford the desired phosphoramidite **14a**-**j** as a mixture of diastereoisomers, as a white or pale-yellow foam.

14a: Yield: 85%; R_f = 0.15 (2:1 *i*-Hexane/EtOAc); $^{31}P\{^1H\}$ NMR (162 MHz, acetone-d_6, 298 K) δ (ppm): 150.1, 148.7; HRMS (ESI) *m*/*z*: $[M+H]^+$ Calcd. for $C_{58}H_{75}N_9O_{12}PSi$ 1148.5037; Found 1148.5052.

14b: Yield: 85%; R_f = 0.50 (1:1 *i*-Hexane/EtOAc); $^{31}P\{^1H\}$ NMR (162 MHz, CD_2Cl_2, 298 K) δ (ppm): 150.6, 149.2; HRMS (ESI) *m*/*z*: $[M+H]^+$ Calcd. for $C_{59}H_{77}N_9O_{12}PSi$ 1162.5193; Found 1162.5221.

14c: Yield: 77%; R_f = 0.35 (1:1 *i*-Hexane/EtOAc); $^{31}P\{^1H\}$ NMR (162 MHz, acetone-d_6, 298 K) δ (ppm): 150.1, 148.7; HRMS (ESI) *m*/*z*: $[M+H]^+$ Calcd. for $C_{61}H_{81}N_9O_{12}PSi$ 1190.5506; Found 1190.5492.

14d: Yield: 75%; R_f = 0.38 (1:1 *i*-Hexane/EtOAc); $^{31}P\{^1H\}$ NMR (162 MHz, acetone-d_6, 298 K) δ (ppm): 150.1, 148.7; HRMS (ESI) *m*/*z*: $[M+H]^+$ Calcd. for $C_{62}H_{83}N_9O_{12}PSi$ 1204.5663; Found 1204.5682.

14e: Yield: 62%; R_f = 0.43 (1:1 *i*-Hexane/EtOAc); $^{31}P\{^1H\}$ NMR (162 MHz, acetone-d_6, 298 K) δ (ppm): 150.2, 148.5; HRMS (ESI) *m*/*z*: $[M+H]^+$ Calcd. for $C_{66}H_{93}N_9O_{13}PSi_2$ 1306.6164; Found 1306.6189.

14f: Yield: 80%; R_f = 0.30 (6:4 DCM/EtOAc); $^{31}P\{^1H\}$ NMR (162 MHz, acetone-d_6, 298 K) δ (ppm) 150.2, 148.6; HRMS (ESI) *m*/*z*: $[M+H]^+$ Calcd. for $C_{61}H_{79}N_9O_{12}PSi$ 1188.5350; Found 1188.5388.

14g: Yield: 90%; R_f = 0.30 (5:3 *i*-Hexane/EtOAc); $^{31}P\{^1H\}$ NMR (162 MHz, acetone-d_6, 298 K) δ (ppm) 150.7, 149.1; HRMS (ESI) *m*/*z*: $[M+H]^+$ Calcd. for $C_{65}H_{81}N_9O_{12}PSi$ 1238.5506; Found 1238.5530.

14h: Yield: 89%; R_f = 0.30 (6:4 DCM/EtOAc); $^{31}P\{^1H\}$ NMR (162 MHz, acetone-d_6, 298 K) δ (ppm) 150.1, 148.6; HRMS (ESI) *m*/*z*: $[M+H]^+$ Calcd. for $C_{61}H_{81}N_9O_{12}PSSi$ 1222.5227; Found 1222.5215.

14i: Yield: 65%; R_f = 0.15 (92:8 DCM/EtOAc); $^{31}P\{^1H\}$ NMR (162 MHz, acetone-d_6, 298 K) δ (ppm): 150.1, 148.7; HRMS (ESI) *m*/*z*: $[M+H]^+$ Calcd. for $C_{68}H_{84}O_{16}N_{10}PSi$ 1355.5567; Found 1355.5590.

14j: Yield: 89%; R_f = 0.39 (2:1 EtOAc/*i*-Hexane); $^{31}P\{^1H\}$ NMR (162 MHz, acetone-d_6, 298 K) δ (ppm): 150.3, 148.6; HRMS (ESI) *m*/*z*: $[M+H]^+$ Calcd. for $C_{50}H_{67}N_9O_8PSi$ 980.4614; Found 980.4611.

2.4 Nucleobase-modified N^6-triglycinylcarbamoyl adenosine nucleoside

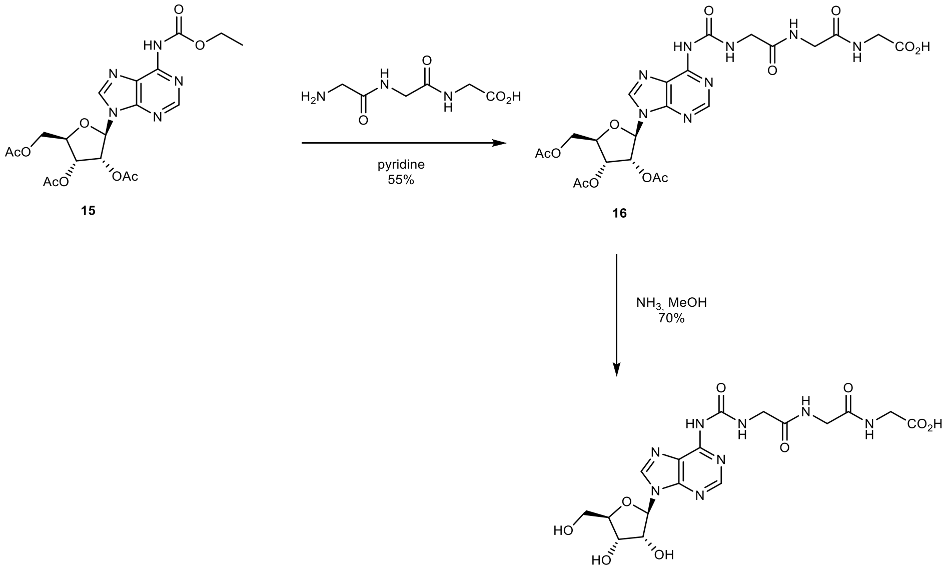

Scheme S4. Synthesis of N^6-triglycinylcarbamoyl adenosine **17**.

The compound **15** was synthesized according to a procedure previously described in the literature.[6,7]

Acetyl protected N^6-triglycinylcarbamoyl adenosine 16: Carbamate derivative **15** (0.25 g, 0.54 mmol, 1.0 equiv.) was dissolved in dry pyridine and **H-Gly-Gly-Gly-OH** (0.20 g, 1.1 mmol, 2.0 equiv.) was added. The mixture was stirred under reflux for 7 h and at r.t. overnight. After that, the crude was filtered and concentrated. The crude was resuspended in toluene and concentrated. Finally, the crude was crystallized from EtOH affording the product **16** as a white solid (0.18 g, 0.30 mmol, 55% yield). IR (ATR) $\tilde{\nu}$ (cm^{-1}): 3353 (w), 1745 (m), 1732 (m), 1697 (m), 1608 (w), 1590 (w), 1515 (s), 1216 (s), 1038 (s), 902 (w); ^{1}H NMR (400 MHz, DMSO-d_6, 298 K) δ (ppm): 12.58 (br s, 1H), 9.94 (s, 1H), 9.66 (t, J = 5.3 Hz, 1H), 8.65 (s, 1H), 8.59 (s, 1H), 8.38 (t, J = 5.8 Hz, 1H), 8.21 (t, J = 5.8 Hz, 1H), 6.30 (d, J = 5.4 Hz, 1H), 6.03 (dd, J = 5.4, 5.4 Hz, 1H), 5.63 (dd, J = 5.4, 5.4 Hz, 1H), 4.44-4.38 (m, 2H), 4.29-4.24 (m, 1H), 3.98 (d, J = 5.3 Hz, 2H), 3.77-3.75 (m, 4H), 2.12 (s, 3H), 2.04 (s, 3H), 2.01 (s, 3H); ^{13}C{^{1}H} NMR (100 MHz, DMSO-d_6, 298 K) δ (ppm): 171.2, 170.1, 169.5, 169.3, 169.2 (x2), 153.5, 151.1, 150.5, 150.1, 142.7, 120.5, 85.8, 79.6, 72.0, 70.0, 62.7, 43.1, 41.8, 40.6, 20.5, 20.4, 20.2; HRMS (ESI) m/z: [M+Na]$^+$ Calcd. for $C_{23}H_{28}O_{12}N_8Na$ 631.1718; Found 631.1721.

N^6-triglycinylcarbamoyl adenosine 17: Protected adenosine derivative **16** (0.12 g, 0.20 mmol, 1.0 equiv.) was dissolved in 7 N NH_3 in MeOH. The reaction was heated at 40°C for 1.5 h and at r.t. overnight. After that, the crude was concentrated. Finally, the crude product was recrystallized from EtOH (5 mL) affording the product as a white solid (67 mg, 0.14 mmol, 70% yield). IR (ATR) $\tilde{\nu}$ (cm^{-1}): 3281 (m), 2936 (w), 1691 (s), 1658 (s), 1551 (s), 1470 (s), 1240 (s), 1058 (s), 794 (m), 690 (s); ^{1}H NMR (400 MHz, DMSO-d_6, 298 K) δ (ppm): 9.70 (t, J = 5.0 Hz, 1H), 8.68 (s, 1H), 8.56 (s, 1H), 8.41 (t, J = 5.7 Hz, 1H), 7.83 (t, J = 5.0 Hz, 1H), 5.98 (d, J = 5.6 Hz, 1H), 4.59 (dd, J = 5.6, 5.6 Hz, 1H), 4.18 (dd, J = 5.6, 5.6 Hz, 1H), 3.99-3.98 (m, 3H), 3.74 (d, J = 5.7 Hz, 2H), 3.71-3.56 (m, 4H); ^{13}C{^{1}H} NMR (100 MHz, DMSO-d_6, 298 K) δ (ppm): 171.2, 169.2, 168.5, 153.6, 150.9, 150.4, 150.3, 142.2, 120.4, 87.7, 85.7, 73.8, 70.3, 61.3, 43.0, 42.1, 42.0; HRMS (ESI) m/z: [M+H]$^+$ Calcd. for $C_{17}H_{23}O_9N_8$ 483.1582; Found 483.1583.

2.5 Nucleobase-modified N^6-methylurea adenosine nucleoside

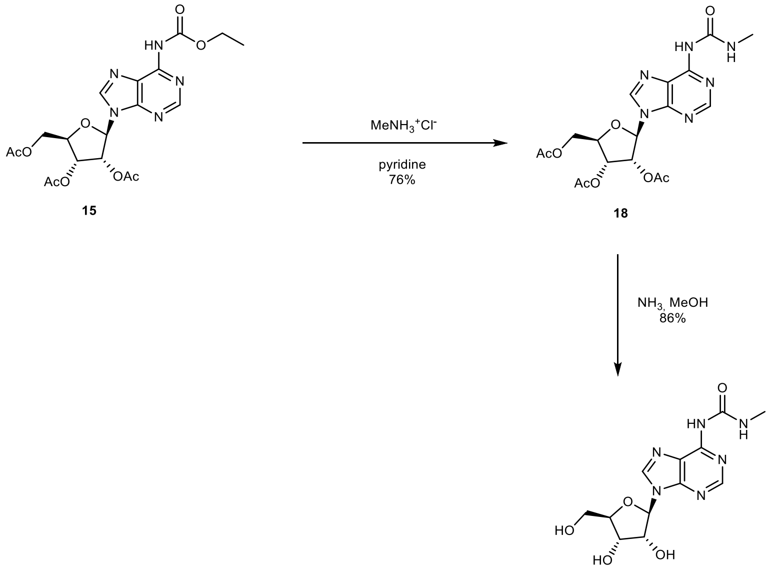

Scheme S5. Synthesis of N^6-methylurea adenosine **19**.

Acetyl protected N^6-methylurea adenosine 18: Carbamate derivative **15** (0.40 g, 0.86 mmol, 1.0 equiv.) was dissolved in dry pyridine and methylammonium chloride (0.17 g, 2.6 mmol, 3.0 equiv.) was added. The mixture was stirred under reflux overnight. After that, the crude was filtered, washed with EtOAc and concentrated. The crude was suspended in toluene and concentrated. Finally, the crude was purified by silica gel column chromatography (20 g, 95:5 DCM/IPA) affording the product **18** as a white foam (0.30 g, 0.65 mmol, 76% yield). IR (ATR) $\tilde{\nu}$ (cm^{-1}): 3246 (w), 1744 (m), 1699 (m), 1590 (m), 1544 (m), 1469 (w), 1365 (w), 1212 (s), 1046 (m), 797 (w); ^1H NMR (400 MHz, DMSO-d_6, 298 K) δ (ppm): 9.74 (s, 1H), 9.20 (c, J = 4.6 Hz, 1H), 8.63 (s, 1H), 8.57 (s, 1H), 6.29 (d, J = 5.3 Hz, 1H), 6.03 (dd, J = 5.3, 5.3 Hz, 1H), 5.64 (dd, J = 5.3, 5.3 Hz, 1H), 4.43-4.23 (m, 3H), 2.83 (d, J = 4.6 Hz, 3H), 2.12 (s, 3H), 2.04 (s, 3H), 2.01 (s, 3H); $^{13}C\{^1H\}$ NMR (100 MHz, DMSO-d_6, 298 K) δ (ppm): 170.1, 169.5, 169.3, 153.9, 151.1, 150.5, 150.0, 142.6, 120.3, 85.8, 79.6, 72.0, 70.0, 62.7, 26.3, 20.5, 20.4, 20.2; HRMS (ESI) *m*/*z*: $[M+H]^+$ Calcd. for $C_{18}H_{23}O_8N_6$ 451.1571; Found 451.1573.

N^6-methylurea adenosine 19: Protected N^6-methylurea adenosine **18** (0.26 g, 0.58 mmol, 1.0 equiv.) was dissolved in 7 N NH_3 in MeOH. The reaction was heated at 40°C for 1.5 h and at r.t. overnight. After that, the crude was concentrated. Finally, the crude was triturated in EtOH, filtered and washed with EtOH affording the product as a white solid (0.16 g, 0.50 mmol, 86% yield). IR (ATR) $\tilde{\nu}$ (cm^{-1}): 3360 (w), 1703 (m), 1584 (m), 1537 (m), 1462 (m), 1297 (m), 1245 (s), 1103 (m), 1057 (m), 795 (m); ^1H NMR (400 MHz, DMSO-d_6, 298 K) δ (ppm): 9.57 (br s, 1H), 9.24 (c, J = 4.5 Hz, 1H), 8.65 (s, 1H), 8.54 (s, 1H), 5.97 (d, J = 5.7 Hz, 1H), 5.53 (d, J = 5.7 Hz, 1H), 5.24 (d, J = 4.8 Hz, 1H), 5.15 (dd, J = 5.7, 5.7 Hz, 1H); 4.62-4.58 (m, 1H), 4.19-4.15 (m, 1H), 3.98-3.95 (m, 1H), 3.71-3.54 (m, 2H), 2.83 (d, J = 4.5 Hz, 3H); $^{13}C\{^1H\}$ NMR (100 MHz, DMSO-d_6, 298 K) δ (ppm): 154.0, 150.8, 150.3, 150.2, 142.2, 120.2, 87.7, 85.7, 73.8, 70.3, 61.3, 26.3; HRMS (ESI) *m*/*z*: $[M+H]^+$ Calcd. for $C_{12}H_{17}O_5N_6$ 325.1254; Found 325.1257.

2.6 Nucleobase-modified N^6-triglycinylcarbamoyl adenosine nucleoside under prebiotic conditions

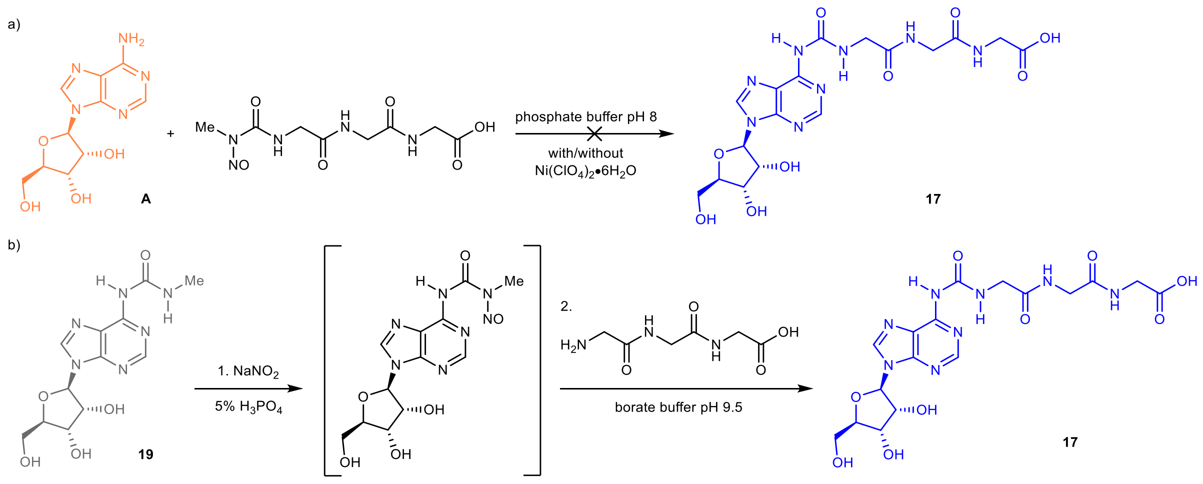

Scheme S6. Synthesis of N^6-triglycinylcarbamoyl adenosine **17** under prebiotic conditions using: a) nitroso derivative of the *N*-methylurea peptide and b) N^6-methylurea adenosine **19**.

Method A:[8] Adenosine **A** (2.67 mg, 10 µmol, 1.0 equiv.) was dissolved in 30 mM phosphate buffer pH 8 (370 µL). The nitroso derivative of the *N*-methylurea peptide (5.50 mg, 20 µmol, 2.0 equiv.) was dissolved in water (40 µL) and added to the adenosine's solution. Either water (40 µL) or $Ni(ClO_4)_2$•$6H_2O$ (91.34 mg, 250 µmol, 25 equiv.) in water (40 µL) was added and the reaction was heated at 70°C for 24 h in a ThermoMixer. Finally, an aliquot (50 µL) of the reaction crude was diluted with water (up to 1 mL), filtered and analyzed by LC-MS (Buffer A: 2 mM $HCOONH_4$ pH 5.5 in H_2O and buffer B: 2 mM $HCOONH_4$ pH 5.5 in 20:80 H_2O/MeCN; Gradient: 0-20% of B in 30 min; Flow rate = 0.15 mL·min^{-1} and Injection: 5 µL).

This prebiotic synthetic method did not afford the N^6-triglycinylcarbamoyl adenosine **17**. We only detected the formation of traces of inosine when using the Ni(II) salt.

Method B: Step 1. N^6-methylurea adenosine **19** (1 mg, 3.08 µmol, 1.0 equiv.) was dissolved in 5% H_3PO_4 in water (140 µL) and cooled to 0°C in an ice bath. $NaNO_2$ (2.66 mg, 38.54 µmol, 12.5 equiv.) was dissolved in water (10 µL) and added to the previous solution. The reaction was incubated at 0°C for 2 h and -20°C for 22 h. After that, the adenosine's solution was allowed to reach 0°C. Step 2. The peptide (5.83 mg, 30.84 µmol, 10 equiv.) was dissolved in 30 mM borate buffer pH 9.5 (3 mL) and cooled down to 0°C. The adenosine's solution was added to the peptide's solution and the pH was adjusted to 9.5 with 4 N NaOH (60 µL). The reaction was stirred at r.t. for 1 h. Finally, an aliquot (25 µL) of the reaction crude was diluted with water (up to 1 mL), filtered and analyzed by LC-MS (Buffer A: 2 mM $HCOONH_4$ pH 5.5 in H_2O and buffer B: 2 mM $HCOONH_4$ pH 5.5 in 20:80 H_2O/MeCN; Gradient: 0-20% of B in 30 min; Flow rate = 0.15 mL·min^{-1} and Injection: 5 µL).

This prebiotic synthetic method afforded the N^6-triglycinylcarbamoyl adenosine **17** in 65% yield. The assignment and amount of the compounds observed in the HPL-chromatogram (Figure S1) was performed by analyzing separate solutions of those synthesized using non-prebiotic methods. Mass spectrometry analyses confirmed the assignments.

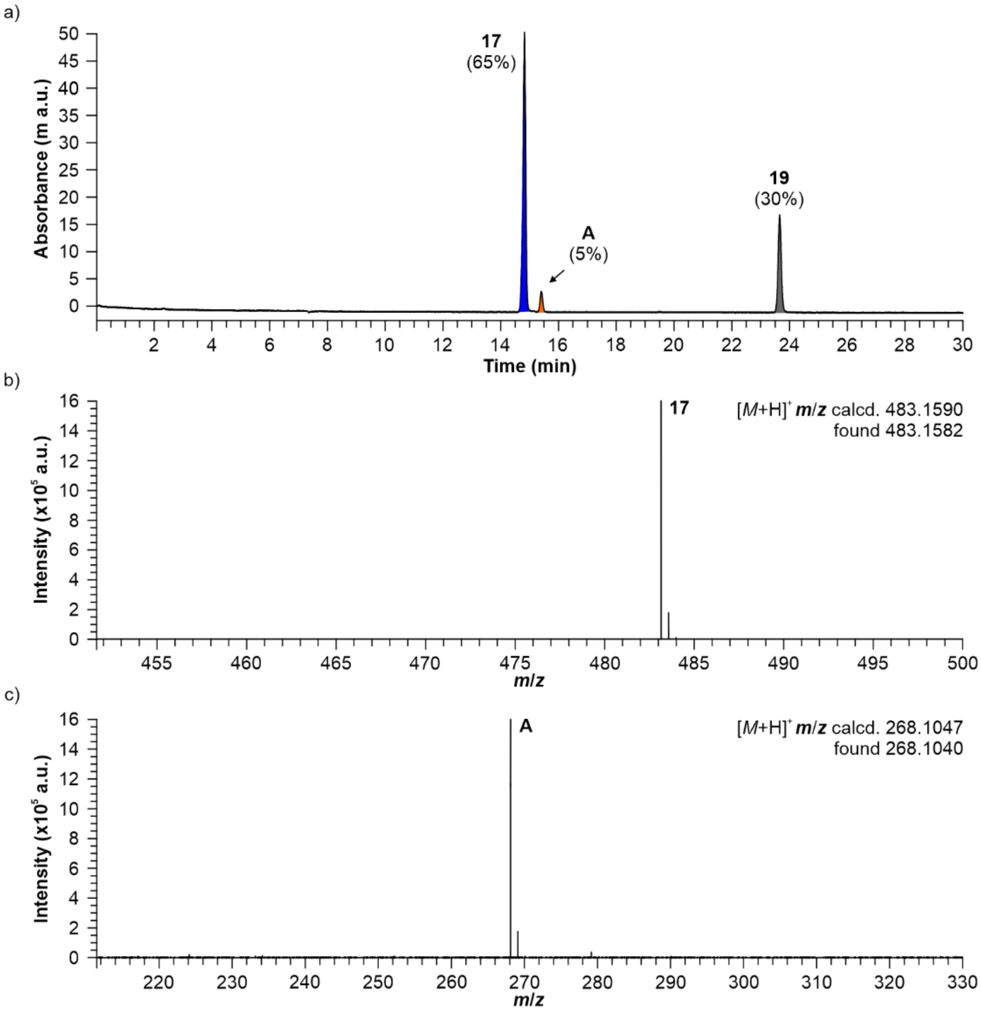

Figure S1. a) HPL-chromatogram of the reaction crude using the **Method B** shown in Scheme S6; mass spectra of the chromatographic peaks observed at: b) 14.8 and c) 15.4 min. The mass spectra confirmed the formation of the compounds **17** and **A**.

2.7 Nucleobase-modified 5-methyluridine 2’-methoxy phosphoramidite

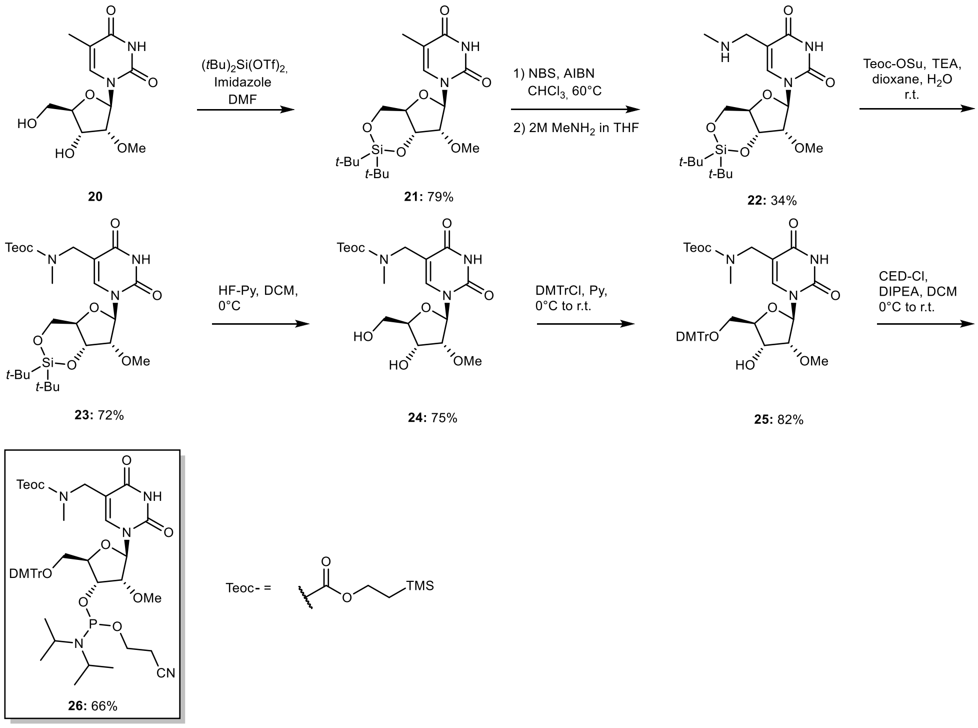

Scheme S7. Synthesis of nucleobase-modified 5-methyluridine 2’-methoxy phosphoramidite.

General procedure for the synthesis of 21:

A suspension of 2′-OMe m^{5}U **20** (1.0 equiv.) in DMF was cooled to 0°C. Di-*tert*-butylsilyl *bis*(trifluoromethanesulfonate) (1.1 equiv.) was added dropwise and the mixture was stirred at r.t. for 30 min. To the reaction was added imidazole (2.5 equiv.) and the resulting solution was stirred at r.t. for 16 h. The crude was concentrated under reduced pressure and the residue was redissolved in EtOAc and washed with water, aq. sat. $NaHCO_3$ solution and brine. The organic layer was dried ($MgSO_4$), filtered and concentrated. The crude was purified by silica gel column chromatography to yield **21** as a white foam.

21: Yield: 79%; R_f = 0.29 (5:1 DCM/EtOAc); IR (ATR) $\tilde{\nu}$ (cm^{-1}): 2933 (w), 2859 (w), 1681 (m), 1471 (m), 1365 (w), 1323 (w), 1266 (w), 1148 (m), 1131 (m), 1064 (s), 1038 (m), 1919 (w), 952 (w), 907 (s), 826 (s), 727 (s); ^{1}H NMR (400 MHz, $CDCl_3$, 298 K) δ (ppm): 7.02 (s, 1H), 5.63 (d, J = 0.8 Hz, 1H), 4.48-4.45 (m, 1H), 4.07-3.95 (m, 3H), 3.94-3.90 (m, 1H), 3.61 (s, 3H), 1.93 (s, 3H), 1.07 (s, 9H), 1.03 (s, 9H); ^{13}C{^{1}H} NMR (100 MHz, $CDCl_3$, 298 K) δ (ppm): 163.6, 149.7, 136.1, 111.2, 91.9, 82.2, 77.4, 74.5, 67.4, 59.3, 27.5, 27.2, 22.9, 20.5, 12.8; HRMS (ESI) *m/z*: [M+H]$^+$ Calcd. for $C_{19}H_{33}N_2O_6Si$ 413.2102; Found 413.2106.

General procedure for the synthesis of 22:

A solution of **21** (1.0 equiv.) in dry $CHCl_3$ was heated at 60°C. *N*-bromosuccinimide (NBS) (1.2 equiv., previously purified by recrystallization) and azobisisobutyronitrile (AIBN) (0.12 equiv.) were added and the reaction was stirred under reflux for 1.5 h. After that, the reaction mixture was cooled to r.t. and $MeNH_2$ (2 M in THF, 5.0 equiv.) was added. The resulting suspension was stirred for 2 h at r.t. and, subsequently, it was diluted with aq. sat. $NaHCO_3$ solution. The crude was extracted three times with DCM. The combined organic layers were dried ($MgSO_4$), filtered and concentrated. The crude was purified by silica gel column chromatography to furnish **22** as a yellow foam.

22: Yield: 34%; R_f = 0.30 (9:1 DCM/IPA); IR (ATR) $\tilde{\nu}$ (cm^{-1}): 2934 (w), 2859 (w), 1680 (s), 1468 (m), 1245 (s), 1201 (w), 1132 (m), 1064 (m), 1034 (m), 961 (w), 852 (w), 826 (s), 735 (w); ^{1}H NMR (400 MHz, acetone-d_6, 298 K) δ (ppm): 8.02 (s, 1H), 5.81 (s, 1H), 4.43-4.32 (m, 2H), 4.25-4.18 (m, 1H), 4.11 (d, J = 5.0 Hz, 1H), 4.08-3.98 (m, 2H), 3.84 (d, J = 7.0 Hz, 2H), 3.58 (s, 3H), 2.62 (s, 3H), 1.07 (s, 9H), 1.03 (s, 9H); ^{13}C{^{1}H} NMR (100 MHz, acetone-d_6, 298 K) δ (ppm): 163.9, 150.5, 142.6, 107.5, 91.7, 82.9, 77.8, 75.4, 67.8, 59.2, 45.8, 33.3, 27.8, 27.5, 23.1, 20.9; HRMS (ESI) *m/z*: [M+H]$^+$ Calcd. for $C_{20}H_{36}N_3O_6Si$ 442.2368; Found 442.2370.

General procedure for the synthesis of 23:

To a solution of **22** (1.0 equiv.) in 1,4-dioxane and H_2O (1:1 v/v) were added teoc-OSu (1.1 equiv.) and triethylamine (TEA) (1.5 equiv.). The mixture was stirred at r.t. for 16 h. After that, the crude was diluted with water and extracted three times with Et_2O. The combined organic layers were washed with water, dried ($MgSO_4$), filtered and concentrated. The obtained residue was purified by silica gel column chromatography to yield the teoc-protected compound **23** as a white solid.

23: Yield: 72%; R_f = 0.53 (95:5 DCM/IPA); IR (ATR) $\tilde{\nu}$ (cm^{-1}): 2948 (w), 2894 (w), 2859 (w), 1725 (m), 1464 (w), 1384 (w), 1280 (w), 1245 (s), 1198 (m), 1139 (m), 1057 (m), 1029 (m), 955 (w), 920 (w), 826 (s), 744 (m), 691 (w); For major rotamer: ^{1}H NMR (400 MHz, acetone-*d*$_6$, 298 K) δ (ppm): 10.27 (br s, 1H), 7.54 (s, 1H), 5.76 (s, 1H), 4.47 (d, *J* = 4.1 Hz, 1H), 4.27-3.95 (m, 8H), 3.59 (s, 3H), 2.94 (s, 3H), 1.08 (s, 9H), 1.04-1.00 (m, 11H), 0.06 (s, 9H); ^{13}C{^{1}H} NMR (100 MHz, acetone-*d*$_6$, 298 K) δ (ppm): 150.6, 139.7, 111.1, 91.4, 83.0, 77.7, 75.4, 68.1, 63.8, 59.2, 45.5, 35.3, 27.8, 27.5, 23.2, 20.9, 18.4, -1.3; HRMS (ESI) *m*/*z*: $[M+H]^+$ Calcd. for $C_{26}H_{48}N_3O_8Si_2$ 586.2975; Found 586.2981.

General procedure for the synthesis of 24:

The modified 2′-OMe 5-methyluridine **23** (1.0 equiv.) was dissolved in DCM/pyridine (9:1 v/v) and cooled to 0°C in a plastic reaction vessel. Subsequently, a solution of 70% HF-pyridine (5.0 equiv.) was slowly added, and the reaction mixture was stirred at 0°C for 2 h. The reaction was quenched by adding aq. sat. $NaHCO_3$ and the crude was extracted three times with DCM. The combined organic layers were washed with water, dried ($MgSO_4$), filtered and concentrated. The crude product was purified by silica gel column chromatography to afford the diol compound **24** as a white foam.

24: Yield: 75%; R_f = 0.22 (100:5 DCM/MeOH); IR (ATR) $\tilde{\nu}$ (cm^{-1}): 3060 (w), 2951 (w), 1710 (m), 1463 (m), 1401 (m), 1249 (s), 1214 (m), 1114 (m), 1086 (m), 1062 (m), 988 (w), 938 (w), 838 (s), 769 (m), 694 (w); For major rotamer: ^{1}H NMR (400 MHz, $CDCl_3$, 298 K) δ (ppm): 10.17 (br s, 1H), 8.09 (s, 1H), 5.99 (d, *J* = 4.3 Hz, 1H), 4.34 (s, 1H), 4.28-4.12 (m, 3H), 4.11-3.92 (m, 5H), 3.92-3.74 (m, 2H), 3.47 (s, 3H), 2.95 (s, 3H), 1.02 (s, 2H), 0.04 (s, 9H) (some proton signals appeared too broad for an unequivocal assignment); ^{13}C{^{1}H} NMR (100 MHz, $CDCl_3$, 298 K) δ (ppm): 163.9, 157.2, 151.2, 140.3, 138.6, 111.0, 87.9, 84.4, 69.9, 63.9, 62.1, 58.5, 45.8, 35.3, 18.3, -1.4 (some carbon signals appeared too broad for an unequivocal assignment); HRMS (ESI) *m*/*z*: $[M+H]^+$ Calcd. for $C_{18}H_{32}N_3O_8Si$ 446.1953; Found 446.1954.

General procedure for the synthesis of 25:

To a solution of the 2′-OMe 3′,5′-deprotected 5-methyluridine derivative **24** (1.0 equiv.) in pyridine was added 4,4′-dimethoxytrityl chloride (DMTrCl) (1.5 equiv.). After stirring at r.t. for 16 h, the reaction mixture was concentrated and purified by silica gel column chromatography with an addition of 0.1% of pyridine to the eluent to afford the DMTr-protected compound **25** as a white foam.

25: Yield: 82%; R_f = 0.34 (1:1 DCM/EtOAc); IR (ATR) $\tilde{\nu}$ (cm^{-1}): 2953 (w), 1694 (m), 1607 (w), 1508 (m), 1461 (m), 1397 (w), 1344 (w), 1298 (w), 1245 (s), 1175 (m), 1166 (m), 1063 (m), 1032 (s), 962 (w), 832 (s), 756 (w), 726 (w); For major rotamer: ^{1}H NMR (400 MHz, acetone-*d*$_6$, 298 K) δ (ppm): 10.20 (br s, 1H), 7.74 (s, 1H), 7.60-7.50 (m, 2H), 7.46-7.38 (m, 4H), 7.32 (t, *J* = 7.8 Hz, 2H), 7.25-7.20 (m, 1H), 6.89 (d, *J* = 8.9 Hz, 4H), 5.95 (s, 1H), 4.45-4.21 (m, 1H), 4.17-3.90 (m, 4H), 3.87-3.66 (m, 8H), 3.59-3.37 (m, 5H), 2.88 (s, 3H), 1.00-0.91 (m, 2H), 0.02 (s, 9H) (some proton signals appeared too broad for an unequivocal assignment); ^{13}C{^{1}H} NMR (100 MHz, acetone-*d*$_6$, 298 K) δ (ppm): 163.9, 159.6, 156.8, 151.0, 146.1, 140.4, 136.9, 131.1, 129.1, 128.7, 127.5, 114.0, 88.5, 87.2, 84.1, 70.3, 64.5, 63.6, 58.7, 55.5, 46.3, 35.6, 18.4, -1.4 (some carbon signals appeared too broad for an unequivocal assignment); HRMS (ESI) *m*/*z*: $[M-H]^-$ Calcd. for $C_{39}H_{50}N_3O_{10}Si$ 746.3114; Found 746.3113.

General procedure for the synthesis of phosphoramidite 26:

A solution of 5′-DMTr-protected compound **25** (1.0 equiv.) and DIPEA (4.0 equiv.) in dry DCM was cooled to 0°C. To this solution was slowly added 2-cyanoethyl *N,N*-diisopropylchlorophosphoramidite (CED-Cl) (2.5 equiv.) and the reaction mixture was stirred at r.t. for 5 h. The reaction was quenched by addition of aq. sat. $NaHCO_3$ and the crude was extracted three times with DCM. The combined organic layers were dried ($MgSO_4$), filtered and concentrated under reduced pressure. After purification by silica gel column chromatography with an addition of 0.1% pyridine and co-lyophilization from benzene the desired phosphoramidite **26** was obtained as a mixture of diastereoisomers and rotamers as a white foam.

26: Yield: 66%; R_f = 0.19 (1:1 DCM/EtOAc); ^{31}P{^{1}H} NMR (162 MHz, acetone-*d*$_6$, 298 K) δ (ppm): 150.0, 149.9, 149.8, 149.7; HRMS (ESI) *m*/*z*: $[M-H]^-$ Calcd. for $C_{48}H_{67}N_5O_{11}PSi$ 948.4338; Found 948.4333.

2.8 Nucleobase-modified 2'-methoxy N^6-carbamoyl adenosine phosphoramidite

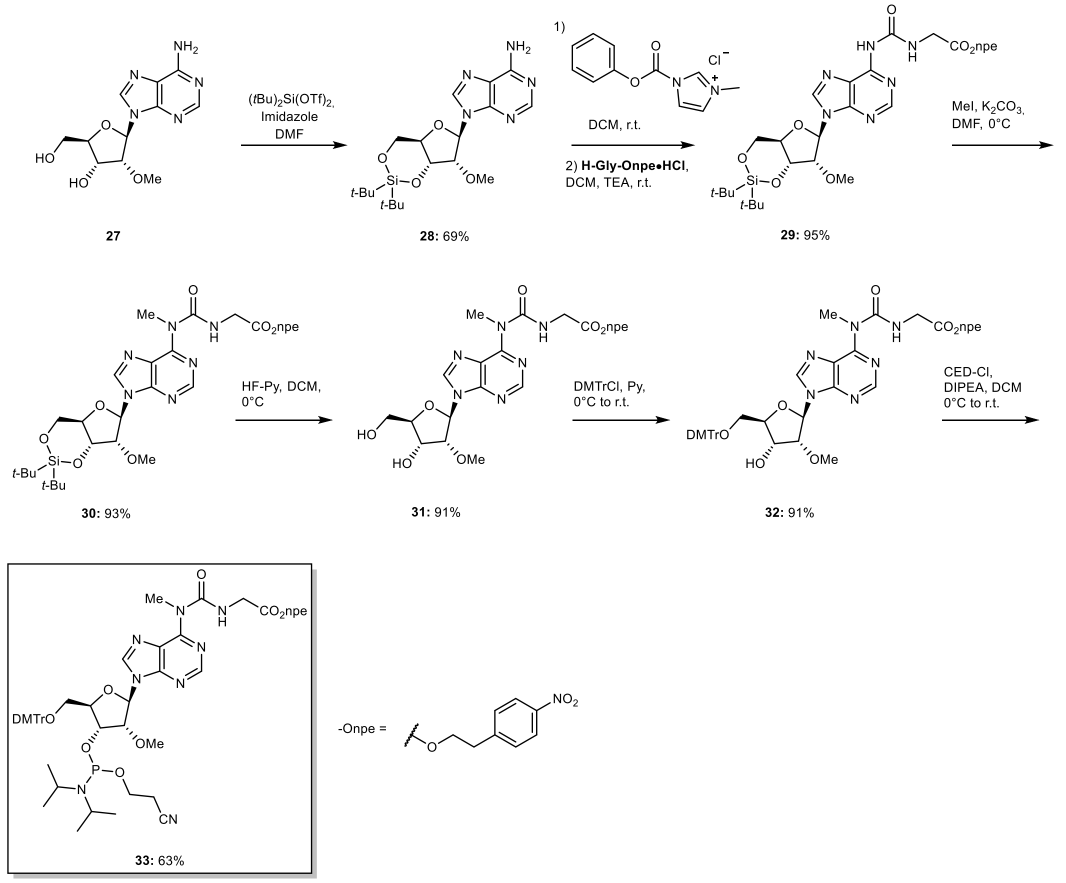

Scheme S8. Synthesis of nucleobase-modified 2'-methoxy N^6-carbamoyl adenosine phosphoramidite.

General procedure for the synthesis of 28:

A suspension of 2′-OMe adenosine **27** (1.0 equiv.) in DMF was cooled to 0°C. Di-*tert*-butylsilyl *bis*(trifluoromethanesulfonate) (1.1 equiv.) was added dropwise and the mixture was stirred at r.t. for 30 min. To the reaction was added imidazole (2.5 equiv.) and the resulting solution was stirred at r.t. for 16 h. The reaction was concentrated under reduced pressure and the residue was redissolved in EtOAc and washed with water, aq. sat. $NaHCO_3$ solution and brine. The organic layer was dried ($MgSO_4$), filtered and concentrated. The crude was purified by silica gel column chromatography to yield **28** as a white foam.

28: Yield: 69%; R_f = 0.21 (100:1 DCM/MeOH); IR (ATR) $\tilde{\nu}$ (cm^{-1}): 3319 (w), 3161 (m), 2933 (w), 1669 (s), 1600 (s), 1472 (s), 1367 (m), 1328 (m), 1260 (m), 1207 (m), 1133 (s), 1069 (s), 1027 (s), 966 (s), 907 (w), 829 (s), 739 (s), 653 (s); ^{1}H NMR (400 MHz, DMSO-d_6, 298 K) δ (ppm): 8.32 (s, 1H), 8.13 (s, 1H), 7.36 (s, 2H), 6.01 (s, 1H), 4.89 (dd, J = 9.0, 4.8 Hz, 1H), 4.34 (d, J = 4.8 Hz, 1H), 4.31 (d, J = 4.8 Hz, 1H), 4.03-3.94 (m, 2H), 3.54 (s, 3H), 1.08 (s, 9H), 1.01 (s, 9H); ^{13}C{^{1}H} NMR (100 MHz, DMSO-d_6, 298 K) δ (ppm): 156.6, 153.2, 149.2, 140.4, 119.6, 88.6, 82.1, 76.9, 74.5, 67.3, 58.8, 27.7, 27.4, 22.7, 20.4; HRMS (ESI) m/z: [M+H]$^+$ Calcd. for $C_{19}H_{32}N_5O_4Si$ 422.2218; Found 422.2220.

General procedure for the synthesis of 29:

To a solution of silyl-protected 2′-OMe adenosine **28** (1.0 equiv.) in DCM was added 1-*N*-methyl-3-phenoxycarbonyl-imidazolium chloride (2.0 equiv.). The resulting suspension was stirred at r.t. for 16 h and then **H-aa-Onpe•HCl** (2.0 equiv.) together with NEt_3 (2.0 equiv.) was added. After stirring for 16 h, the reaction mixture was quenched by the addition of aq. sat. $NaHCO_3$ and the crude was extracted three times with DCM. The combined organic layers were dried ($MgSO_4$), filtered and concentrated *in vacuo*. Purification by silica gel column chromatography furnished the amino acid-modified adenosine derivative **29** as a white foam.

29: Yield: 95%; R_f = 0.23 (100:1 DCM/MeOH); IR (ATR) $\tilde{\nu}$ (cm^{-1}): 3235 (w), 2934 (w), 2856 (m), 1747 (m), 1702 (s), 1587 (m), 1518 (s), 1467 (s), 1343 (s), 1257 (m), 1187 (s), 1138 (s), 1062 (s), 1014 (m), 825 (s), 736 (m), 651 (s); ^{1}H NMR (400 MHz, $CDCl_3$, 298 K) δ (ppm): 9.99 (t, J = 5.6 Hz, 1H), 8.77 (s, 1H), 8.51 (s, 1H), 8.26 (s, 1H),

8.08 (d, J = 8.7 Hz, 2H), 7.38 (d, J = 8.7 Hz, 2H), 6.01 (s, 1H), 4.65 (dd, J = 9.6, 4.6 Hz, 1H), 4.50-4.38 (m, 3H), 4.27 (d, J = 4.6 Hz, 1H), 4.22-4.14 (m, 3H), 4.05 (dd, J = 9.6, 9.6 Hz, 1H), 3.69 (s, 3H), 3.09 (t, J = 6.6 Hz, 2H), 1.09 (s, 9H), 1.06 (s, 9H); $^{13}C\{^1H\}$ NMR (100 MHz, $CDCl_3$, 298 K) δ (ppm): 170.0, 154.3, 151.3, 150.3, 149.9, 146.9, 145.5, 142.1, 129.9, 123.8, 121.1, 89.7, 82.4, 77.3, 74.9, 67.6, 64.7, 59.5, 42.2, 35.0, 27.5, 27.2, 22.9, 20.5; HRMS (ESI) *m/z*: $[M+H]^+$ Calcd. for $C_{30}H_{42}N_7O_9Si$ 672.2808; Found 672.2808.

General procedure for the synthesis of 30:

The amino acid-modified 2′-OMe adenosine derivative **29** (1.0 equiv.) was dissolved in DMF and cooled to 0°C. To the solution were added K_2CO_3 (3.0 equiv.) together with MeI (2.0 equiv.) and the reaction was stirred at r.t. for 2 h. The reaction mixture was diluted with H_2O and extracted three times with EtOAc. The combined organic layers were washed with water, dried ($MgSO_4$), filtered and concentrated. The obtained residue was purified by silica gel column chromatography to give **30** as a white foam.

30: Yield: 93%; R_f = 0.32 (1:1 *i*-Hexane/EtOAc); IR (ATR) $\tilde{\nu}$ (cm^{-1}): 2932 (w), 1857 (w), 1746 (m), 1682 (s), 1567 (s), 1517 (s), 1467 (s), 1343 (s), 1266 (m), 1192 (m), 1135 (s), 1062 (s), 1027 (s), 826 (s), 735 (m), 651 (s); ^1H NMR (400 MHz, $CDCl_3$, 298 K) δ (ppm): 10.95 (t, J = 5.4 Hz, 1H), 8.51 (s, 1H), 8.10 (d, J = 8.7 Hz, 2H), 7.98 (s, 1H), 7.37 (d, J = 8.7 Hz, 2H), 6.02 (s, 1H), 4.62-4.54 (m, 1H), 4.48 (dd, J = 9.2, 5.0 Hz, 1H), 4.43 (t, J = 6.6 Hz, 2H), 4.27-4.12 (m, 4H), 4.03 (d, J = 10.5 Hz, 1H), 3.98 (s, 3H), 3.69 (s, 3H), 3.08 (t, J = 6.6 Hz, 2H), 1.09 (s, 9H), 1.05 (s, 9H); $^{13}C\{^1H\}$ NMR (100 MHz, $CDCl_3$, 298 K) δ (ppm): 170.3, 156.2, 153.2, 151.7, 150.3, 147.0, 145.6, 139.6, 129.9, 123.8, 122.8, 89.7, 82.3, 77.3, 74.8, 67.6, 64.6, 59.5, 43.0, 35.0, 34.8, 27.5, 27.2, 22.9, 20.5; HRMS (ESI) *m/z*: $[M+H]^+$ Calcd. for $C_{31}H_{44}N_7O_9Si$ 686.2964; Found 686.2967.

General procedure for the synthesis of 31:

A solution of the modified 2′-OMe adenosine derivative **30** (1.0 equiv.) in DCM/pyridine (9:1 v/v) inside a plastic reaction vessel was cooled to 0°C. Subsequently, a solution of 70% HF-pyridine (5.0 equiv.) was slowly added and the reaction mixture was stirred at 0°C for 2 h. The reaction mixture was diluted with aq. sat. $NaHCO_3$ solution and extracted three times with DCM. The combined organic layers were washed with water, dried ($MgSO_4$), filtered and concentrated under reduced pressure. The crude product was purified by silica gel column chromatography to isolate the 3’,5’-deprotected adenosine derivative **31** as a white foam.

31: Yield: 91%; R_f = 0.25 (100:5 DCM/MeOH); IR (ATR) $\tilde{\nu}$ (cm^{-1}): 3201 (w), 2935 (w), 1743 (m), 1677 (m), 1568 (s), 1514 (s), 1464 (m), 1343 (s), 1268 (m), 1209 (m), 1110 (m), 1036 (m), 856 (m), 795 (s), 697 (m), 645 (m); ^1H NMR (400 MHz, $CDCl_3$, 298 K) δ (ppm): 10.85 (t, J = 5.4 Hz, 1H), 8.51 (s, 1H), 8.13 (d, J = 8.8 Hz, 2H), 8.01 (s, 1H), 7.39 (d, J = 8.8 Hz, 2H), 5.94-5.91 (m, 2H), 4.72 (dd, J = 7.4, 4.7 Hz, 1H), 4.60 (d, J = 4.7 Hz, 1H), 4.43 (t, J = 6.6 Hz, 2H), 4.37 (d, J = 1.0 Hz, 1H), 4.25-4.09 (m, 2H), 4.01 (s, 3H), 4.00-3.92 (m, 1H), 3.84-3.74 (m, 1H), 3.37 (s, 3H), 3.09 (t, J = 6.6 Hz, 2H), 2.69 (d, J = 1.7 Hz, 1H); $^{13}C\{^1H\}$ NMR (100 MHz, $CDCl_3$, 298 K) δ (ppm): 170.2, 156.0, 153.8, 151.2, 149.6, 147.0, 145.5, 141.6, 129.9, 123.9, 123.9, 89.7, 88.2, 82.3, 70.6, 64.7, 63.4, 59.0, 43.1, 35.0; HRMS (ESI) *m/z*: $[M+H]^+$ Calcd. for $C_{23}H_{28}N_7O_9$ 546.1943; Found 546.1943.

General procedure for the synthesis of 32:

The 3’,5’-deprotected 2′-OMe adenosine derivative **31** (1.0 equiv.) was dissolved in pyridine and DMTrCl (1.5 equiv.) was added. The reaction mixture was stirred at r.t. for 16 h and afterwards the solvents were removed *in vacuo*. Purification by silica gel column chromatography with an addition of 0.1% pyridine afforded the DMTr-protected adenosine derivative **32** as a pale-yellow foam.

32: Yield: 91%; R_f = 0.45 (100:5 DCM/MeOH); IR (ATR) $\tilde{\nu}$ (cm^{-1}): 2358 (w), 1682 (m), 1568 (m) 1509 (s), 1463 (m), 1344 (s), 1249 (m), 1174 (m), 1033 (s), 701 (w), 667 (w); ^1H NMR (400 MHz, $CDCl_3$, 298 K) δ (ppm): 10.84 (t, J = 5.4 Hz, 1H), 8.48-8.42 (m, 2H), 8.10 (d, J = 8.6 Hz, 2H), 7.57 (d, J = 8.6 Hz, 2H), 7.48 (d, J = 7.4 Hz, 2H), 7.39-7.32 (m, 4H), 7.28 (t, J = 7.4 Hz, 2H), 7.24-7.20 (m, 1H), 6.91-6.78 (m, 4H), 6.27 (d, J = 4.0 Hz, 1H), 4.74-4.64 (m, 1H), 4.59 (t, J = 4.5 Hz, 1H), 4.43 (t, J = 6.4 Hz, 2H), 4.28-4.21 (m, 2H), 4.11 (d, J = 5.6 Hz, 2H), 3.92 (s, 3H), 3.77 (s, 6H), 3.53 (s, 3H), 3.45 (d, J = 4.6 Hz, 2H), 3.13 (t, J = 6.4 Hz, 2H); $^{13}C\{^1H\}$ NMR (100 MHz, $CDCl_3$, 298 K) δ (ppm): 170.7, 159.6, 156.5, 153.7, 153.0, 150.7, 147.6, 147.4, 146.0, 141.7, 136.7, 131.0, 130.9, 129.0, 128.6, 127.6, 124.1, 123.2, 113.8, 87.6, 87.1, 84.9, 83.8, 70.6, 65.0, 64.3, 58.8, 55.5, 43.4, 35.3, 34.8; HRMS (ESI) *m/z*: $[M+H]^+$ Calcd. for $C_{44}H_{46}N_7O_{11}$ 848.3249; Found 848.3234.

General procedure for the synthesis of 33:

To a solution of 5’-DMTr-protected 2′-OMe adenosine derivative **32** (1.0 equiv.) in anhydrous DCM, *N*,*N*-diisopropylethylamine (DIPEA) (4.0 equiv.) was added. After cooling down to 0°C, 2-cyanoethyl *N*,*N*-diisopropylchlorophosphoramidite (CED-Cl) (2.5 equiv.) was added dropwise and the reaction mixture was stirred

at r.t. for 5 h. After that, aq. sat. $NaHCO_3$ solution was added to the reaction mixture and the aqueous phase was extracted three times with DCM. The combined organic layers were dried ($MgSO_4$), filtered and concentrated *in vacuo*. The crude product was purified by silica gel column chromatography with addition of 0.1% pyridine and co-lyophilized from benzene to afford the desired phosphoramidite **33** as a mixture of diastereoisomers and as a white foam.

33: Yield: 63%; R_f = 0.25 (1:1 *i*-Hexane/EtOAc); $^{31}P\{^1H\}$ NMR (162 MHz, acetone-d_6, 298 K) δ (ppm): 150.2, 149.7; HRMS (ESI) *m/z*: $[M+H]^+$ Calcd. for $C_{53}H_{63}N_9O_{12}P$ 1048.4328; Found 1048.4309.

3. General information and instruments for oligonucleotides

3.1 Synthesis and purification of oligonucleotides

Phosphoramidites of canonical ribonucleosides (Bz-A-CE, Dmf-G-CE, Ac-C-CE and U-CE) were purchased from LinkTech and Sigma-Aldrich. Oligonucleotides (ONs) were synthesized on a 1 µmol scale using RNA SynBase™ CPG 1000/110 and High Load Glen UnySupport™ as solid supports for strands containing amino acid-modified carbamoyl adenosine and 5-(methyl)aminomethyl uridine derivatives, respectively, using an RNA automated synthesizer (Applied Biosystems 394 DNA/RNA Synthesizer) with a standard phosphoramidite chemistry. ONs were synthesized in DMT-OFF mode using DCA as a deblocking agent in CH_2Cl_2, BTT or Activator 42® as activator in MeCN, Ac_2O as capping reagent in pyridine/THF and I_2 as oxidizer in pyridine/H_2O.

Deprotection of npe and teoc groups

For the deprotection of the *para*-nitrophenylethyl (npe) group in ONs containing amino acid-modified carbamoyl adenosine derivatives, the solid support beads were suspended in a 9:1 THF/DBU solution mixture (1 mL) and incubated at r.t. for 2 h.[9] After that, the supernatant was removed and the beads were washed with THF (3×1 mL).

For the deprotection of the 2-(trimethylsilyl)ethoxycarbonyl (teoc) group in ONs containing 5-(methyl)aminomethyl uridine derivatives, the solid support beads were suspended in a saturated solution of $ZnBr_2$ in 1:1 $MeNO_2$/IPA (1 mL) and incubated at r.t. overnight.[10] After that, the supernatant was removed and the beads were washed with 0.1 M EDTA in water (1 mL) and water (1 mL).

Cleavage from beads, deprotection of TBS groups and precipitation of the synthesized ON

The solid support beads were suspended in a 1:1 aqueous solution mixture (0.6 mL) of 30% NH_4OH and 40% $MeNH_2$. The suspension was heated at 65ºC (8 min for SynBase™ CPG 1000/110 and 60 min for High Load Glen UnySupport™). Subsequently, the supernatant was collected and the beads were washed with water (2×0.3 mL). The combined aqueous solutions were concentrated under reduced pressure using a SpeedVac concentrator. After that, the crude was dissolved in DMSO (100 µL) and triethylamine trihydrofluoride (125 µL) was added. The solution was heated at 65ºC for 1.5 h. Finally, the ON was precipitated by adding 3 M NaOAc in water (25 µL) and *n*-butanol (1 mL). The mixture was kept at -80ºC for 2 h and centrifuged at 4ºC for 1 h. The supernatant was removed and the white precipitate was lyophilized.

Purification of the synthesized ON by HPLC and desalting

The crude was purified by semi-preparative HPLC (1260 Infinity II Manual Preparative LC System from Agilent equipped with a G7114A detector) using a reverse-phase (RP) VP 250/10 Nucleodur 100-5 C18ec column from Macherey-Nagel. Buffers: A) 0.1 M AcOH/Et_3N in H_2O at pH 7 and B) 0.1 M AcOH/Et_3N in 80% (v/v) MeCN in H_2O. Gradient: 0-25% of B in 45 min. Flow rate = 5 $mL\cdot min^{-1}$. The purified ON was analyzed by RP-HPLC (1260 Infinity II LC System from Agilent equipped with a G7165A detector) using an EC 250/4 Nucleodur 100-3 C18ec from Macherey-Nagel. Gradient: 0-30% or 0-40% of B in 45 min. Flow rate = 1 $mL\cdot min^{-1}$. Finally, the purified ON was desalted using a C18 RP-cartridge from Waters.

Determination of the concentration and the mass of the synthesized ON

The absorbance of the synthesized ON in H_2O solution was measured using an IMPLEN NanoPhotometer® N60/N50 at 260 nm. The extinction coefficient of the single stranded ONs was calculated using the OligoAnalyzer Version 3.0 from Integrated DNA Technologies. For ONs incorporating non-canonical bases, the extinction coefficients were assumed to be identical to those containing only canonical counterparts.

The synthesized ON (2-3 µL) was desalted on a 0.025 µm VSWP filter (Millipore), co-crystallized in a 3-hydroxypicolinic acid matrix (HPA, 1 µL) and analyzed by MALDI-TOF mass spectrometry (negative mode).

3.2 Analysis of coupling and cleavage reactions by HPLC and MALDI-TOF mass spectrometry

The crudes of the coupling and cleavage reactions were analyzed by RP-HPLC using an EC 250/4 Nucleodur 100-3 C18ec column from Macherey-Nagel. Buffers: A) 0.1 M $AcOH/Et_3N$ in H_2O at pH 7 and B) 0.1 M $AcOH/Et_3N$ in 80% (v/v) MeCN in H_2O. Gradient: 0-40% of B in 45 min. Flow rate = 1 $mL{\cdot}min^{-1}$. Injection: 20 µL (1 nmol). The same HPLC method was used for the purification of the products obtained in the coupling and cleavage reactions. The yields of the reactions were calculated by integration of the chromatographic peaks of the products and the use of the calibration curves of the corresponding canonical ONs (see Section 5). In order to simplify the calculations, we assumed that the formed products and the canonical oligonucleotides used for calibration featured identical extinction coefficients, which were calculated for single stranded RNAs. It is expected that double strands and/or secondary structures are disrupted under the HPLC conditions used.

The crudes of the reactions and the isolated products (2-3 µL) were desalted on a 0.025 µm VSWP filter (Millipore), co-crystallized in a 3-hydroxypicolinic acid matrix (HPA, 1 µL) and analyzed by MALDI-TOF mass spectrometry (negative mode).

3.3 Coupling of amino acids and peptides to ONs anchored to the solid support beads

Oligonucleotides (ONs) were synthesized on a 4 µmol scale using the High Load Glen UnySupport^{TM} for strands containing glycine-modified carbamoyl adenosine and 5-valine-methylaminomethyl uridine derivatives using an RNA automated synthesizer (Applied Biosystems 394 DNA/RNA Synthesizer) with a standard phosphoramidite chemistry. The npe and teoc protecting groups were removed as described in Section 3.1 and the solid support beads were dried using a SpeedVac concentrator.

The solid support beads (1 µmol) in an Eppendorf tube were washed with dry DMF (0.3 mL). In a separate Eppendorf tube, Boc-protected amino acid (for altering of the mnm^5U derivatives), npe-protected amino acid (for altering of the m^6g^6A derivatives) or protected peptide (100 µmol), $DMTMM{\bullet}BF_4$ (100 µmol) as activator and dry DIPEA (200 µmol) were dissolved in dry DMF (0.6 mL). Subsequently, the amino acid or peptide solution was added to the solid support beads and the reaction was incubated in an orbital shaker at r.t. for 1 h. The suspension was centrifuged and the supernatant was removed. The solid support beads were washed with dry DMF (2×0.3 mL) and dry MeCN (2×0.3 mL). Finally, the beads were dried using a SpeedVac concentrator.

For the deprotection of the *tert*-butyloxycarbonyl (Boc) group in ONs after the coupling of a Boc-protected amino acid or peptide, the solid support beads were suspended in a 1:1 TFA/CH_2Cl_2 solution mixture (0.5 mL) and incubated for 5 min at r.t.[11] After that, the supernatant was removed and the solid support beads were washed with CH_2Cl_2 (2×0.5 mL). The deprotection of the npe-protected adenosine derivatives was performed as described in Section 3.1.

The ONs containing 5-peptide-methylaminomethyl uridine derivatives were cleaved from the solid support beads using a 1:1 aqueous solution mixture (0.6 mL) of 30% NH_4OH and 40% $MeNH_2$ at 65ºC for 60 min. The ONs containing peptide-modified carbamoyl adenosine derivatives were cleaved from the solid support beads using a 30% NH_4OH aqueous solution (0.6 mL) at r.t. overnight. The following work-up and purification steps were identical to those described in Section 3.1. Based on HPLC analyses, we calculated that the coupling reaction using the solid support beads and $DMTMM{\bullet}BF_4$ as activator proceeded in an extent larger than 70%.

4. Synthesized oligonucleotides using a DNA/RNA automated synthesizer

4.1 Canonical oligonucleotides (CON)

RNA sequences:

CON1; 5’-AAU CGC U-3’

CON2; 5’-GUA CAG CGA UU-3’

CON3; 5’-GUA CAG CGA UUA AUC GCU-3’

CON4; 5’-AmAmUm CmGmCm Um-3’

CON5; 5’-GmUmCm AmGmUm AmCmAm GmCmGm AmUmUm-3’

CON6; 5’-GmUmCm AmGmUm AmCmAm GmCmGm AmUmUm AmAmUm CmGmCm Um-3’

Table S1. HPLC retention times (0-30% of B in 45 min) and MALDI-TOF mass spectrometric analysis (negative mode) of canonical oligonucleotides.

Strand	t_R (min)	*m/z* calcd. for [*M*-H]$^-$	found
**CON1**	23.6	2162.3	2162.0
**CON2**	23.1	3487.5	3486.9
**CON3**	23.9	5712.8	5711.7

Table S2. HPLC retention times (0-40% of B in 45 min) and MALDI-TOF mass spectrometric analysis (negative mode) of canonical oligonucleotides.

Strand	t_R (min)	*m/z* calcd. for [*M*-H]$^-$	found
**CON4**	23.3	2261.6	2260.1
**CON5**	18.8	4772.7	4772.8
**CON6**	18.6	6998.0	6995.1

The sequences of **CON1**-**6** are similar to those of the modified ONs used in the coupling reactions. These canonical ONs were used for the development of HPLC calibration curves in Section 5.

4.2 Donor oligonucleotides (ON1) with a complementary sequence

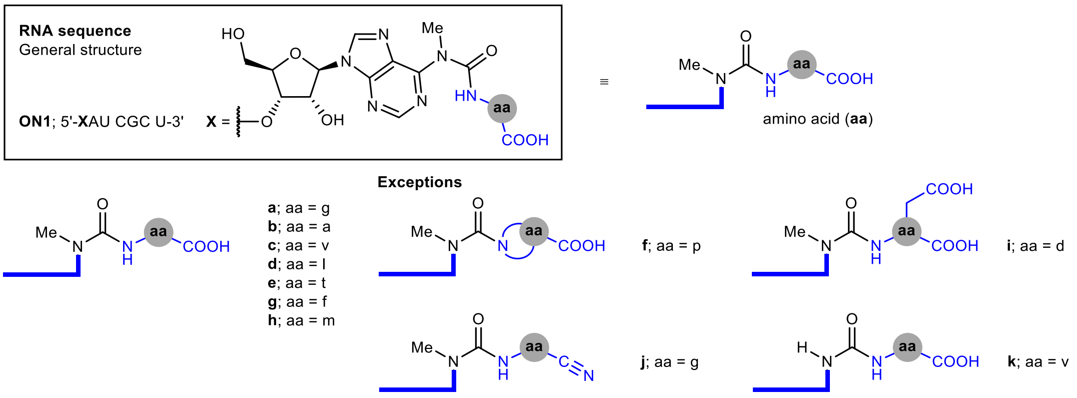

Figure S2. RNA sequence and general structure of amino acid-modified carbamoyl adenosine derivatives.

Other RNA donor strands with longer sequences:

ON1l: 5’-**X**CU AUU GAG U-3’; **X** = m$^{6}\underline{\text{v}}^{6}$A

ON1m: 5’-**X^1**AU CGC UGU ACC CUA UUG AGU **X^2**-3’; **X^1** = m$^{6}\underline{\text{v}}^{6}$A; **X^2** = m$^{6}\underline{\text{g}}^{6}$A

ON1n: 5’-**X**AU CGC UGU AC-3’; **X** = m$^{6}\underline{\text{v}}^{6}$A

ON1o: 5’-**X**AmUm CmGmCm Um-3’; **X** = m$^{6}\underline{\text{g}}^{6}$Am

ON1p: 5’-**X**AmUm CmGm-3’; **X** = m$^{6}\underline{\text{g}}^{6}$Am

ON1q: 5'-**X**AmUm-3'; **X** = m^{6}g^{6}Am

Table S3. HPLC retention times (0-40% of B in 45 min) and MALDI-TOF mass spectrometric analysis (negative mode) of **ON1**.

Strand	t_R (min)	*m/z* calcd. for [*M*-H]$^-$	found
**ON1a**; **X** = m^{6}g^{6}A	18.8	2277.4	2278.4
**ON1b**; **X** = m^{6}a^{6}A	20.2	2291.4	2290.0
**ON1c**; **X** = m^{6}v^{6}A	22.2	2319.4	2317.8
**ON1d**; **X** = m^{6}l^{6}A	24.3	2333.4	2331.6
**ON1e**; **X** = m^{6}t^{6}A	18.9	2321.4	2320.0
**ON1f**; **X** = m^{6}p^{6}A	18.0	2317.4	2316.8
**ON1g**; **X** = m^{6}f^{6}A	24.5	2368.6	2365.4
**ON1h**; **X** = m^{6}m^{6}A	23.2	2351.4	2350.4
**ON1i**; **X** = m^{6}d^{6}A	17.2	2335.4	2334.3
**ON1j**; **X** = m^{6}g^{6}A (amino nitrile)	21.2	2258.4	2258.5
**ON1k**; **X** = v^{6}A (non-methylated)	20.6	2305.4	2302.2
**ON1l**; **X** = m^{6}v^{6}A	22.3	3300.5	3301.1
**ON1m**; **X¹** = m^{6}v^{6}A and **X²** = m^{6}g^{6}A	23.1	7231.0	7233.7
**ON1n**; **X** = m^{6}v^{6}A	23.2	3604.6	3603.4
**ON1o**; **X** = m^{6}g^{6}Am	23.8	2375.5	2374.4
**ON1p**; **X** = m^{6}g^{6}Am	23.6	1736.4	1735.1
**ON1q**; **X** = m^{6}g^{6}Am	23.1	1058.2	1058.2

4.3 Acceptor oligonucleotides (ON2) with a complementary sequence

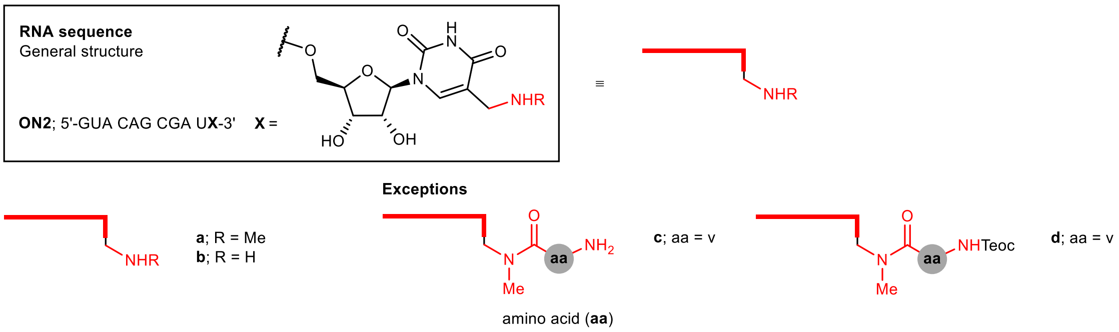

Figure S3. RNA sequence and general structure of (methyl)aminomethyl uridine derivatives.

Other RNA acceptor strands with longer sequences:

ON2e 5'-GUA CAG CGA U**X¹**A CUC AAU AG**X²**-3'; **X¹** = gmnm^{5}U; **X²** = nm^{5}U

ON2f: 5'-GUA CAG CGA U**X**A CUC AAU AGG-3'; **X** = vmnm^{5}U

ON2g: 5'-GmUmAm CmAmGm CmGmAm Um**X**-3'; **X** = mnm^{5}U

ON2h: 5'-GmUmCm AmGmUm AmCmAm GmCmGm AmUm**X**-3'; **X** = mnm^{5}Um

Table S4. HPLC retention times (0-40% of B in 45 min) and MALDI-TOF mass spectrometric analysis (negative mode) of **ON2**.

Strand	t_R (min)	*m/z* calcd. for [*M*-H]$^-$	found
**ON2a**; **X** = mnm^{5}U	17.4	3530.5	3529.7
**ON2b**; **X** = nm^{5}U	17.8	3516.5	3515.9
**ON2c**; **X** = vmnm^{5}U	18.6	3629.6	3627.2
**ON2d**; **X** = Teoc-vmnm^{5}U	37.7	3773.7	3776.9
**ON2e**; **X¹** = gmnm^{5}U and **X²** = nm^{5}U	18.7	6806.0	6806.4
**ON2f**; **X** = vmnm^{5}U	19.9	6858.0	6857.7
**ON2g**; **X** = mnm^{5}U	23.0	3670.5	3670.4
**ON2h**; **X** = mnm^{5}Um	24.2	5025.9	5026.0

4.4 Donor oligonucleotides with non-complementary sequences

RNA sequences that are not fully complementary to the acceptor **ON2**:

ON1r: 5'-**X**AU **A**GC U-3'; **X** = m^{6}g^{6}A (one mismatch marked in red)

ON1s: 5'-**X**A**G** C**C**C U-3'; **X** = m^{6}g^{6}A (two mismatches marked in red)

Table S5. HPLC retention times (0-40% of B in 45 min) and MALDI-TOF mass spectrometric analysis (negative mode) of **ON1r** and **ON1s**.

Strand	t_R (min)	*m/z* calcd. for [*M*-H]$^-$	found
**ON1r**; **X** = m^{6}g^{6}A	20.0	2301.4	2301.2
**ON1s**; **X** = m^{6}g^{6}A	19.5	2276.4	2275.7

5. HPLC calibration curves using canonical oligonucleotides (CON1-6) and hairpin-type intermediate (ON3a)

Canonical oligonucleotides, **CON1**-**6**, and hairpin-type intermediate, **ON3a**, were used for the development of HPLC calibration curves. Separate stock solutions of **CON1-6** and **ON3a** were prepared in water (100 μM). Separate standard solutions containing 1.2; 1.0; 0.8; 0.6; 0.4; 0.2 and 0.1 nmol of **CON1**-**6** and **ON3a** were prepared in a final volume of 20 µL. The standard solutions were injected in an analytical HPLC equipped with a C18 column and using buffers A and B (gradient: 0-30% or 0-40% of B in 45 min; flow rate = 1 mL·min^{-1}). The absorbance was monitored at 260 nm and the areas of the chromatographic peaks were determined by integration of the HPL-chromatograms. The plot of the chromatographic area (a.u.) versus the amount (nmol) of each oligonucleotide followed a linear relationship.

Calibration curve of **CON1**

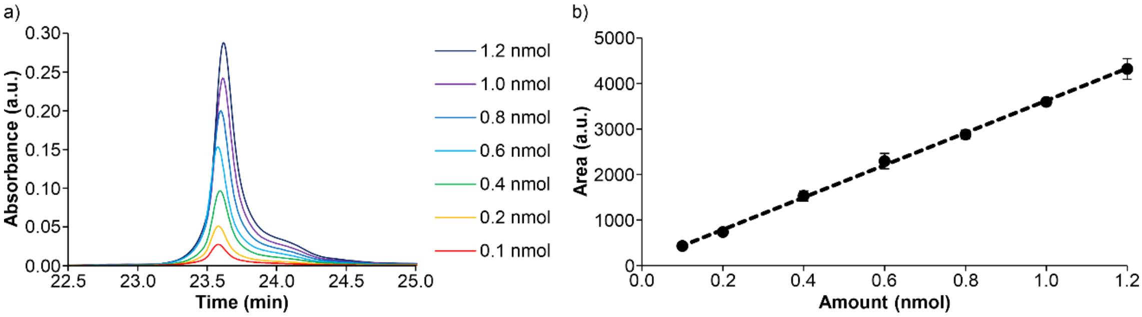

Figure S4. a) Selected region of the HPL-chromatograms upon the injection of incremental amounts (nmol) and b) chromatographic area (a.u.) *vs.* amount (nmol) of **CON1**. In b) the line shows the fit of the data to a linear regression equation. Error bars are standard deviations from three independent experiments.

Calibration curve of **CON2**

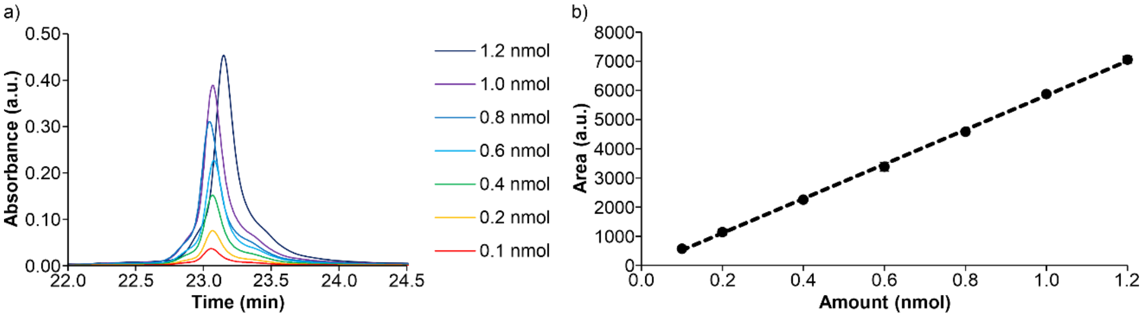

Figure S5. a) Selected region of the HPL-chromatograms upon the injection of incremental amounts (nmol) and b) chromatographic area (a.u.) *vs.* amount (nmol) of **CON2**. In b) the line shows the fit of the data to a linear regression equation. Error bars are standard deviations from three independent experiments.

Calibration curve of **CON3**

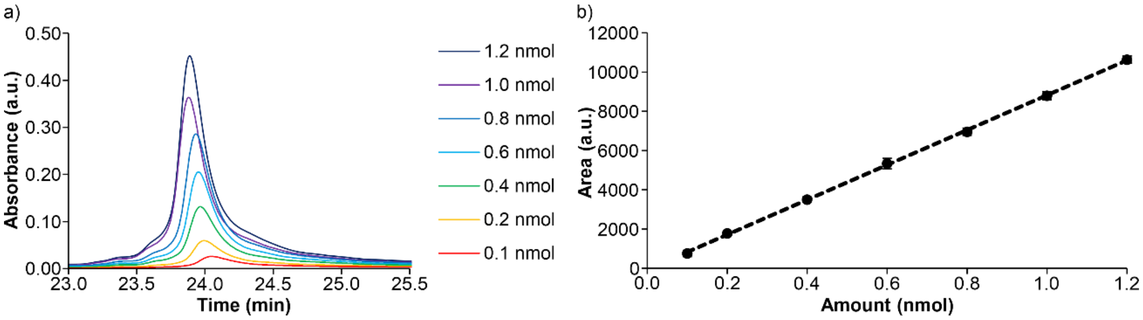

Figure S6. a) Selected region of the HPL-chromatograms upon the injection of incremental amounts (nmol) and b) chromatographic area (a.u.) *vs.* amount (nmol) of **CON3**. In b) the line shows the fit of the data to a linear regression equation. Error bars are standard deviations from three independent experiments.

Calibration curve of **ON3a**

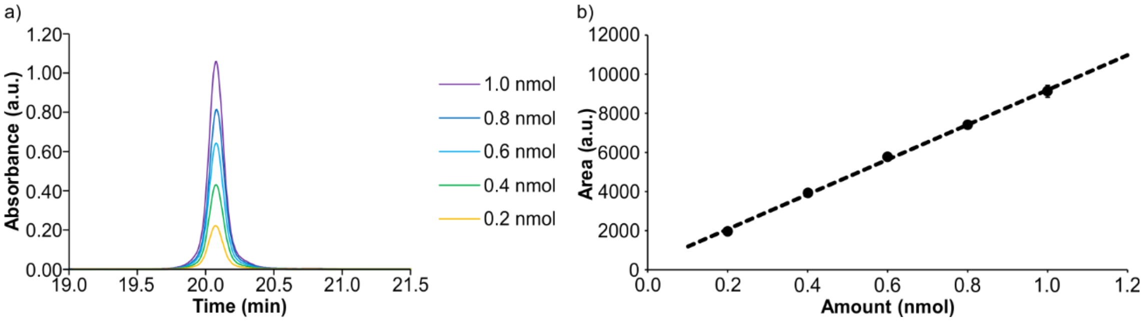

Figure S7. a) Selected region of the HPL-chromatograms upon the injection of incremental amounts (nmol) and b) chromatographic area (a.u.) *vs.* amount (nmol) of **ON3a**. In b) the line shows the fit of the data to a linear regression equation. Error bars are standard deviations from three independent experiments.

The results of the calibration curves of **CON3** (canonical oligonucleotide) and **ON3a** (hairpin-type intermediate) were very similar (Table S6).

Calibration curve of **CON4**

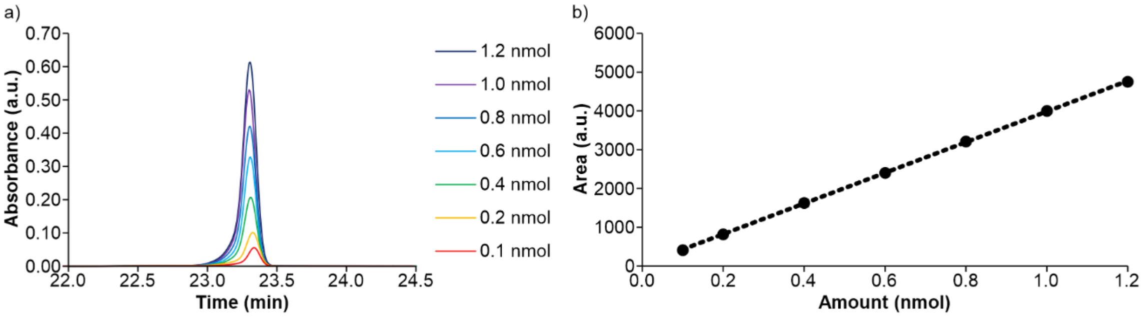

Figure S8. a) Selected region of the HPL-chromatograms upon the injection of incremental amounts (nmol) and b) chromatographic area (a.u.) *vs.* amount (nmol) of **CON4**. In b) the line shows the fit of the data to a linear regression equation. Error bars are standard deviations from three independent experiments.

Calibration curve of **CON5**

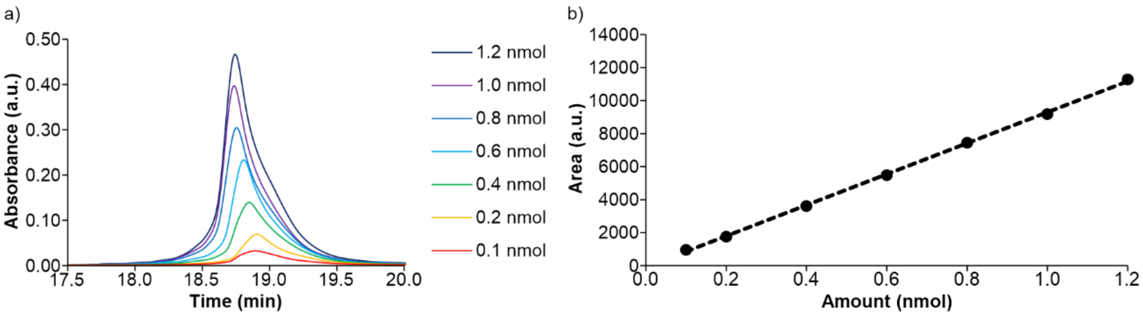

Figure S9. a) Selected region of the HPL-chromatograms upon the injection of incremental amounts (nmol) and b) chromatographic area (a.u.) *vs.* amount (nmol) of **CON5**. In b) the line shows the fit of the data to a linear regression equation. Error bars are standard deviations from three independent experiments.

Calibration curve of **CON6**

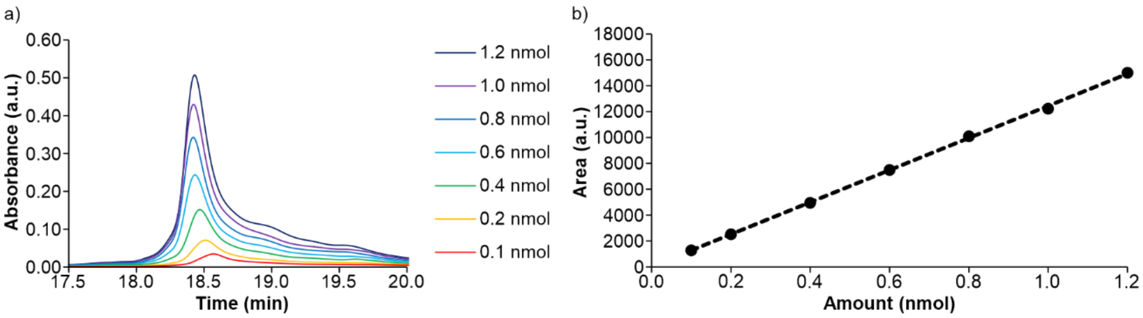

Figure S10. a) Selected region of the HPL-chromatograms upon the injection of incremental amounts (nmol) and b) chromatographic area (a.u.) *vs.* amount (nmol) of **CON6**. In b) the line shows the fit of the data to a linear regression equation. Error bars are standard deviations from three independent experiments.

Table S6. Calibration curves (y = mx + n) obtained by HPLC analyses of **CON1**-**6** and **ON3a** and calculated extinction coefficients of **CON1-6** using the OligoAnalyzer Version 3.0 from Integrated DNA Technologies.

Strand	Slope, m ($nmol^{-1}$)	Intercept, n	r^2	ε ($M^{-1}·cm^{-1}$)
**CON1**	3534.2	82.3	0.9989	65500
**CON2**	5903.7	-73.3	0.9994	107200
**CON3**	8885.4	-64.6	0.9997	170700
**ON3a**	8890.5	299.2	0.9986	170700[a]
**CON4**	3952.4	32.36	0.9999	68800
**CON5**	9376.2	-83.62	0.9995	153800
**CON6**	12405.0	41.49	0.9996	221900

[a] In order to simplify the calculations, the extinction coefficient of **ON3a** was assumed to be identical to that of **CON3**.

6. Coupling reactions between donor and acceptor oligonucleotides, ON1 and ON2

Stock solutions of pH buffer (400 mM), NaCl (1 M) and activator (500 mM, Figure S11) were prepared in water. Subsequently, equimolar amounts of **ON1** and **ON2** (3-5 nmol) were annealed at 95ºC for 4 min in water containing NaCl (half of the volume required for the reaction). Finally, buffer, NaCl, activator solutions and water were added to the ONs' solution and the reaction was incubated in a ThermoMixer at 25°C for 24 h.

Concentration of the components in the reaction mixture: 50 µM of **ON1**, 50 µM of **ON2**, 100 mM of buffer, 100 mM of NaCl and 50 mM of activator (see figure footnotes for details).

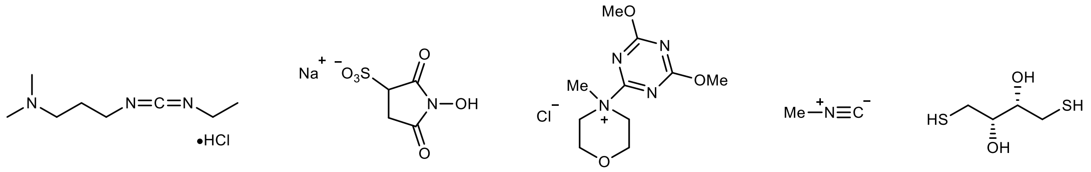

EDC **Sulfo-NHS** **DMTMM•Cl** **MeNC** **DTT**

Figure S11. Activators of carboxylic acid and nitrile groups.

The crudes of the reactions (20 µL, 1 nmol) were analyzed as indicated in Section 3.2.

6.1 Control experiments

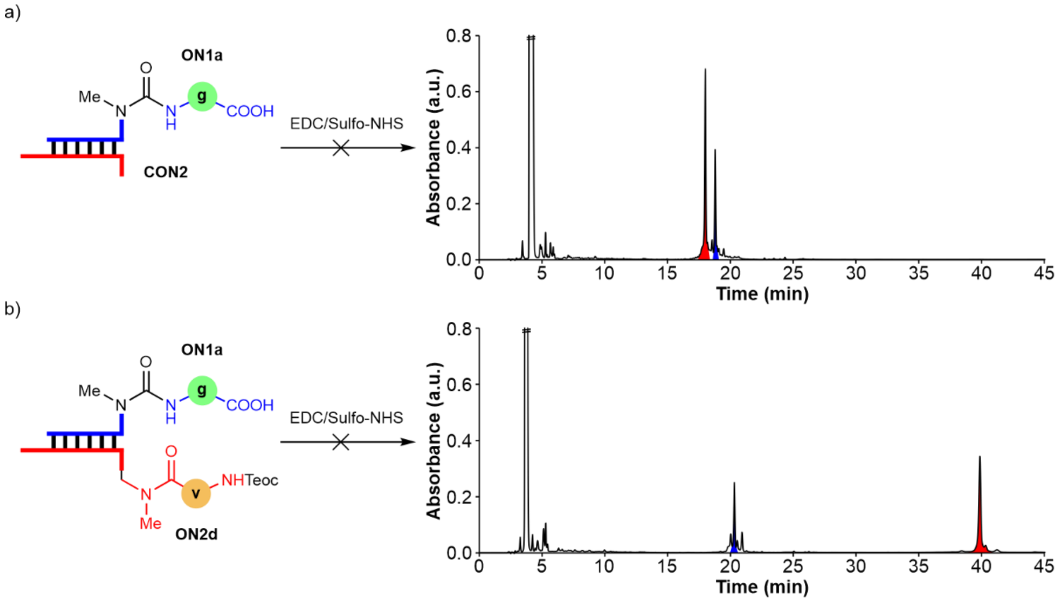

Figure S12. HPLC-chromatograms of the reactions of **ON1a**; **X** = m^{6}g^{6}A with: a) **CON2** (complementary canonical ON) and b) **ON2d**; **X** = Teoc-vmnm^{5}U in MES buffer at pH 6 using EDC/Sulfo-NHS as activator.

Control reactions using the donor strand **1a** and the RNA strand lacking the mnm group on the 3'-terminal uridine base **CON2** or the protected 3'-vmnm^{5}U-RNA-5' acceptor strand **ON2d** did not provide noticeable evidence for the formation of the corresponding hairpin-type intermediate products.

6.2 Screening of activators using ON1a (m^{6}g^{6}A) and ON2a (mnm^{5}U)

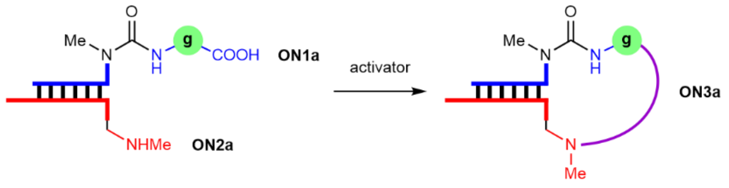

Scheme S9. Coupling of **ON1a**; **X** = m^{6}g^{6}A with **ON2a**. The formed peptide bond is marked in purple.

MES buffer at pH 6 (adjusted with NaOH)

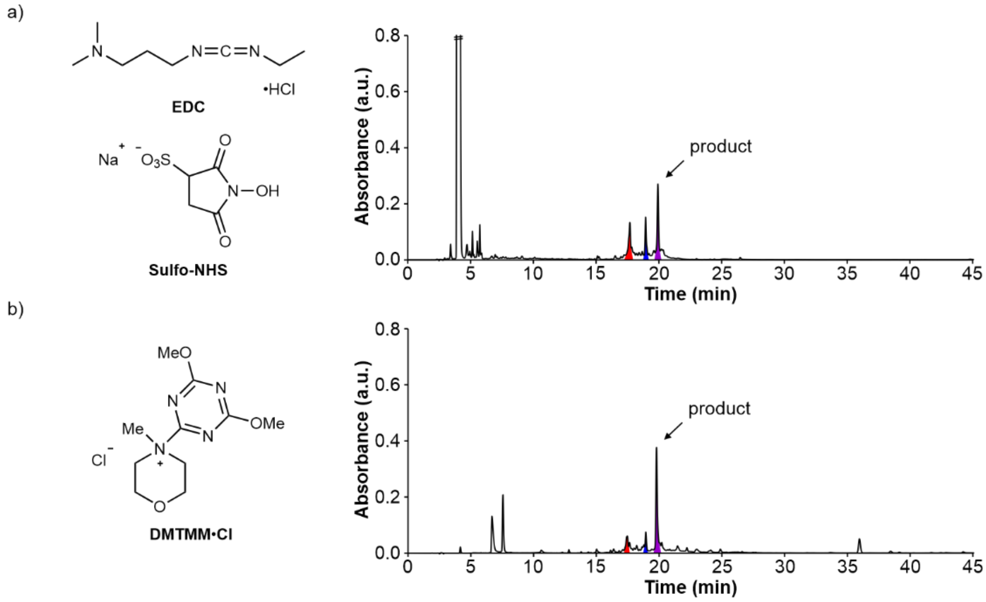

Figure S13. HPL-chromatograms of the reactions of **ON1a**; **X** = m^{6}g^{6}A with **ON2a** using: a) EDC/Sulfo-NHS and b) DMTMM•Cl as activators.

Table S7. Results obtained in the coupling reactions of **ON1a**; **X** = m^{6}g^{6}A with **ON2a** (average of, at least, two experiments).

Activators	pH	Time (h)	Average Yield ± Error (%)[a]
EDC/Sulfo-NHS	6	24	16±4
DMTMM•Cl	6	24	33±2

[a] Calculated yield from the chromatographic peak of the product using the calibration curve of **CON3**.

MOPS buffer at pH 7 (adjusted with NaOH)

Table S8. Results obtained in the coupling reactions of **ON1a**; **X** = m^{6}g^{6}A with **ON2a** (average of, at least, two experiments).

Activators	pH	Time (h)	Average Yield ± Error (%)[a]
EDC/Sulfo-NHS	7	24	20±2

[a] Calculated yield from the chromatographic peak of the product using the calibration curve of **CON3**.

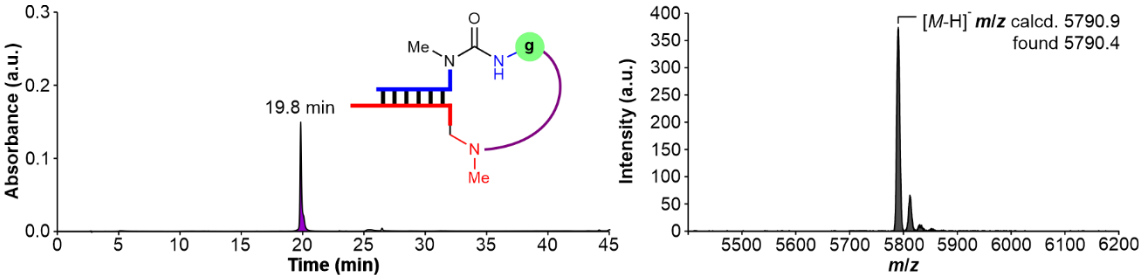

Figure S14. Left) HPL-chromatogram and right) MALDI-TOF mass spectrum (negative mode) of the isolated product **ON3a**.

6.3 Screening of activators using ON1a (m^{6}g^{6}A) and ON2b (nm^{5}U)

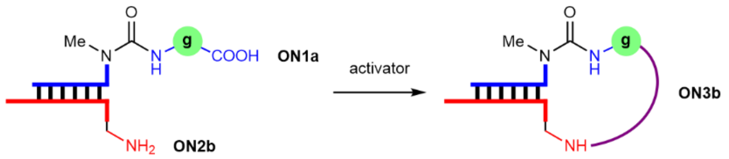

Scheme S10. Coupling of **ON1a**; **X** = m^{6}g^{6}A with **ON2b**. The formed peptide bond is marked in purple.

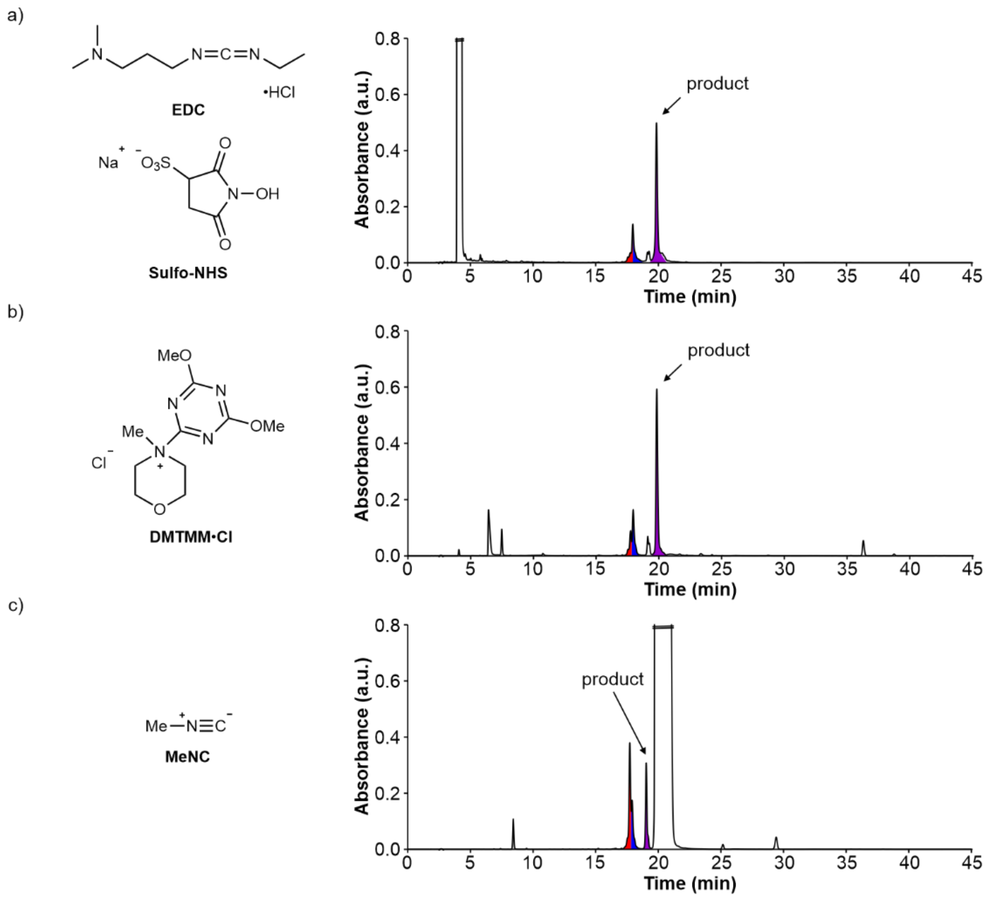

Figure S15. HPL-chromatograms of the reactions of **ON1a**; **X** = m^{6}g^{6}A with **ON2b** using: a) EDC/Sulfo-NHS; b) DMTMM•Cl and c) MeNC as activators. MES buffer (100 mM) at pH 6 in a) and b). DCI buffer (50 mM) at pH 6 in c).

Table S9. Results obtained in the coupling reactions of **ON1a**; **X** = m^{6}g^{6}A with **ON2b** (average of, at least, two experiments).

Activators	pH	Time (h)	Average Yield ± Error (%)[a]
EDC/Sulfo-NHS	6	24	64±2
DMTMM•Cl	6	24	66±2
MeNC	6	120	28±4

[a] Calculated yield from the chromatographic peak of the product using the calibration curve of **CON3**.

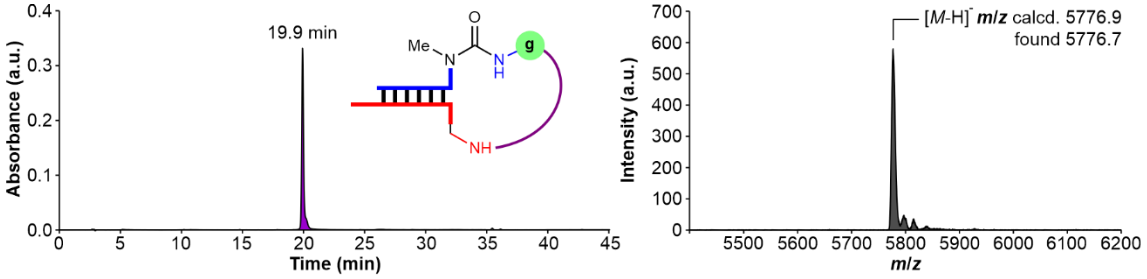

Figure S16. left) HPL-chromatogram and right) MALDI-TOF mass spectrum (negative mode) of the isolated product **ON3b**.

6.4 Screening of activators using ON1a (m^{6}g^{6}A) and ON2c (vmnm^{5}U)

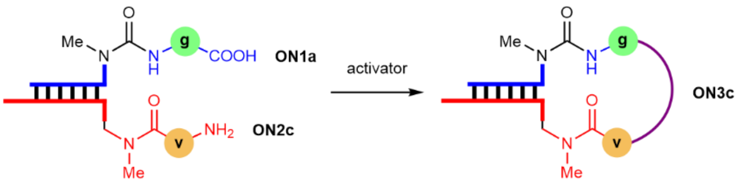

Scheme S11. Coupling of **ON1a**; **X** = m^{6}g^{6}A with **ON2c**. The formed peptide bond is marked in purple.

Buffer at pH 6

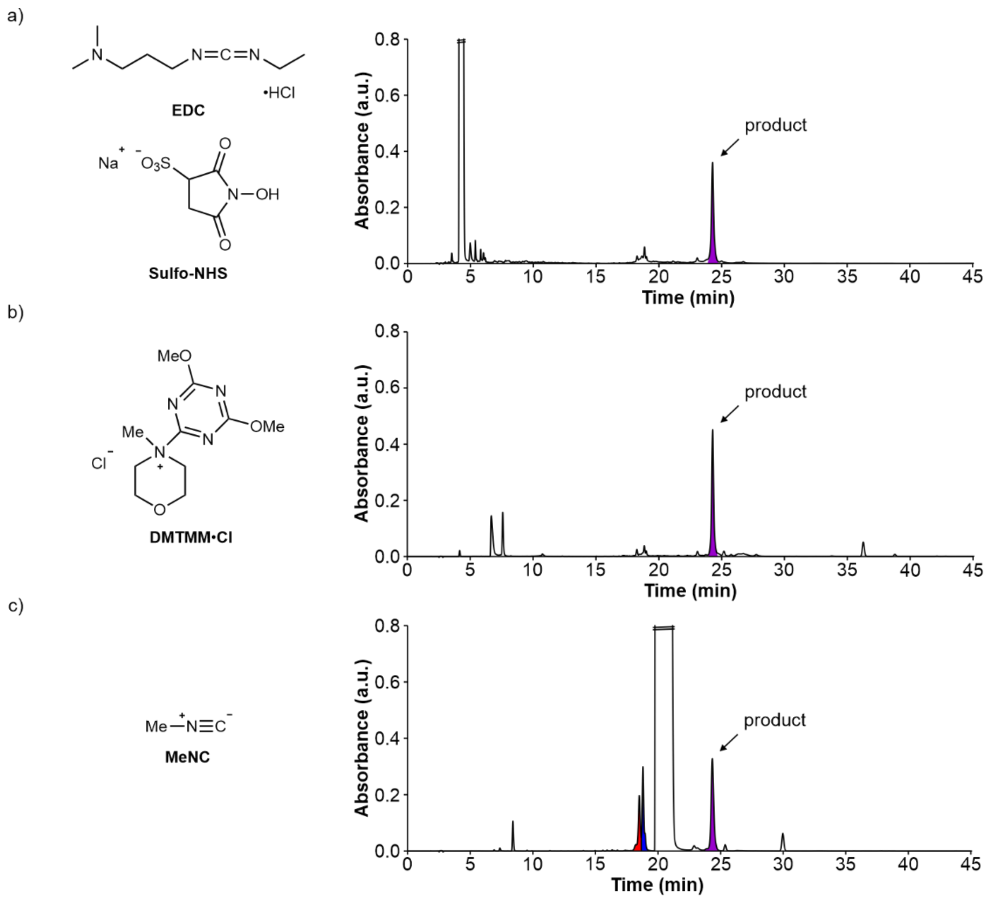

Figure S17. HPL-chromatograms of the reactions of **ON1a**; **X** = m^{6}g^{6}A with **ON2c** using: a) EDC/Sulfo-NHS; b) DMTMM•Cl and c) MeNC as activators. MES buffer (100 mM) at pH 6 in a) and b). DCl buffer (50 mM) at pH 6 in c).

Table S10. Results obtained in the coupling reactions of **ON1a**; **X** = m^{6}g^{6}A with **ON2c** (average of, at least, two experiments).

Activators	pH	Time (h)	Average Yield ± Error (%)[a]
EDC/Sulfo-NHS	6	24	56±1
DMTMM•Cl	6	24	60±2
MeNC	6	120	50±5

[a] Calculated yield from the chromatographic peak of the product using the calibration curve of **CON3**.

MOPS buffer at pH 7 (adjusted with NaOH)

Table S11. Results obtained in the coupling reactions of **ON1a**; **X** = m^{6}g^{6}A with **ON2c** (average of, at least, two experiments).

Activators	pH	Time (h)	Average Yield ± Error (%)[a]
EDC/Sulfo-NHS	7	24	50±5
DMTMM•Cl	7	24	23±1

[a] Calculated yield from the chromatographic peak of the product using the calibration curve of **CON3**.

MOPS buffer at pH 8 (adjusted with NaOH)

Table S12. Results obtained in the coupling reactions of **ON1a**; **X** = m^{6}g^{6}A with **ON2c** (average of, at least, two experiments).

Activators	pH	Time (h)	Average Yield ± Error (%)[a]
EDC/Sulfo-NHS	8	24	34±1
DMTMM•Cl	8	24	5±2

[a] Calculated yield from the chromatographic peak of the product using the calibration curve of **CON3**.

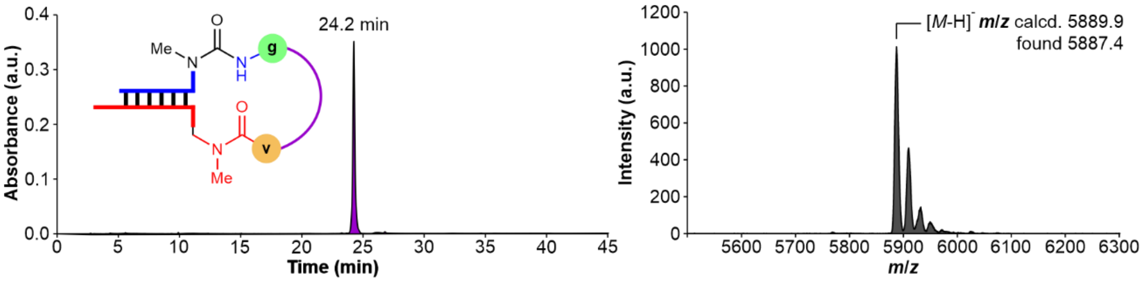

Figure S18. left) HPL-chromatogram and right) MALDI-TOF mass spectrum (negative mode) of the isolated product **ON3c**.

6.5 Coupling reactions of ON1j (m^{6}g^{6}A, amino nitrile) with ON2a-c

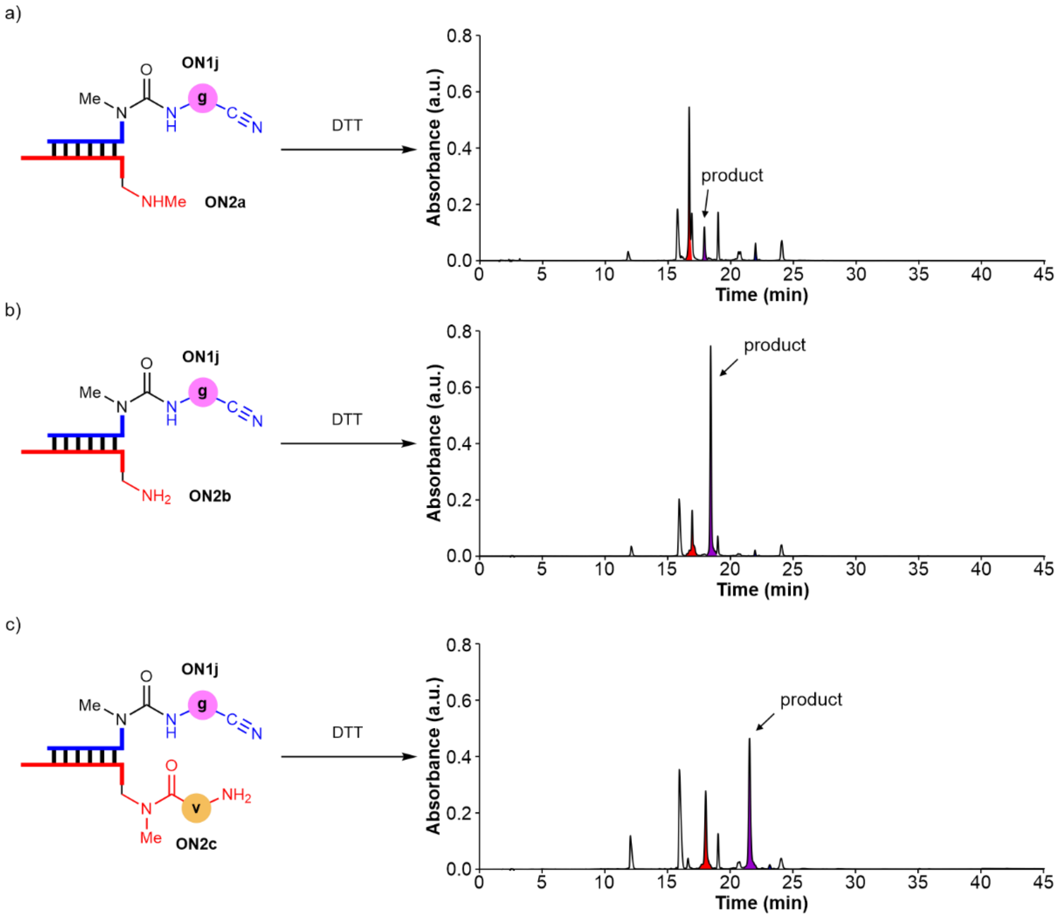

Figure S19. HPL-chromatograms of the reactions of **ON1j**; **X** = m^{6}g^{6}A (amino nitrile) with: a) **ON2a**; **X** = mnm^{5}U; b) **ON2b**; **X** = nm^{5}U and c) **ON2c**; **X** = vmnm^{5}U in boric acid buffer at pH 8 using DTT as activator.

Table S13. Results obtained in the coupling reactions of **ON1j**; **X** = m^{6}g^{6}A (amino nitrile) with **ON2a**-**c** using DTT as activator (average of, at least, two experiments).

Donor strand	Acceptor strand	Average Yield ± Error (%)[a]
**ON1j**; **X** = m^{6}g^{6}A (amino nitrile)	**ON2a**; **X** = mnm^{5}U	12±1
	**ON2b**; **X** = nm^{5}U	65±2
	**ON2c**; **X** = vmnm^{5}U	42±1

[a] Calculated yield from the chromatographic peak of the product using the calibration curve of **CON3**.

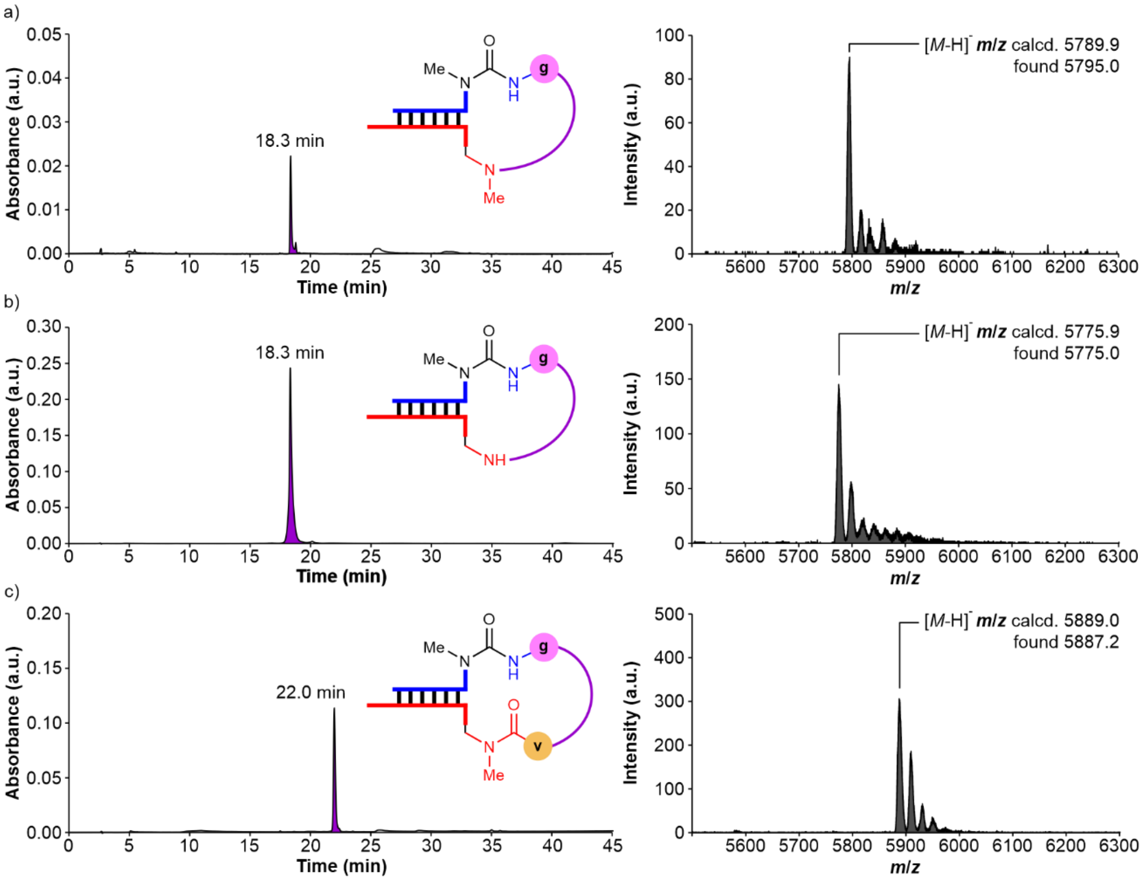

Figure S20. left) HPL-chromatograms and right) MALDI-TOF mass spectra (negative mode) of the isolated products from the reactions of **ON1j**; **X** = m^{6}g^{6}A (amino nitrile) with: a) **ON2a**; **X** = mnm^{5}U; b) **ON2b**; **X** = nm^{5}U and c) **ON2c**; **X** = vmnm^{5}U.

6.6 Coupling reactions of ON1b-i (m^{6}aa^{6}A) with ON2a

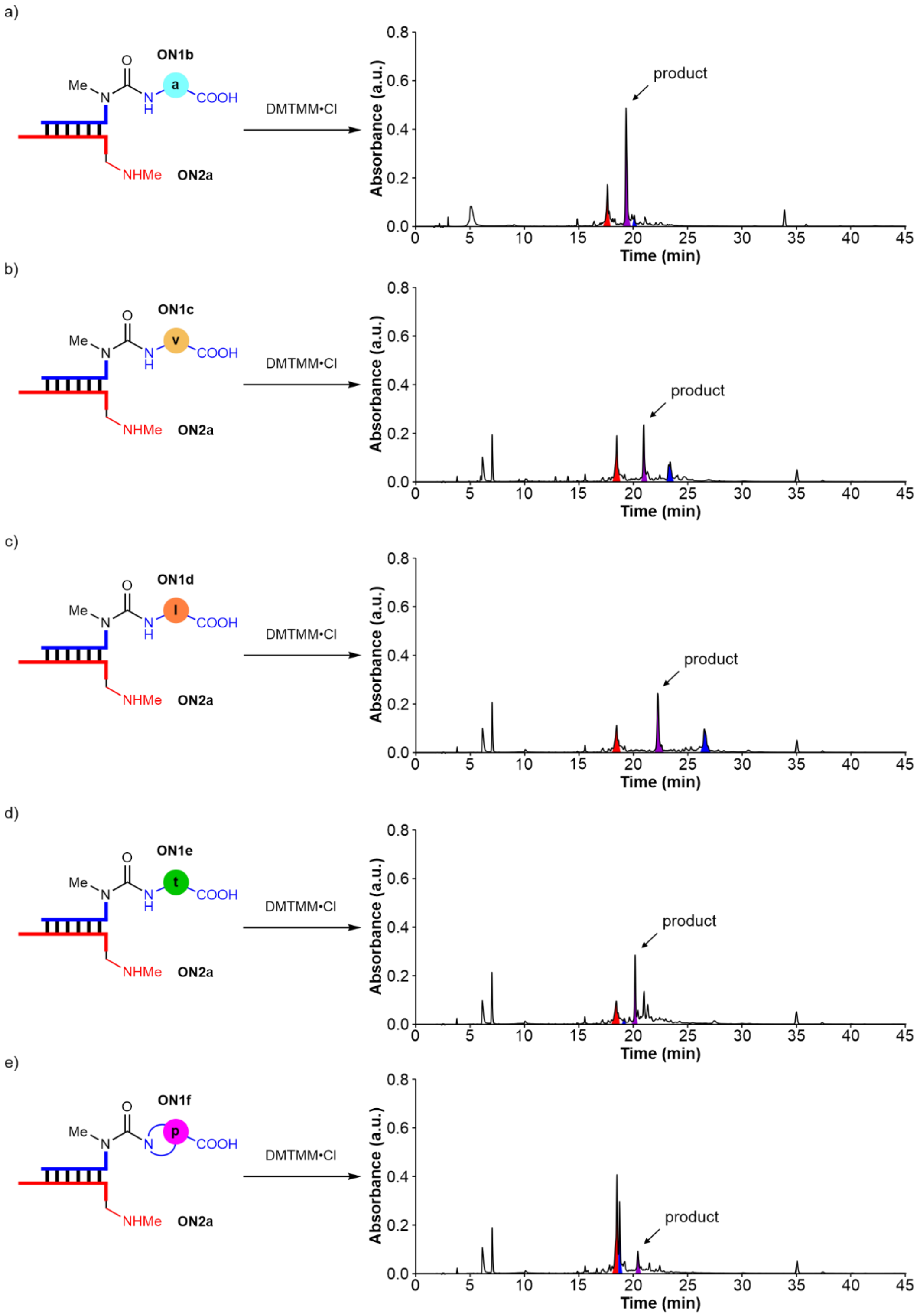

Figure S21. HPL-chromatograms of the reactions of **ON2a**; **X** = mnm^{5}U with: a) **ON1b**; **X** = m$^6\underline{a}^6$A; b) **ON1c**; **X** = m$^6\underline{v}^6$A; c) **ON1d**; **X** = m$^6\underline{l}^6$A; d) **ON1e**; **X** = m$^6\underline{t}^6$A and e) **ON1f**; **X** = m$^6\underline{p}^6$A in MES buffer at pH 6 using DMTMM•Cl as activator.

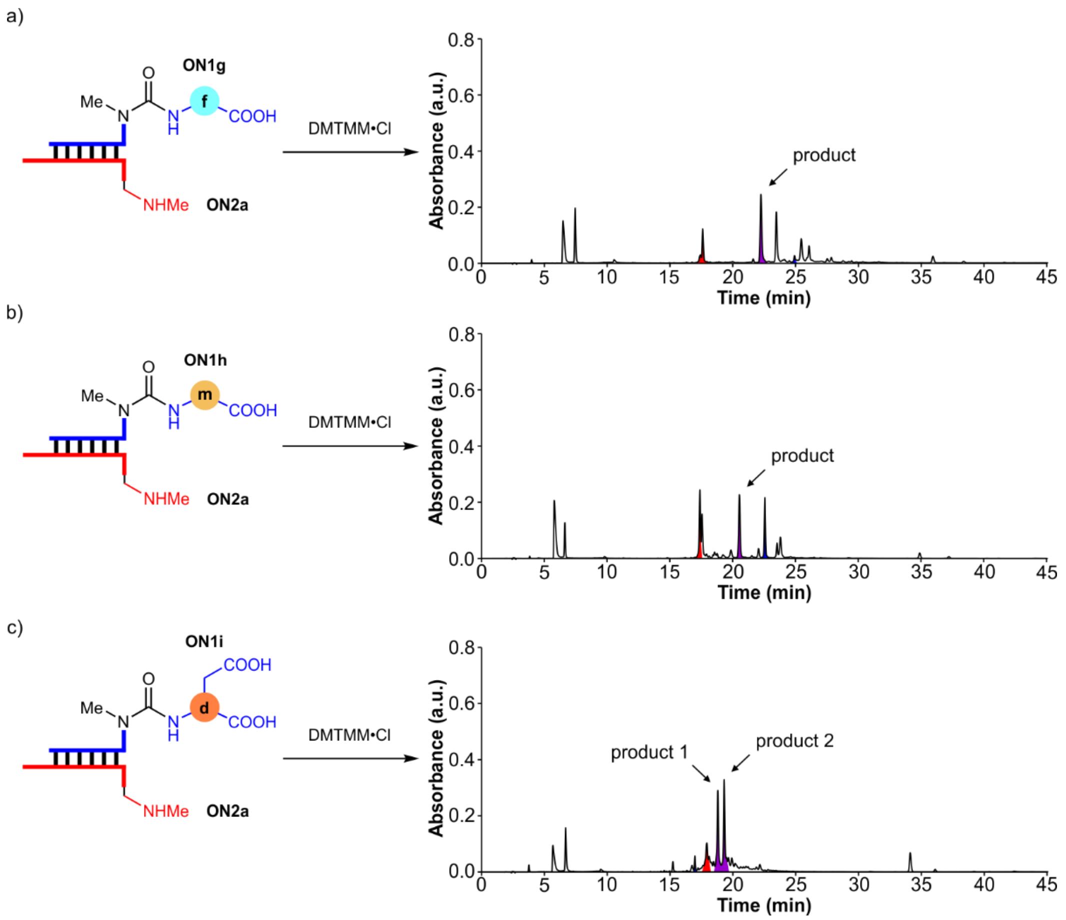

Figure S22. HPL-chromatograms of the reactions of **ON2a**; **X** = mnm^{5}U with: a) **ON1g**; **X** = m^{6}f^{6}A; b) **ON1h**; **X** = m^{6}m^{6}A and c) **ON1i**; **X** = m^{6}d^{6}A in MES buffer at pH 6 using DMTMM•Cl as activator. For **ON1i**, the two peaks corresponded to the products of the reaction of the Asp *α*-COOH and of the side chain COOH. An assignment was not performed.

Table S14. Results obtained in the coupling reactions of **ON1b-i**; **X** = m^{6}aa^{6}A with **ON2a** using DMTMM•Cl as activator (average of, at least, two experiments).

Donor strand	Acceptor strand	Average Yield ± Error (%)[a]
**ON1b**; **X** = m^{6}a^{6}A	**ON2a**; **X** = mnm^{5}U	51±1
**ON1c**; **X** = m^{6}v^{6}A		21±1
**ON1d**; **X** = m^{6}l^{6}A		27±1
**ON1e**; **X** = m^{6}t^{6}A		18±5
**ON1f**; **X** = m^{6}p^{6}A		11±1
**ON1g**; **X** = m^{6}f^{6}A		27±1
**ON1h**; **X** = m^{6}m^{6}A		22±1
**ON1i**; **X** = m^{6}d^{6}A		28±4; 26±3[b]

[a] Calculated yield from the chromatographic peak of the product using the calibration curve of **CON3**. [b] For **ON1i**, the two yields describe the reaction of the Asp *α*-COOH and of the side chain COOH. An assignment was not performed.

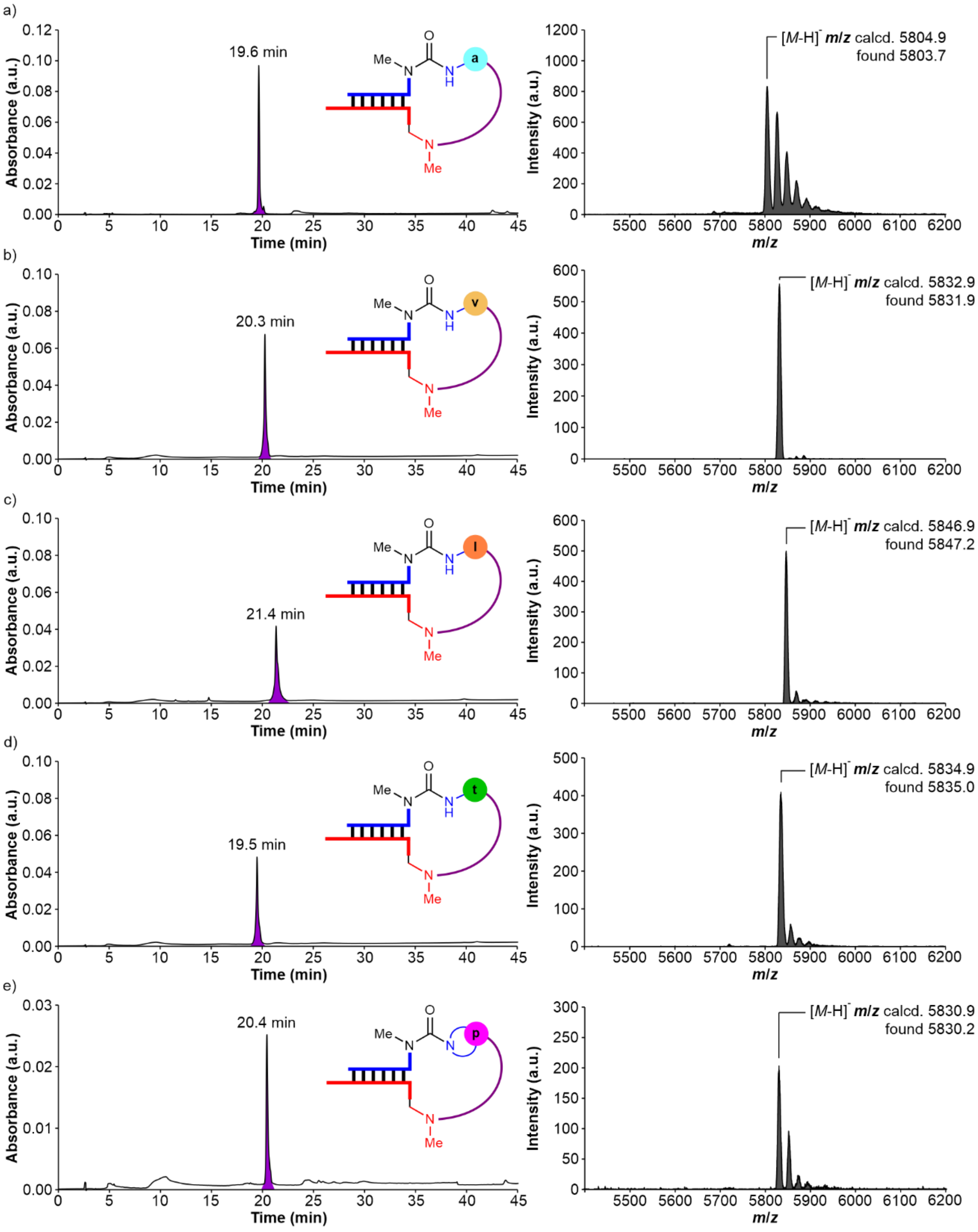

Figure S23. left) HPL-chromatograms and right) MALDI-TOF mass spectra (negative mode) of the isolated products from the reactions of **ON2a**; **X** = mnm^{5}U with: a) **ON1b**; **X** = m^{6}a^{6}A; b) **ON1c**; **X** = m^{6}v^{6}A; c) **ON1d**; **X** = m^{6}l^{6}A; d) **ON1e**; **X** = m^{6}t^{6}A and e) **ON1f**; **X** = m^{6}p^{6}A.

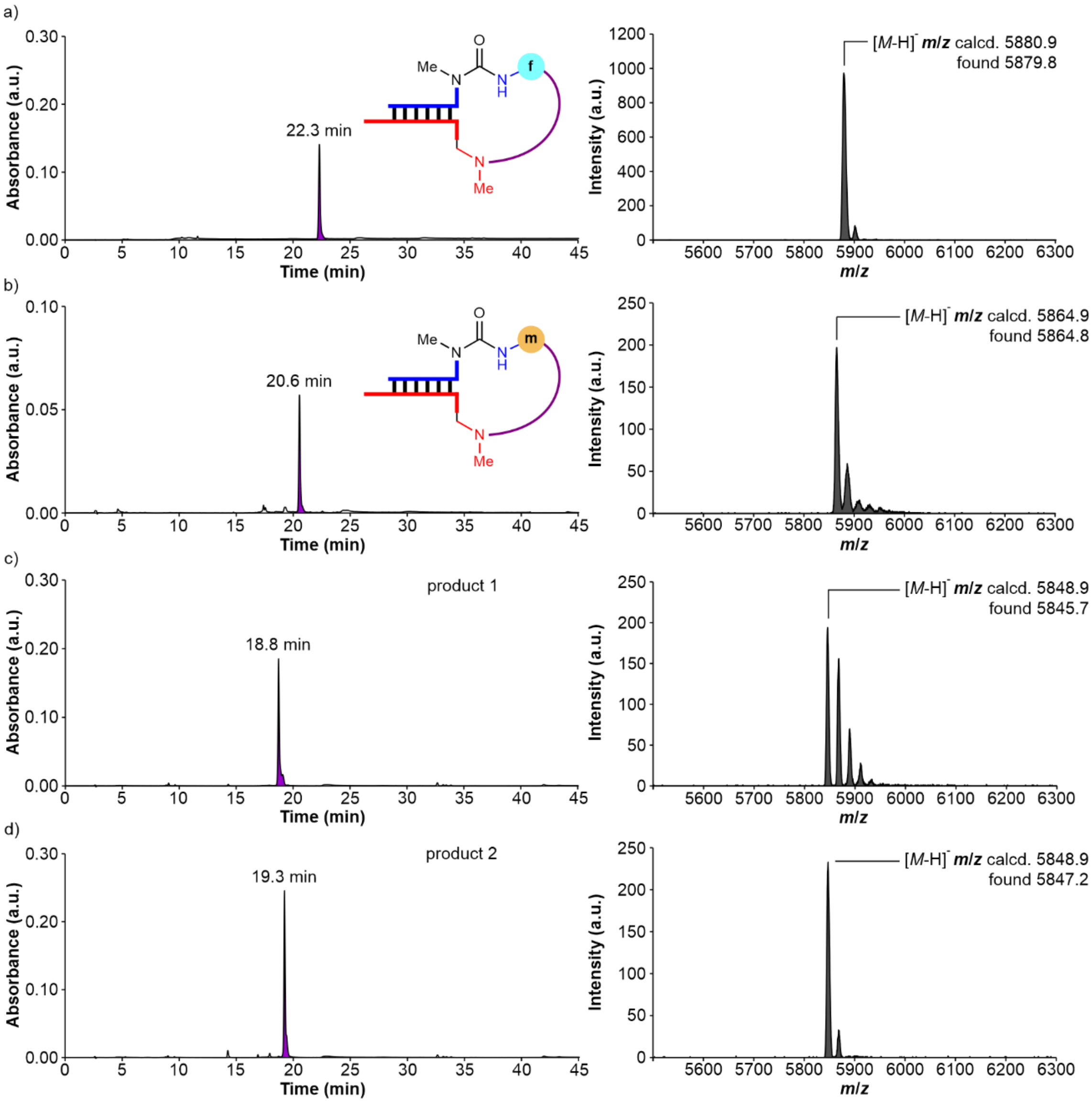

Figure S24. left) HPL-chromatograms and right) MALDI-TOF mass spectra (negative mode) of the isolated products from the reactions of **ON2a**; **X** = mnm^{5}U with: a) **ON1g**; **X** = m^6<u>f</u>^{6}A; b) **ON1h**; **X** = m^6<u>m</u>^{6}A; c) **ON1i**; **X** = m^6<u>d</u>^{6}A and d) **ON1i**; **X** = m^6<u>d</u>^{6}A.

6.7 Coupling reactions of ON1b-i (m^{6}aa^{6}A) with ON2c

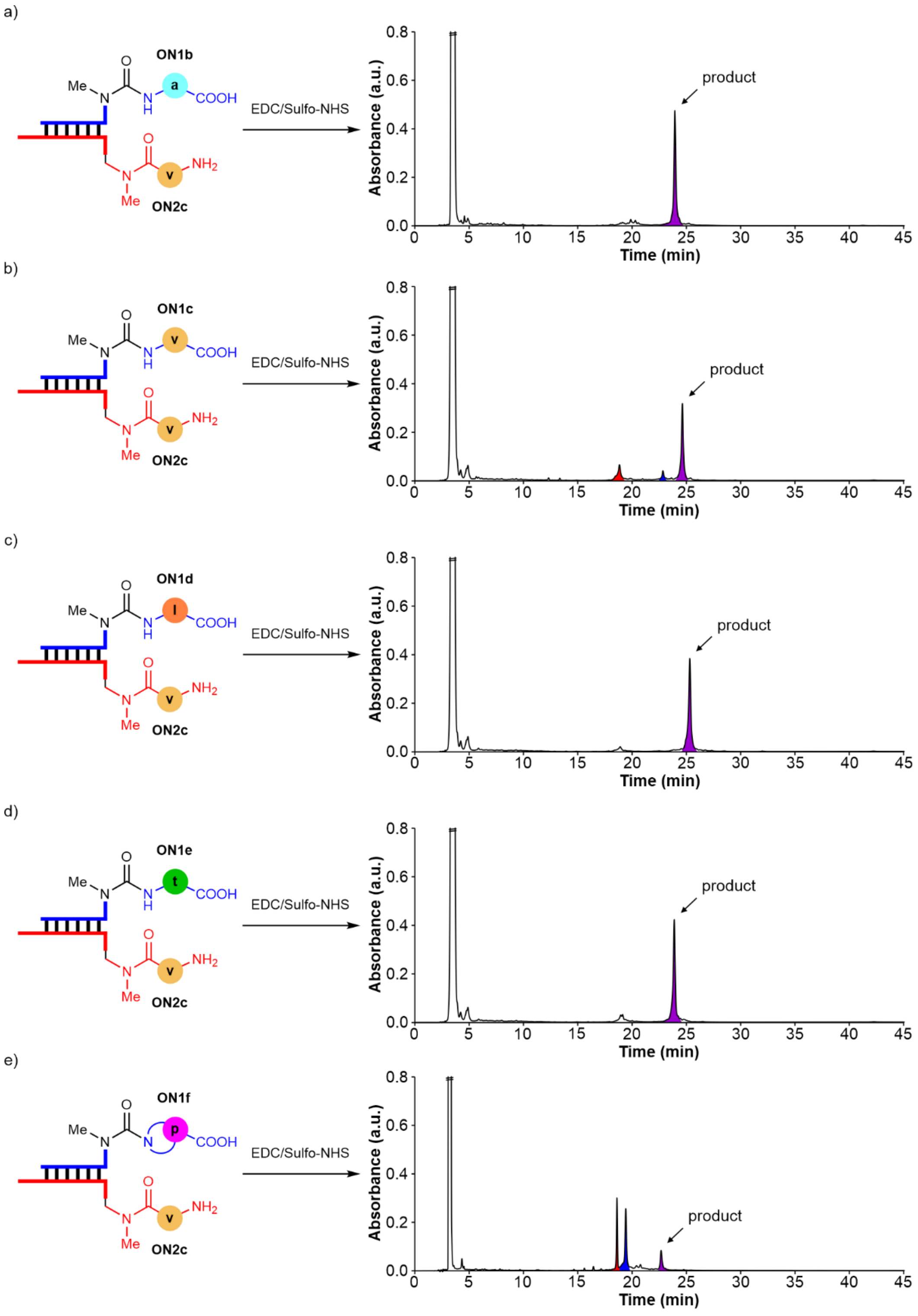

Figure S25. HPL-chromatograms of the reactions of **ON2c**; **X** = $\underline{v}$mnm^{5}U with: a) **ON1b**; **X** = m$^6\underline{a}^6$A; b) **ON1c**; **X** = m$^6\underline{v}^6$A; c) **ON1d**; **X** = m$^6\underline{l}^6$A; d) **ON1e**; **X** = m$^6\underline{t}^6$A and e) **ON1f**; **X** = m$^6\underline{p}^6$A in MES buffer at pH 6 using EDC/Sulfo-NHS as activator.

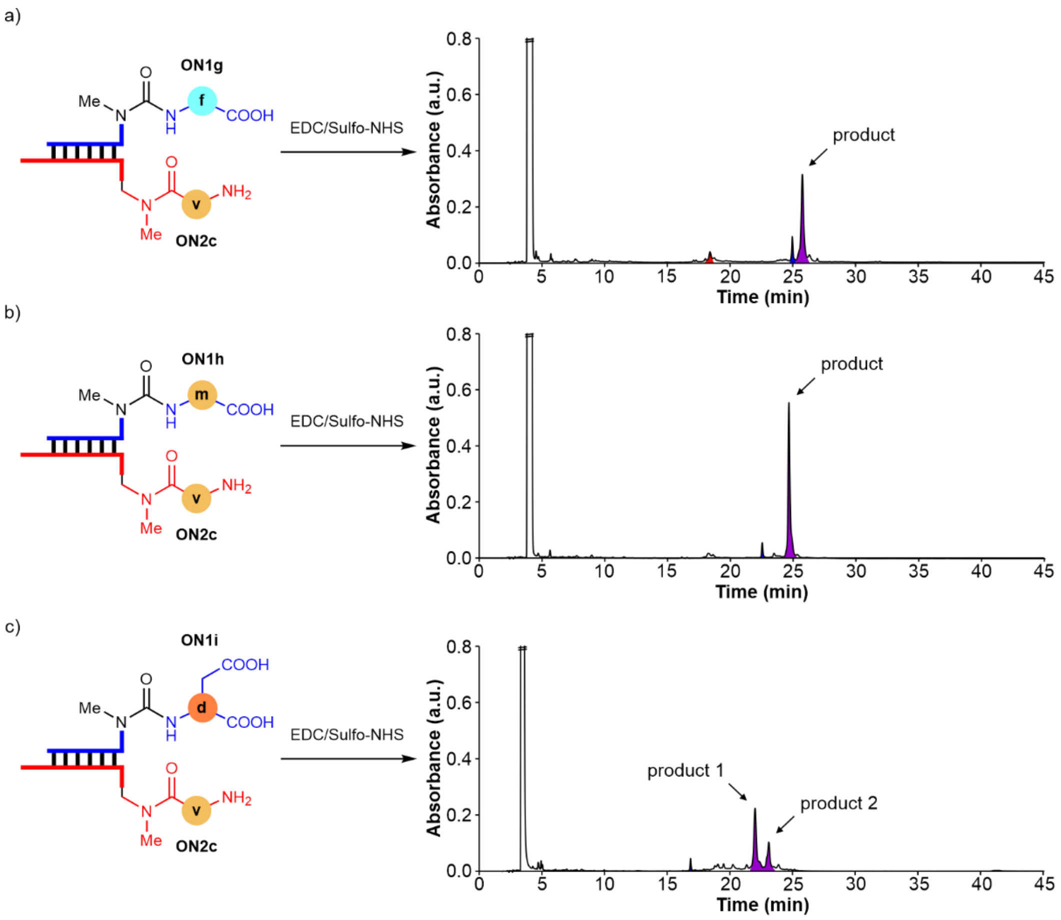

Figure S26. HPL-chromatograms of the reactions of **ON2c**; **X** = $\underline{v}$mnm^{5}U with: a) **ON1g**; **X** = m$^6\underline{f}^6$A; b) **ON1h**; **X** = m$^6\underline{m}^6$A and c) **ON1i**; **X** = m$^6\underline{d}^6$A in MES buffer at pH 6 using EDC/Sulfo-NHS as activator. For **ON1i**, the two peaks corresponded to the products of the reaction of the Asp *α*-COOH and of the side chain COOH. An assignment was not performed.

Table S15. Results obtained in the coupling reactions of **ON1b**-**i**; **X** = m$^6\underline{aa}^6$A with **ON2c** using EDC/Sulfo-NHS as activator (average of, at least, two experiments).

Donor strand	Acceptor strand	Average Yield ± Error (%)[a]
**ON1b**; **X** = m$^6\underline{a}^6$A		76±2
**ON1c**; **X** = m$^6\underline{v}^6$A		54±1
**ON1d**; **X** = m$^6\underline{l}^6$A		77±1
**ON1e**; **X** = m$^6\underline{t}^6$A		77±1
**ON1f**; **X** = m$^6\underline{p}^6$A	**ON2c**; **X** = $\underline{v}$mnm^{5}U	18±4 (55±5)[b]
**ON1g**; **X** = m$^6\underline{f}^6$A		50±1
**ON1h**; **X** = m$^6\underline{m}^6$A		70±2
**ON1i**; **X** = m$^6\underline{d}^6$A		34±1; 17±2[c]

[a] Calculated yield from the chromatographic peak of the product using the calibration curve of **CON3**. [b] Using DMTMM•Cl as activator. [c] For **ON1i**, the two yields describe the reaction of the Asp *α*-COOH and of the side chain COOH. An assignment was not performed.

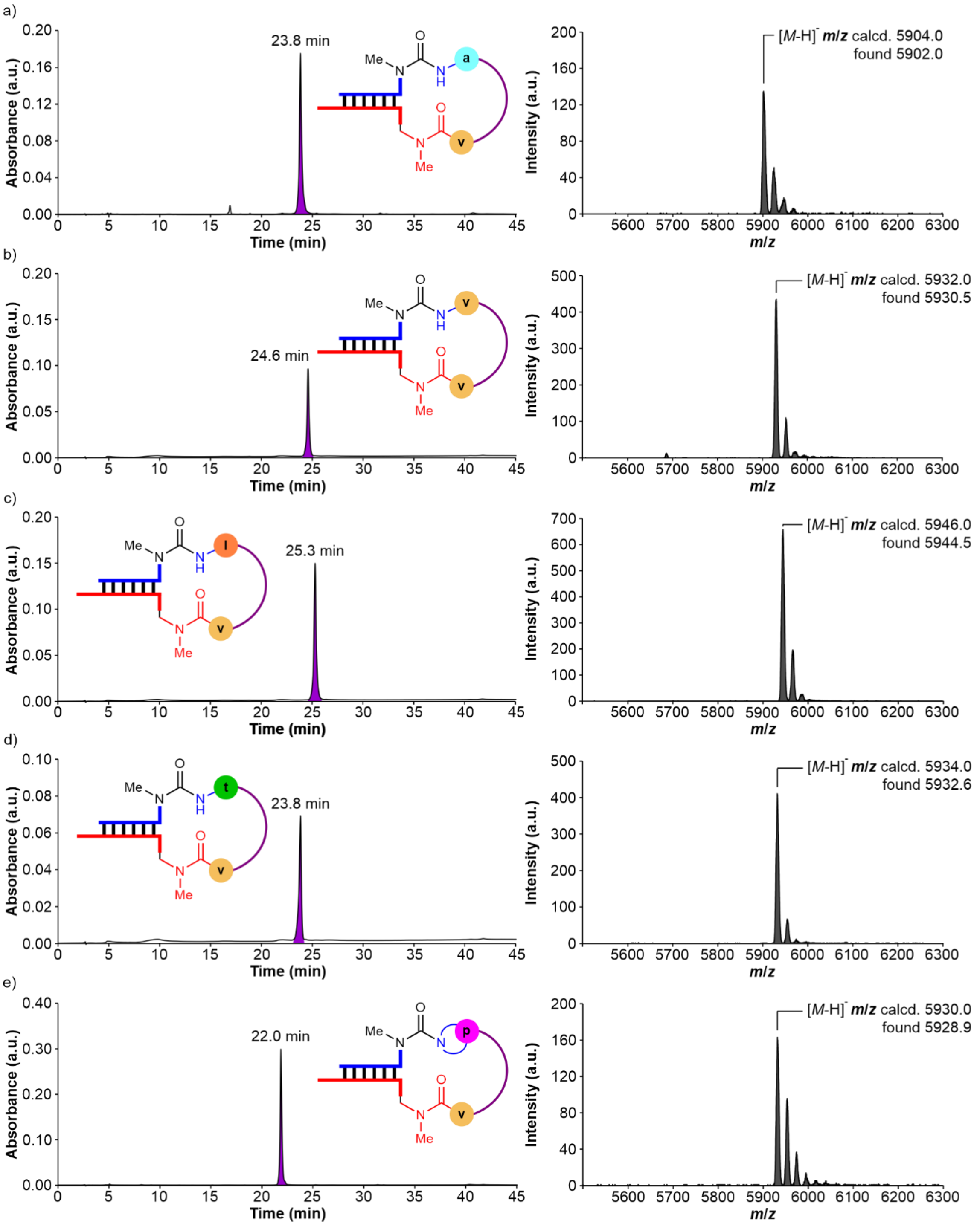

Figure S27. left) HPL-chromatograms and right) MALDI-TOF mass spectra (negative mode) of the isolated products from the reactions of **ON2c**; **X** = vmnm^{5}U with: a) **ON1b**; **X** = m^{6}a^{6}A; b) **ON1c**; **X** = m^{6}v^{6}A; c) **ON1d**; **X** = m^{6}l^{6}A; d) **ON1e**; **X** = m^{6}t^{6}A and e) **ON1f**; **X** = m^{6}p^{6}A.

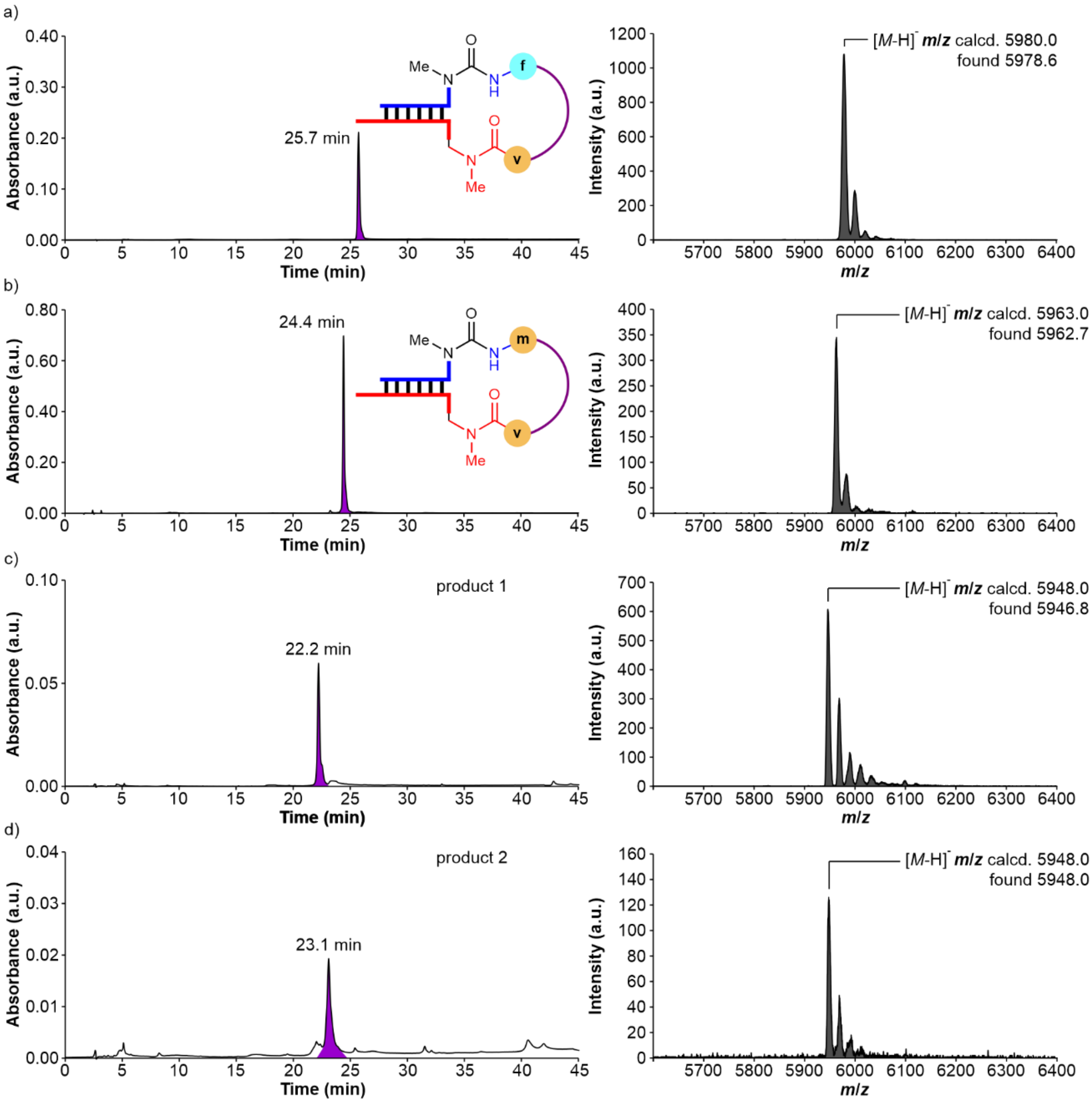

Figure S28. left) HPL-chromatograms and right) MALDI-TOF mass spectra (negative mode) of the isolated products from the reactions of **ON2c**; **X** = $\underline{\text{v}}$mnm^{5}U with: a) **ON1g**; **X** = m$^6\underline{\text{f}}^6$A; b) **ON1h**; **X** = m$^6\underline{\text{m}}^6$A; c) **ON1i**; **X** = m$^6\underline{\text{d}}^6$A and d) **ON1i**; **X** = m$^6\underline{\text{d}}^6$A.

7. Synthesized peptide-oligonucleotides using solid support beads

7.1 Donor peptide-oligonucleotides with a complementary sequence

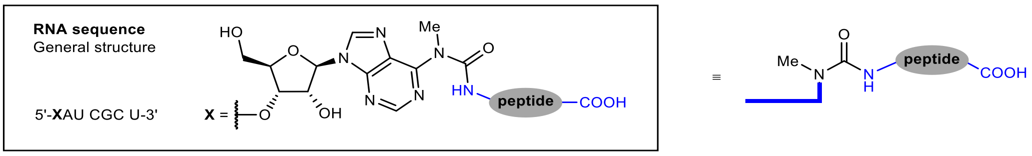

Figure S29. RNA sequence and general structure of peptide-modified carbamoyl adenosine derivatives.

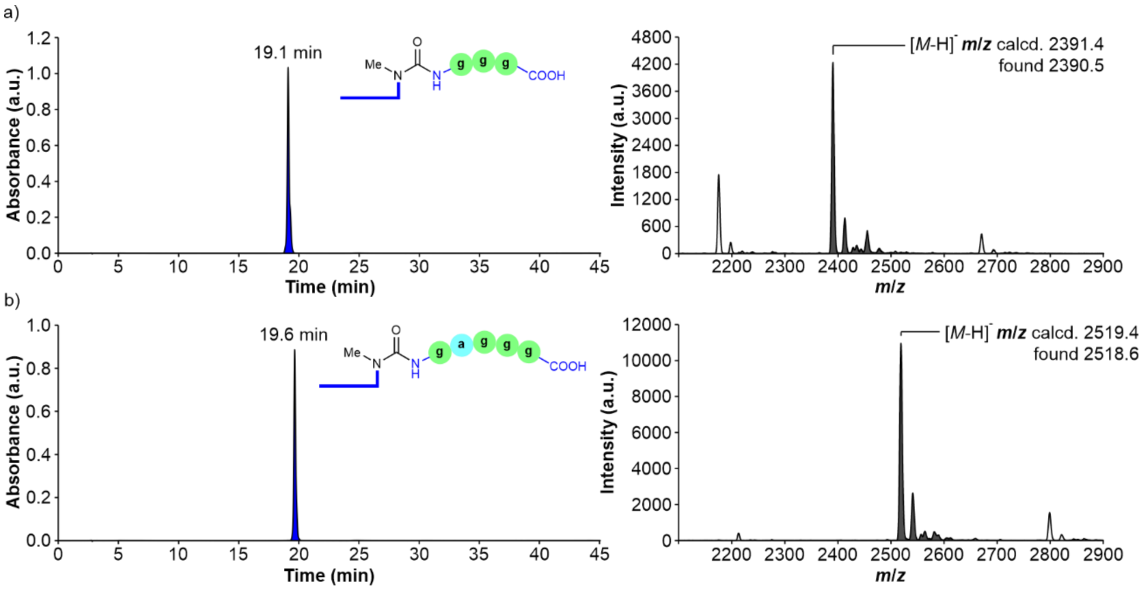

Figure S30. left) HPL-chromatograms and right) MALDI-TOF mass spectra (negative mode) of the synthesized peptide-oligonucleotides: a) 5'-m^6(ggg)^{6}A-RNA-3' and b) 5'-m^6(gaggg)^{6}A-RNA-3'.

7.2 Acceptor peptide-oligonucleotides with a complementary sequence

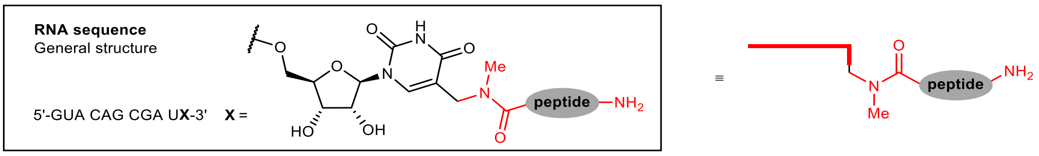

Figure S31. RNA sequence and general structure of peptide-modified methylaminomethyl uridine derivatives.

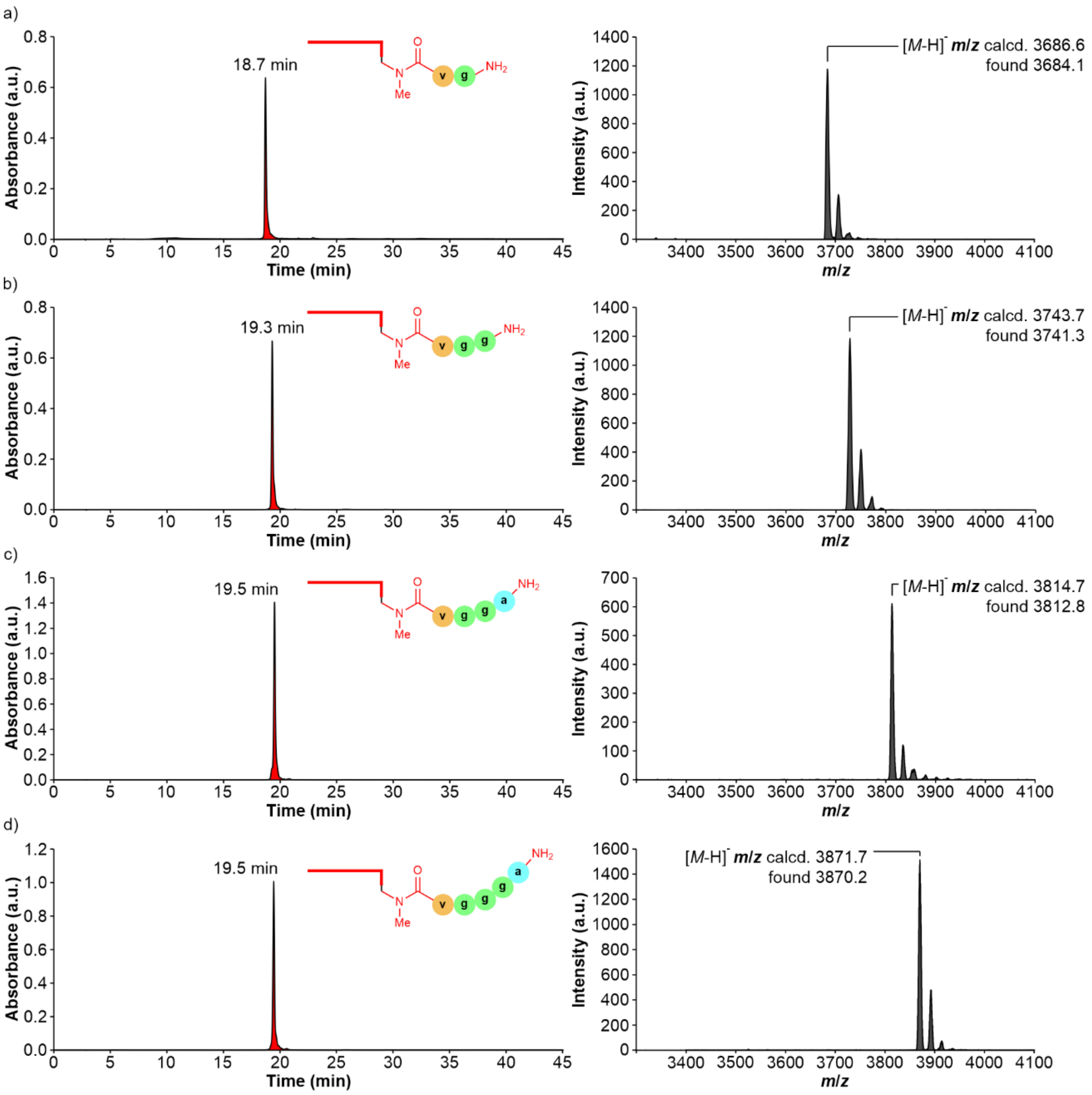

Figure S32. left) HPL-chromatograms and right) MALDI-TOF mass spectra (negative mode) of the synthesized peptide-oligonucleotides: a) 3'-gvmnm^{5}U-RNA-5'; b) 3'-ggvmnm^{5}U-RNA-5'; c) 3'-aggvmnm^{5}U-RNA-5' and d) 3'-agggvmnm^{5}U-RNA-5'.

5’-GmUmAm CmAmGm CmGmAm Um**X**-3’; **X** = agg mnm^{5}U

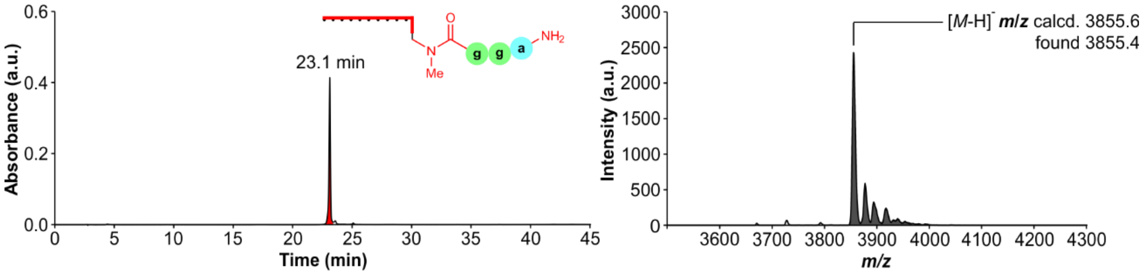

Figure S33. left) HPL-chromatogram and right) MALDI-TOF mass spectrum (negative mode) of the synthesized peptide-oligonucleotide 3’-aggmnm^{5}U-RNA-5’ containing 2’-OMe nucleosides.

8. Coupling reactions between donor and acceptor peptide-oligonucleotides

The peptide coupling reactions were carried out under identical conditions to those described in Section 0.

8.1 Coupling reactions of donor peptide-oligonucleotides with ON2c

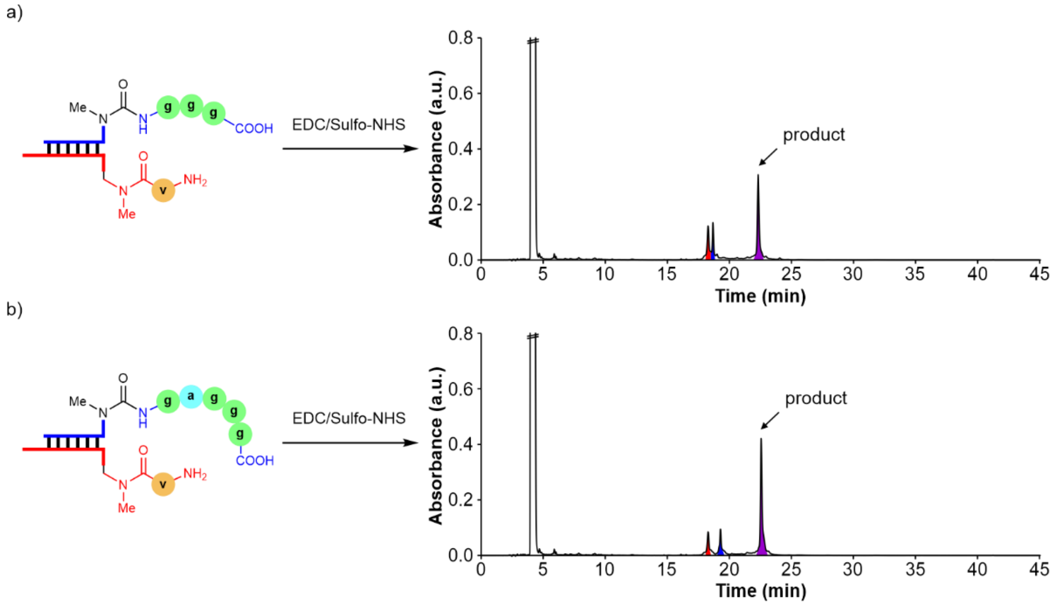

Figure S34. HPL-chromatograms of the reactions of **ON2c**; **X** = vmnm^{5}U with: a) 5’-m^6(ggg)^{6}A-RNA-3’ and b) 5’-m^6(gaggg)^{6}A-RNA-3’ in MES buffer at pH 6 using EDC/Sulfo-NHS as activator.

Table S16. Results obtained in the coupling reactions of **ON2c**; **X** = vmnm^{5}U with peptide-modified donor oligonucleotides using EDC/Sulfo-NHS as activator (average of, at least, two experiments).

Donor strand	Acceptor strand	Average Yield ± Error (%)[a]
5'-m^6(ggg)^{6}A-RNA-3'	**ON2c**; **X** = vmnm^{5}U	35±1
5'-m^6(gaggg)^{6}A-RNA-3'		43±1

[a] Calculated yield from the chromatographic peak of the product using the calibration curve of **CON3**.

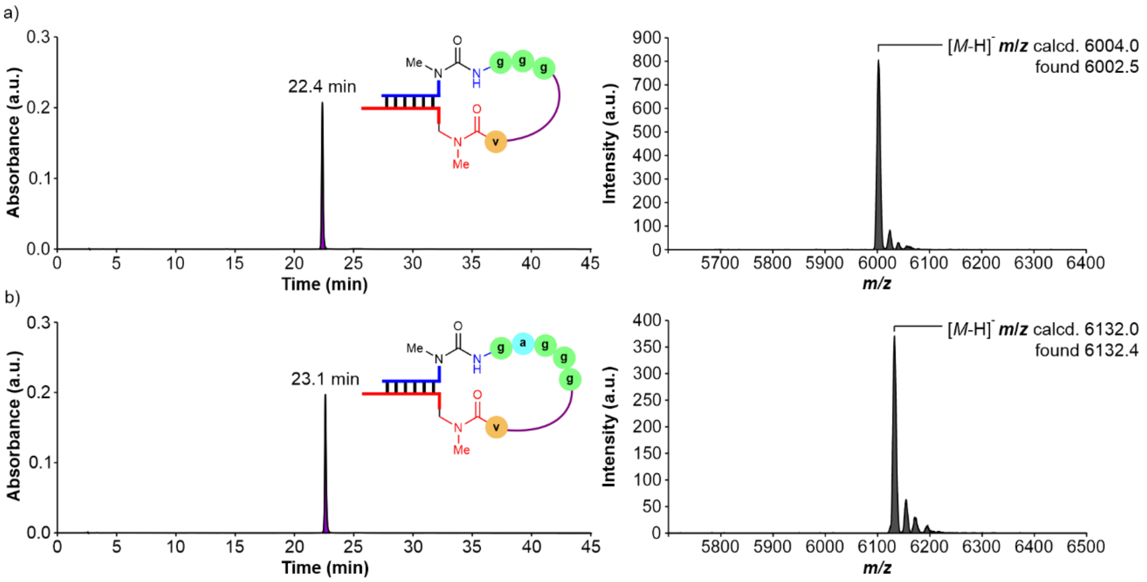

Figure S35. left) HPL-chromatograms and right) MALDI-TOF mass spectra (negative mode) of the isolated products from the reactions of **ON2c**; **X** = vmnm^{5}U with: a) 5'-m^6(ggg)^{6}A-RNA-3' and b) 5'-m^6(gaggg)^{6}A-RNA-3'.

8.2 Coupling reactions of ON1a (m^6g^6A) with acceptor peptide-oligonucleotides

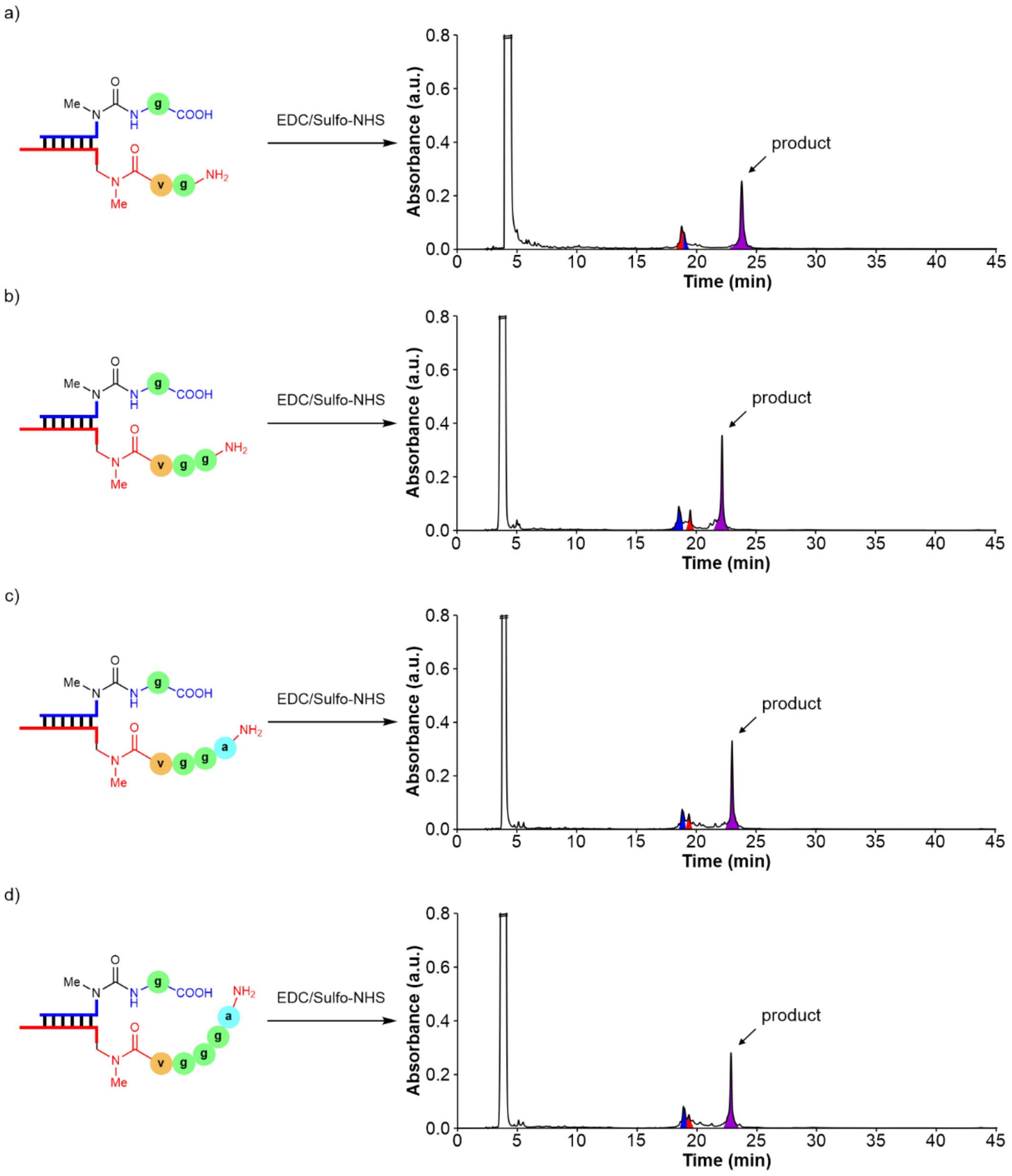

Figure S36. HPL-chromatograms of the reactions of **ON1a**; **X** = m^6g^6A with: a) 3'-gvmnm^{5}U-RNA-5'; b) 3'-ggvmnm^{5}U-RNA-5'; c) 3'-aggvmnm^{5}U-RNA-5' and d) 3'-agggvmnm^{5}U-RNA-5' in MES buffer at pH 6 using EDC/Sulfo-NHS as activator.

Table S17. Results obtained in the coupling reactions of **ON1a**; **X** = m^6g^6A with peptide-modified acceptor oligonucleotides using EDC/Sulfo-NHS as activator (average of, at least, two experiments).

Donor strand	Acceptor strand	Average Yield ± Error (%)[a]
**ON1a**; **X** = m^6g^6A	3'-gvmnm^{5}U-RNA-5'	51±1
	3'-ggvmnm^{5}U-RNA-5'	46±4
	3'-aggvmnm^{5}U-RNA-5'	40±1
	3'-agggvmnm^{5}U-RNA-5'	40±3 (57±2)[b]

[a] Calculated yield from the chromatographic peak of the product using the calibration curve of **CON3**. [b] Using DMTMM•Cl as activator.

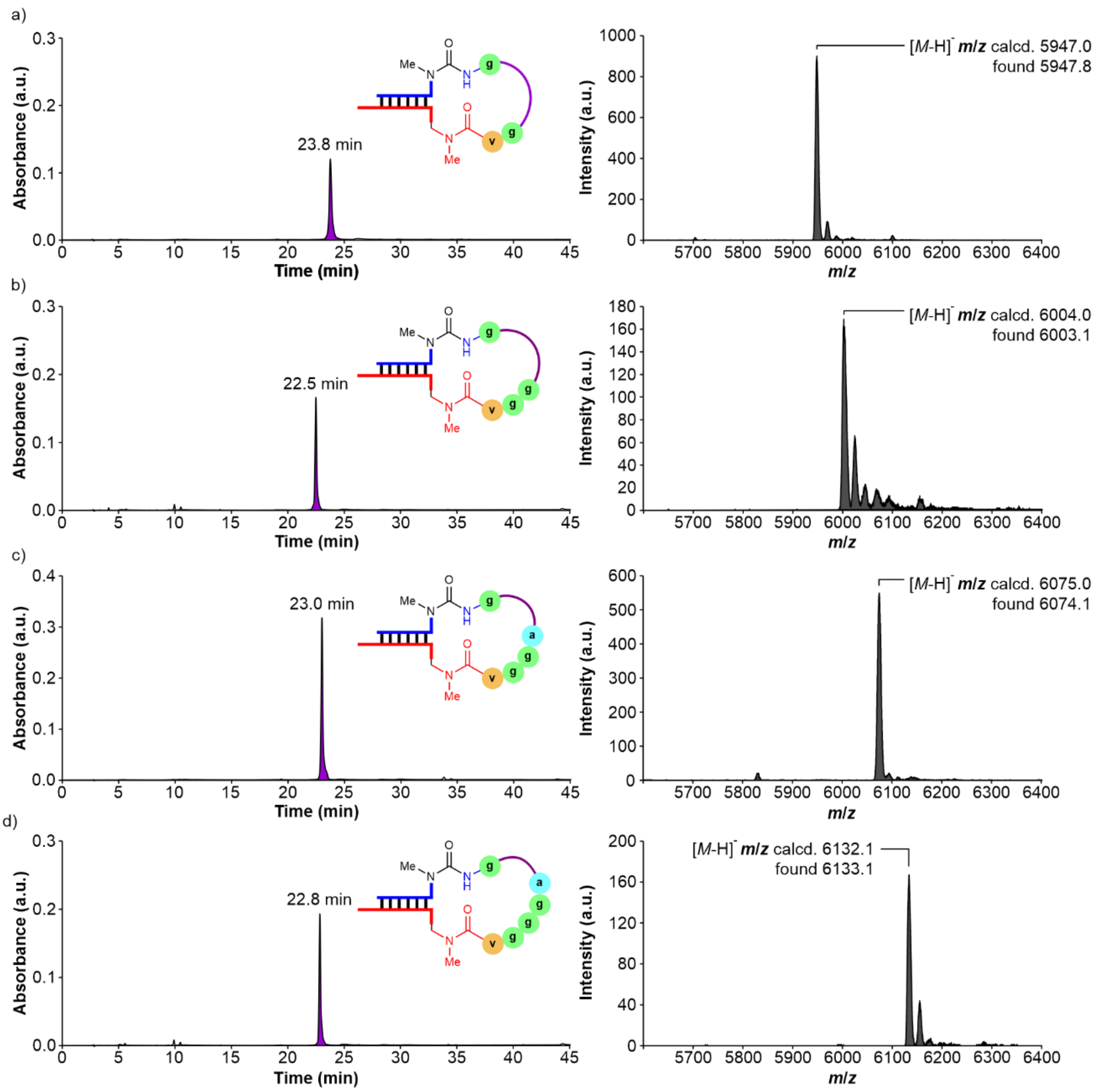

Figure S37. left) HPL-chromatograms and right) MALDI-TOF mass spectra (negative mode) of the isolated products from the reactions of **ON1a**; **X** = m^{6}g^{6}A with: a) 3'-gvmnm^{5}U-RNA-5'; b) 3'-ggvmnm^{5}U-RNA-5'; c) 3'-aggvmnm^{5}U-RNA-5' and d) 3'-agggvmnm^{5}U-RNA-5'.

8.3 Coupling reactions of donor and acceptor peptide-oligonucleotides

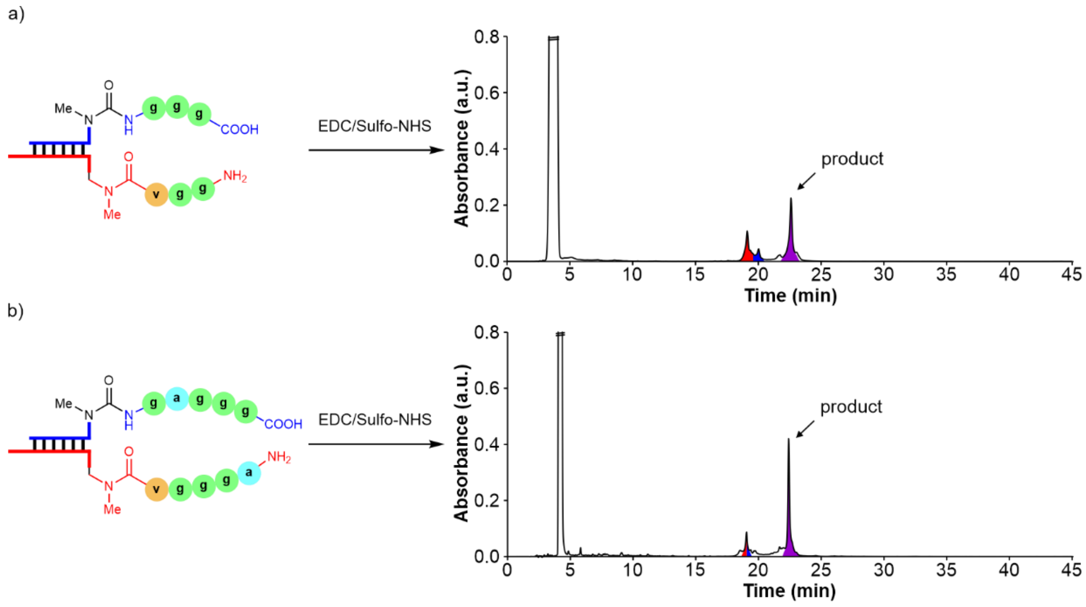

Figure S38. HPL-chromatograms of the reactions of: a) 5'-m^6(ggg)^{6}A-RNA-3' with 3'-ggvmnm^{5}U-RNA-5' and b) 5'-m^6(gaggg)^{6}A-RNA-3' with 3'-agggvmnm^{5}U-RNA-5' in MES buffer at pH 6 using EDC/Sulfo-NHS as activator.

Table S18. Results obtained in the coupling reactions of peptide-modified donor and acceptor oligonucleotides using EDC/Sulfo-NHS as activator (average of, at least, two experiments).

Donor strand	Acceptor strand	Average Yield ± Error (%)[a]
5'-m^6(ggg)^{6}A-RNA-3'	3'-ggvmnm^{5}U-RNA-5'	53±1
5'-m^6(gaggg)^{6}A-RNA-3'	3'-agggvmnm^{5}U-RNA-5'	56±3

[a] Calculated yield from the chromatographic peak of the product using the calibration curve of **CON3**.

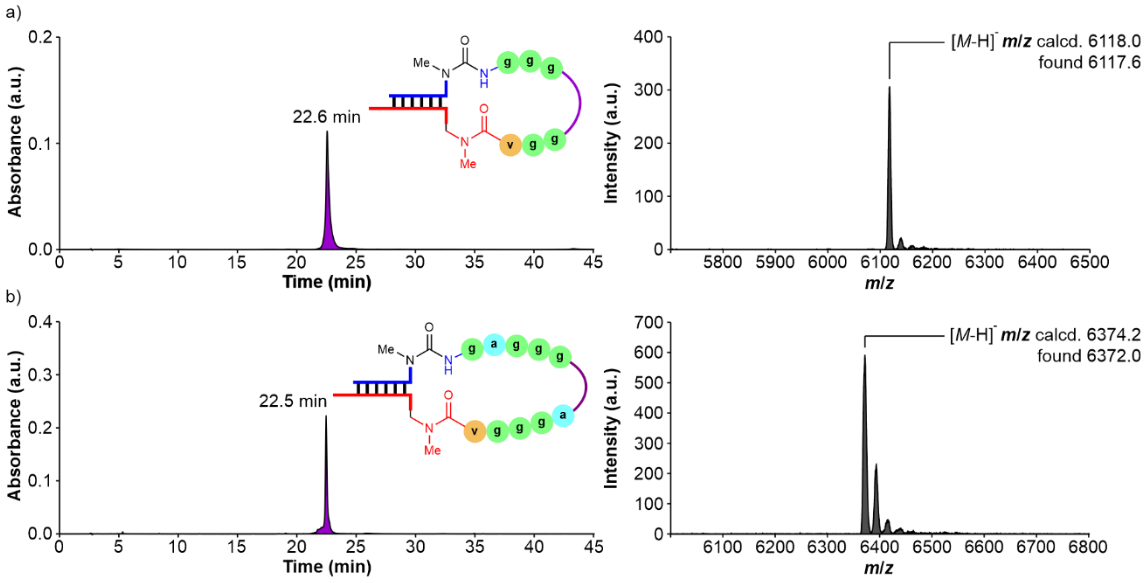

Figure S39. left) HPL-chromatograms and right) MALDI-TOF mass spectra (negative mode) of the isolated products from the reactions of: a) 5'-m^6(ggg)^{6}A-RNA-3' with 3'-ggvmnm^{5}U-RNA-5' and b) 5'-m^6(gaggg)^{6}A-RNA-3' with 3'-agggvmnm^{5}U-RNA-5'.

9. Concentration of the product versus time in selected coupling reactions

The peptide coupling reactions were carried out under identical conditions to those described in Section 0 using DMTMM•Cl as activator.

The data (concentration of product *vs.* time) was fit to the corresponding theoretical kinetic model using the Parameter Estimation Module of COPASI software Version 4.29.[12] We introduced the theoretical kinetic model shown below:

Double strand → Hairpin-type Intermediate; k_{app}

The initial concentration of the double strand was refined as variable but constrained between 30 and 50 × 10^{-6} M. The fit of the data returned the rate constant value k_{app}. This fitting procedure is similar to that reported by others in the literature.[13]

In all cases, the fit of the experimental data was good based on the residual values, reported as sum of squared residuals (SSR), and the visual inspection of the curves.

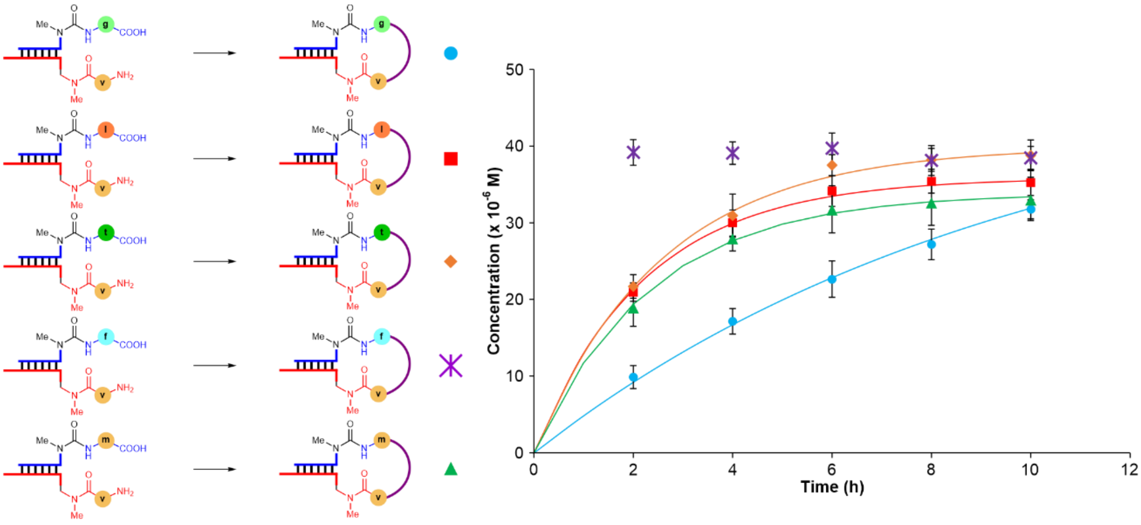

Figure S40. Concentration of the product (M) *vs.* time (h) in selected peptide coupling reactions using DMTMM•Cl as activator. Lines show fit of the data to the theoretical kinetic model. Error bars are the standard deviations.

Table S19. Calculated rate constant values for selected coupling reactions (average of, at least, two experiments).

Donor strand	Acceptor strand	k_{app} (h^{-1})[a]	SSR[b]
**ON1a**; **X** = m^{6}g^{6}A		0.12±0.02	2.00 × 10^{-12}
**ON1d**; **X** = m^{6}l^{6}A		0.42±0.02	8.20 × 10^{-13}
**ON1e**; **X** = m^{6}t^{6}A	**ON2c**; **X** = vmnm^{5}U	0.39±0.04	2.50 × 10^{-12}
**ON1g**; **X** = m^{6}f^{6}A		>1	n.d.
**ON1h**; **X** = m^{6}m^{6}A		0.42±0.04	5.80 × 10^{-13}

[a] Errors are indicated as standard deviations. [b] SSR = Sum of squared residuals.

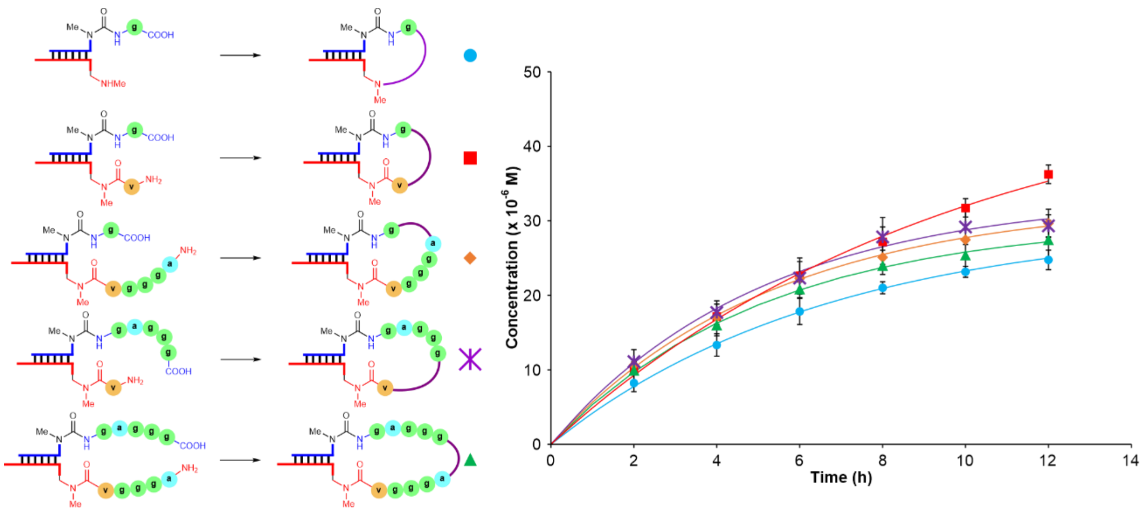

Figure S41. Concentration of the product (M) *vs.* time (h) in selected peptide coupling reactions using DMTMM•Cl as activator. Lines show fit of the data to the theoretical kinetic model. Error bars are the standard deviations.

Table S20. Calculated rate constant values for selected coupling reactions (average of, at least, two experiments).

Donor strand	Acceptor strand	k_{app} (h^{-1})[a]	SSR[b]
**ON1a**; **X** = m^{6}g^{6}A	**ON2a**; **X** = mnm^{5}U	0.14±0.02	3.36 × 10^{-13}
**ON1a**; **X** = m^{6}g^{6}A	**ON2c**; **X** = vmnm^{5}U	0.12±0.02	2.00 × 10^{-12}
**ON1a**; **X** = m^{6}g^{6}A	3'-agggvmnm^{5}U-RNA-5'	0.18±0.02	5.59 × 10^{-13}
5'-m^6(gaggg)^{6}A-RNA-3'	**ON2c**; **X** = vmnm^{5}U	0.19±0.02	3.82 × 10^{-12}
5'-m^6(gaggg)^{6}A-RNA-3'	3'-agggvmnm^{5}U-RNA-5'	0.19±0.01	4.45 × 10^{-13}

[a] Errors are indicated as standard deviations. [b] SSR = Sum of squared residuals.

10. Coupling reactions between oligonucleotides containing multiple donor or acceptor units

The peptide coupling reactions were carried out under identical conditions to those described in Section 0.

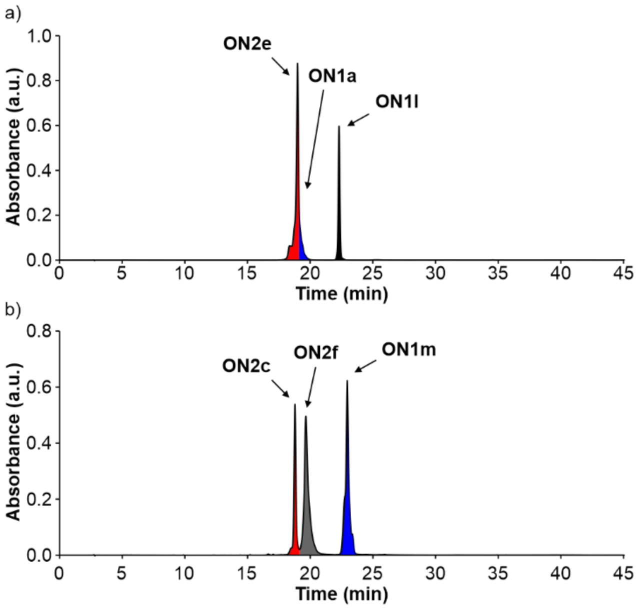

Figure S42. HPL-chromatograms of equimolar mixtures of: a) **ON1a**; **X** = m^{6}g^{6}A, **ON1l**; **X** = m^{6}v^{6}A and **ON2e**; **X^1** = gmnm^{5}U and **X^2** = nm^{5}U, and b) **ON1m**; **X^1** = m^{6}v^{6}A and **X^2** = m^{6}g^{6}A, **ON2c**; **X** = vmnm^{5}U and **ON2f**; **X** = vmnm^{5}U.

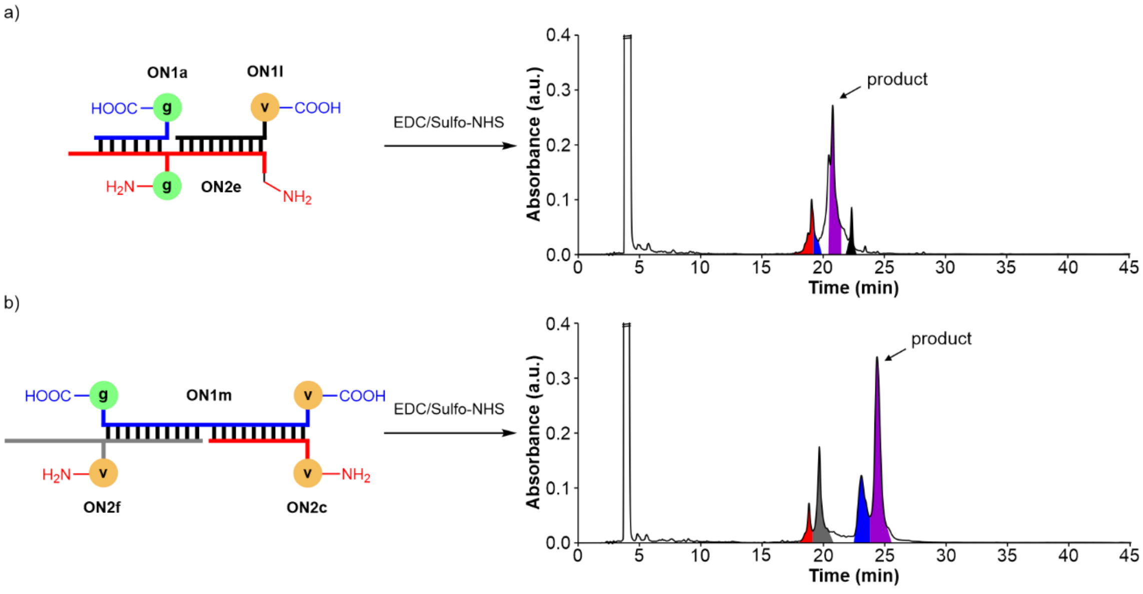

Figure S43. HPL-chromatograms of the reactions of: a) **ON1a**; **X** = $\mathrm{m^6\underline{g}^6A}$, **ON1l**; **X** = $\mathrm{m^6\underline{v}^6A}$ and **ON2e**; **X¹** = $\mathrm{\underline{g}mnm^5U}$ and **X²** = $\mathrm{nm^5U}$ and b) **ON1m**; **X¹** = $\mathrm{m^6\underline{v}^6A}$ and **X²** = $\mathrm{m^6\underline{g}^6A}$, **ON2c**; **X** = $\mathrm{\underline{v}mnm^5U}$ and **ON2f**; **X** = $\mathrm{\underline{v}mnm^5U}$ in MES buffer at pH 6 using EDC/Sulfo-NHS as activator. The terminal functional groups of the ONs (urea and amide) are omitted for clarity.

Table S21. Results obtained in the coupling reactions of oligonucleotides containing multiple donor or acceptor units using EDC/Sulfo-NHS as activator (average of, at least, two experiments).

Donor strand	Acceptor strand	Average Yield ± Error (%)[a]
**ON1a**; **X** = $\mathrm{m^6\underline{g}^6A}$ **ON1l**; **X** = $\mathrm{m^6\underline{v}^6A}$	**ON2e**; **X¹** = $\mathrm{\underline{g}mnm^5U}$ and **X²** = $\mathrm{nm^5U}$	35±2 (29±1)[b]
**ON1m**; **X¹** = $\mathrm{m^6\underline{v}^6A}$ and **X²** = $\mathrm{m^6\underline{g}^6A}$	**ON2c**; **X** = $\mathrm{\underline{v}mnm^5U}$ **ON2f**; **X** = $\mathrm{\underline{v}mnm^5U}$	35±3 (32±2)[b]

[a] Calculated yield from the chromatographic peak of the product based on the total area of the initial components (Figure S42). [b] Using DMTMM•Cl as activator.

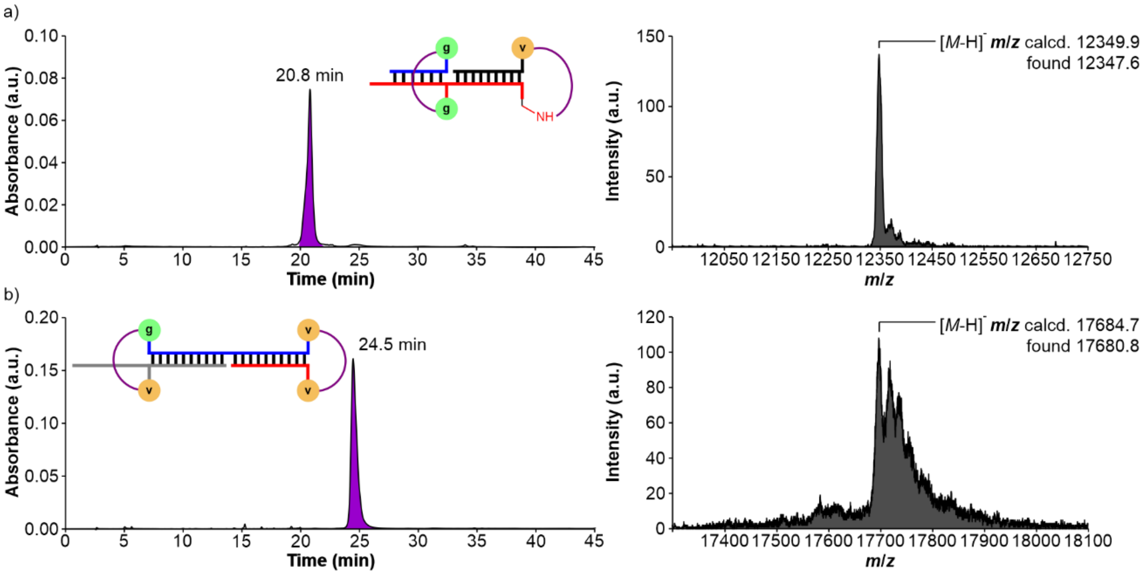

Figure S44. left) HPL-chromatograms and right) MALDI-TOF mass spectra (negative mode) of the isolated products from the reactions of: a) **ON1a**; **X** = $\mathrm{m^6\underline{g}^6A}$, **ON1l**; **X** = $\mathrm{m^6\underline{v}^6A}$ and **ON2e**; **X¹** = $\mathrm{\underline{g}mnm^5U}$ and **X²** = $\mathrm{nm^5U}$ and b) **ON1m**; **X¹** = $\mathrm{m^6\underline{v}^6A}$ and **X²** = $\mathrm{m^6\underline{g}^6A}$, **ON2c**; **X** = $\mathrm{\underline{v}mnm^5U}$ and **ON2f**; **X** = $\mathrm{\underline{v}mnm^5U}$.

11. Coupling reactions between ON2c and donor oligonucleotides with non-complementary sequences

The peptide coupling reactions were carried out under identical conditions to those described in Section 0.

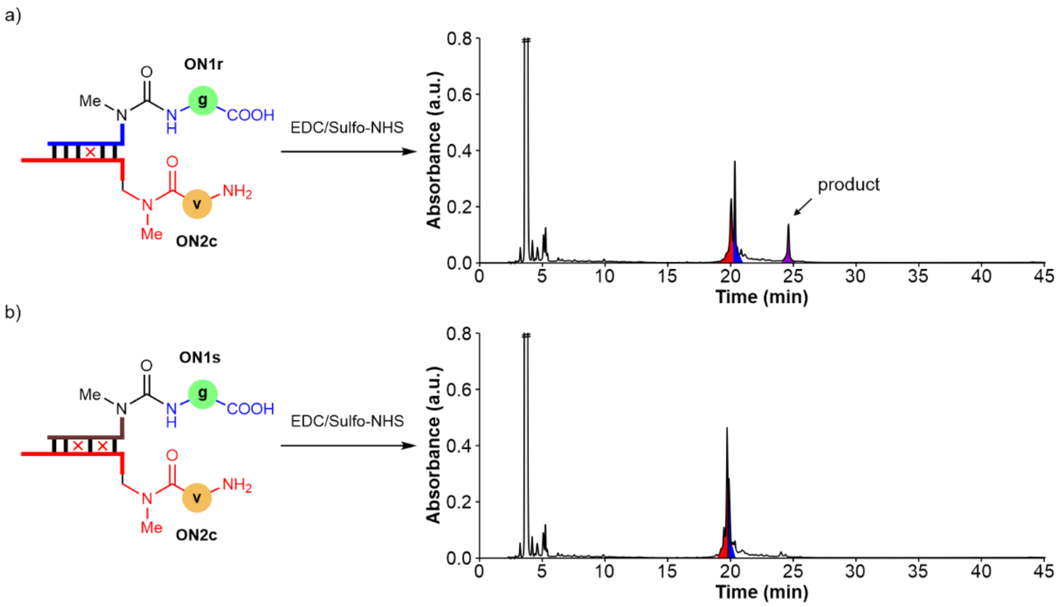

Figure S45. HPL-chromatograms of the reactions of **ON2c**; **X** = vmnm^{5}U with: a) **ON1r**; **X** = m^{6}g^{6}A and b) **ON1s**; **X** = m^{6}g^{6}A in MES buffer at pH 6 using EDC/Sulfo-NHS as activator.

Table S22. Results obtained in the coupling reactions of **ON2c**; **X** = vmnm^{5}U with **ON1r**; **X** = m^{6}g^{6}A or **ON1s**; **X** = m^{6}g^{6}A using EDC/Sulfo-NHS as activator.

Donor strand	Acceptor strand	Yield (%)[a]
**ON1r**; **X** = m^{6}g^{6}A	**ON2c**; **X** = vmnm^{5}U	~14 (~35)[b]
**ON1s**; **X** = m^{6}g^{6}A		< 3 (~12)[b]

[a] Estimated yield from the chromatographic peak of the product using the calibration curve of **CON3**. Note that we assumed that the formed product features an extinction coefficient similar to that of **CON3**. [b] Using DMTMM•Cl as activator.

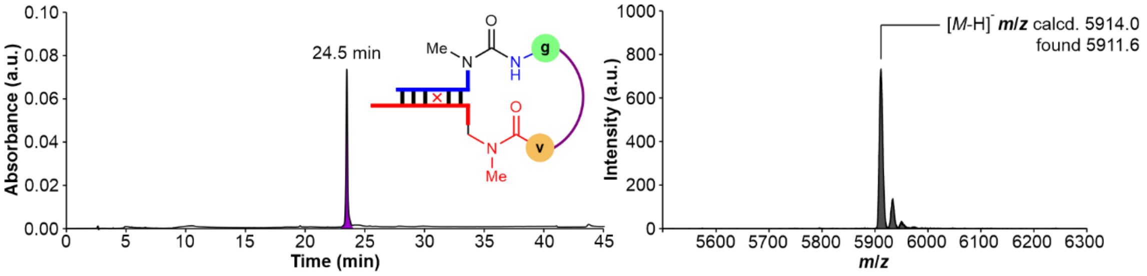

Figure S46. left) HPL-chromatogram and right) MALDI-TOF mass spectrum (negative mode) of the isolated product from the reaction of **ON2c**; **X** = vmnm^{5}U with **ON1r**; **X** = m^{6}g^{6}A.

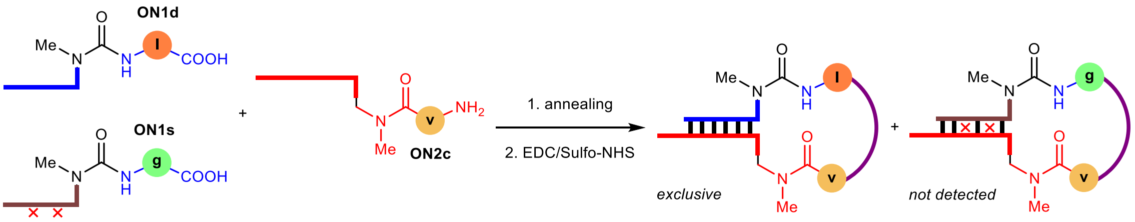

Scheme S12. Annealing and coupling reaction of **ON1d**; **X** = m^{6}l^{6}A, **ON1s**; **X** = m^{6}g^{6}A and **ON2c**; **X** = vmnm^{5}U. The formed peptide bonds are marked in purple.

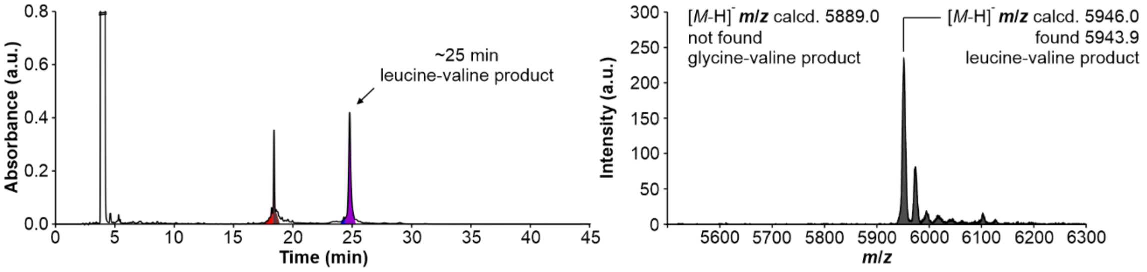

Figure S47. left) HPL-chromatogram and right) MALDI-TOF mass spectrum (negative mode) of the reaction of **ON1d**; **X** = m$^6\underline{l}^6$A, **ON1s**; **X** = m$^6\underline{g}^6$A and **ON2c**; **X** = $\underline{v}$mnm^{5}U in MES buffer at pH 6 using EDC/Sulfo-NHS as activator.

Table S23. Results obtained in the coupling reaction of **ON1d**; **X** = m$^6\underline{l}^6$A, **ON1s**; **X** = m$^6\underline{g}^6$A and **ON2c**; **X** = $\underline{v}$mnm^{5}U using EDC/Sulfo-NHS as activator (average of, at least, two experiments).

Donor strand	Acceptor strand	Average Yield ± Error of lv-peptide (%)[a]	Yield of gv-peptide (%)
**ON1d**; **X** = m$^6\underline{l}^6$A<br>**ON1s**; **X** = m$^6\underline{g}^6$A	**ON2c**; **X** = $\underline{v}$mnm^{5}U	65±2	not detected

[a] Calculated yield from the chromatographic peak of the product using the calibration curve of **CON3**.

12. Coupling reactions between ON2c and donor oligonucleotides with different lengths

The peptide coupling reactions were carried out under identical conditions to those described in Section 0.

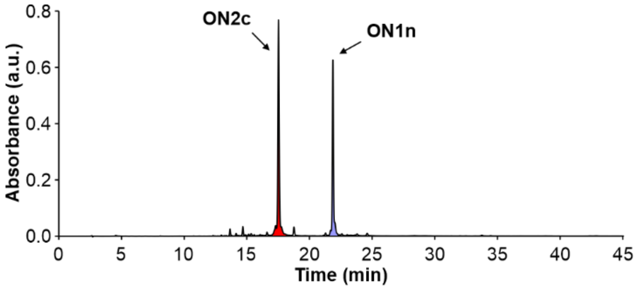

Figure S48. HPL-chromatogram of an equimolar mixture of **ON1n**; **X** = m$^6\underline{v}^6$A and **ON2c**; **X** = $\underline{v}$mnm^{5}U.

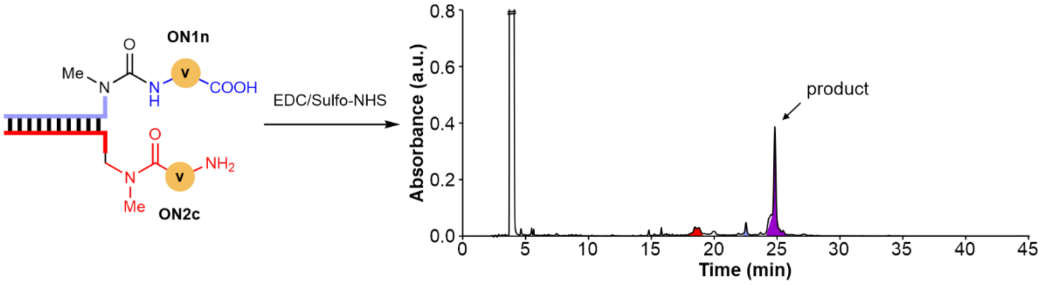

Figure S49. HPL-chromatogram of the reaction of **ON1n**; **X** = m$^6\underline{v}^6$A with **ON2c**; **X** = $\underline{v}$mnm^{5}U in MES buffer at pH 6 using EDC/Sulfo-NHS as activator.

Table S24. Result obtained in the coupling reaction of **ON1n**; **X** = m$^6\underline{v}^6$A with **ON2c**; **X** = $\underline{v}$mnm^{5}U using EDC/Sulfo-NHS as activator (average of, at least, two experiments).

Donor strand	Acceptor strand	Average Yield ± Error (%)[a]
**ON1n**; **X** = m$^6\underline{v}^6$A	**ON2c**; **X** = $\underline{v}$mnm^{5}U	49±1

[a] Calculated yield from the chromatographic peak of the product based on the total area of the initial components (Figure S48).

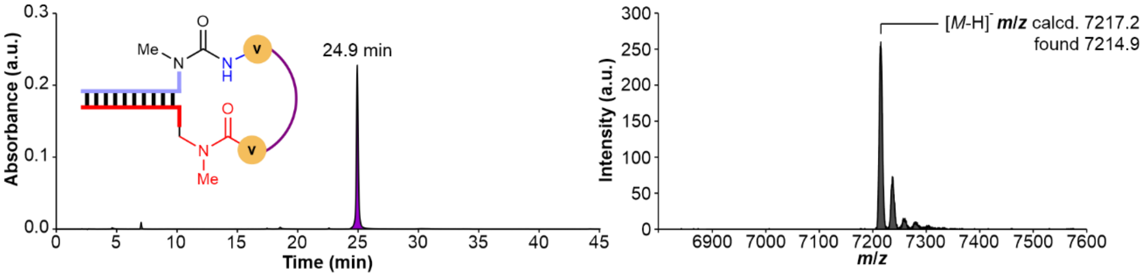

Figure S50. left) HPL-chromatogram and right) MALDI-TOF mass spectrum (negative mode) of the isolated product from the reaction of **ON1n**; **X** = m$^6\underline{v}^6$A with **ON2c**; **X** = $\underline{v}$mnm^{5}U.

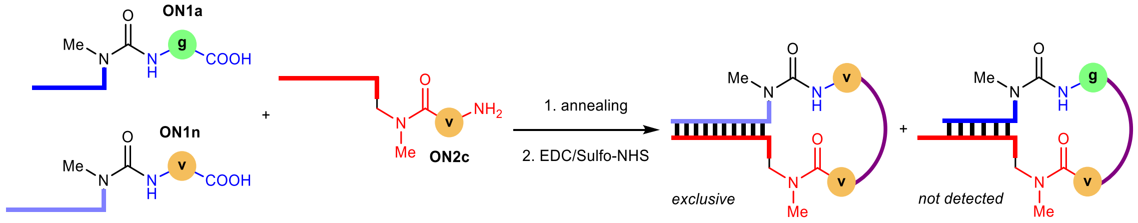

Scheme S13. Annealing and coupling reaction of **ON1a**; **X** = m$^6\underline{g}^6$A, **ON1n**; **X** = m$^6\underline{v}^6$A and **ON2c**; **X** = $\underline{v}$mnm^{5}U. The formed peptide bonds are marked in purple.

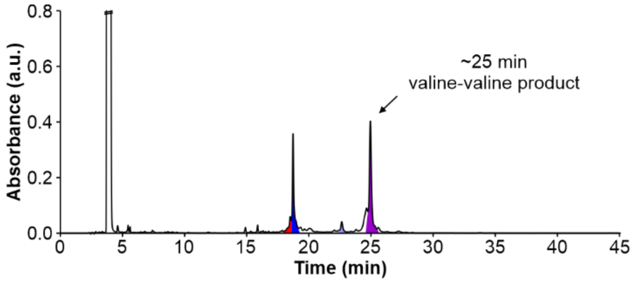

Figure S51. HPL-chromatogram of the reaction of **ON1a**; **X** = m$^6\underline{g}^6$A, **ON1n**; **X** = m$^6\underline{v}^6$A and **ON2c**; **X** = $\underline{v}$mnm^{5}U in MES buffer at pH 6 using EDC/Sulfo-NHS as activator.

Table S25. Results obtained in the coupling reaction of **ON1a**; **X** = m$^6\underline{g}^6$A, **ON1n**; **X** = m$^6\underline{v}^6$A and **ON2c**; **X** = $\underline{v}$mnm^{5}U using EDC/Sulfo-NHS as activator (average of, at least, two experiments).

Donor strand	Acceptor strand	Average Yield ± Error of $\underline{vv}$-peptide (%)[a]	Yield of $\underline{gv}$-peptide (%)[a]
**ON1a**; **X** = m$^6\underline{g}^6$A<br>**ON1n**; **X** = m$^6\underline{v}^6$A	**ON2c**; **X** = $\underline{v}$mnm^{5}U	49±2	not detected

[a] Calculated yield from the chromatographic peak of the product based on the total area of the initial components (Figure S48).

13. Stability of selected acceptor oligonucleotides (ON2)

The oligonucleotide (0.5 nmol) was added to an Eppendorf tube. Buffer, NaCl and water were added to the ON's solution and the reaction was heated in a Thermocycler.

Concentration of the components in the reaction mixture: 10-50 µM of oligonucleotide, 100 mM of buffer and 100 mM of NaCl (see figure footnotes for details).

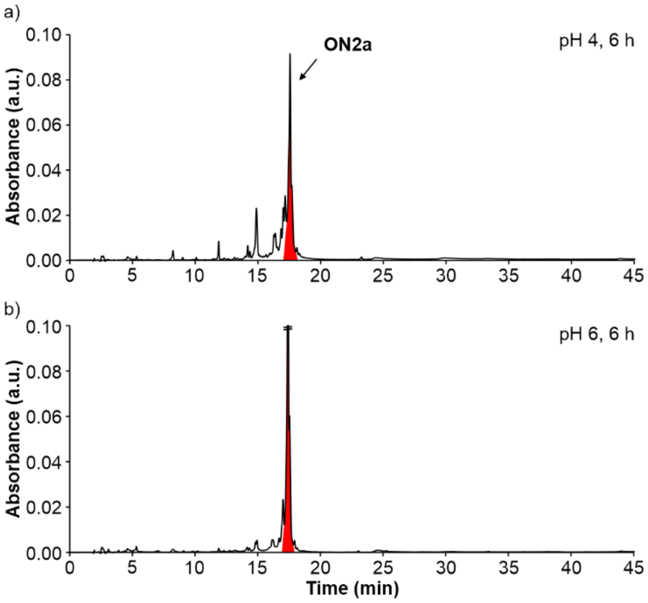

Figure S52. HPL-chromatograms of the stability of **ON2a**; **X** = mnm^{5}U in: a) acetate buffer at pH 4 and b) MES buffer at pH 6 after 6 h at 90°C.

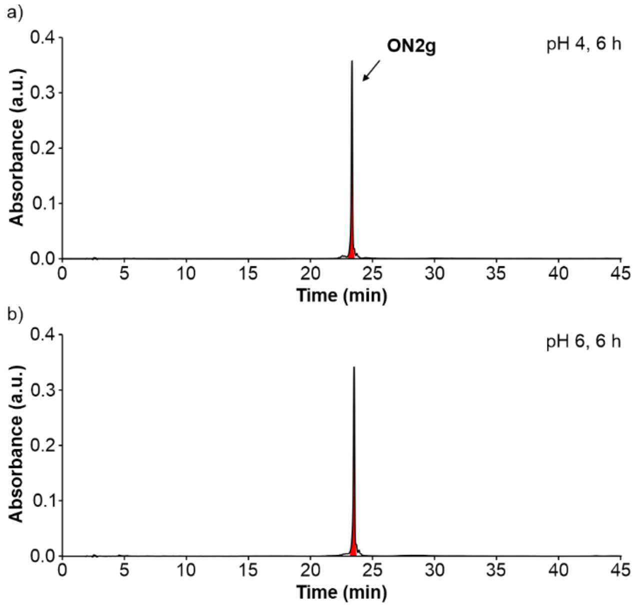

Figure S53. HPL-chromatograms of the stability of **ON2g**; **X** = mnm^{5}U in: a) acetate buffer at pH 4 and b) MES buffer at pH 6 after 6 h at 90°C.

Table S26. Results obtained in the stability of **ON2a** and **ON2g** (average of, at least, two experiments).[a]

		Average Amount ± Error (%)	
**pH**	**Time (h)**	**ON2a**	**ON2g**
4	6	40±3	>95
6	6	70±5	>95

[a] Calculated amounts from the chromatographic peaks using the corresponding calibration curves.

14. Cleavage of urea in selected oligonucleotides and cyclic peptide products

The cleavage reactions were carried out under identical conditions to those described in Section 13.

14.1 Cleavage reactions of ON1c (m^6v^6A) and ON1k (v^6A) at pH 5

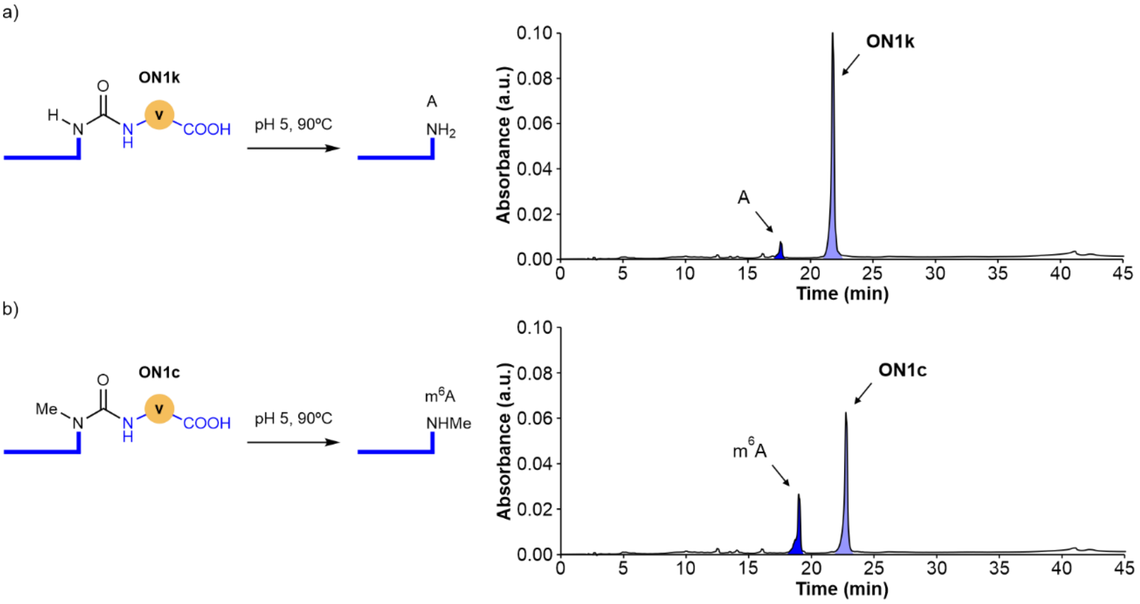

Figure S54. HPL-chromatograms of the cleavage reactions of: a) **ON1k**; **X** = $\underline{v}^6$A and b) **ON1c**; **X** = $m^6\underline{v}^6$A in acetate buffer at pH 5 after 12 h at 90°C.

These experiments indicated that the urea cleavage reaction of the unmethylated $\underline{aa}^6$A-RNA donor strand **ON1k** was slower than that of the methylated version, $m^6\underline{aa}^6$A-RNA **ON1c**.

Table S27. Results obtained in the cleavage reactions of **ON1c** and **ON1k** (average of, at least, two experiments).[a]

		**Average Amount ± Error (%)**	
**pH**	**Time (h)**	**ON1k**	**A-strand**
5	12	85±3	10±1
		**ON1c**	**m^6A-strand**
		65±1	20±1

[a] Calculated amounts from the chromatographic peaks using the corresponding calibration curves.

14.2 Cleavage reaction of ON3a (m^6g^6A coupled with mnm^5U)

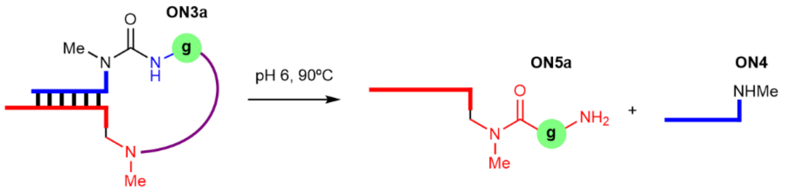

Scheme S14. Cleavage of urea in **ON3a**. The peptide bond is marked in purple.

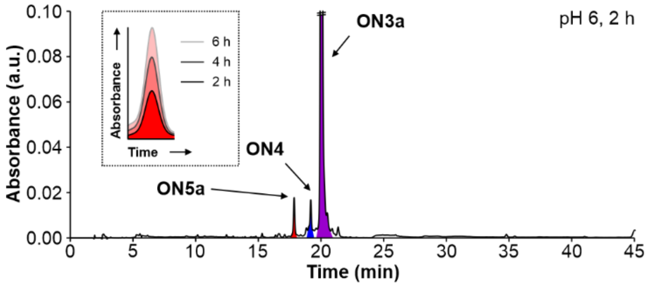

Figure S55. HPL-chromatogram of the cleavage reaction of **ON3a** in MES buffer at pH 6 after 2 h at 90°C. Inset shows the selected region of the HPL-chromatograms after 2, 4 and 6 h.

Table S28. Results obtained in the cleavage reaction of **ON3a** (average of, at least, two experiments).[a]

		Average Amount ± Error (%)		
**pH**	**Time (h)**	**ON3a**	**ON4 (m^6A)**	**ON5a ($\underline{g}mnm^5U$)**
6	6	75±2	15±1	15±1 (t_R = 17.5 min)

[a] Calculated amounts from the chromatographic peaks using the corresponding calibration curves.

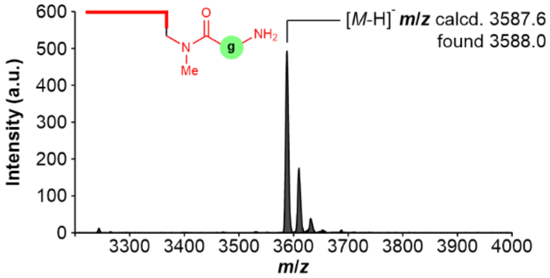

Figure S56. MALDI-TOF mass spectrum (negative mode) of the isolated **ON5a** ($\underline{g}mnm^5U$).

Additional experiments at pH 4 and pH 6

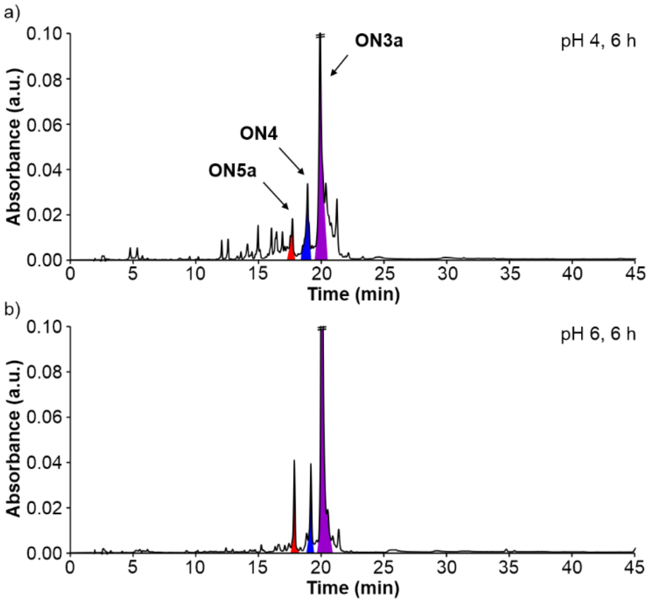

Figure S57. HPL-chromatograms of the cleavage reactions of **ON3a** in: a) acetate buffer at pH 4 and b) MES buffer at pH 6 after 6 h at 90°C.

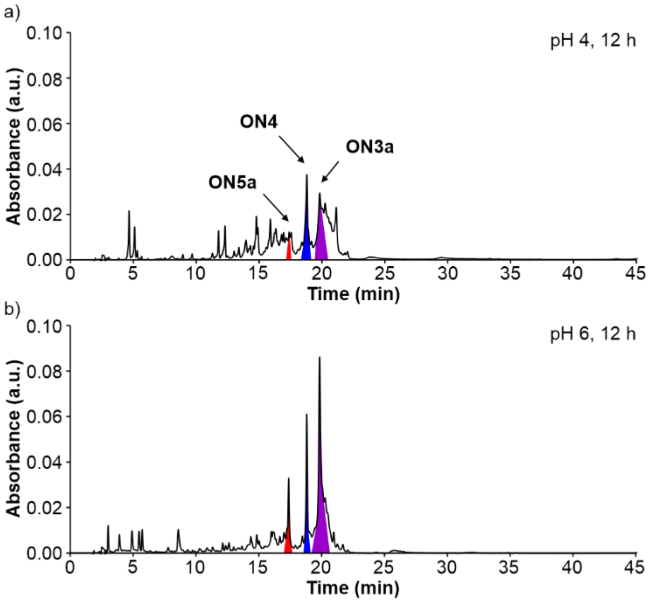

Figure S58. HPL-chromatograms of the cleavage reactions of **ON3a** in: a) acetate buffer at pH 4 and b) MES buffer at pH 6 after 12 h at 90°C.

Table S29. Results obtained in the cleavage reaction of **ON3a** (average of, at least, two experiments).[a]

		**Average Amount ± Error (%)**
**pH**	**Time (h)**	**ON5a (gmnm^{5}U)**
4	6	10±2
	12	n.d.
6	6	15±1
	12	10±1

[a] Calculated amounts from the chromatographic peak using the calibration curve of **CON2**. n.d. = not determined.

14.3 Cleavage reactions of ON3c (m^{6}g^{6}A coupled with vmnm^{5}U)

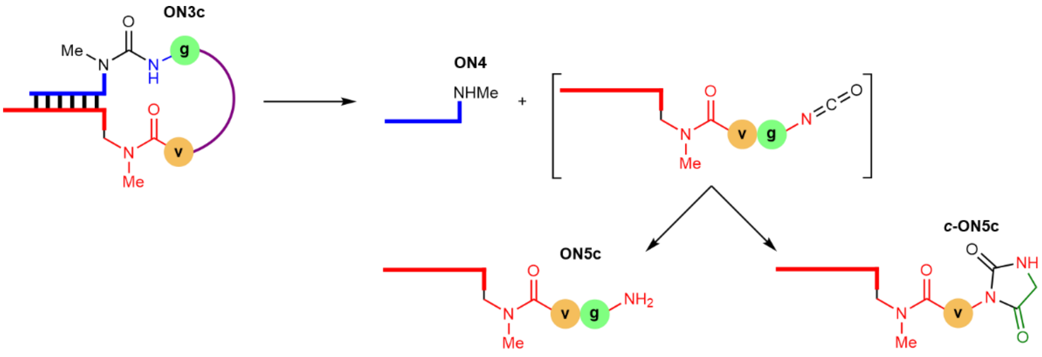

Figure S59. Cleavage of urea in **ON3c**. The peptide bond is marked in purple.

Cleavage reactions at 60°C

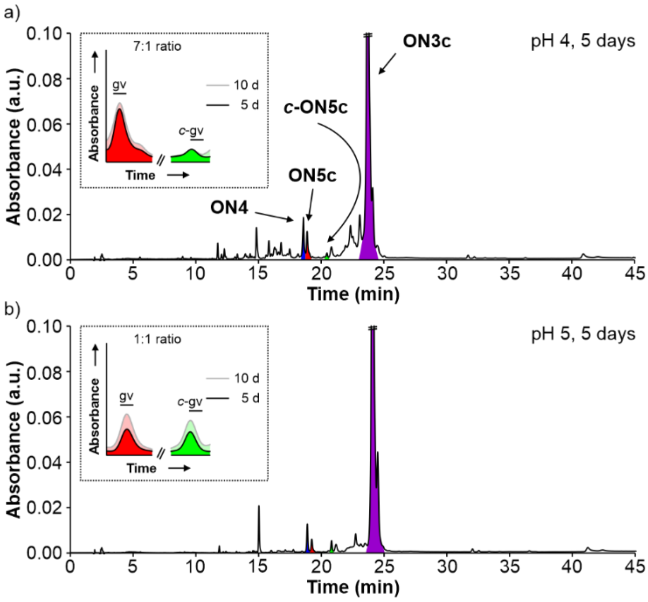

Figure S60. HPL-chromatograms of the cleavage reactions of **ON3c** in acetate buffer at: a) pH 4 and b) pH 5 after 5 days at 60°C. Inset shows the selected region of the HPL-chromatograms after 5 and 10 days.

Table S30. Results obtained in the cleavage reactions of **ON3c** at 60°C (average of, at least, two experiments).[a]

		Average Amount ± Error (%)				
**pH**	**Time (days)**	**ON3c**	**ON4 (m^{6}A)**	**ON5c (gvmnm^{5}U)**	**c-ON5c (c-gvmnm^{5}U)**	**Ratio (ON5c/c-ON5c)**
4	10	50±2	10.5±1	9±1 (t_R = 19.5 min)	1.5±0.5 (t_R = 21.0 min)	~7:1
5	10	80±3	6±1	3±1	3±1	~1:1

[a] Calculated amounts from the chromatographic peaks using the corresponding calibration curves.

Cleavage reactions at 90°C

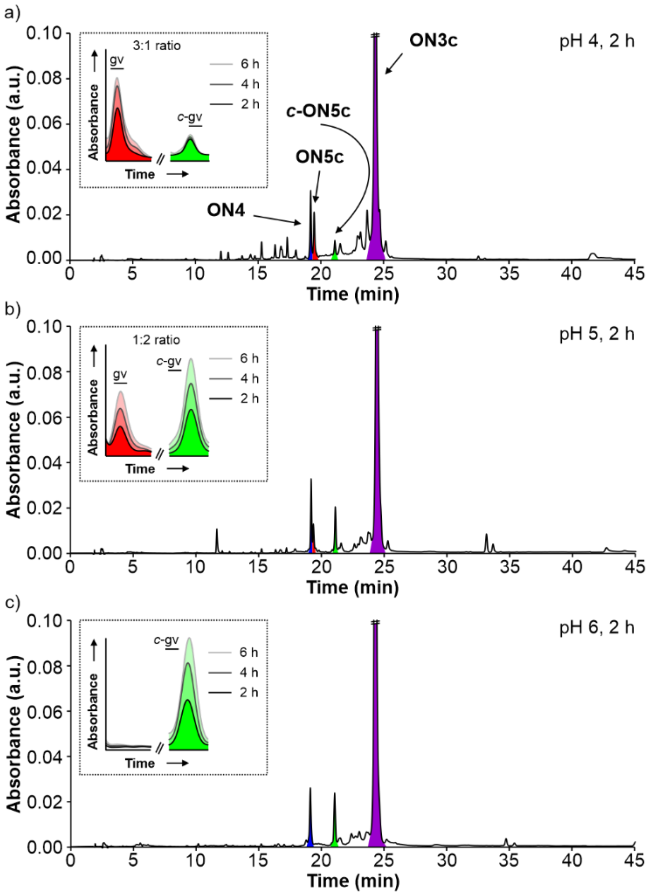

Figure S61. HPL-chromatograms of the cleavage reactions of **ON3c** in: a) acetate buffer at pH 4; b) acetate buffer at pH 5 and c) MES buffer at pH 6 after 2 h at 90°C. Inset shows the selected region of the HPL-chromatograms after 2, 4 and 6 h.

Table S31. Results obtained in the cleavage reactions of **ON3c** at 90°C (average of, at least, two experiments).[a]

		**Average Amount ± Error (%)**				
**pH**	**Time (h)**	**ON3c**	**ON4 (m^{6}A)**	**ON5c (gvmnm^{5}U)**	***c*-ON5c (*c*-gvmnm^{5}U)**	**Ratio (ON5c/*c*-ON5c)**
4	6	30±3	20±2	15±2 (t_R = 19.5 min)	5±1 (t_R = 21.0 min)	~3:1
5	6	55±3	25±2	8±1	17±3	~1:2
6	6	60±2	25±1	-	25±1	-

[a] Calculated amounts from the chromatographic peaks using the corresponding calibration curves.

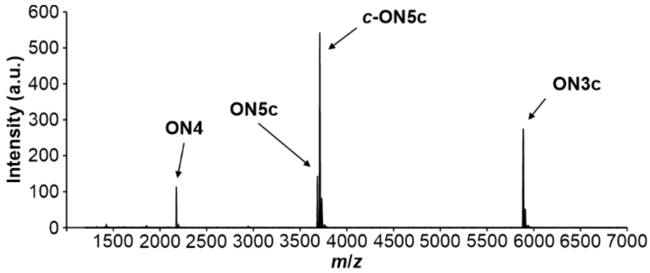

Figure S62. MALDI-TOF mass spectrum (negative mode) of the cleavage reaction of **ON3c** in acetate buffer at pH 5 after 2 h at 90°C. A similar MALDI-TOF mass spectrum was obtained at pH 4. The indicated peaks correspond to the $[M\text{-H}]^-$ ions.

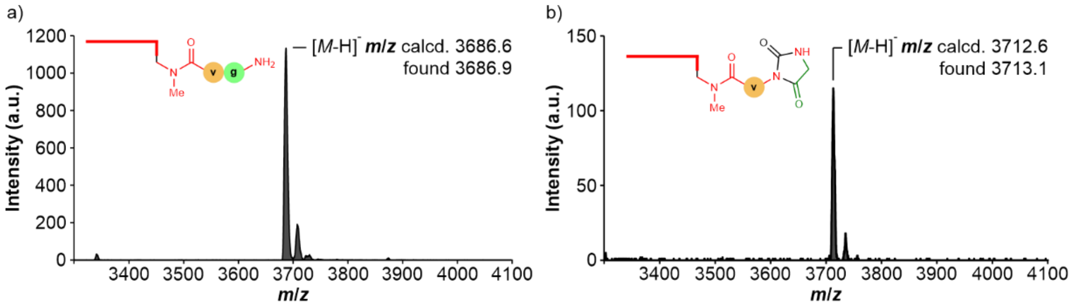

Figure S63. MALDI-TOF mass spectrum (negative mode) of the isolated: a) **ON5c** (gvmnm^{5}U) and b) ***c*-ON5c** (*c*-gvmnm^{5}U).

14.4 Cleavage reactions of peptide-oligonucleotides at pH 4

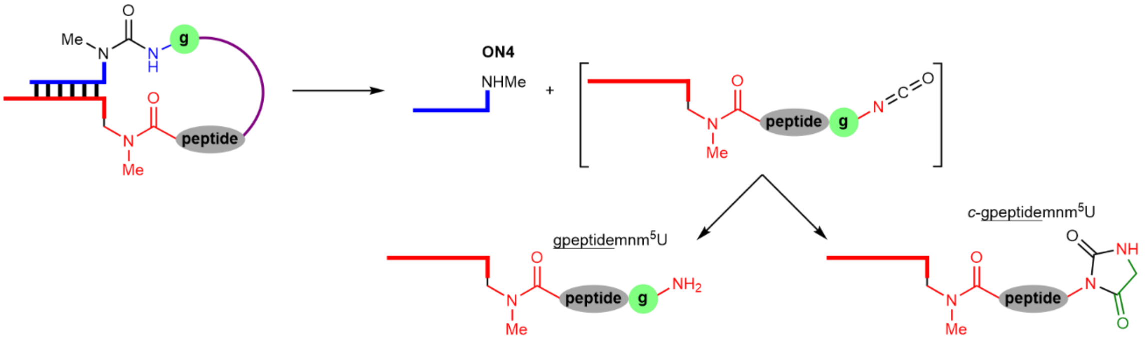

Scheme S15. Cleavage of urea in gpeptide-oligonucleotides. The peptide bond is marked in purple.

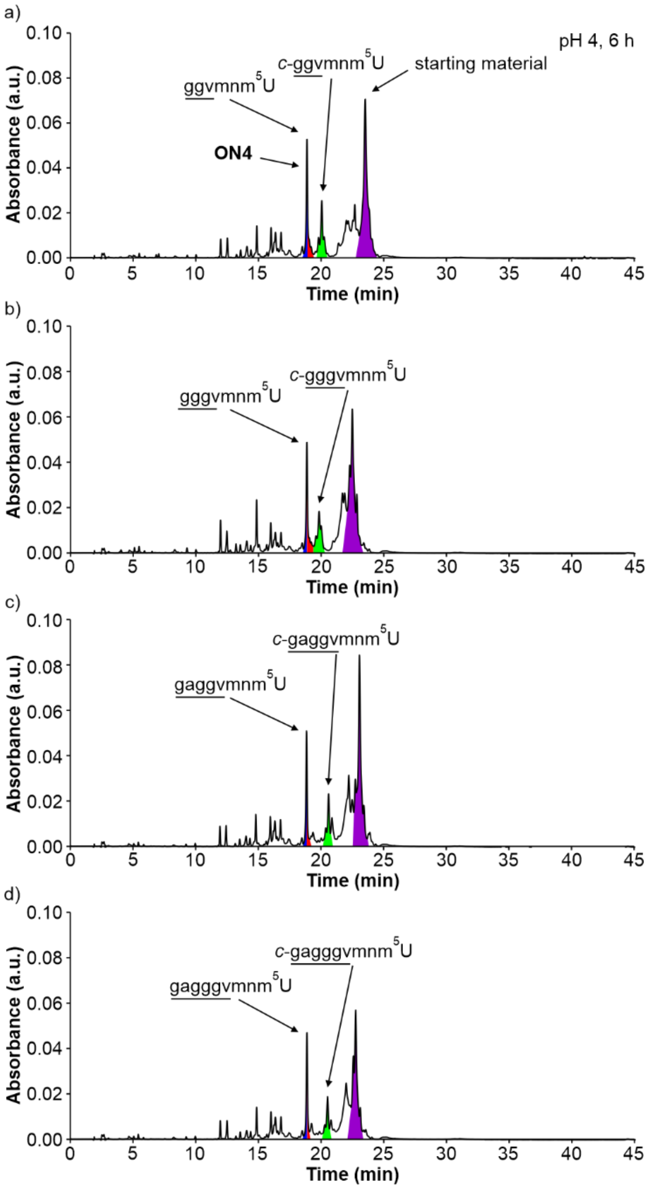

Figure S64. HPL-chromatograms of the cleavage reactions of peptide-oligonucleotides (Section 8.2) in acetate buffer at pH 4 to give: a) ggvmnm^{5}U; b) gggvmnm^{5}U; c) gaggvmnm^{5}U and d) gagggvmnm^{5}U oligonucleotides, together with hydantoin side products, after 6 h at 90°C.

The 3'-H_2N-peptidemnm^{5}U-RNA-5' and m^{6}A products overlap in the HPL-chromatograms. Therefore, they were isolated as a mixture in a single fraction.

Table S32. Results obtained in the cleavage reactions of peptide-oligonucleotides (Section 8.2).[a]

3'-H_2N-peptidemnm^{5}U-RNA-5'	Amount (%)
3'-ggvmnm^{5}U-RNA-5'	~12
3'-gggvmnm^{5}U-RNA-5'	~10
3'-gaggvmnm^{5}U-RNA-5'	~10
3'-gagggvmnm^{5}U-RNA-5'	~10

[a] Estimated amounts assuming that the 3'-H_2N-peptidemnm^{5}U-RNA-5' products and the hydantoin counterparts were formed in a similar extent.

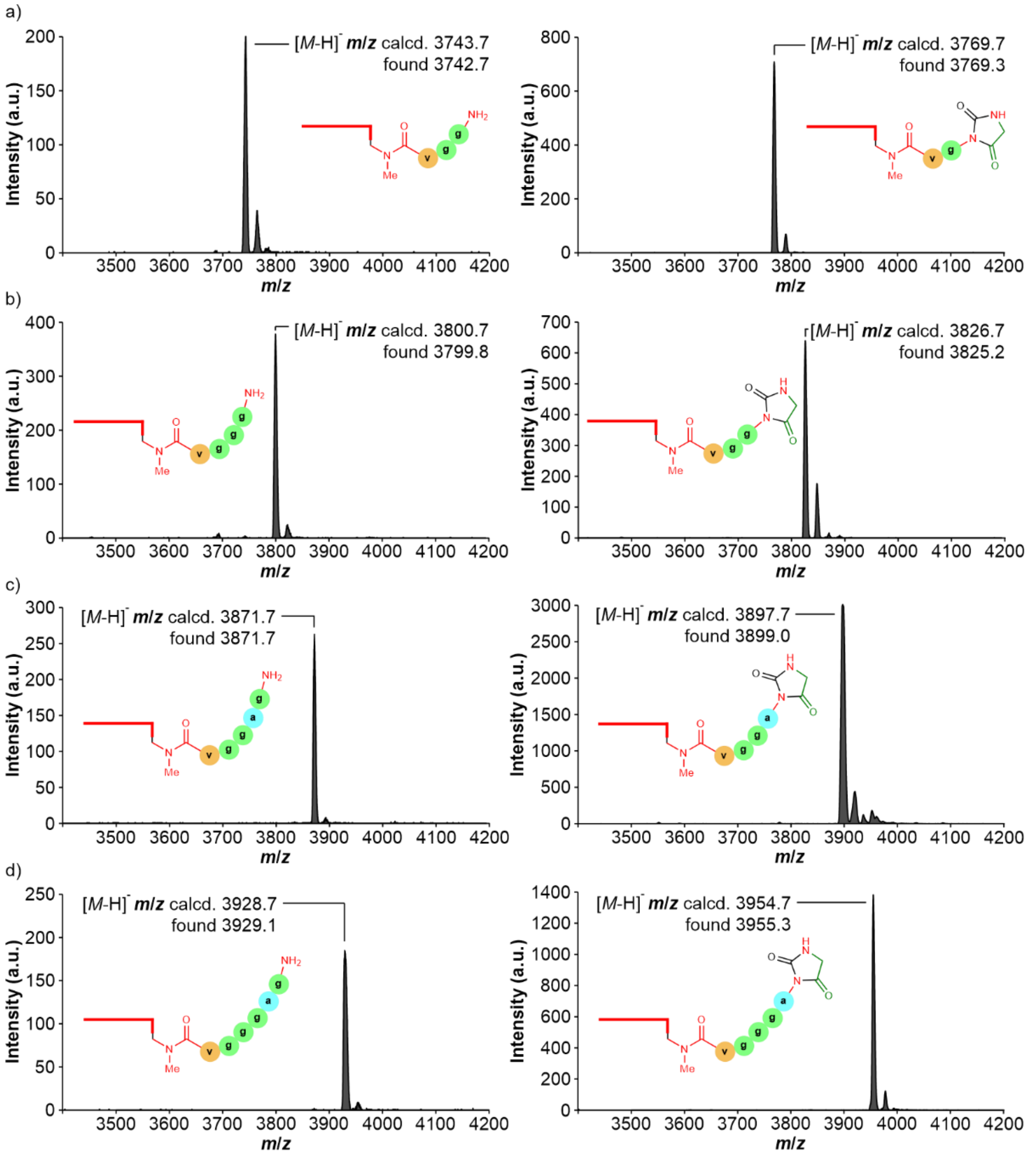

Figure S65. MALDI-TOF mass spectra (negative mode) of the isolated: a) ggvmnm^{5}U; b) gggvmnm^{5}U; c) gaggvmnm^{5}U and d) gagggvmnm^{5}U oligonucleotides (left) and hydantoin side products (right). Note that the analyzed 3'-H_2N-peptidemnm^{5}U-RNA-5' samples (left) contained the m^{6}A product (*m/z* region not shown).

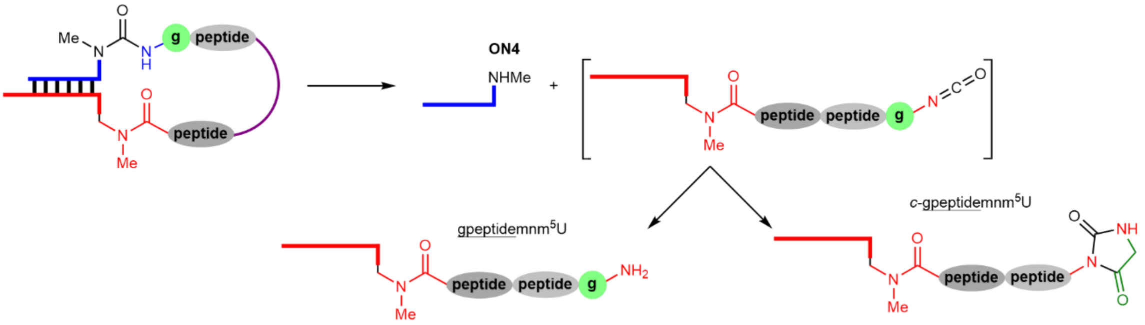

Figure S66. Cleavage of urea in gpeptide-peptidemnm^{5}U-oligonucleotides. The peptide bond is marked in purple.

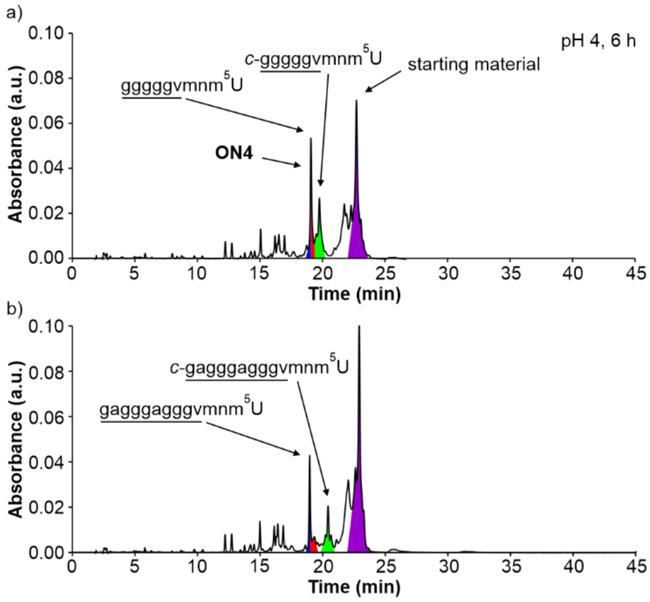

Figure S67. HPL-chromatograms of the cleavage reactions of peptide-oligonucleotides (Section 8.3) in acetate buffer at pH 4 to give: a) gggggvmnm^{5}U and b) gagggagggvmnm^{5}U oligonucleotides, together with hydantoin side products, after 6 h at 90°C.

The 3'-H_2N-peptidemnm^{5}U-RNA-5' and m^{6}A products overlap in the HPL-chromatograms. In addition, the 3'-H_2N-peptidemnm^{5}U-RNA-5' in a) overlaps with the hydantoin side product.

Table S33. Results obtained in the cleavage reactions of peptide-oligonucleotides (Section 8.3).[a]

3'-H_2N-peptidemnm^{5}U-RNA-5'	Amount (%)
3'-gggggvmnm^{5}U-RNA-5'	~10
3'-gagggagggvmnm^{5}U-RNA-5'	~9

[a] Estimated amounts assuming that the 3'-H_2N-peptidemnm^{5}U-RNA-5' products and the hydantoin counterparts were formed in a similar extent.

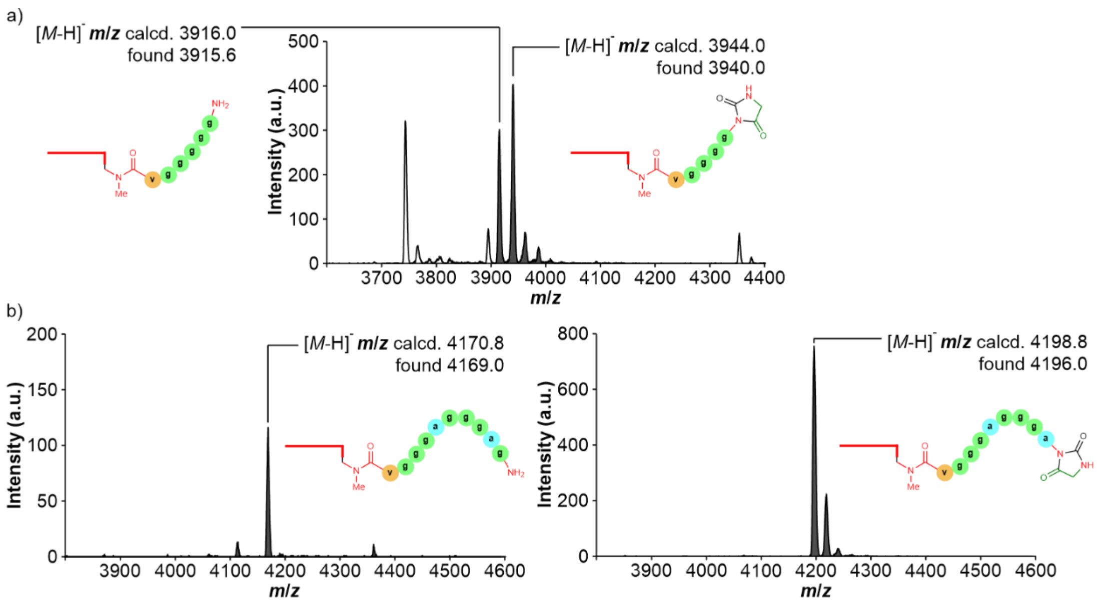

Figure S68. MALDI-TOF mass spectra (negative mode) of the isolated: a) ggggvmnm^{5}U and b) gaggagggvmnm^{5}U. Hydantoin side products are also shown. Note that the analyzed 3'-H_2N-peptidemnm^{5}U-RNA-5' samples contained the m^{6}A product (*m/z* region not shown).

15. Coupling and cleavage reactions between donor and acceptor oligonucleotides containing 2'-OMe nucleosides

The peptide coupling and urea cleavage reactions were carried out under identical conditions to those described in Section 0 and Section 13, respectively.

15.1 Coupling and cleavage reactions of ON1a (m^{6}g^{6}A) with ON2g

Each coupling reaction was performed using 1 equiv. of **ON1a** with respect to the acceptor oligonucleotide, **ON2g** or **ON5g**.

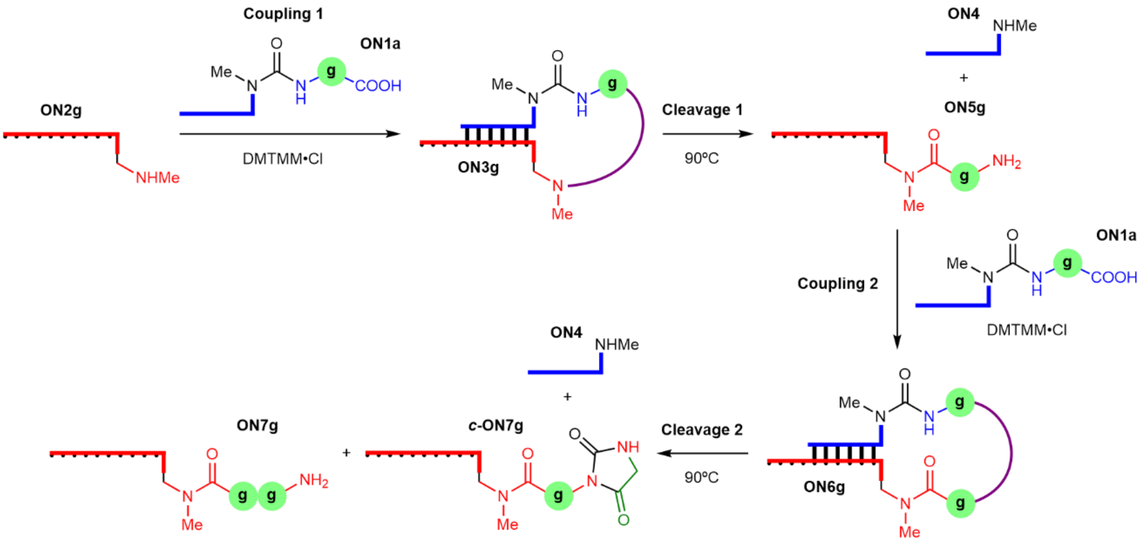

Scheme S16. Coupling and cleavage of **ON1a**; **X** = m^{6}g^{6}A with **ON2g**. The formed peptide bond is marked in purple.

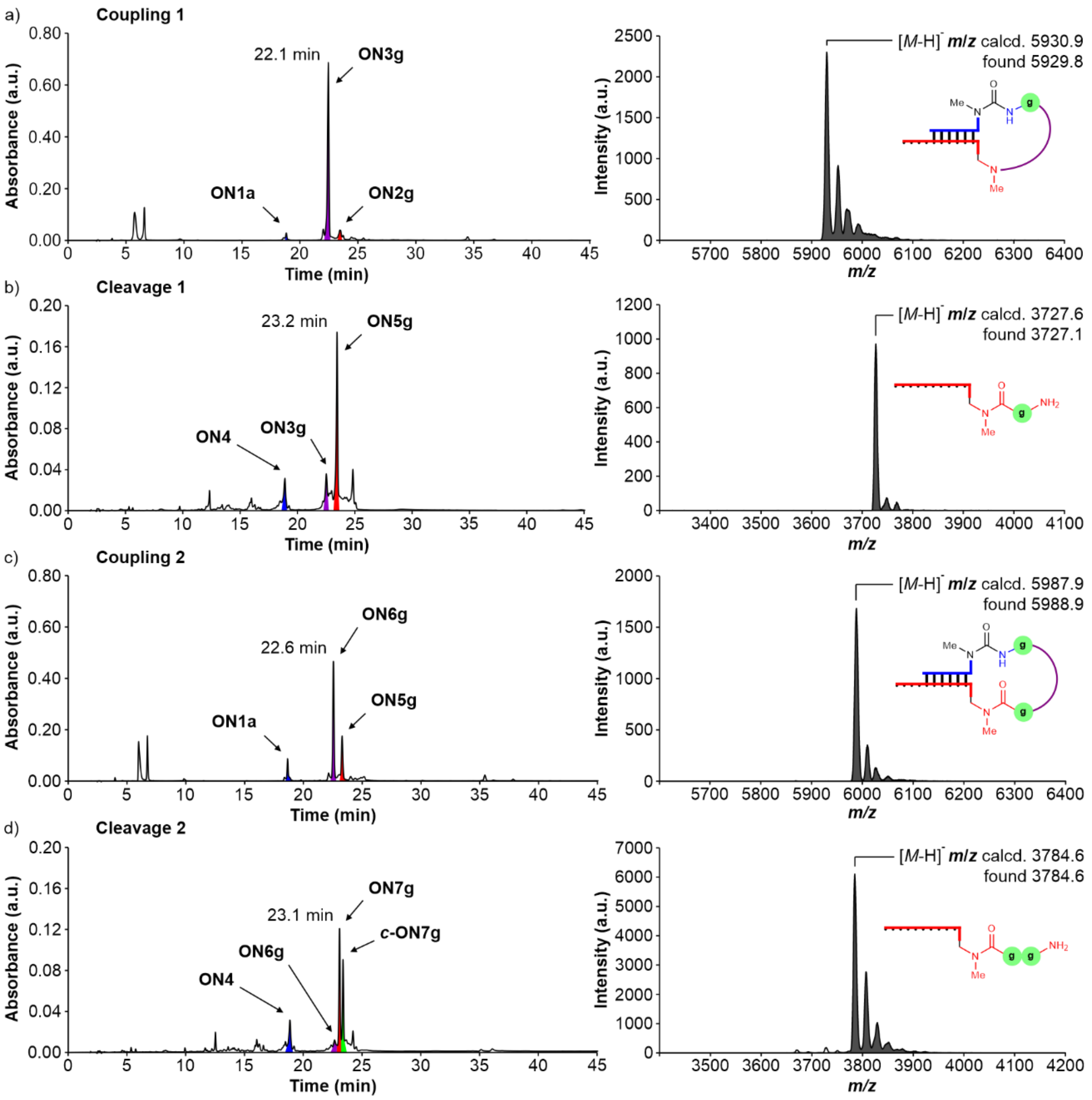

Figure S69. left) HPL-chromatograms of the reactions of **ON1a**; **X** = m^{6}g^{6}A with **ON2g**: a) coupling 1; b) cleavage 1; c) coupling 2 and d) cleavage 2. The product of each step was separated by HPLC and added into the next reaction. right) MALDI-TOF mass spectra (negative mode) of the isolated products from the reactions a)-d).

Table S34. Results obtained in the coupling and cleavage reactions of **ON1a**; **X** = m^{6}g^{6}A with **ON2g**.

Steps	Activators	pH	T (°C)	Time (h)	Yield (%)[a]
Coupling 1 (**ON3g**)	EDC/Sulfo-NHS	6	25	24	~39
	DMTMM•Cl	6	25	24	~69
Cleavage 1 (**ON5g**)	-	4	90	24	60
	-	6	90	24	46
Coupling 2 (**ON6g**)	DMTMM•Cl	6	25	24	~42
Cleavage 2 (**ON7g**)	-	4	90	24	34

[a] Calculated/estimated amounts from the chromatographic peaks using the corresponding calibration curves.

One pot reaction

The one pot reaction was performed with 15 nmol of **ON2g** as starting acceptor strand. 15 nmol of donor strand **ON1a** or **ON1g** were added for each coupling reaction. After each coupling reaction and the second cleavage, the crude was filtered using an Amicon® ultra centrifugal filter (3 kDa Nominal Molecular Weight Cut-Off) to remove the remaining activator and exchange the buffer solution. The volume of the solution was maintained constant throughout the five reaction steps. 20 µL of the crude (1 nmol) were analyzed by HPLC after the second coupling, the second cleavage and the third coupling reactions.

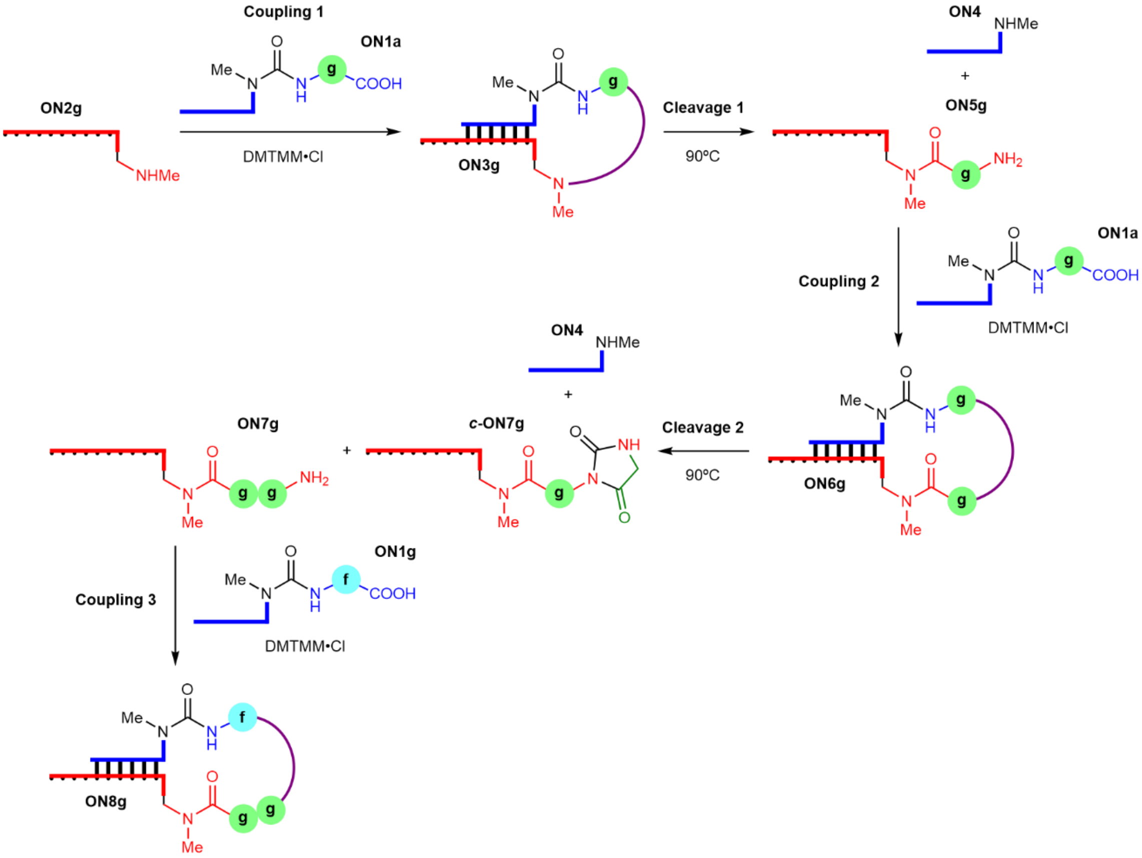

Figure S70. Coupling and cleavage of **ON1a**; **X** = m^{6}g^{6}A and **ON1g**; **X** = m^{6}f^{6}A with **ON2g**. The formed peptide bond is marked in purple.

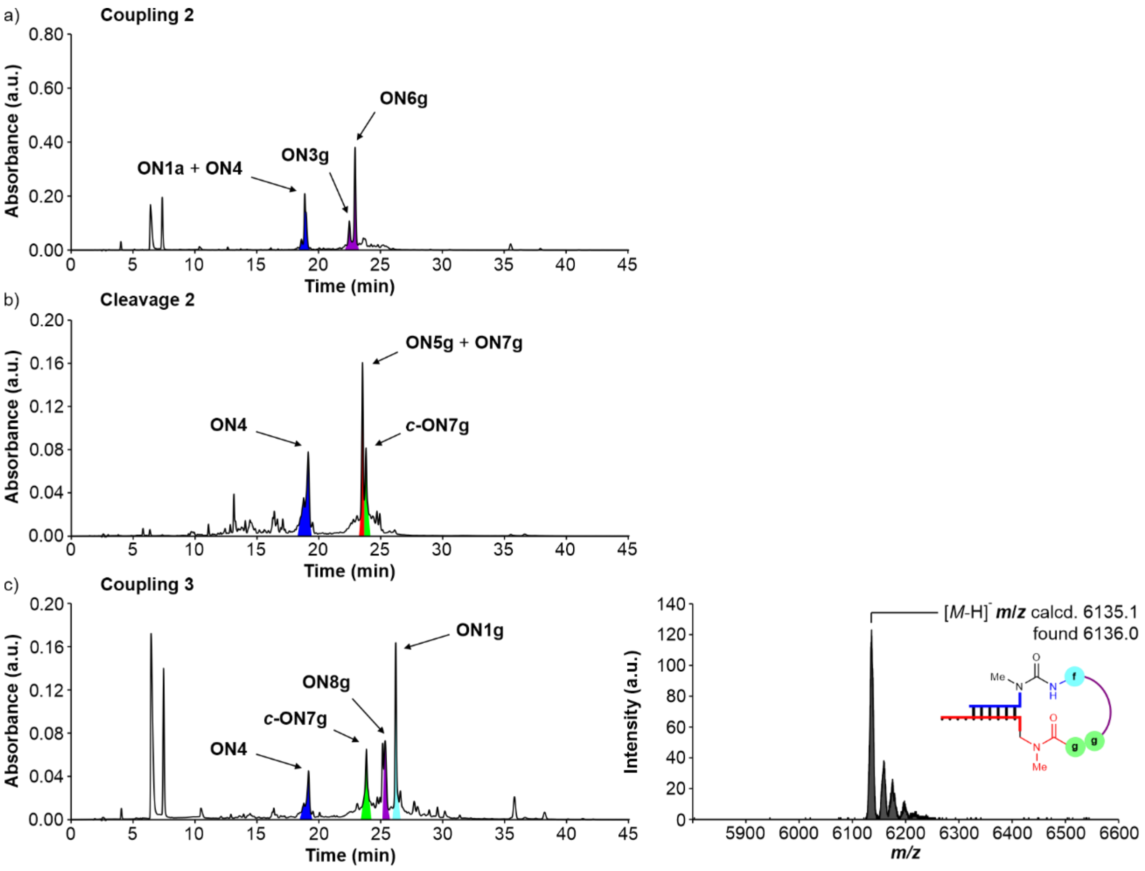

Figure S71. HPL-chromatograms of the one pot reaction of **ON1a**; **X** = m^{6}g^{6}A and **ON1g**; **X** = m^{6}f^{6}A with **ON2g**: a) coupling 2; b) cleavage 2 and c) coupling 3.

Table S35. Results obtained in the one pot reaction of **ON1a**; **X** = m^{6}g^{6}A and **ON1g**; **X** = m^{6}f^{6}A with **ON2g**.

Steps	Activators	pH	T (°C)	Time (h)	Yield (%)[a]
Coupling 2 (**ON6g**)	DMTMM•Cl	6	25	24	~36 in three steps
Cleavage 2 (**ON5g** + **ON7g**)	-	4	90	24	23 in four steps
Coupling 3 (**ON8g**)	DMTMM•Cl	6	25	24	~10 in five steps

[a] Calculated/estimated amounts from the chromatographic peaks using the corresponding calibration curves.

15.2 Coupling and cleavage reactions of ON1o (m^{6}g^{6}Am) with ON2h

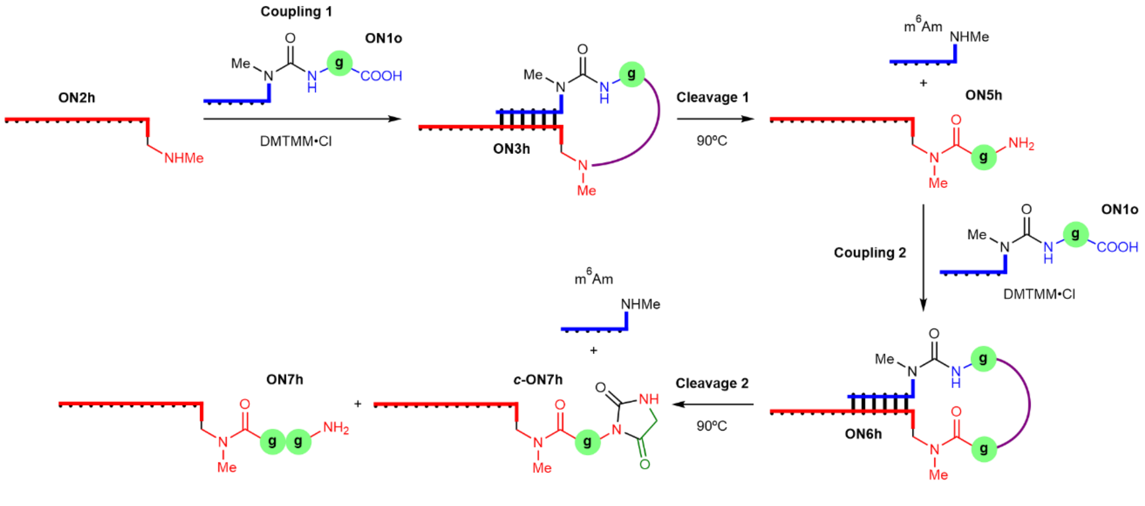

Figure S72. Coupling and cleavage of **ON1o**; **X** = m^{6}g^{6}Am with **ON2h**. The formed peptide bond is marked in purple.

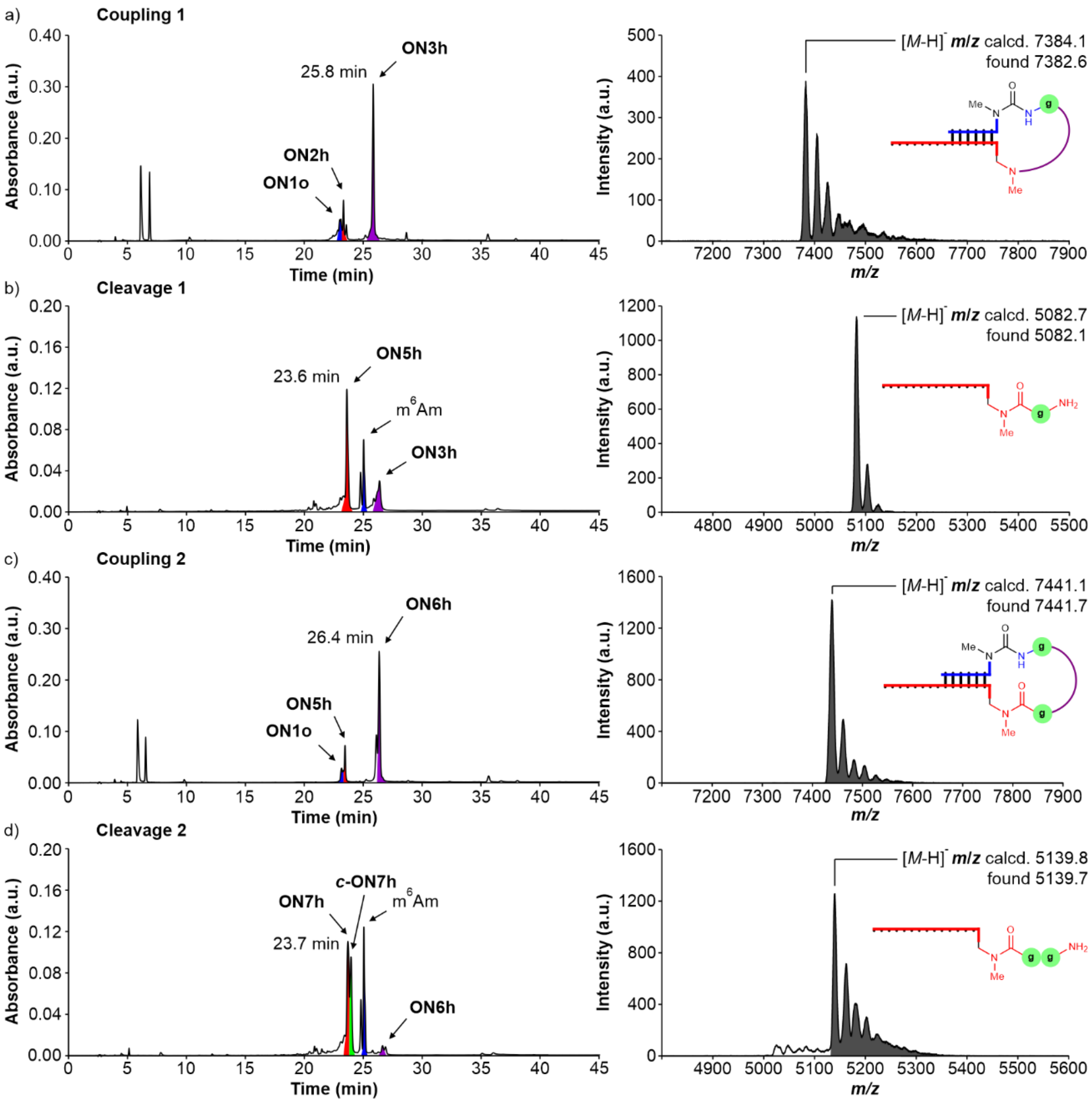

Figure S73. left) HPL-chromatograms of the reactions of **ON1o**; **X** = m$^6\underline{g}^6$Am with **ON2h**: a) coupling 1; b) cleavage 1; c) coupling 2 and d) cleavage 2. The product of each step was separated by HPLC and added into the next reaction. right) MALDI-TOF mass spectra (negative mode) of the isolated products from the reactions a)-d).

Table S36. Results obtained in the coupling and cleavage reactions of **ON1o**; **X** = m$^6\underline{g}^6$Am with **ON2h**.

**Steps**	**Activators**	**pH**	**T (°C)**	**Time (h)**	**Yield (%)[a]**
Coupling 1 (**ON3h**)	DMTMM•Cl	6	25	24	46
Cleavage 1 (**ON5h**)	-	4	90	48	30
Coupling 2 (**ON6h**)	DMTMM•Cl	6	25	24	41
Cleavage 2 (**ON7h**)	-	4	90	48	28

[a] Calculated amounts from the chromatographic peaks using the corresponding calibration curves.

15.3 Coupling and cleavage reactions of donor and acceptor-peptide oligonucleotides

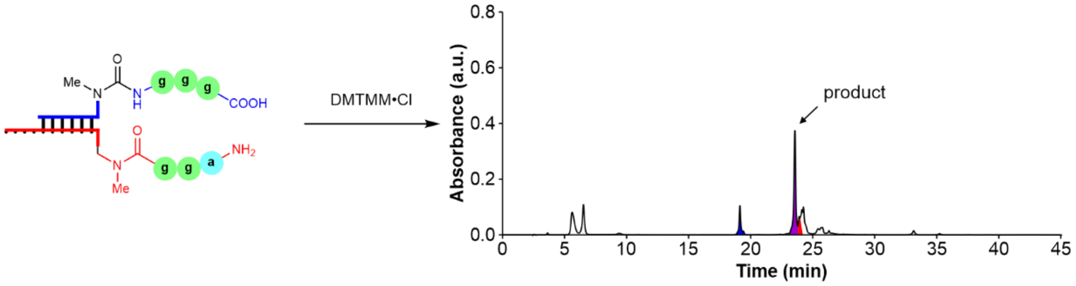

Figure S74. HPL-chromatogram of the reaction of 5'-m^6(ggg)^{6}A-RNA-3' with 3'-aggmnm^{5}U-RNA-5' containing 2'-OMe nucleosides in MES buffer at pH 6 using DMTMM•Cl as activator.

Table S37. Result obtained in the coupling reaction of peptide-modified donor and acceptor oligonucleotides using DMTMM•Cl as activator.

Donor strand	Acceptor strand	Yield (%)[a]
5'-m^6(ggg)^{6}A-RNA-3'	3'-aggmnm^{5}U-RNA-5'	~50

[a] Estimated yield from the chromatographic peak of the product using the calibration curve of **CON3**.

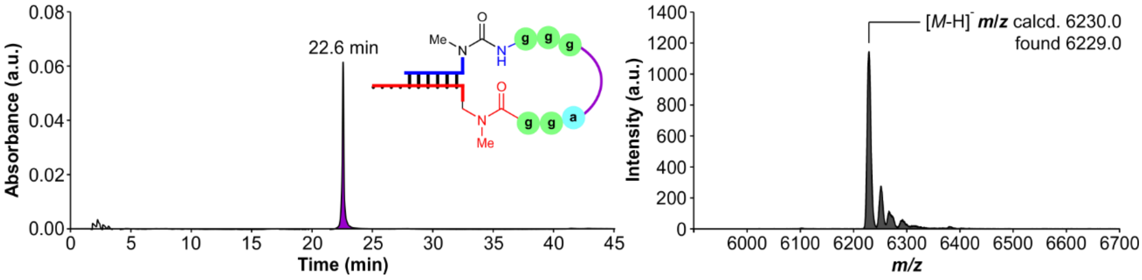

Figure S75. left) HPL-chromatogram and right) MALDI-TOF mass spectrum (negative mode) of the isolated product from the reaction of 5'-m^6(ggg)^{6}A-RNA-3' with 3'-aggmnm^{5}U-RNA-5' containing 2'-OMe nucleosides.

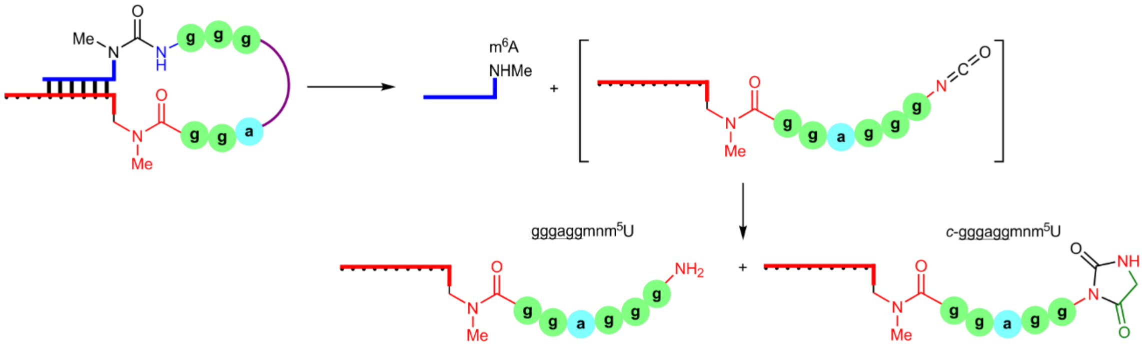

Scheme S17. Cleavage of urea in peptide-peptidemnm^{5}U-oligonucleotide. The peptide bond is marked in purple.

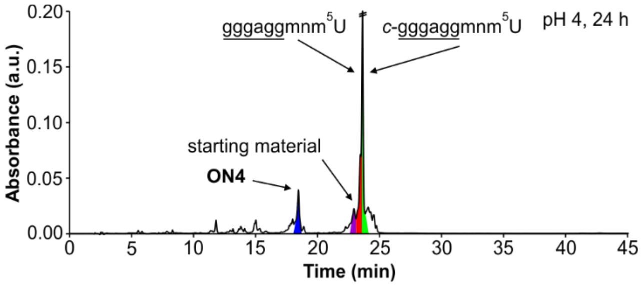

Figure S76. HPL-chromatogram of the cleavage reaction of peptide-oligonucleotide in acetate buffer at pH 4 to give gggaggmnm^{5}U and *c*-gggaggmnm^{5}U oligonucleotides after 24 h at 90°C.

The 3'-H_2N-peptidemnm^{5}U-RNA-5' and hydantoin side products overlap in the HPL-chromatogram.

Table S38. Result obtained in the cleavage reaction of peptide-oligonucleotide.[a]

Product oligonucleotides containing 2'-OMe nucleosides	Amount (%)
3'-gggaggmnm^{5}U-RNA-5' and 3'-*c*-gggaggmnm^{5}U-RNA-5'	~85 (t_R = 23.6 min)

[a] Estimated amount from the chromatographic peak using the calibration curve of **CON2**.

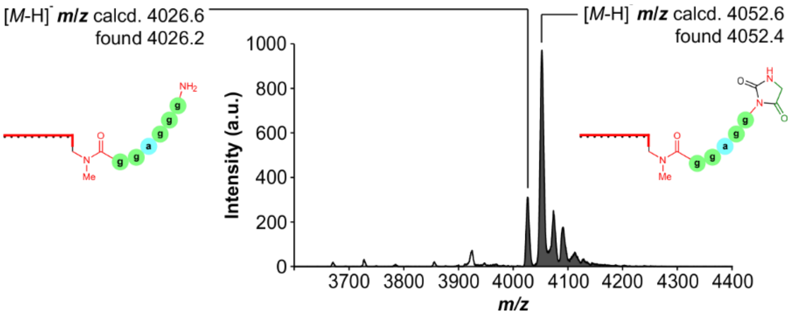

Figure S77. MALDI-TOF mass spectra (negative mode) of the isolated gggaggmnm^{5}U and hydantoin side product.

15.4 Coupling reactions between ON2g and donor oligonucleotides of different length

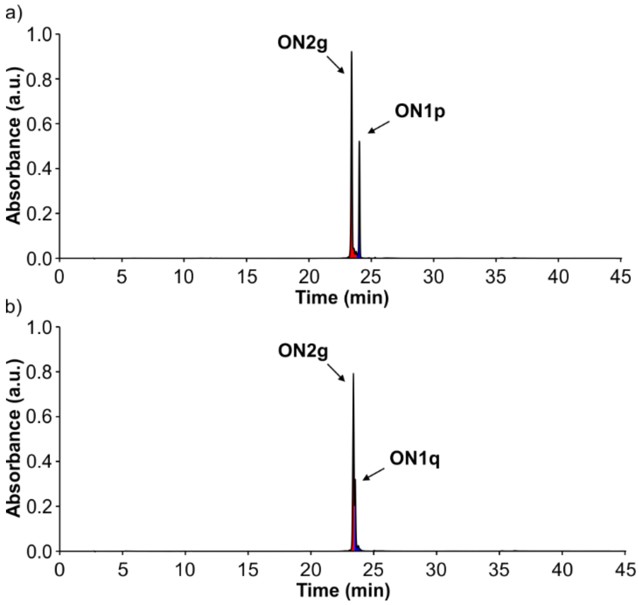

Figure S78. HPL-chromatograms of an equimolar mixture of **ON2g**; **X** = mnm^{5}U with: a) **ON1p**; **X** = m^{6}g^{6}Am and b) **ON1q**; **X** = m^{6}g^{6}Am.

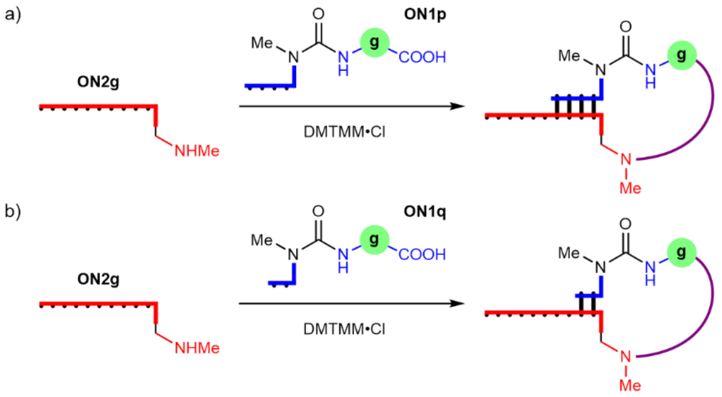

Scheme S18. Coupling of **ON2g**; **X** = mnm^{5}U with: a) **ON1p**; **X** = m^{6}g^{6}Am and b) **ON1q**; **X** = m^{6}g^{6}Am. The formed peptide bond is marked in purple.

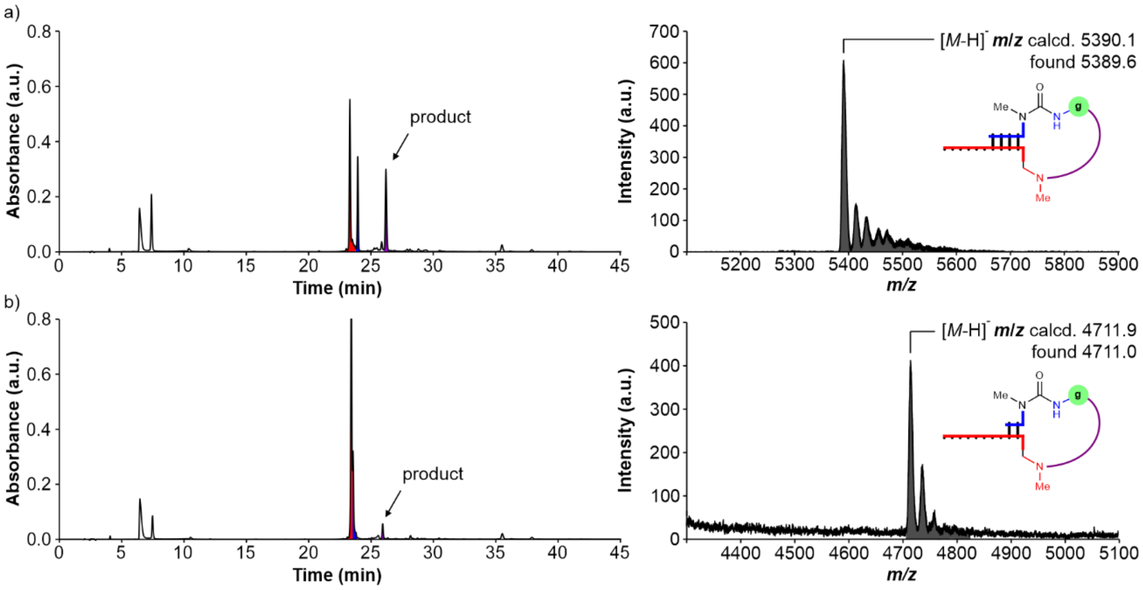

Figure S79. left) HPL-chromatograms of the reactions of: a) **ON1p**; **X** = m^{6}g^{6}Am and b) **ON1q**; **X** = m^{6}g^{6}Am with **ON2g**; **X** = mnm^{5}U in MES buffer at pH 6 using DMTMM•Cl as activator. The reaction b) was carried out at 0°C using 1 M NaCl. right) MALDI-TOF mass spectra (negative mode) of the isolated products.

Table S39. Results obtained in the coupling reactions of **ON2g**; **X** = mnm^{5}U with **ON1p**; **X** = m^{6}g^{6}Am or **ON1q**; **X** = m^{6}g^{6}Am using DMTMM•Cl as activator (average of, at least, two experiments).

**Donor strand**	**Acceptor strand**	**Average Yield ± Error (%)**[a]
**ON1p**; **X** = m^{6}g^{6}Am	**ON2g**; **X** = mnm^{5}U	19±2 (t_R = 26.2 min)
**ON1q**; **X** = m^{6}g^{6}Am		5±1[b] (t_R = 26.0 min)

[a] Calculated yield from the chromatographic peak of the product based on the total area of the initial components (Figure S78). [b] Using 1 M NaCl at 0°C.

16. Determination of melting temperatures by UV spectroscopic experiments

The UV melting curves were measured on a JASCO V-650 spectrometer at 260 nm using 10 mm QS cuvettes with a scanning rate of 1°C·min^{-1}. The obtained UV spectroscopic data were fit to the corresponding function to determine the melting temperature/s.

For double strands of non-self-complementary oligonucleotides, the data were fit to a two-state melting model, *i.e.* double strand – random coil equilibrium, using a mono-sigmoidal Boltzmann function.[14] On the contrary, the data were fit to a three-state melting model, *i.e.* double strand – hairpin – random coil equilibria, for single strands of self-complementary oligonucleotides using a double-sigmoidal Boltzmann function.[15,16]

For the experiments, we prepared aqueous solutions containing equimolar amounts of the oligonucleotides (5 μM), 10 mM phosphate buffer at pH 7 and 150 mM NaCl. The oligonucleotides were annealed by heating to 95°C for 4 min and, subsequently, by cooling down slowly to 5°C before the variable-temperature UV spectroscopic experiment.

16.1 Melting temperature of a double strand from canonical oligonucleotides

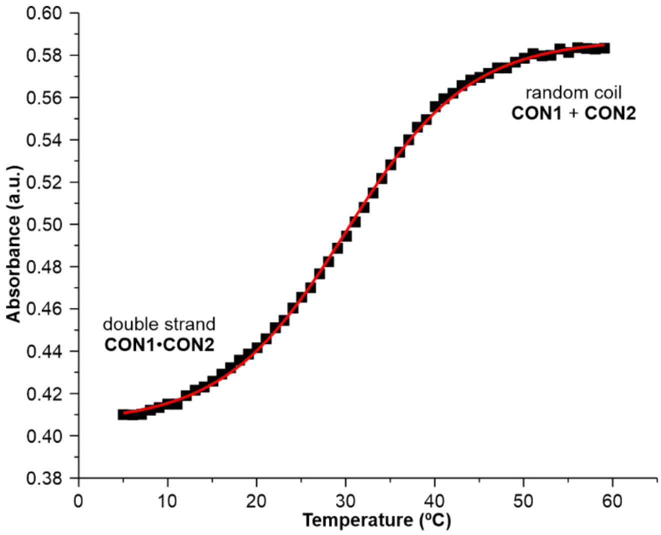

Figure S80. Melting curve of **CON1** and **CON2**. Line shows the fit of the data to a two-state melting model using a mono-sigmoidal Boltzmann function. T_m = 30.1°C.

16.2 Melting temperatures of double strands from donor and acceptor oligonucleotides

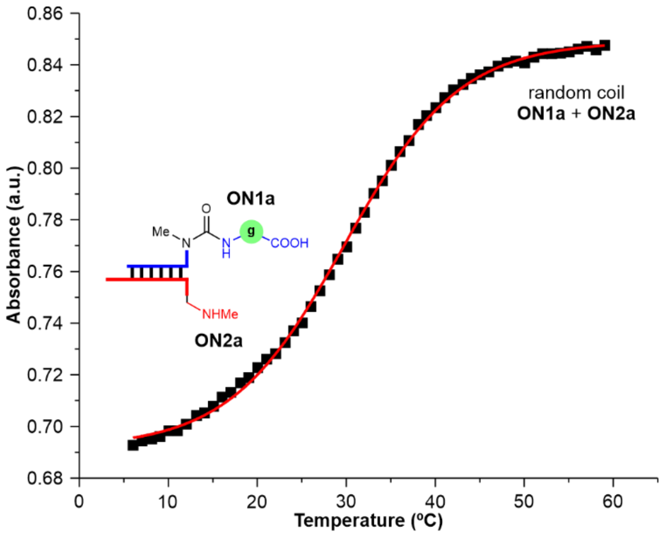

Figure S81. Melting curve of **ON1a**; **X** = m^{6}g^{6}A and **ON2a**; **X** = mnm^{5}U. Line shows the fit of the data to a two-state melting model using a mono-sigmoidal Boltzmann function. T_m = 30.4°C.

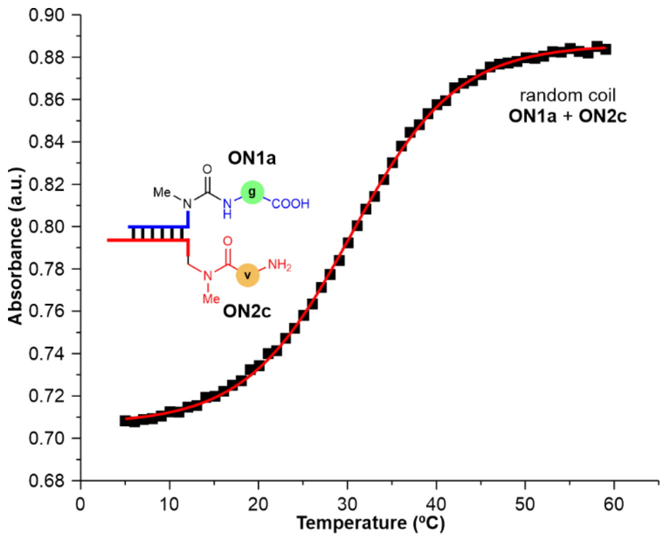

Figure S82. Melting curve of **ON1a**; **X** = m^{6}g^{6}A and **ON2c**; **X** = vmnm^{5}U. Line shows the fit of the data to a two-state melting model using a mono-sigmoidal Boltzmann function. T_m = 30.5°C.

The melting temperatures of the double strands containing modified A and U bases, **ON1a**; **X** = m^{6}g^{6}A, **ON2a**; **X** = mnm^{5}U and **ON2c**; **X** = vmnm^{5}U, were very similar to those determined for canonical oligonucleotides, **CON1** and **CON2**.

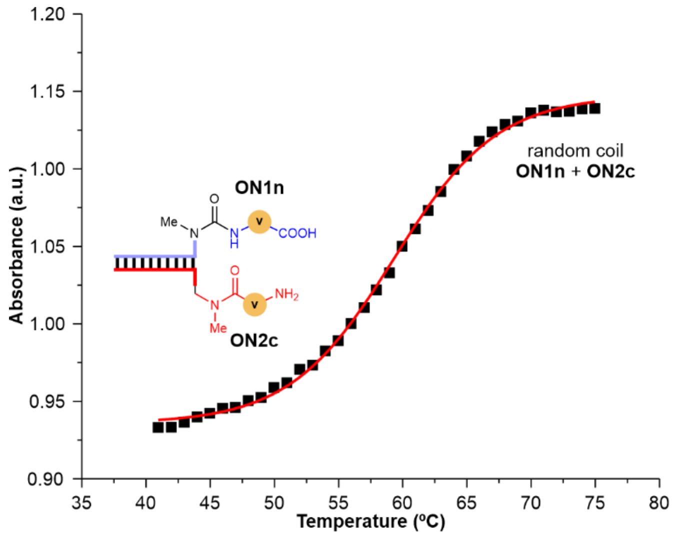

Figure S83. Melting curve of **ON1n**; **X** = m^{6}v^{6}A and **ON2c**; **X** = vmnm^{5}U. Line shows the fit of the data to a two-state melting model using a mono-sigmoidal Boltzmann function. T_m = 59.2°C.

16.3 Melting temperatures of double strands from donor and acceptor peptide-oligonucleotides

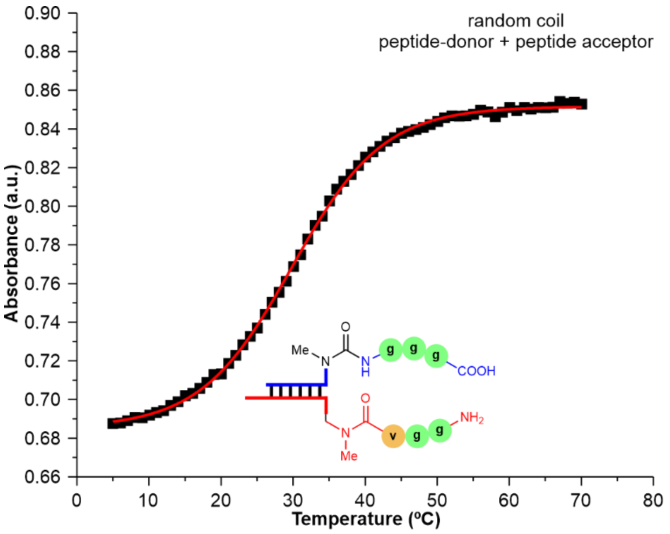

Figure S84. Melting curve of 5’-m^6(ggg)^{6}A-RNA-3’ with 3’-ggvmnm^{5}U-RNA-5’. Line shows the fit of the data to a two-state melting model using a mono-sigmoidal Boltzmann function. T_m = 30.0°C.

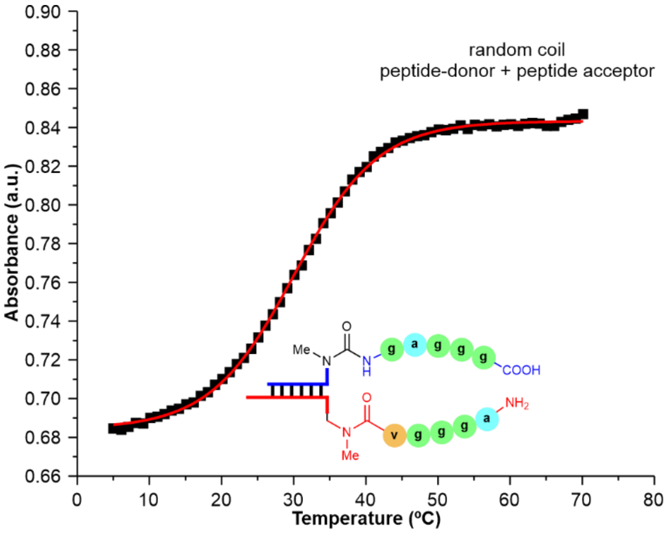

Figure S85. Melting curve of 5’-m^6(gaggg)^{6}A-RNA-3’ with 3’-agggvmnm^{5}U-RNA-5’. Line shows the fit of the data to a two-state melting model using a mono-sigmoidal Boltzmann function. T_m = 29.8°C.

16.4 Melting temperatures of selected cyclic peptide products

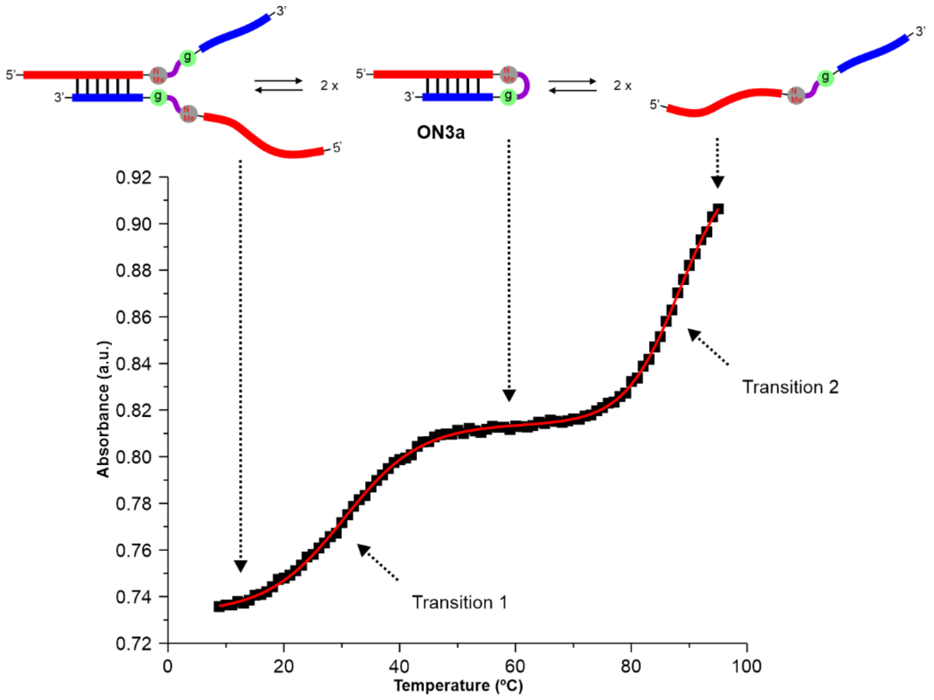

Figure S86. Melting curve of **ON3a**. Line shows the fit of the data to a three-state melting model using a double-sigmoidal Boltzmann function. T_{m1} = 30.8°C and T_{m2} = 87.5 °C. Top panel shows representation of the three states involved in the two transitions.

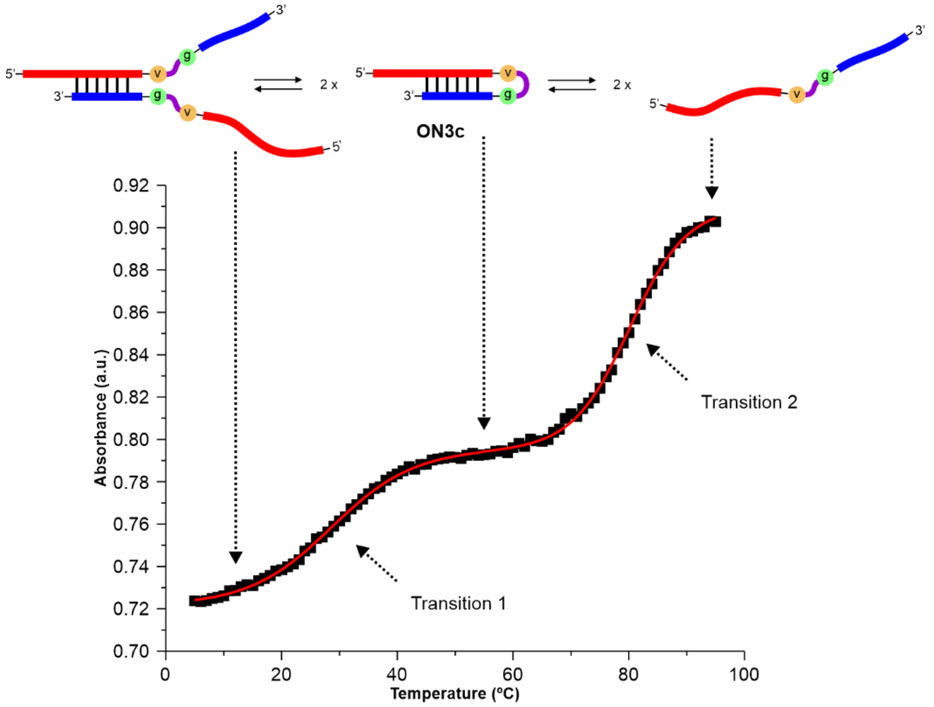

Figure S87. Melting curve of **ON3c**. Line shows the fit of the data to a three-state melting model using a double-sigmoidal Boltzmann function. T_{m1} = 28.4°C and T_{m2} = 80.1°C. Top panel shows representation of the three states involved in the two transitions.

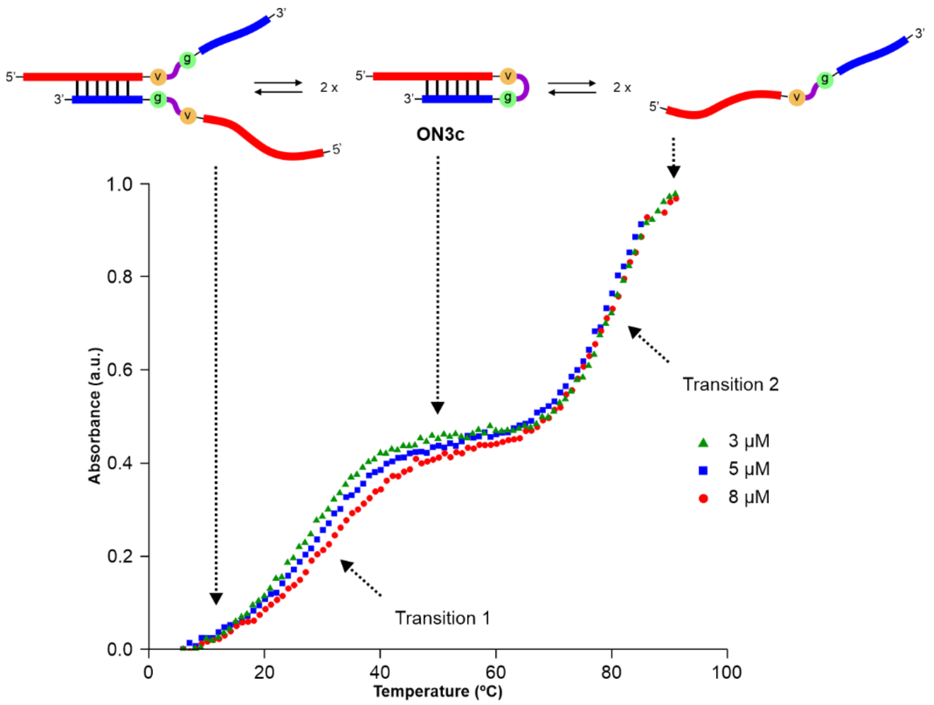

Figure S88. Normalized melting curves of **ON3c** at 3, 5 and 8 μM concentration. Top panel shows representation of the three states involved in the two transitions.

We observed a two-step melting profile in the experiments performed with the RNA oligonucleotides **ON3a** and **ON3c** (**Figure S86**, **Figure S87** and **Figure S88**). At low temperature (transition 1), the double strand (duplex) is transformed into the hairpin. At high temperature (transition 2), the hairpin is converted into the random coil. The intermolecular and intramolecular dissociation of the base pairs, *i.e*. breaking of hydrogen-bonding and π-stacking interactions, is induced by the increase in temperature over the course of the experiments.[15,16]

17. NMR spectra of synthesized compounds

^{1}H and $^{13}C\{^{1}H\}$ NMR spectra of compound 3a

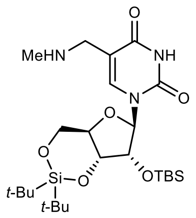

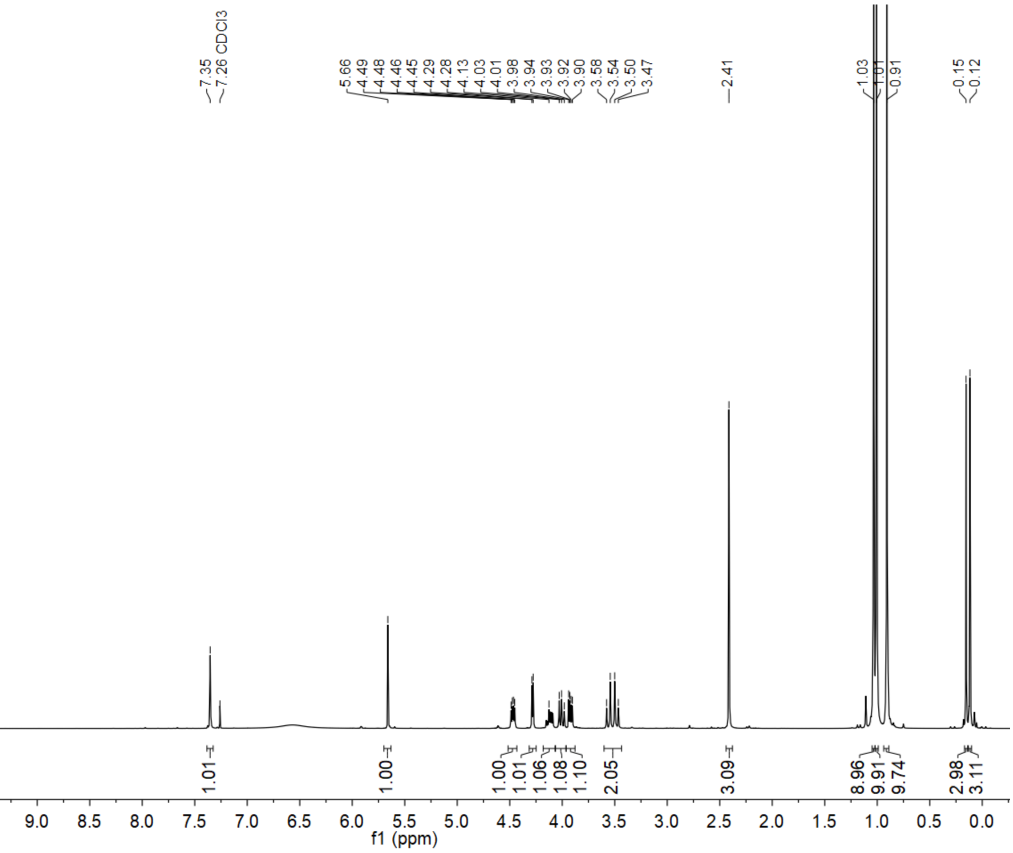

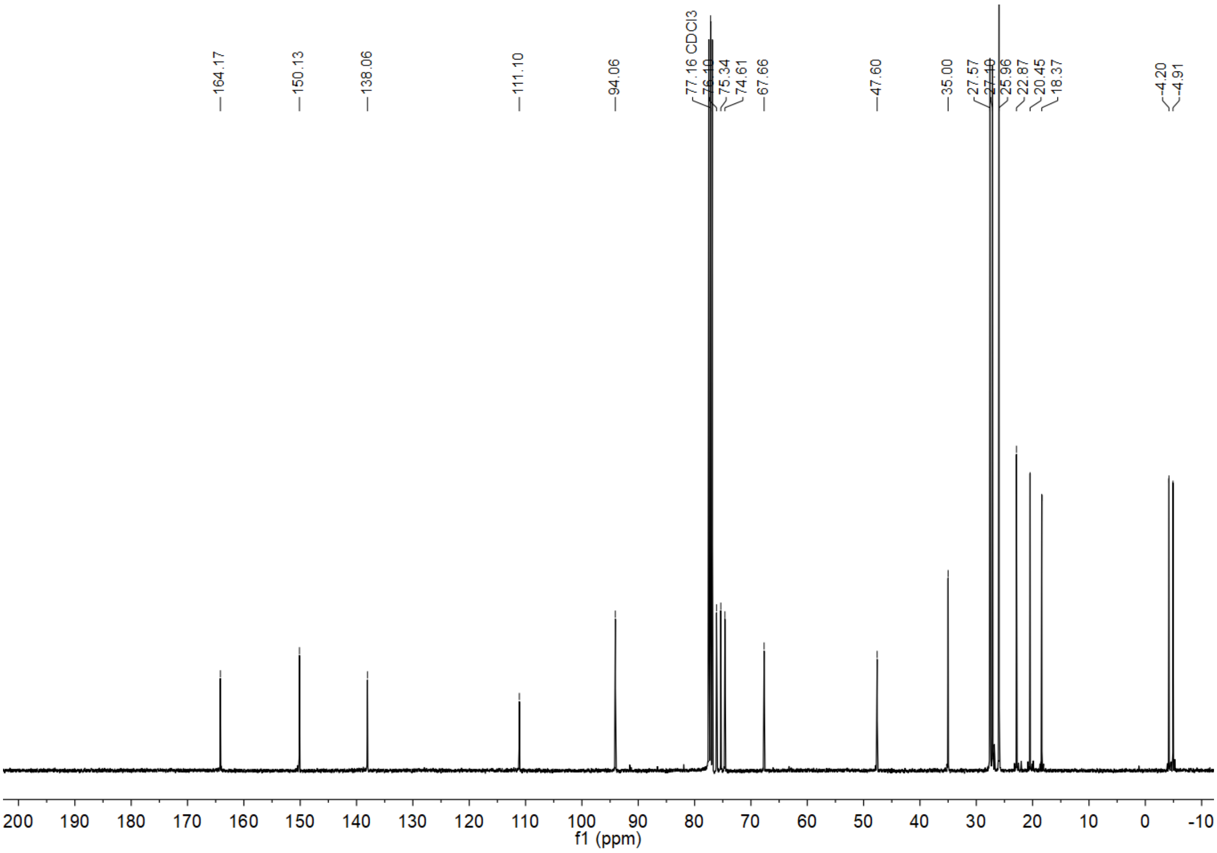

^{1}H and $^{13}C\{^{1}H\}$ NMR spectra of compound 3b

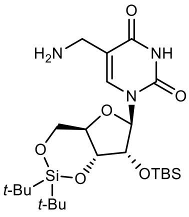

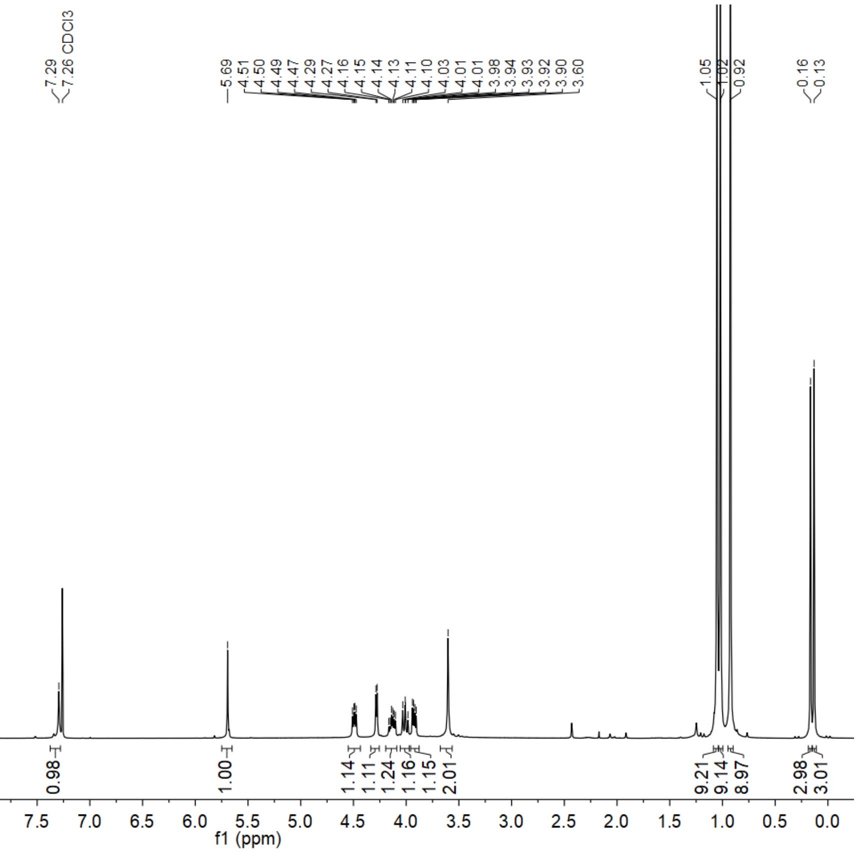

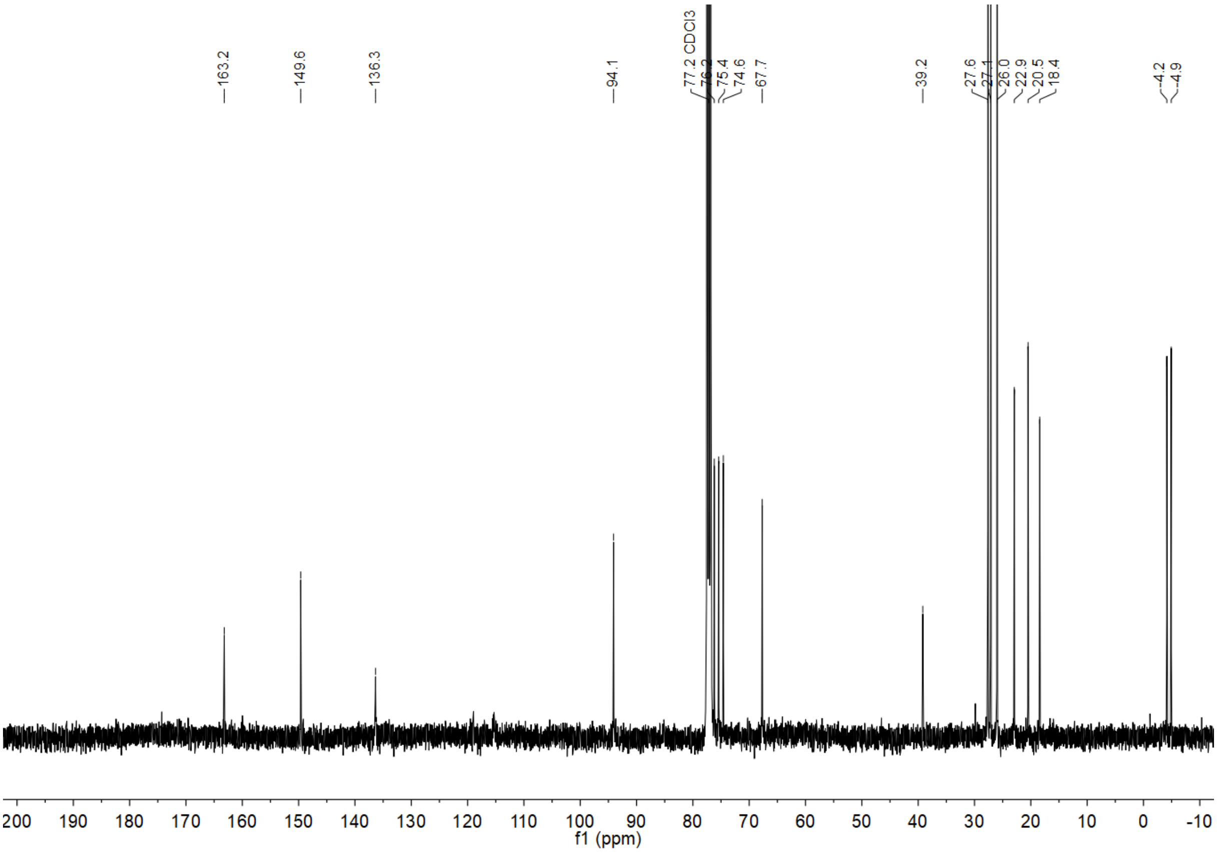

^{1}H and ^{13}C{^{1}H} NMR spectra of compound 4a

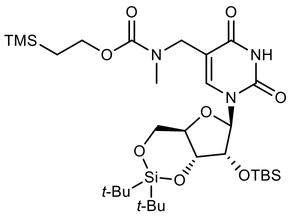

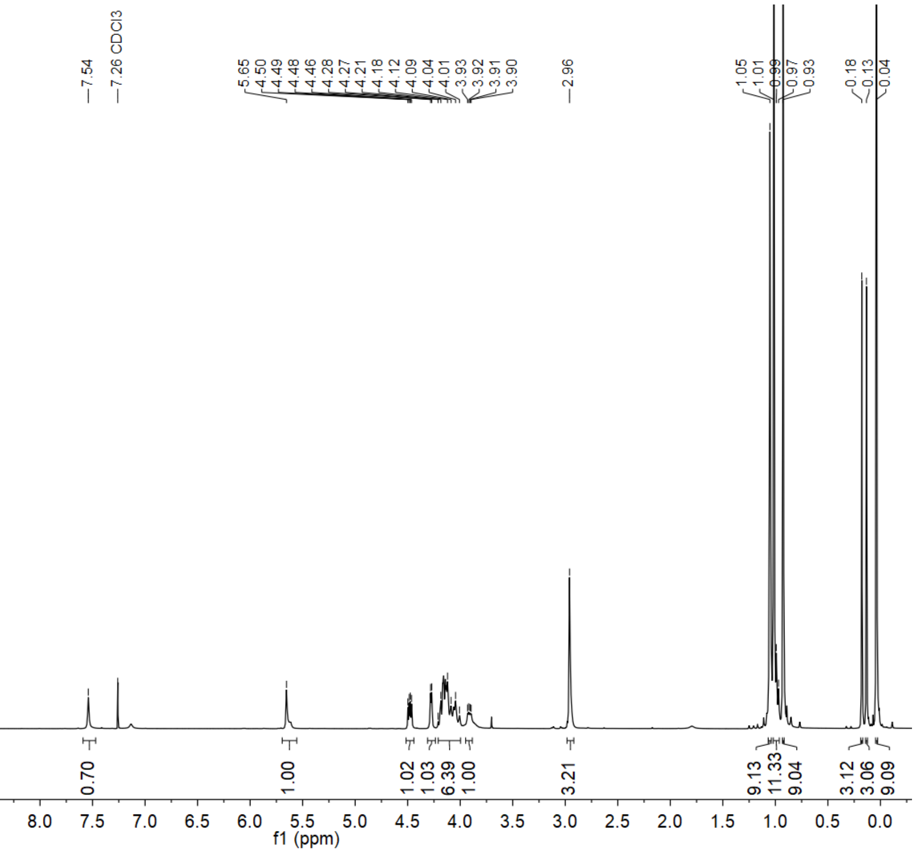

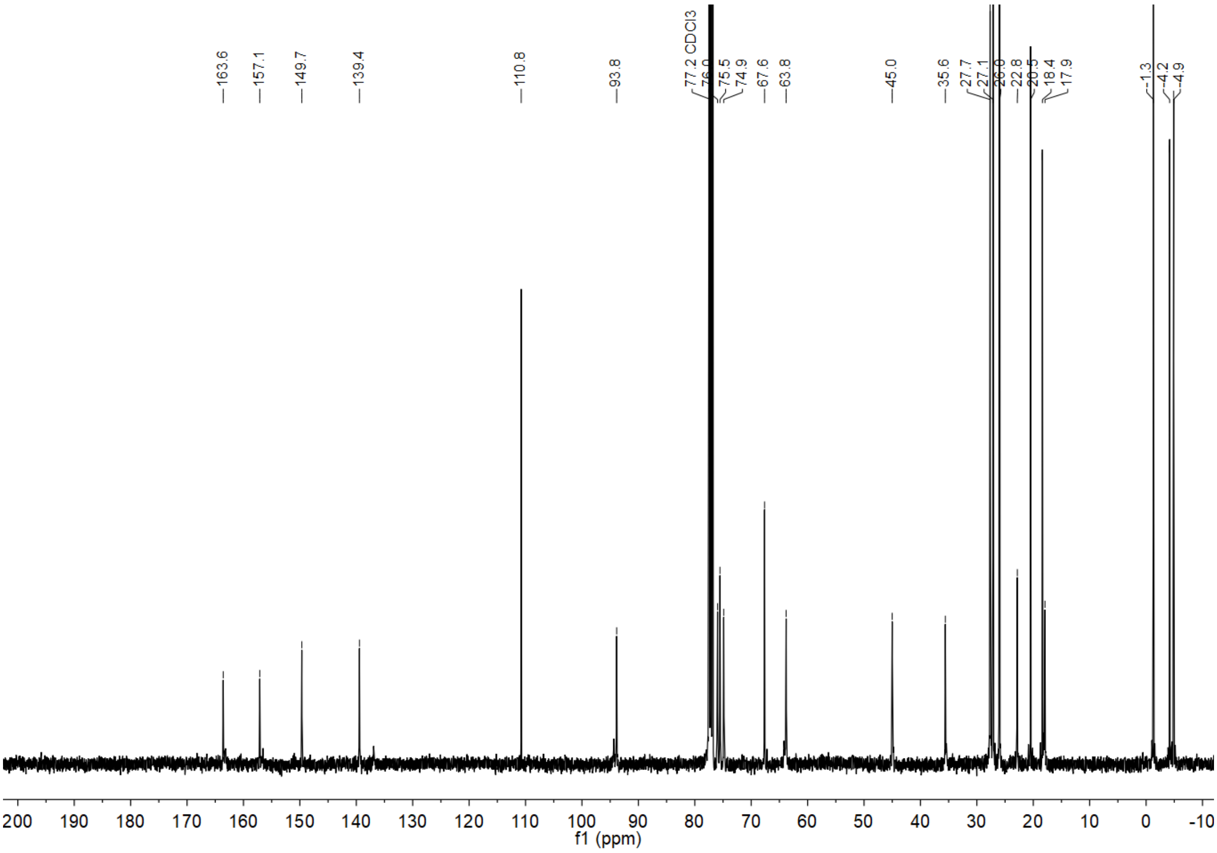

^{1}H and ^{13}C{^{1}H} NMR spectra of compound 4b

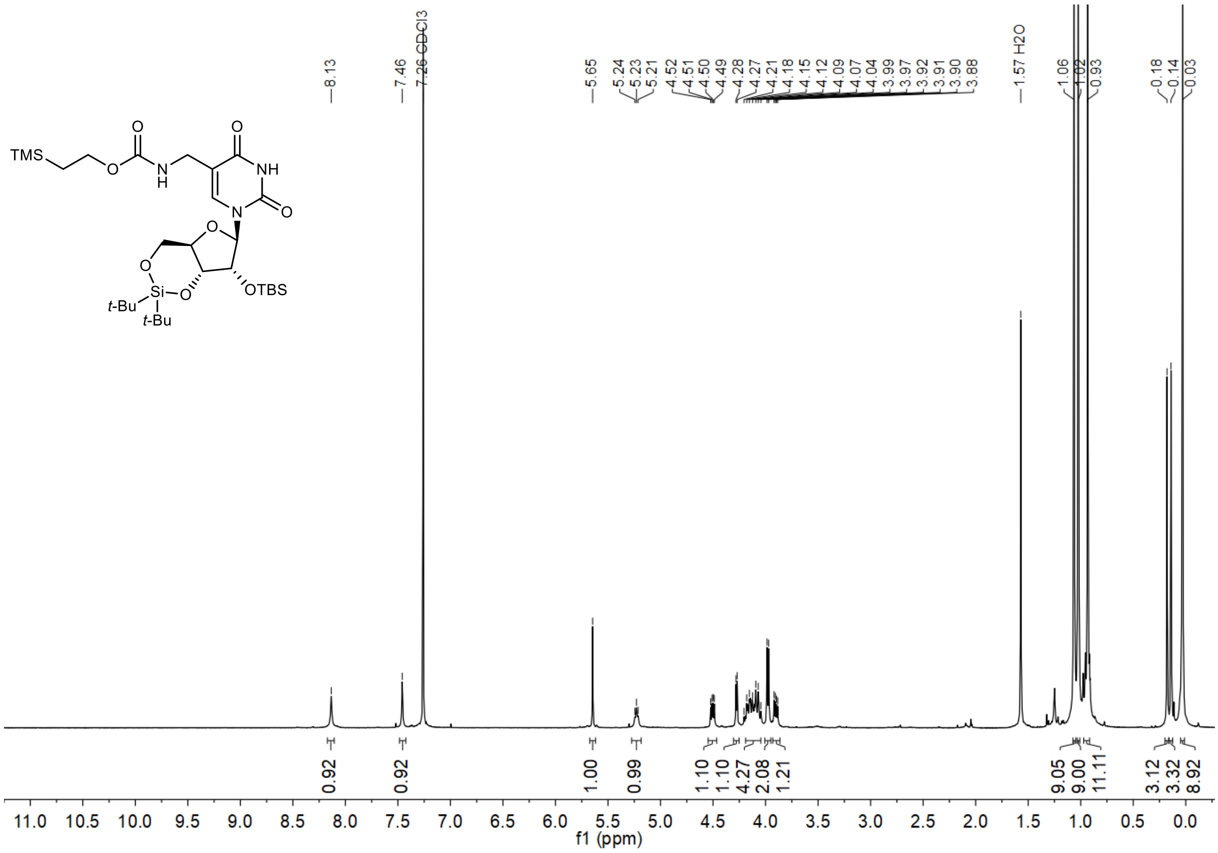

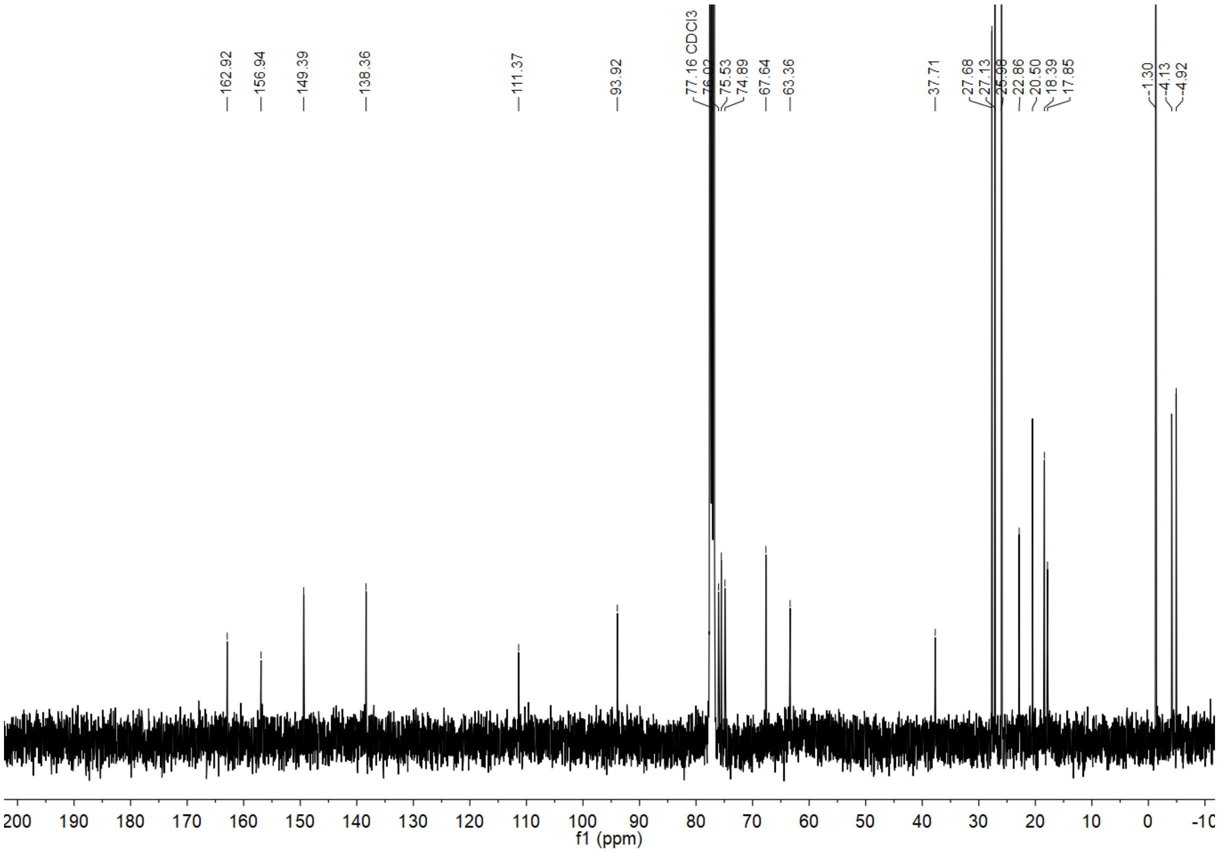

^{1}H and $^{13}C\{^{1}H\}$ NMR spectra of compound 4c

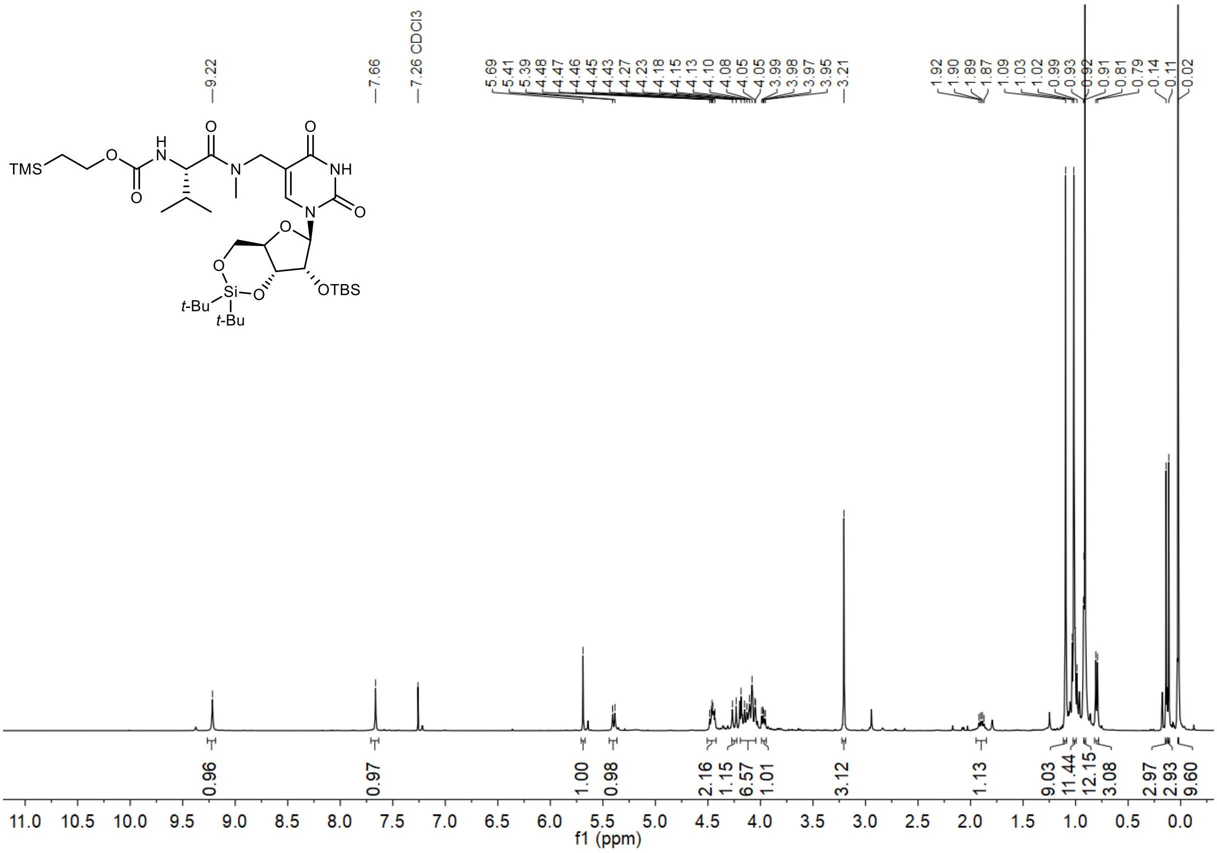

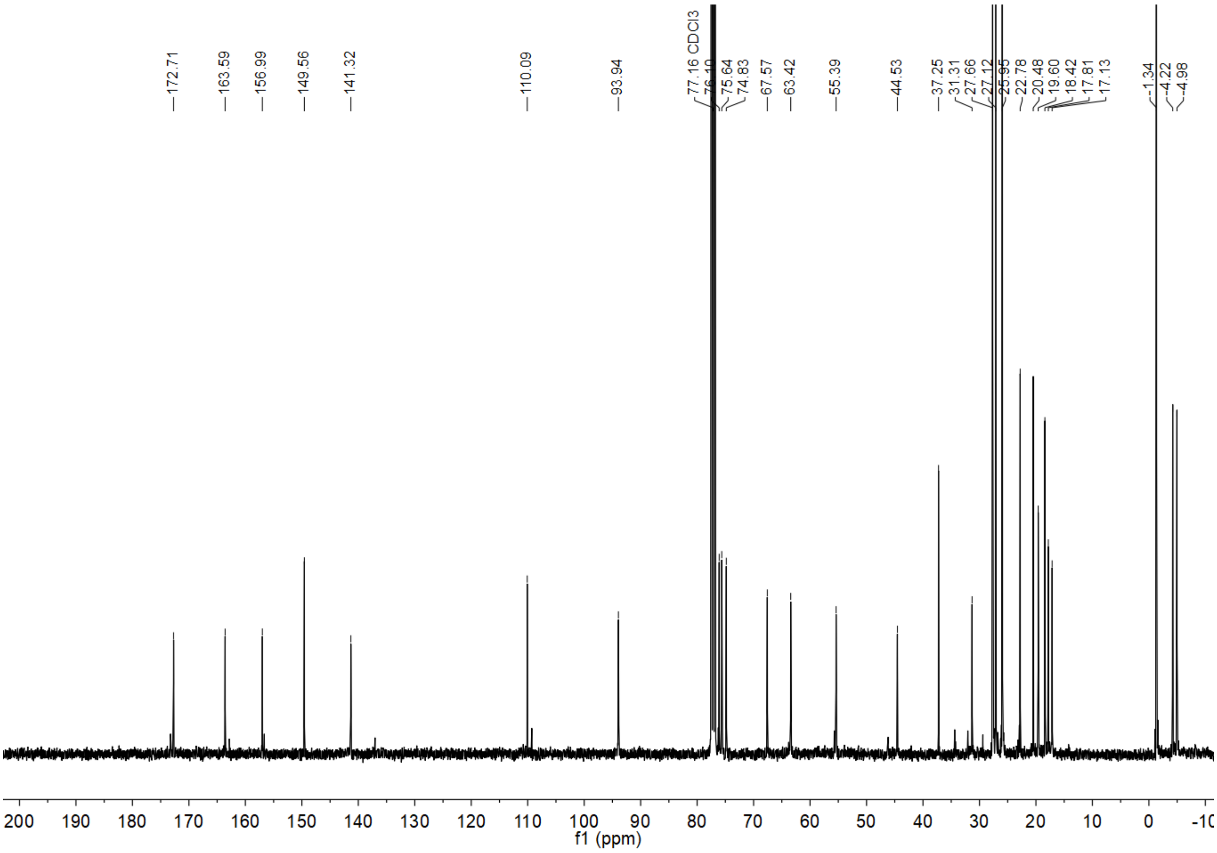

^{1}H and ^{13}C{^{1}H} NMR spectra of compound 5a

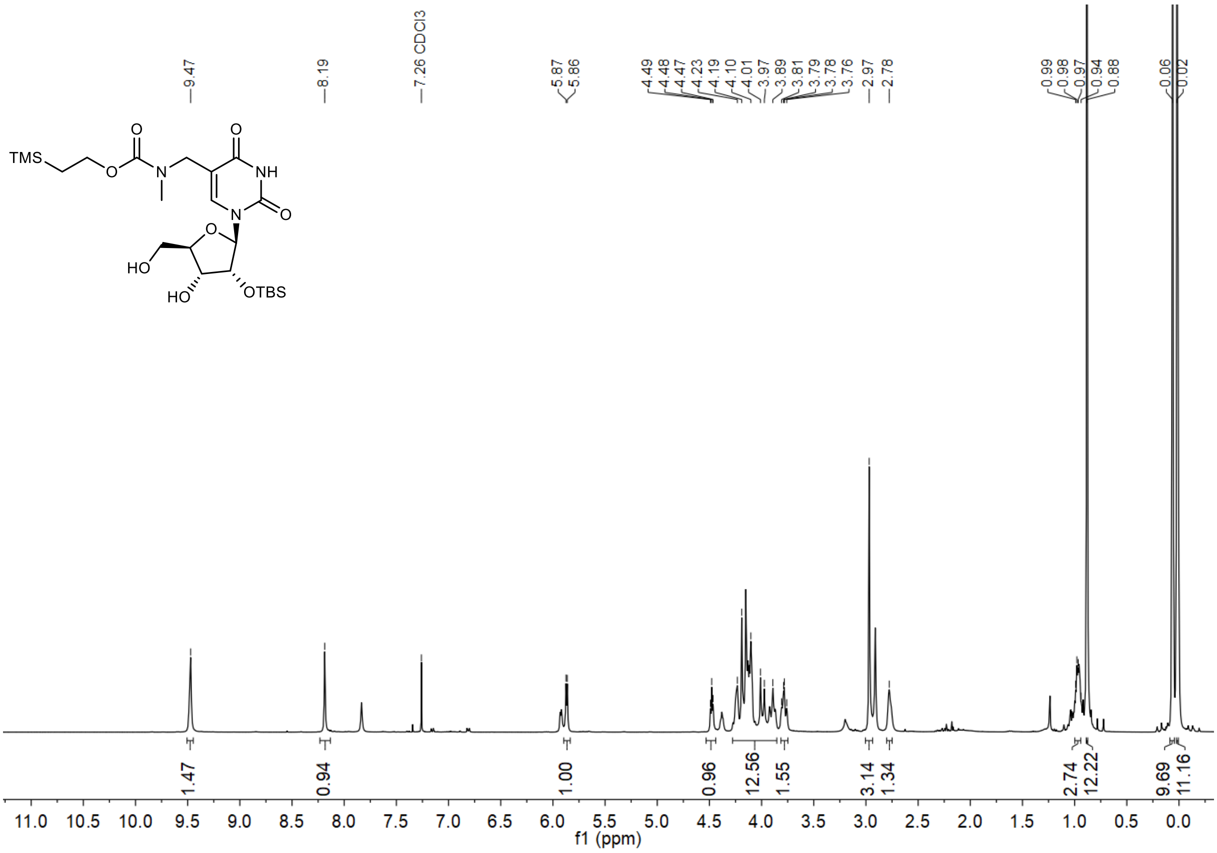

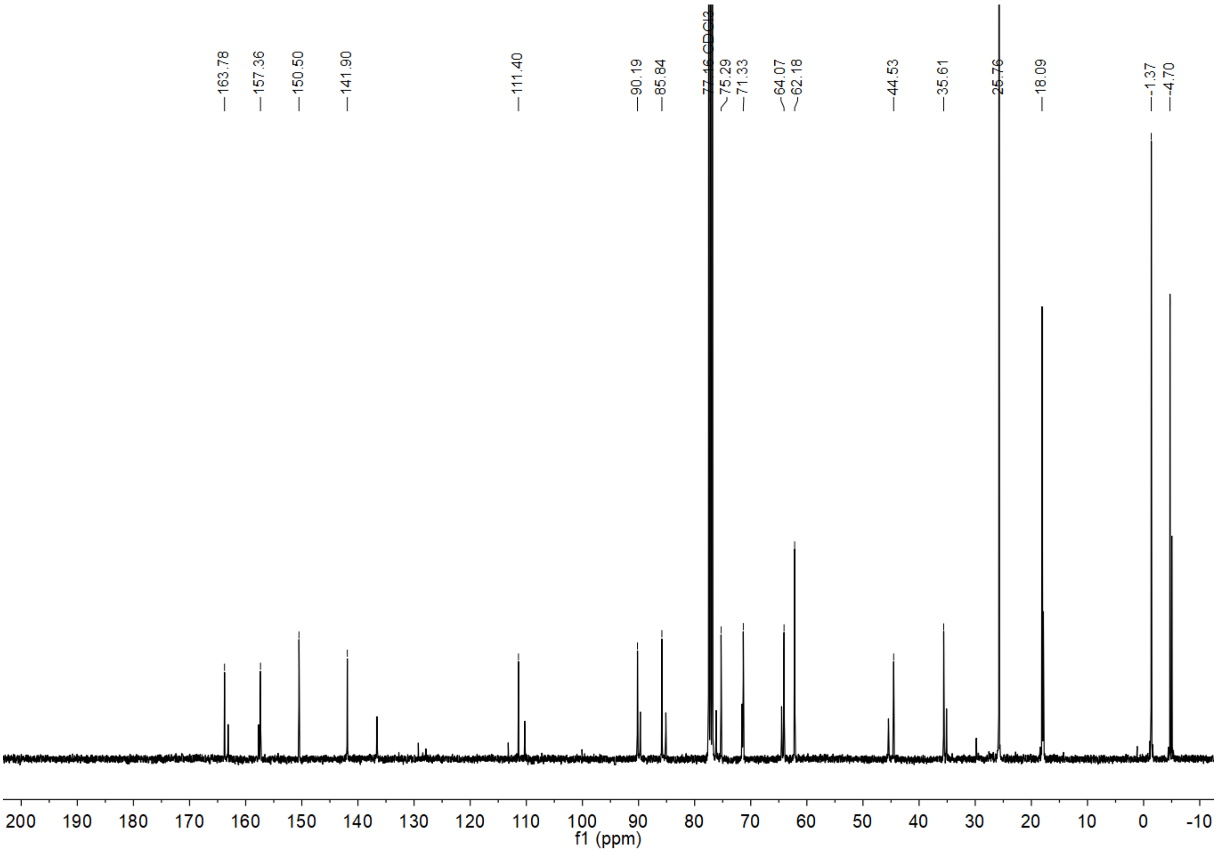

^{1}H and ^{13}C{^{1}H} NMR spectra of compound 5b

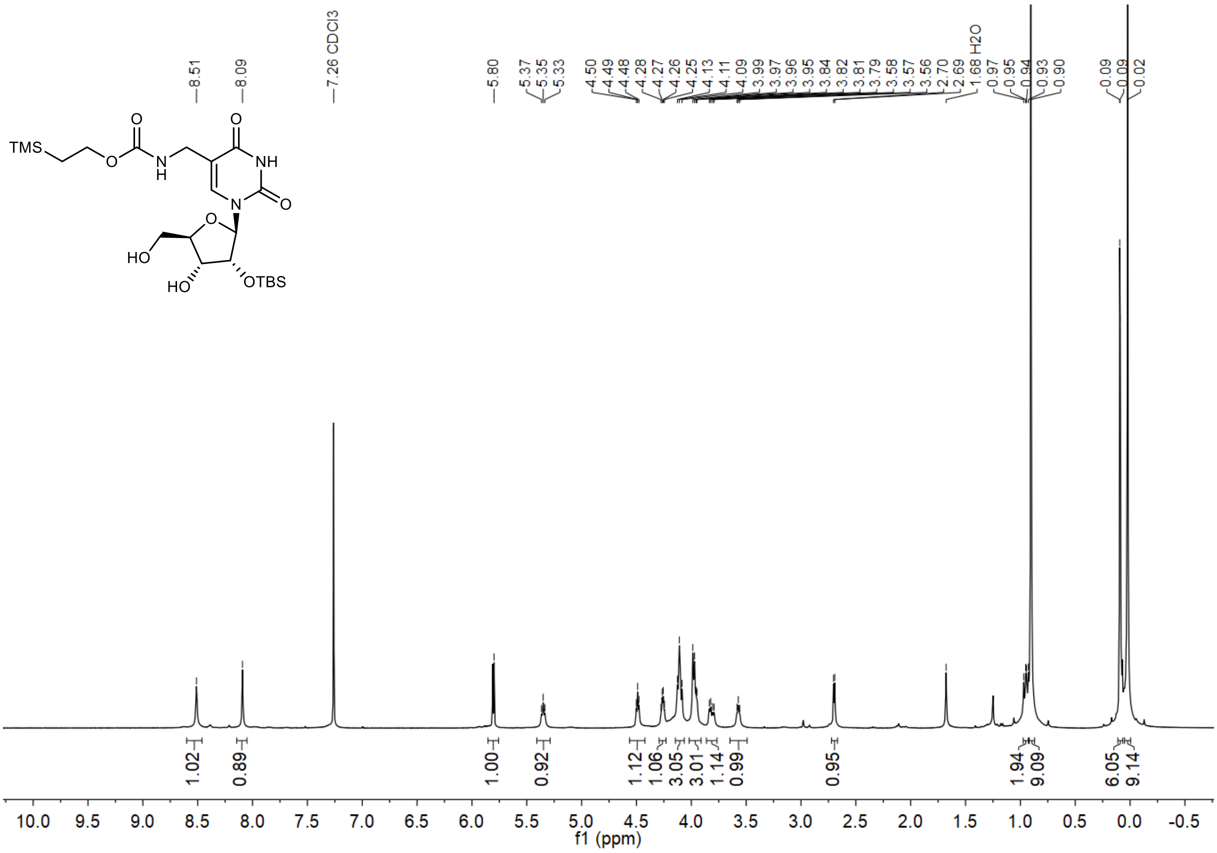

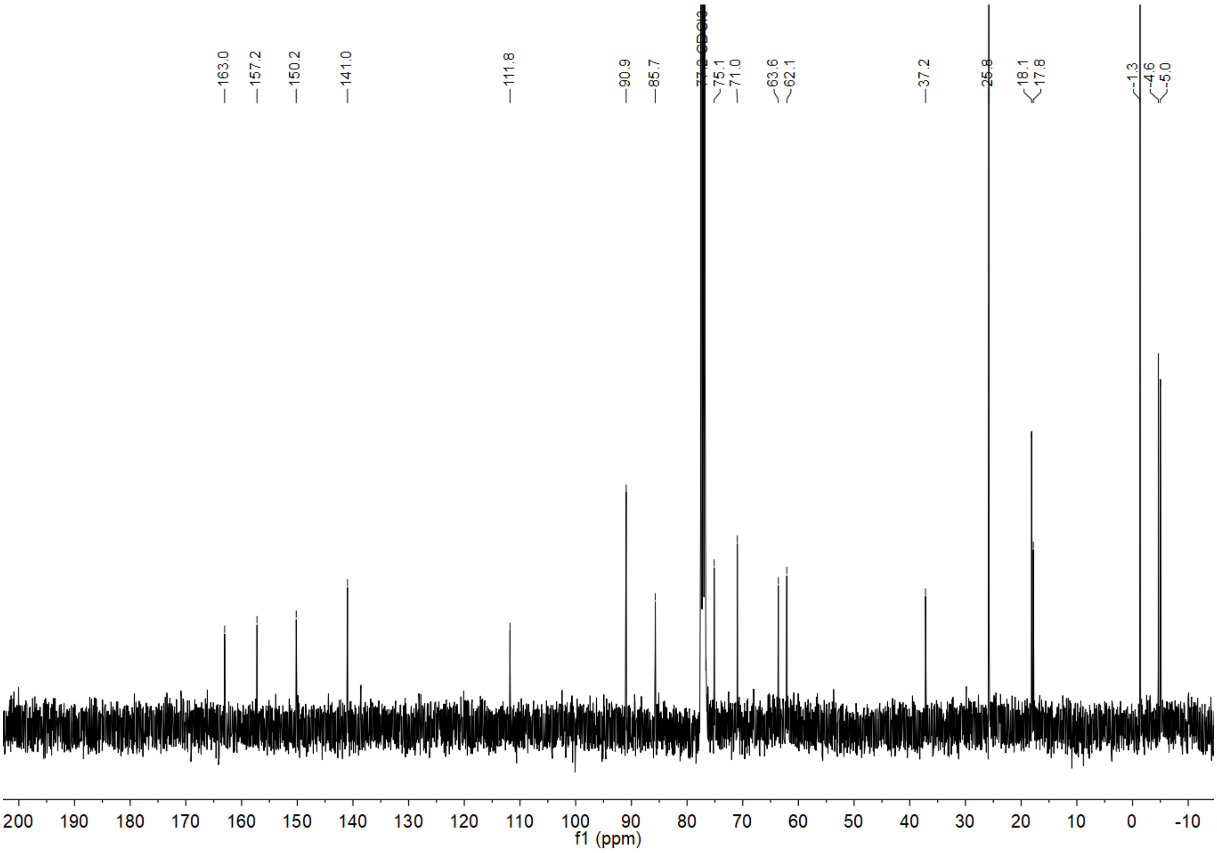

^1H and $^{13}C\{^1H\}$ NMR spectra of compound 5c

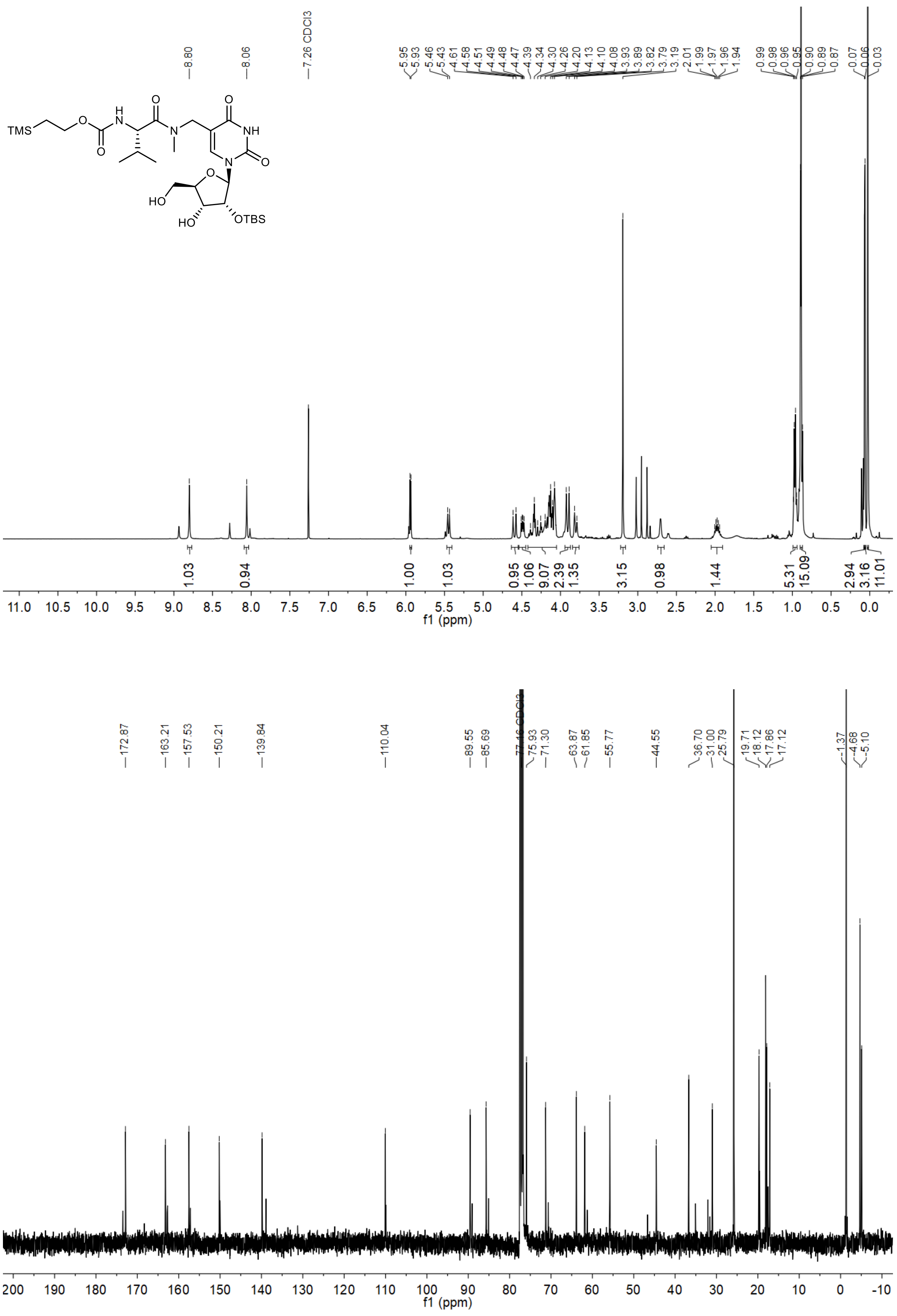

^{1}H and ^{13}C{^{1}H} NMR spectra of compound 6a

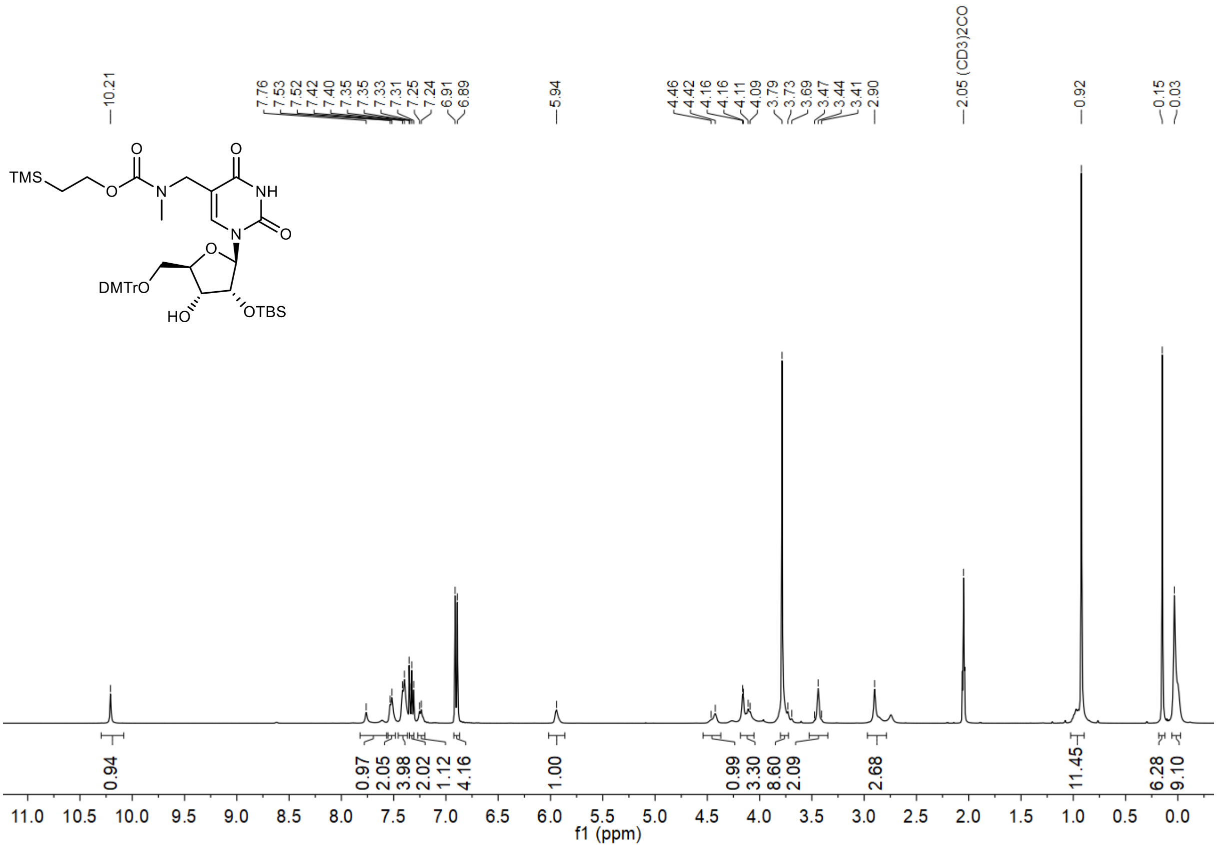

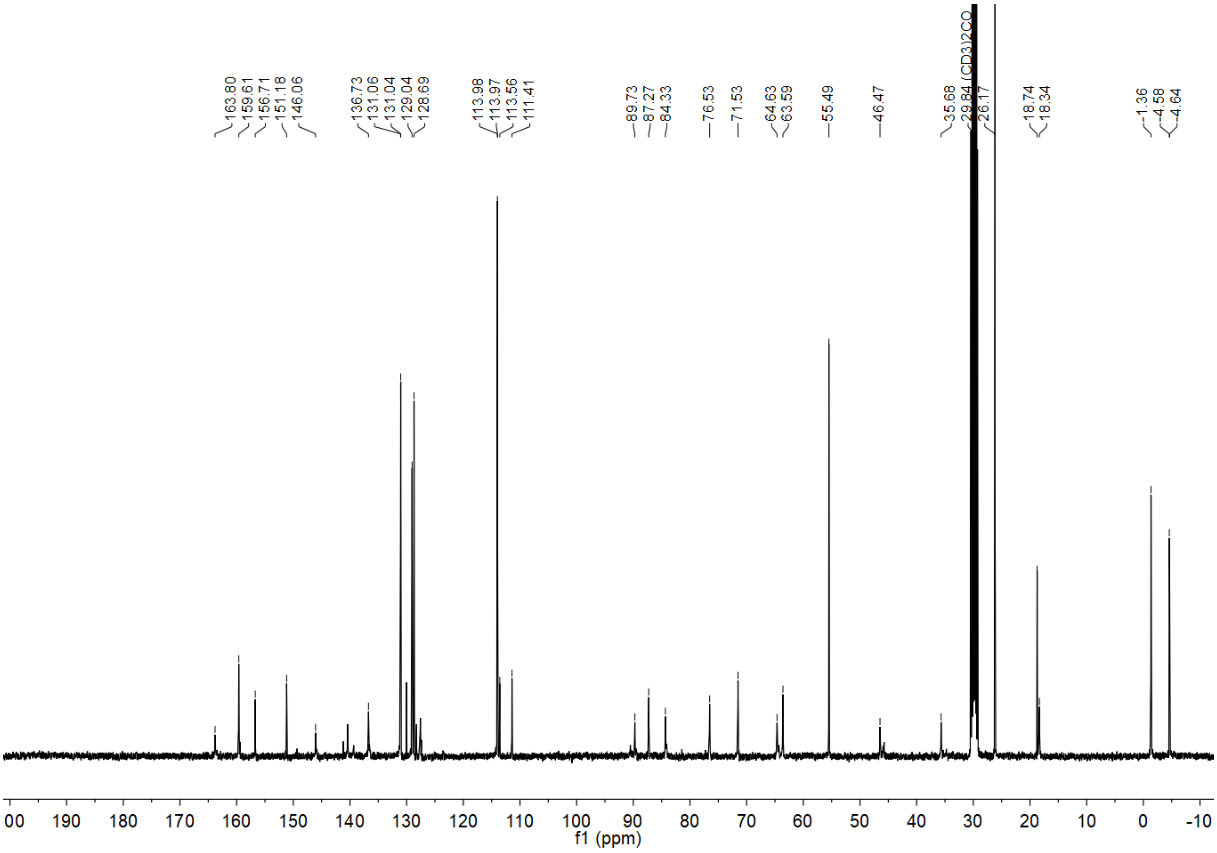

^{1}H and ^{13}C{^{1}H} NMR spectra of compound 6b

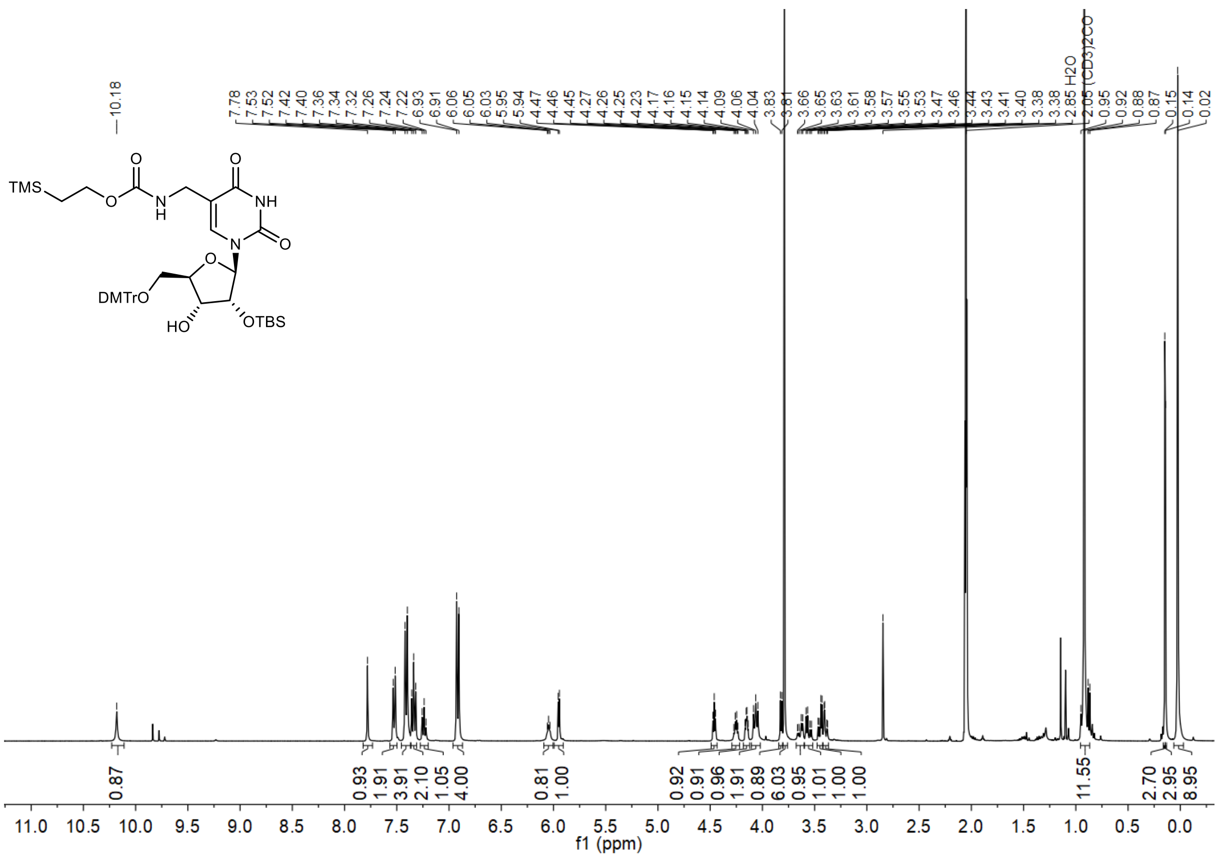

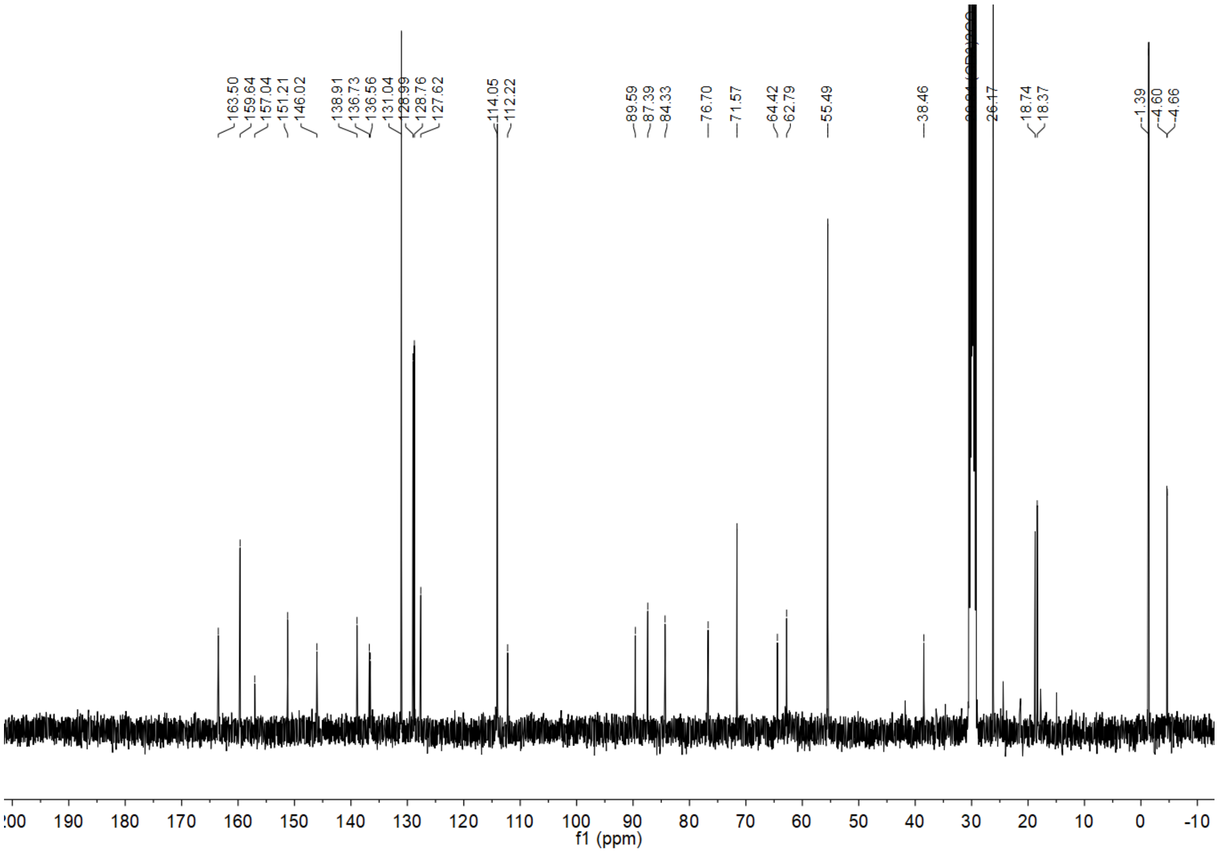

^{1}H and $^{13}C\{^{1}H\}$ NMR spectra of compound 6c

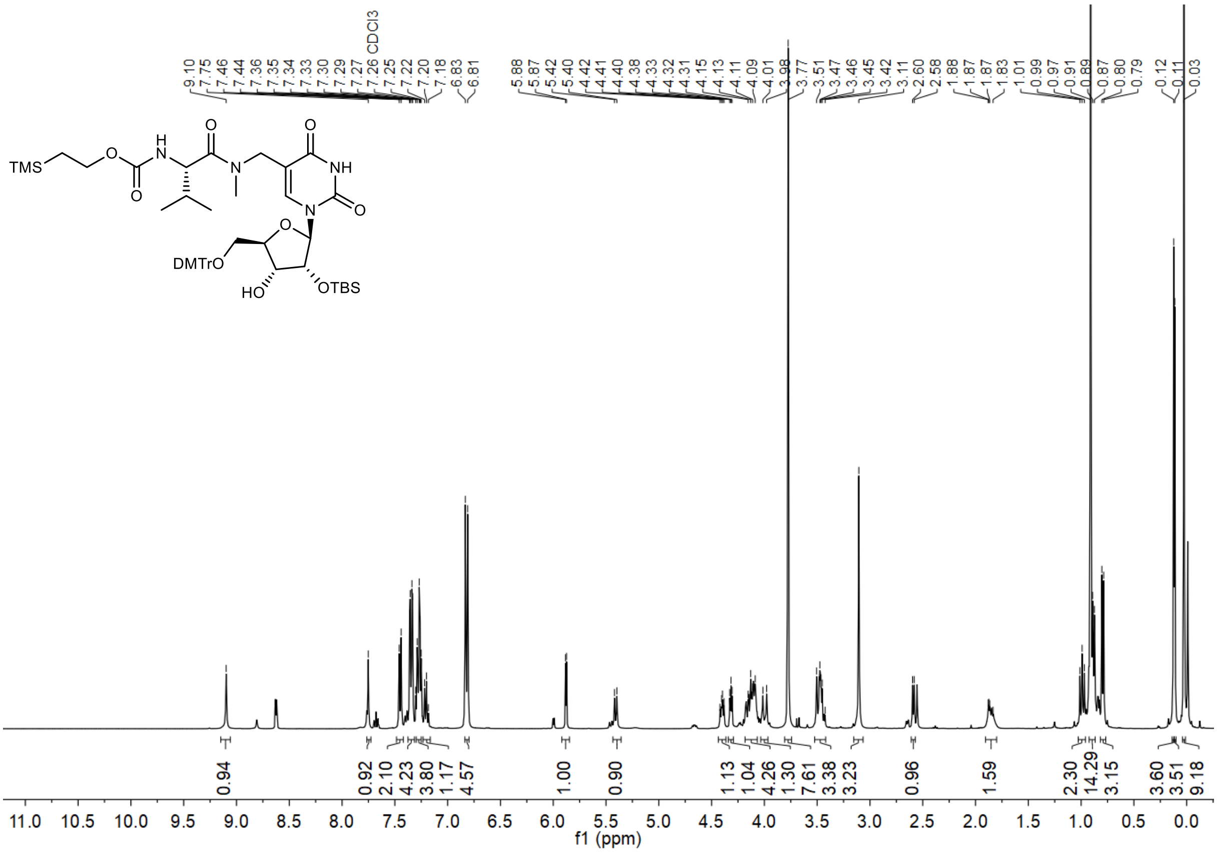

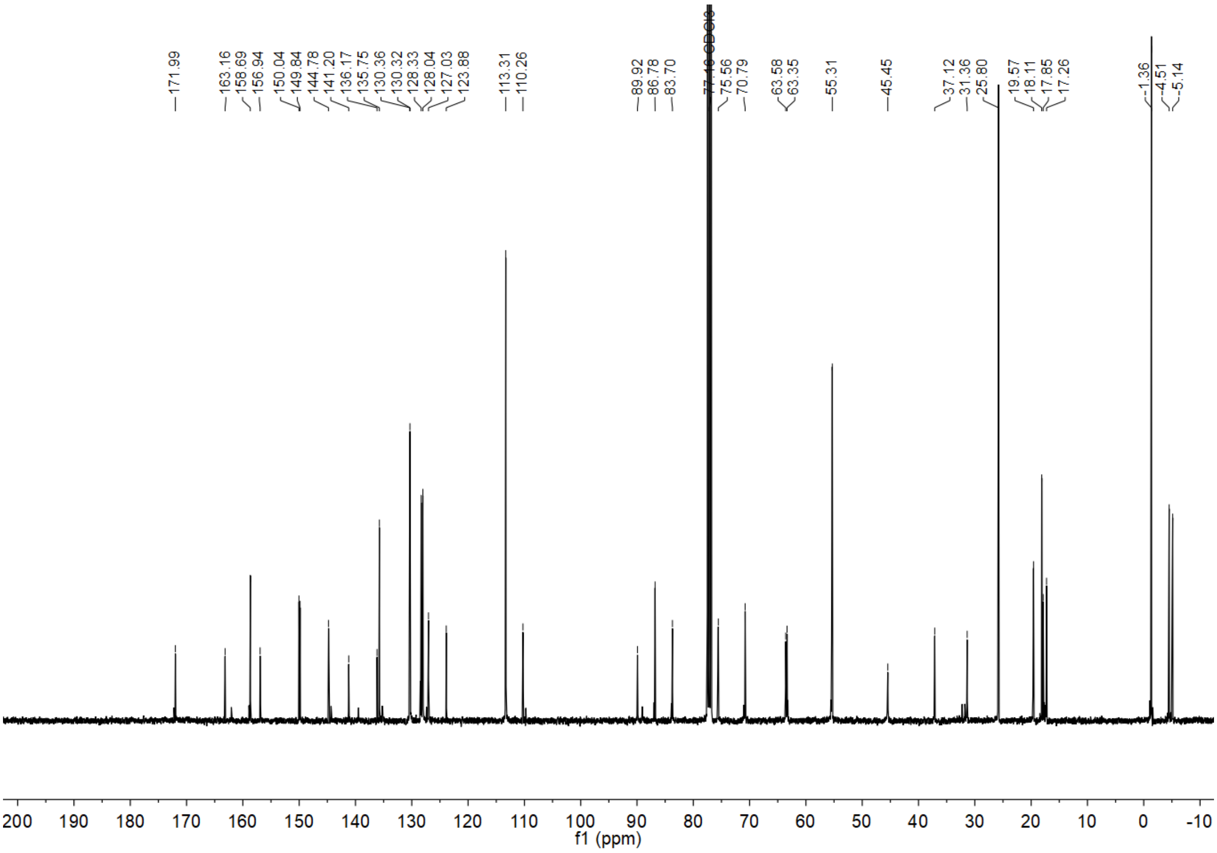

$^{31}P\{^1H\}$ NMR spectrum of compound 7a

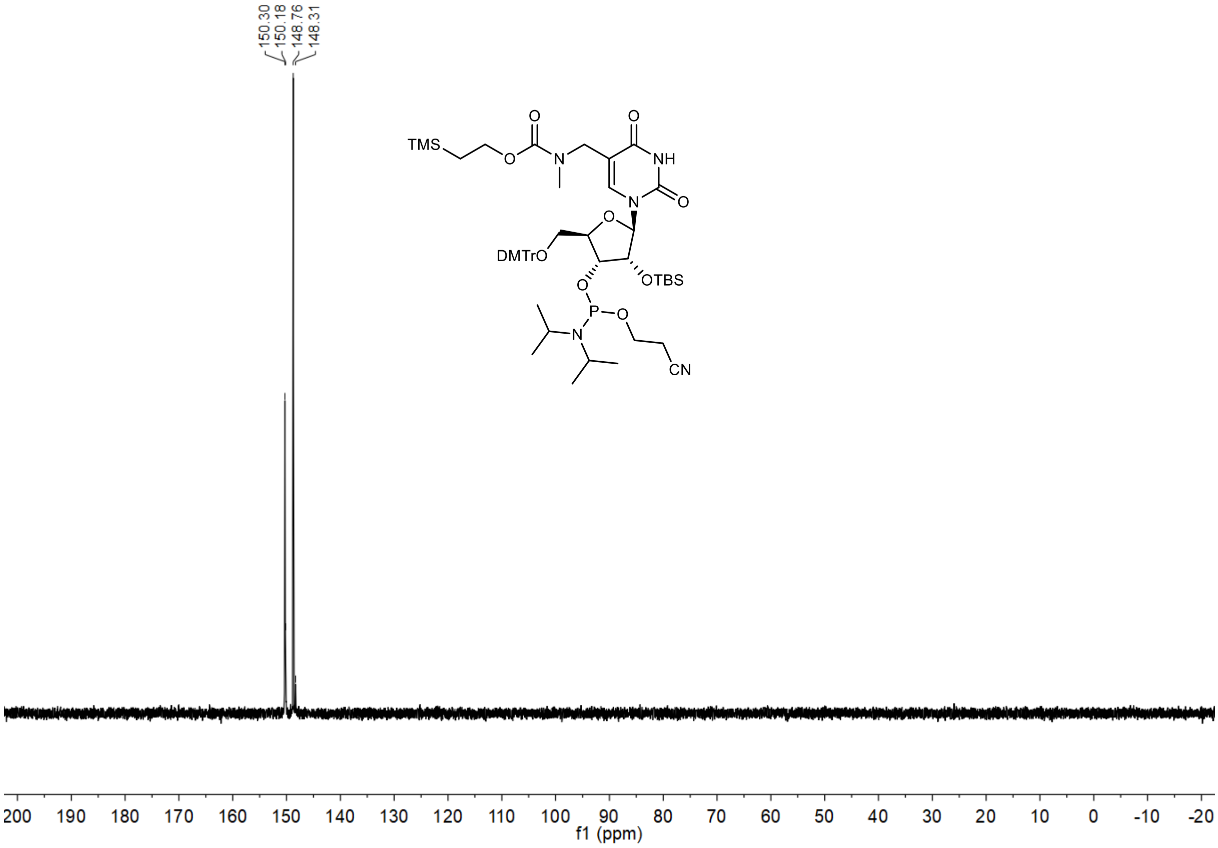

$^{31}P\{^1H\}$ NMR spectrum of compound 7b

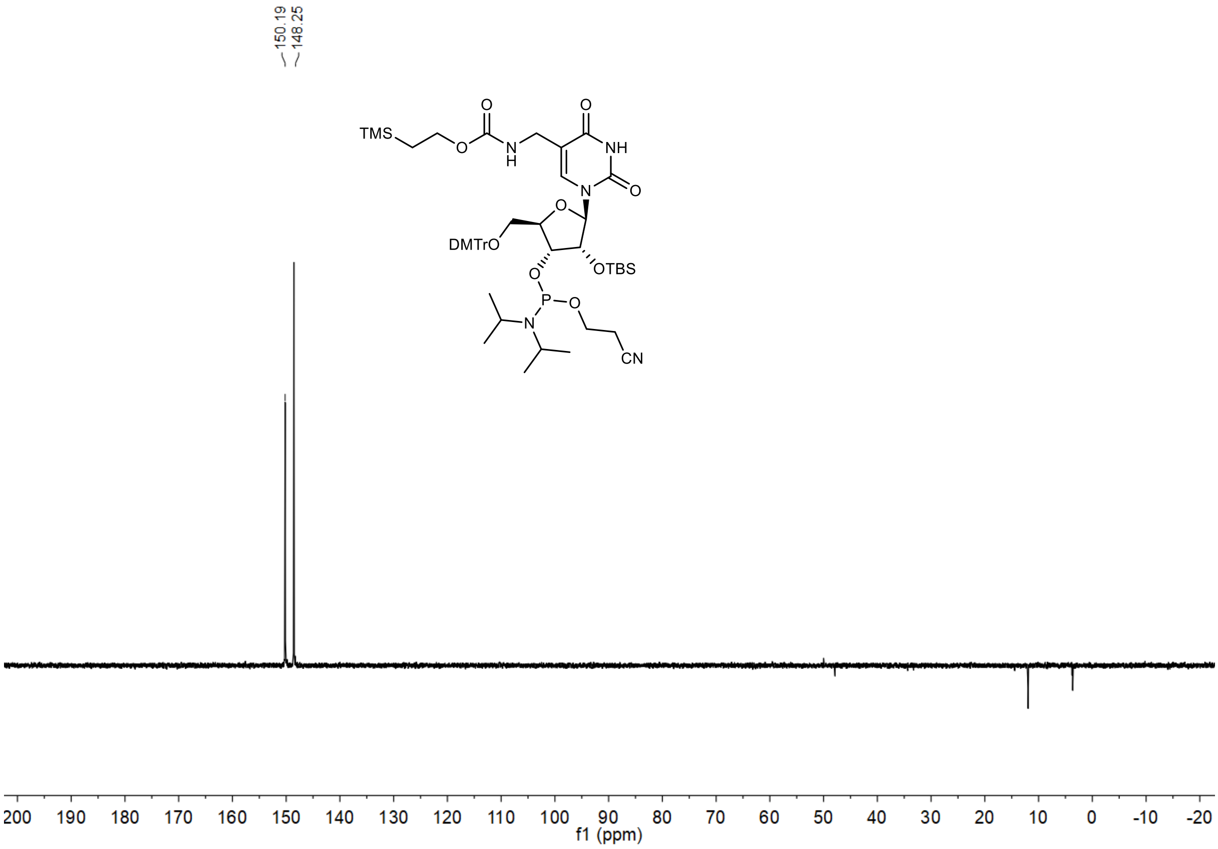

$^{31}P\{^{1}H\}$ NMR spectrum of compound 7c

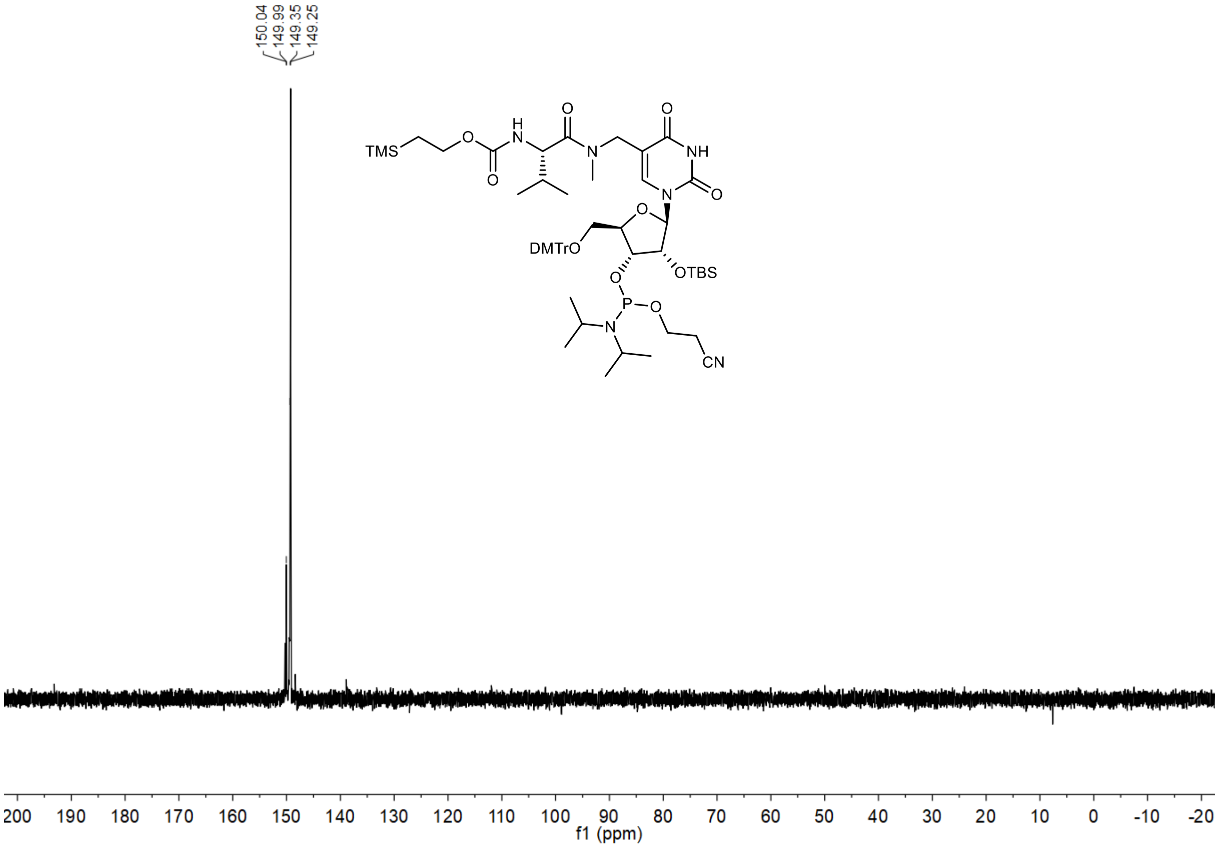

^{1}H and $^{13}C\{^{1}H\}$ NMR spectra of compound H-Ala-Onpe•HCl

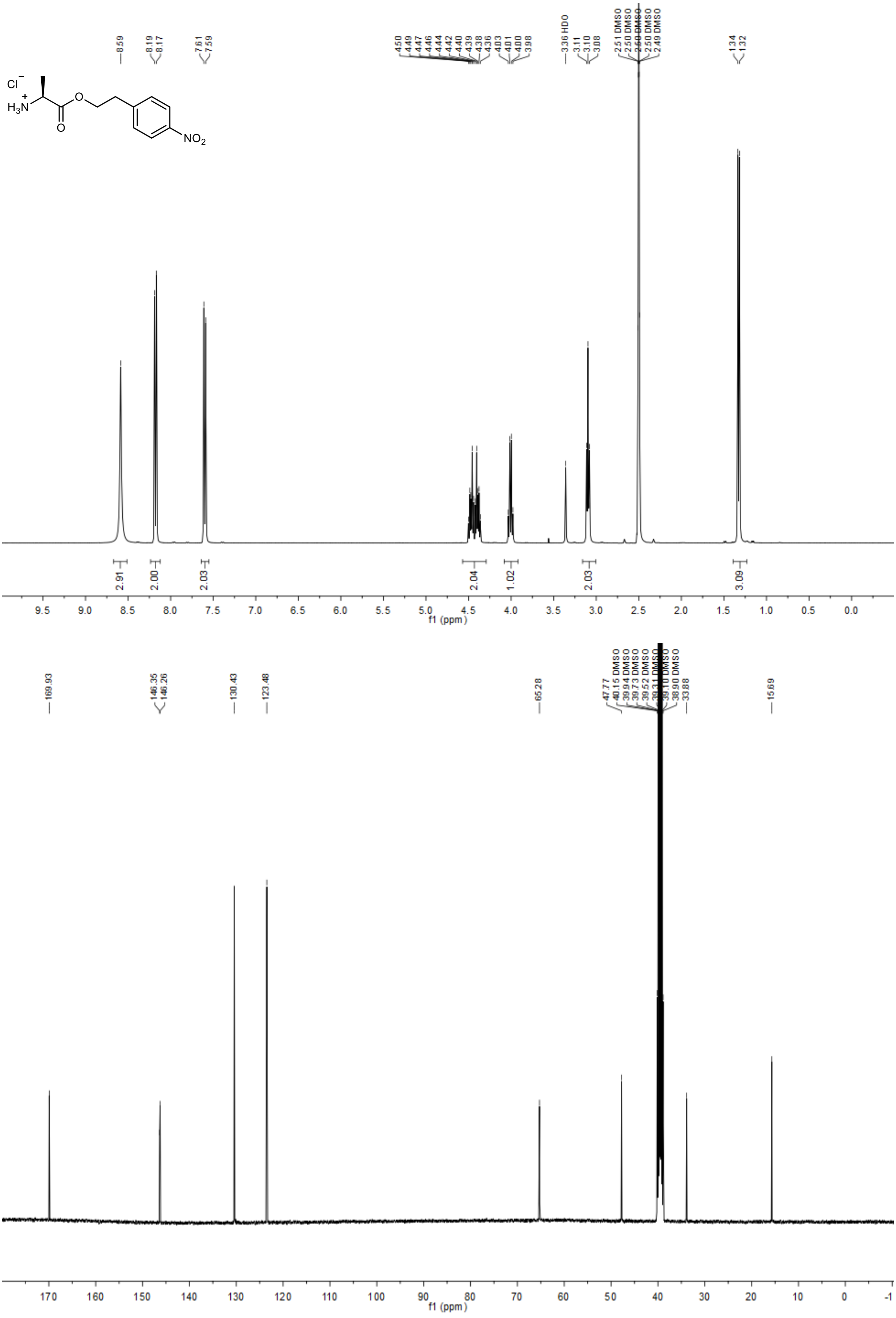

^{1}H and $^{13}C\{^{1}H\}$ NMR spectra of compound H-Leu-Onpe•HCl

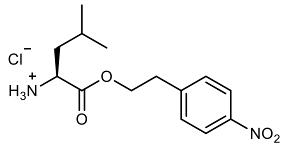

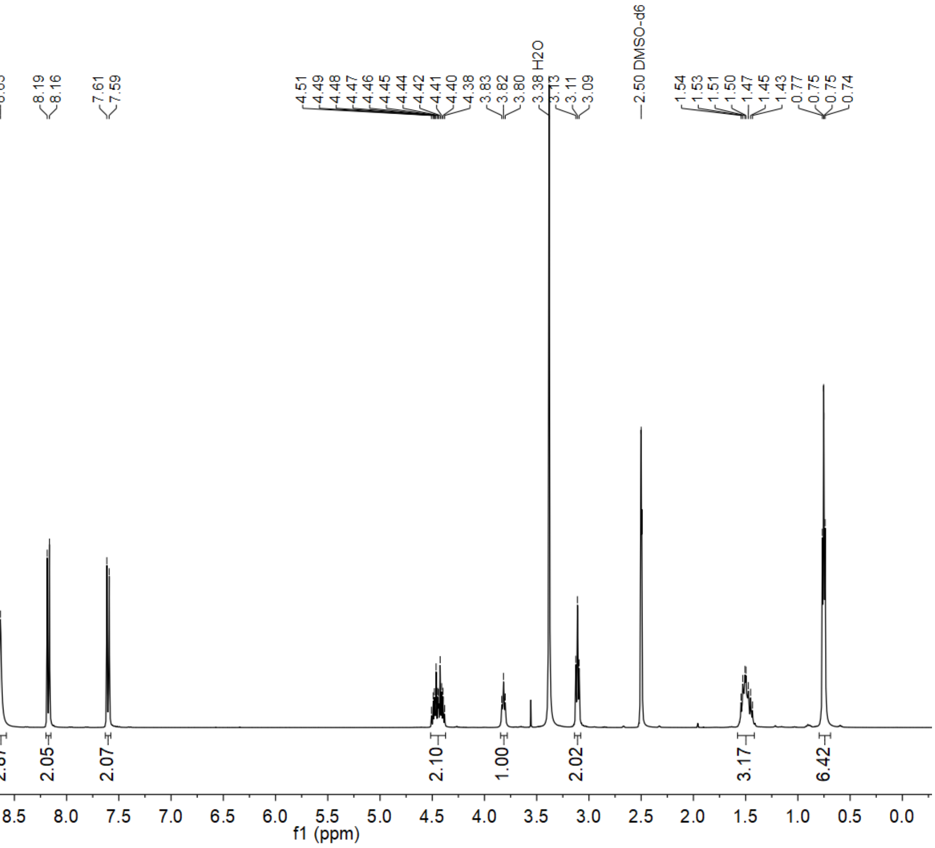

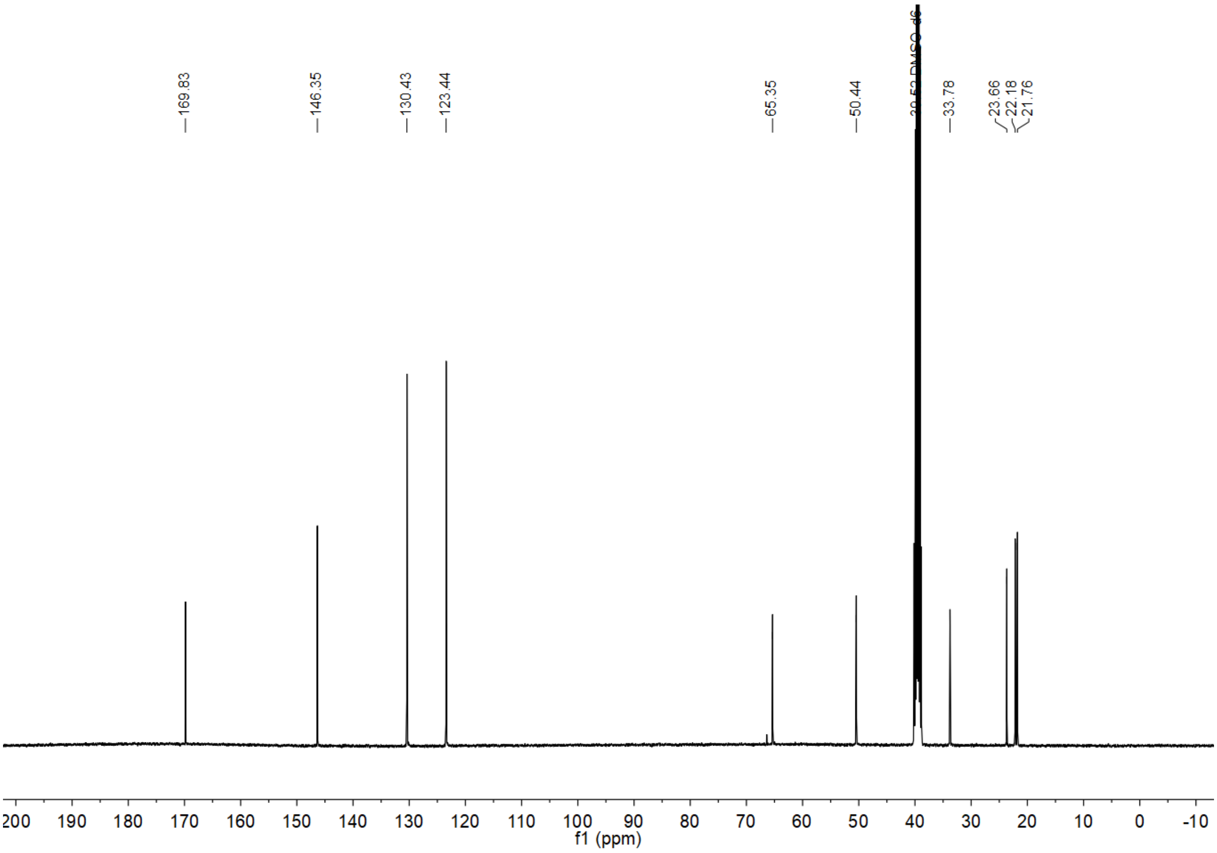

^{1}H and ^{13}C{^{1}H} NMR spectra of compound H-Pro-Onpe

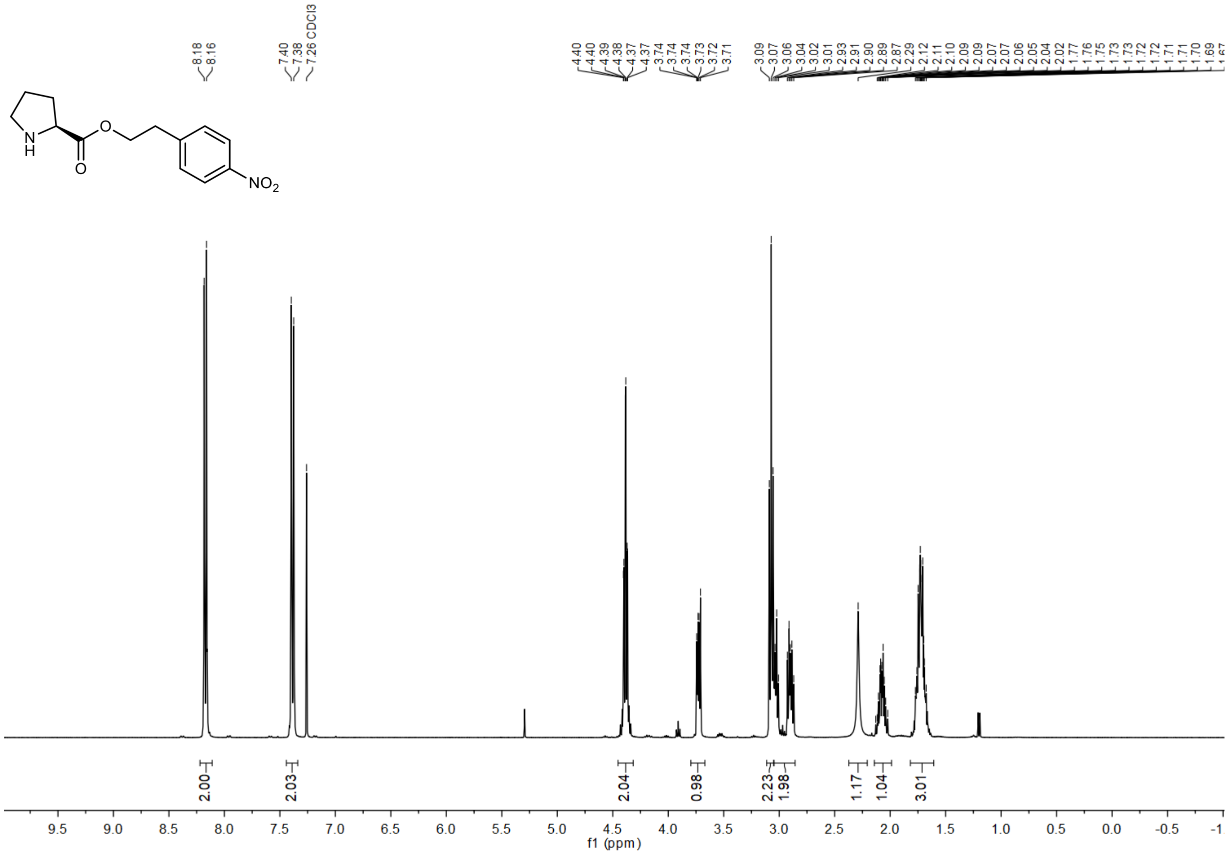

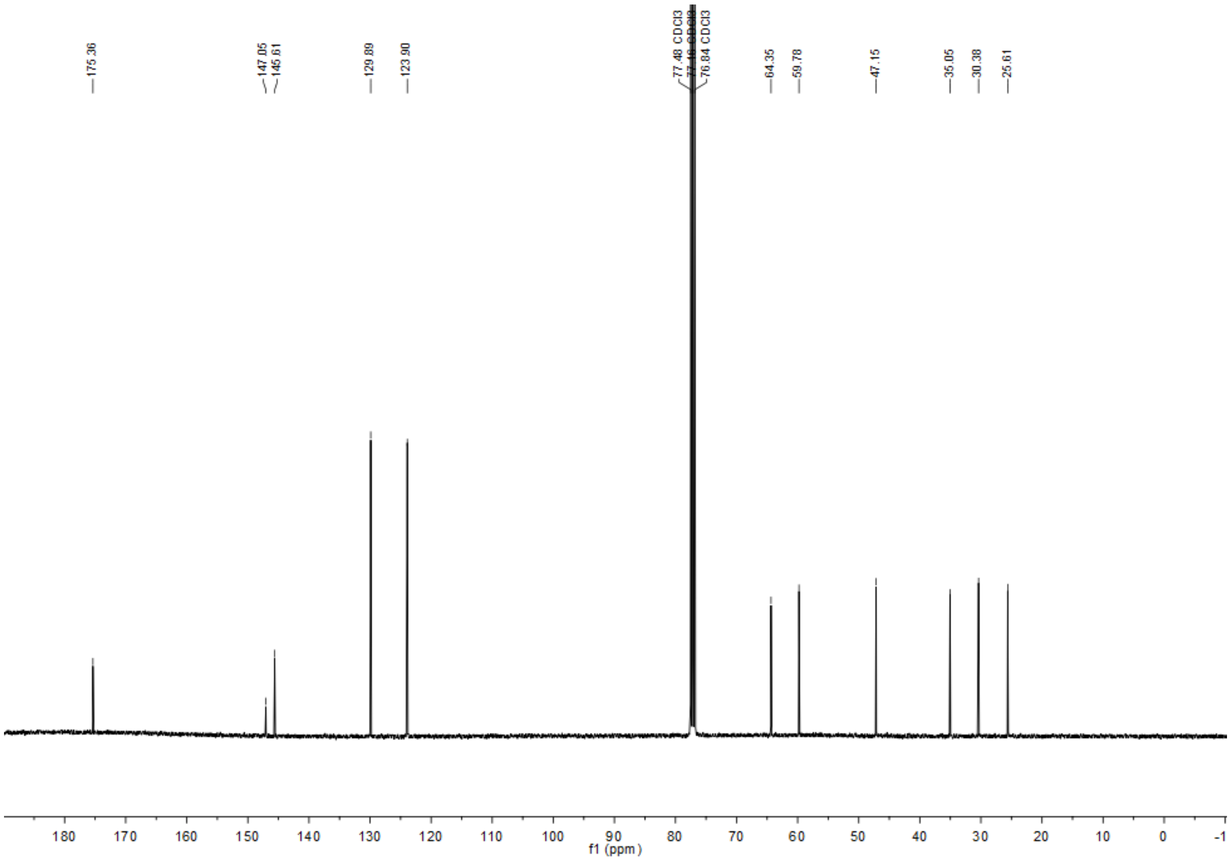

^{1}H and $^{13}C\{^{1}H\}$ NMR spectra of compound H-Met-Onpe•HCl

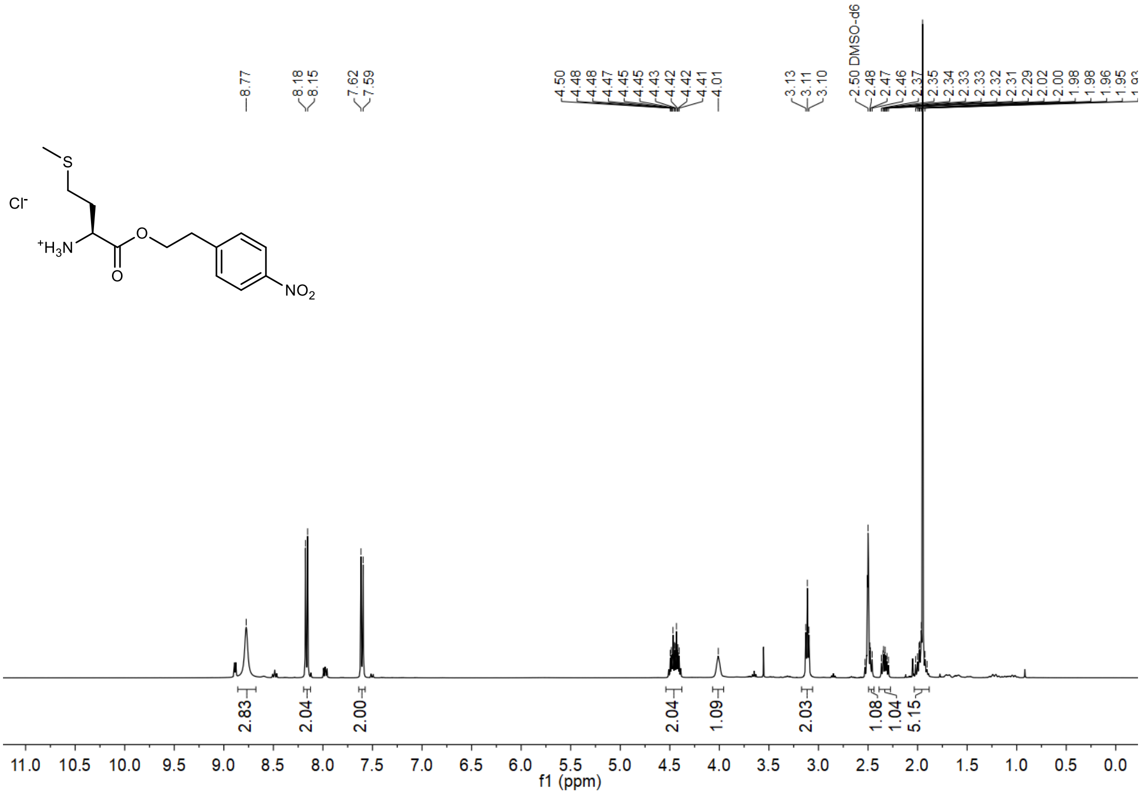

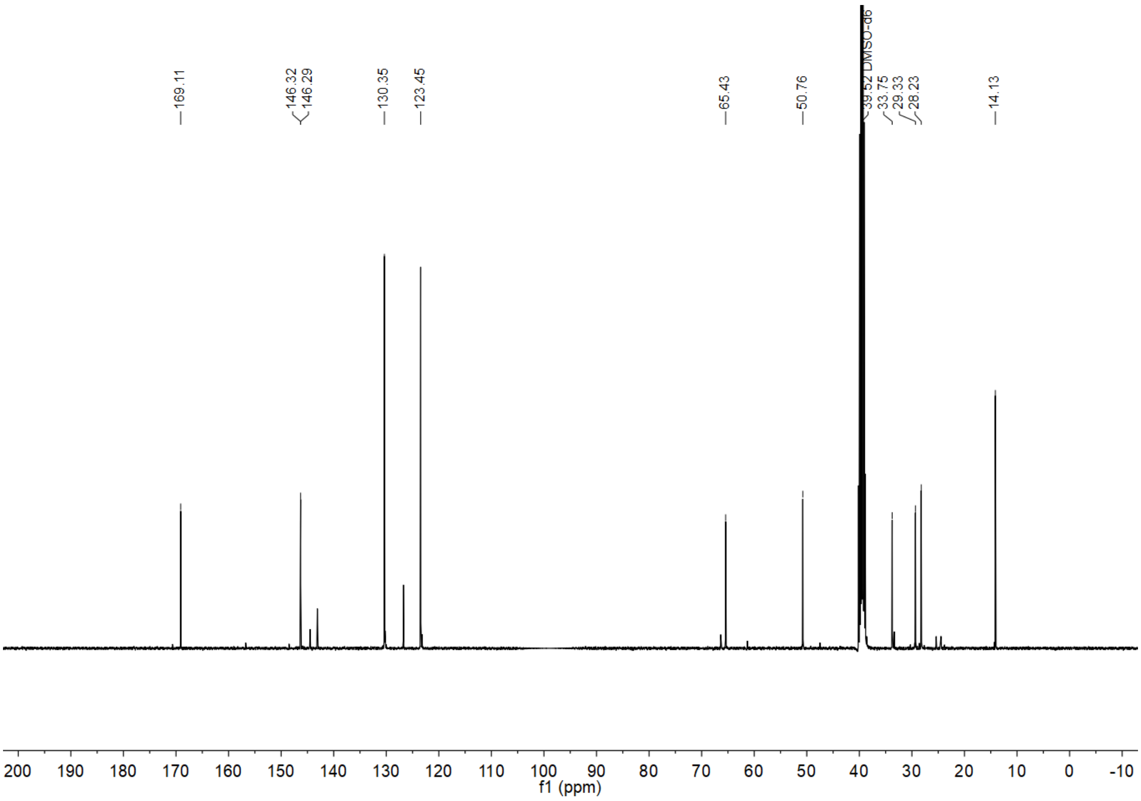

^{1}H and ^{13}C{^{1}H} NMR spectra of compound H-Gly-Gly-Onpe•HCl

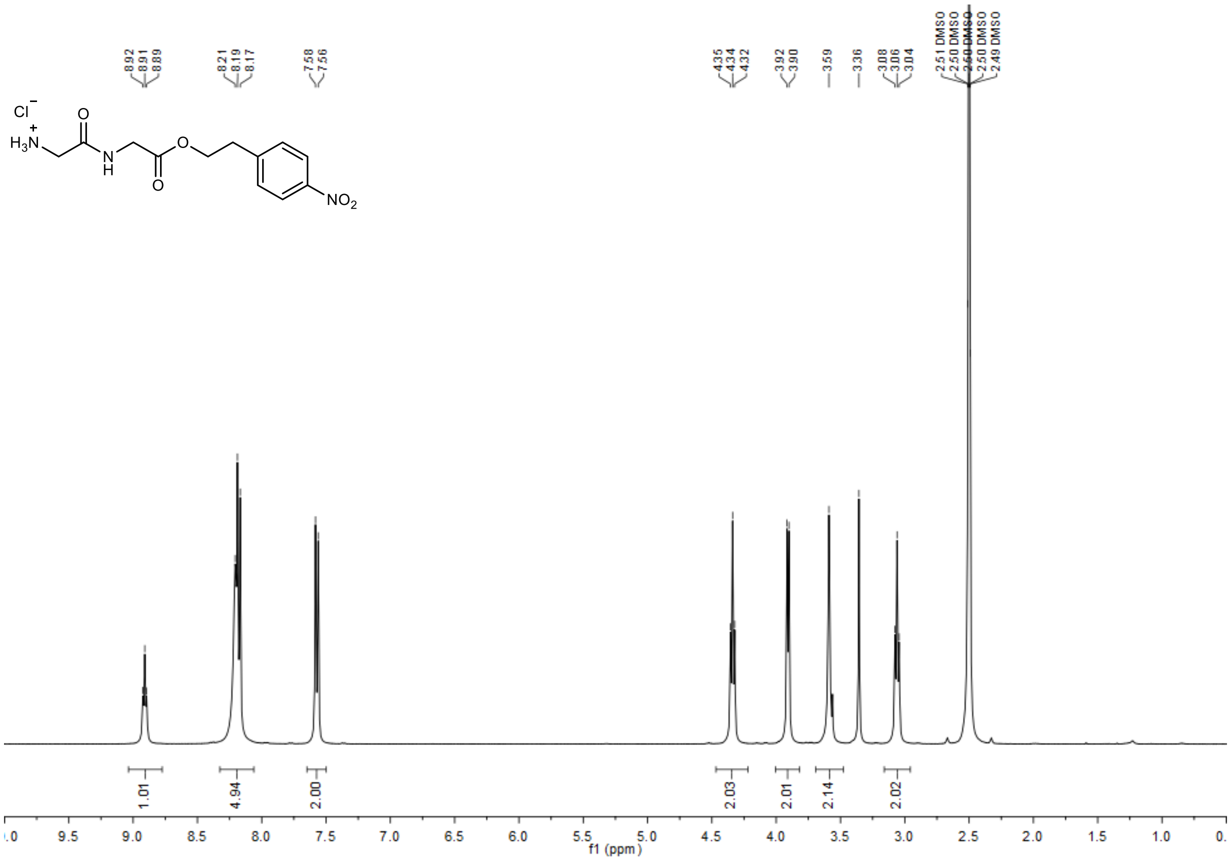

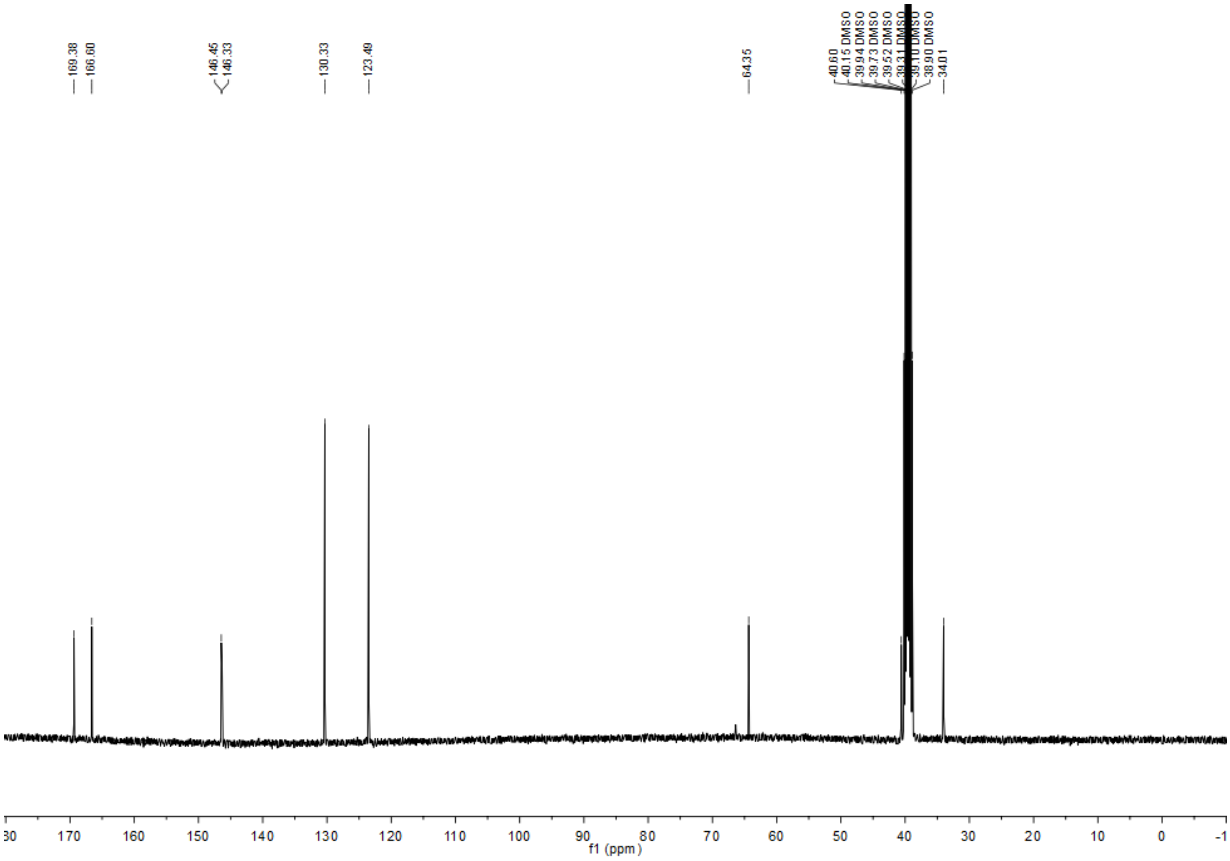

^{1}H and ^{13}C{^{1}H} NMR spectra of compound H-Ala-Gly-Gly-Gly-Onpe•HCl

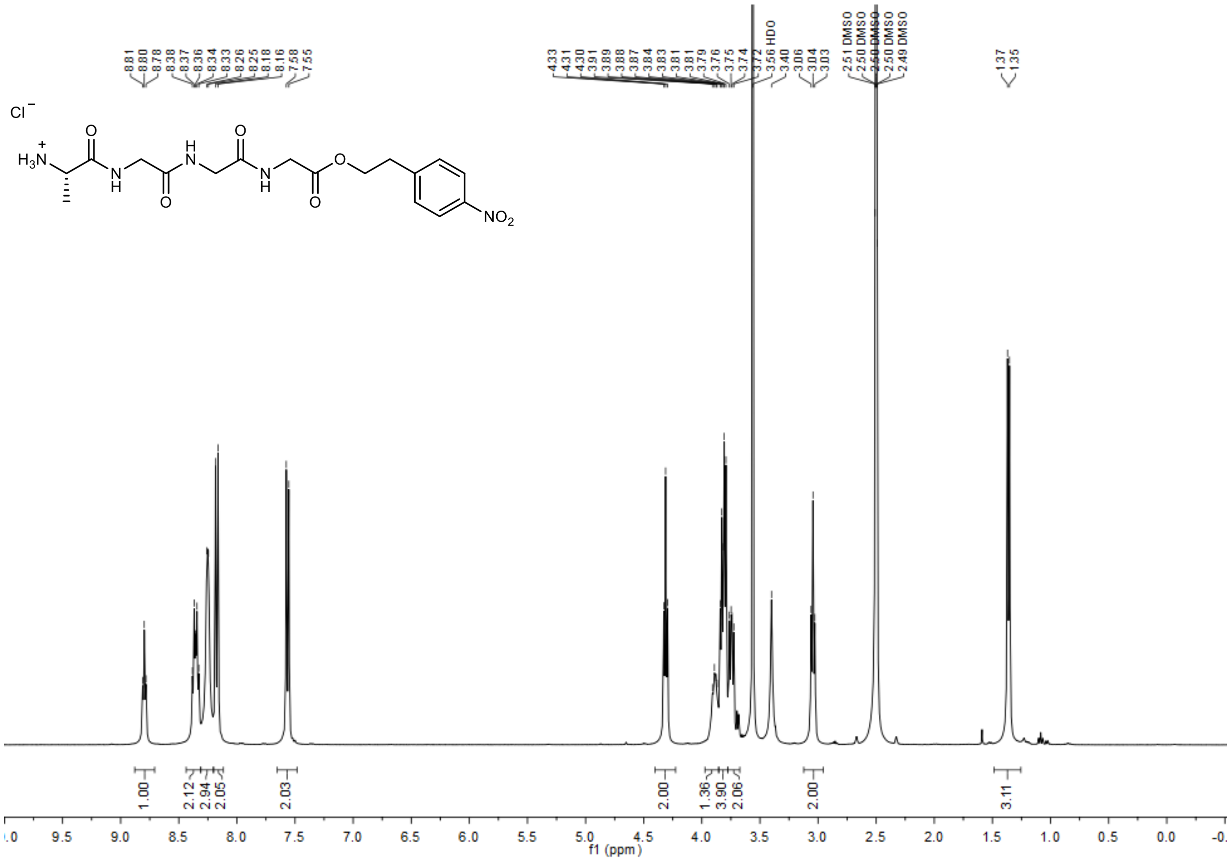

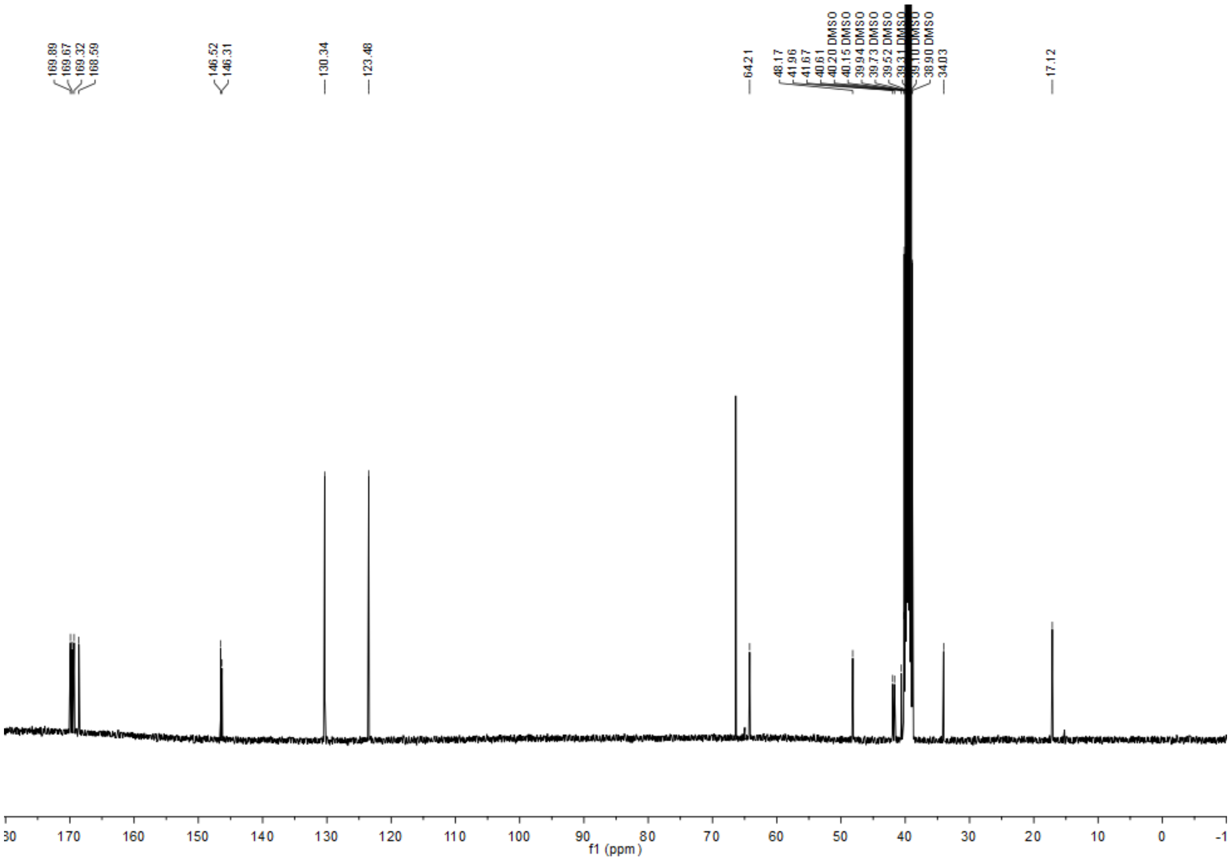

^{1}H and ^{13}C{^{1}H} NMR spectra of compound 10b

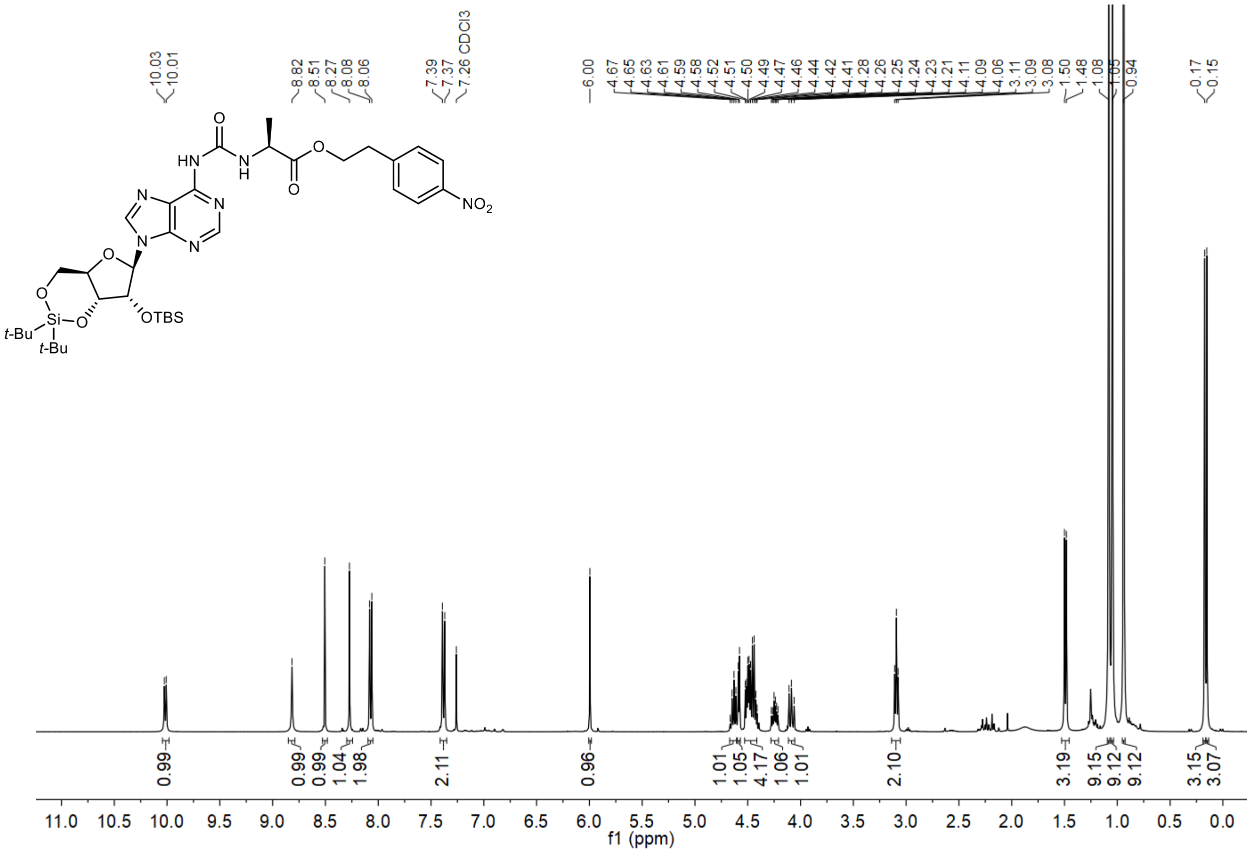

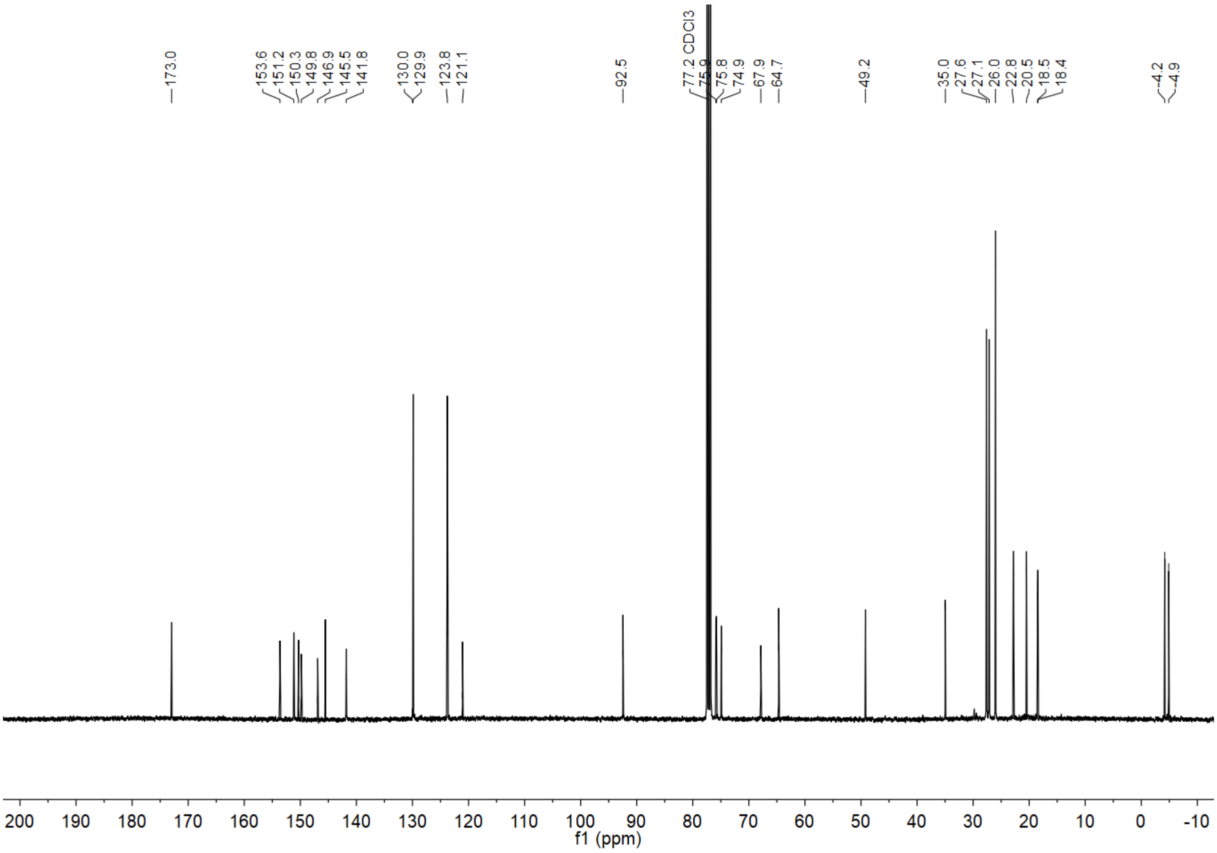

^{1}H and ^{13}C{^{1}H} NMR spectra of compound 10d

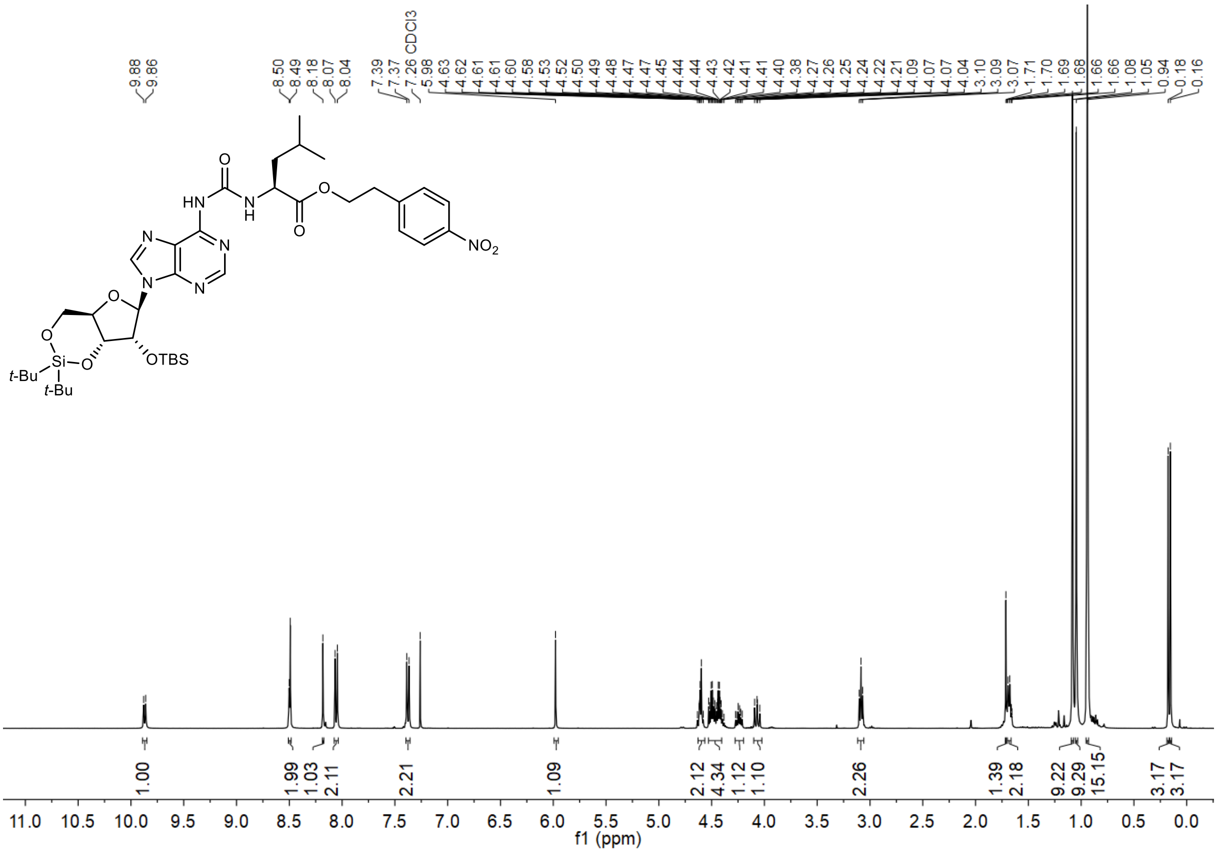

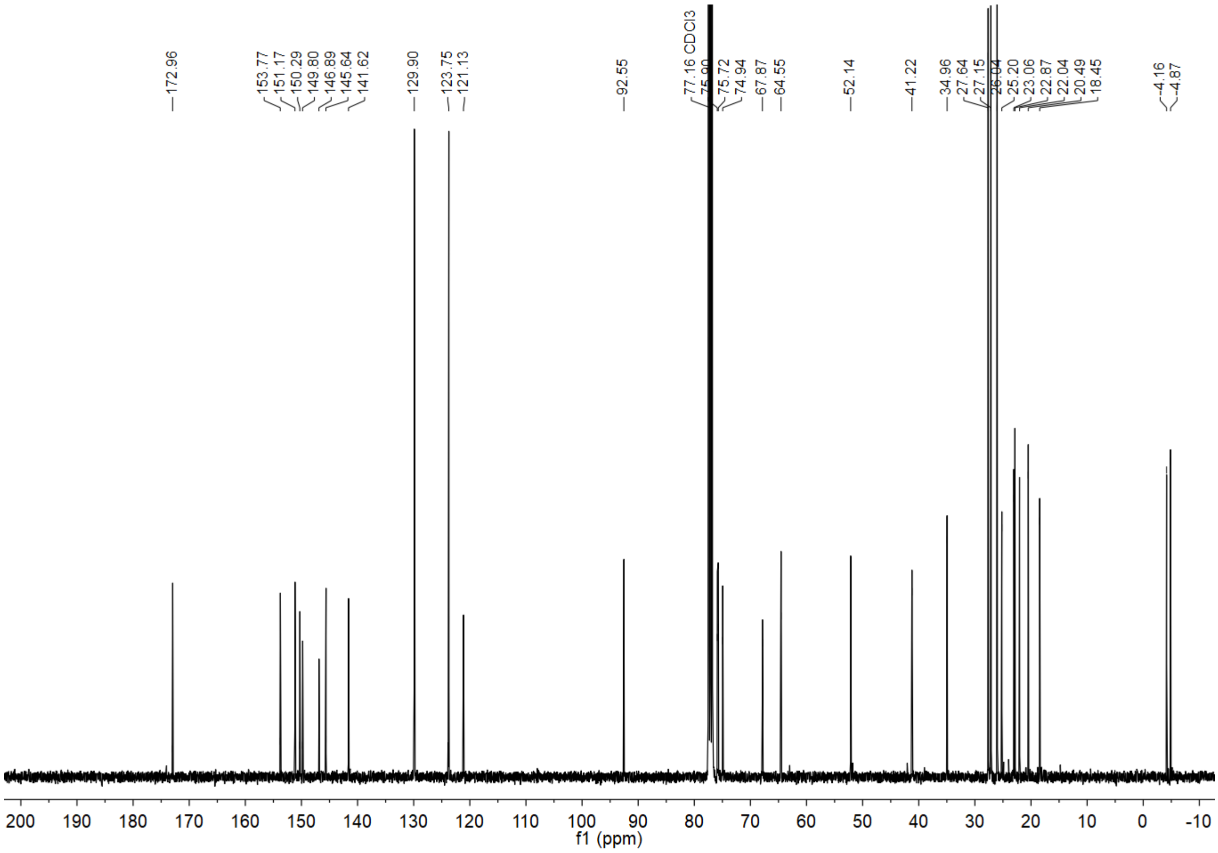

^{1}H and ^{13}C{^{1}H} NMR spectra of compound 10f

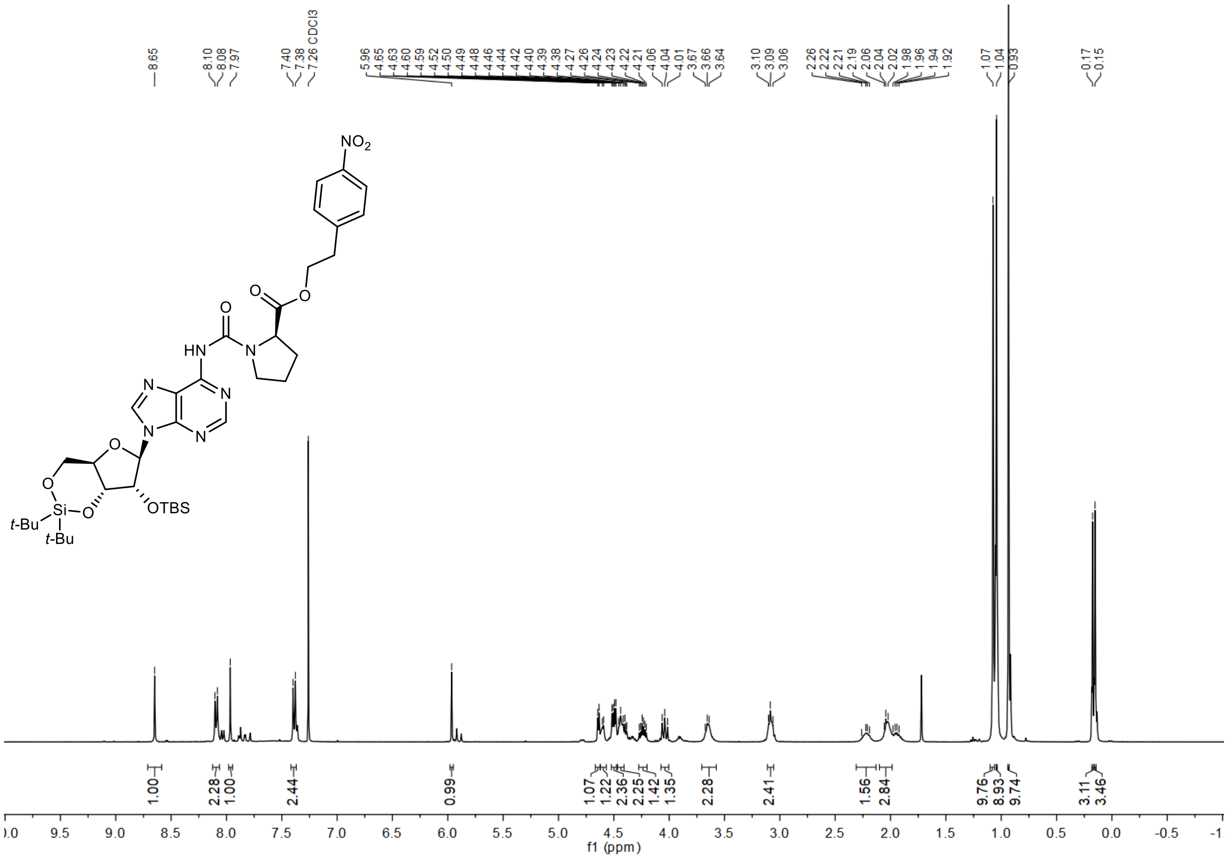

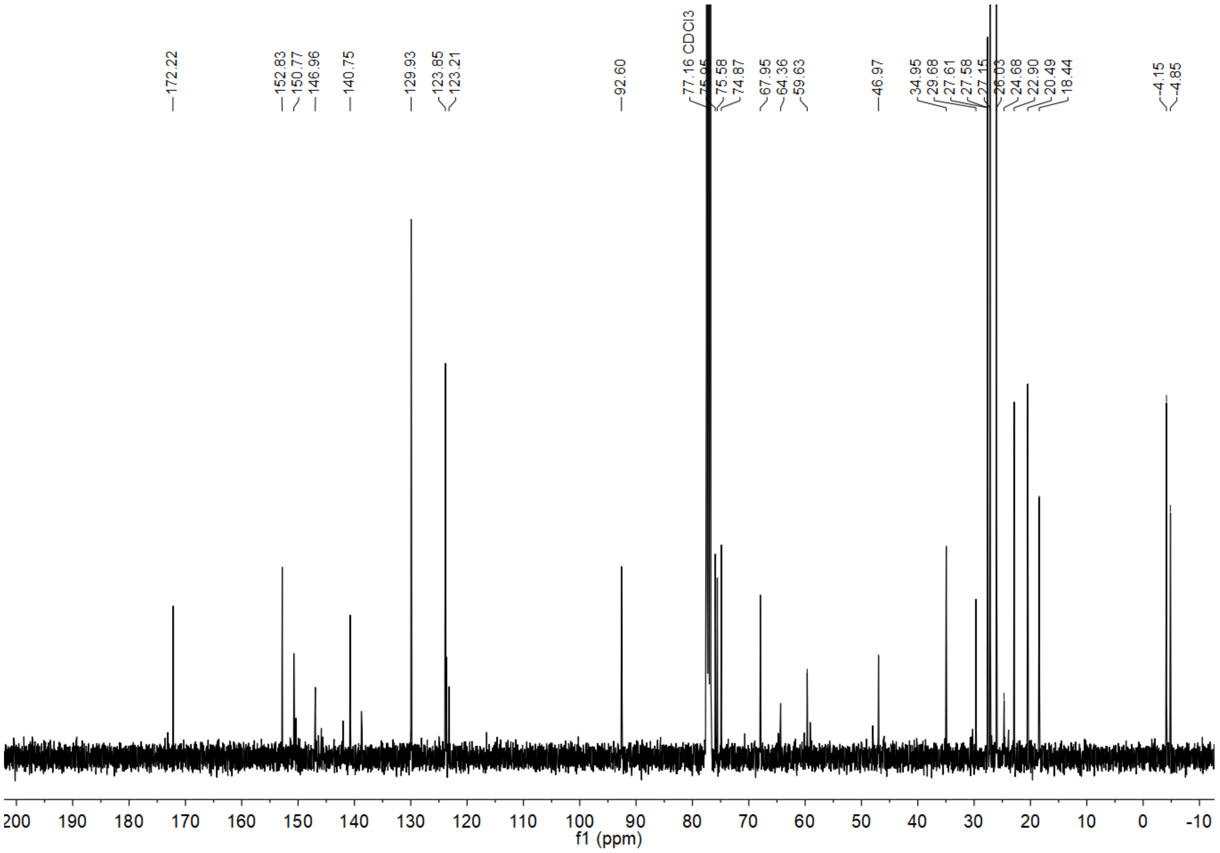

^{1}H and ^{13}C{^{1}H} NMR spectra of compound 10h

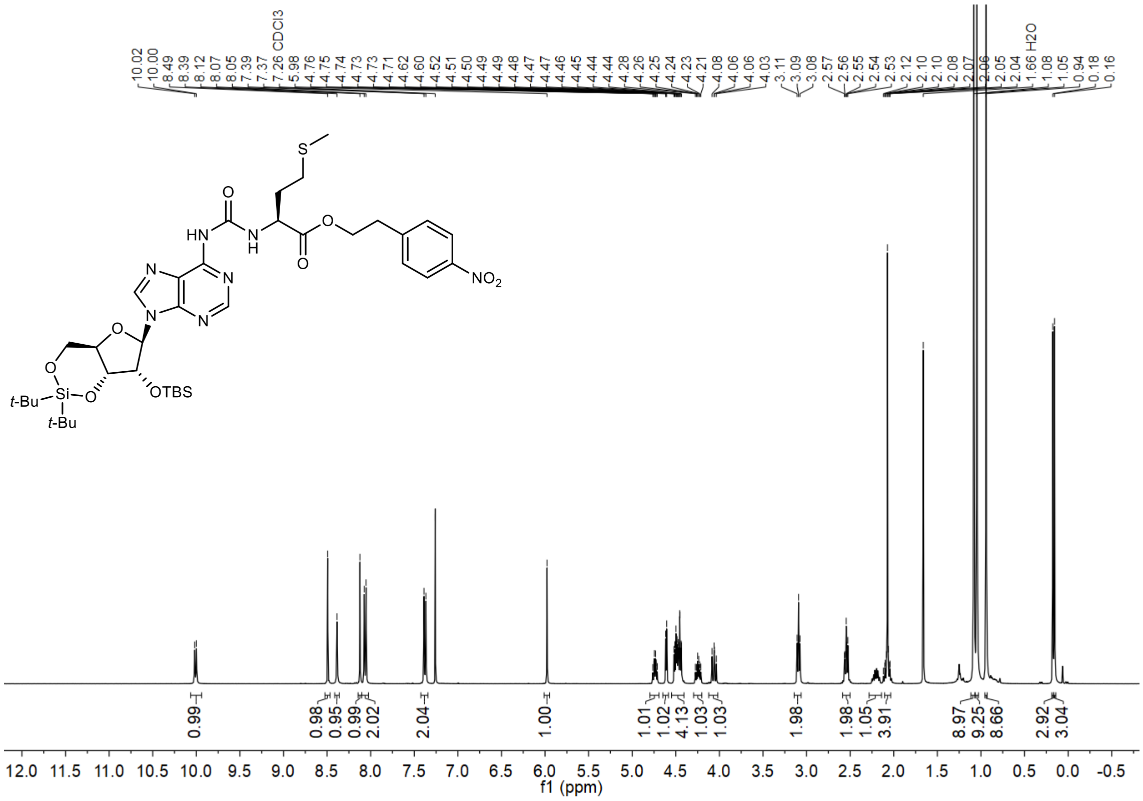

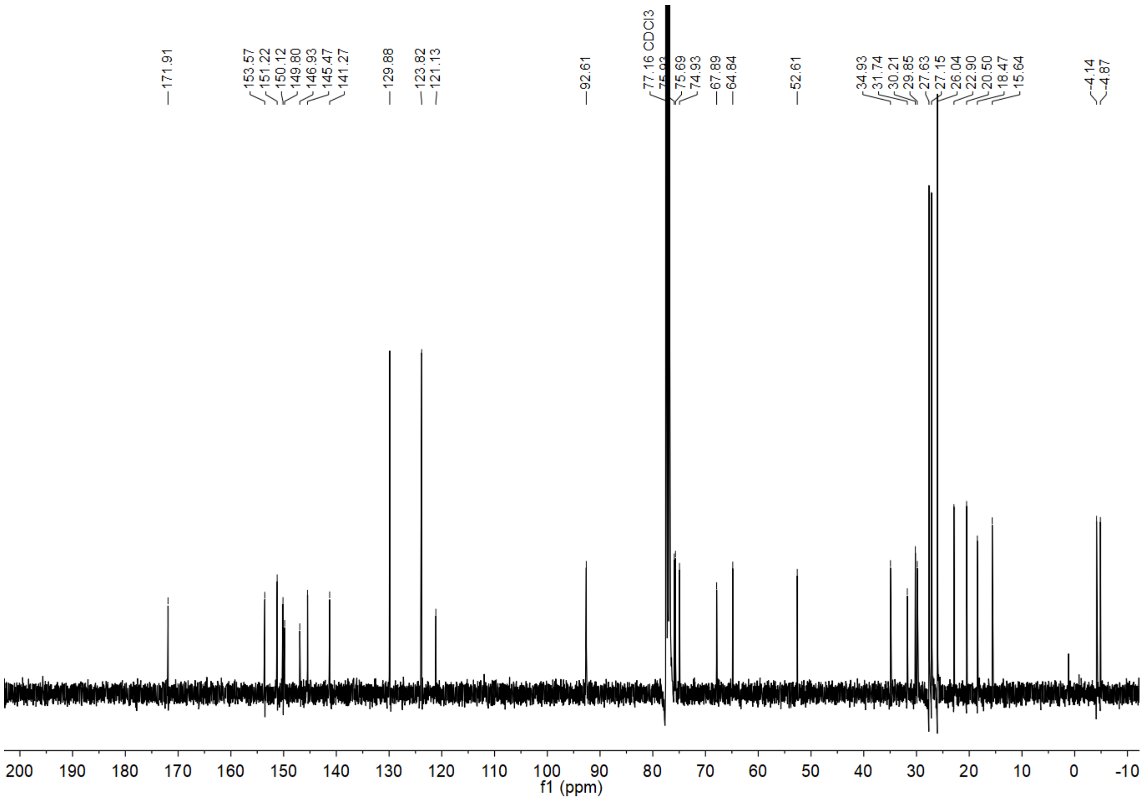

^{1}H and ^{13}C{^{1}H} NMR spectra of compound 10j

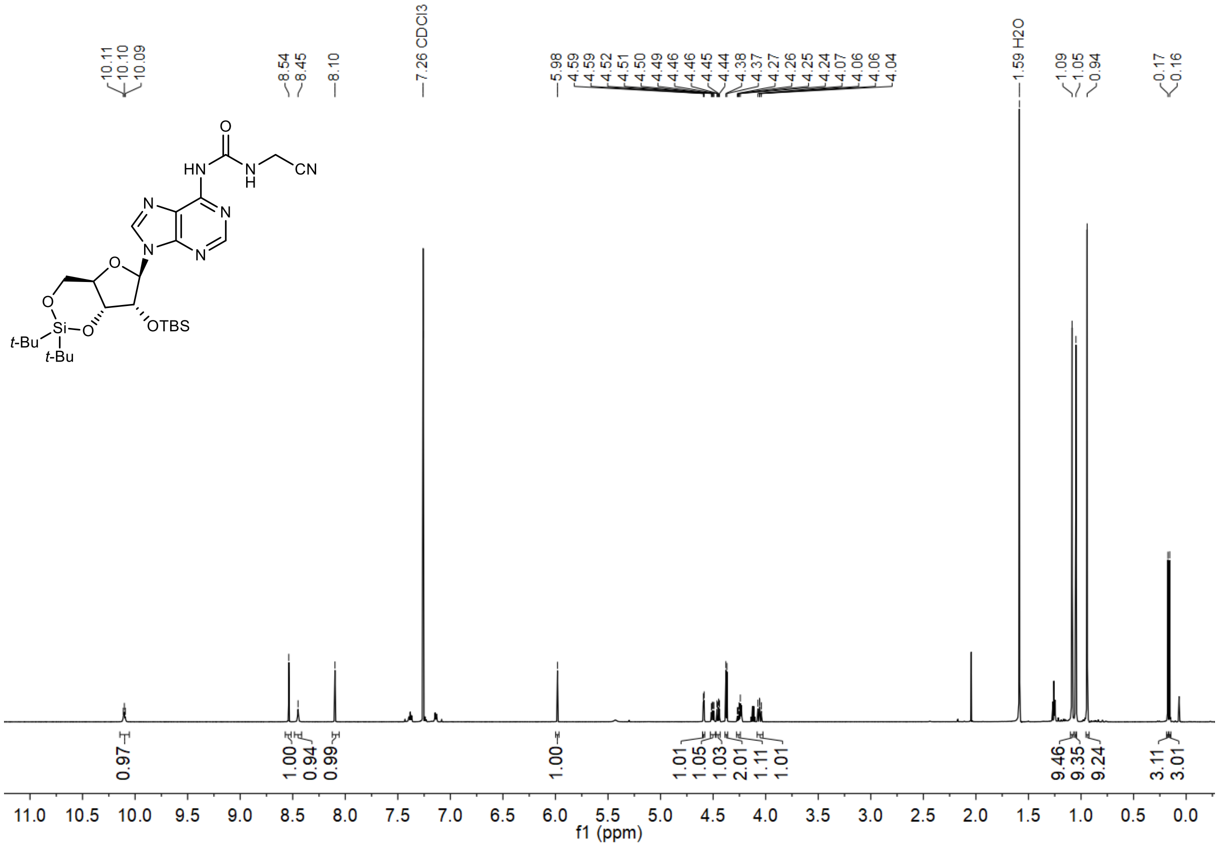

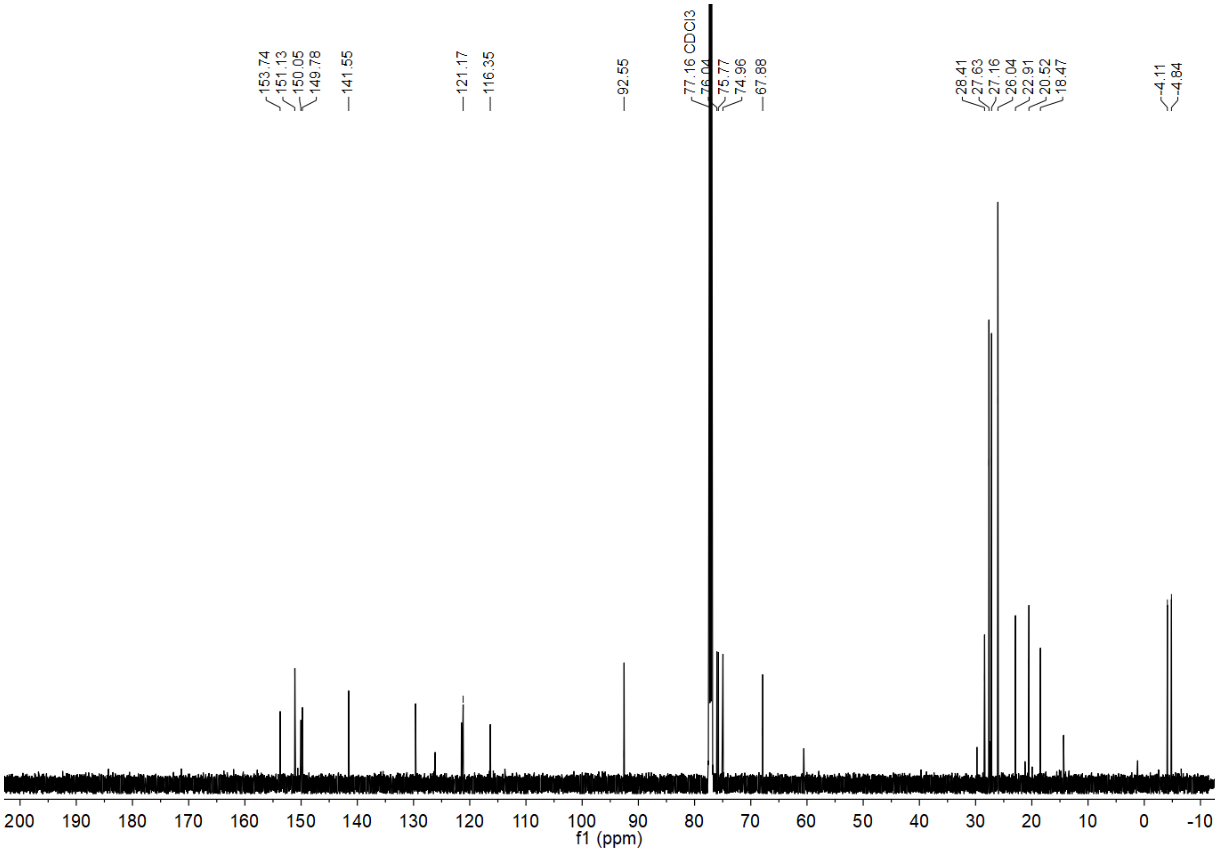

^{1}H and ^{13}C{^{1}H} NMR spectra of compound 11a

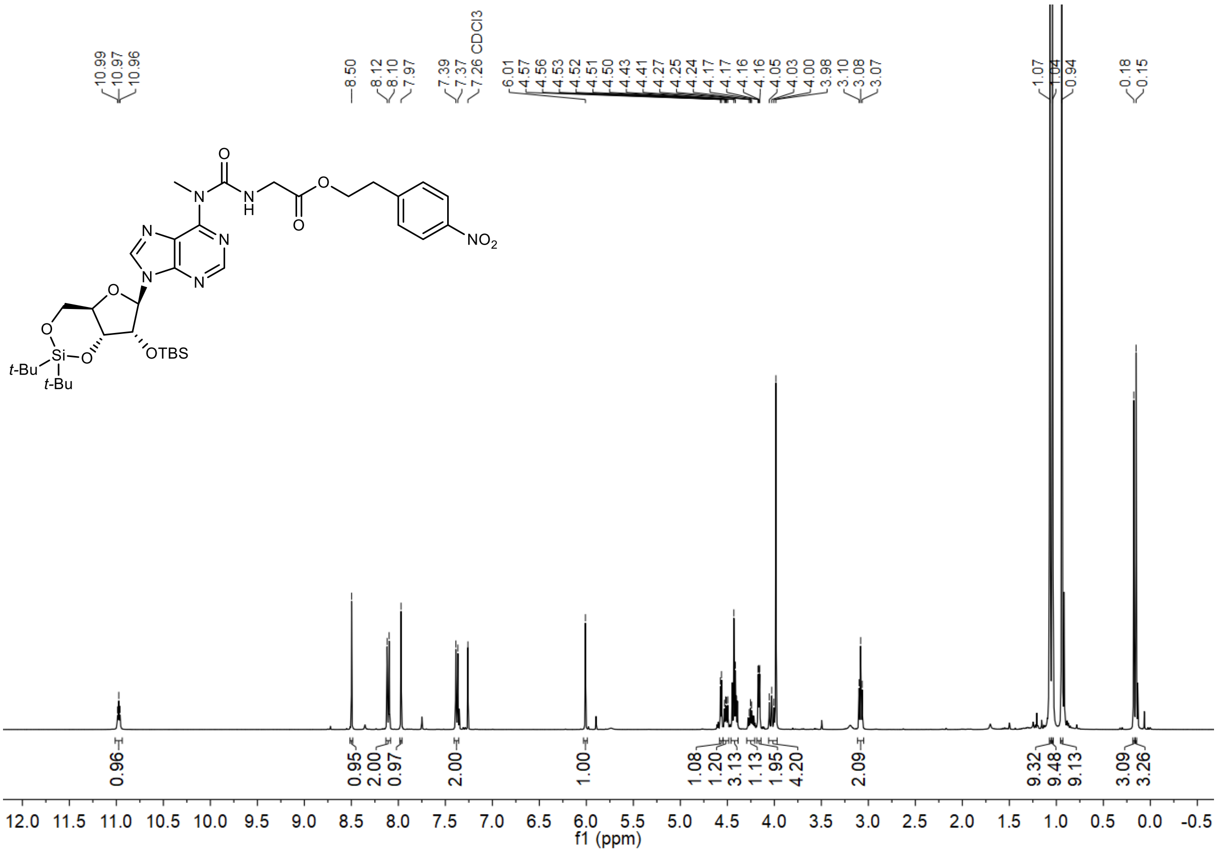

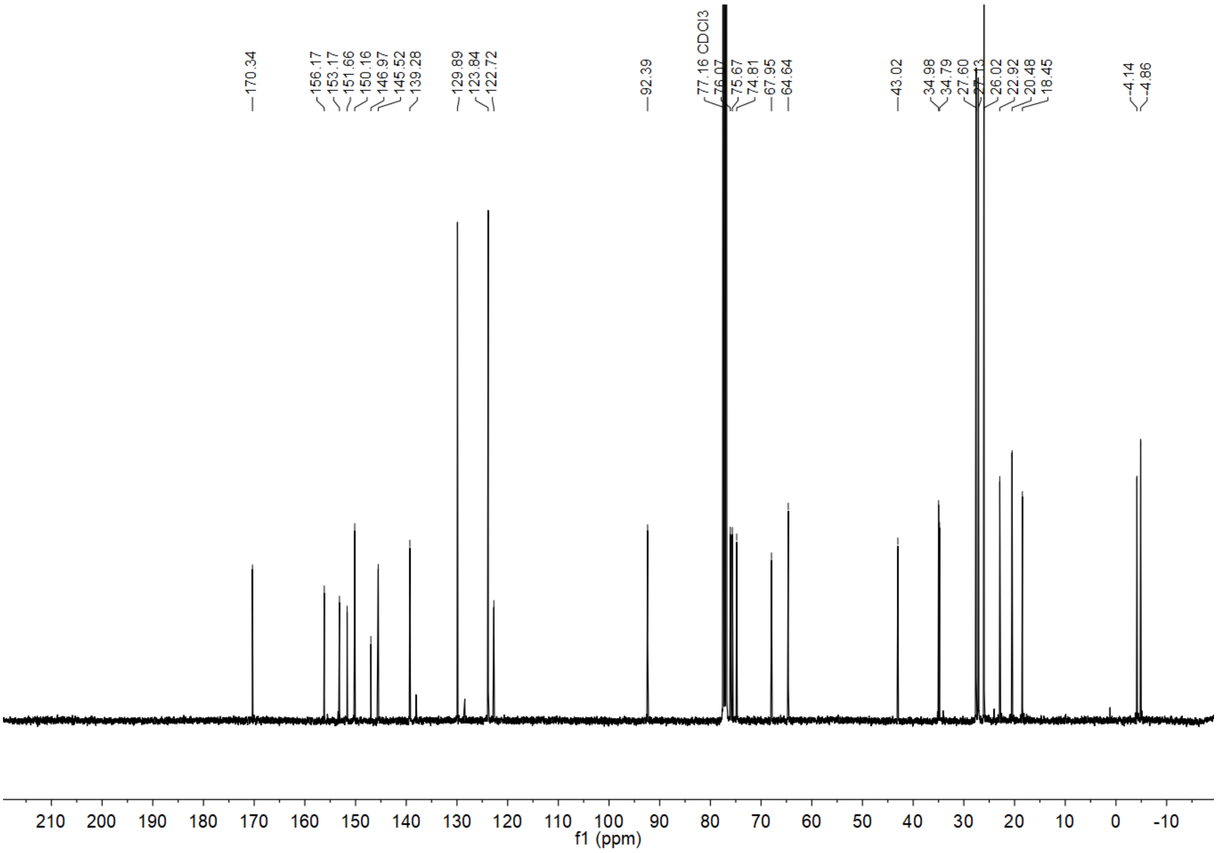

^{1}H and ^{13}C{^{1}H} NMR spectra of compound 11b

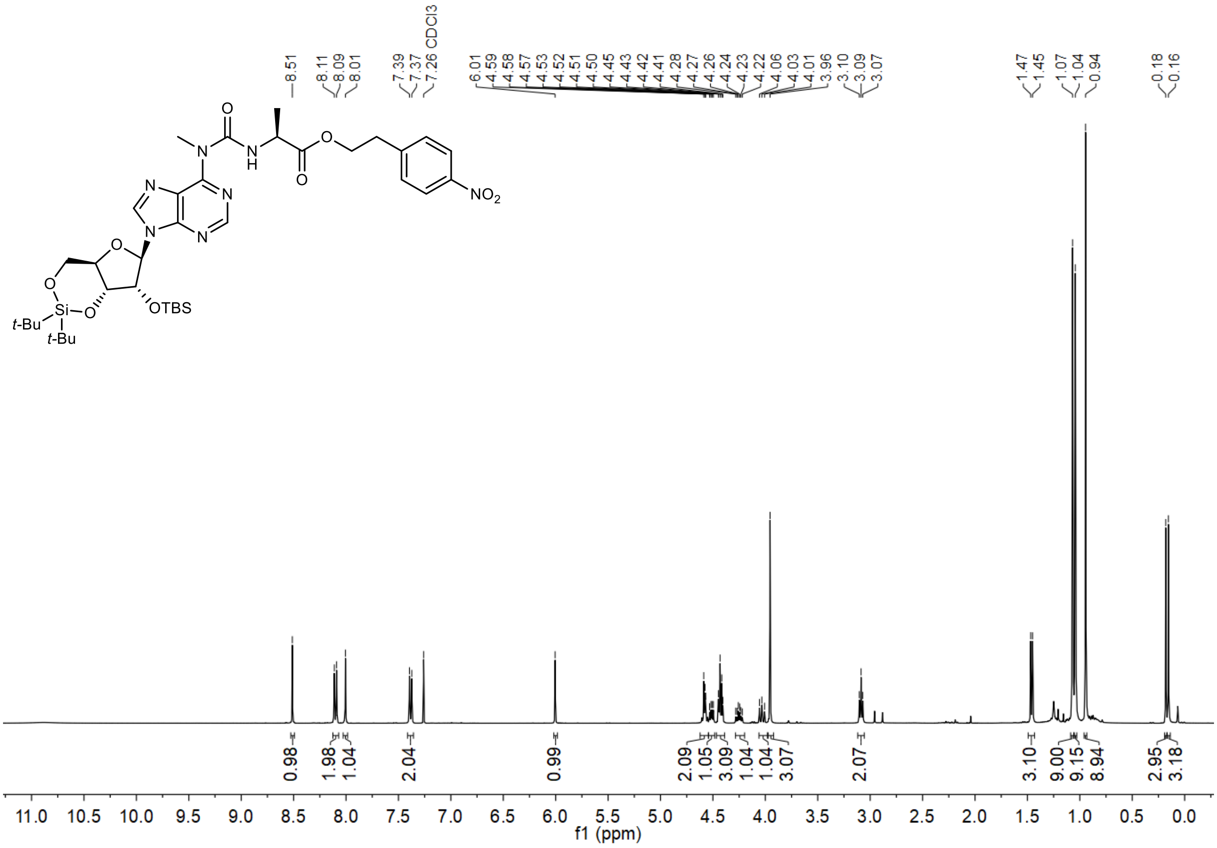

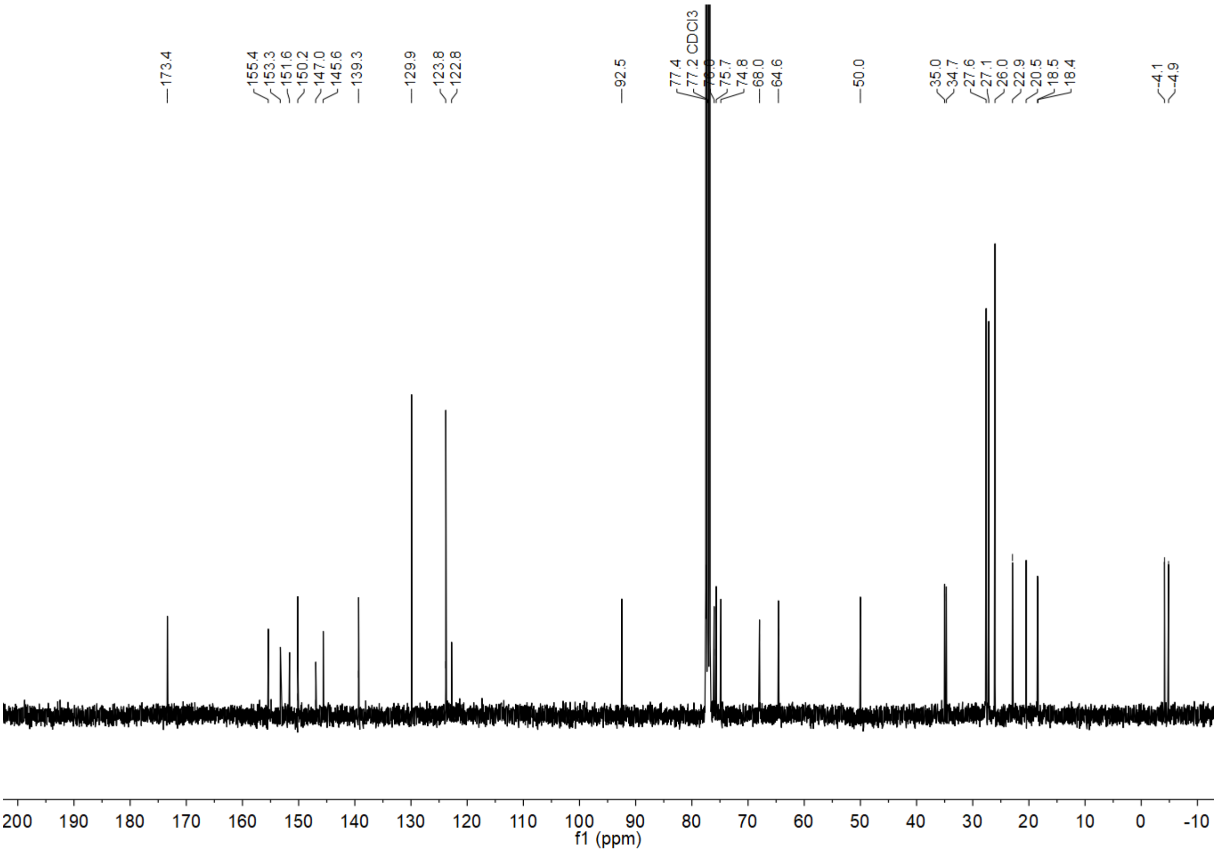

^{1}H and ^{13}C{^{1}H} NMR spectra of compound 11c

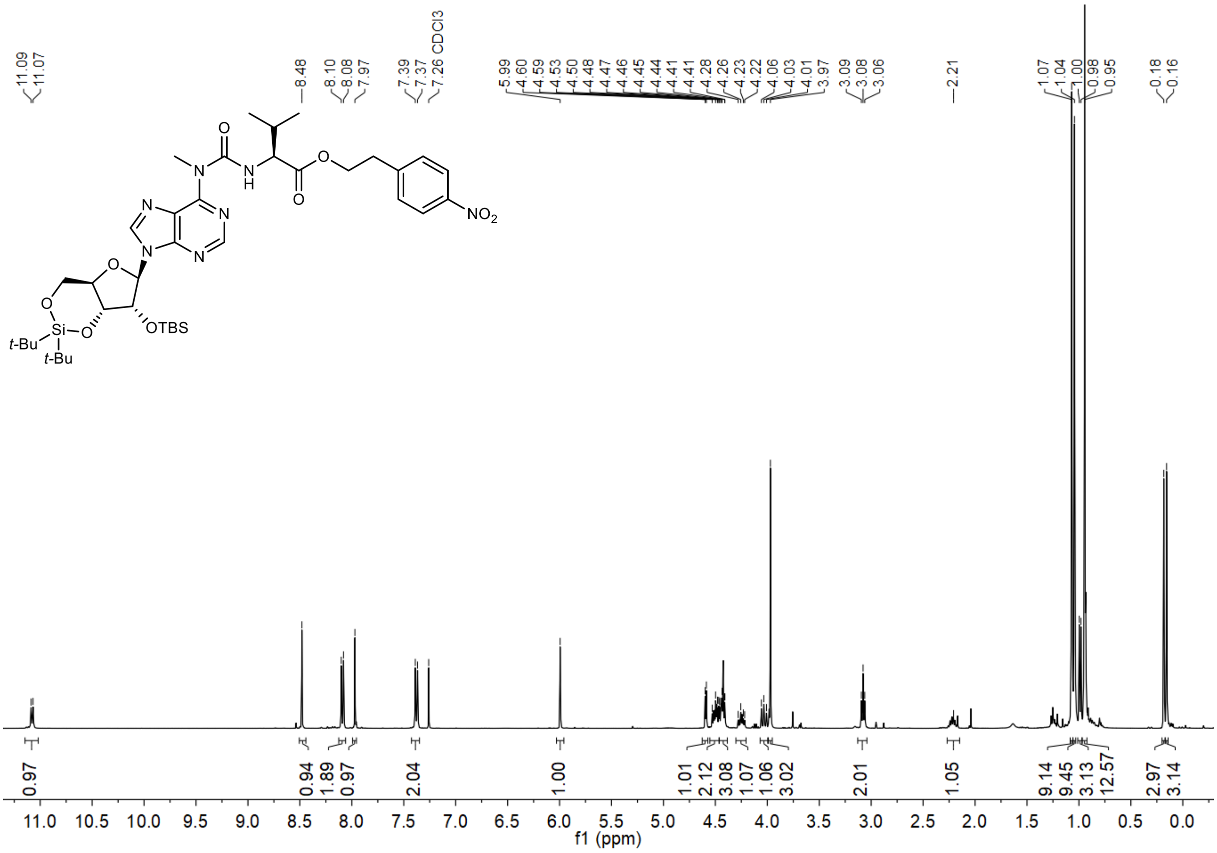

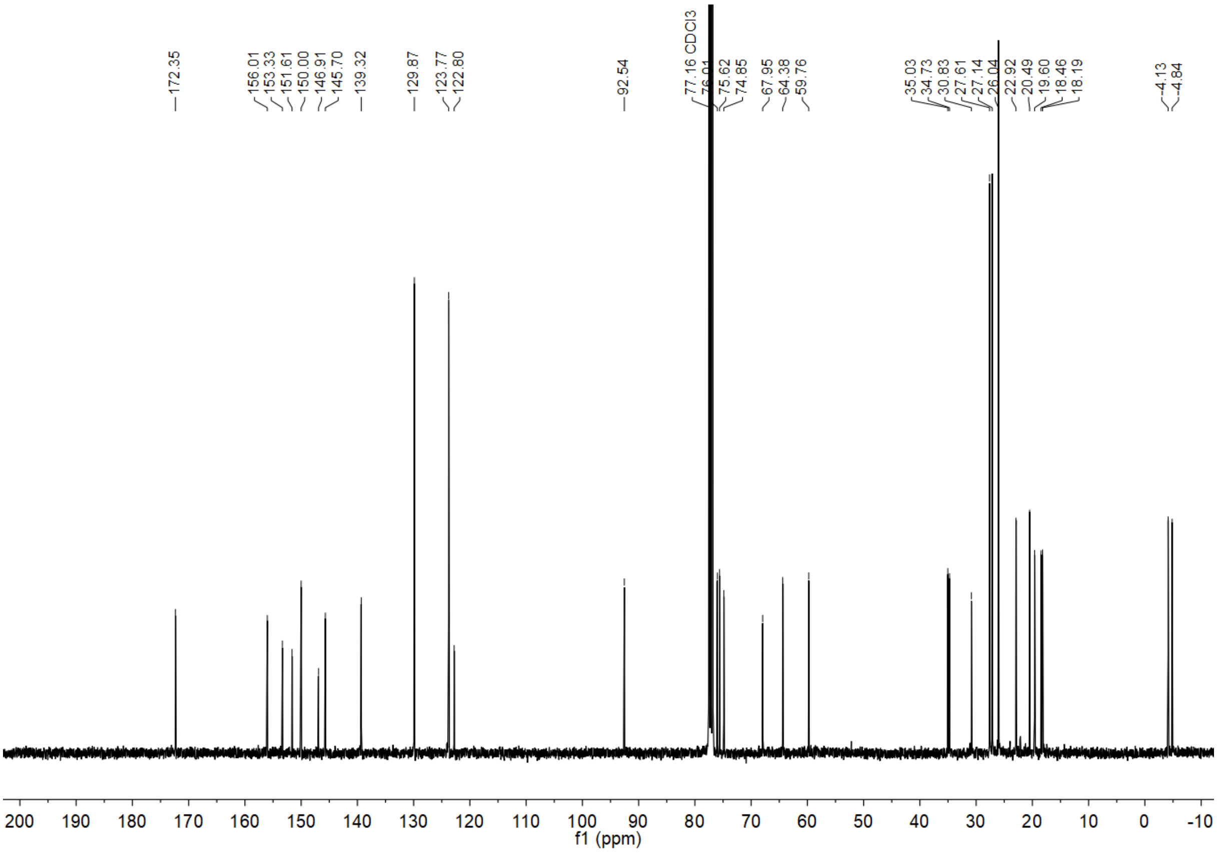

^{1}H and ^{13}C{^{1}H} NMR spectra of compound 11d

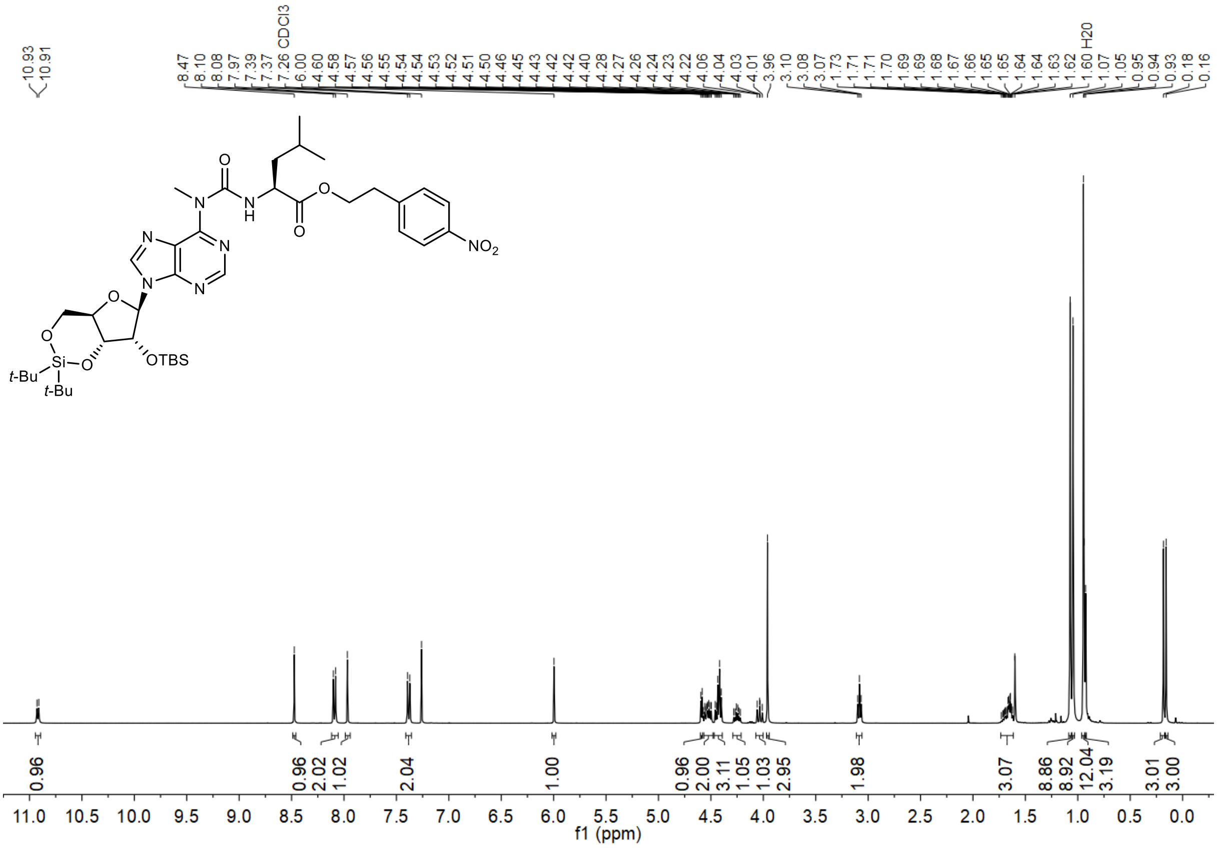

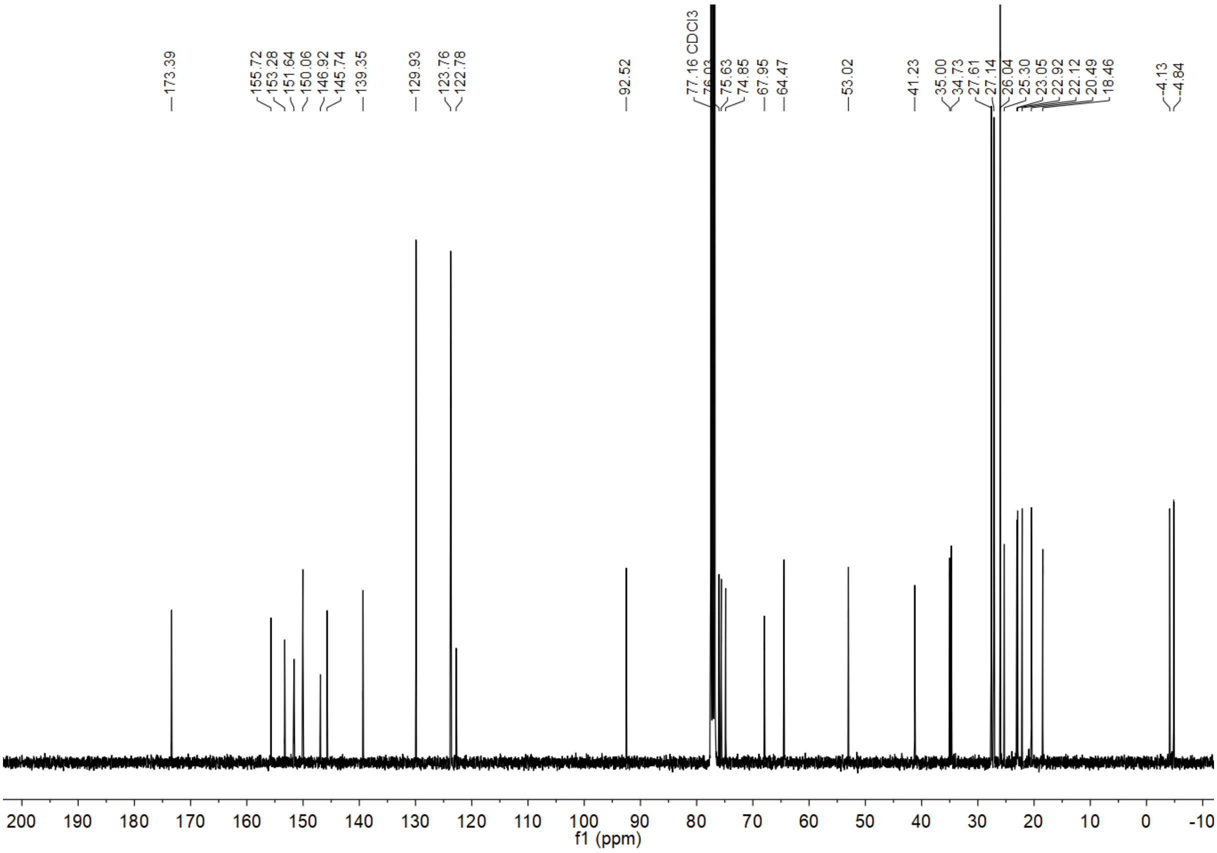

^{1}H and $^{13}C\{^{1}H\}$ NMR spectra of compound 11e

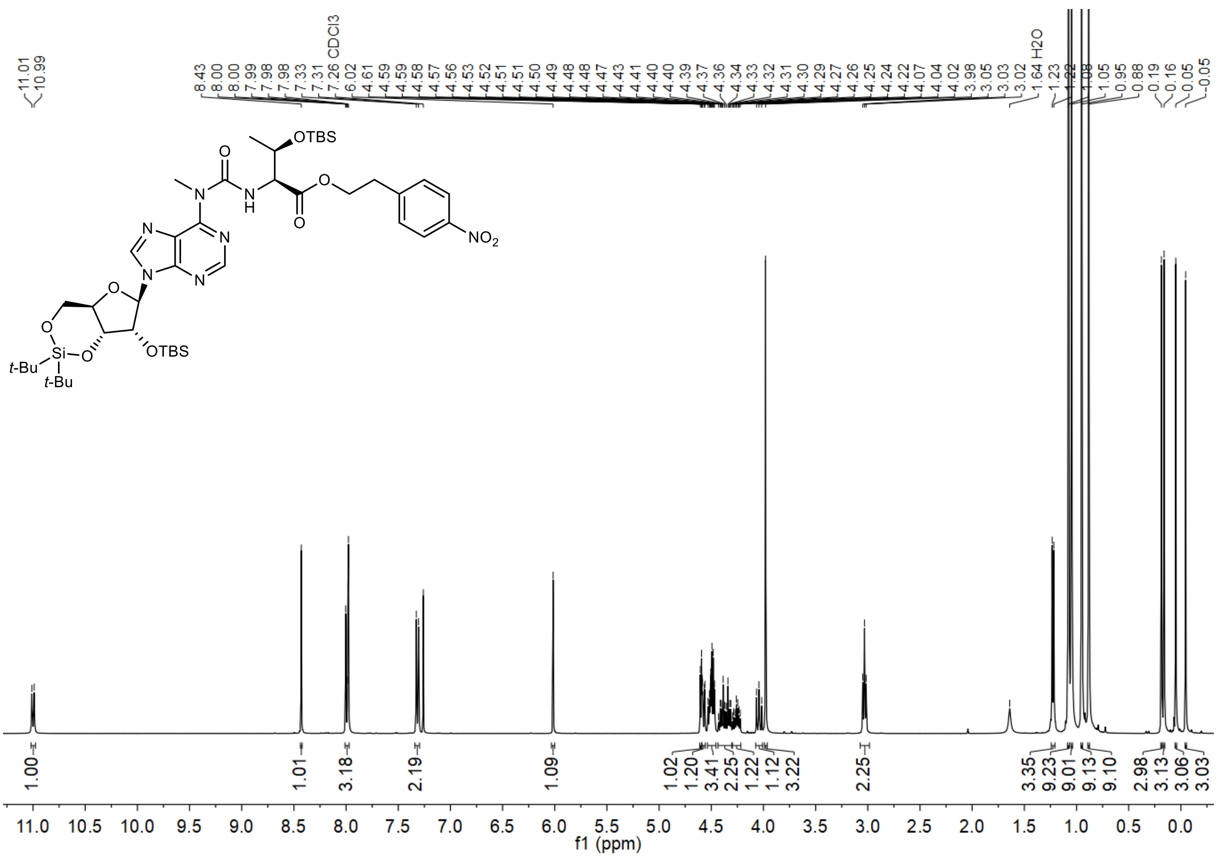

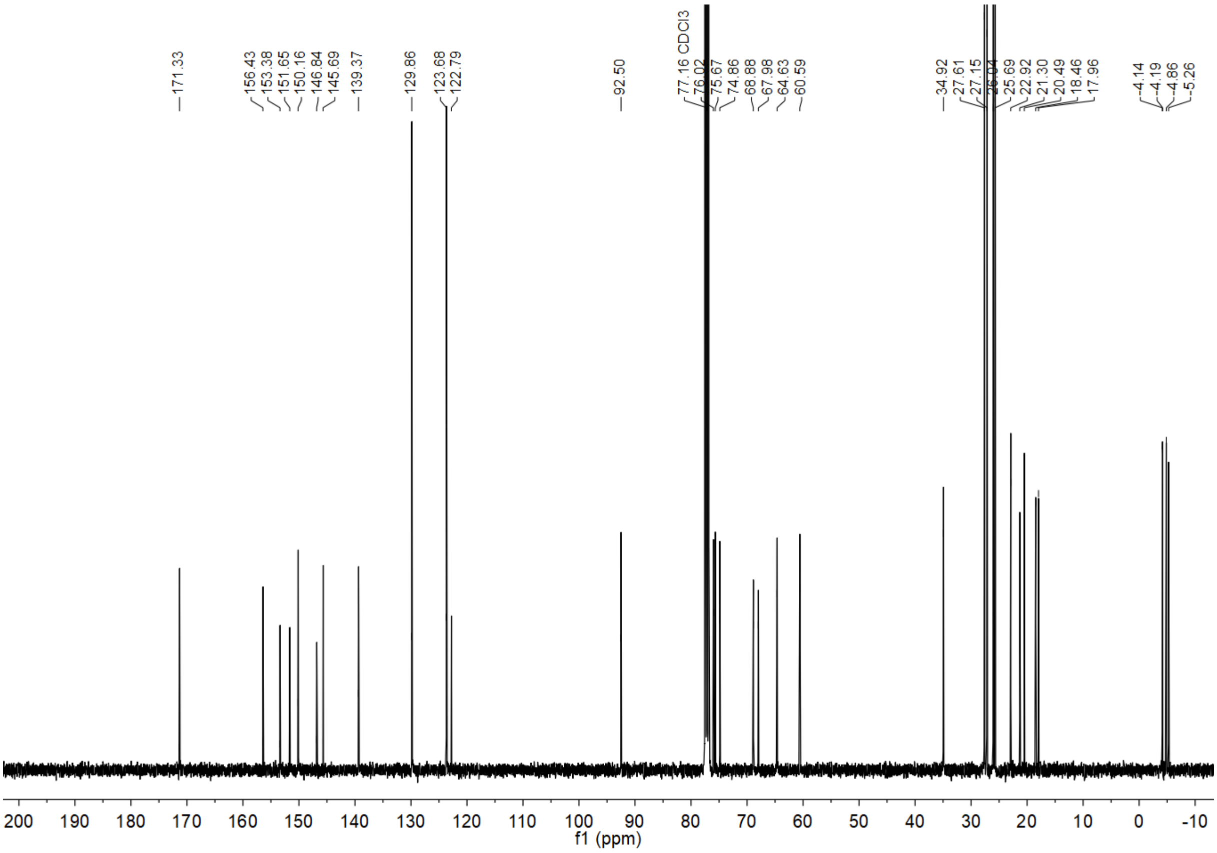

^{1}H and ^{13}C{^{1}H} NMR spectra of compound 11f

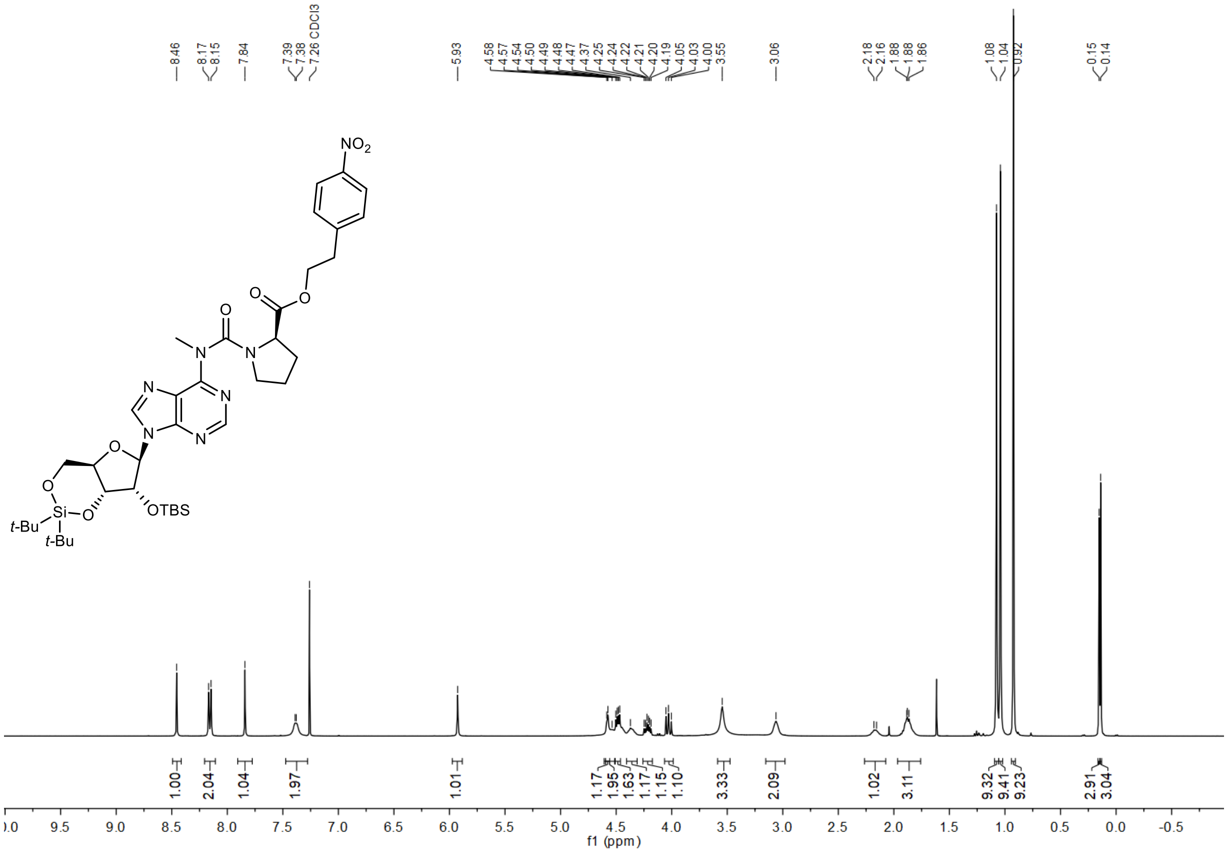

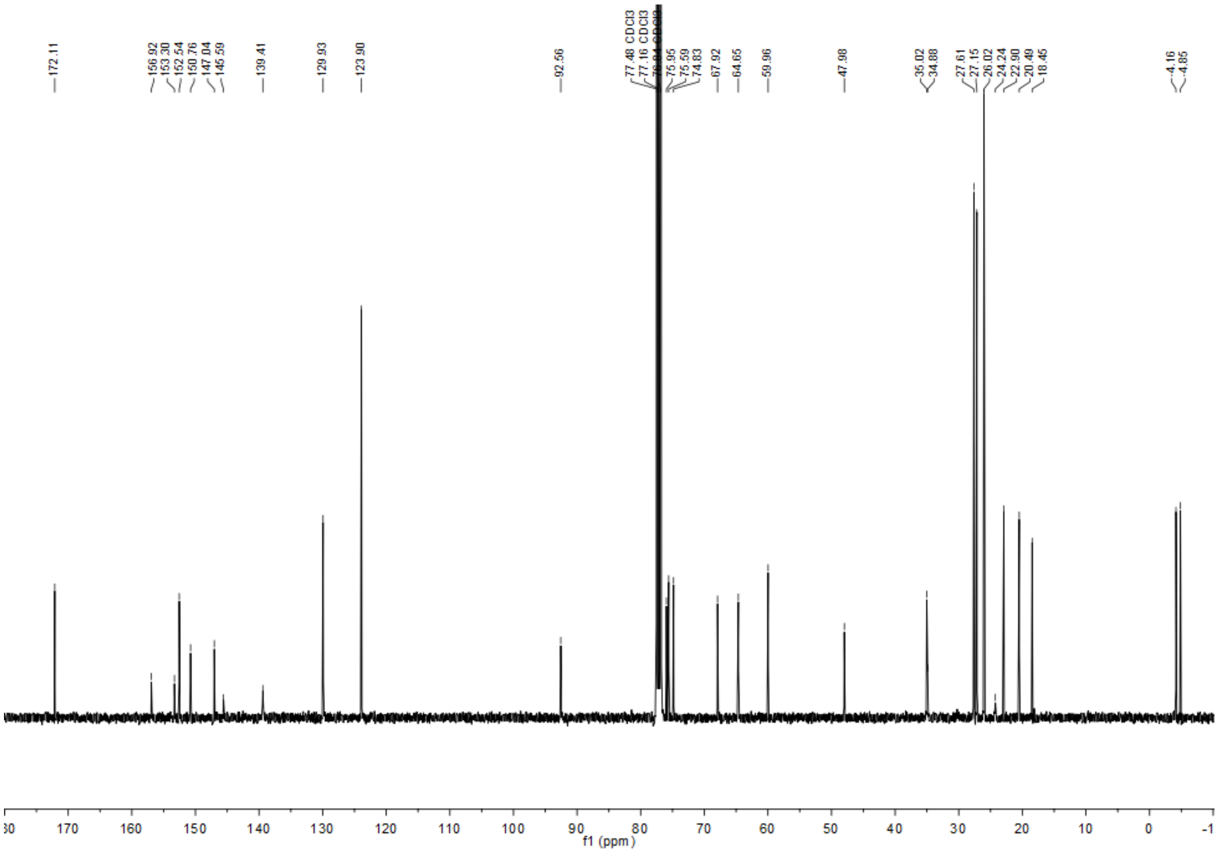

^{1}H and ^{13}C{^{1}H} NMR spectra of compound 11g

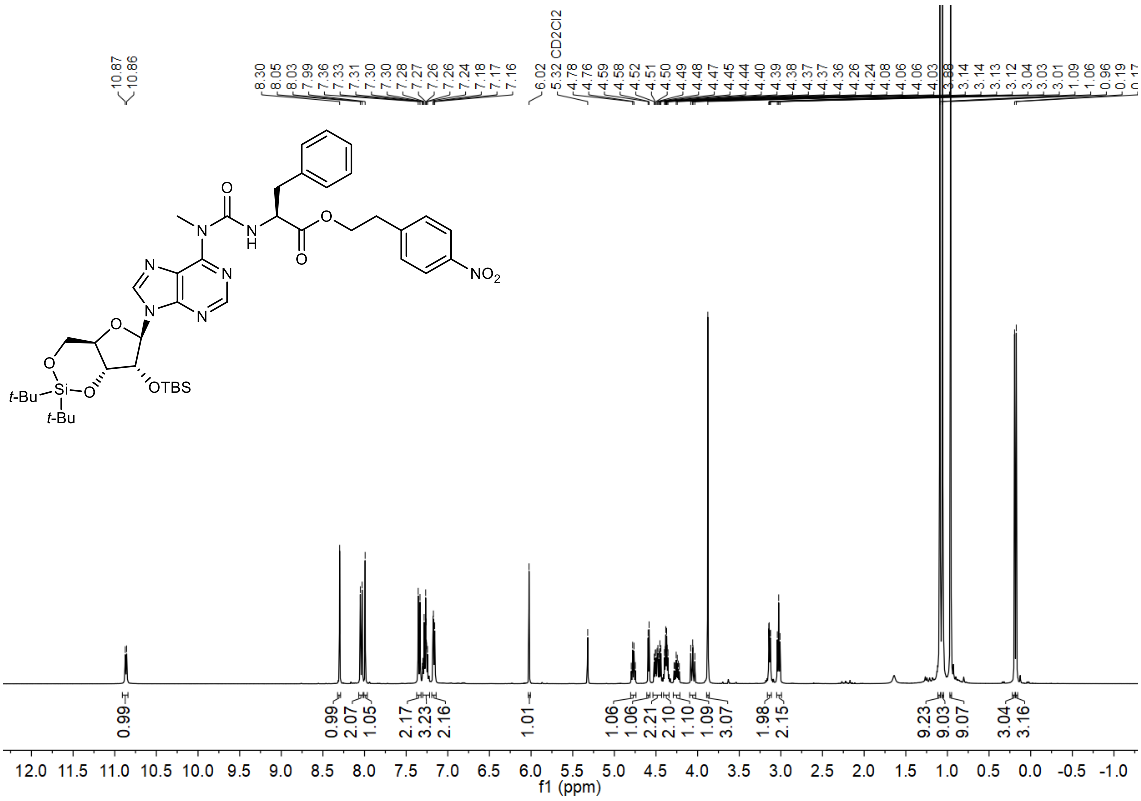

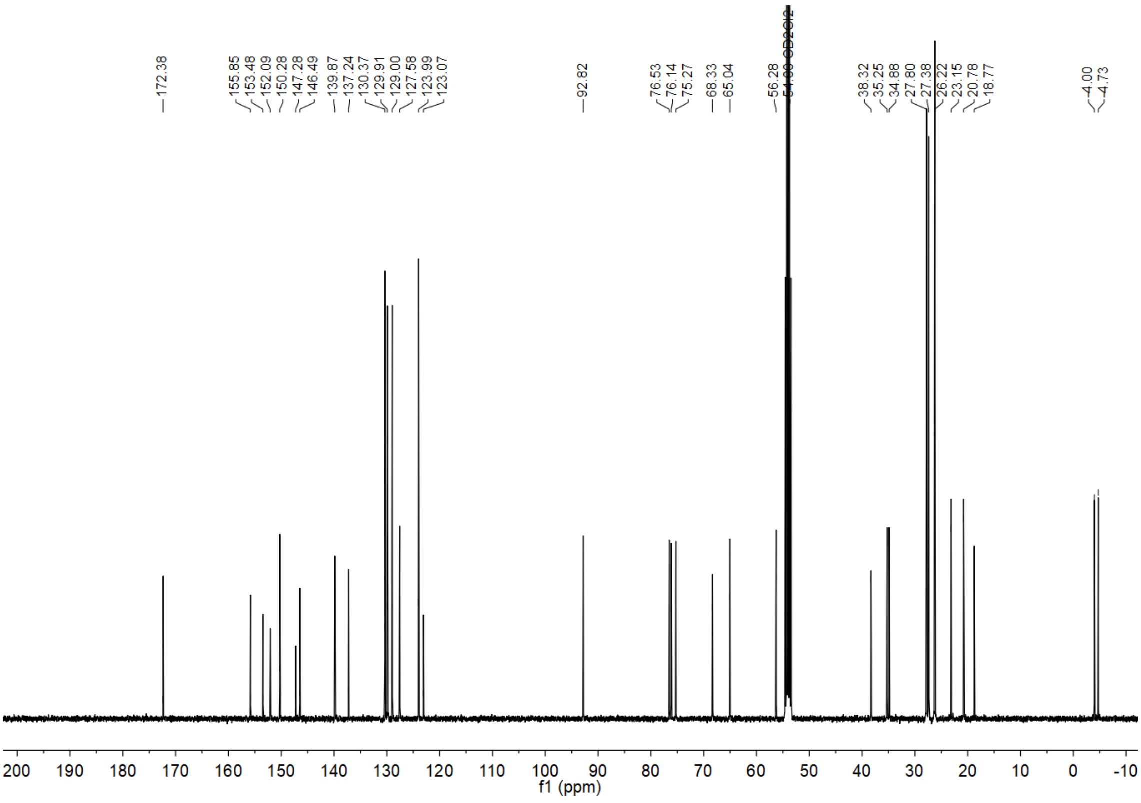

^{1}H and ^{13}C{^{1}H} NMR spectra of compound 11h

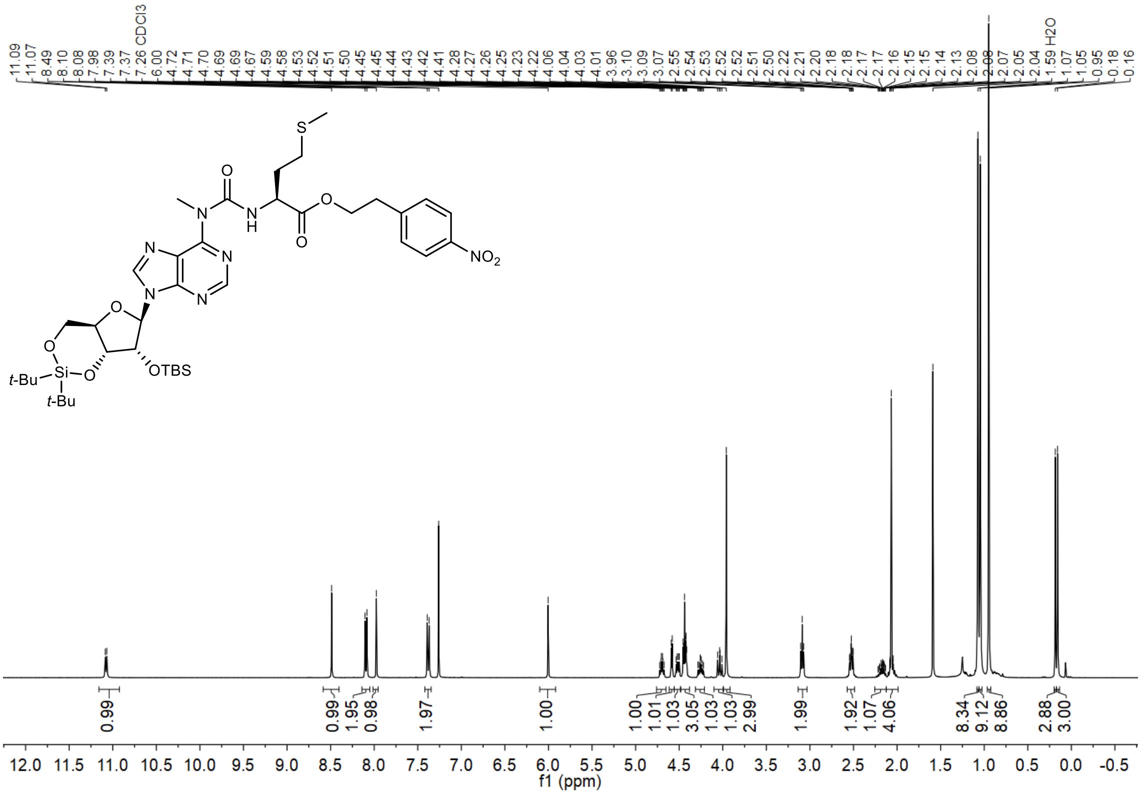

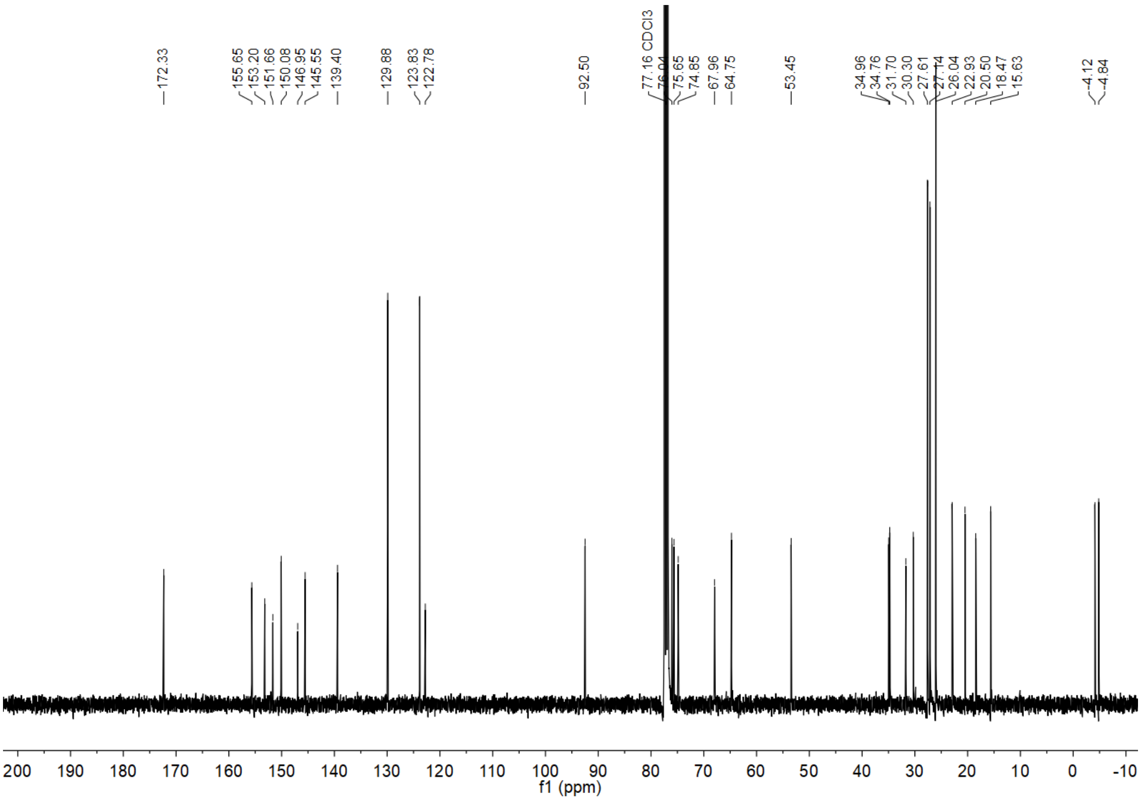

^{1}H and ^{13}C{^{1}H} NMR spectra of compound 11i

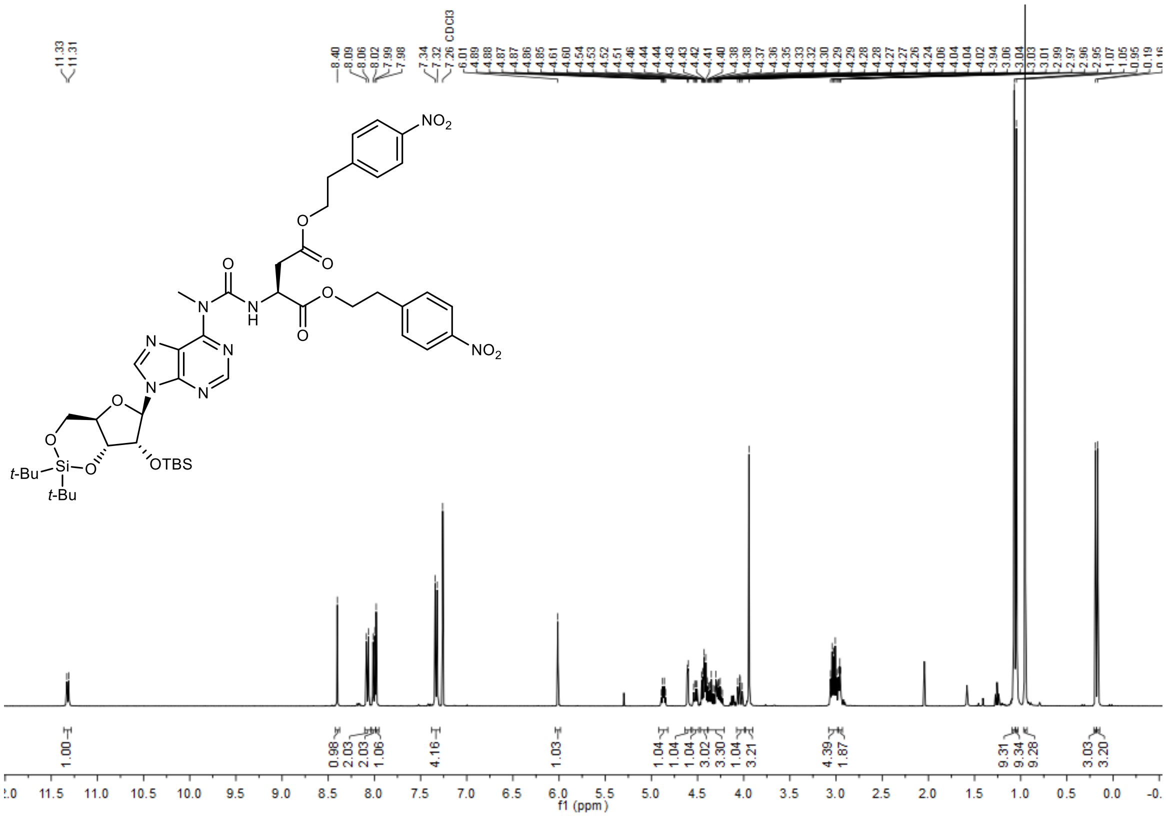

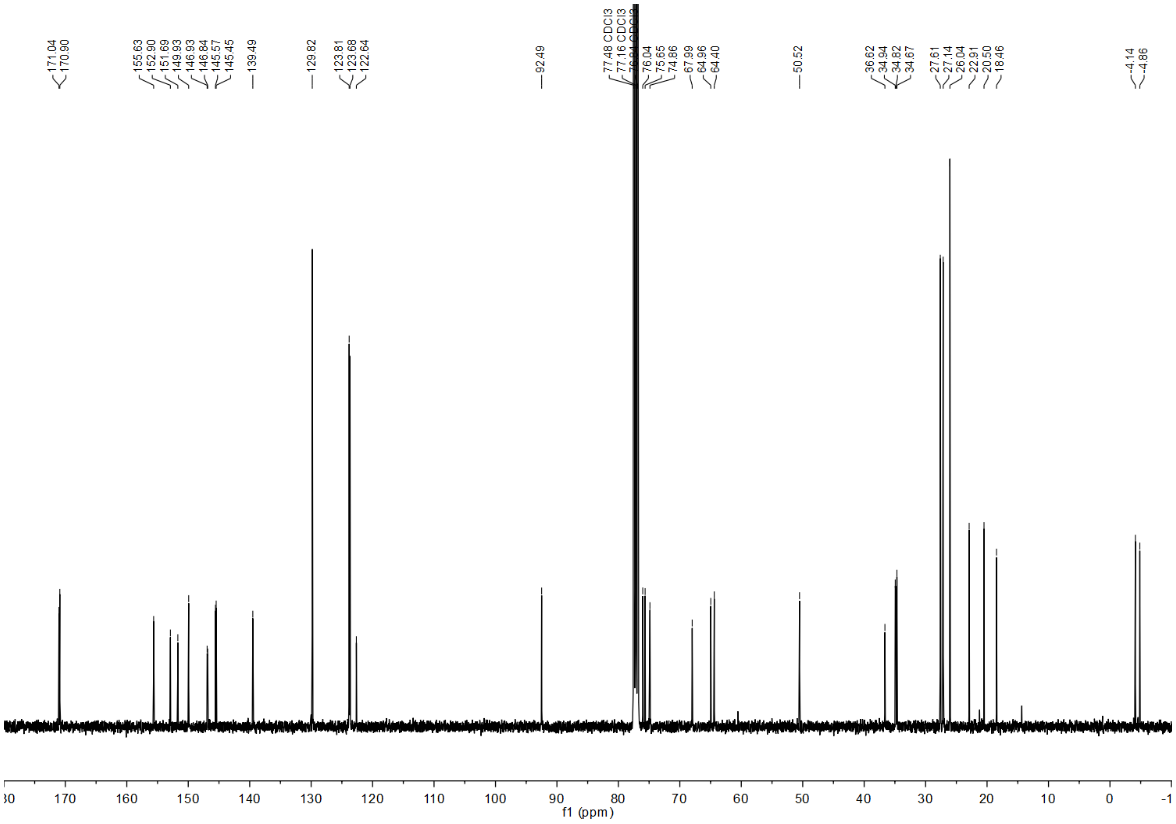

^{1}H and ^{13}C{^{1}H} NMR spectra of compound 11j

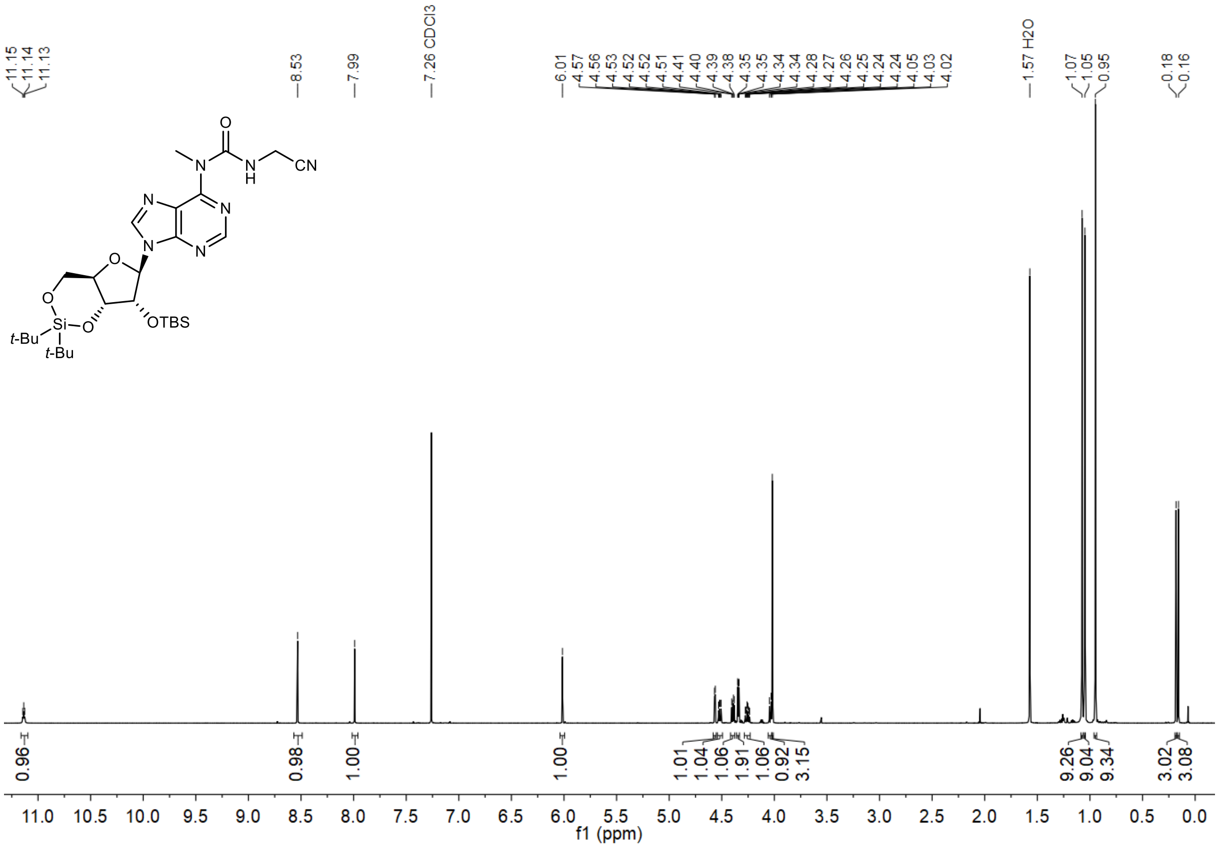

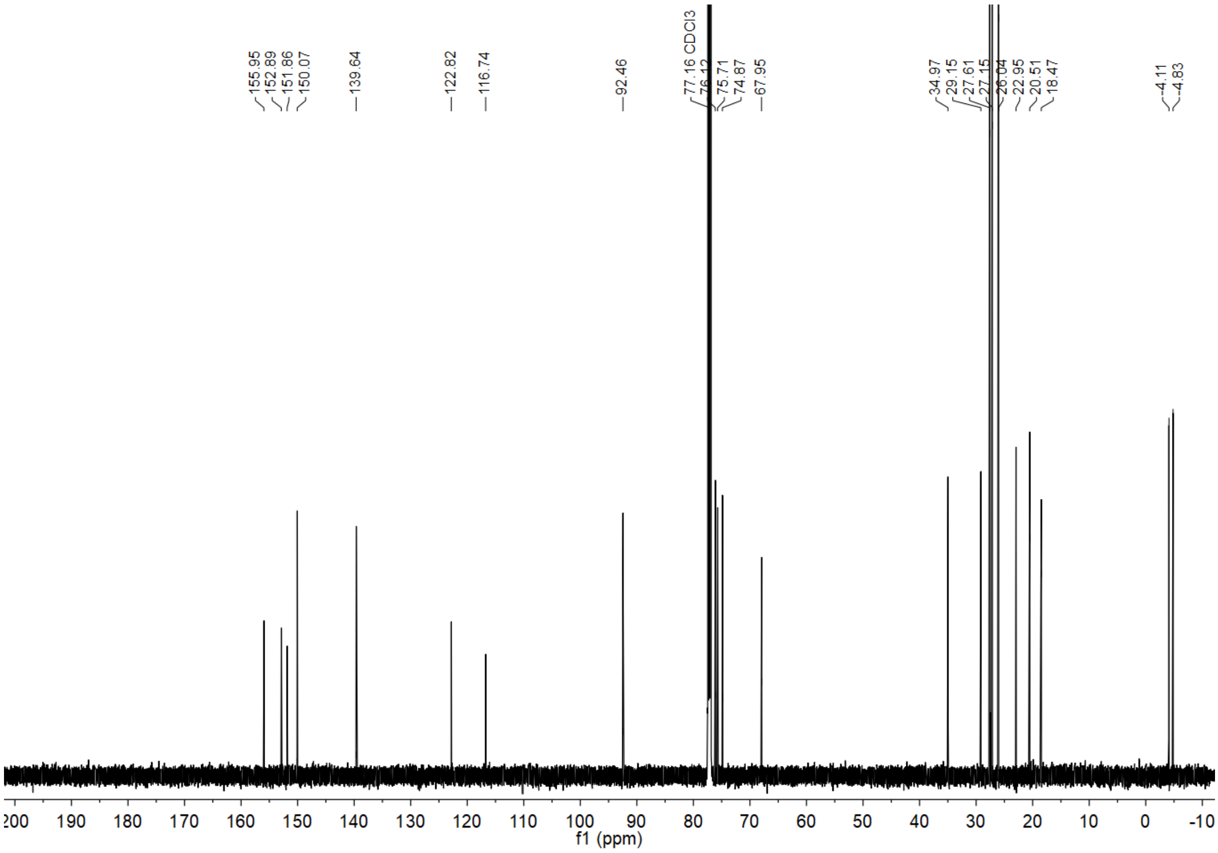

^{1}H and ^{13}C{^{1}H} NMR spectra of compound 12a

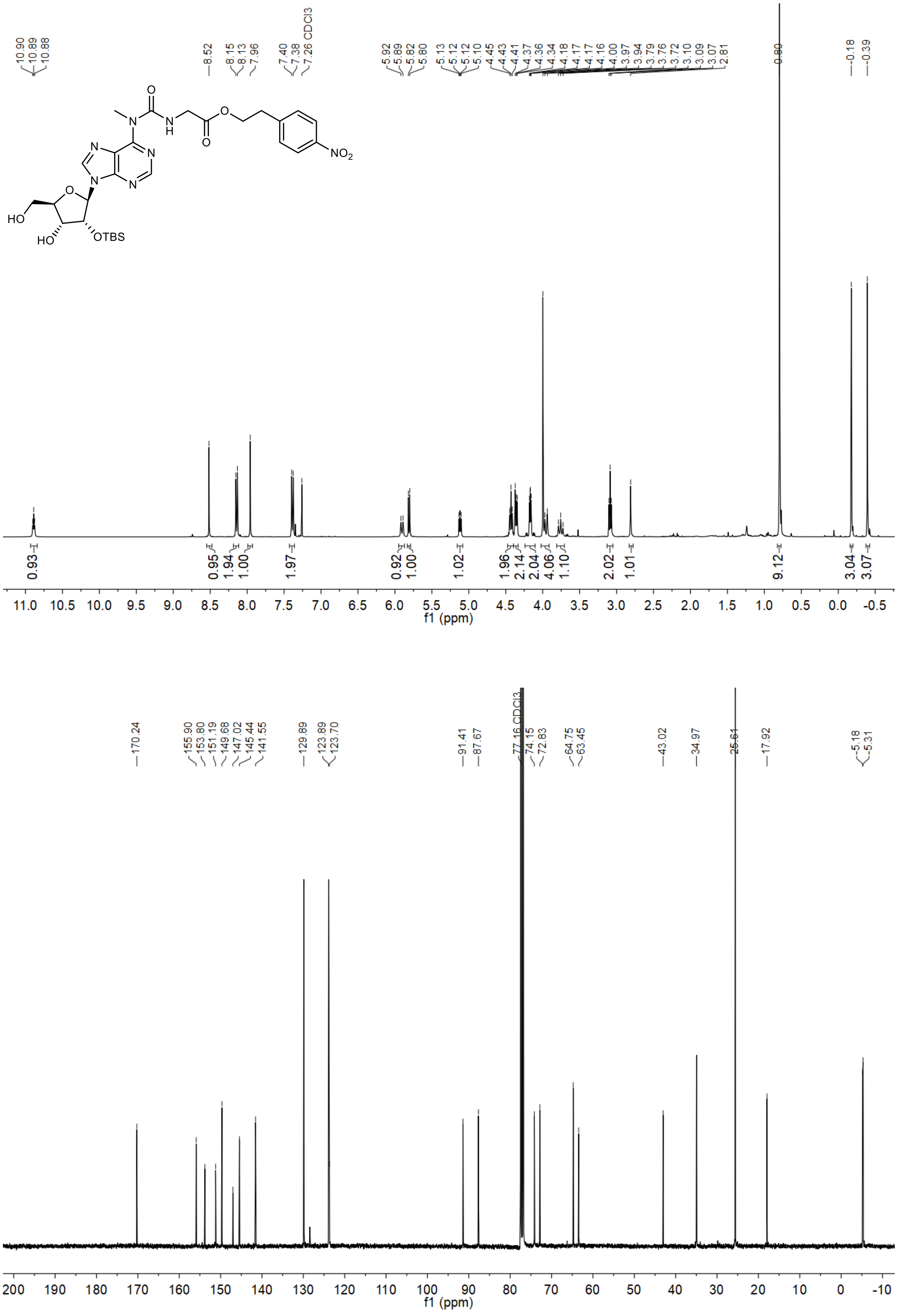

^{1}H and ^{13}C{^{1}H} NMR spectra of compound 12b

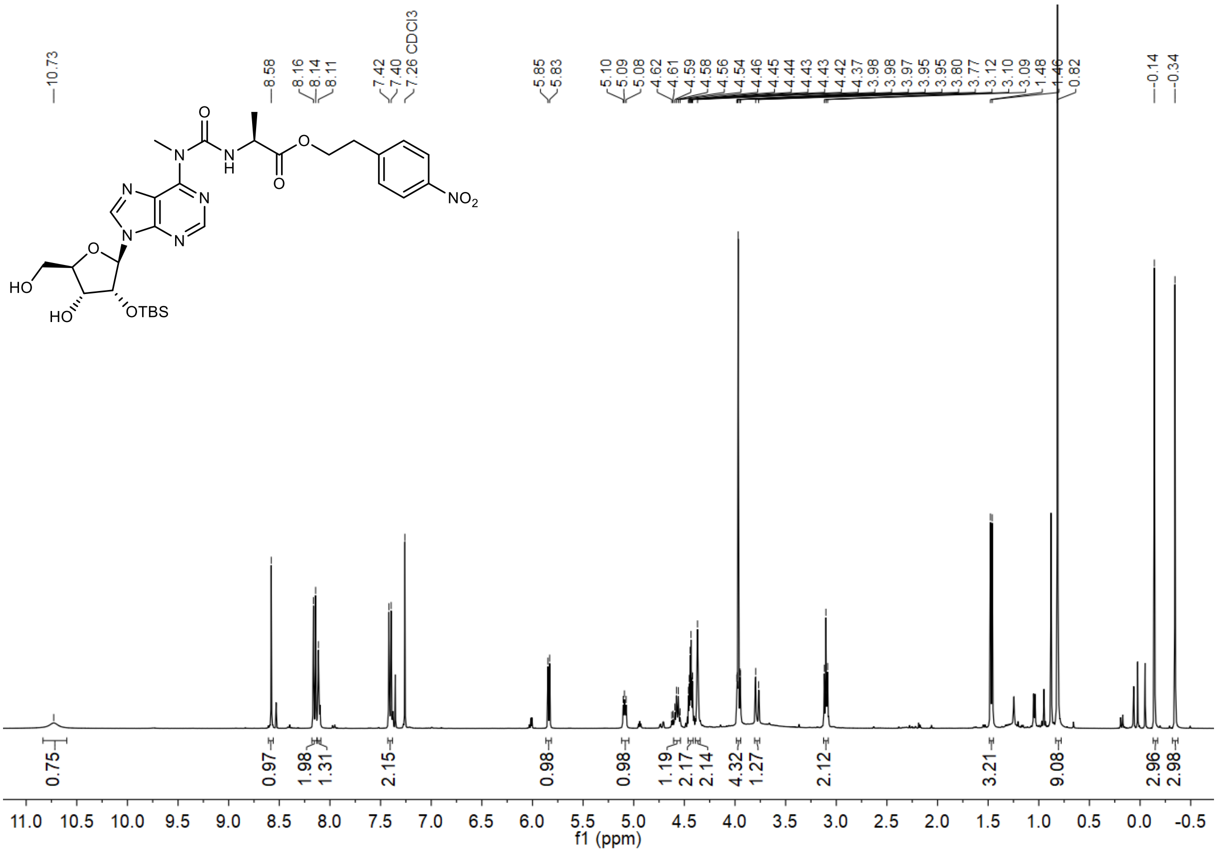

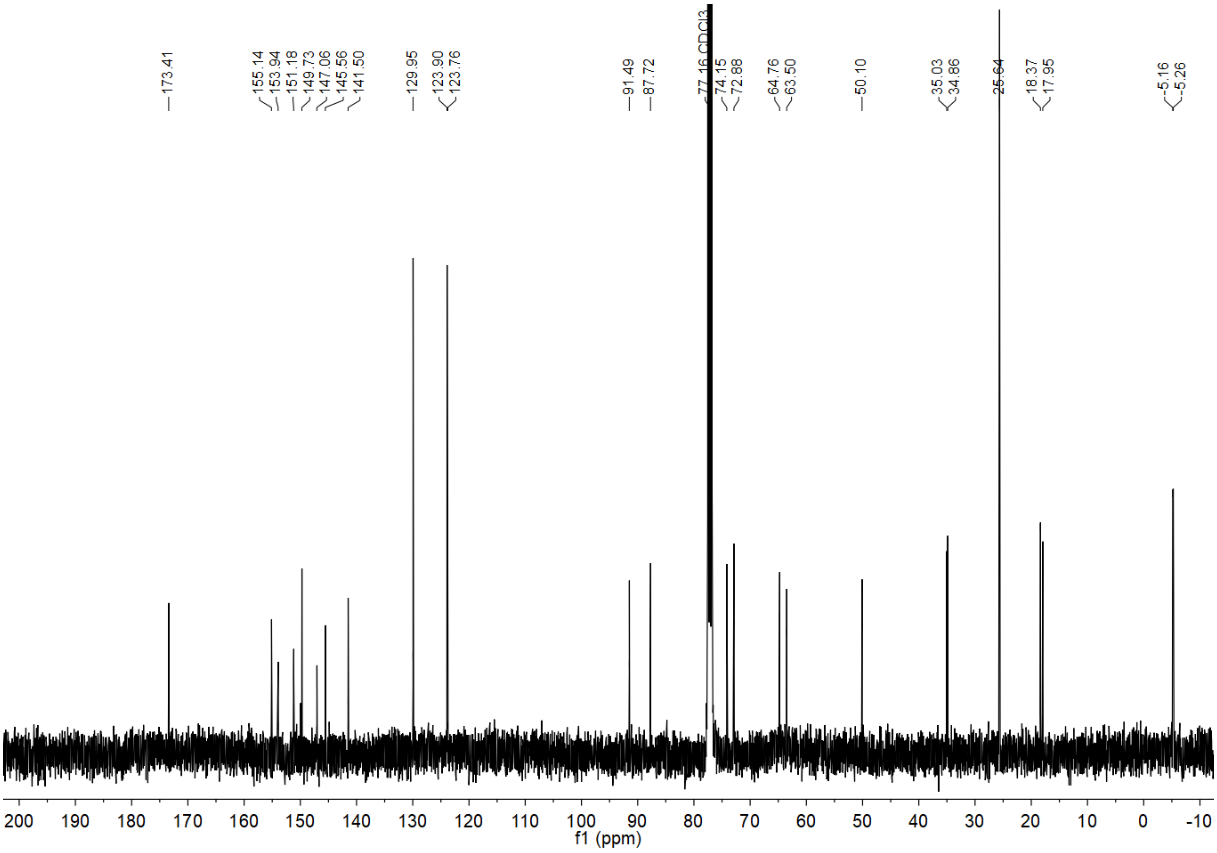

^{1}H and ^{13}C{^{1}H} NMR spectra of compound 12c

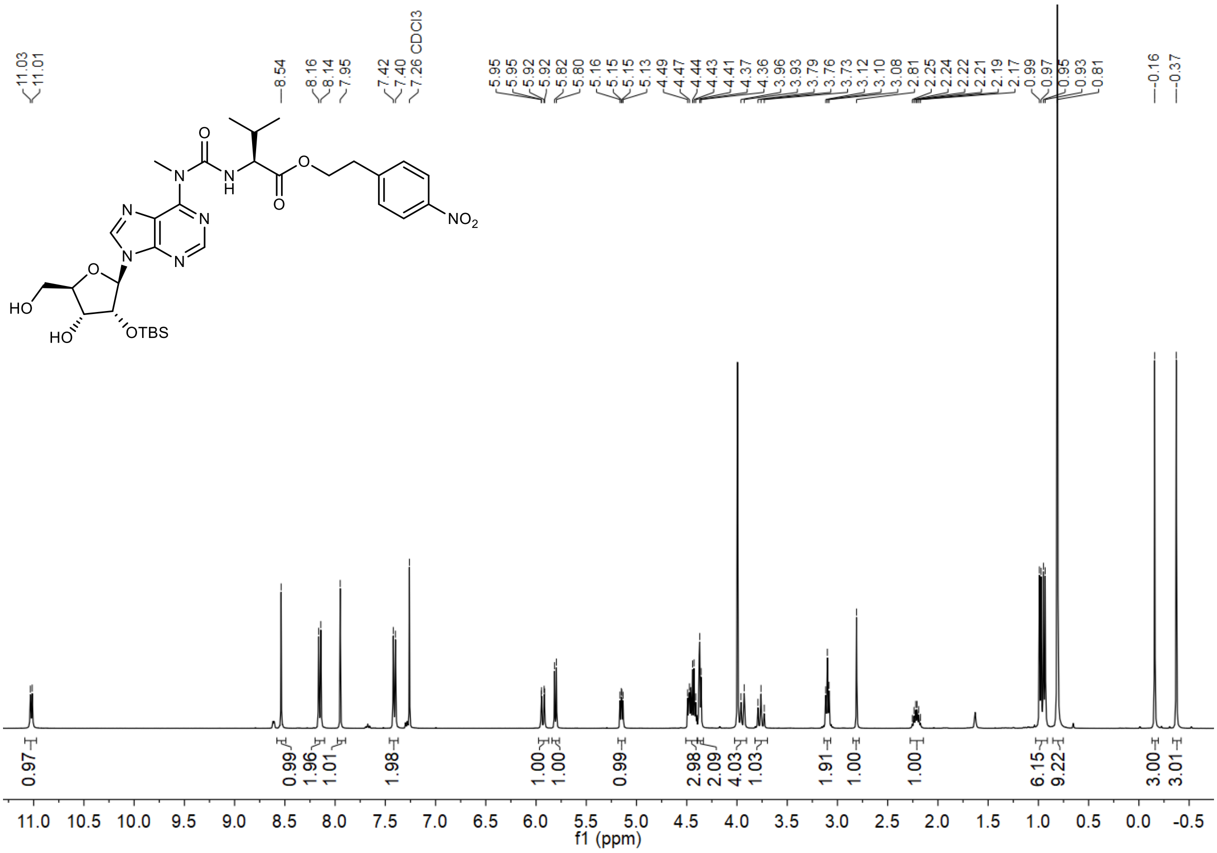

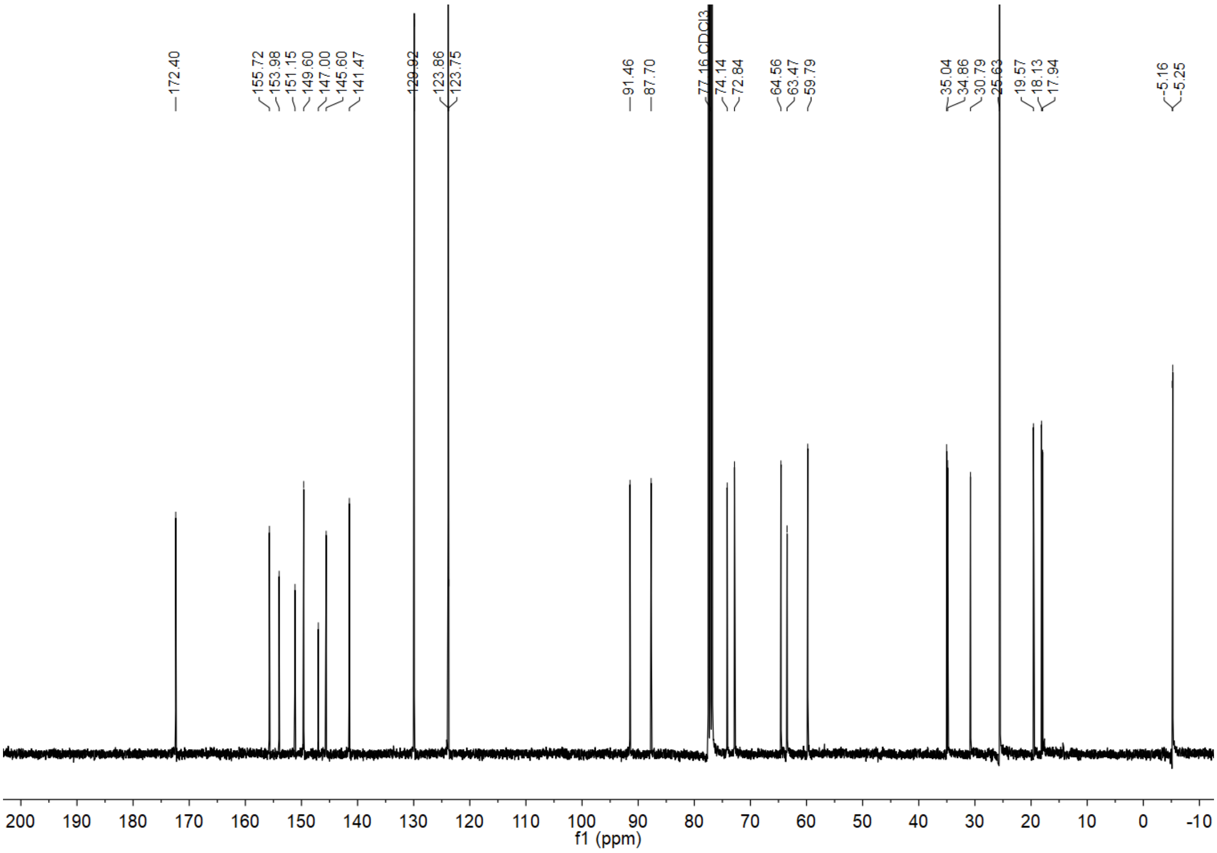

^{1}H and $^{13}C\{^{1}H\}$ NMR spectra of compound 12d

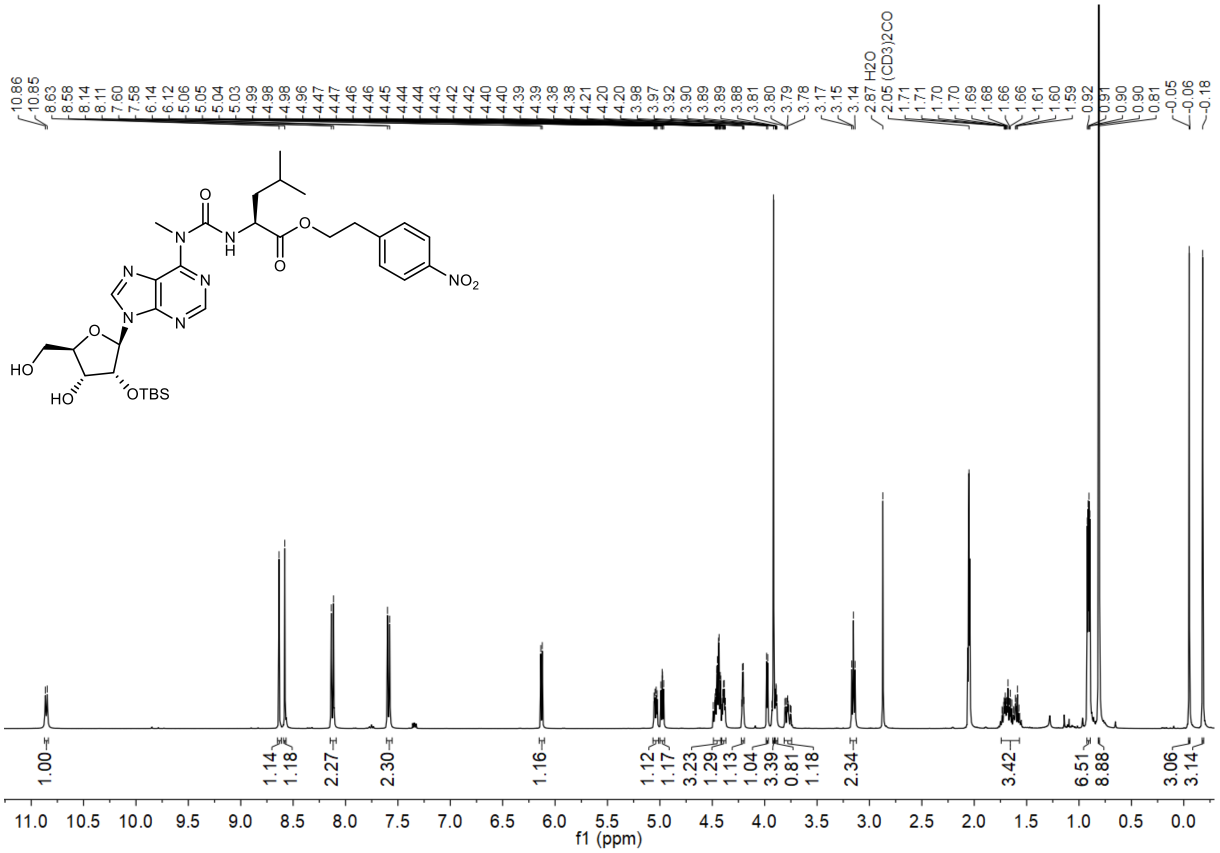

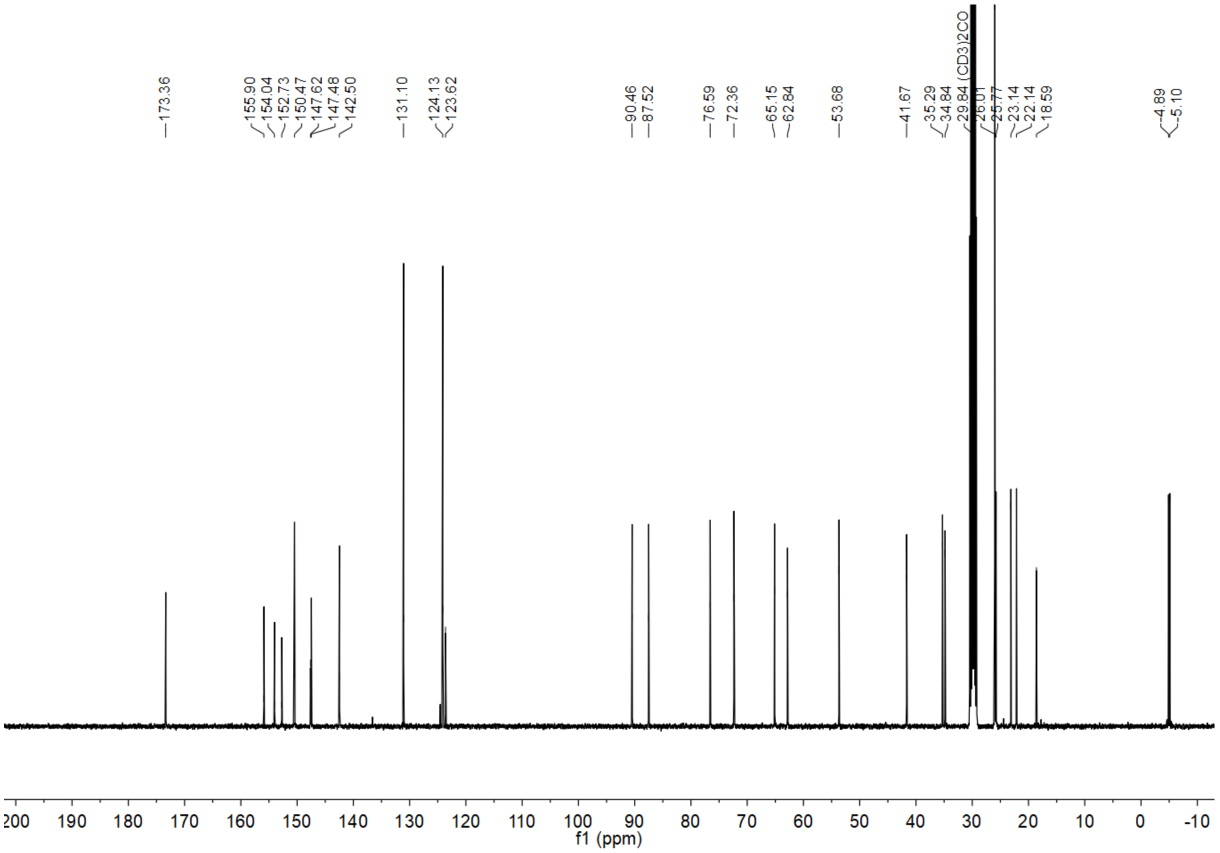

^{1}H and ^{13}C{^{1}H} NMR spectra of compound 12e

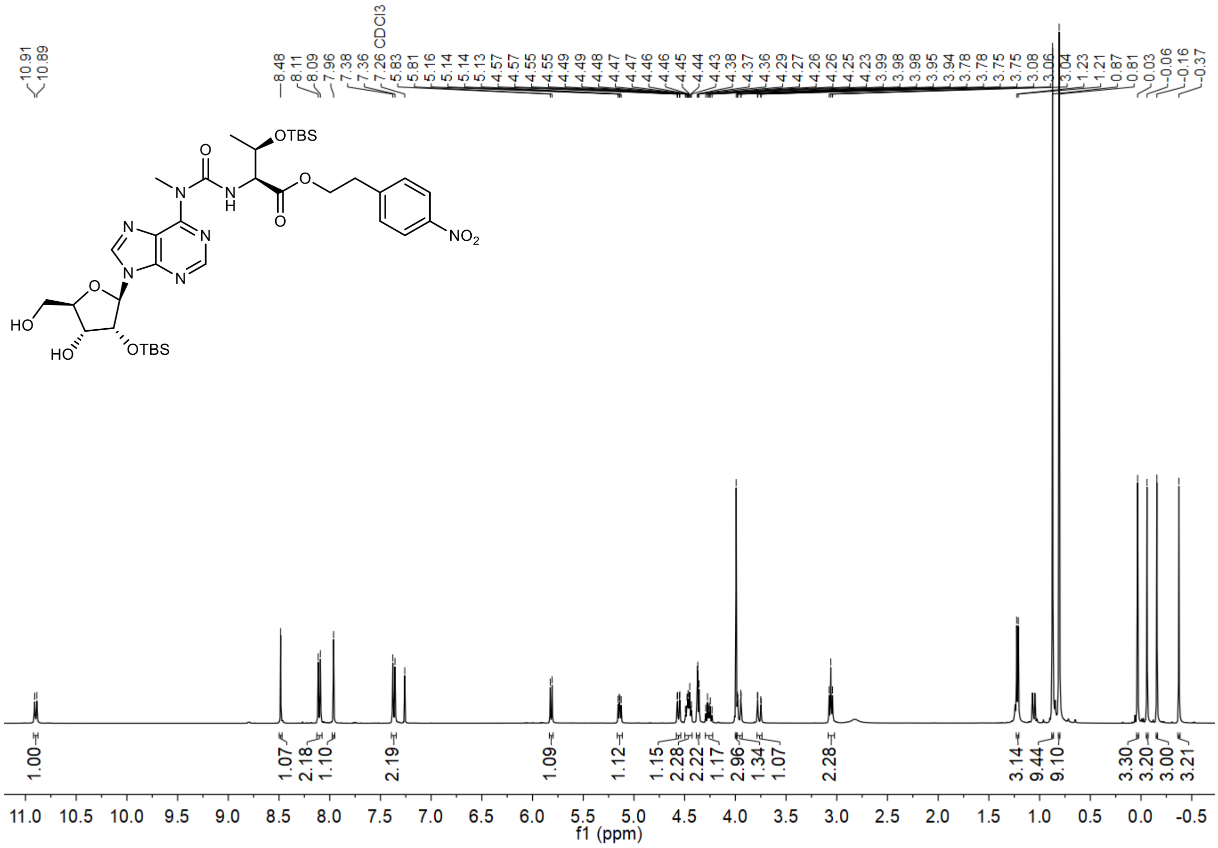

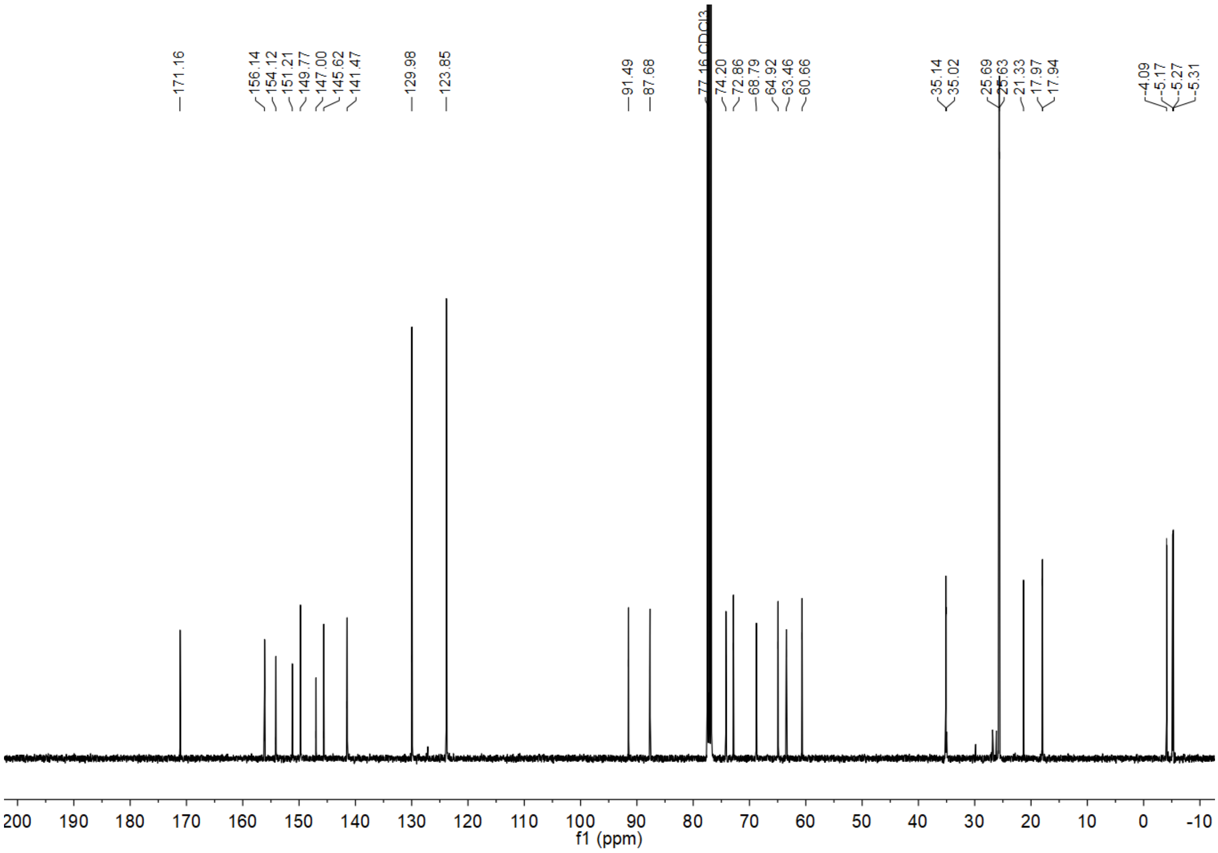

^{1}H and $^{13}C\{^{1}H\}$ NMR spectra of compound 12f

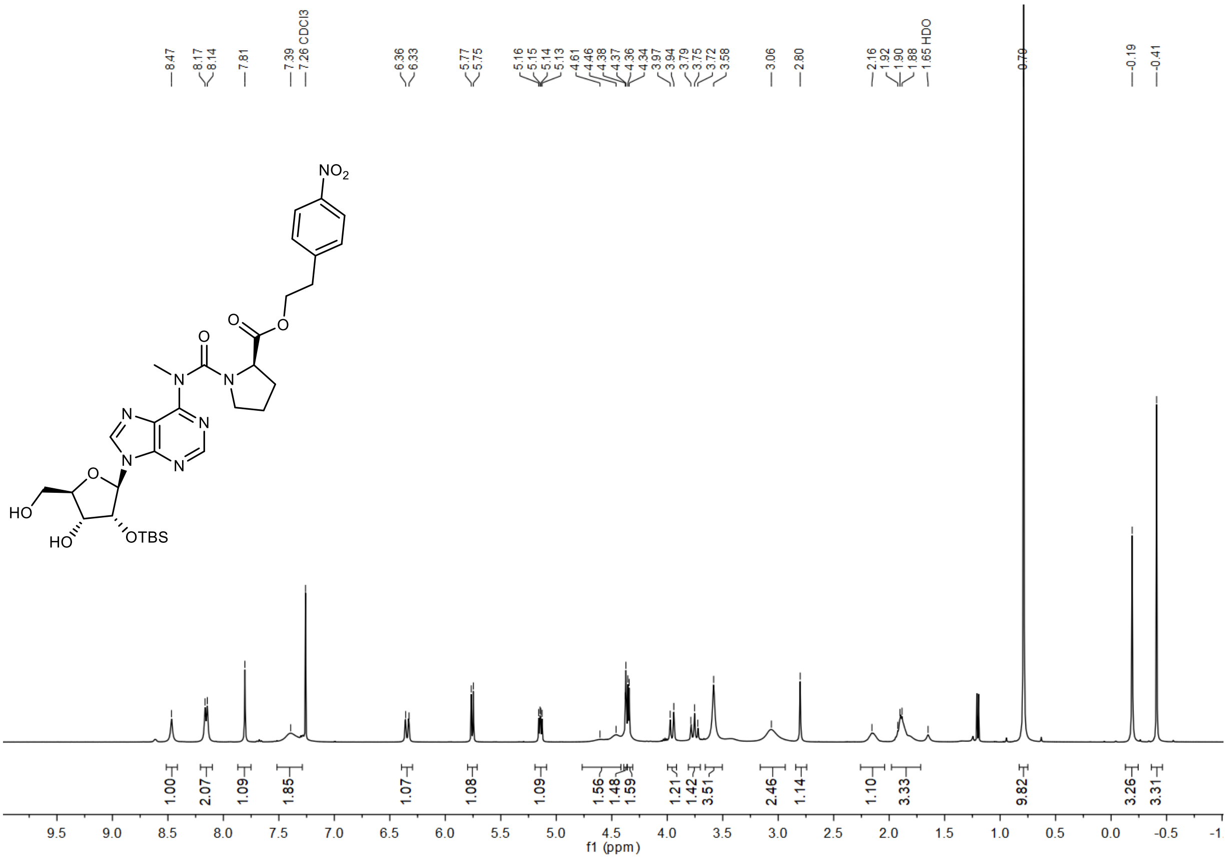

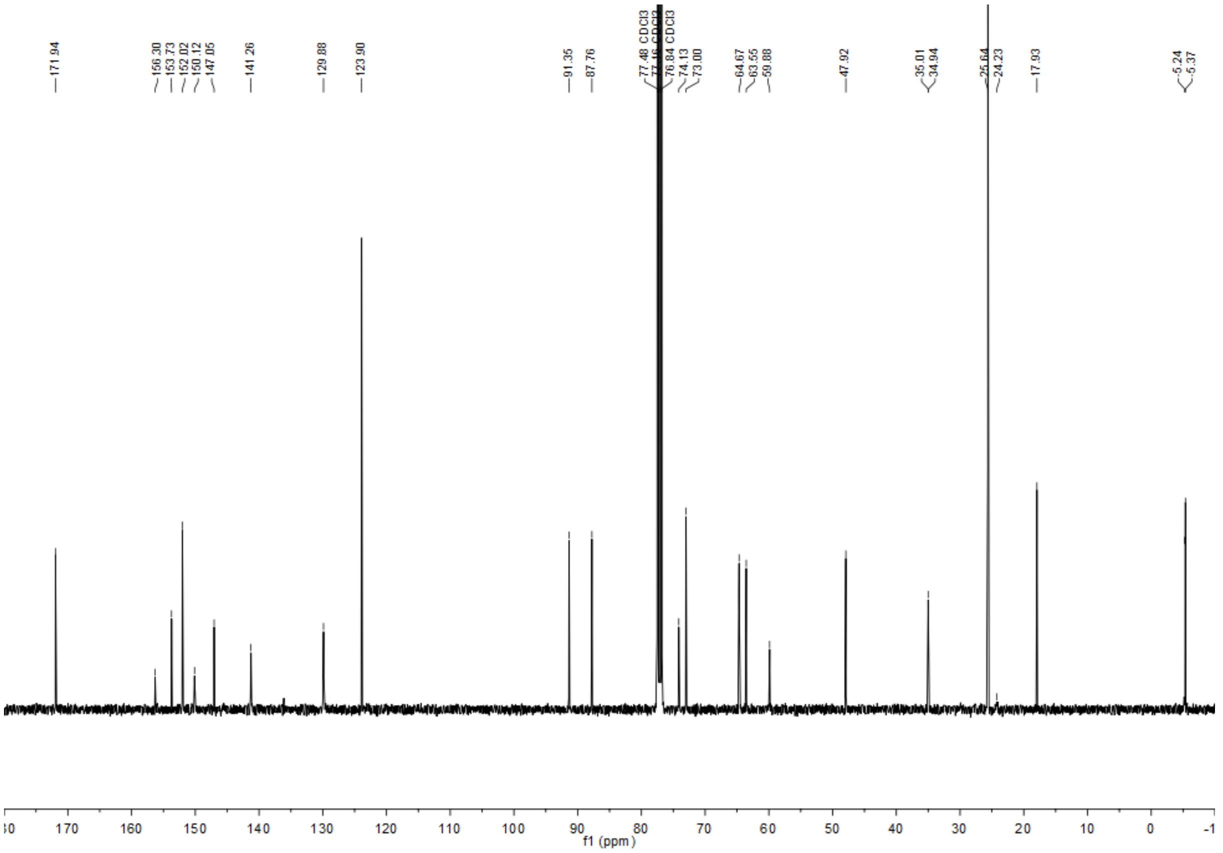

^{1}H and $^{13}C\{^{1}H\}$ NMR spectra of compound 12g

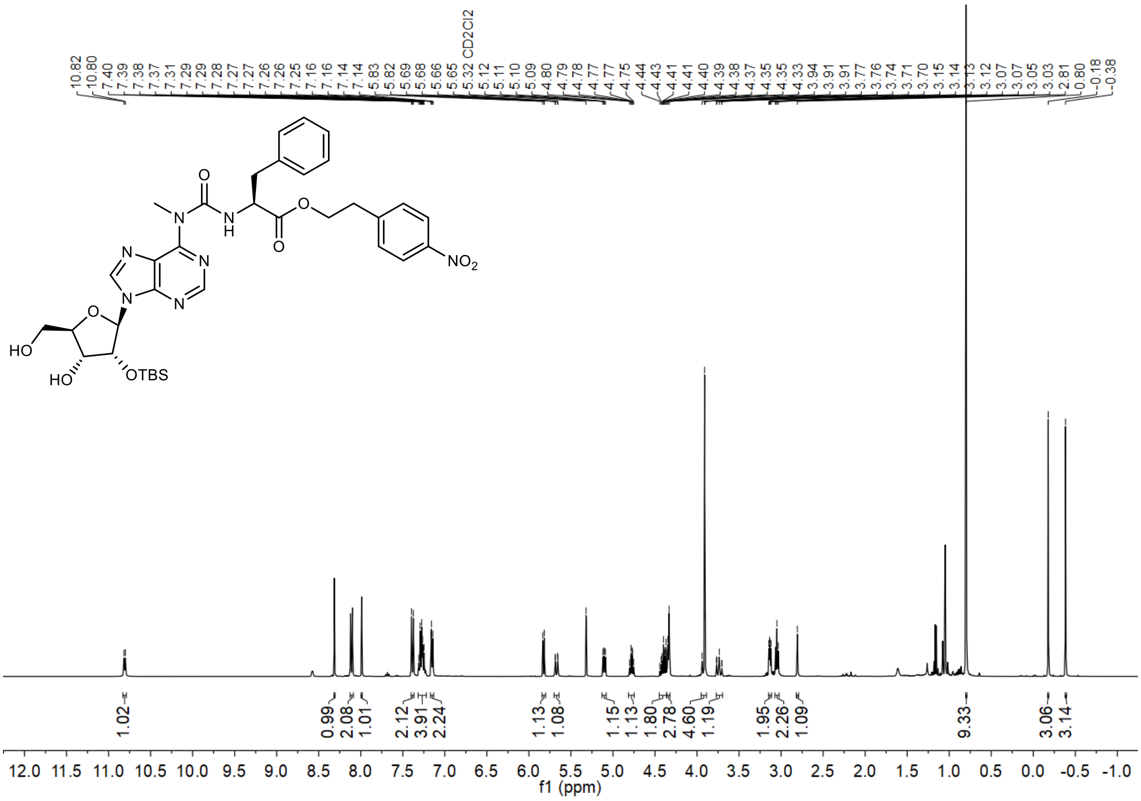

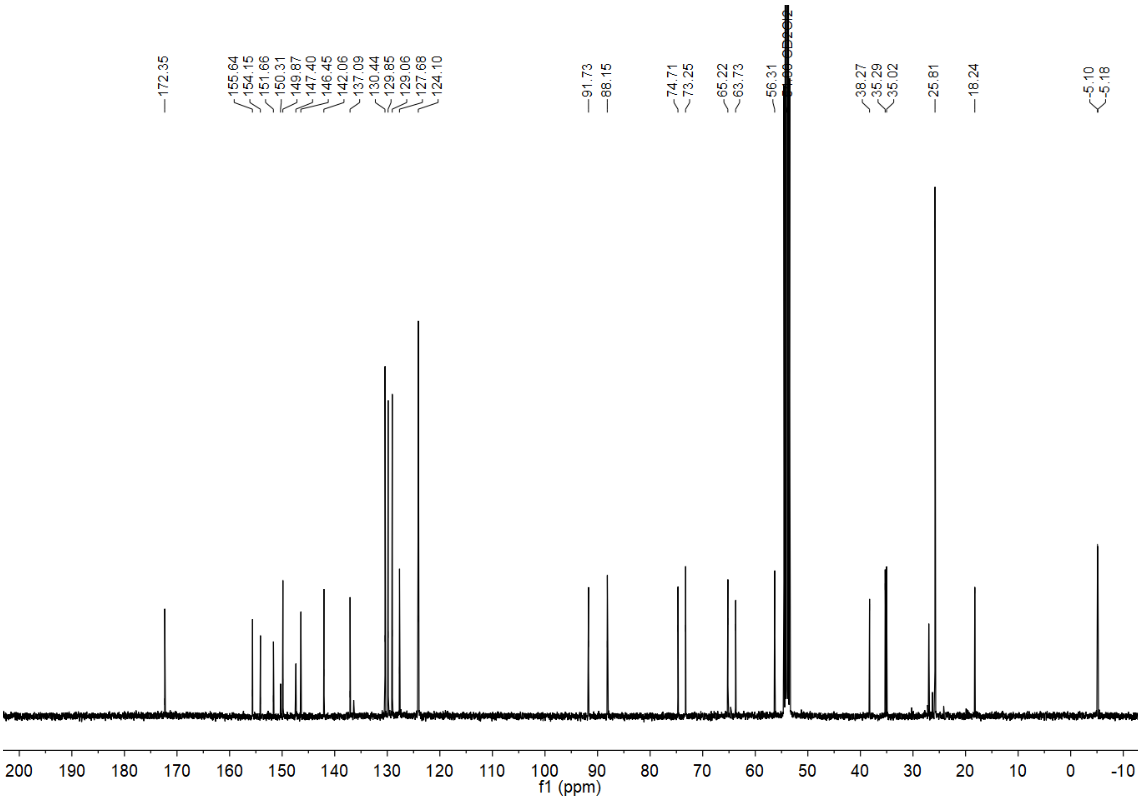

^{1}H and ^{13}C{^{1}H} NMR spectra of compound 12h

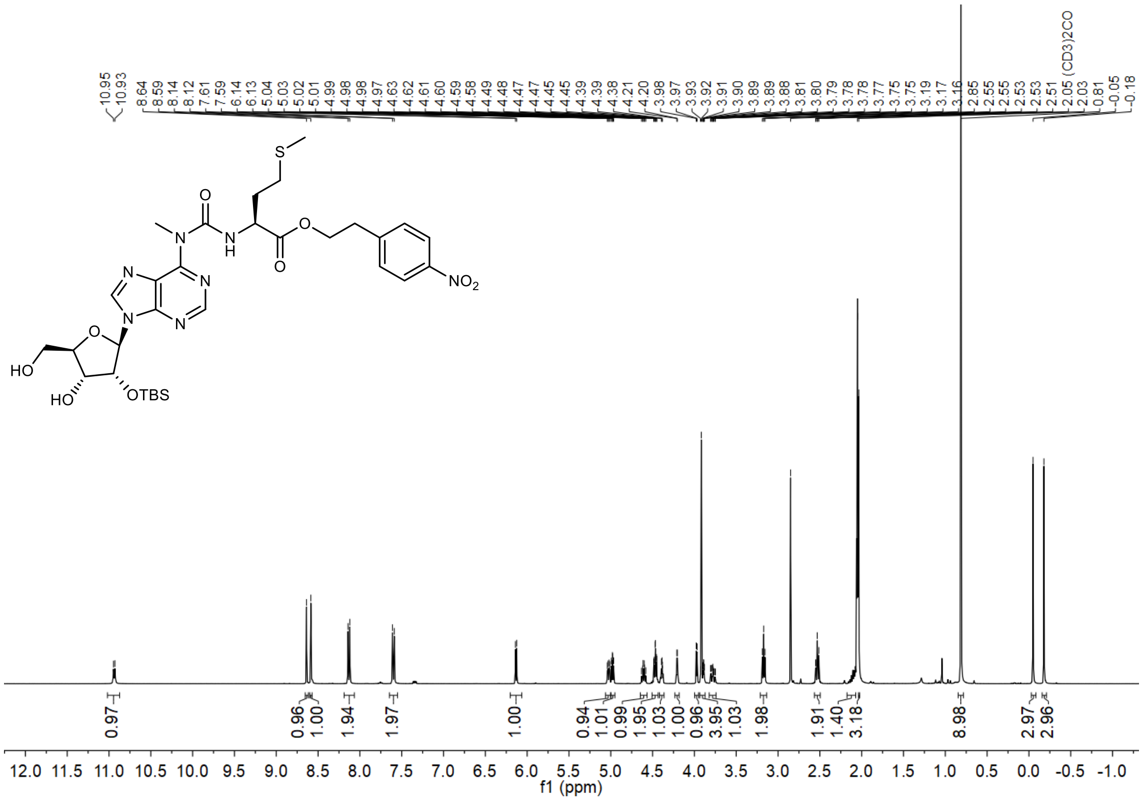

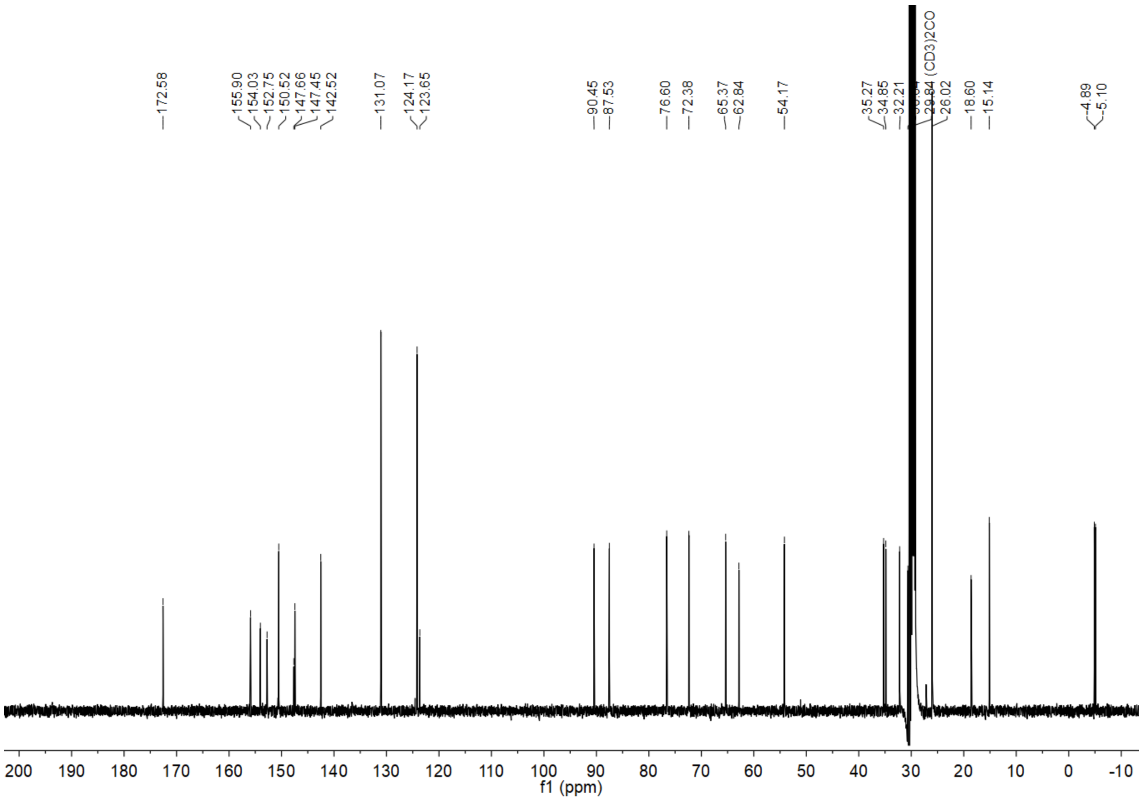

^{1}H and ^{13}C{^{1}H} NMR spectra of compound 12i

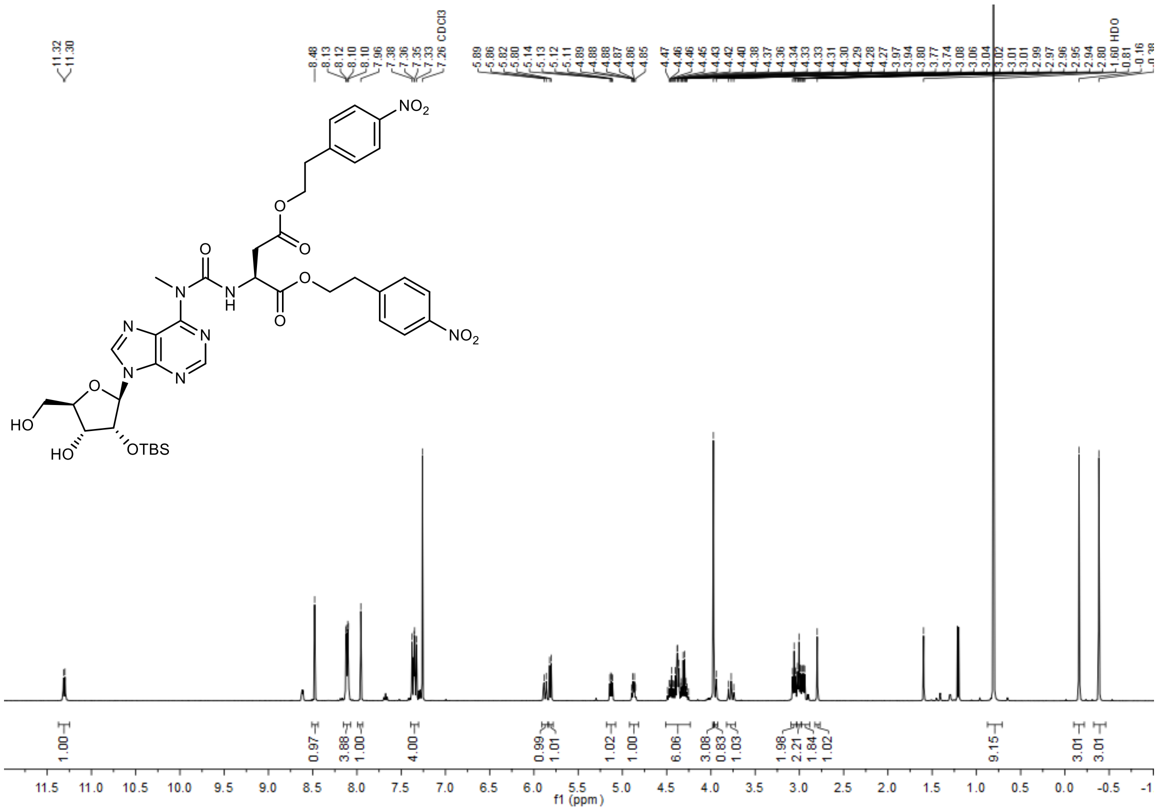

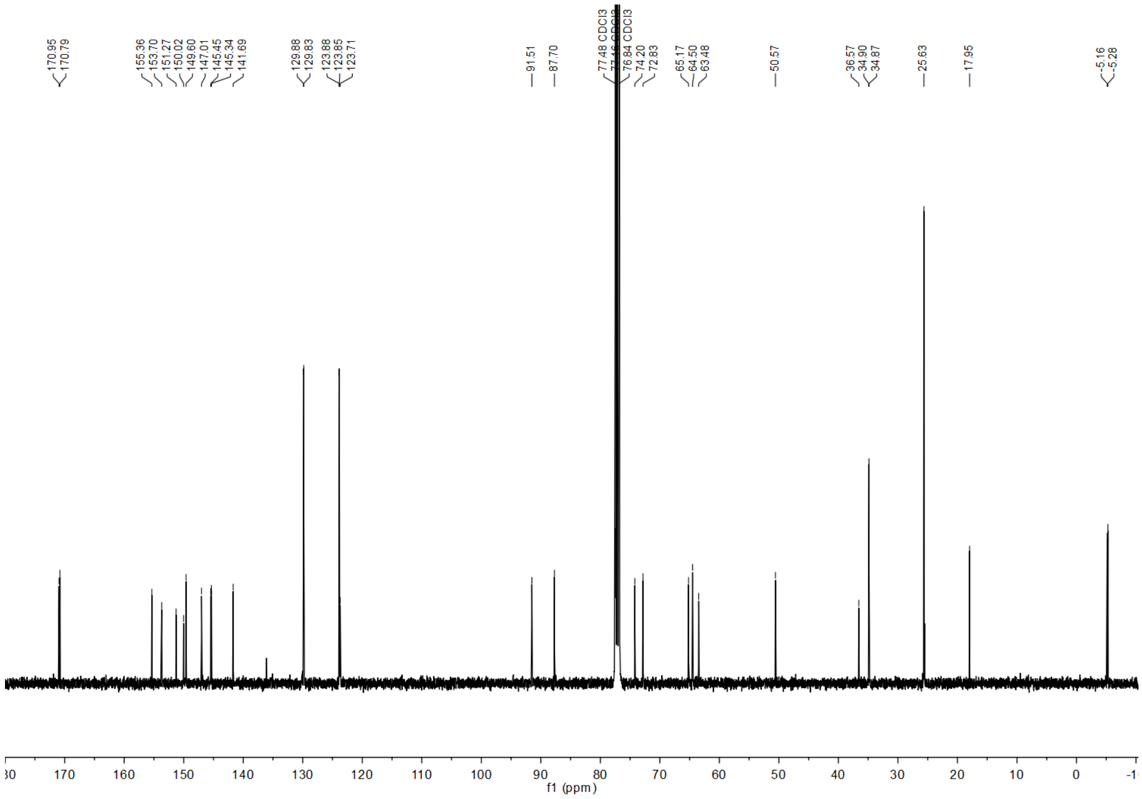

^{1}H and $^{13}C\{^{1}H\}$ NMR spectra of compound 12j

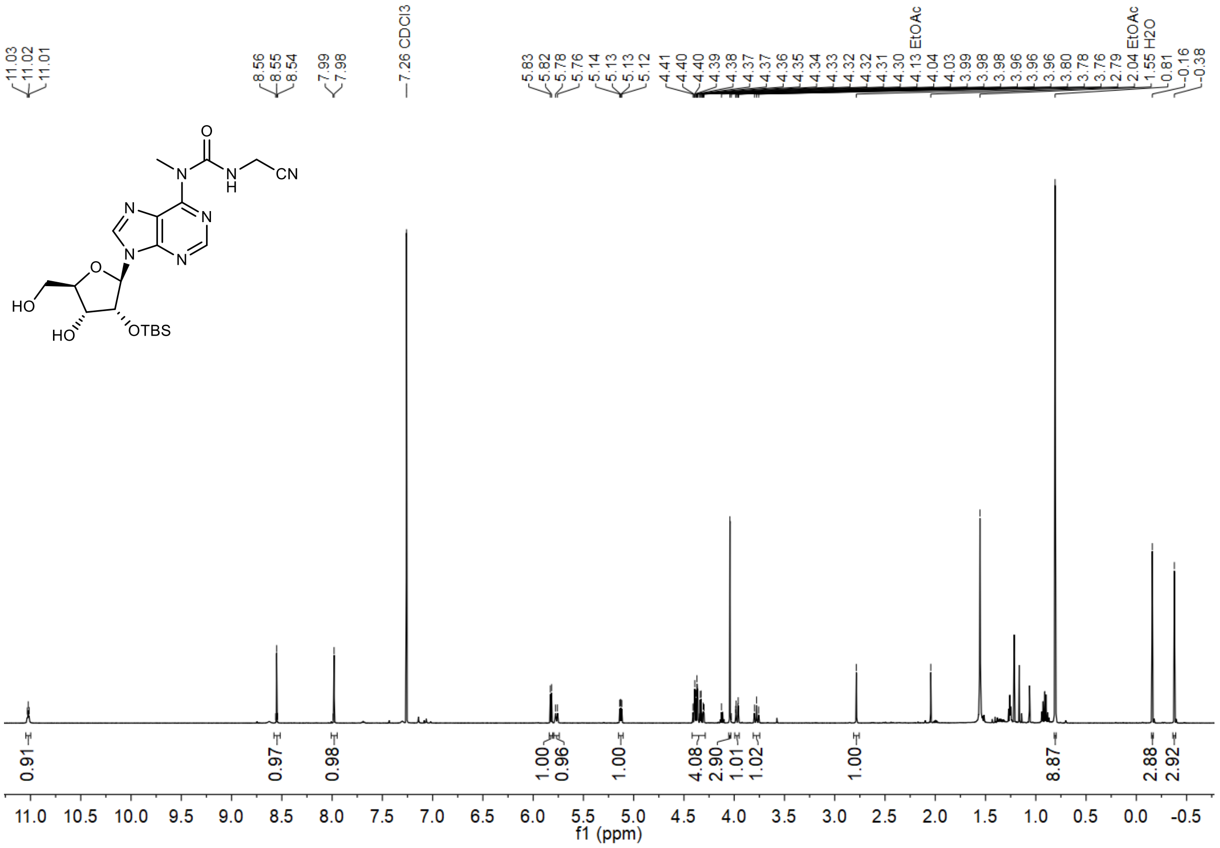

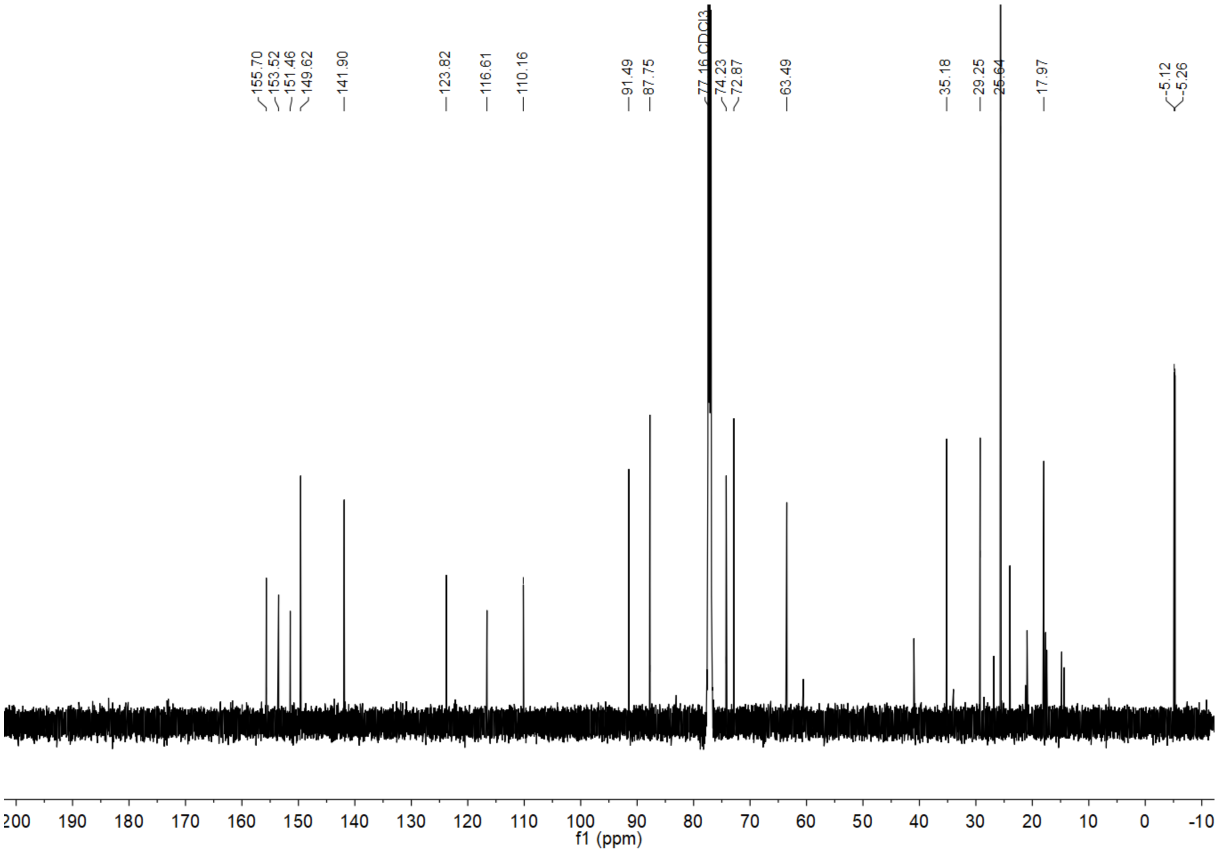

^{1}H and ^{13}C{^{1}H} NMR spectra of compound 13a

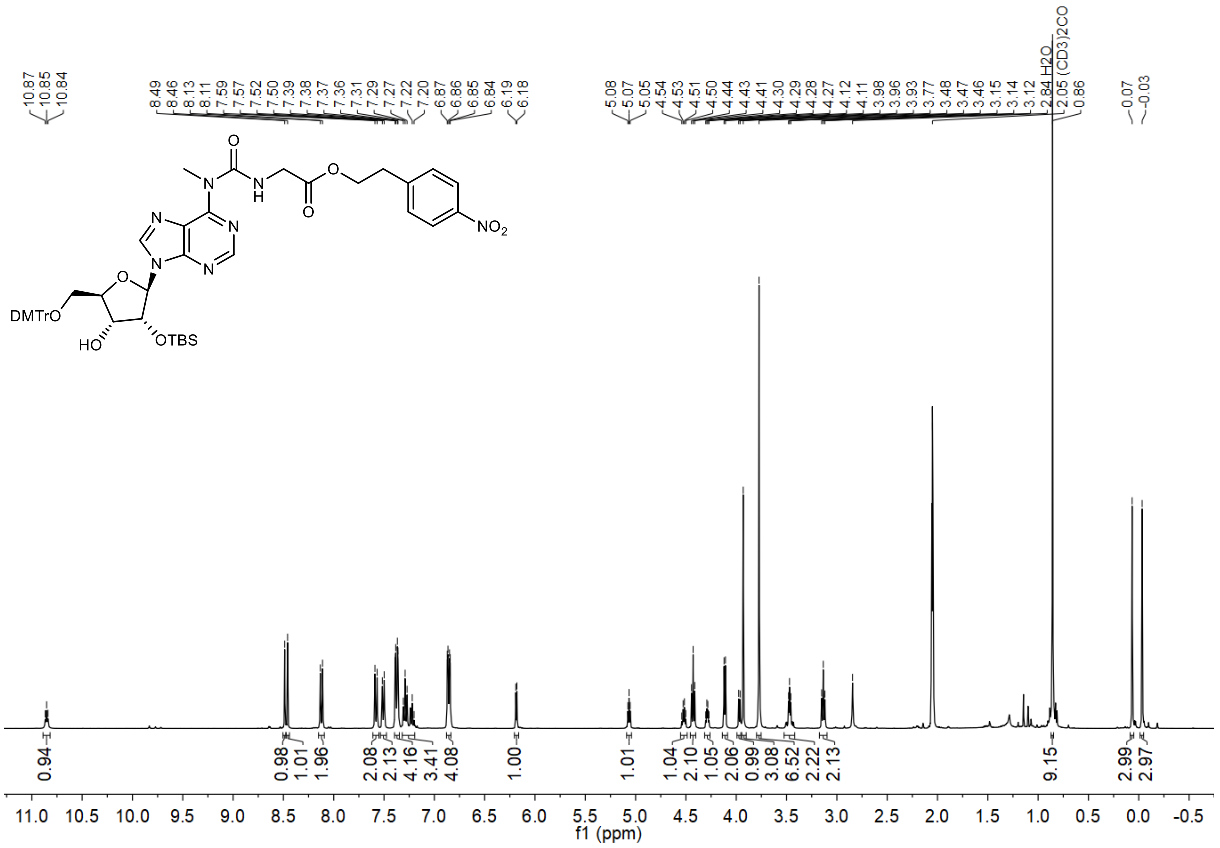

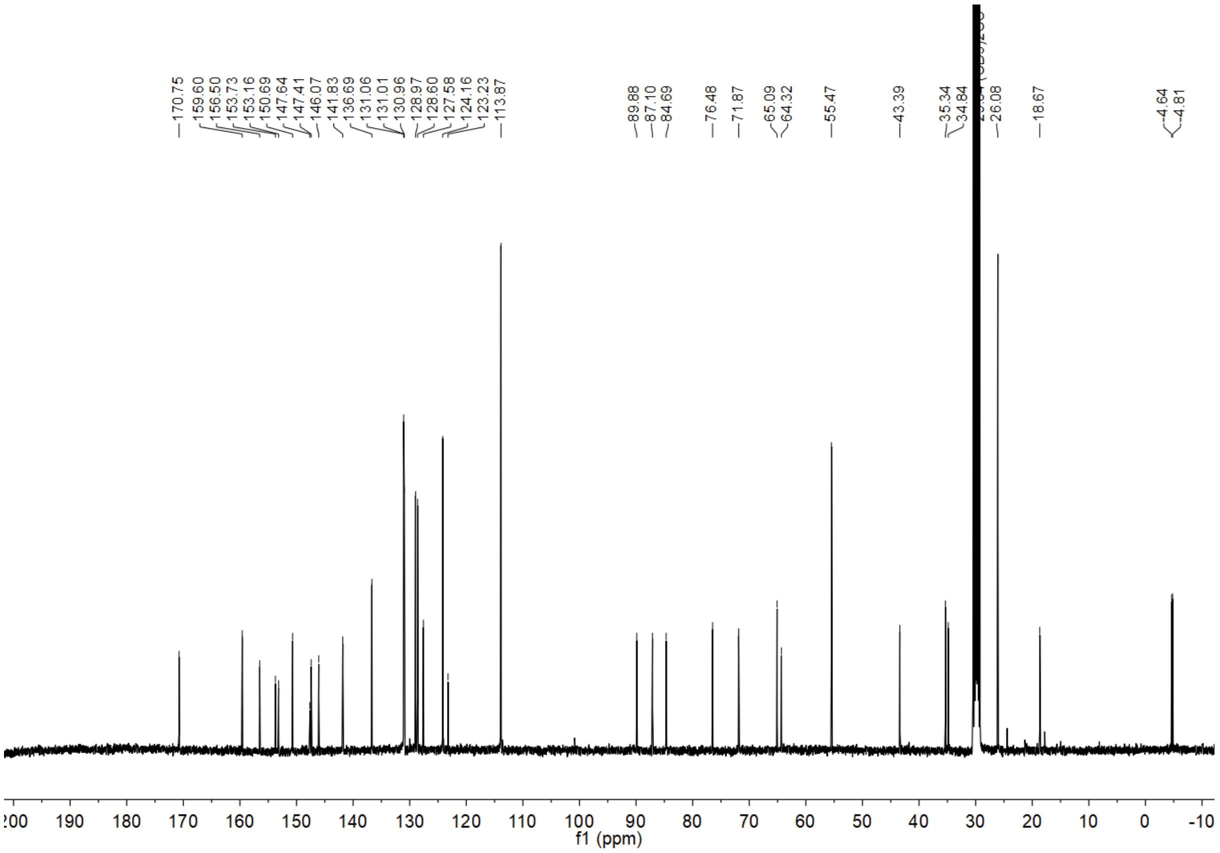

^{1}H and ^{13}C{^{1}H} NMR spectra of compound 13b

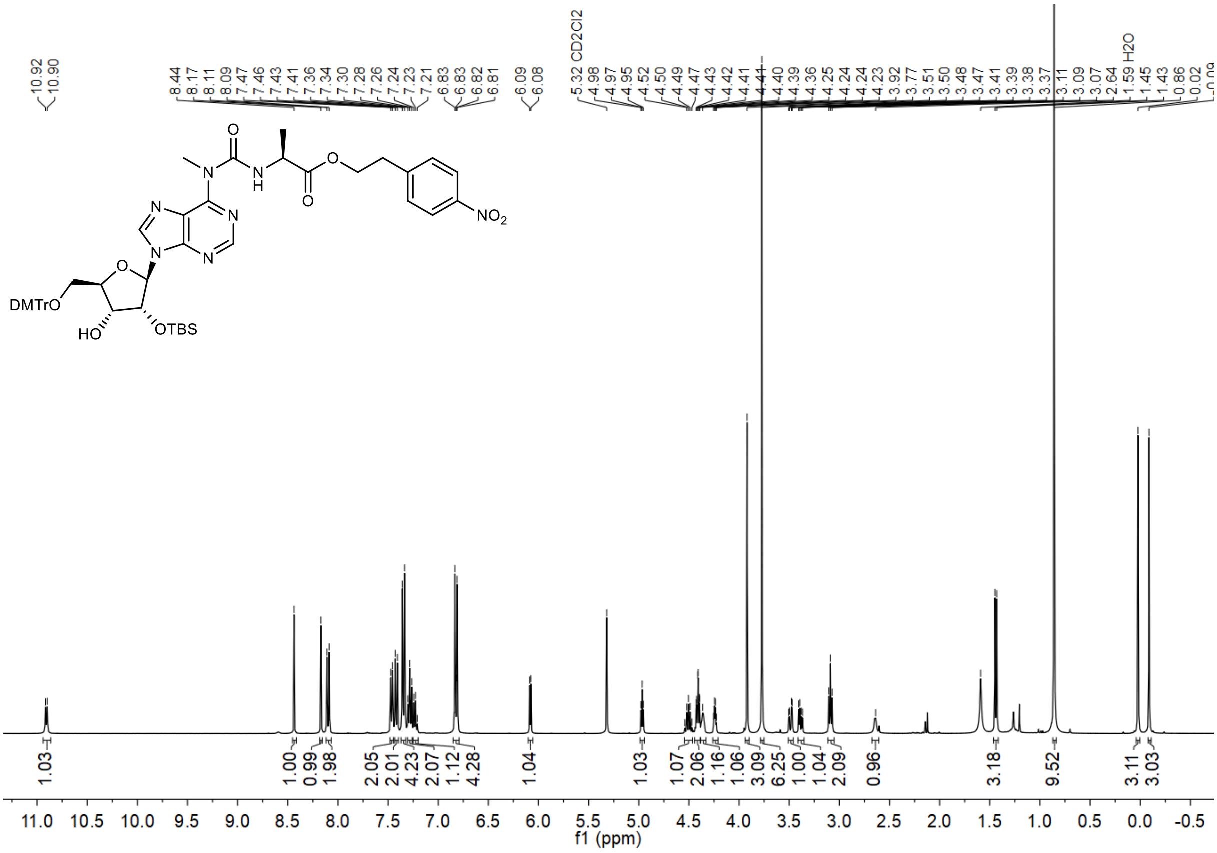

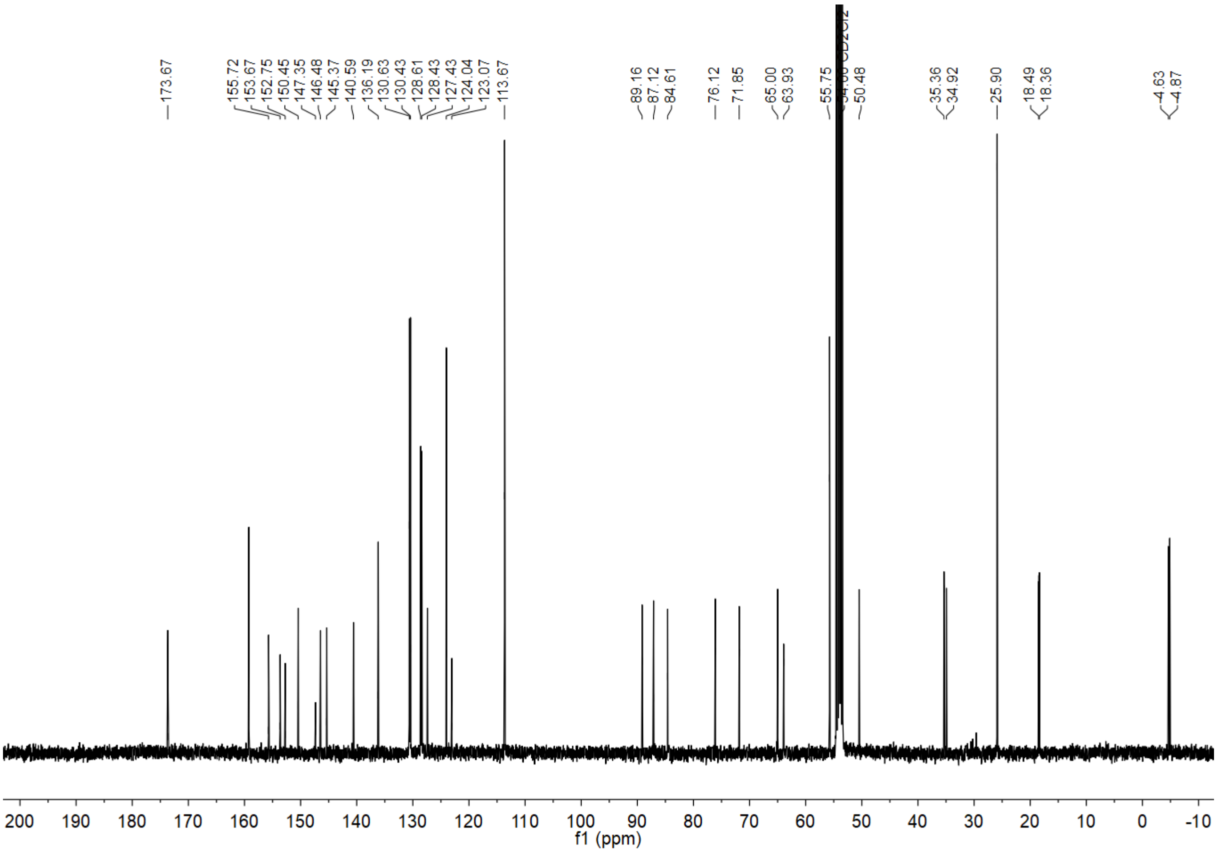

^{1}H and ^{13}C{^{1}H} NMR spectra of compound 13c

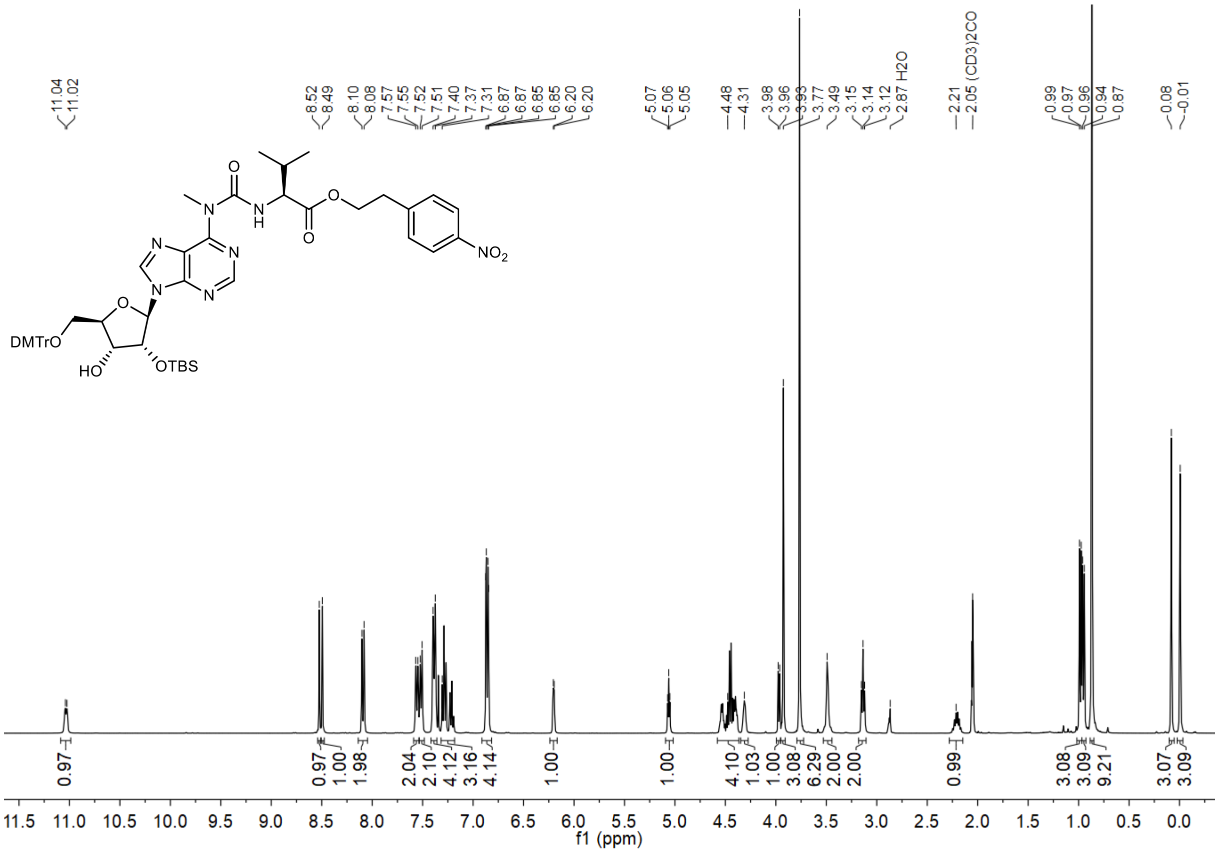

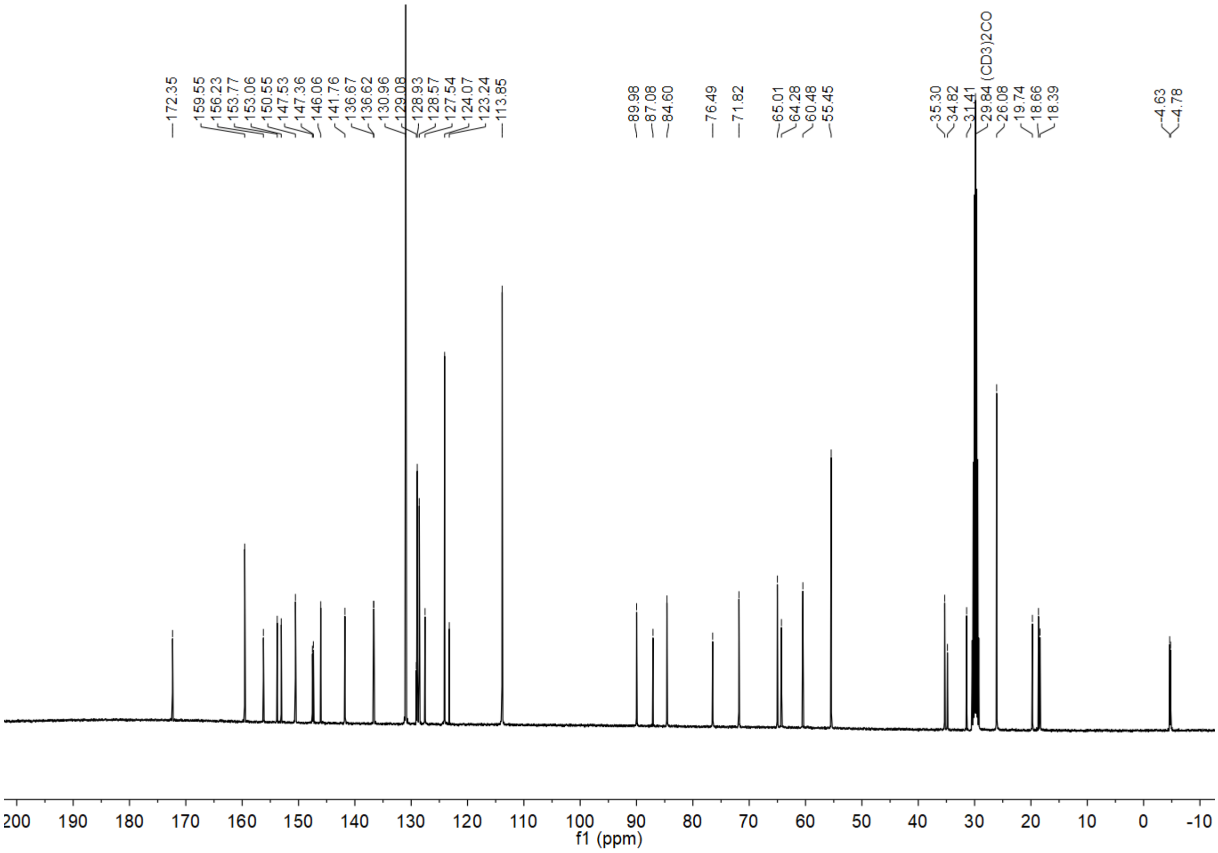

^{1}H and ^{13}C{^{1}H} NMR spectra of compound 13d

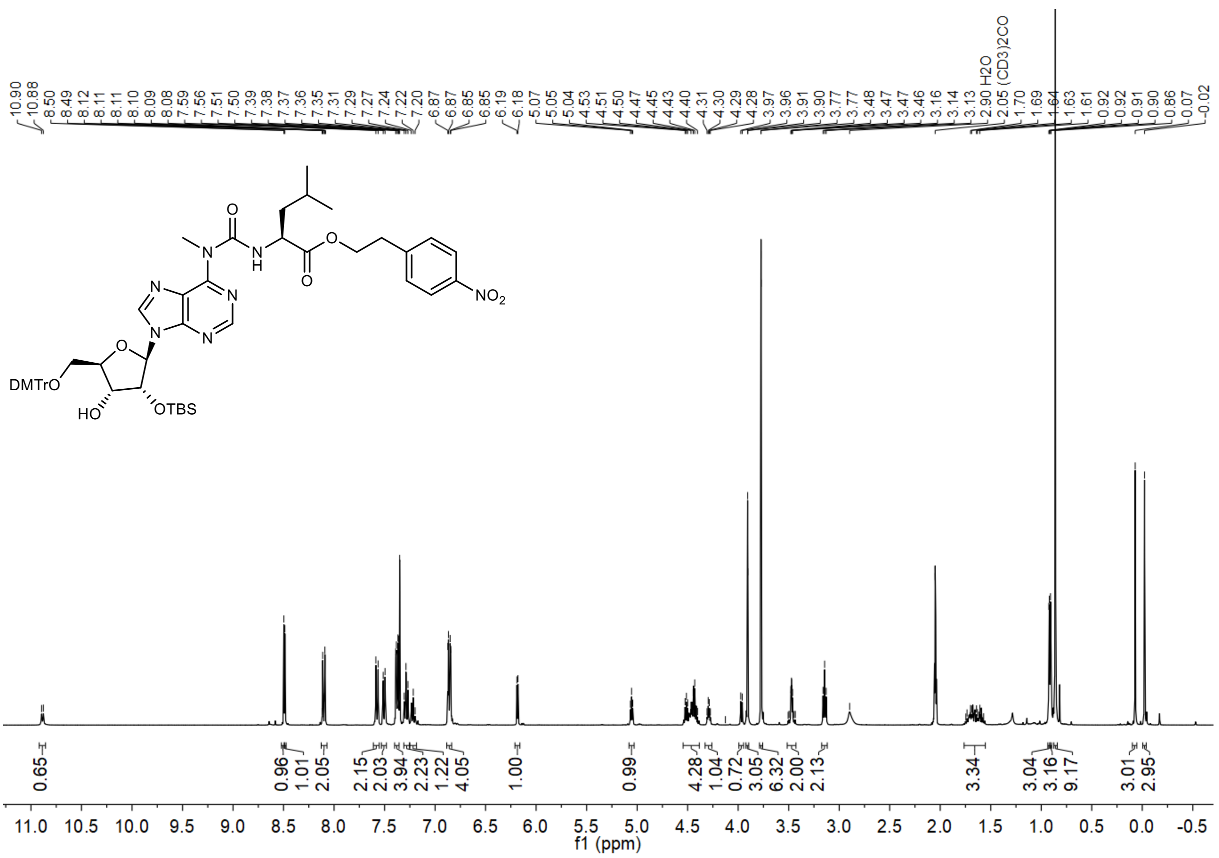

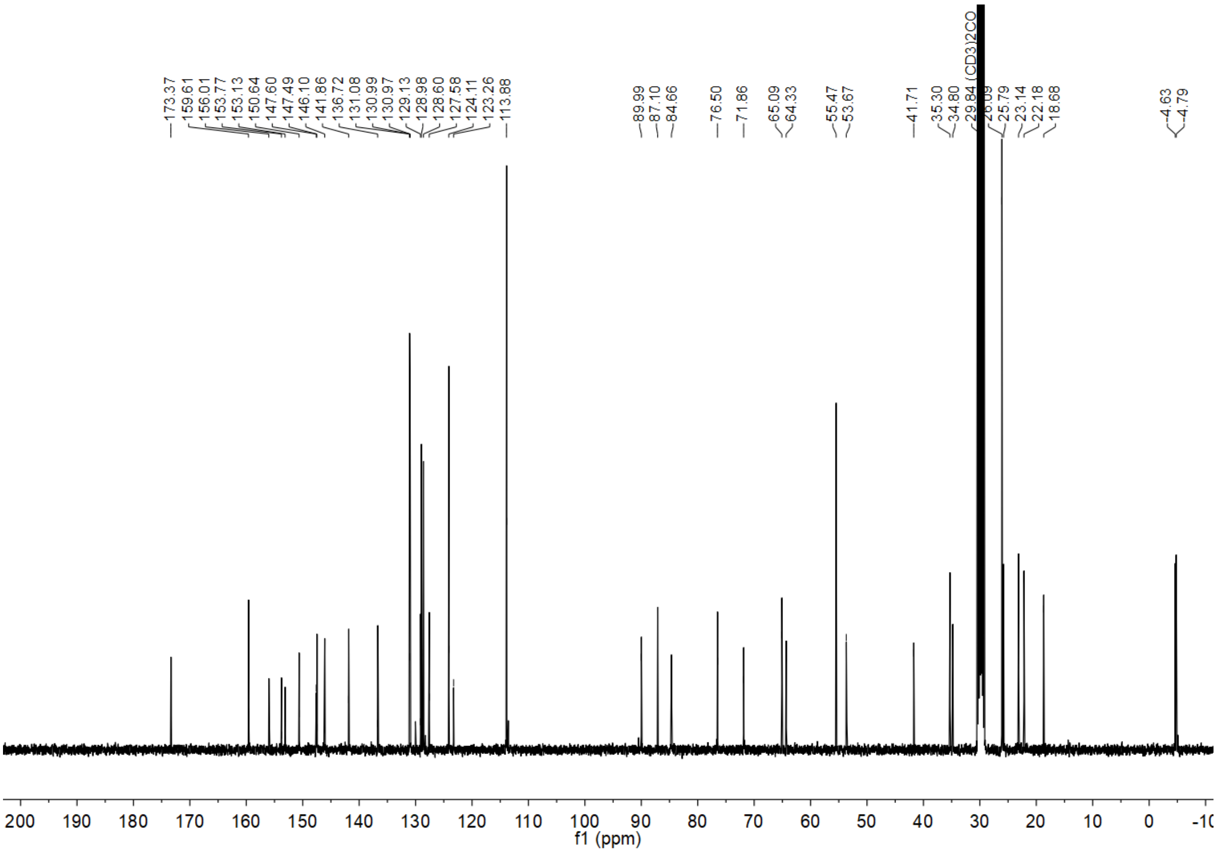

^{1}H and ^{13}C{^{1}H} NMR spectra of compound 13e

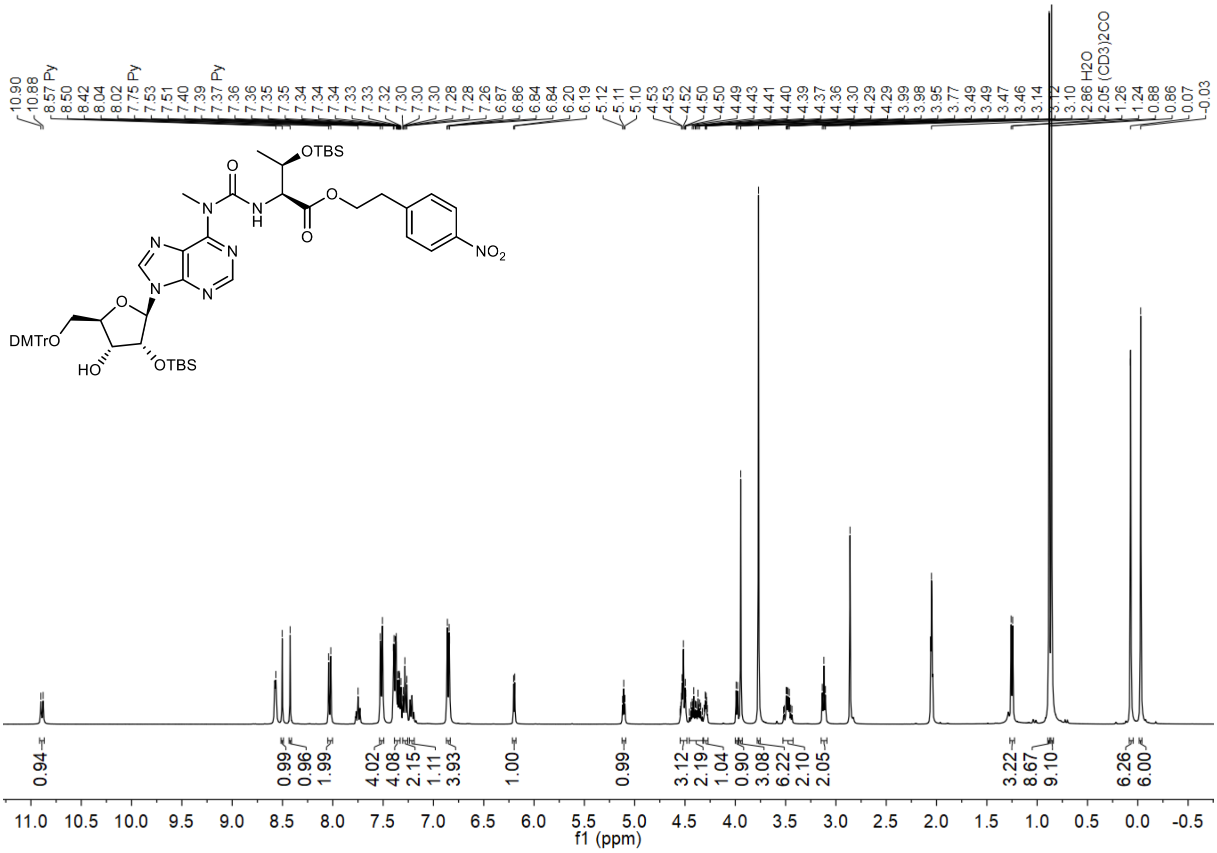

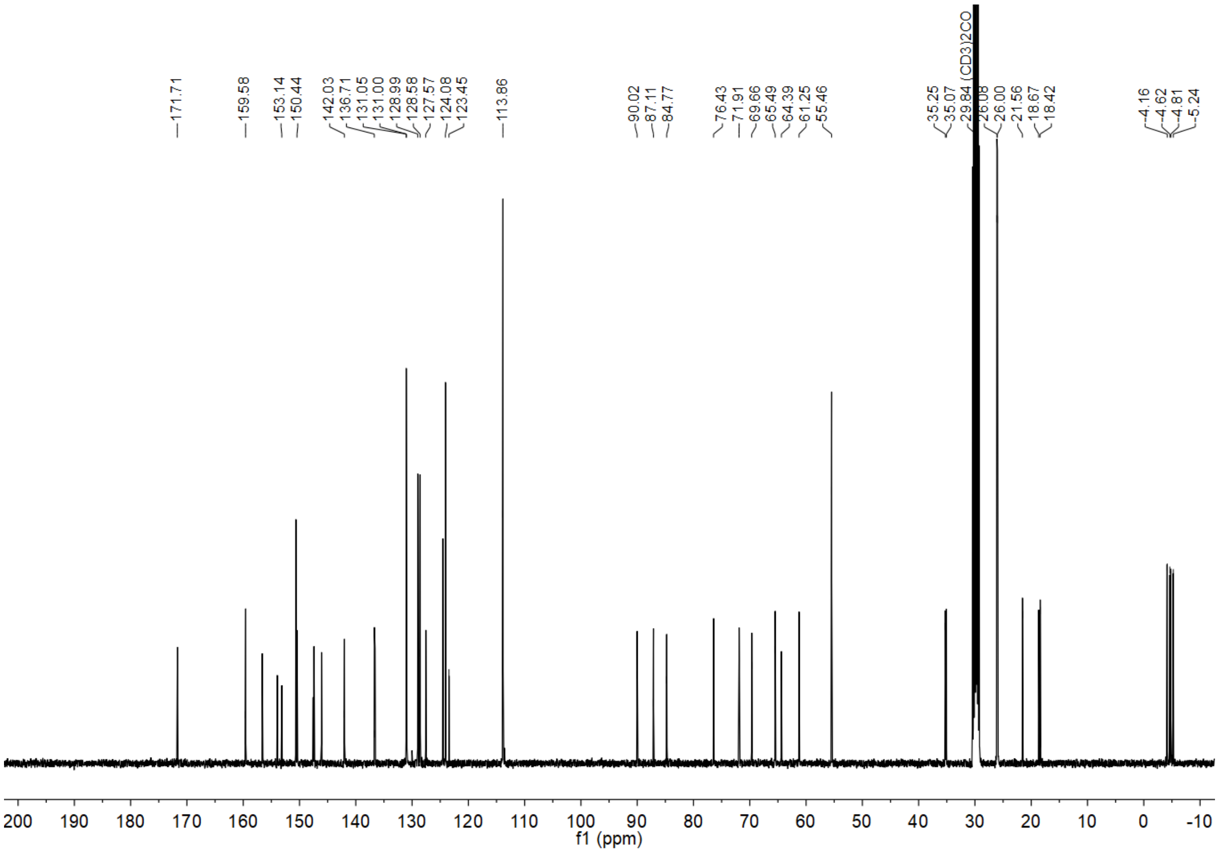

^{1}H and ^{13}C{^{1}H} NMR spectra of compound 13f

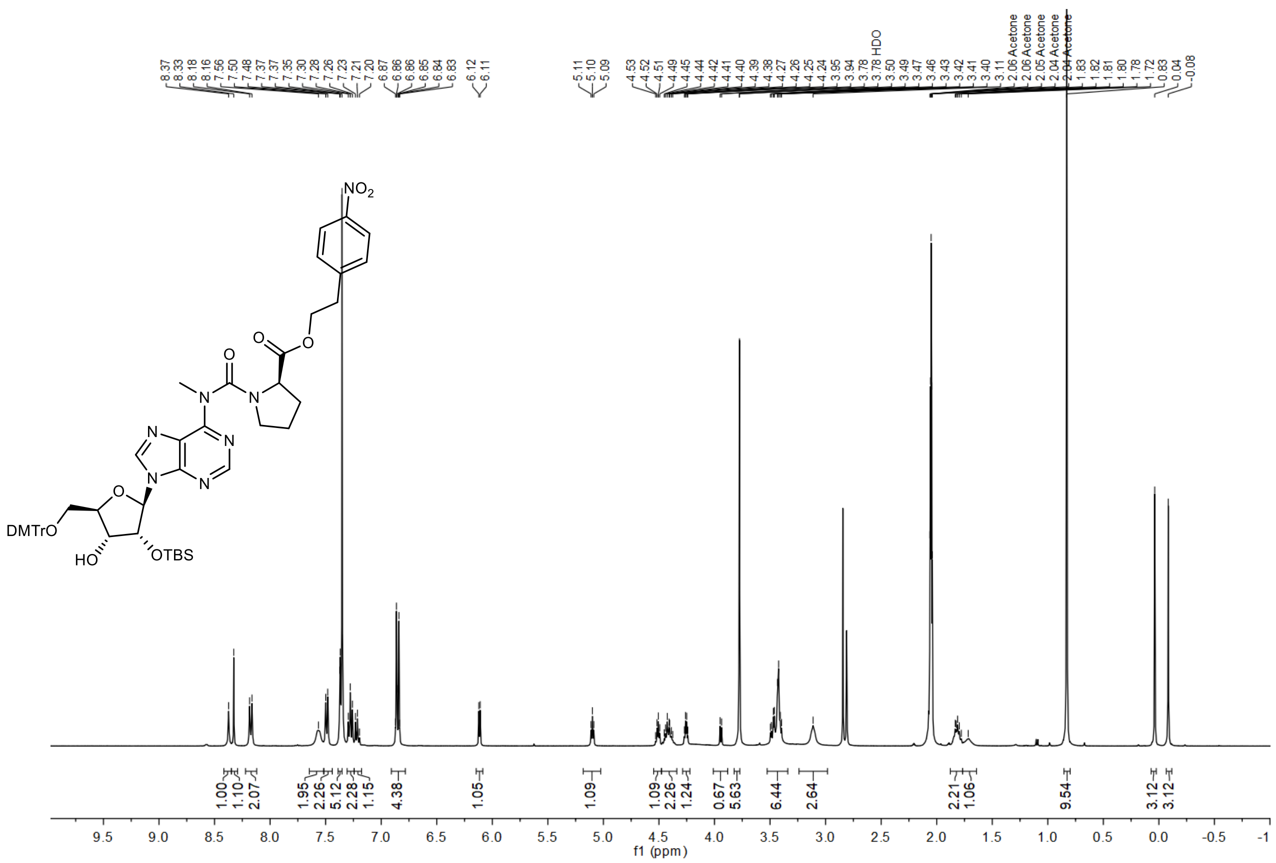

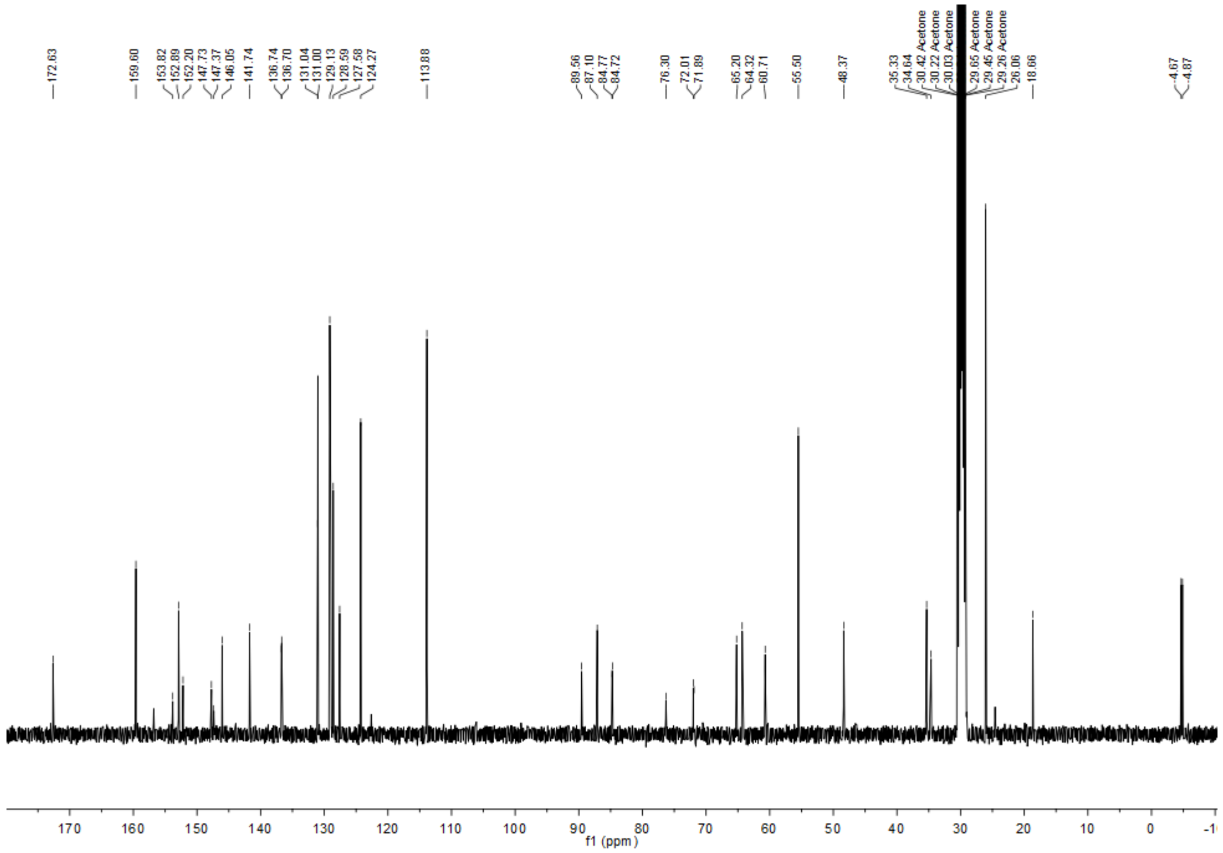

^{1}H and $^{13}C\{^{1}H\}$ NMR spectra of compound 13g

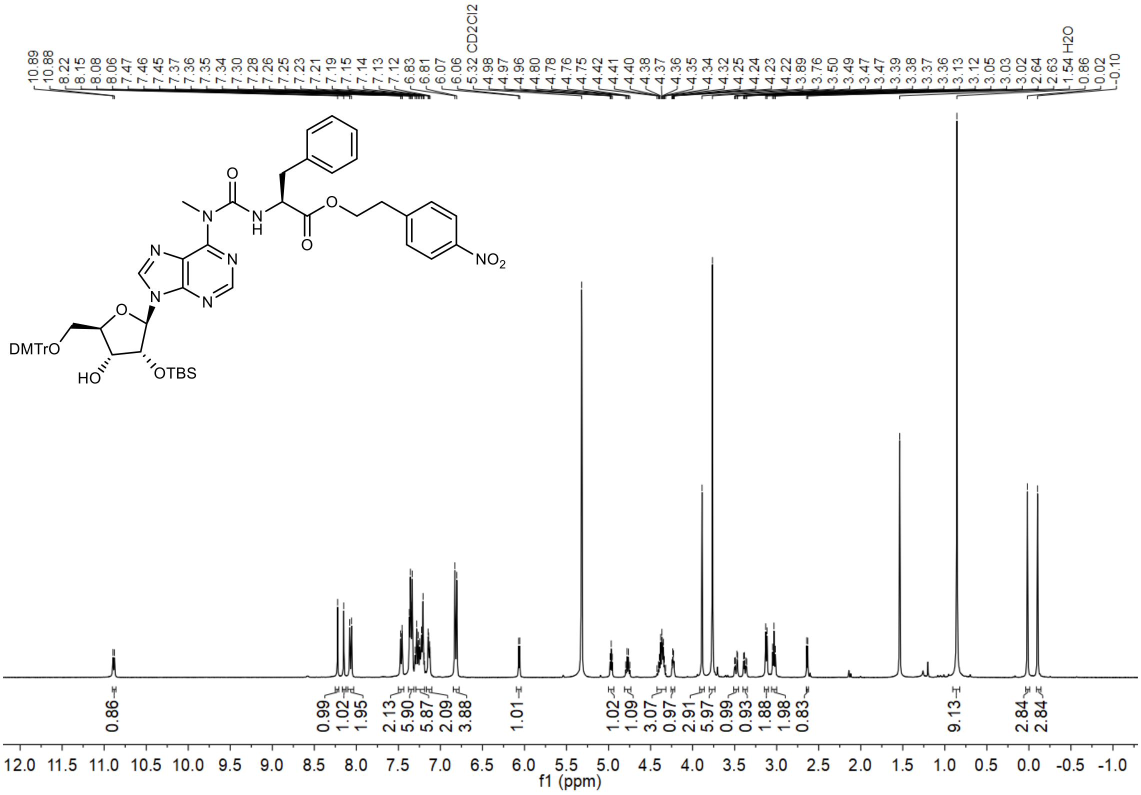

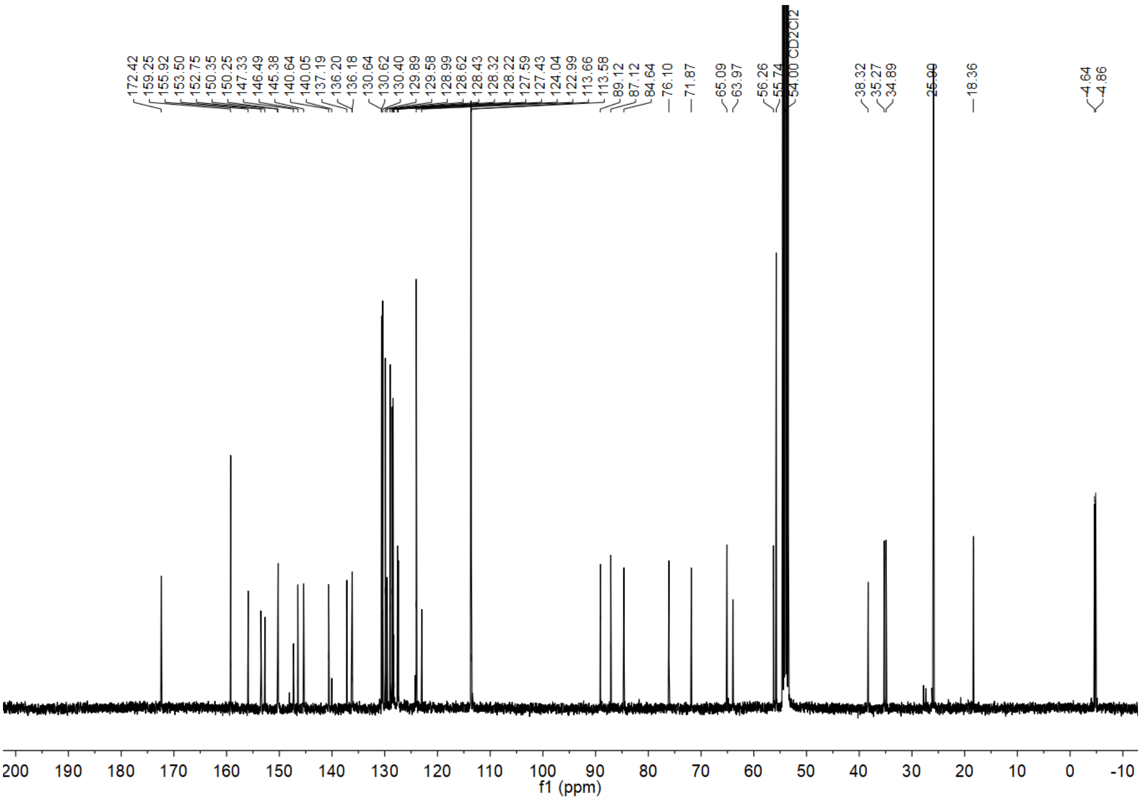

^{1}H and $^{13}C\{^{1}H\}$ NMR spectra of compound 13h

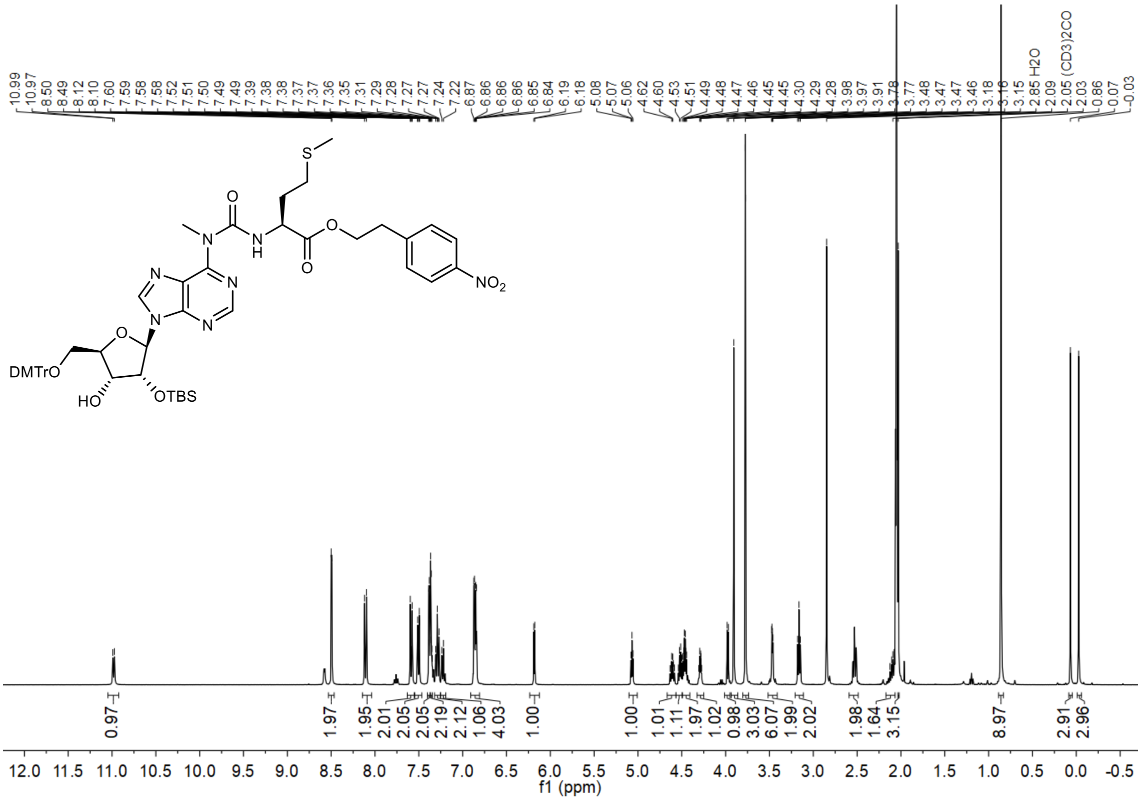

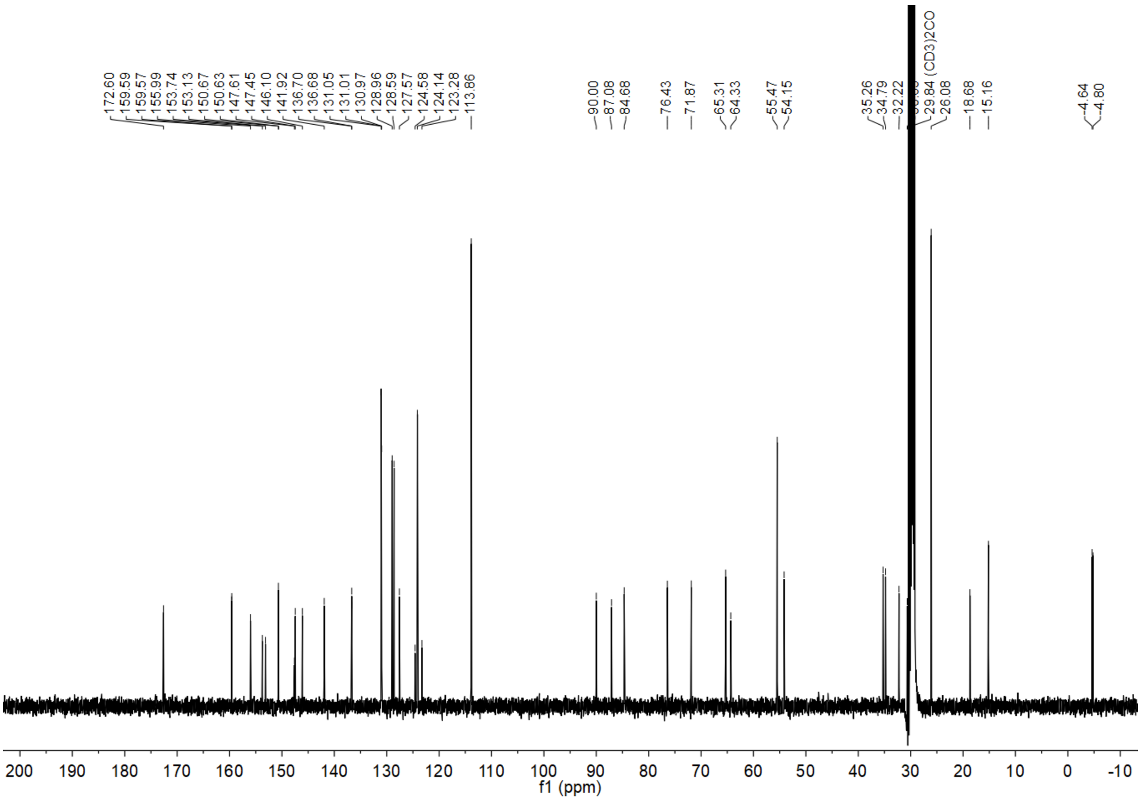

^{1}H and $^{13}C\{^{1}H\}$ NMR spectra of compound 13i

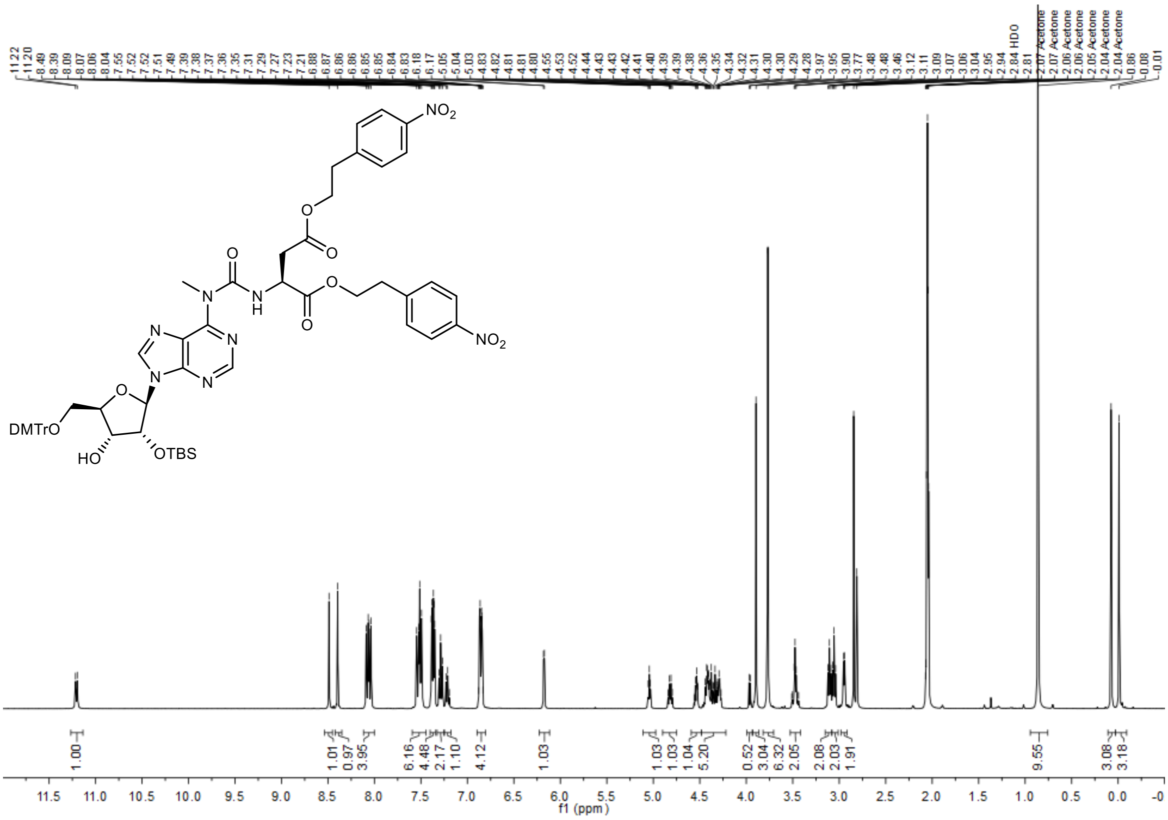

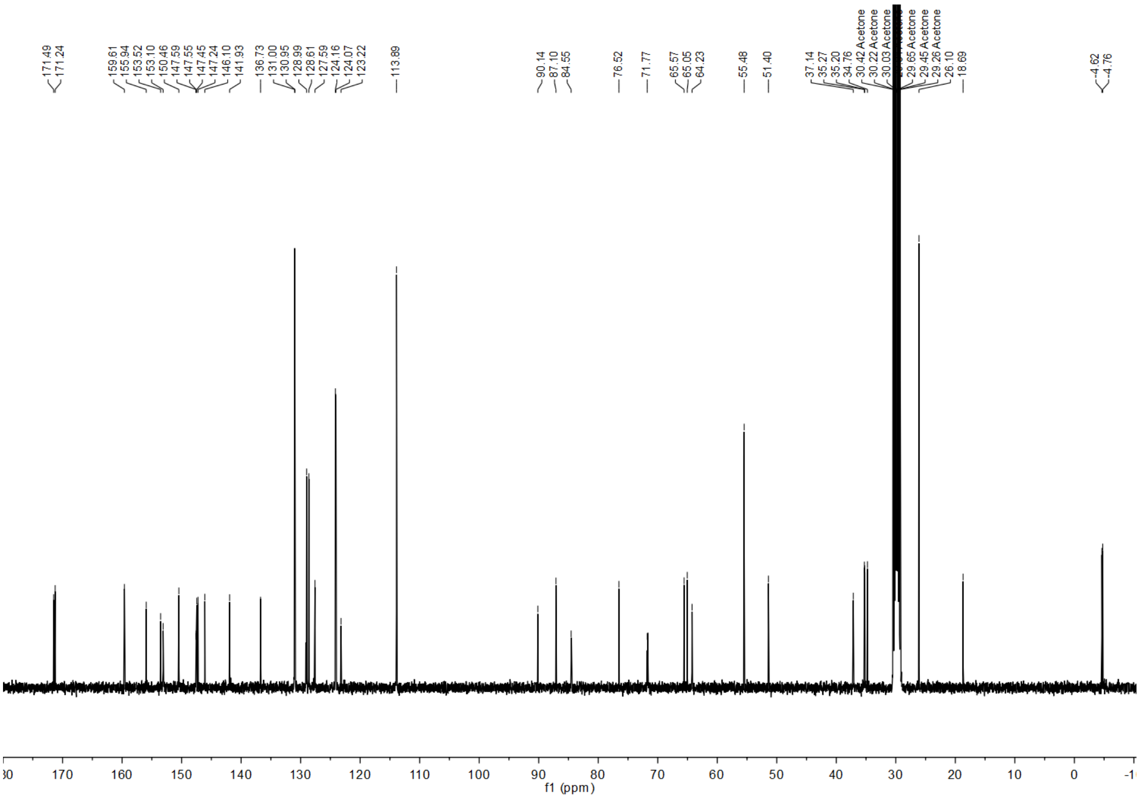

^{1}H and ^{13}C{^{1}H} NMR spectra of compound 13j

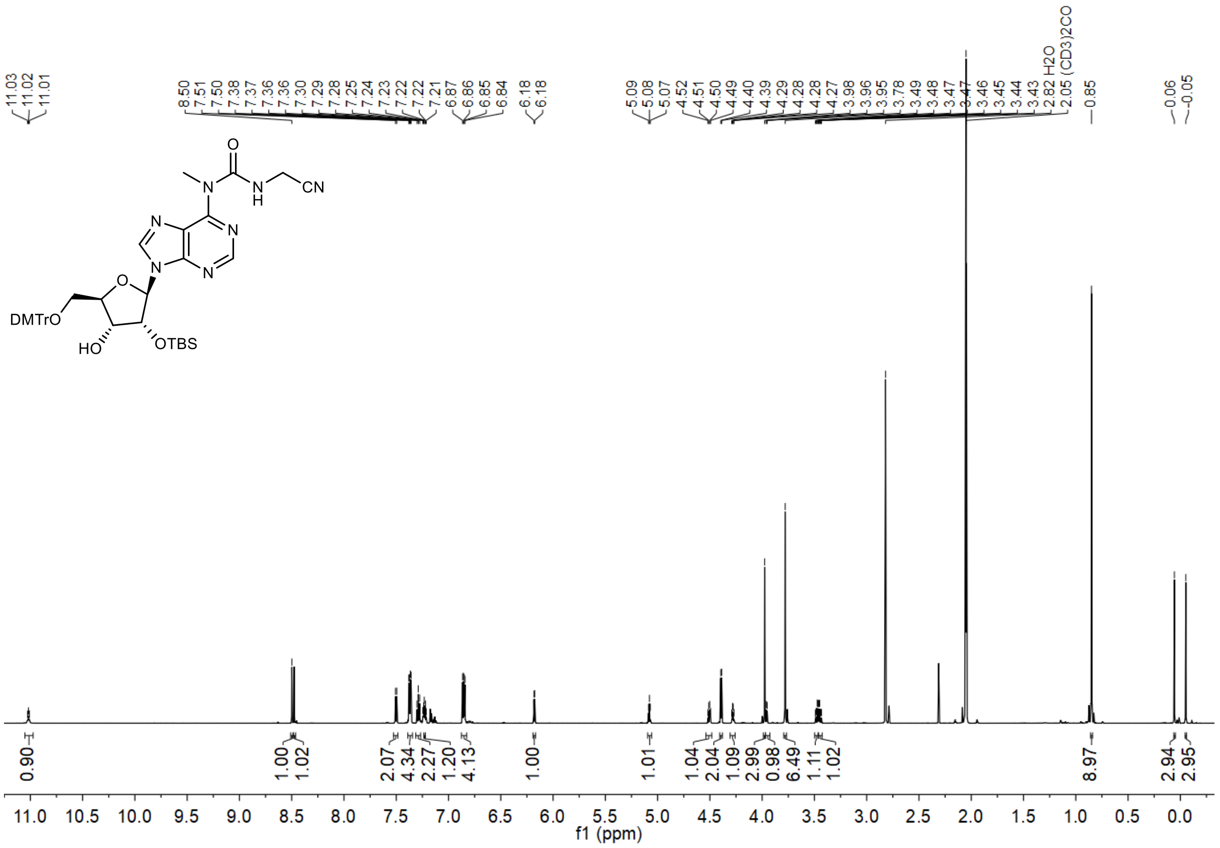

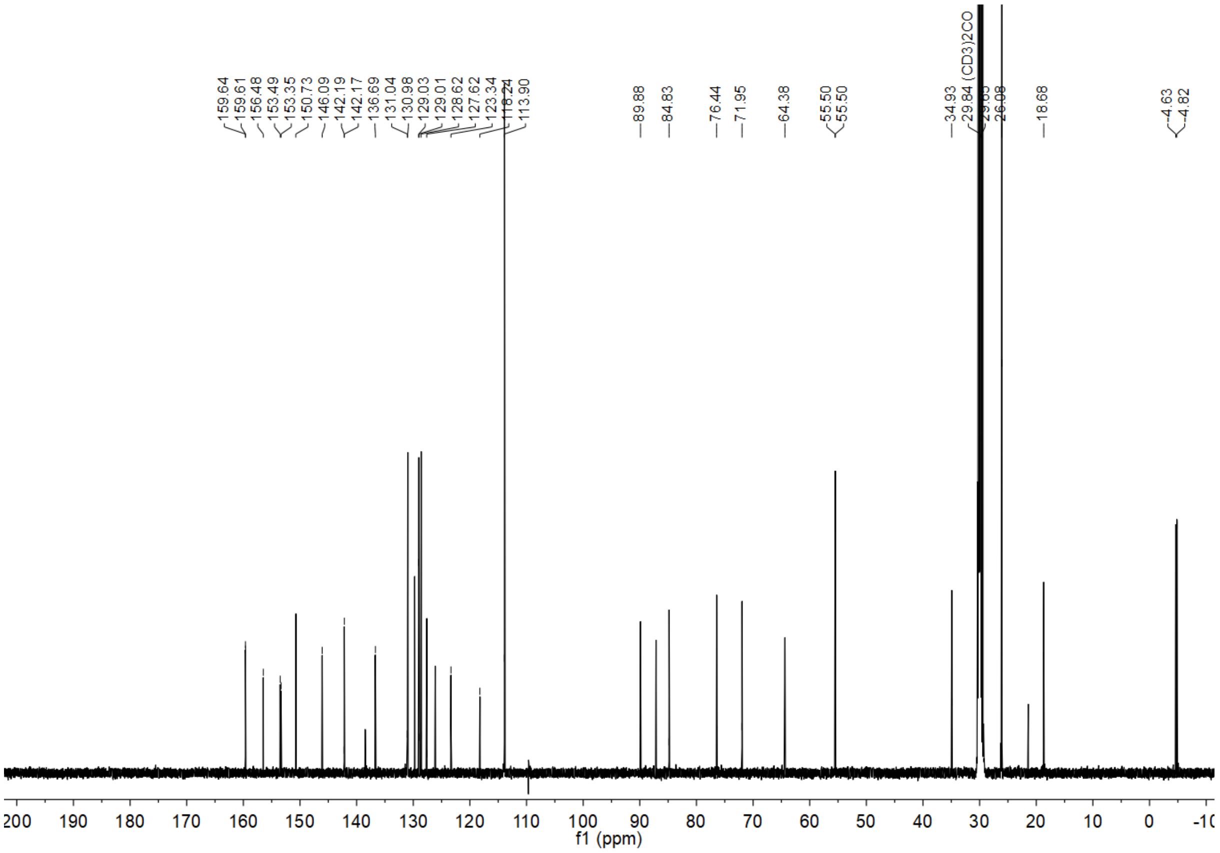

$^{31}P\{^1H\}$ NMR spectrum of compound 14a

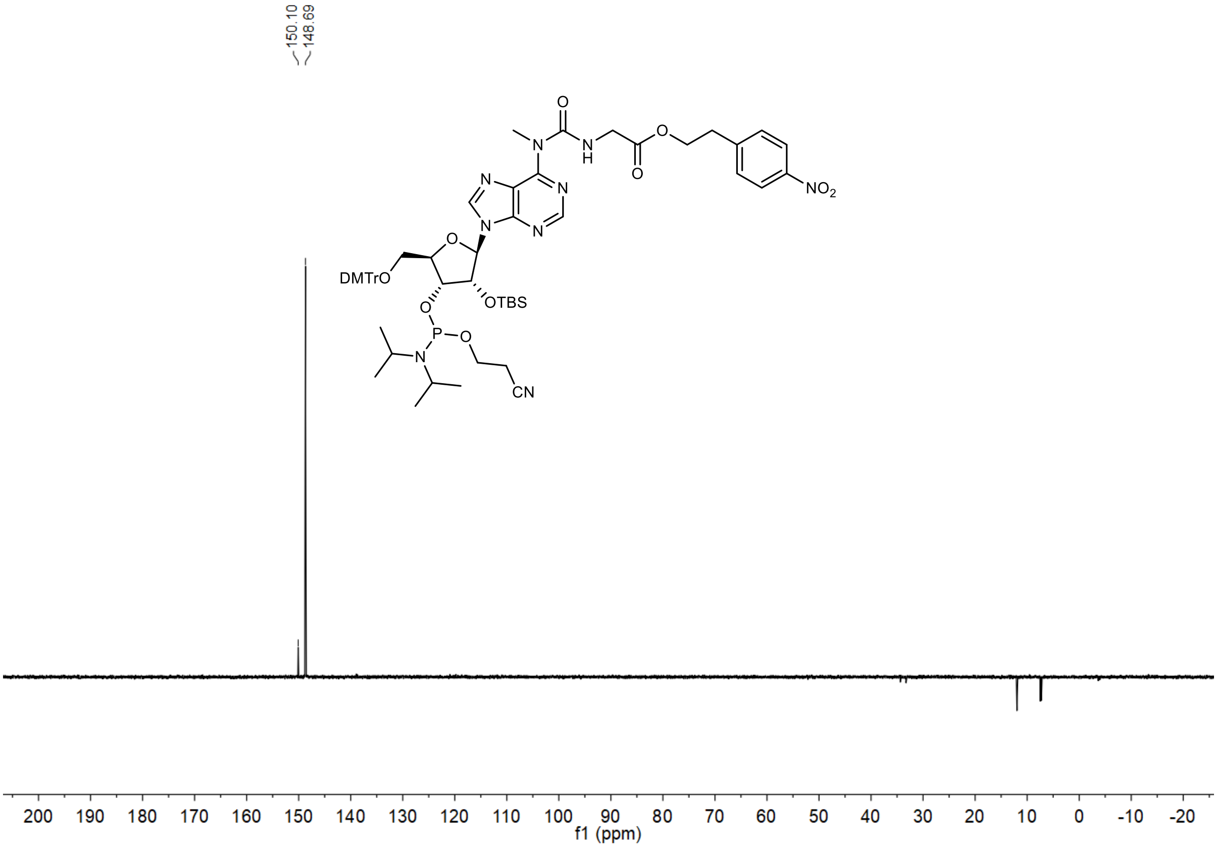

$^{31}P\{^1H\}$ NMR spectrum of compound 14b

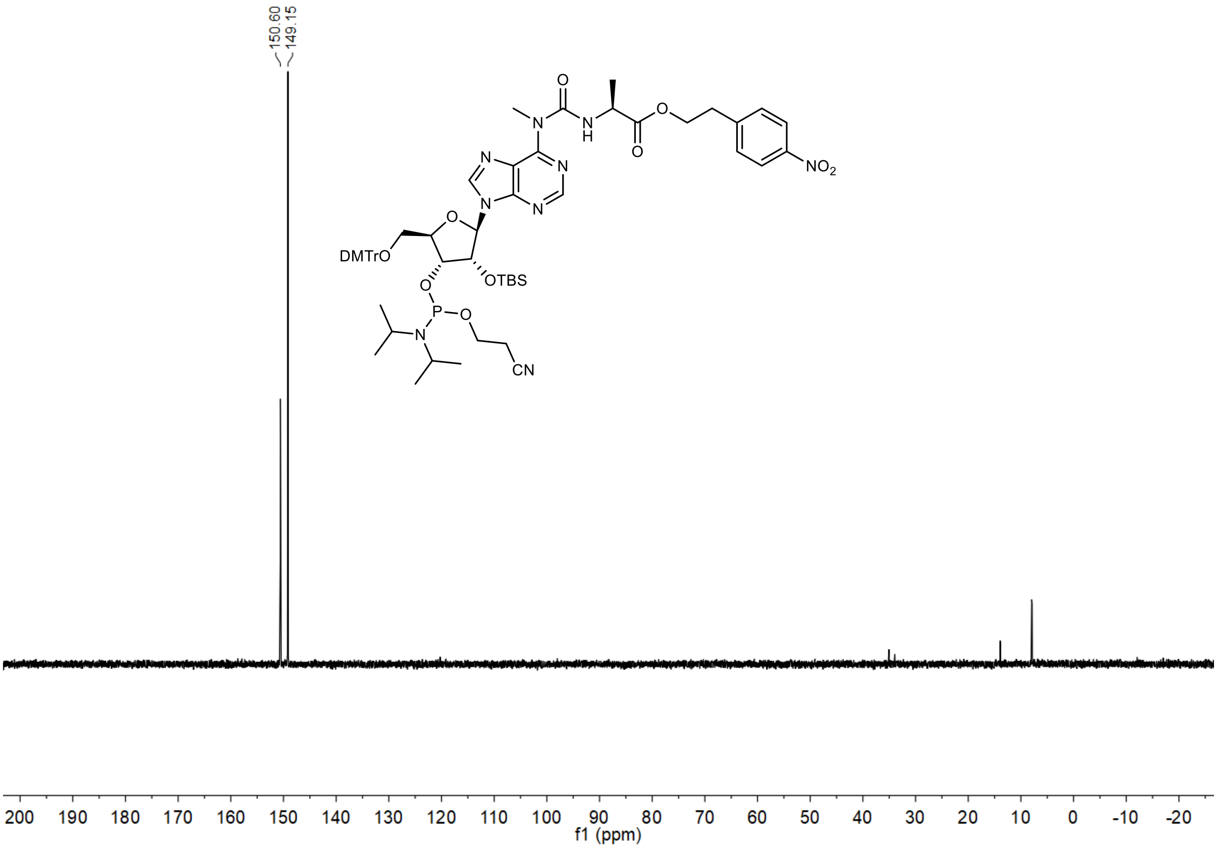

$^{31}P\{^1H\}$ NMR spectrum of compound 14c

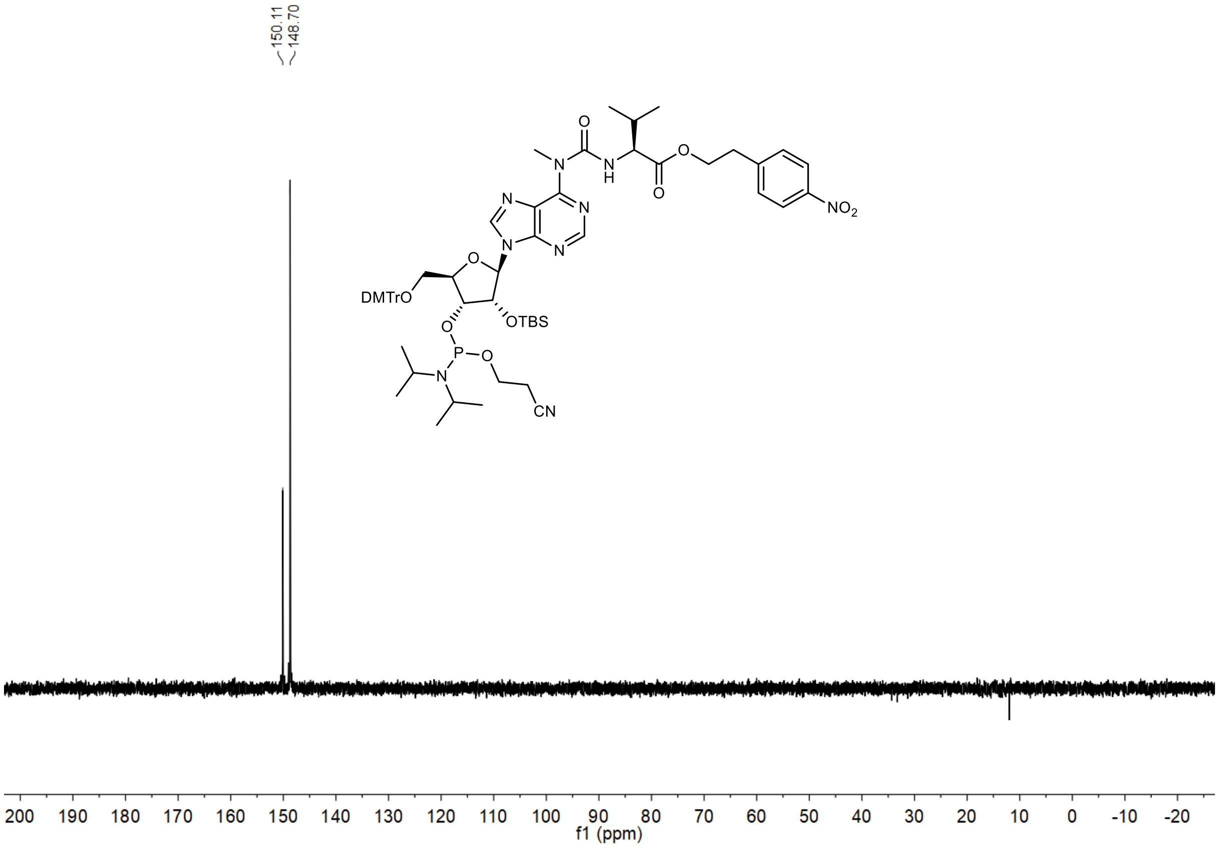

$^{31}P\{^1H\}$ NMR spectrum of compound 14d

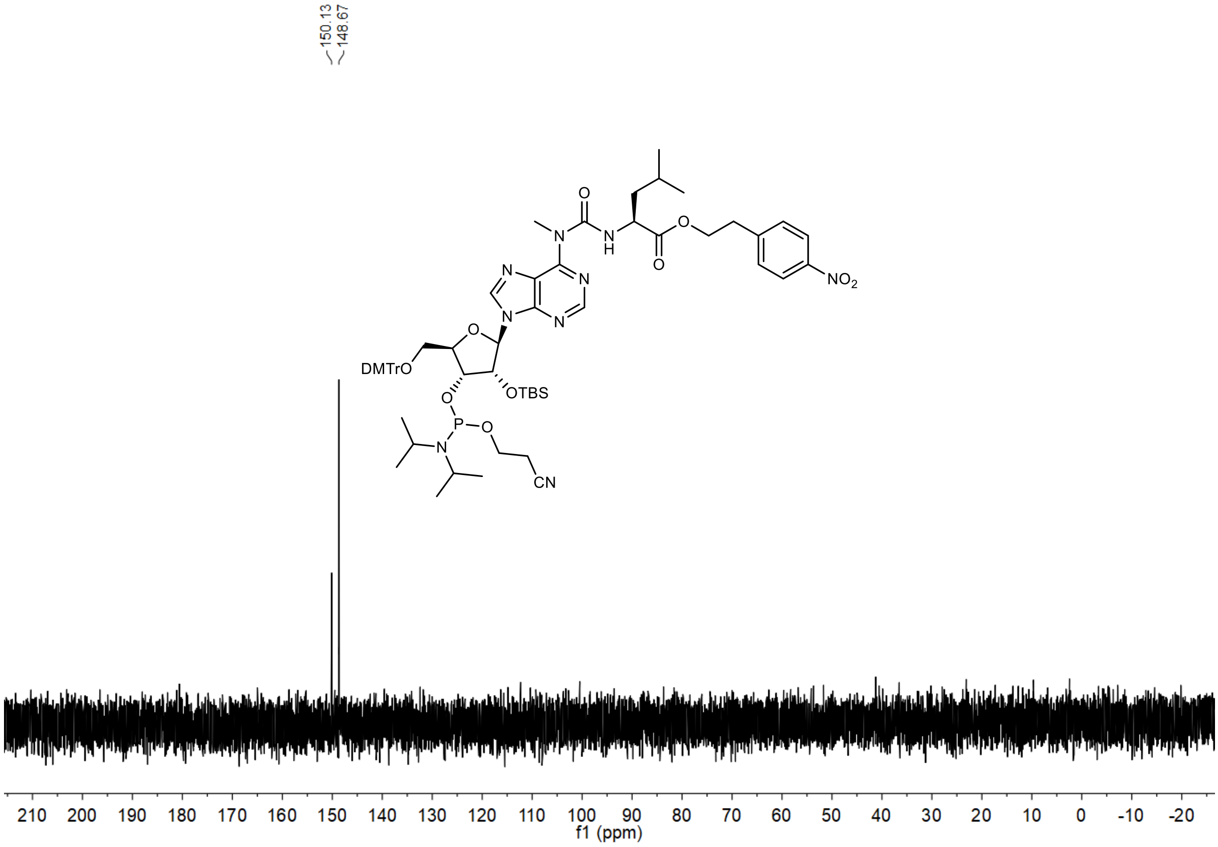

$^{31}P\{^{1}H\}$ NMR spectrum of compound 14e

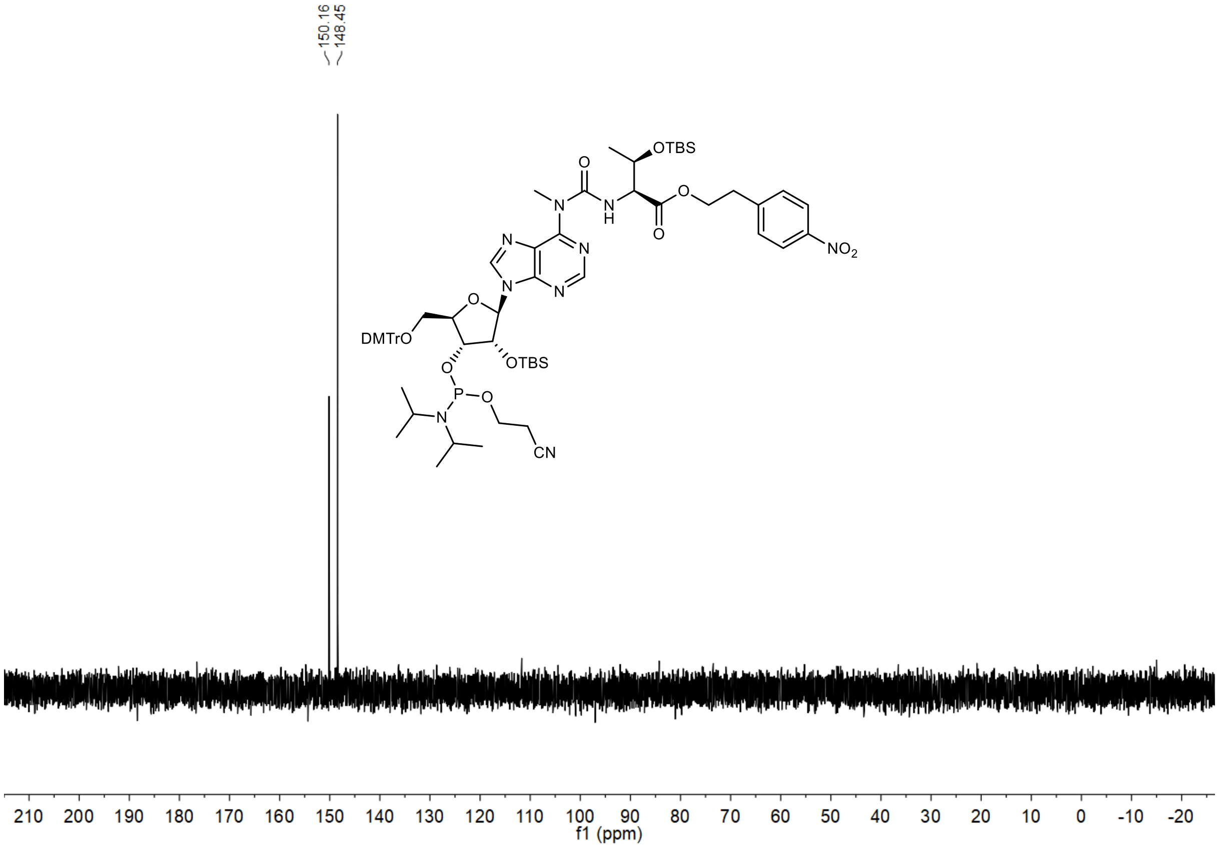

$^{31}P\{^{1}H\}$ NMR spectrum of compound 14f

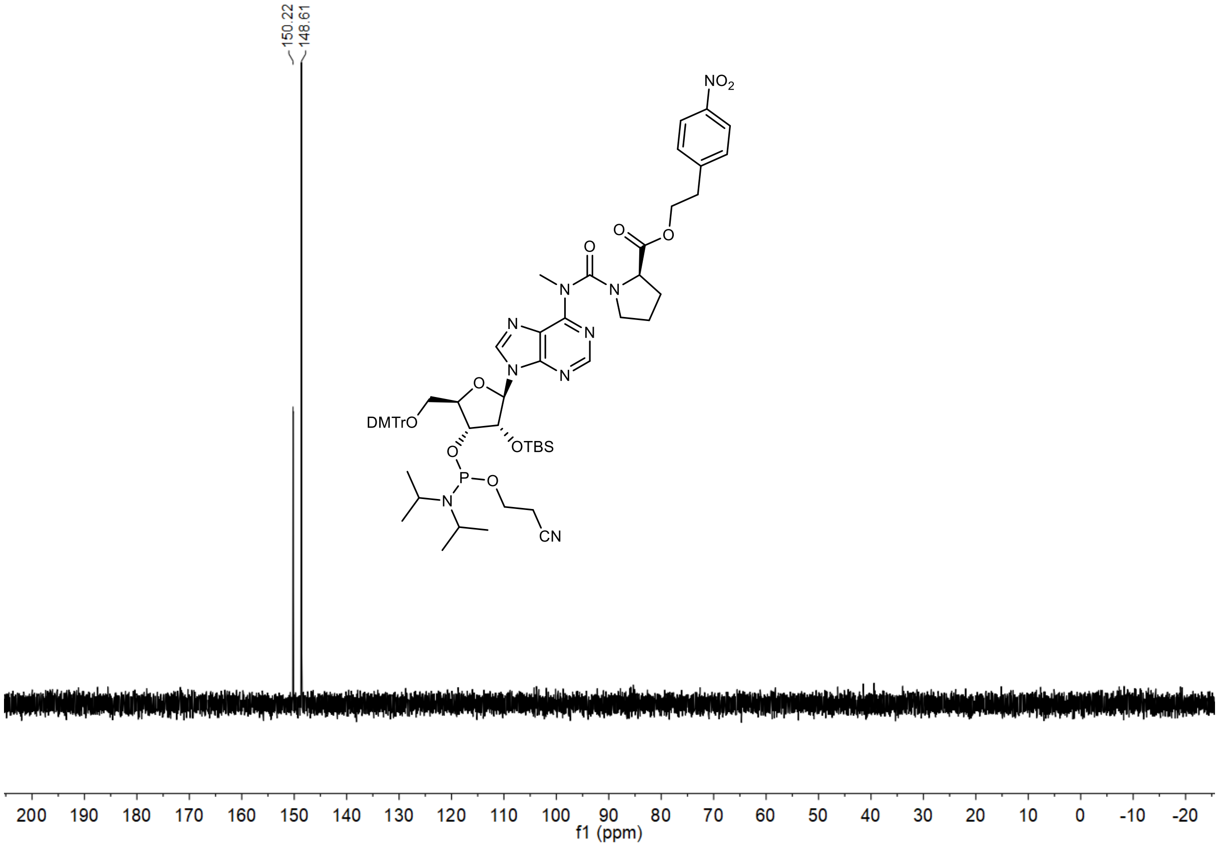

$^{31}P\{^1H\}$ NMR spectrum of compound 14g

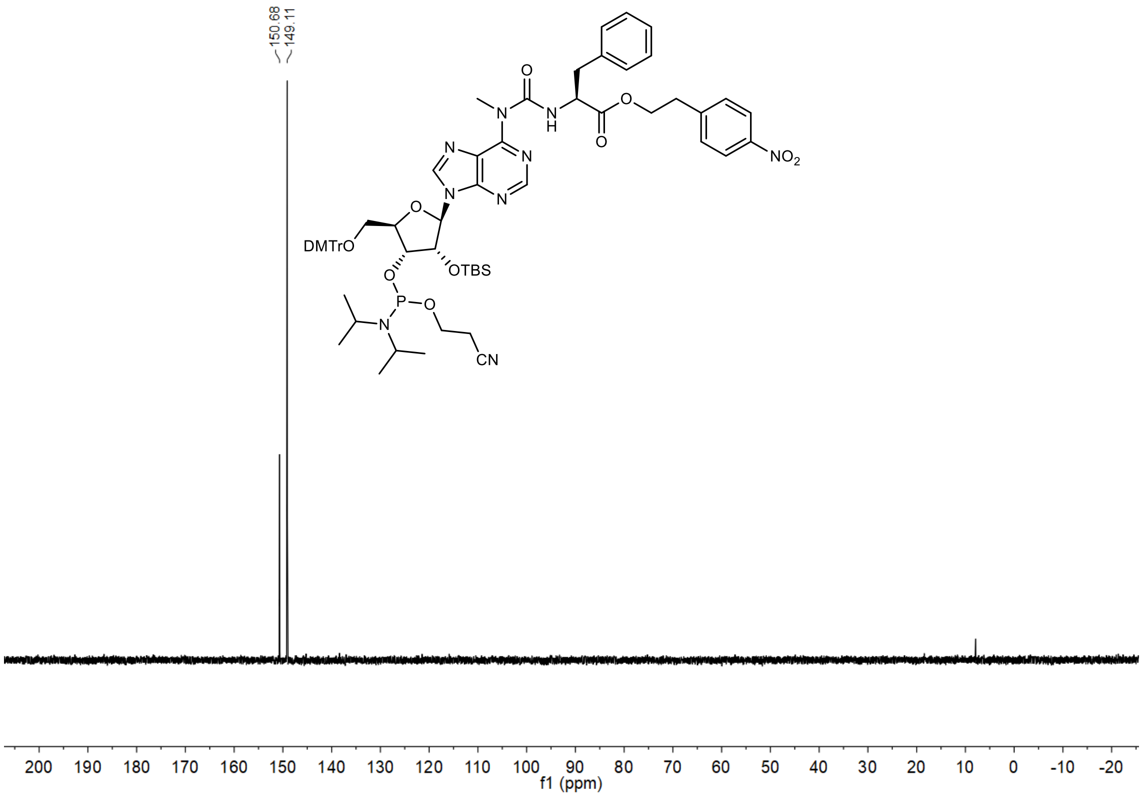

$^{31}P\{^1H\}$ NMR spectrum of compound 14h

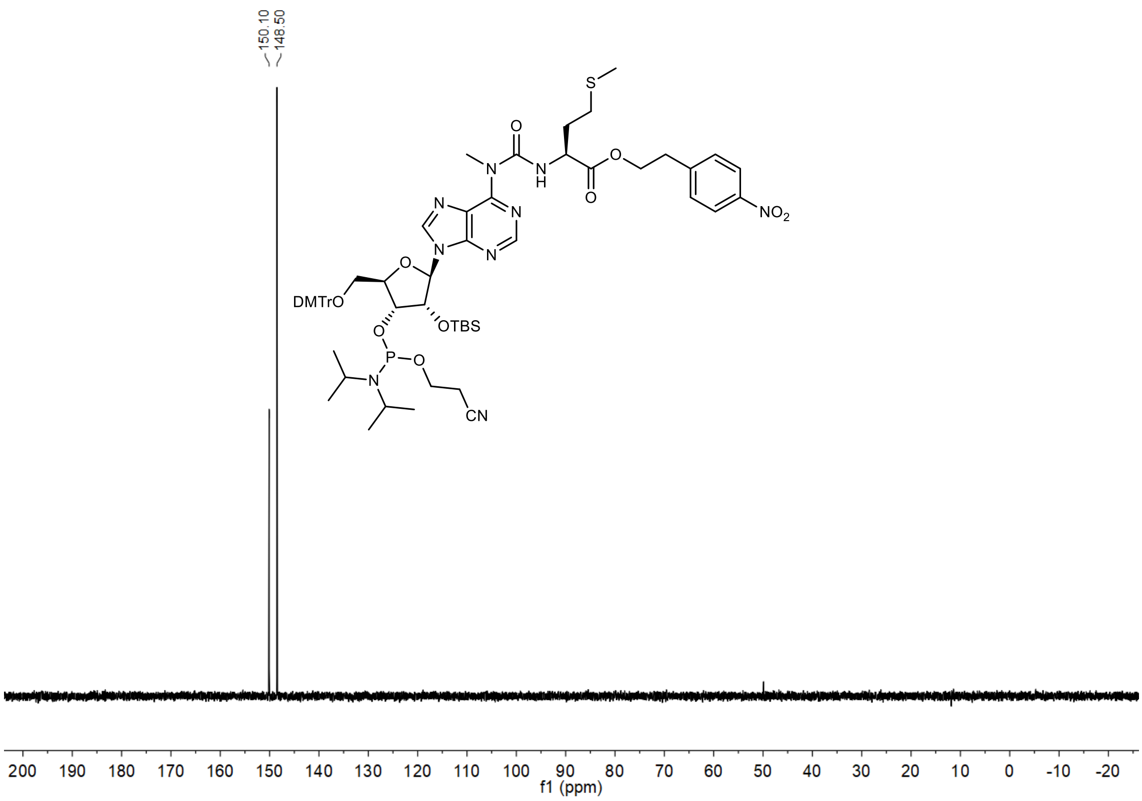

$^{31}P\{^1H\}$ NMR spectrum of compound 14i

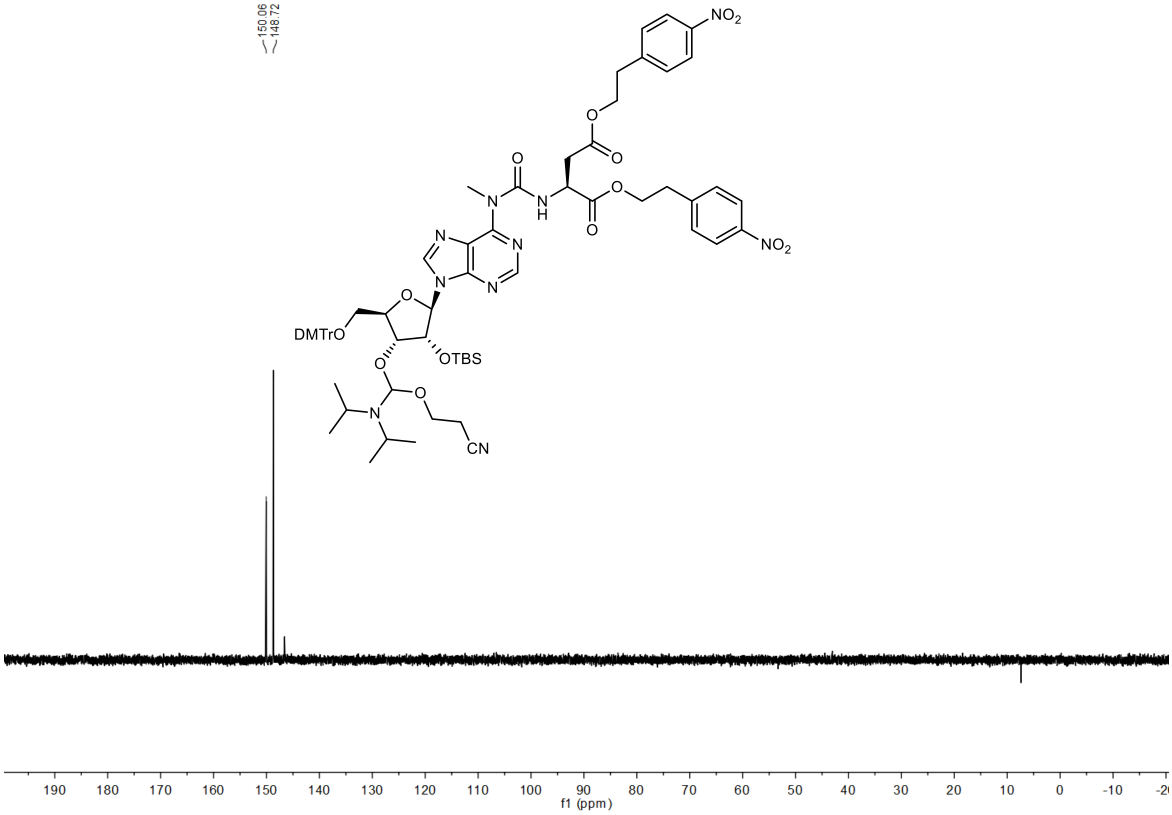

$^{31}P\{^1H\}$ NMR spectrum of compound 14j

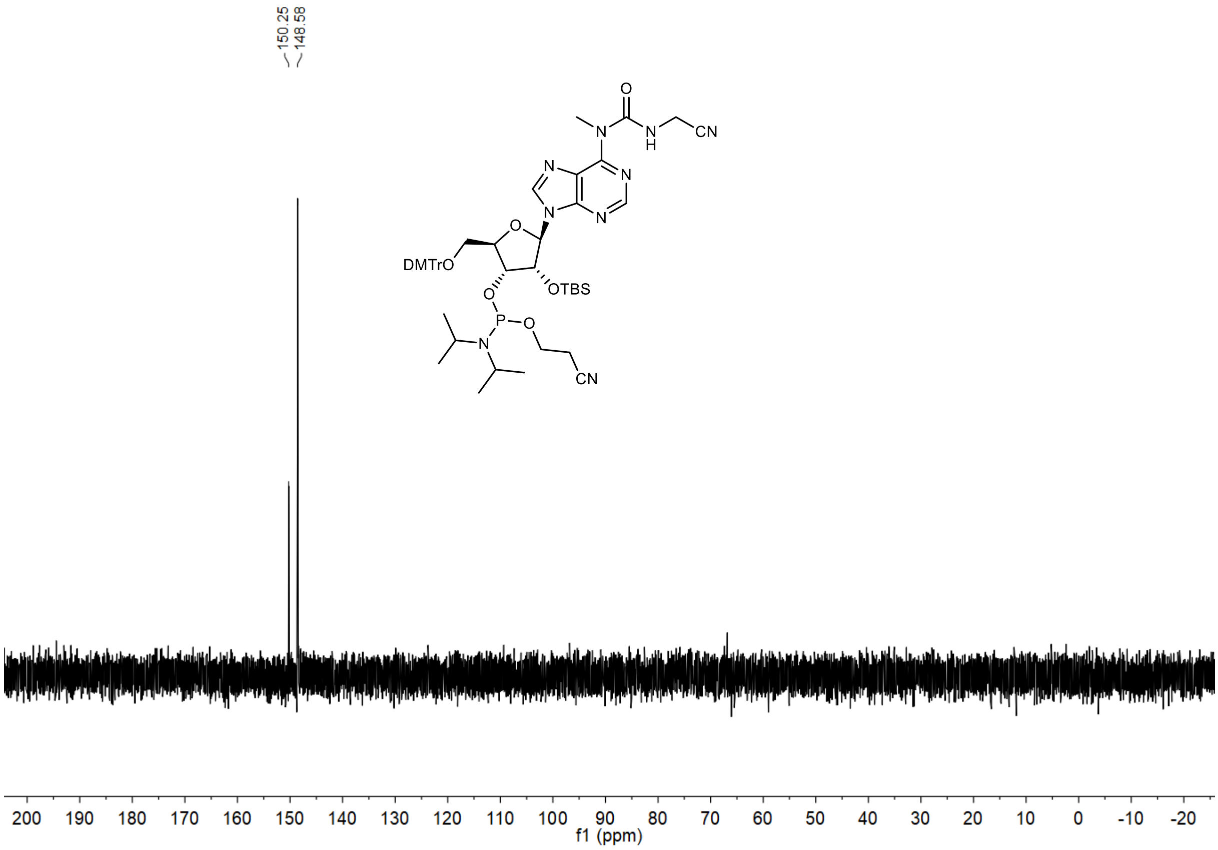

^{1}H and ^{13}C{^{1}H} NMR spectra of compound 16

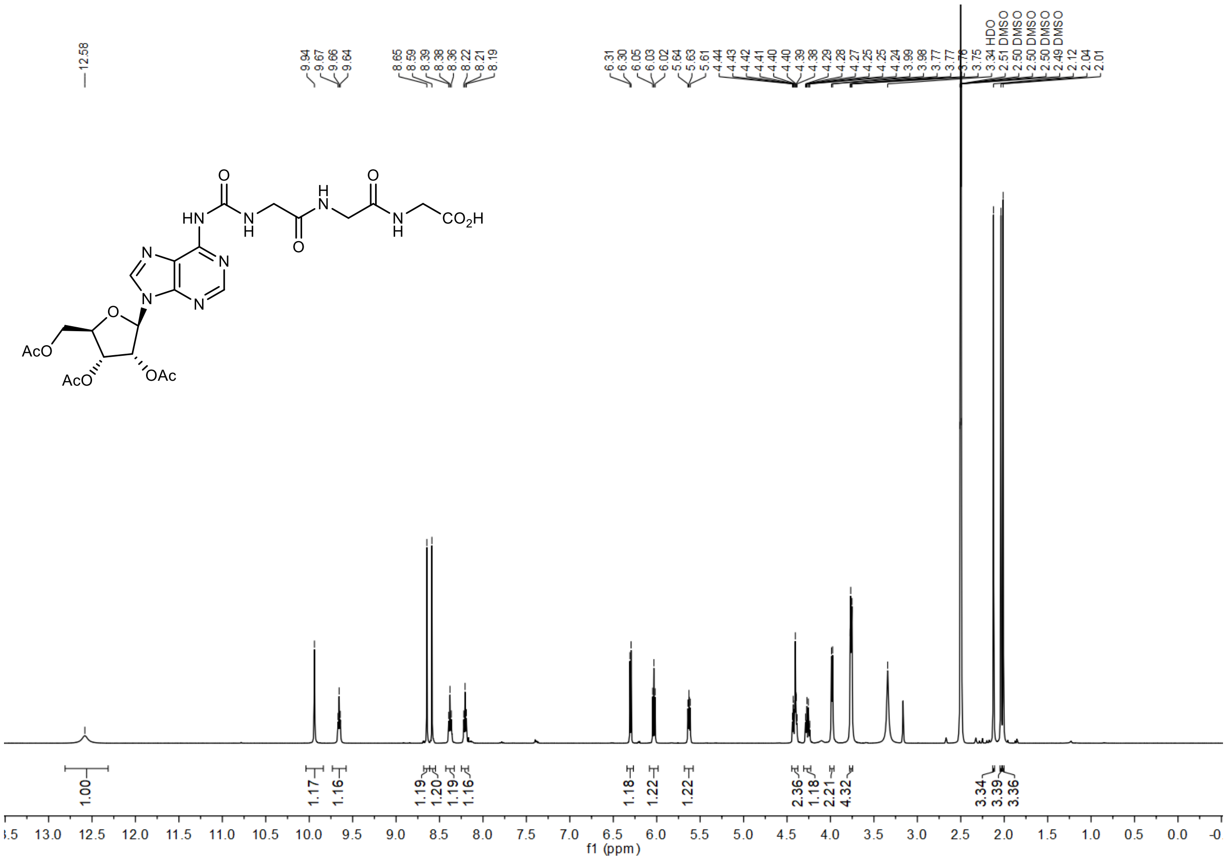

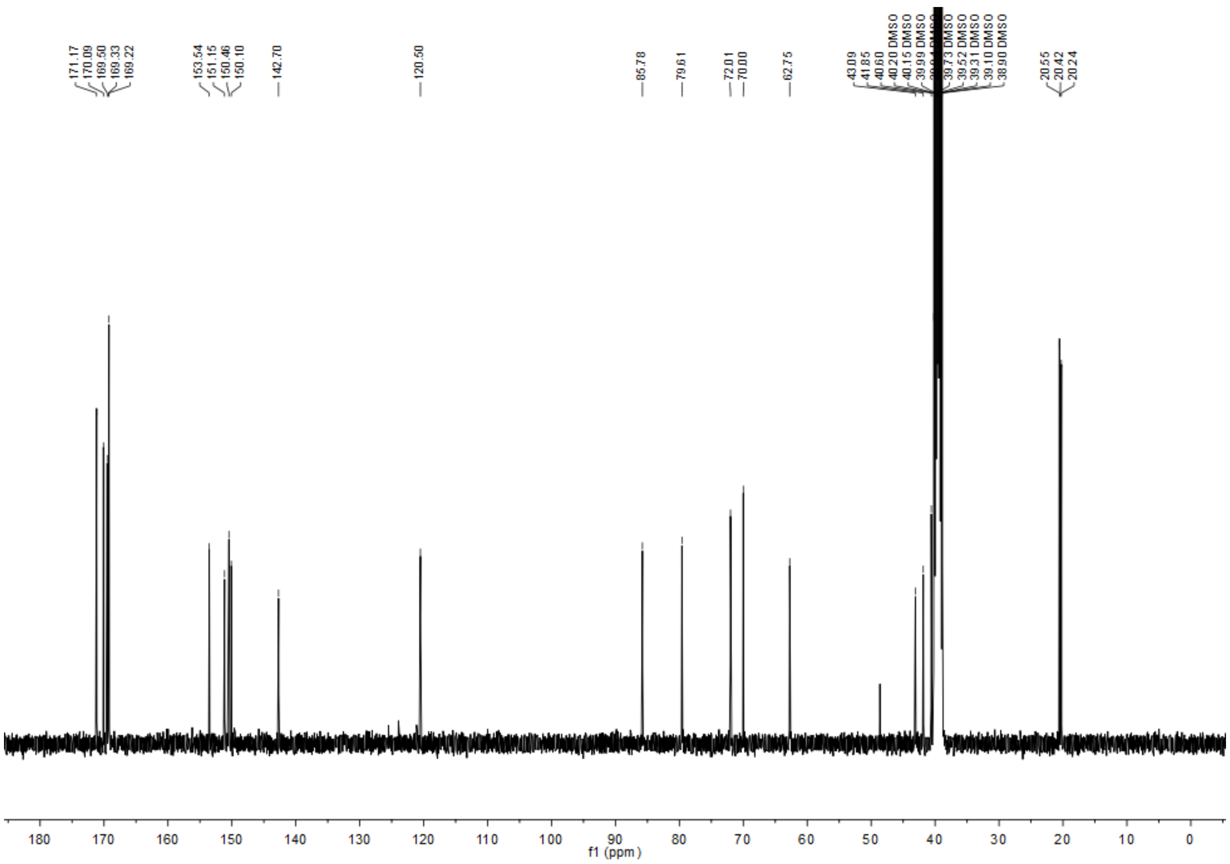

^{1}H and ^{13}C{^{1}H} NMR spectra of compound 17

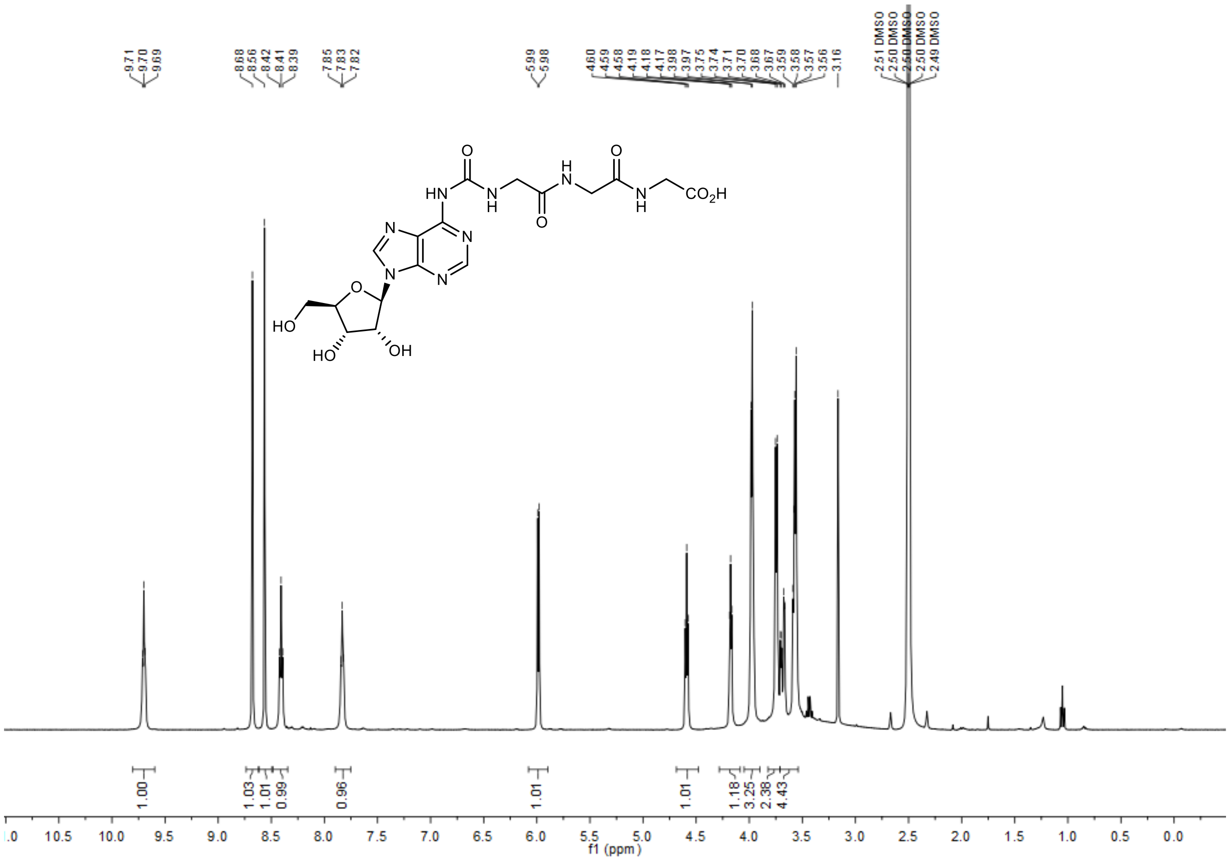

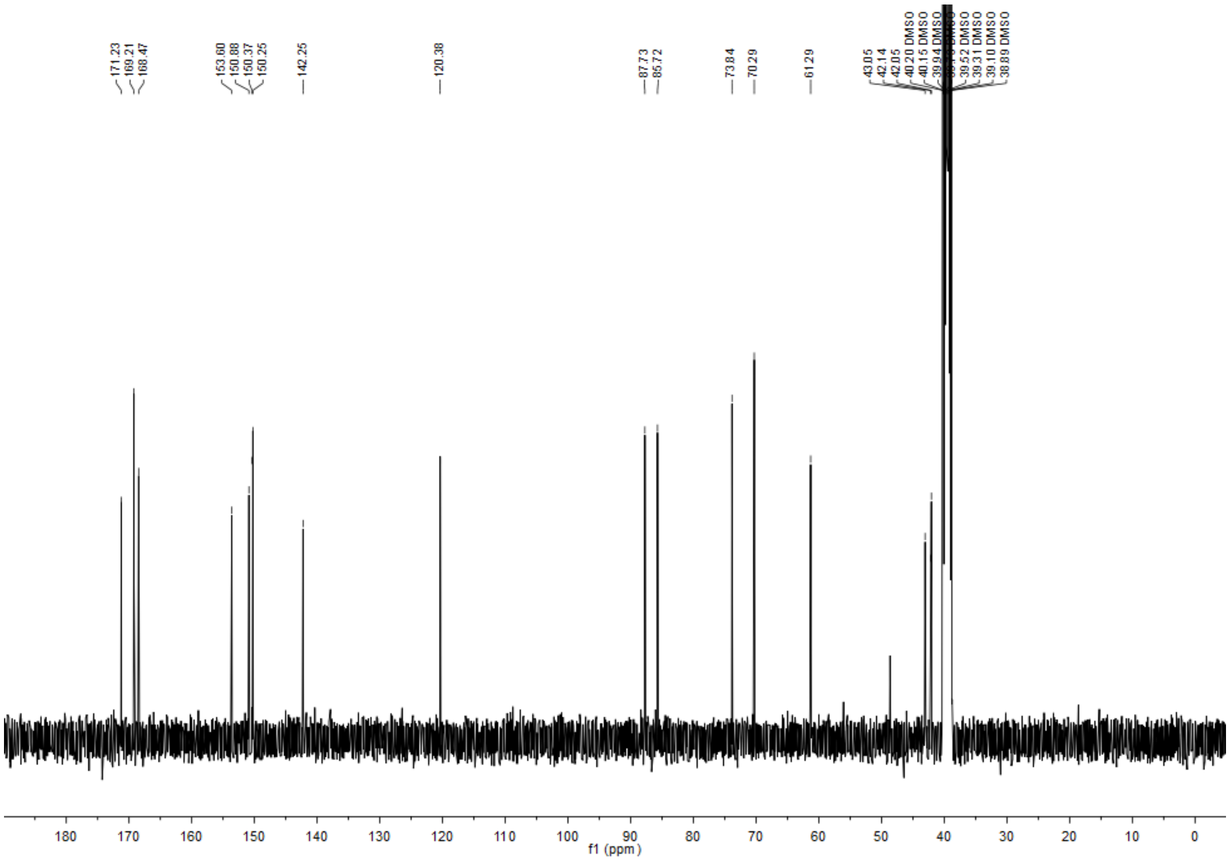

^{1}H and ^{13}C{^{1}H} NMR spectra of compound 18

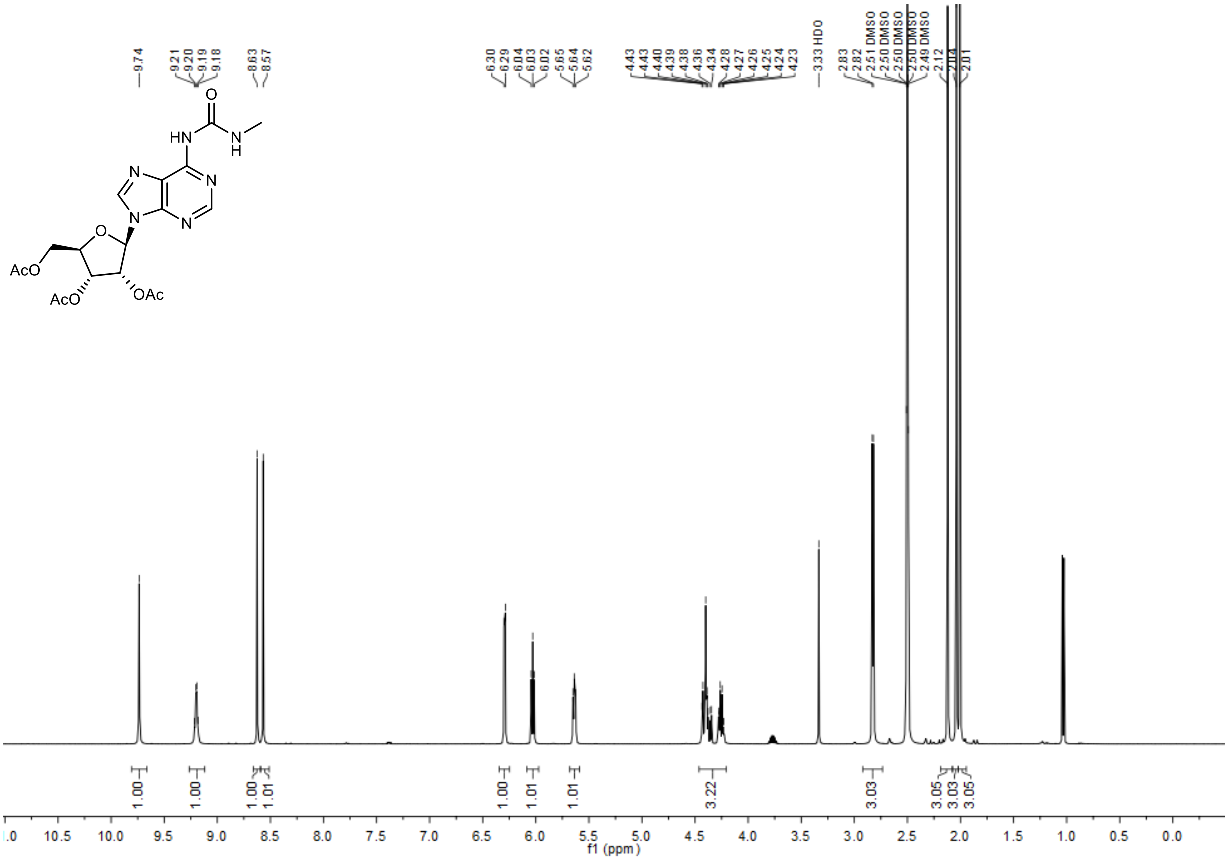

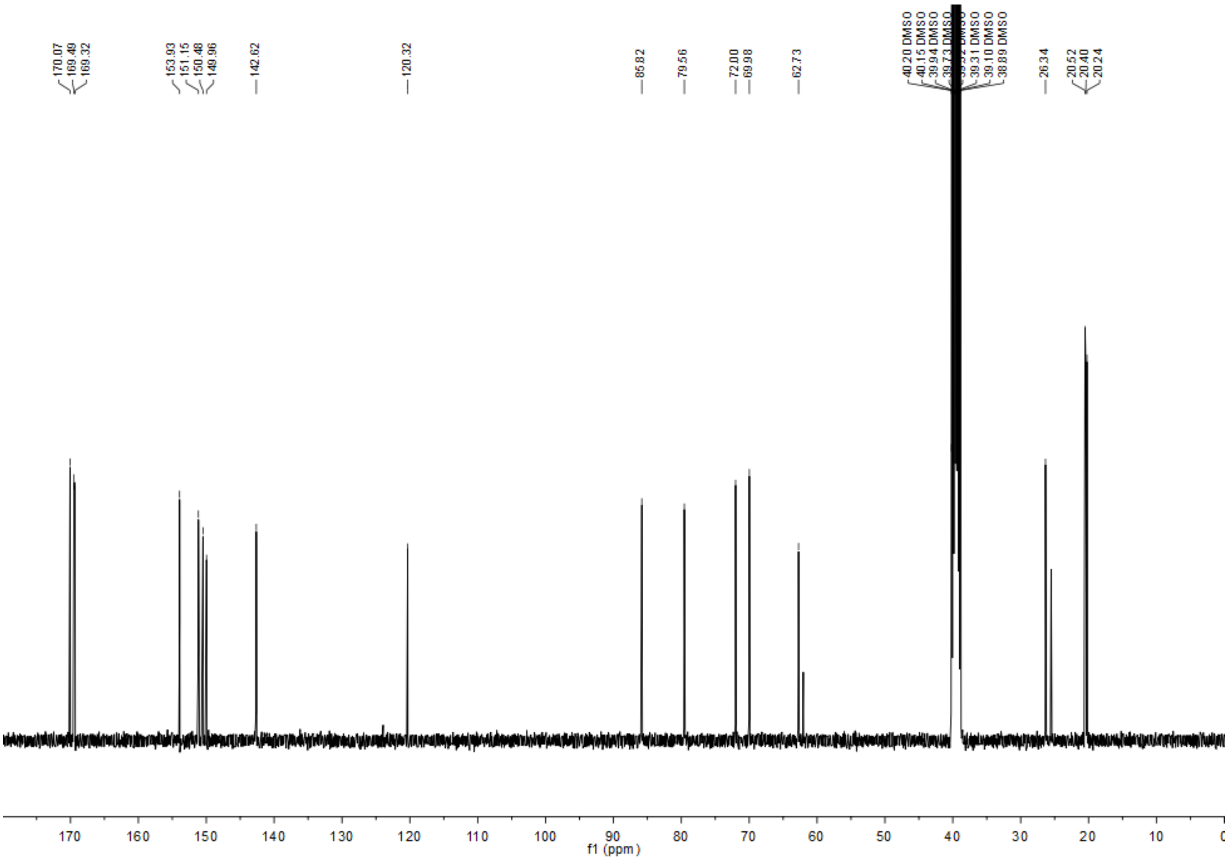

^{1}H and $^{13}C\{^{1}H\}$ NMR spectra of compound 19

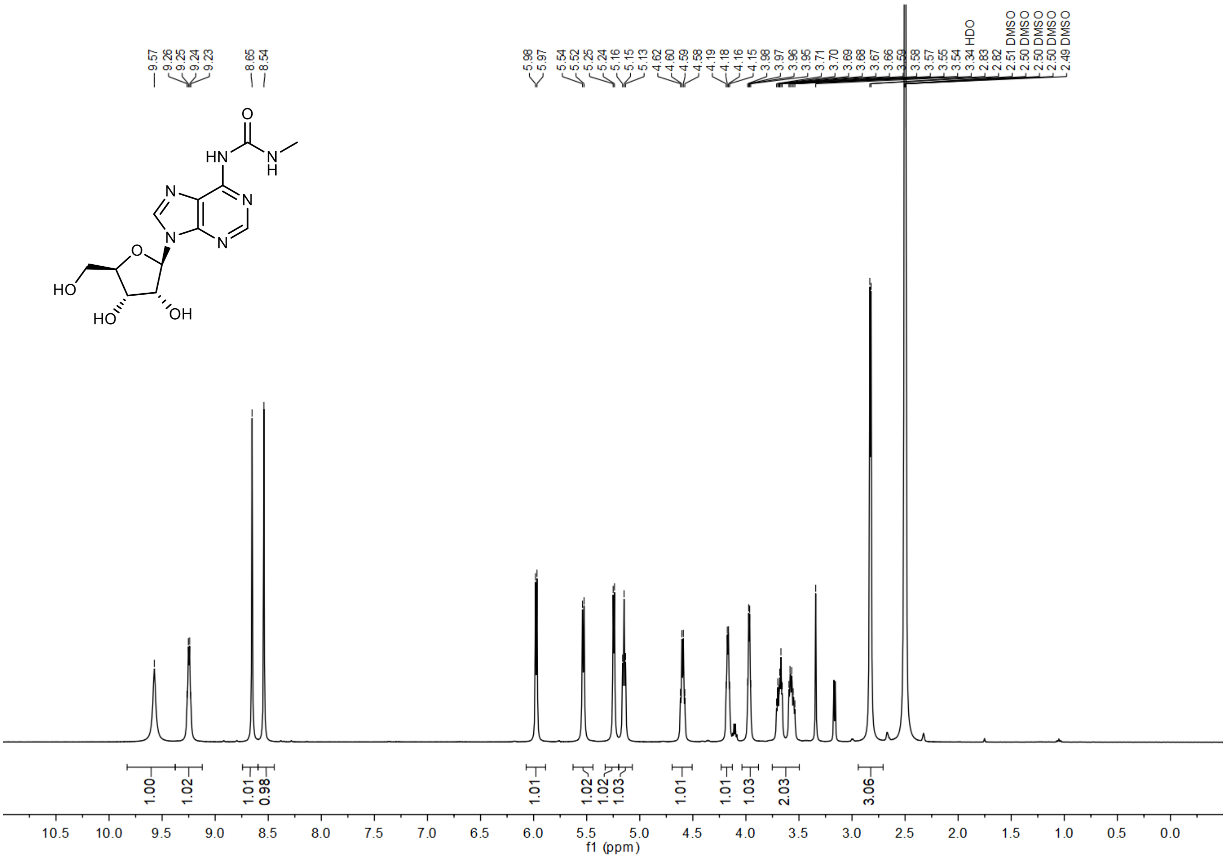

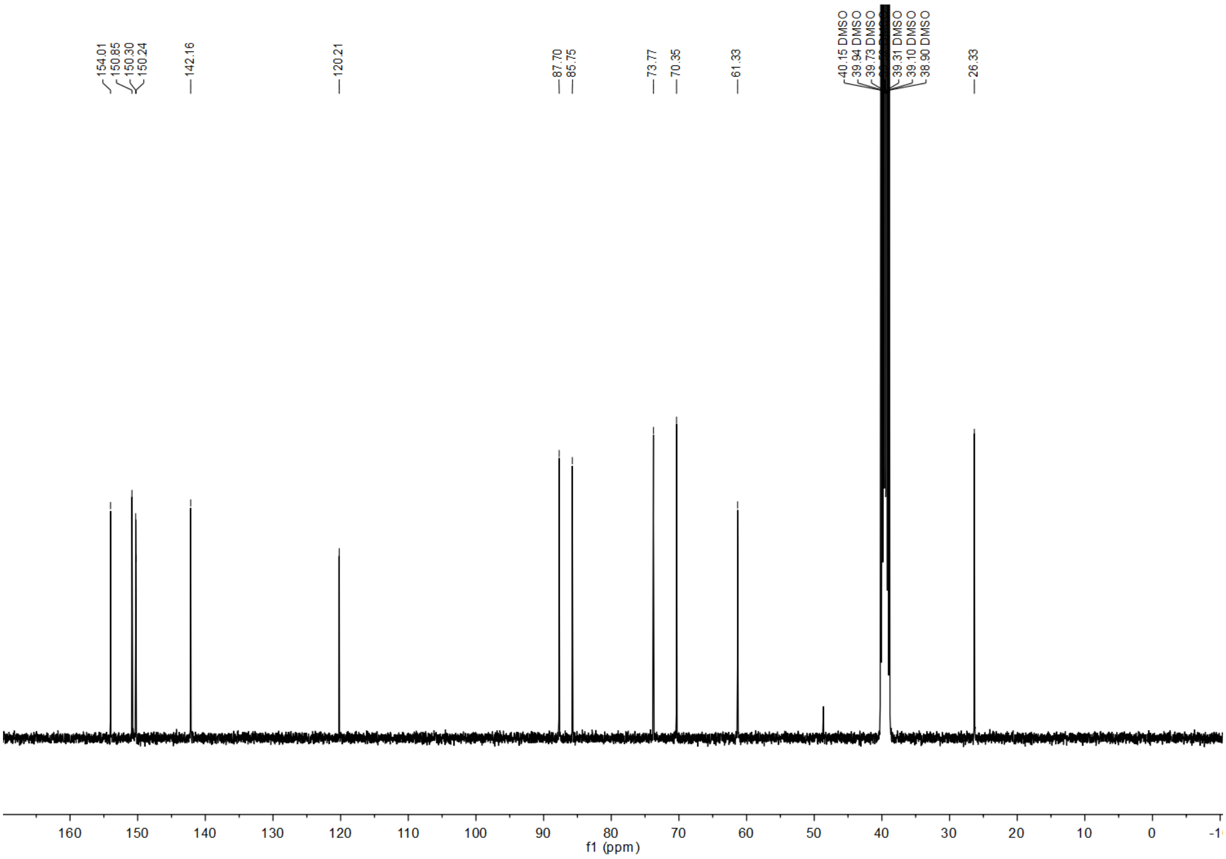

^{1}H and ^{13}C{^{1}H} NMR spectra of compound 21

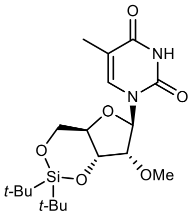

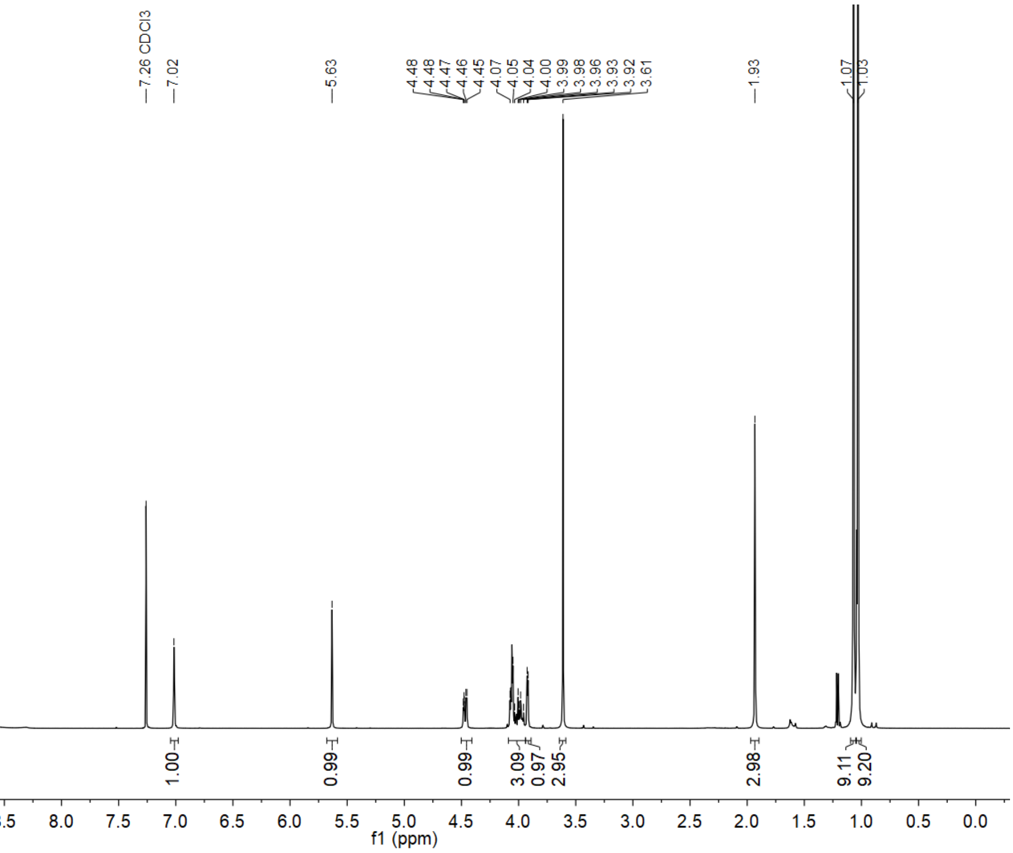

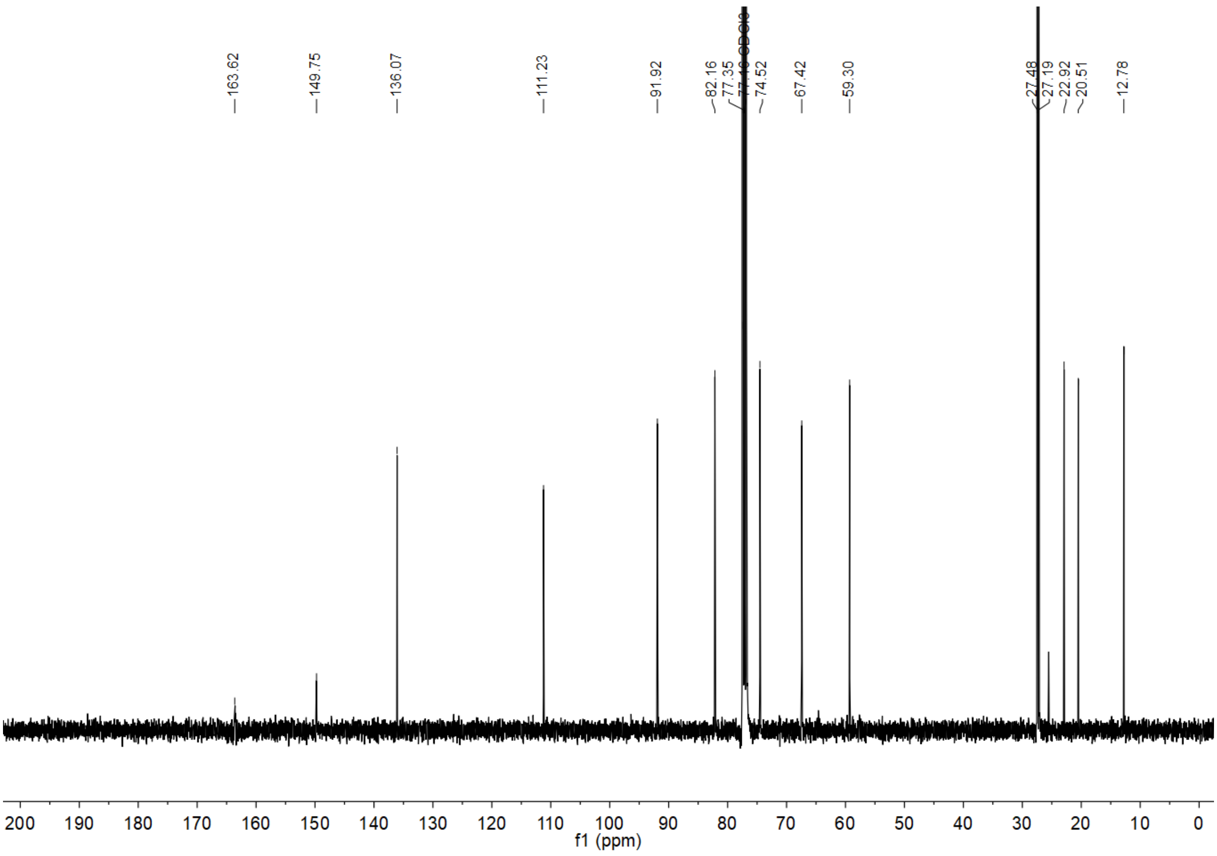

^{1}H and ^{13}C{^{1}H} NMR spectra of compound 22

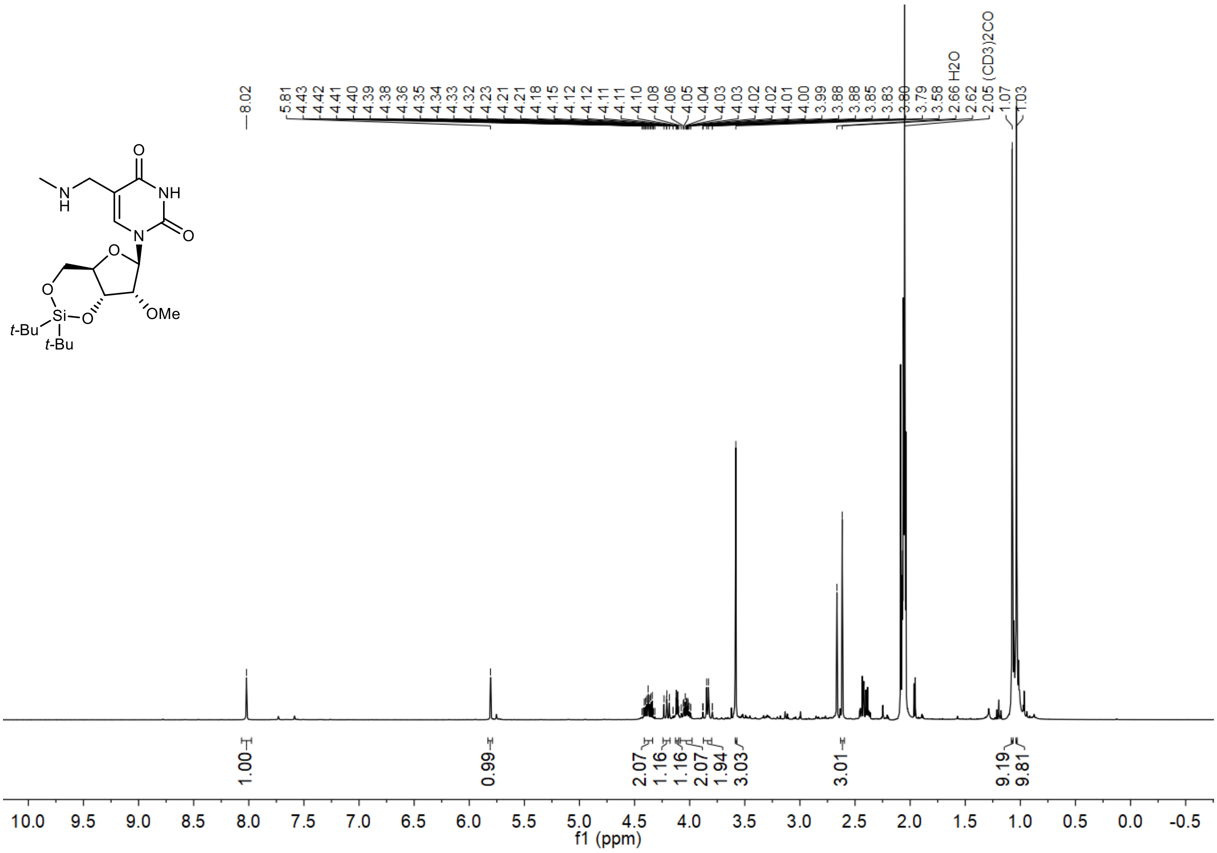

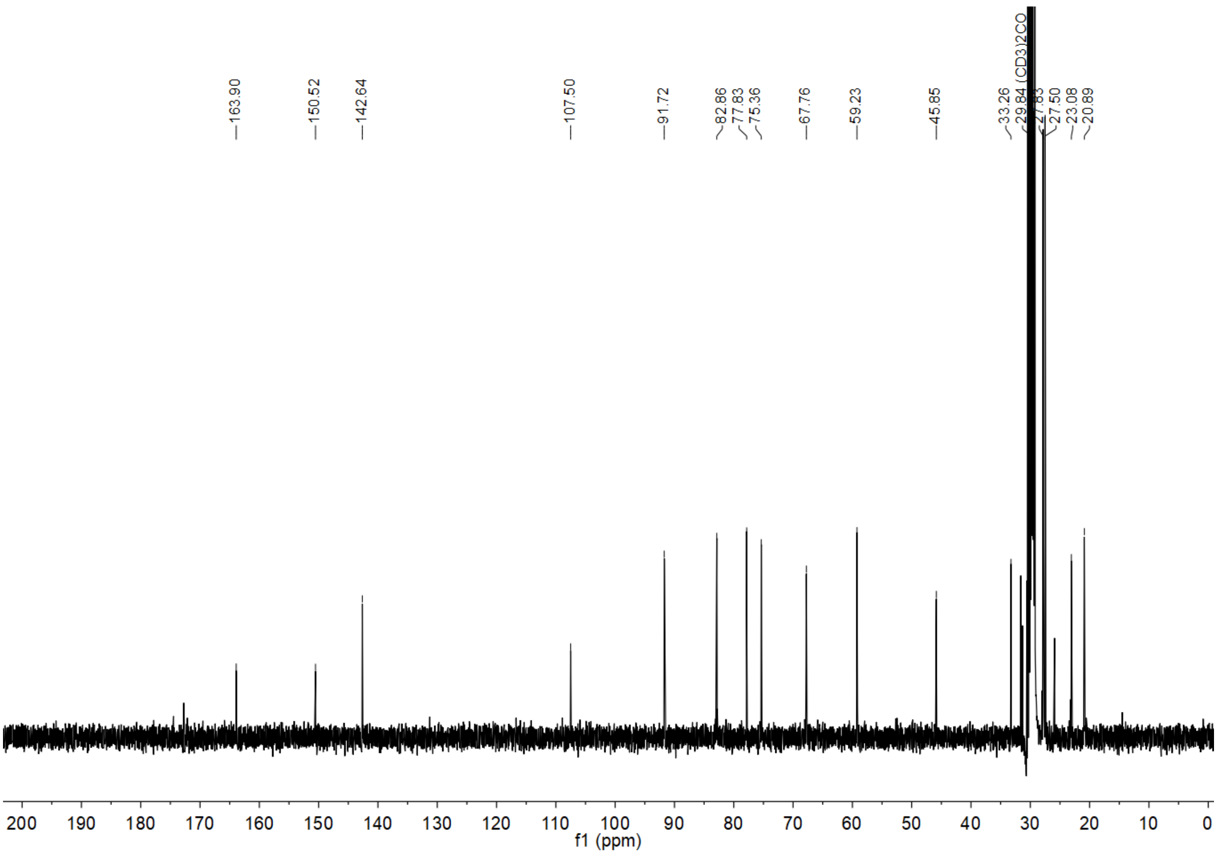

^{1}H and ^{13}C{^{1}H} NMR spectra of compound 23

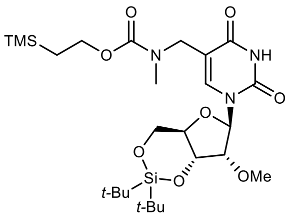

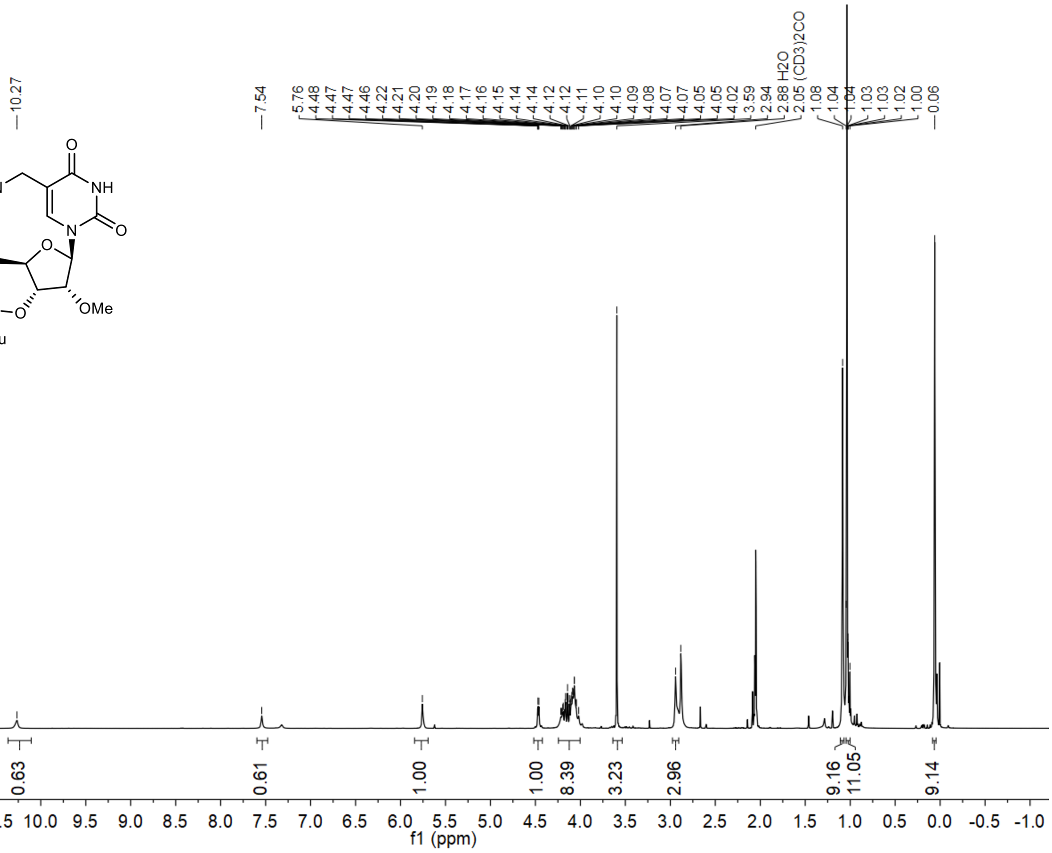

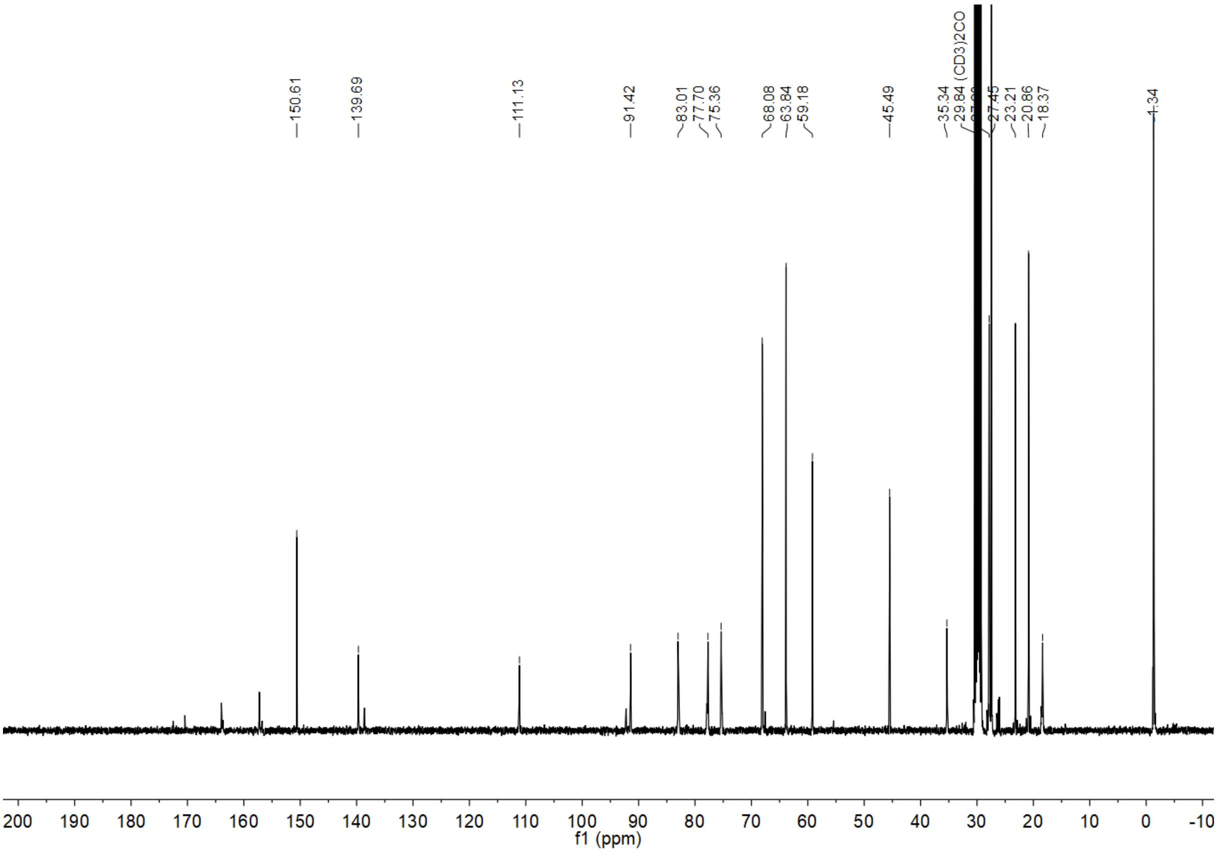

^{1}H and ^{13}C{^{1}H} NMR spectra of compound 24

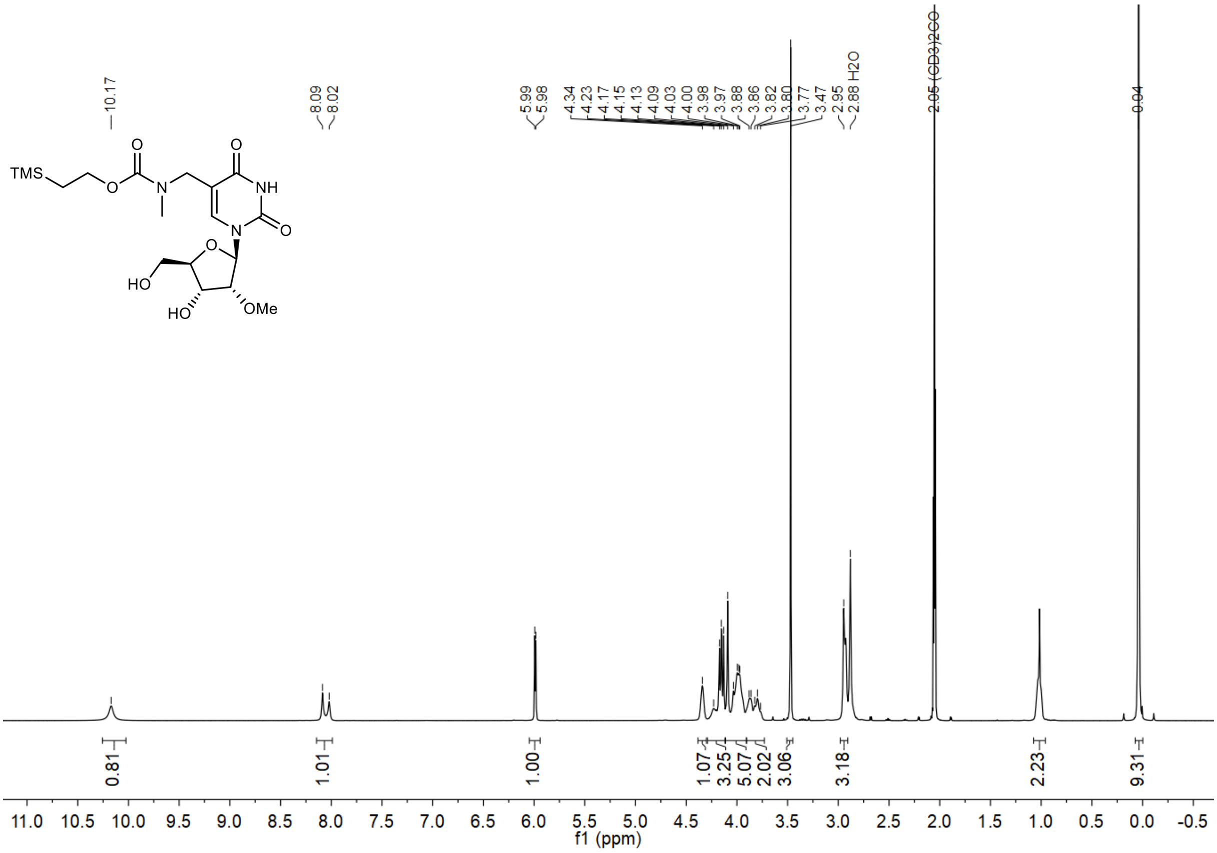

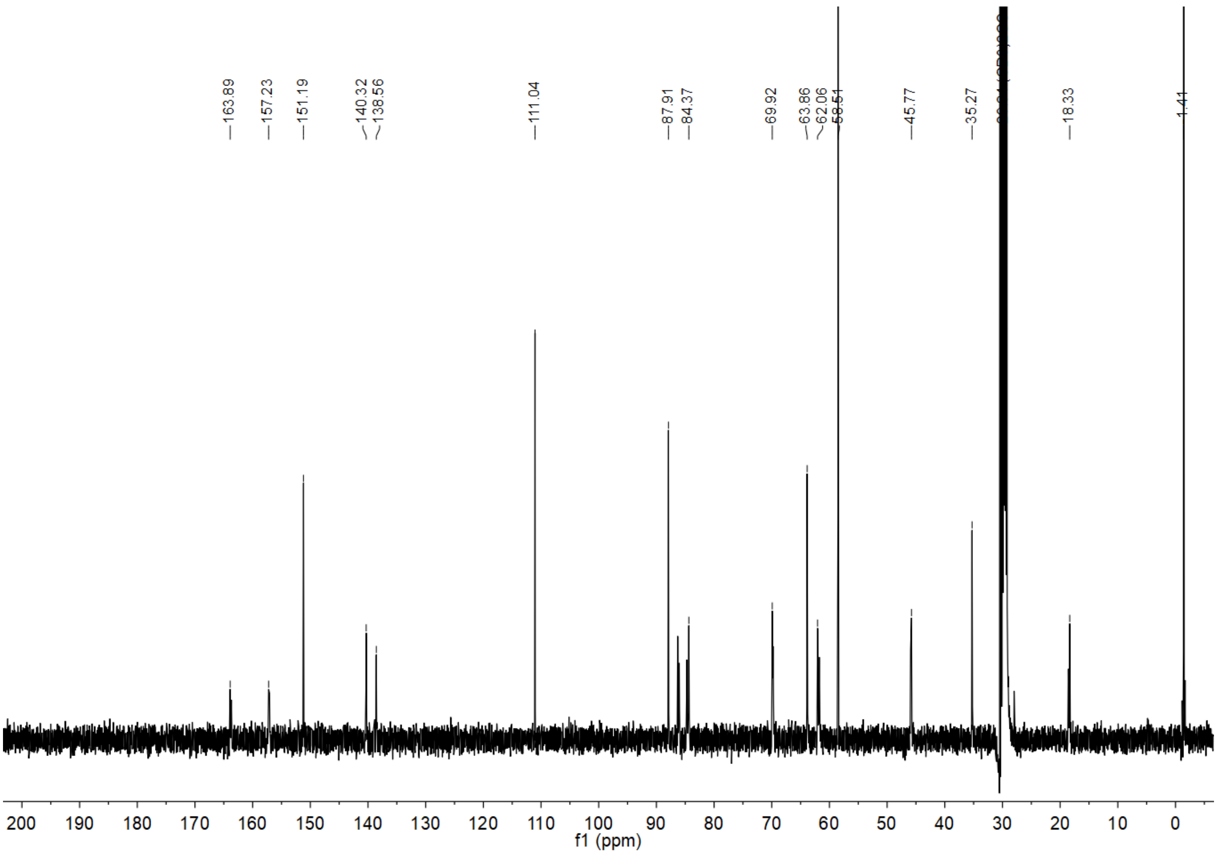

^{1}H and $^{13}C\{^{1}H\}$ NMR spectra of compound 25

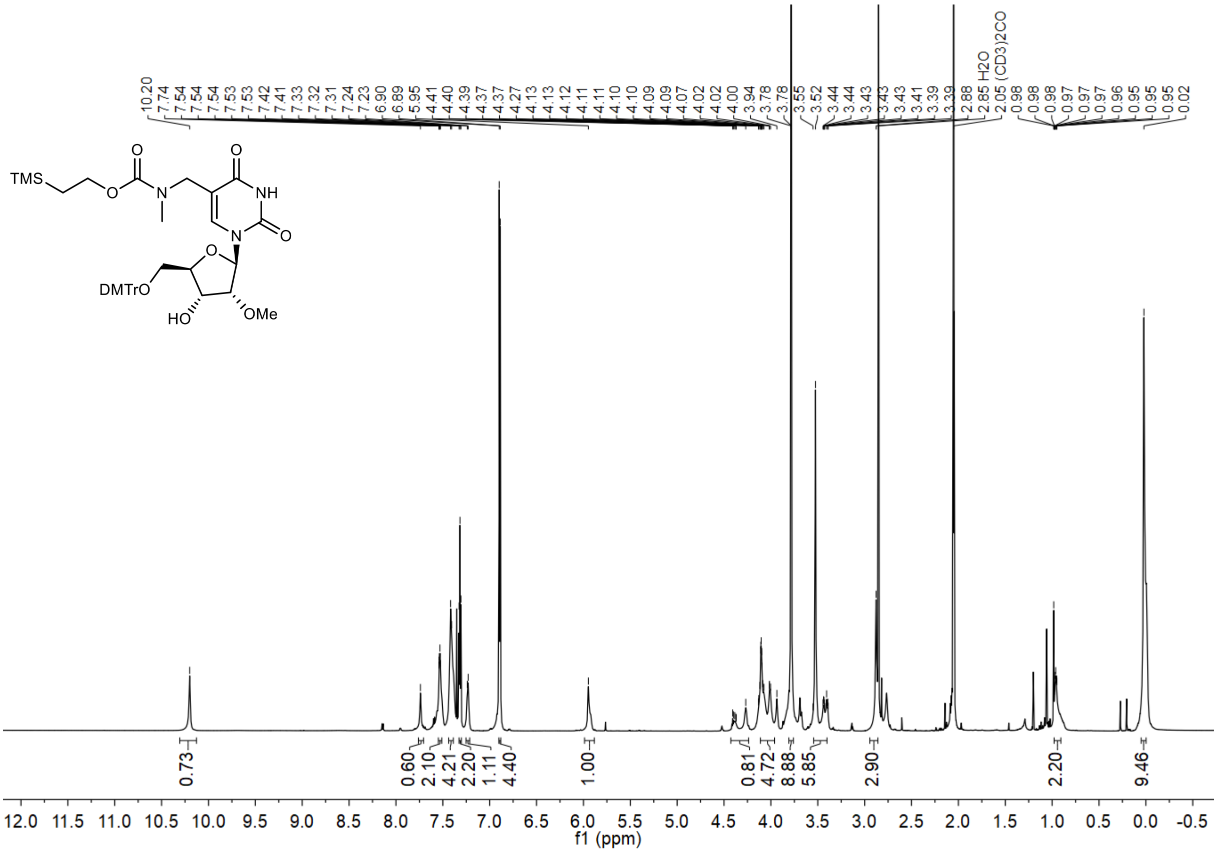

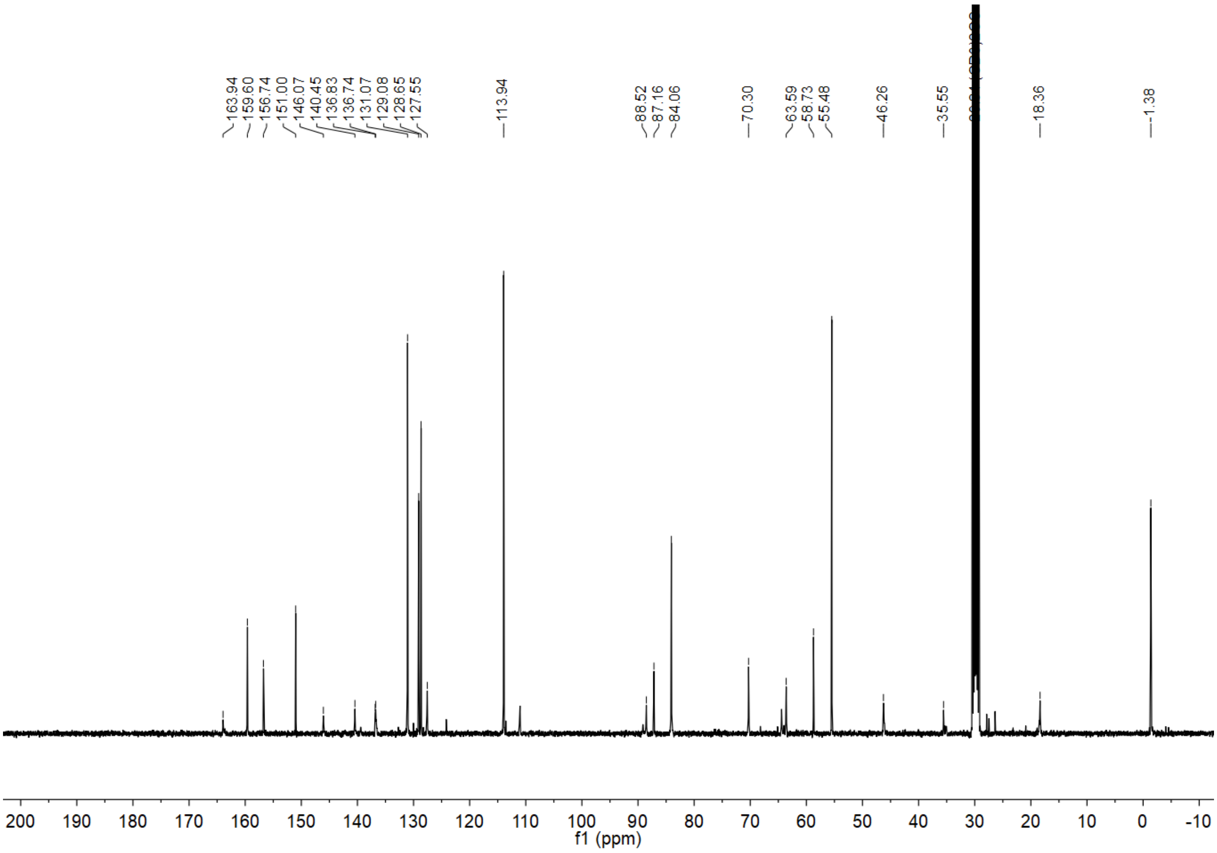

$^{31}P\{^{1}H\}$ NMR spectrum of compound 26

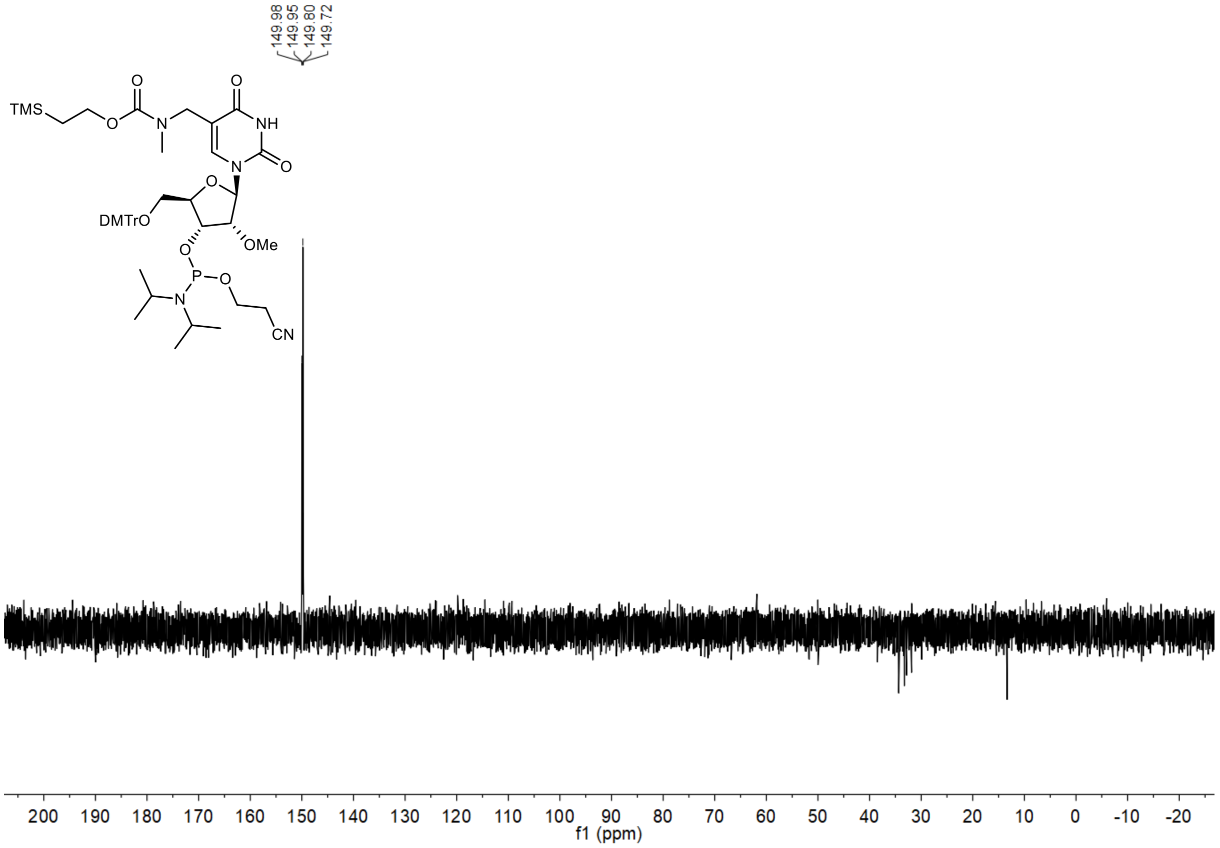

^{1}H and ^{13}C{^{1}H} NMR spectra of compound 28

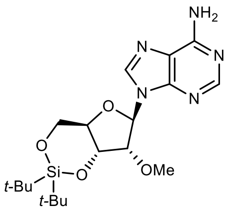

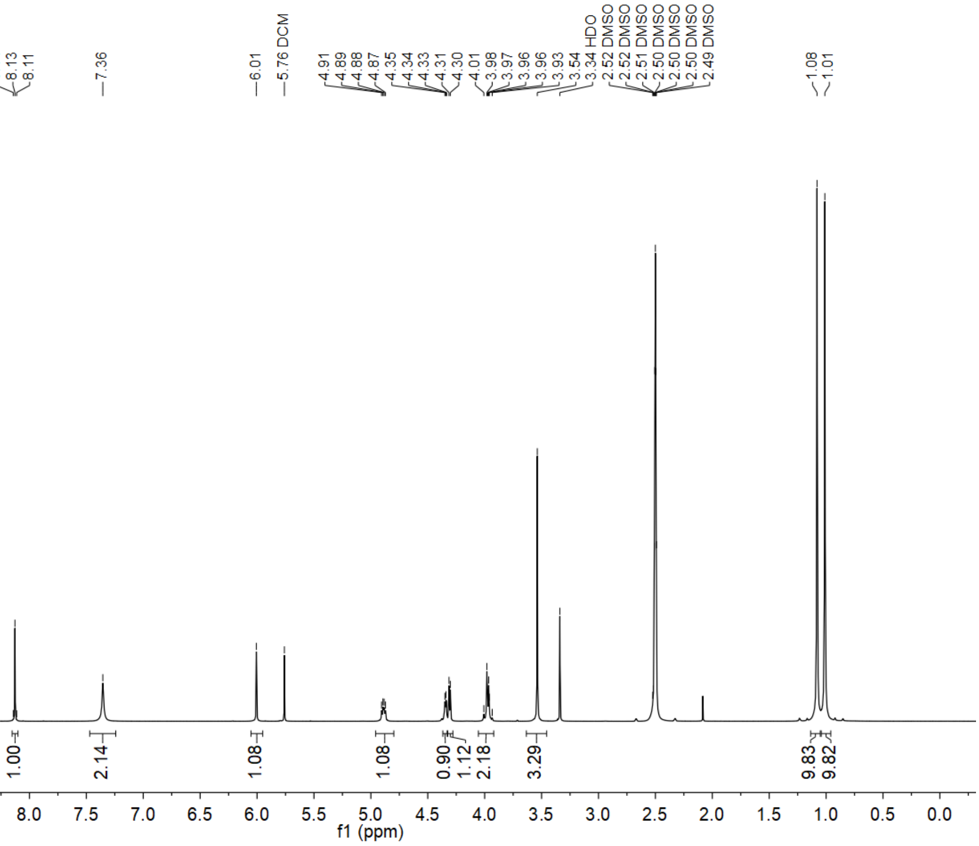

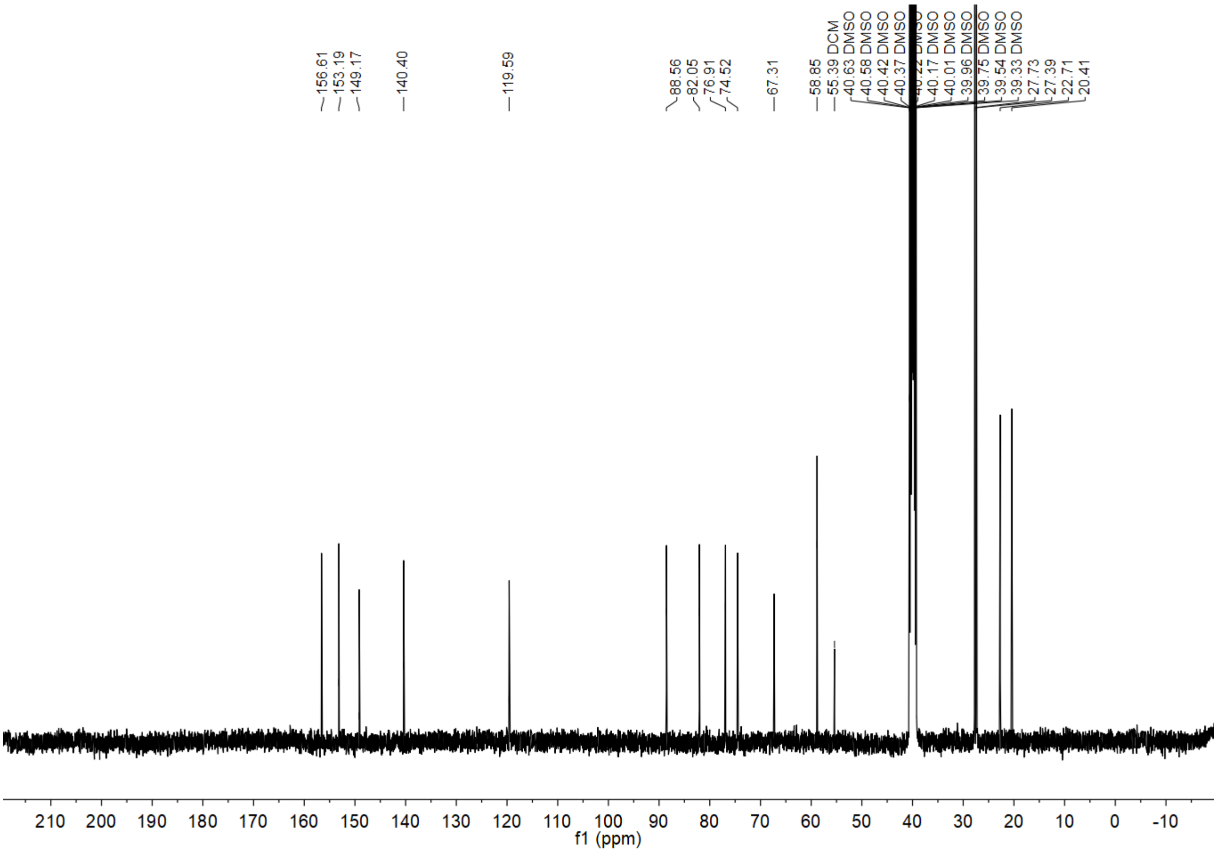

^{1}H and ^{13}C{^{1}H} NMR spectra of compound 29

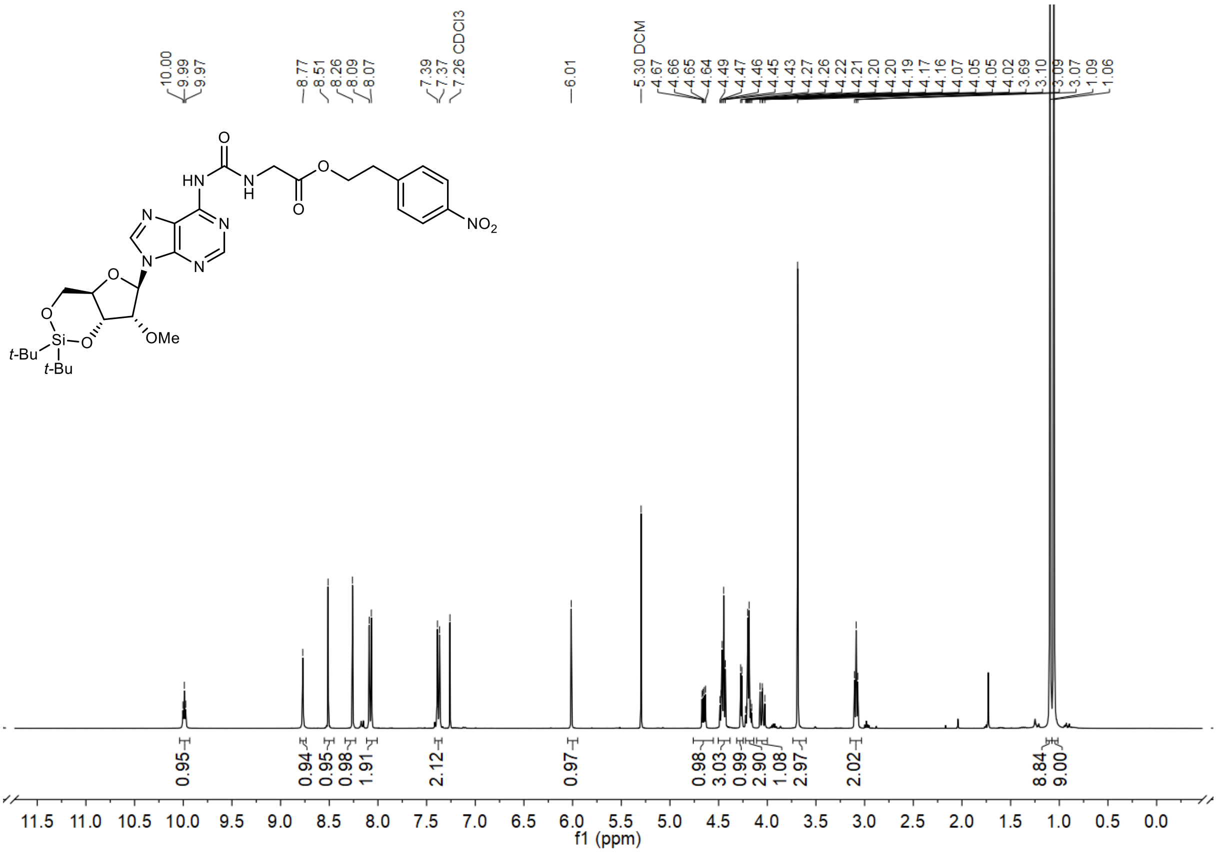

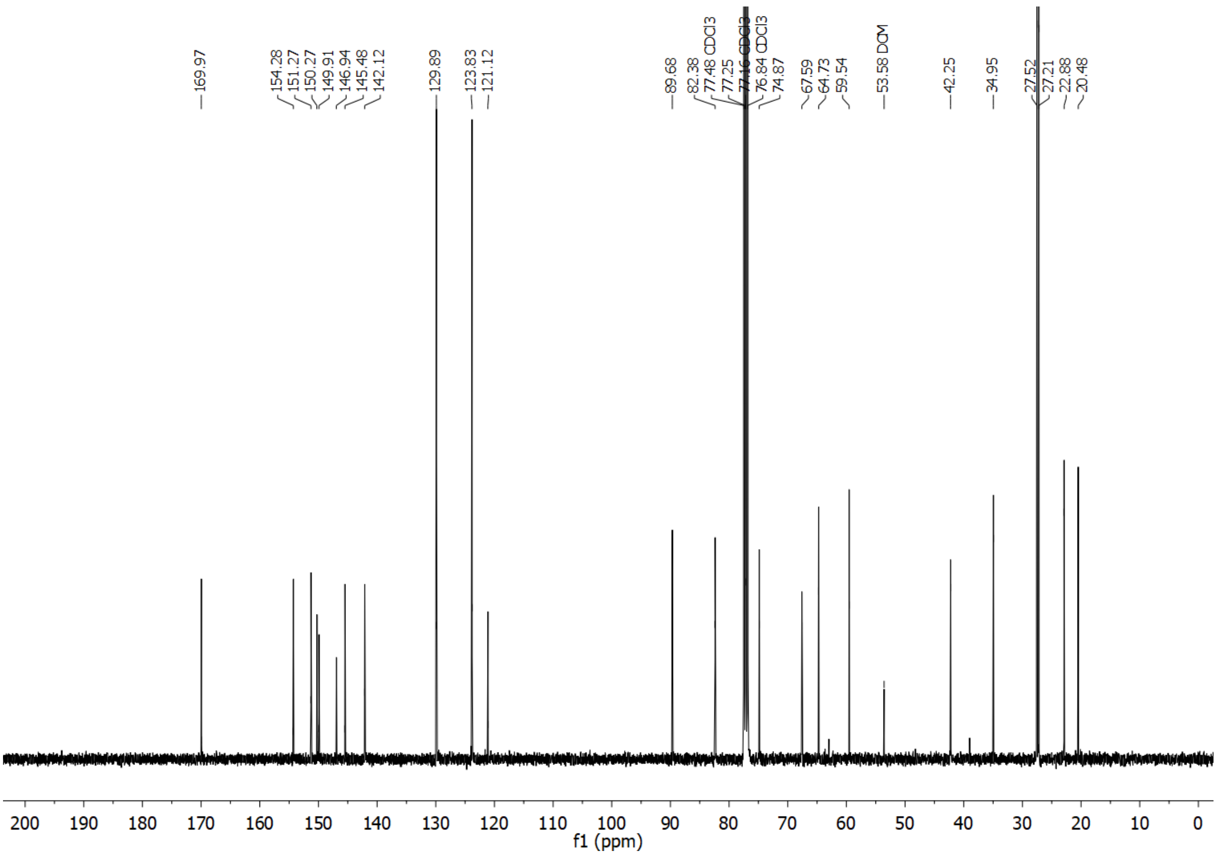

^{1}H and ^{13}C{^{1}H} NMR spectra of compound 30

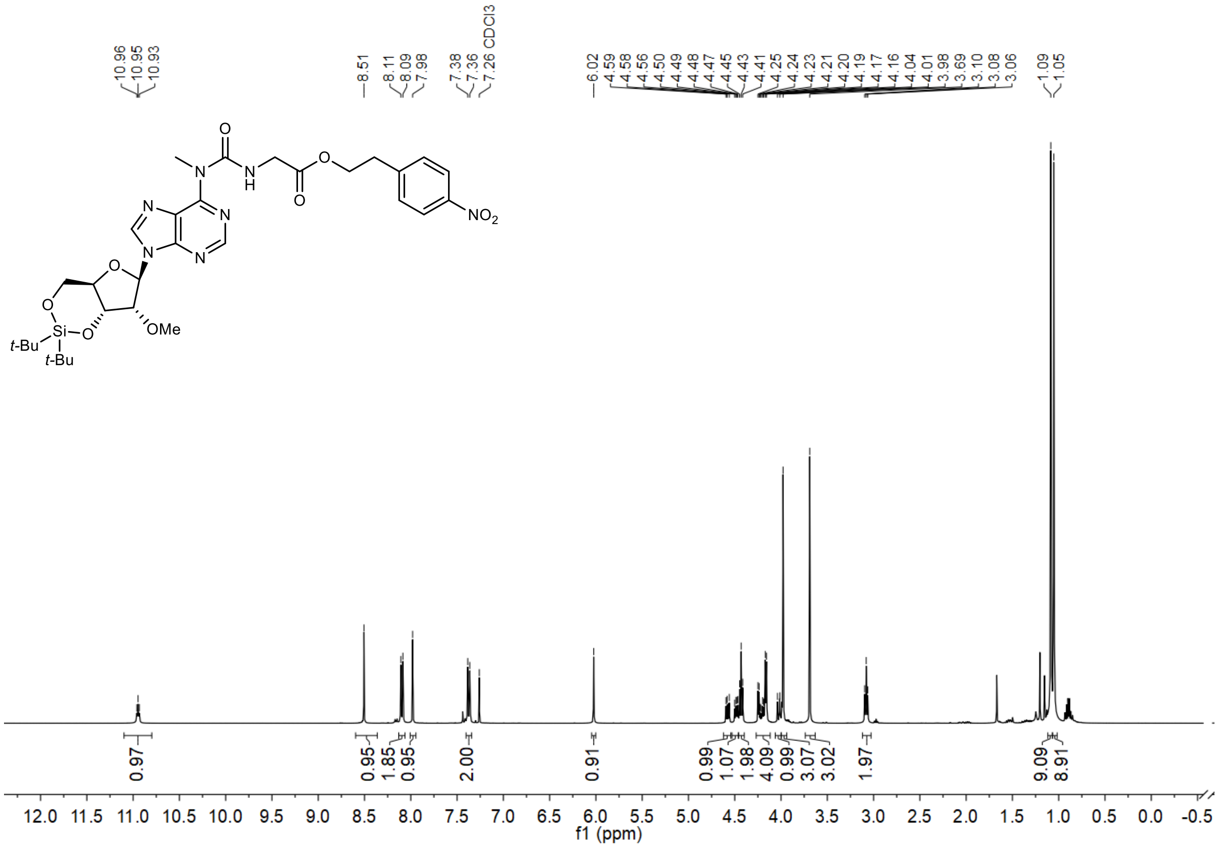

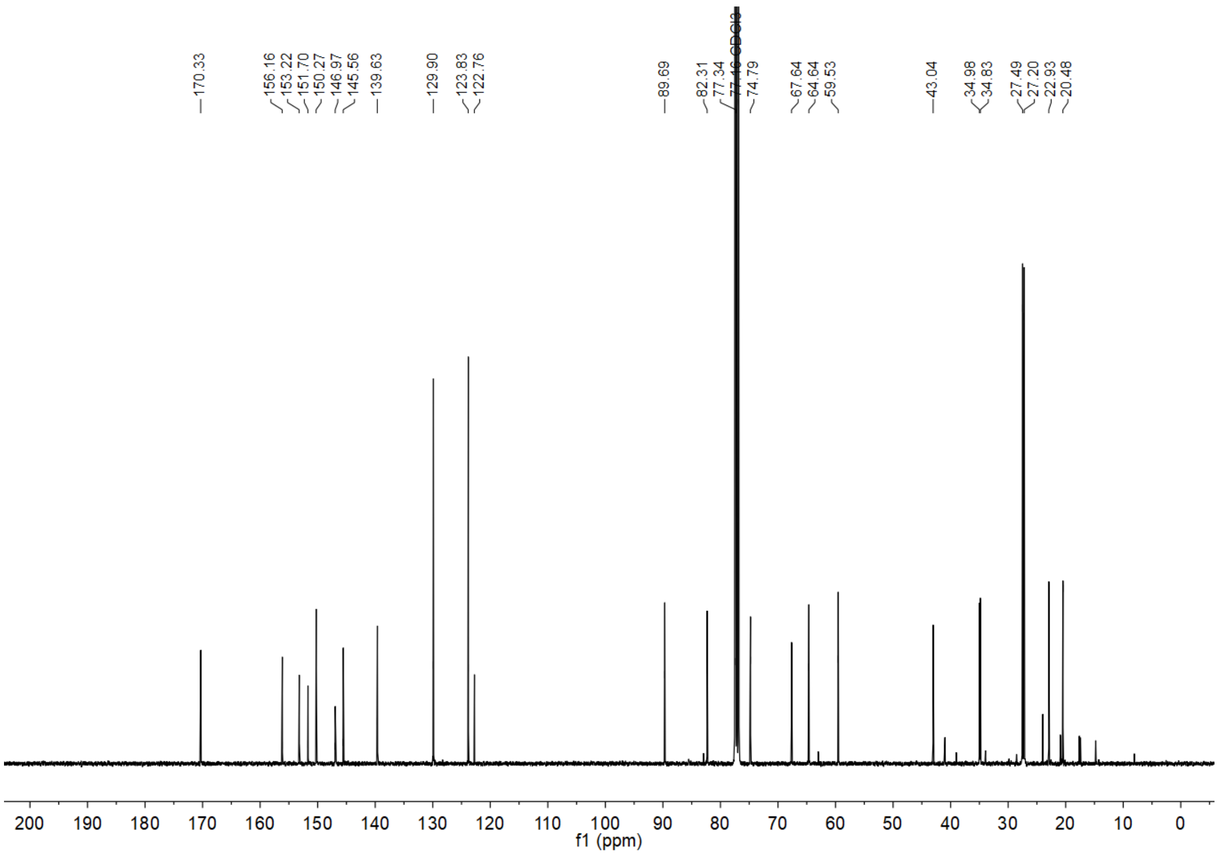

^{1}H and ^{13}C{^{1}H} NMR spectra of compound 31

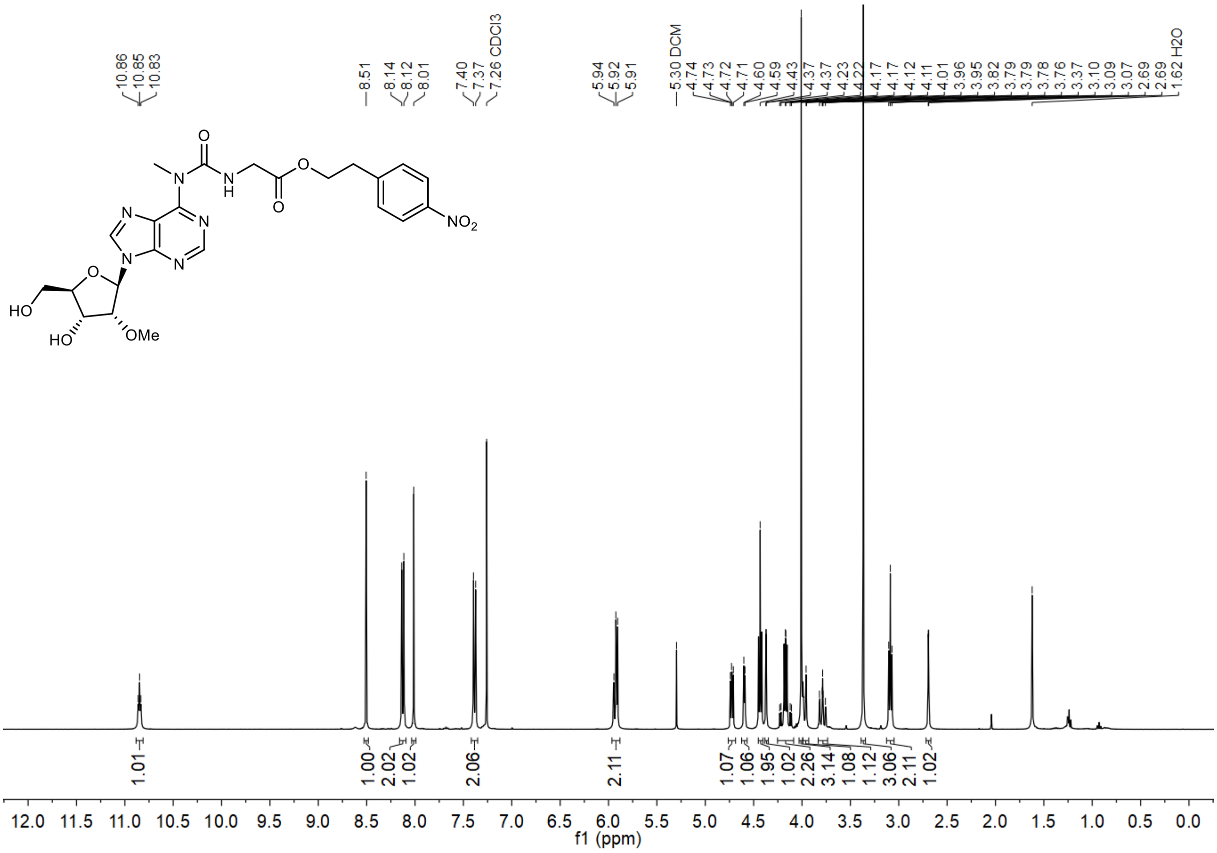

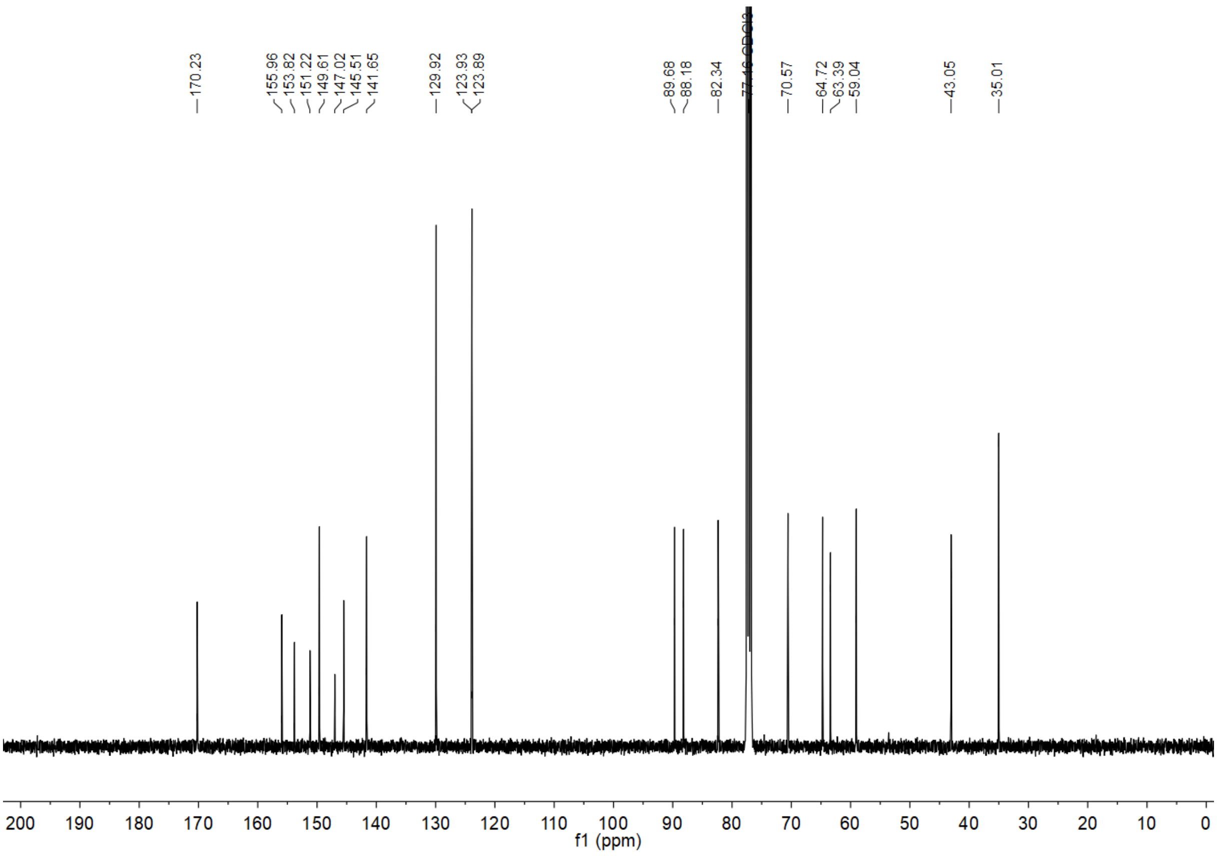

^{1}H and ^{13}C{^{1}H} NMR spectra of compound 32

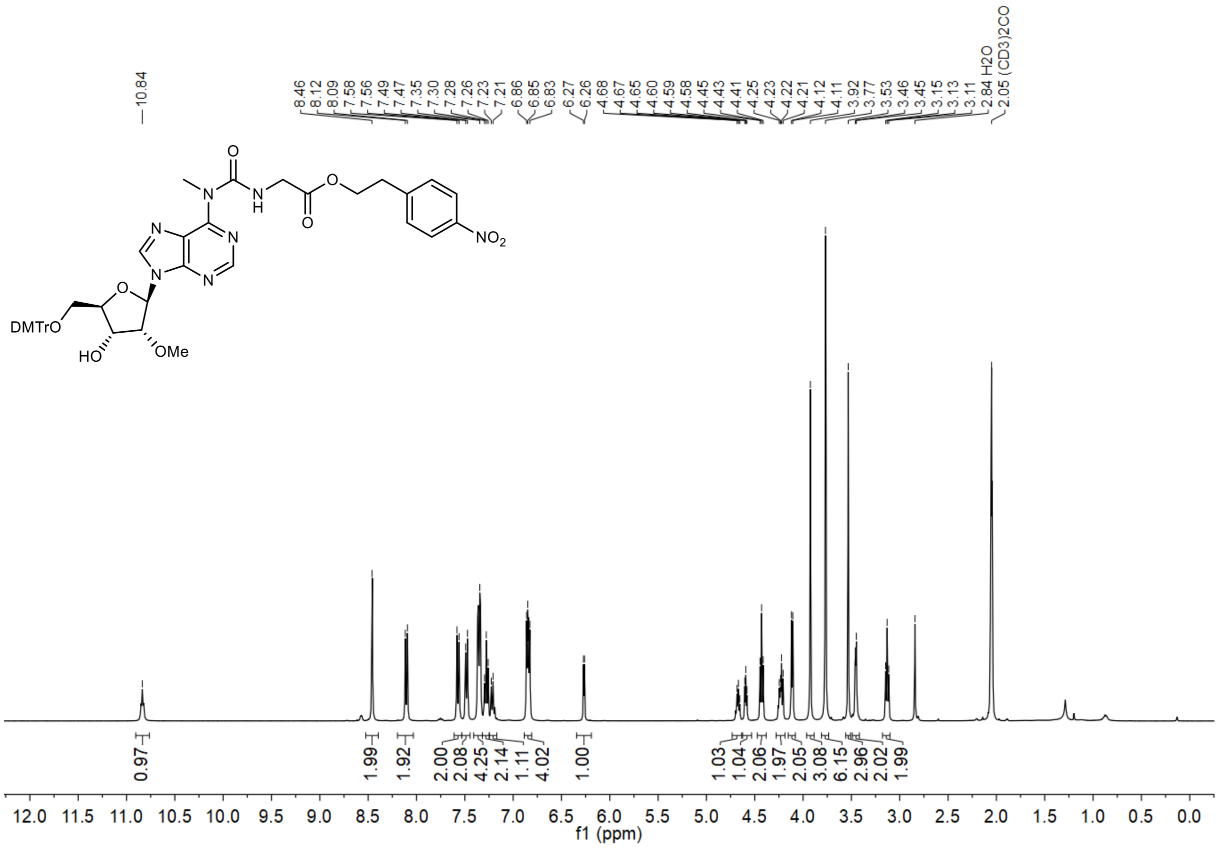

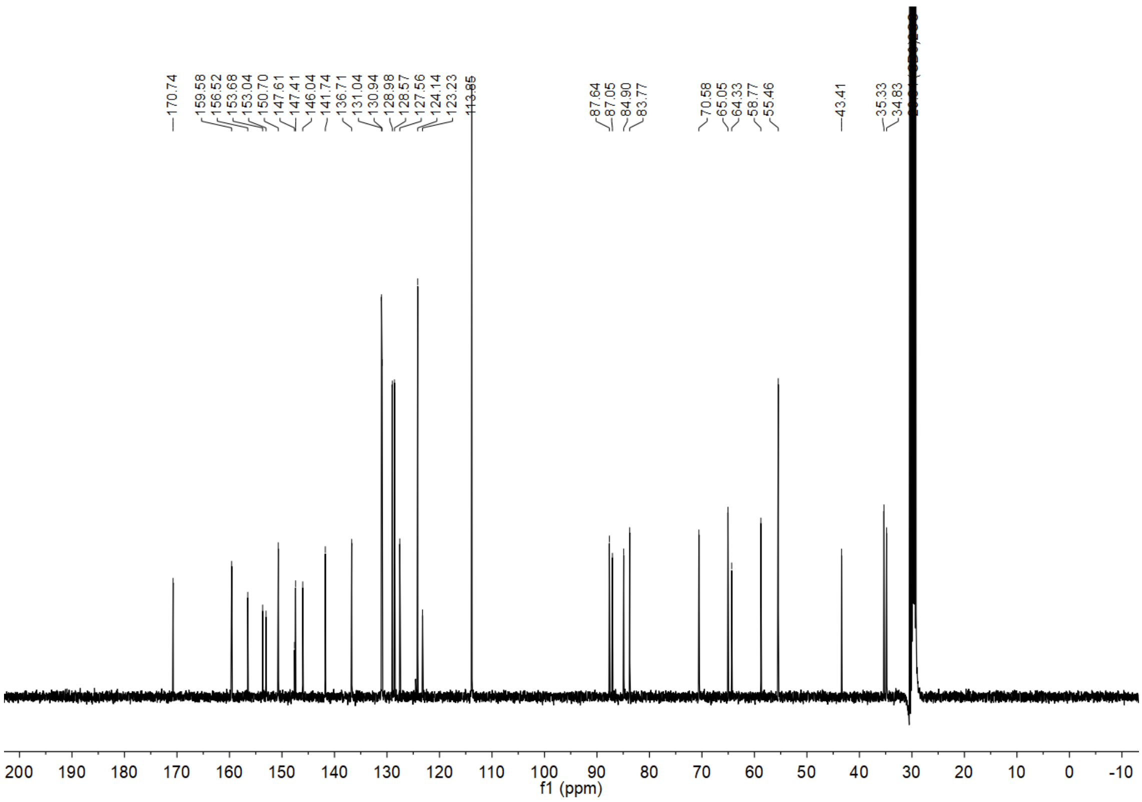

$^{31}P\{^1H\}$ NMR spectrum of compound 33

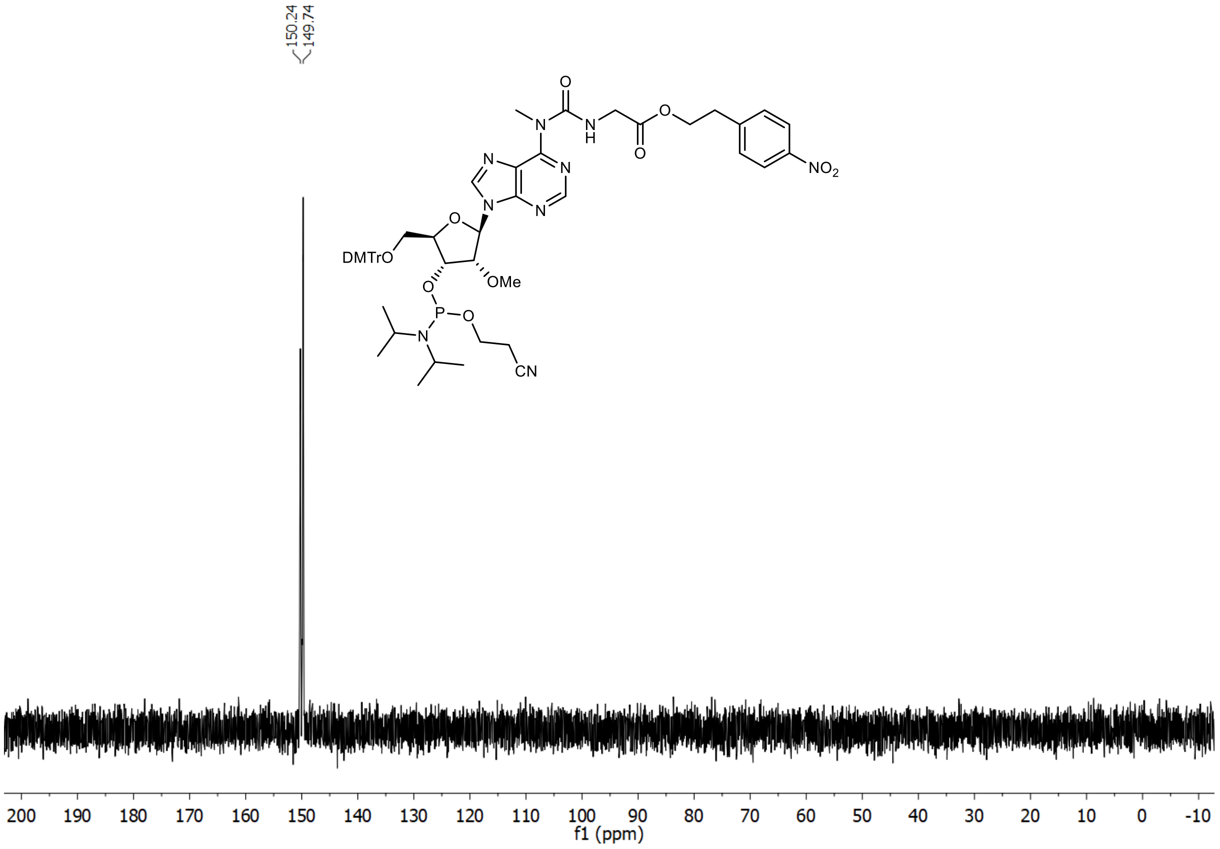

18. References

1. Fulmer, G. R. *et al.* NMR Chemical Shifts of Trace Impurities: Common Laboratory Solvents, Organics, and Gases in Deuterated Solvents Relevant to the Organometallic Chemist. *Organometallics* **29**, 2176-2179 (2010).

2 . Tanpure, A. A. & Balasubramanian, S. Synthesis and Multiple Incorporations of 2′-*O*-Methyl-5-hydroxymethylcytidine, 5-Hydroxymethylcytidine and 5-Formylcytidine Monomers into RNA Oligonucleotides. *ChemBioChem* **18**, 2236-2241 (2017).

3. Shute, R. E. & Rich, D. H. Synthesis and Evaluation of Novel Activated Mixed Carbonate Reagents for the Introduction of the 2-(Trimethylsilyl)ethoxycarbonyl(Teoc)-Protecting Group. *Synthesis* **1987**, 346-349 (1987).

4. Nainytė, M. *et al.* Amino Acid Modified RNA Bases as Building Blocks of an Early Earth RNA-Peptide World. *Chem. Eur. J.* **26**, 14856–14860 (2020).

5. Serebryany, V. & Beigelman, L. An efficient preparation of protected ribonucleosides for phosphoramidite RNA synthesis. *Tetrahedron Lett.* **43**, 1983-1985 (2002).

6. Sundaram, M., Crain, P. F. & Davis, D. R. Synthesis and Characterization of the Native Anticodon Domain of *E. coli* $tRNA^{Lys}$: Simultaneous Incorporation of Modified Nucleosides mnm^5s^2U, t^6A, and Pseudouridine Using Phosphoramidite Chemistry. *J. Org. Chem.* **65**, 5609-5614 (2000).

7 . Matuszewski, M. & Sochacka, E. Stability studies on the newly discovered cyclic form of tRNA N^6-threonylcarbamoyladenosine (ct^6A). *Bioorg. Med. Chem. Lett.* **24**, 2703-2706 (2014).

8. Schneider, C. *et al.* Noncanonical RNA Nucleosides as Molecular Fossils of an Early Earth—Generation by Prebiotic Methylations and Carbamoylations. *Angew. Chem. Int. Ed.* **57**, 5943-5946 (2018).

9. Himmelsbach, F., Schulz, B. S., Trichtinger, T., Charubala, R. & Pfleiderer, W. The *p*-Nitrophenylethyl (NPE) Group: A Versatile New Blocking Group for Phosphate and Aglycone Protection in Nucleosides and Nucleotides. *Tetrahedron* **40**, 59-72 (1984).

10. Ferreira, F. & Morvan, F. Silyl Protecting Groups for Oligonucleotide Synthesis Removed by a $ZnBr_2$ Treatment. *Nucleosides, Nucleotides, and Nucleic Acids* **24**, 1009-1013 (2005).

11. Usanov, D. L., Chan, A. I., Maianti, J. P. & Liu, D. R. Second-generation DNA-templated macrocycle libraries for the discovery of bioactive small molecules. *Nat. Chem.* **10**, 704-714 (2018).

12. Hoops, S. *et al.* COPASI—a COmplex PAthway SImulator. *Bioinformatics* **22**, 3067-3074 (2006).

13. Jash, B., Tremmel, P., Jovanovic, D. & Richert, C. Single nucleotide translation without ribosomes. *Nat. Chem.* **13**, 751-757 (2021).

14. Mochrie, S. G. J. The Boltzmann factor, DNA melting, and Brownian ratchets: Topics in an introductory physics sequence for biology and premedical students. *Am. J. Phys* **79**, 1121-1126 (2011).

15. Senior, M. M., Jones, R. A. & Breslauer, K. J. Influence of loop residues on the relative stabilities of DNA hairpin structures. *Proc. Natl. Acad. Sci. U. S. A.* **85**, 6242-6246 (1988).

16. Xodo, L. E., Manzini, G., Quadrifoglio, F., Marel, G. v. d. & van Boom, J. H. Hairpin structures in synthetic oligodeoxynucleotides: sequence effects on the duplex-to-hairpin transition. *Biochimie* **71**, 793-803 (1989).
